# Exploring and Re-Assessing Reverse Anomeric Effect in 2-Iminoaldoses Derived from Mono- and Polynuclear Aromatic Aldehydes

**DOI:** 10.3390/molecules29174131

**Published:** 2024-08-30

**Authors:** Esther M. S. Pérez, Esther Matamoros, Pedro Cintas, Juan C. Palacios

**Affiliations:** 1 Department of Organic and Inorganic Chemistry, Faculty of Sciences, and IACYS—Green Chemistry and Sustainable Development Unit, University of Extremadura, 06006 Badajoz, Spain; 2 Departamento de Química Orgánica, Universidad de Málaga, 29071 Málaga, Spain; 3 Instituto de Investigación Biomédica de Málaga y Plataforma en Nanomedicina—IBIMA, Plataforma Bionand, Parque Tecnológico de Andalucía, 29590 Málaga, Spain

**Keywords:** anomeric effects, 2-iminoaldoses, reverse anomeric effect, Schiff bases, spectroscopic characterization, computational chemistry

## Abstract

A curious and noticeable structural feature in Schiff bases from 2-aminoaldoses is the fact that imino tautomers arranged equatorially in the most stable ring conformation exhibit a counterintuitive reverse anomeric effect (RAE) in the mutarotational equilibrium, i.e., the most stable and abundant anomer is the equatorial one (β). As shown by our very recent research, this effect arises from the total or partial inhibition of the exo-anomeric effect due to the presence of an intramolecular hydrogen bond between the anomeric hydroxyl and the iminic nitrogen in the axial anomer (α). When the Schiff base adopts either an enamine structure or the imino group is protonated, the exo-anomeric effect is restored, and the axial α-anomer becomes the most stable species. Although the intramolecular H-bonding should appropriately be interpreted as a genuine stereoelectronic effect, the magnitude of the RAE could be affected by other structural parameters. Herein and through a comprehensive analysis of benzylidene, cinnamylidene, naphthalene, phenanthrene, and anthracene aldehydes, we show the robustness of the RAE effect, which is similar in extent to simple aldehydes screened so far, irrespective of the size and/or hydrophobicity of the substituent at the nitrogen atom.

## 1. Introduction

Imines or Schiff bases constitute privileged scaffolds dating back to the early days of synthetic organic chemistry that can easily be generated by condensation of carbonyl groups and primary amines. This transformation takes place through the intermediacy of a carbinolamine that undergoes further dehydration, leading to a double carbon-nitrogen bond [1]. Over the years, imines derived from carbohydrates have been extensively studied in view of a broad range of applications, such as recognition of naturally occurring amino acids using fluorescence and absorption measurements. Titration of d-glucosamine salicylidenimine (**1**) with all of the 20 naturally occurring amino acids resulted in large fluorescence enhancements in the case of aromatic amino acids only, thus enabling the recognition of such amino acids down to 1.5–3 ppm through switch-on fluorescence behavior [2,3]. Sugar imines and l-amino acids self-assemble by generating 1:1 hydrogen-bonded complexes and forming amphiphilic nanofibers through π–π interactions [4], although other non-covalent interactions may be involved as well. The recognition of M^2+^ ions in solution and selective recognition of Cu^2+^ in HEPES (a zwitterionic sulfonic acid) buffer are based on the formation of glyco-imino-conjugates [5,6]. On the other hand, several imino conjugates of aldoses and d-glucosamine (**2**), which are transition state analogues, are potent inhibitors of glycosidases extracted from soybean and jack bean meal [7].

**Chart 1:**
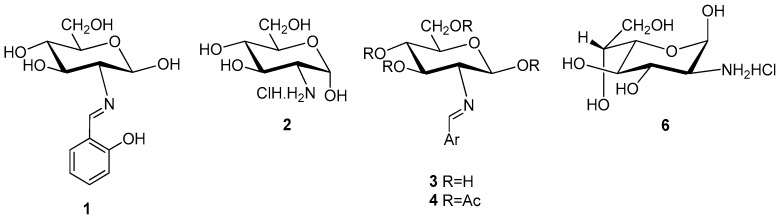
Structures of compounds **1**–**6**.

The first imine derived from 2-amino-2-deoxyaldoses (**1**) was reported in as early as 1913 [8,9,10]. Later on, Wacker and Fritz in 1967 [11] and Panov et al. in 1973 [12] prepared a series of imines (**3**) derived from 2-amino-2-deoxy-d-glucopyranose (**2**) with benzaldehydes and their per-*O*-acetyl derivatives (**4**), and demonstrated by ^1^H-NMR spectroscopy the appearance of equatorial anomers (β). In fact, with only one exception, all the known Schiff bases obtained from **2** and substituted benzaldehydes devoid of hydroxyl groups at the *ortho* position crystallize as β-anomers [13] (Chart 1). Certainly, this is a surprising and unexpected behavior because, in general, other aminoaldose derivatives show an axial stereochemistry (α-anomer), as a consequence of the anomeric effect.

**Scheme 1:**
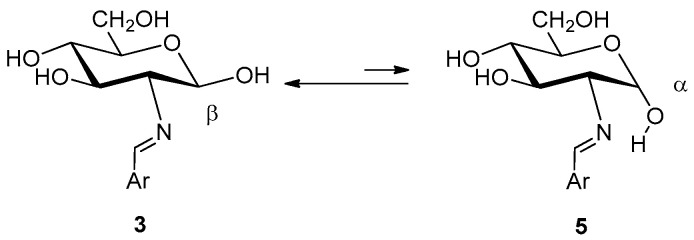
Reverse anomeric effect in 2-arylimino aldoses.

For imines of 2-aminoaldoses in solution, however, an equilibrium between α- and β-anomers can be detected where the latter largely predominates (Scheme 1). This behavior can be regarded in terms of a reverse anomeric effect (RAE), with values in the range of 1.9–2.3 kcal/mol. This stabilization of the equatorial anomer neutralizes and exceeds the anomeric effect. Theoretical calculations show that this stereoelectronic effect results from the reduction (or elimination) of the stabilizing exo-anomeric effect in the axial anomer (**5**), owing to the formation of a hydrogen bond between the anomeric hydroxyl and the imine nitrogen. Moreover, solvent effects (modeled as discrete solvation) support the preferential formation of the equatorial anomer (β) [13]. The present work sheds light into the influence exerted by the aromatic residue on the anomeric effect through a full set of spectral analyses in solution and computational assessment.

## 2. Results and Discussion

### 2.1. Synthesis of 2-Amino-2-Deoxyaldose Imines

**Chart 2:**
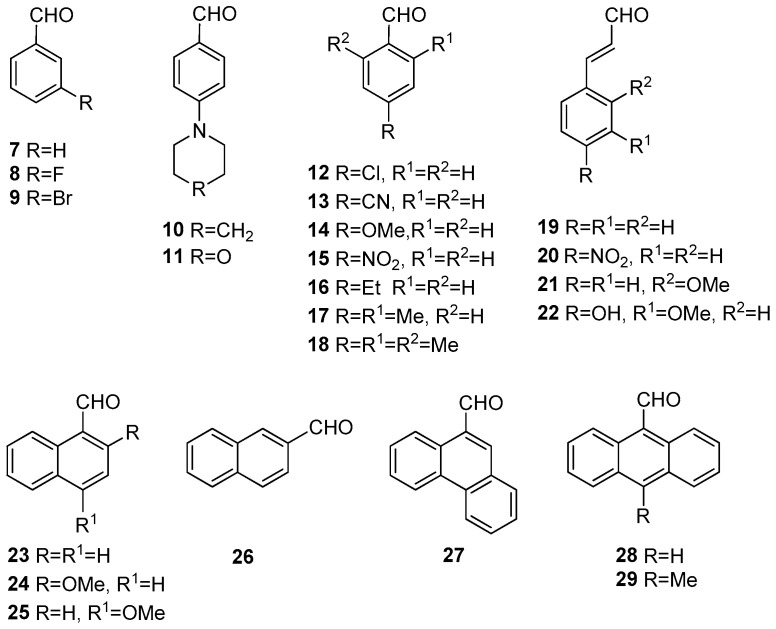
Aldehydes used in the preparation of imines.

We employed as starting aminoaldoses the hydrochlorides of d-glucosamine (**2**) and 2-amino-2-deoxy-α-d-*glycero*-l-*gluco*-heptopyranose (**6**) [14,15,16], which were condensed with aldehydes derived from aliphatic and mono- and polynuclear aromatic hydrocarbons (Chart 2). Thus, the present study involves new Schiff bases obtained by reaction of benzaldehydes (**7**–**18**) with **2** and heptose **6**, because for the latter, only the heptosimine derivative **37** had been described [17]. Since the lone electron pair on the nitrogen atom lies in the nodal plane of the unsaturated arylimino fragment, the substituents at the aromatic ring can only exert inductive electronic effects. Therefore, special interest has been paid to benzaldehydes (**7**–**9**, **12**, **13**) with strong electron-withdrawing groups (EWG), which would decrease the basicity of the nitrogen atom and weaken the hydrogen bond with the anomeric hydroxyl. On the other hand, the presence of electron-donating groups (EDG) would exert the opposite effect (**10** and **11**).

Also, the structural variation has been extended to imines derived from cinnamylidene aldehydes (**19**–**22**), in which the ethylene linker separates the bulky aromatic ring from the sugar moiety, which could alleviate the steric hindrance while facilitating the transmission of electronic effects (i.e., the classical *vinylogy principle*). Furthermore, imines derived from naphthalene, phenanthrene, and anthracene aldehydes have been obtained, which lack a hydroxyl group adjacent to the aldehyde group (**23**–**29**). These compounds allow us to evaluate the potential steric effects associated with their volume, along with the influence of increasing the hydrophobicity of the iminic functionality on the tautomeric equilibrium.

**Chart 3:**
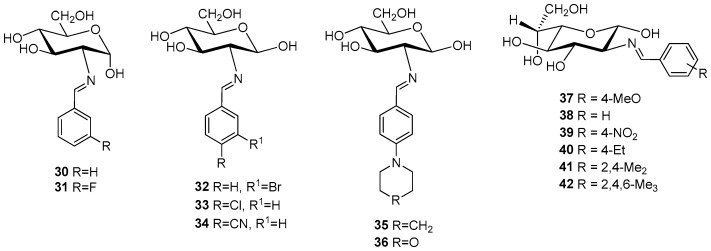
Imines from **2** and **6** with substituted benzaldehydes.

Thus, the condensation of **2** with benzaldehydes **7**–**13** afforded the corresponding imines **30**–**36**. Like all imines of **2** described to date, **32**–**36** crystallize as β-anomers, and **30** and **31** as α-anomers nevertheless. When using 2-amino-2-deoxy-α-d-*glycero*-l-*gluco*-heptopyranose hydrochloride (**6**), the β-configured imines **37**–**41** were obtained (Chart 3). The behavior of this aminoheptose runs parallel to that of **2**, since all chiral carbons that make up the pyranose ring show an enantiomeric relationship (l-*gluco*).

**Scheme 2:**
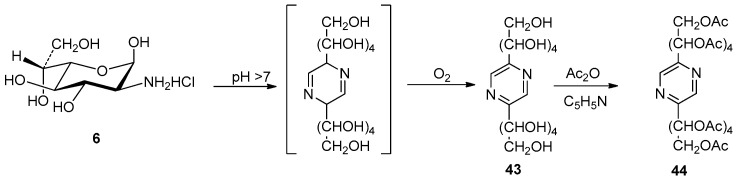
Generation of substituted pyrazines from **6** under basic conditions.

When 2,4,6-trimethylbenzaldehyde was employed, the corresponding imine **42** was not obtained; instead, 2,5-*bis*-(d-*galacto*-pentitol-1-yl)pyrazine (**43**) was isolated, the latter formed by self-condensation of **6** in basic medium (Scheme 2). This substance was characterized through its deca-*O*-acetyl derivative **44**, prepared by conventional acetylation. The generation of this type of heterocyclic compound from alkaline solutions of d-glucosamine, d-mannosamine and d-fructosamine is well documented in the previous literature [18].

**Chart 4:**
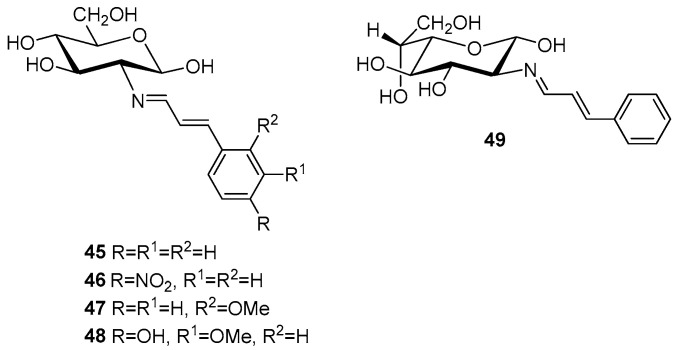
Imines synthetized from cinnamylidene aldehydes.

By using the cinnamylidene aldehydes **19**–**22**, the corresponding β-anomers of Schiff bases **45**–**49** could be obtained as well (Chart 4). Compounds **45** [19], **46** [20], and **48** [20] were previously described.

**Chart 5:**
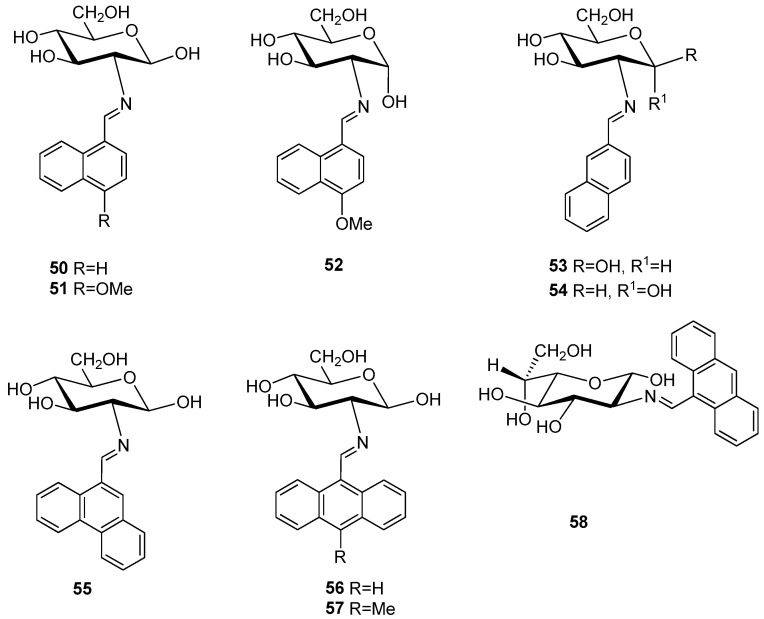
Imines derived from polynuclear arylaldehydes.

In striking contrast, the condensations of **2** with **23**–**26** led to different results depending on the starting aldehyde (Chart 5). With 1-naphthaldehyde, the β-configured imine was obtained (**50**). However, when 4-methoxy-1-naphthaldehyde or 2-naphthaldehyde were used, sometimes the β-anomer (**51** or **53**) and sometimes the α-anomer (**52** or **54**) crystallized. As already shown, no product could be isolated from the condensation with 2-methoxy-1-naphthaldehyde (**24**) [20]. Finally, imines **55**–**58**, all with β-anomeric configuration, were prepared from aldehydes derived from phenanthrene and anthracene (**27**–**29**).

**Scheme 3:**
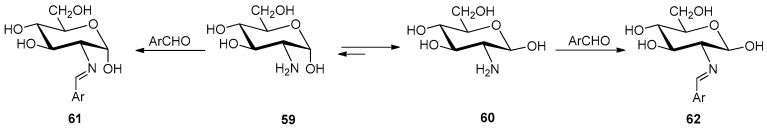
Formation of α and β-anomers of arylimines from **2**.

Clearly, the synthesis of α-anomers **30**, **31**, **52** and **54** is interesting, because only one related case having this abnormal configuration has been described in our previous study, involving the reaction of **2** with **18** [13]. All reactions took place in hydroalcoholic media by treating 2-aminoaldose hydrochlorides with sodium hydroxide or sodium bicarbonate to release the free bases of the α-anomers (**59**). Interconversion then occurs between the two anomers (**59**, **60**), which condense with the aryl aldehyde present (**61**, **62**) (Scheme 3). Reactions are often heated at ~60 °C for a few minutes, because the aromatic aldehyde can be poorly soluble at room temperature.

A preliminary assessment of the above-mentioned examples indicates that, in general, reactions conducted at room temperature with concomitant imine crystallization in short times (less than 15 min) led to α-anomers. In slow reactions taking long (several hours) before crystallization, the β-anomer is usually isolated.

### 2.2. Structural Characterization

In the infrared (FT-IR) spectra of imines **30**–**42** and **50**–**58**, the absorption of the C=N bond at ~1635–1650 cm^−1^ stands out. Both ^1^H and ^13^C NMR spectra support the assigned structures (Tables S1–S6 and S10–S12). Thus, the α-anomeric configuration of **30**, **31**, **52** and **54** is inferred from the low value of *J*_1,2_ (3.8 Hz) and by the downfield shift of H-1 and the upfield shift of the C-1 atom [13], relative to the corresponding signals for β-anomers. The rest of the imines show high coupling constants *J*_1,2_ (~7–9 Hz), consistent in all cases with the β-anomer. In addition, the IR spectra of cinnamylidene derivatives **45**–**49** also show the absorptions arising from the stretching vibration of the ethylene double bond at ~1620 cm^−1^. In the proton spectra, the signals of the iminic proton and those of the ethylene fragment should be mentioned, i.e., a doublet at ~7.1 ppm and double doublet at ~6.9 ppm. The large coupling constants between such protons (*J*_CH=CH_~16 Hz) indicate that the stereochemistry around the carbon double bond is *trans* (*E*). The high coupling constants *J*_1,2_ (~8.6 Hz) and the chemical shift of the anomeric carbon (δ_C1_~95 ppm) point to an equatorial (β) disposition of the anomeric hydroxyl in all cases (Tables S7–S9).

β-Imines **32**–**36**, **45**–**48**, **50**, **51**, **53** and **55**–**57** show coupling constants between the proton and the anomeric carbon (^1^*J*), measured in the coupled ^13^C NMR spectra, of ~160 Hz [21,22,23,24,25]. For pyranoid derivatives of d-glucosamine, β-anomers show values of ^1^*J*_C1-H1_~160 Hz, while this value increases to ~170 Hz for α-anomers. These considerations also apply to the derivatives of 2-amino-2-deoxy-d-*glycero*-l-*gluco*-heptopyranose (**37**–**42**, **49** and **58**), due to the enantiomorphous relationship among the chiral centers of its pyranose ring (l-*gluco*) and those of **2** (d-*gluco*). Moreover, α-anomers show higher rotational powers (typically, [α]_D_ > +100° in pyridine) than β-anomers (typically, [α]_D_ < +50° in pyridine).

**Chart 6:**
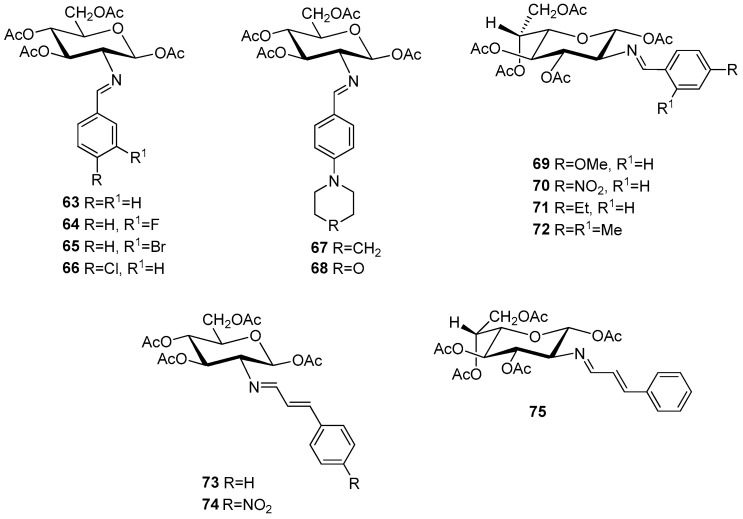
Per-*O*-acetyl imines from benzaldehydes and cinnamaldehydes.

**Chart 7:**
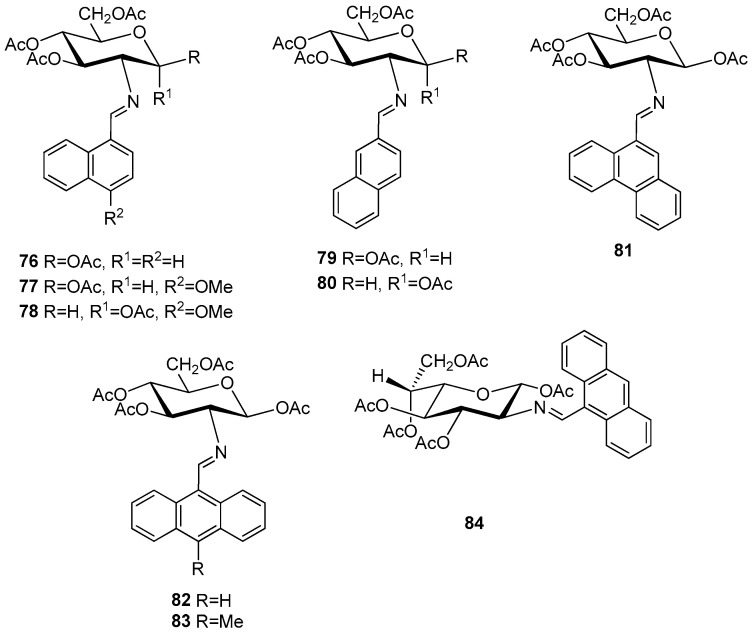
Per-*O*-acetyl imines from polynuclear aryl aldehydes.

The pyranose structure of **30**–**42** and **50**–**58** could further be confirmed by transforming some unprotected compounds into the corresponding per-*O*-acetyl derivatives **63**–**84** (Chart 6 and Chart 7), which were obtained in good yields by treatment with acetic anhydride in pyridine at ambient temperature [11,26].

**Scheme 4:**
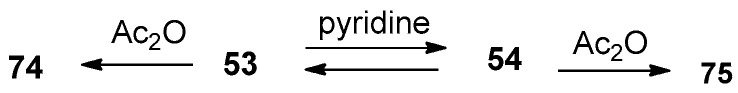
Partial anomerization during acetylation of imines.

Acetylation of **54** led to an approximately equimolar mixture of both β-(**79**) and α-anomers (**80**), thereby evidencing that during the acetylation process, **54** had enough time to partially transform into its β-anomer (**53**) (Scheme 4). Acetylation of **52** also led to an anomeric mixture dominated by the α-anomer **78**, which was obtained in pure form by fractional crystallization. The corresponding β-anomer (**77**) could easily be obtained from **51**.

In order to minimize the anomerization reaction, we attempted the acetylation of compounds **30** and **31** at a lower temperature (<−10 °C); however, in both cases, the β-anomer was obtained (**63** and **64**, respectively).

**Chart 8:**
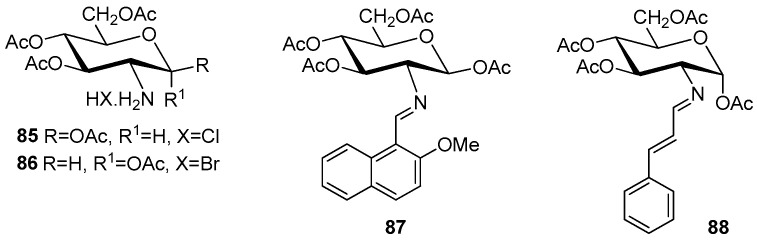
Structures of compounds **85**–**88**.

Alternatively, both α- and β-anomers of the per-*O*-acetyl imines derived from **2** could be obtained as pure anomers starting from the corresponding hydrohalides **85** [26] and **86** [27,28,29]. Accordingly, we were able to prepare **87**, whose unprotected imine could not be isolated, or the α-anomers **80** and **88** (Chart 8). The homologous imines from **6** were likewise synthesized from the corresponding hydrohalides **89** [17] and **91** [17].

The structures assigned to the new acetylated products **63**–**84**, **87** and **88** are in agreement with their elemental analyses, together with other physical and spectroscopic data (Tables S13–S24), which in turn confirm those of the parent imines. Again, derivatives **78** and **80** show low coupling constants *J*_1,2_ (<4 Hz), consistent with an axial arrangement of the anomeric acetate (α), whereas the rest of the per-*O*-acetyl imines exhibit large *J*_1,2_ (~8 Hz) constants, indicative of an equatorial arrangement (β) for the aforementioned acetate. In the acetylated derivatives, the values of ^1^*J*_C1-H1_ are ~177 Hz and ~166 Hz for α- and β-anomers, respectively, maintaining a diagnostic difference of ~10 Hz [21,22,23,24,25].

**Chart 9:**
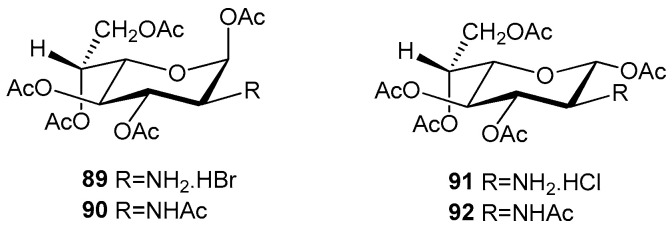
Structures of compounds **89**–**92**.

The carbon atom carrying the imine group (C-2) of **30**–**42**, **45**–**58**, **63**–**84**, **87** and **88** appears at an unusual downfield (~72–79 ppm), which deviates from other 2-amino-2-deoxyaldose derivatives [21,22]. For example, d-glucosamine itself in D_2_O shows values of 55 ppm and 58 ppm for the C-2 of the α- and β-anomers, respectively [30]. Likewise, the C-2 signals of **6** and 1,3,4,6,7-penta-*O*-acetyl-2-acetamido-2-deoxy-α-d-*glycero*-l-*gluco*-heptopyranose (**90**) appear at 51 ppm and 53 ppm for the β-anomer (**92**) [16] (Tables S25–S27). Such compounds were obtained through an unequivocal synthesis from **89** [17] and **91** [17] (Chart 9).

### 2.3. Mutarotation of Imines

**Scheme 5:**
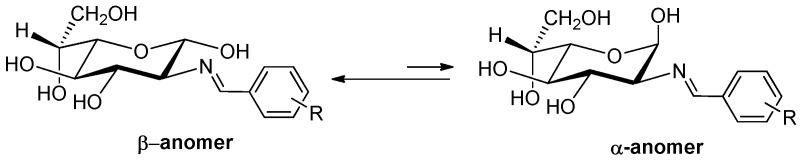
Anomeric equilibrium in imines derived from **6**.

**Chart 10:**
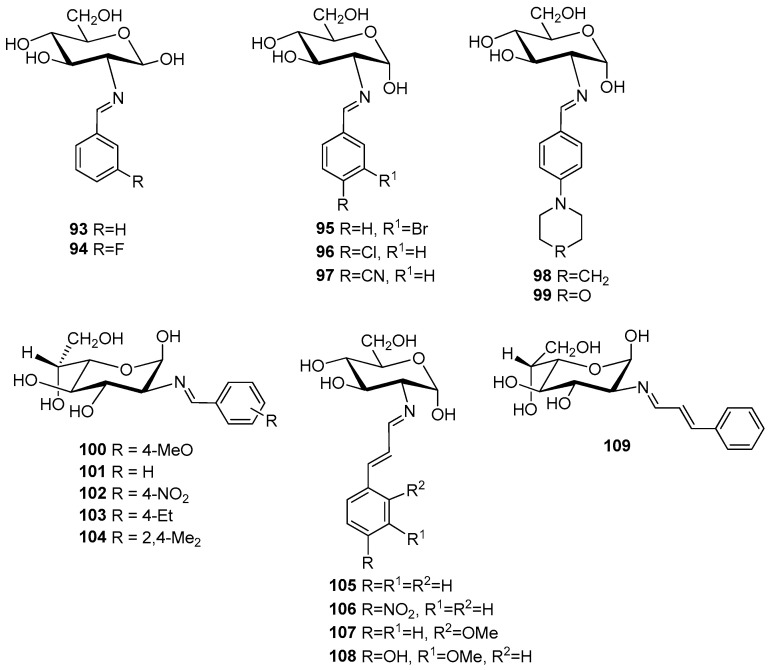
Anomers of imines **30***–***41**.

We performed a study on the mutarotation of imines in DMSO, whose origin could be ascribed not only to the existence of an anomeric equilibrium, but also to other phenomena such as tautomeric equilibria, sugar ring-size variation, conformational equilibria, typical carbohydrate rearrangements, reactions with solvent molecules, etc. The mutarotational behavior of imines **32**–**36** in solution is identical to that described for other imines of **2** derived from benzaldehydes [13], and imines **37**–**41** derived from **6** behave in the same way (Scheme 5). These imines only equilibrate with their respective α-anomers (**93**–**104**), while α-imines **30** and **31** do so with their β-anomers **93** and **94**, with the anomeric ratio remaining unaffected for some months (Chart 10).

Moreover, imines **45**–**49** in DMSO-*d*_6_ solution only equilibrate with their corresponding α-anomers (**105**–**109**). The spectroscopic data confirm the structure and anomeric configuration of such minor products. Thus, for example, the α-anomer **108** presents coupling constants ^3^*J*_H1,H2_^α^ = 3.3 Hz, ^1^*J*_C1,H1_^α^ = 165.5 Hz, and the β-anomer (**48**), ^3^*J*_H1,H2_^β^ = 8.4 Hz, ^1^*J*_C1,H1_^β^ = 153.7 Hz. Equilibration experiments conducted in pyridine-*d*_5_ are practically identical, albeit in this solvent, the equilibrium is reached very quickly, as shown by the following data recorded for the temporal variation in the β-anomer of **38**: just dissolved (85.9%); 1 h (85.9%); 8 h (86.0%); 1 d (85.3%); 3 d (84.5%); 5 d (87.9%), and finally 86.7%, i.e., without apparent variation over time.

**Chart 11:**
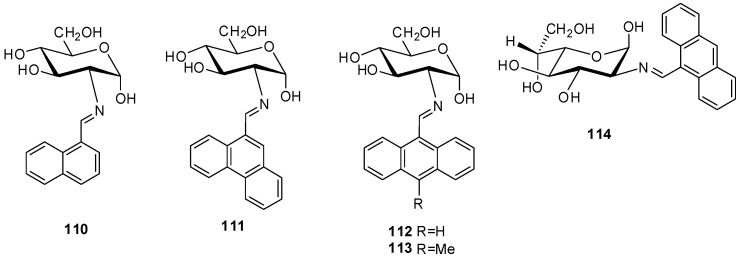
Axial anomers of imines **45***–***58**.

**Figure 1:**
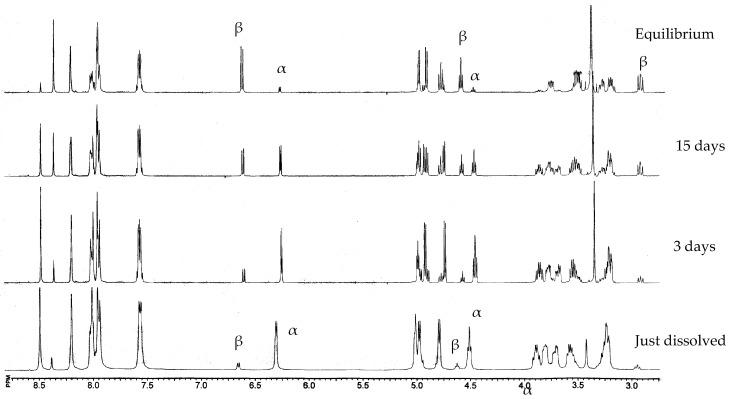
Evolution of **54** in DMSO-*d*_6_ solution.

The mutarotational behavior of imines derived from **2** and **6** with aldehydes bearing fused aromatic rings is similar as well (**110**–**114**) (Chart 11). Most of them appear as the β-anomer (**50**, **51**, **53**, **55**–**58**), although we were able to isolate two α-anomers (**52** and **54**). These compounds in solution slowly equilibrate with their β-anomers (**88** and **89**), which represent the dominant species (Figure 1). When the latter are allowed to evolve in solution, the final percentages of each anomer at equilibrium are approximately the same.

Table 1 shows the percentage variation for the β-anomer of some imines using DMSO-*d*_6_ and pyridine-*d*_5_ as solvents. In both cases, the results obtained are similar, although in pyridine the equilibration occurs faster. Probably the basic nature of this solvent is behind the rapid anomerization, since it is known that this phenomenon is sensitive to general acidic and basic catalysis [31]. This rapid anomerization in pyridine explains the failure to prepare per-*O*-acetylated α-anomers from α-imines, such as **30** and **31**, or the formation of mixtures of both anomers (as happens with **52** and **54**). The absence of typical oxazolidine signals at ~5–6 ppm and ~90–97 ppm [32,33,34,35,36,37], rules out the possibility of an equilibrium involving such five-membered heterocycles, which result from addition of the anomeric hydroxyl to the imine bond.

### 2.4. Conformational Analysis

**Figure 2:**
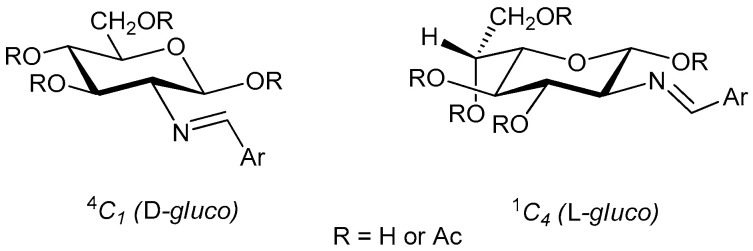
Preferred conformations in solution for imines derived from **2** and **6**.

The high coupling constants *J*_2,3_ ≈ *J*_3,4_ ≈ *J*_4,5_ ≥ 9 Hz fully agree with a d-*gluco* configuration in ^4^*C*_1_ conformation for all the imines derived from **2** and ^1^*C*_4_ (l-*gluco*) for those based on heptose **6** (Figure 2) [38,39].

**Figure 3:**
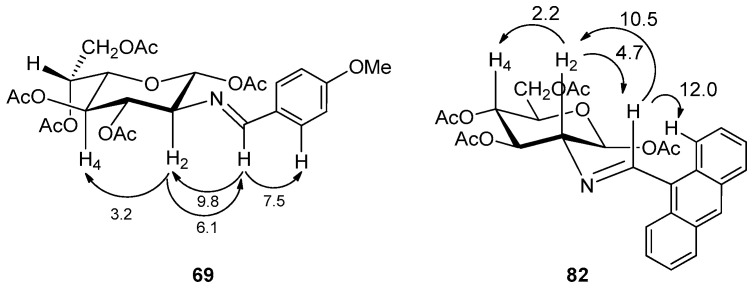
NOE effects measured for imines **69** and **82**.

NOE experiments [40,41] carried out on compound **69** gave rise to the enhancements shown in Figure 3, which confirmed the proximity of H-2, the iminic hydrogen, and one of the *ortho* hydrogens at the aromatic ring, all consistent with a ^1^*C*_4_ (l-*gluco*) conformation and coincidental with that determined through NMR spectroscopic data. Similar NOE enhancements were observed for **82** having a ^4^*C*_1_ (d-*gluco*) conformation. The absence of a NOE effect between the H-1 and H-2 protons is in agreement with their β-configuration.

**Figure 4:**
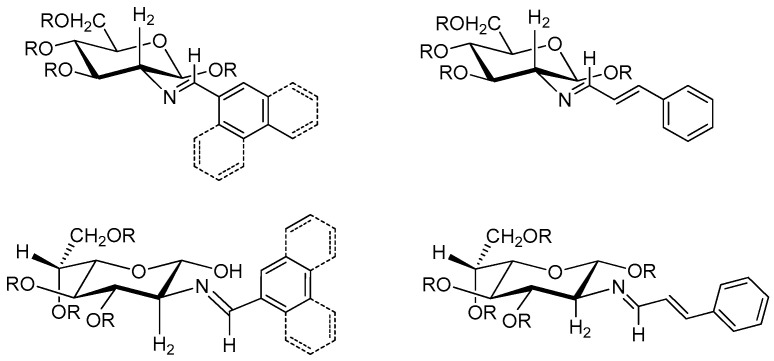
Conformational arrangements of benzylidene, naphthalene, phenantrene, and cinnamylidene derivatives (R=H or Ac).

Such NOE effects, together with those determined in other imines from **2** [13], strongly support some key structural features, namely the planarity of the arylimino group, its (*E*)-configuration, and the fact that the half-plane containing the entire conjugated unsaturated system is approximately perpendicular to the plane of the pyranose ring (Figure 4).

Again, the large coupling constants between the ethylene protons (*J*_CH=CH_~16 Hz) measured for **45**–**49** and their acetyl derivatives (**73**–**75**) indicate that the stereochemistry of the double bond is *trans* (*E*). Furthermore, the coupling constant between the imine proton and the neighboring ethylene proton (*J*_CH-CH=N_~8.8 Hz) evidences that both protons maintain an antiperiplanar relationship (Figure 4). It is interesting to note that the H-2 signal in imines is usually the most deshielded resonance, appearing at ~2.9 ppm, except in the case of anthracenes **56**–**58** and their acetylated derivatives **82**–**84**, which are shifted downfield (Δδ_H-2_~0.3 ppm); and the same happens to the iminic hydrogen of these compounds (Δδ_CH=N_~0.8–1 ppm). Such variations are not shown by phenanthrene derivatives **55** and **80**, which behave similarly to naphthalenes **50**–**54** and **76**–**80**. The origin of the observed variations lies most likely in the spatial arrangement of the anthracene nucleus, whose proximity to the H-2 and CH=N protons would cause this deshielding.

**Figure 5:**
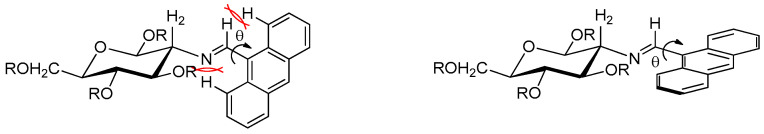
Conformational arrangements of anthracene derivatives, around the dihedral angle θ_N=C-C1-C2_.

Steric effects are noticeable in imines derived from anthracene, involving both the iminic hydrogen and the nitrogen atom. Steric tension can in part be relieved by rotating the aromatic system, although this reduces the delocalization through the imine double bond (Figure 5).

**Figure 6:**
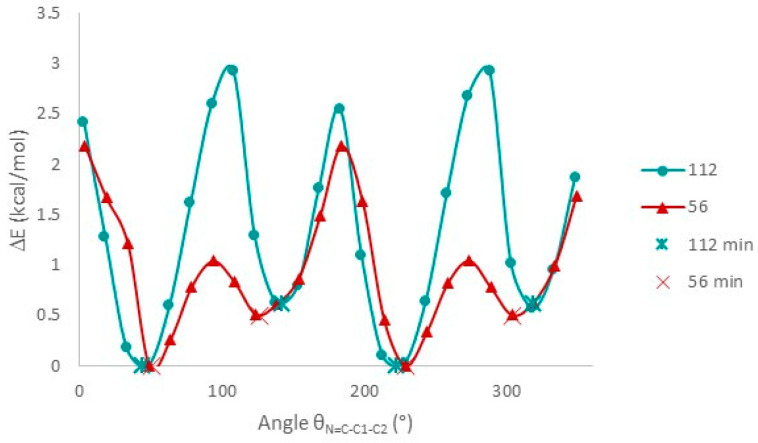
Conformational profiles of the aryl moiety of **56** and **112** at the M06-2X/6-311G(d,p) level.

Accordingly, a conformational analysis of the aryl moiety of **56** and **112** has been achieved, and the energy landscape of the arrangements generated around the NC-C_arom_ bond calculated, i.e., by rotating the dihedral angle θ_N=C-C1-C2_ from 0° to 360° with a step size of 15° each. The DFT study was performed using the 6-311G(d,p) [42,43] and def2-TZVP valence-triple-ζ [44] basis sets, with all geometries optimized in the gas phase at the B3LYP [45,46] and M06-2X [47] levels of theory without any geometrical restriction. The M06-2X/def2-TZVP combination has been reported to provide suitable geometry optimization in terms of cost and accuracy for carbohydrate derivatives [48,49,50]. Solvent effects were simulated using the SMD method [51]. Such results are shown in Figure 6 and Table 2.

**Figure 7:**
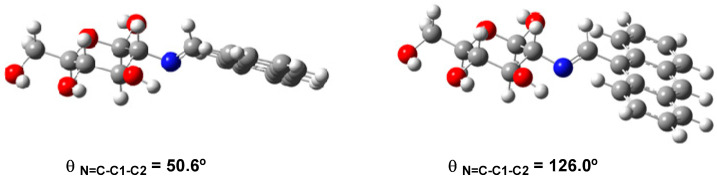
Optimized structures for the two conformational minima of **56** at the M06-2X/6-311G(d,p) level.

The graph is repeated every 180°, and the two minima obtained are almost identical for both anomers, the most stable conformers corresponding to dihedral angle values θ_N=C-C1-C2_ from ~40° to 50° (Figure 7). In other words, the minima represent a compromise to reach electron delocalization while reducing steric hindrance. Calculations using the 6-311G(d,p) basis set afford similar results for both anomers. However at the def2-TZVP level, the angle rotated by the β-anomer to reach the first minimum is similar, but in the opposite direction (−40° to −43°). The calculated conformation corresponding to the most stable point for other polynuclear imines shows dihedral angle values θ_H2-C2-N=CH_ from ~0° to 7° (*vide infra*); the identical conformation is inferred from NOE effects.

### 2.5. Theoretical Analysis of Imine Stability

**Figure 8:**
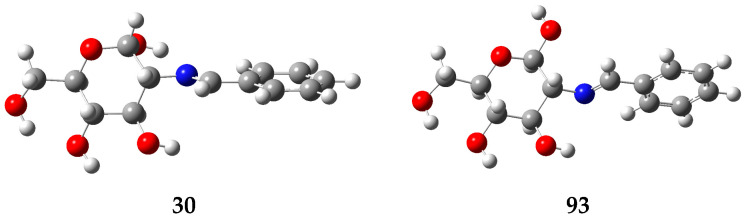
Stable arrangements of hydroxyl groups in structures **30** and **93** at the M06-2X/6-311G(d,p) level.

A computational study to determine the relative stability of the different species involved in mutarotational equilibria appears to be a compulsory task. The simplest imine pair derived from benzaldehyde (**30**/**93**) was selected to shorten the computational cost. The number of possible conformations is exceedingly high: the three staggered conformations of three hydroxyls and the iminic substituent of the pyranose ring, together with the nine (3 × 3) conformations adopted by the hydroxymethyl group at C-5, which amount to 3^6^ = 729 conformations for each anomer. Some simplifications can be envisaged for the hydroxyl groups, taking into account that the most stable conformations will be those leading to intramolecular hydrogen bonding. We then considered several dispositions, and the most stable conformers correspond to **30** and **93**, which differ only by the orientation of the anomeric OH group (Figure 8). This hydroxyl is oriented towards the electron pair of the nitrogen atom, enabling an intramolecular hydrogen bond in the α-anomer (**30**). In the β-anomer, that hydroxyl is arranged along the direction of the endocyclic oxygen (**93**). This methodology reduces drastically the number of structures to be calculated.

**Scheme 6:**
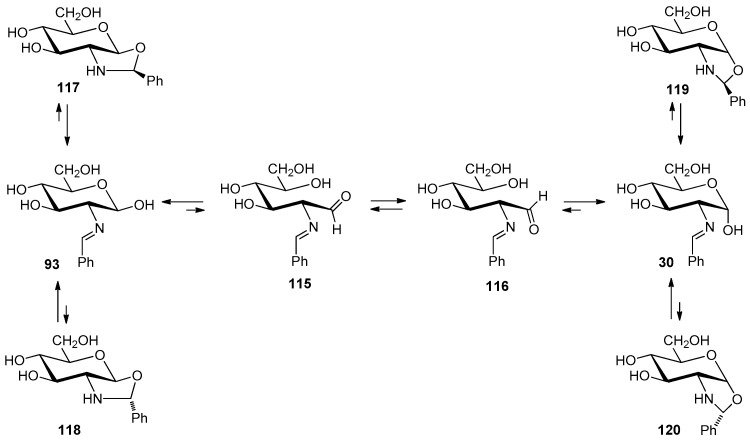
Acyclic and cyclic structures considered in mutarotational equilibria.

All the potential species involved in mutarotational equilibria (**30**, **93**, **115**, and **116**) [52], and the heterocycles that could have been formed by reaction of the imino group with the anomeric hydroxyl (**117**–**118**), have been taken into account and are depicted in Scheme 6.

**Chart 12:**
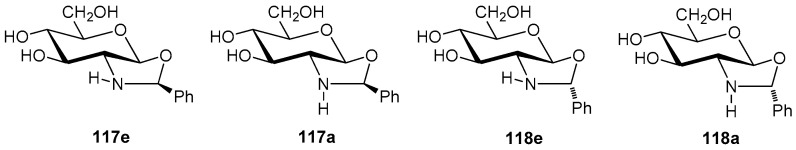
Axial and pseudo-equatorial orientations of the N-H bond in **117** and **118**.

**Figure 9:**
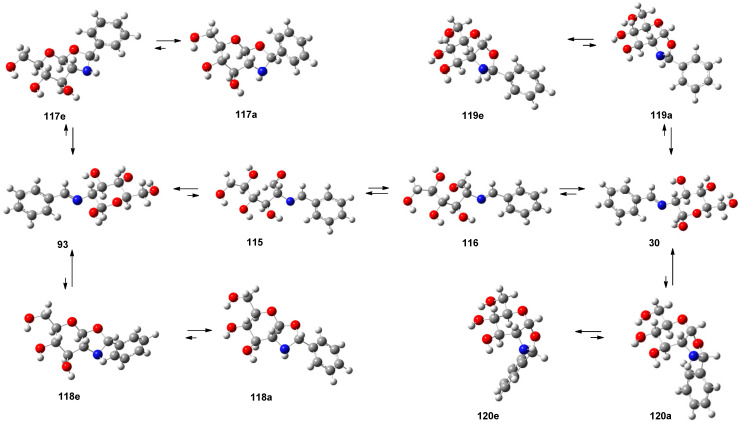
Optimized structures of **30**, **93** and **117**–**120** computed at the M06-2X/6-311G(d,p) level.

Moreover, for bicyclic structures like **117**–**120**, the two possible orientations of the hydrogen atom at the NH group, i.e., either axial (**a**) or pseudo-equatorial (**e**), have been considered as well (Chart 12 and Table 3). The tabulated data also collect the relative energies obtained by computation in the gas phase and using bulk solvation in DMSO, the solvent where NMR spectra are recorded (for optimized structures, see Figure 9).

Results obtained with the two hybrid functionals, B3LYP and M06-2X, are quite similar. Both in the gas phase and DMSO, the imine having an anomeric α-configuration is slightly more stable than its β-counterpart, although the difference is, indeed, so small that they can be ranked with identical stability. Both anomers (**30** and **93**) are interconverted through an acyclic aldehydic form. We estimated the energy of the two conformations adopted by the side chain along with the orientations of the aldehyde group that would lead to each anomer (**115** and **116**) [52]. The pronounced energy difference with respect to the corresponding pyranoid forms, in general ≥7 kcal/mol, explains why the acyclic forms hardly reach detectable concentrations in NMR experiments. It is well known, for example, that although the acyclic form of d-glucose is present in aqueous solutions, more than 99% actually exists as pyranose structures [38,39].

Regarding the possible cyclic structures, it is worth noting that those arising from the cyclization of the β-anomer, *trans*-oxazolidines **117** and **118**, are much less stable than the imine structure, ΔΔ*G*^DMSO^ = Δ*G*_oxaz.trans_ − Δ*G*_imine_ ≥ 8.6 kcal/mol (in DMSO), probably due to the strain associated with *trans*-fusion of a six-membered ring to a constrained pentagonal cycle. In stark contrast, for *cis*-oxazolidines **119** and **120,** the steric strain is lower, yet imines represent the most stable tautomers, ΔΔ*G*^DMSO^ = Δ*G*_oxaz.cis_ − Δ*G*_imine_ ≥ 0.9 kcal/mol (in DMSO).

Either axial or pseudo-equatorial arrangements of the NH cause little variations in the case of **117** and **118**, with the equatorial arrangement being more stable (ΔΔ*G* = Δ*G*_e_ − Δ*G*_a_ ≤ 1.9 kcal/mol in DMSO). However, for **119** and **120,** the axial disposition becomes significantly stabilized (ΔΔ*G* = Δ*G*_a_ − Δ*G*_e_ ≤ 3.2 kcal/mol in DMSO). The small energy difference with respect to **119e** (0.89 kcal/mol in DMSO) is surprising, suggesting it is possible that the species could be formed in the reaction mixture. However, as indicated above, the signals characteristic of the oxazolidine ring at ~5 ppm are not observed in the ^1^H NMR spectrum, thereby ruling out this speculation.

### 2.6. Anomeric Stabilization of 2-Aminoaldose Derivatives

A well-established principle in conformational analysis is that electronegative anomeric groups preferentially adopt an axial arrangement at the pyranose ring of sugars. This predisposition, contrary to expectations based on steric or solvation factors [53,54,55,56,57,58,59,60], is attributed to the existence of a stereoelectronic effect known as the anomeric effect. Its origin is associated with the hyperconjugation of the electron pairs on oxygen with the anomeric bond, also called the endo-anomeric effect. In turn, the anomeric substituent can generate a similar effect, involving the bonding orbital of the anomeric carbon and the oxygen of the pyranoid ring, which is known as exo-anomeric effect [54]. Both effects, together with neighboring *gauche* effects [61,62,63], are mainly responsible for the conformational arrangements of sugar derivatives and their reactivity [64]. In the absence of other factors, the exo-anomeric effect constitutes the most dominant interaction, even in the α-anomer.

The anomeric effect in carbohydrates is a complex, often puzzling, issue, although it can be interpreted by a combination of steric, resonance, hyperconjugation, inductive, hydrogen bonding, electrostatic, and solvation effects. The extent of such effects depends on the model and level of computation chosen [65]. It is believed that both steric and electronic interactions make contributions to the conformational preference, as any decomposition of such interactions is more or less arbitrary [66]. Some authors suggest that the steric interaction (or eventually a given electrostatic interaction) dominates the anomeric effect [67] and found further computational evidence to disprove the hyperconjugation explanation [68,69]. A cautionary corollary is that no single factor accounts for the axial preference of a substituent, while different and correlated interactions should be involved [70]. Moreover, the hyperconjugation model involving the electron transfer from the ring heteroatom to an excited state of an axial bond is a minor contributor to the anomeric effect. However, the effects exerted by substituents on the anomeric effect in positions other than the anomeric carbon have been scarcely studied. In any case, experimental data show that the most influential substituents are those located at the position adjacent to the anomeric center.

Anomeric stabilization in tetrahydropyranose sugars (*E*_an_), defined as the non-steric stabilization of the axial conformer, can be quantified by correcting the axial preference of a substituent, Δ*G*^o^_an_, with the steric effects favoring an equatorial arrangement, Δ*G*^o^_steric_ (Equation (1)):

*E*_an_ = Δ*G*^o^_an_ − Δ*G*^o^_steric_ = −RTlnK_an_ + A_X_ (1)

where Δ*G*^o^_an_ is the observed free energy change for the balance between the axial and equatorial disposition, i.e., α-anomer ⇄ β-anomer equilibrium (Equation (2)):

Δ*G*^o^_an_ = −RTln([β-anomer]/[α-anomer]) = −RTlnK_an_ (2)

Δ*G*^o^_steric_ can be estimated through non-anomeric model compounds, with the A_x_ values of cyclohexane usually employed to this end (Equation (3)):

Δ*G*^o^_steric_ = −RTln([equatorial]/[axial]) = −A_X_ (3)

Thus, the A_OH_ value for the hydroxyl group in aqueous solution is 1.25 kca/mol [=0.002 × 298 × ln(89/11)] and corresponds to an 89% predominance of cyclohexanol with the OH group placed in equatorial disposition [71,72]. When one varies the substituents at non-anomeric positions, a quantitative relationship for the anomeric hydroxyl group can be expressed by Equation (4):

*E*_an_ (kcal/mol) = −RTlnK_an_ + A_OH_ = −0.6 lnK_an_ + 1.25 (4)

A parameter capable of quantifying the magnitude of the RAE in imines (Δ*G*^o^_rae_) could be determined as the difference between the stabilization due exclusively to the anomeric effect (Δ*G*^o^_ae_) minus the anomeric stabilization in imines (Δ*G*^o^_imine_). If we take the anomeric effect as the value of E_an_ shown by **121** in DMSO-*d*_6_, 0.6 ln[(47.1)/(52.9)] + 1.25 = 1.32 kcal/mol, Equation (5) is obtained:

Δ*G*^o^_rae_ = Δ*G*^o^_ae_ − Δ*G*^o^_imine_ = *E*_an_**^121^** − *E*_an_^imine^ = 1.32 − *E*_an_^imine^ (5)

The values of Ax in tetrahydropyrans are greater than those obtained for cyclohexanes. Accordingly, the calculated anomeric effects (as *E*_an_) in Table 4, Table 5, Table 6 and Table 7 are approximate values. The steric interactions in the axial disposition of the substituent are more intense because the C-O bond in tetrahydropyran is shorter than in cyclohexane. Equation (6) extrapolates approximately the values of A_X_ (for cyclohexane ring) to the corresponding value in a tetrahydropyran ring (A_X_^THP^) [73]:

A_X_^THP^ (kcal/mol) = 1.53 A_X_^cyclohex^ + 0.02 (6)

The A_OH_^THP^ value for the hydroxyl group in tetrahydropyran is 1.93, and the corresponding values of E_an_ would increase by 0.68 (=1.93 − 1.25) kcal/mol (Equation (7)):

*E*_an_^THP^ = −RTlnK_an_ + A_OH_^THP^ = −0.6 lnK_an_ + 1.93 (7)

All the imines studied through this work show a broad preference for an equatorial arrangement of the anomeric hydroxyl, which clearly deviates from the expected anomeric effect. This behavior has its origin in the total or partial inhibition of the exo-anomeric effect in the α-anomer, which stems from the H-bonding between the imine nitrogen and the anomeric hydroxyl. In line with the above equations, all data from the studied equilibria are gathered in Table 4, Table 5, Table 6 and Table 7.

**Chart 13:**
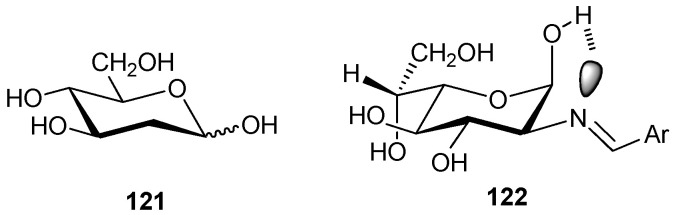
Structures of compounds **121** and **122**.

Special attention has been paid to imines derived from **2** with benzaldehydes bearing strong EWG (**8**, **9**, **12**, **13**). As already mentioned, since the lone pair on the nitrogen lies in the nodal plane of the arylimino system, the electronic effects exerted by the substituents at the aromatic nucleus can only be inductive. EWGs could decrease the basicity of the nitrogen atom, thus weakening the intramolecular H-bond with the anomeric hydroxyl and, as a result, the reverse anomeric effect. EDGs (**10**, **11**) should exhibit the opposite trend. However, no appreciable variations ascribed to the electronic effect of the substituents could be observed, and the extent of the RAE (Δ*G*^o^_rae_) remains above 1 kcal/mol. Data collected in Table 5 show that the behavior of imines derived from 2-amino-2-deoxy-d-*glycero*-l-*gluco*-heptopyranose with substituted benzaldehydes (**37**–**41**) is essentially identical to those derived from d-glucosamine. Therefore, a similar RAE can be invoked (**122**) (Chart 13) with comparable Δ*G*^o^_rae_ values. Data in Table 6 show that separation of the tetrahydropyran ring from the aromatic moiety by the ethylene bridge in imines **45**–**49** has no appreciable effect on the magnitude of the RAE, either.

Also, imines **50**–**58** with fused aromatic rings exhibit the same RAE as those previously mentioned and in similar extent (Table 7). One can conclude with confidence that this effect is neither significantly influenced by the volume of the aromatic substituent nor by its hydrophobicity.

### 2.7. Theoretical Analysis of Anomer Stability

**Figure 10:**
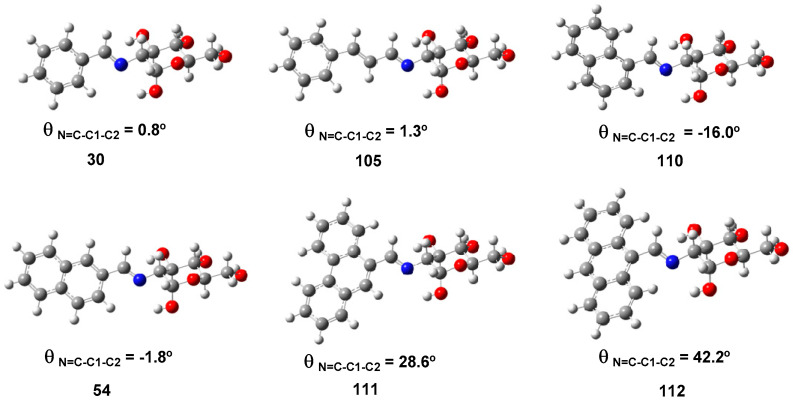
Optimized calculated structures of some representative α-anomers at the M06-2X/def2-TZVP.

**Figure 11:**
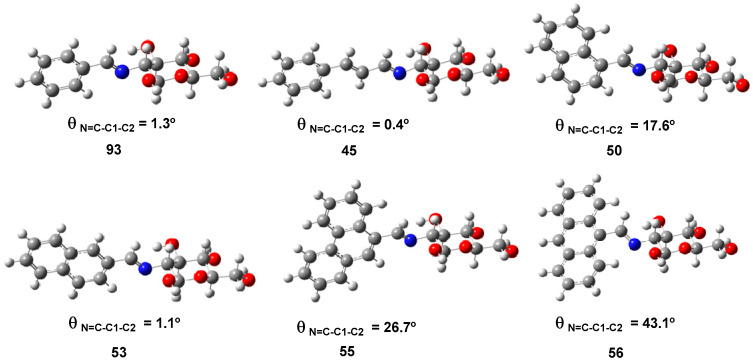
Optimized calculated structures of some representative β-anomers at the M06-2X/def2-TZVP.

Theoretical calculations have also been conducted to assess the relative stability of both anomers through the whole structural range of the imines synthesized here. Since aldehydes **23**, **26** and **27** do not have a plane of symmetry, their imines can adopt two different conformational dispositions for the imine group, which have been calculated as well. Results collected in Table 8 have been obtained with the M06-2X functional and using def2-TZVP as basis set. The def2-TZVP base indicates a greater stability of the β-anomer. The corresponding optimized geometries are shown in Figure 10 and Figure 11.

The energy difference (Δ*G*) calculated in DMSO (SMD method [51]), the solvent in which the anomeric equilibria have been evaluated, has allowed us to determine the expected proportion of the β-anomer at 298 K according to Equation (8) (Table 8, last column). The calculated equilibrium percentages of β-anomers vary from 63 to 85%, similarly to those determined experimentally.

[β] = {exp(−Δ*G*/RT)/[1 + exp(−Δ*G*/RT)]}100 (8)

In all α-anomers, the anomeric hydroxyl is oriented towards the electron pair of the nitrogen, generating an intramolecular hydrogen bond. The β-anomers cannot form it, and this hydroxyl points to the endocyclic oxygen.

**Figure 12:**
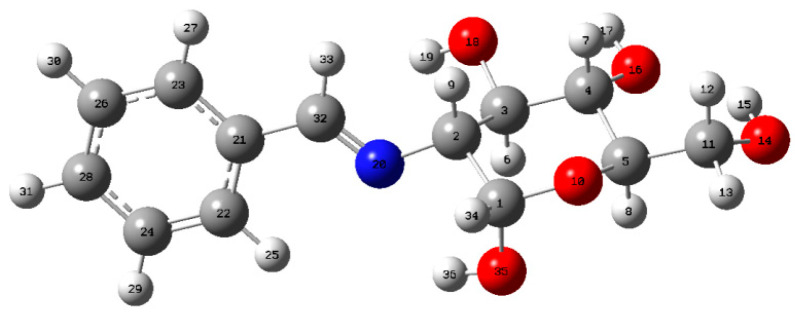
Numbering used in the NBO analysis of both anomers of some representative imines.

The results of a natural bonding orbital (NBO) [74] analysis, carried out for α- and β-anomers of some representative imines and involving the heteroatoms attached to the anomeric carbon and C-2, are shown in Tables S28 and S29 (numbering is shown in Figure 12).

All imines show similar stabilizing interactions for both anomers, regardless of the absence or presence of solvent. The stereoelectronic interactions of the lone pair on the nitrogen atom with the proton at C-2, n→σ*_C2-H_ (~6–7 kcal/mol) and the iminic CH, n→σ*_=CH_ (~12–13 kcal/mol), contribute to the perpendicular disposition of the imino group with respect to the pyranose plane, as deduced by NOE experiments.

The lone pairs on the endocyclic oxygen show delocalization with the antiparallel neighboring C-C and C-H bonds, with values for the n_O10_→σ*_C-C_ and n_O10_→σ*_C-H_ interactions of ~6–8 kcal/mol. In the α-anomer, the interaction responsible for the anomeric effect, n_O10_→σ*_C1-O35_, amounts to ~13.5 kcal/mol. The electron pairs on the anomeric hydroxyl oxygen show similar effects, highlighting an exo-anomeric effect in the β-anomer, n_O35_→σ*_C1-O10_, of ~15–17 kcal/mol. This effect is absent in the α-anomer due to hydrogen bonding between the anomeric hydroxyl and the imine nitrogen.

A more realistic calculation considers explicit solvent molecules, specifically water, around the imine molecule (Table 9). There are five water molecules that interact directly with the hydroxyl groups through intermolecular hydrogen bonds and form the first solvation shell, with the exception of α-anomers, whose anomeric hydroxyl is involved in the H-bond with the iminic nitrogen atom. The proportion of the β-anomer in DMSO, deduced from the relative stability between both anomers, is ~82%–99%, an estimation almost coincidental with the experimental values determined in that solvent. This percentage increases as the solvent’s dielectric constant increases, thus predicting a complete preponderance of the β-anomer in water.

### 2.8. Intramolecular Hydrogen Bond Strength

Based on calculated geometrical data, the strength of this intramolecular H-bonding could be approximately estimated by a well-known empirical relationship (Equation (9)) [75], where d_D…A_ represents the calculated distance between donor (O) and acceptor (N) atoms involved in H-bonding (Table 10).

*E*_HB_ (kcal/mol) = −5.554.10^5^exp(−4.12d_D…A_) (9)

Similar results are obtained at the M06-2X/6-311G(d,p) and def2-TZVP levels of theory, being approximately 6.5–7 kcal/mol, both in the gas phase and in the presence of solvent molecules (DMSO). Such energy values point to a moderate strength of the OH^…^N bond in α-anomers (Table 10, last column).

### 2.9. Inhibition of the Reverse Anomeric Effect

**Scheme 7:**
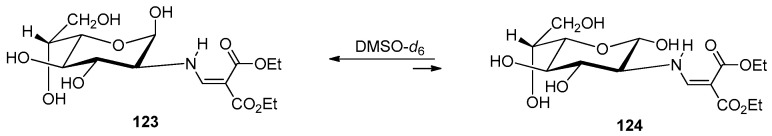
Anomer equilibration in heptose enamino derivatives **123** and **124**.

When an intramolecular hydrogen bond cannot be formed in the α-anomer, then the RAE is totally or partially eliminated. This occurs when the Schiff base adopts an enamine structure. Thus, for example, when enamine **123** [17] is allowed to remain in DMSO-*d*_6_ solution, equilibration with its β-anomer (**124**), which is the minor species (β-form: 15.3%), could be established after more than 2 months (Scheme 7). In this case, the intramolecular H-bonding is established with the carbonyl group of the enamine fragment, thereby inhibiting the bonding to the anomeric hydroxyl, and the exo-anomeric effect is totally or partially restored.

**Chart 14:**
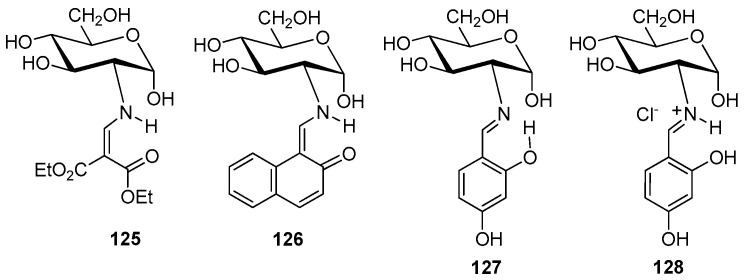
Compounds showing attenuated RAE or completely lacking the effect.

Other examples are illustrated by compounds **125** [76] and **126** [15,20] (Chart 14, Table 11). Schiff base **127** [77] crystallizes as the α-anomer, but in solution, it equilibrates with the β-anomer, which is the predominant species [13]. In this case, the RAE is only partially attenuated.

**Scheme 8:**
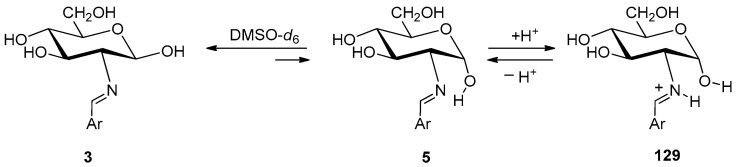
Elimination of RAE by protonation.

Protonation of the nitrogen atom represents the other way to sequester its lone pair. Thus, hydrochloride **128** [77] shows a complete reversal of the anomeric populations with respect to **127** and has an E_an_ coincidental with that of **123** and **125**. The free energy variation for protonated **128** (or deprotonated **127**) is 1.4 kcal/mol, with the exo-anomeric effect playing a dominant role. In addition, a strong intramolecular hydrogen bond is probably generated between the NH^+^ and the axial anomeric hydroxyl (α-anomer) (**129**), which restores the exo-anomeric effect (Scheme 8). The conformational rigidity of the imine group makes difficult the formation of this H-bonding in the β-anomer.

**Scheme 9:**
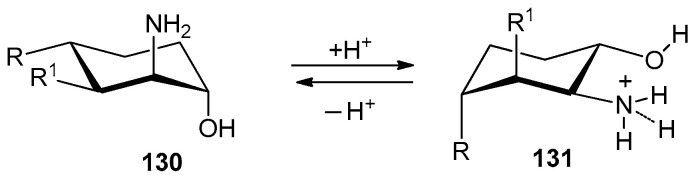
Ring inversion in aminocyclohexanols through protonation.

It is worth pointing out that this type of intramolecular bond formed by protonation has also been described for 2-aminocyclohexanol derivatives and is so powerful that it can invert the chair conformation of the cyclohexane ring (**130**), even though the substituents adopt axial dispositions (**131**) (Scheme 9) [78,79,80,81,82,83,84,85].

**Chart 15:**
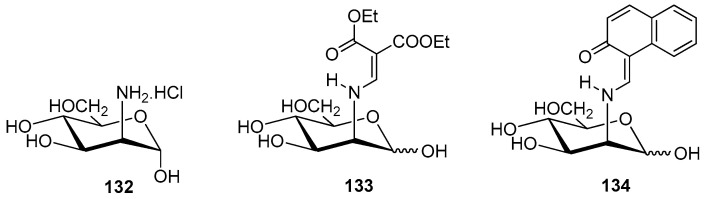
Known d-mannosamine Schiff bases.

In the case of d-mannosamine (**132**) in which the amino group is arranged axially in a ^4^*C*_1_ conformation of the pyranose ring, there is no possibility to remove the exo-anomeric effect. Accordingly, it should adopt a ^1^*C*_4_ chair conformation that would place all the substituents in axial dispositions, thus making an *impossible* structure. In fact, no Schiff bases of this aminosugar with an imine structure have been described, although two enamines have been reported (Chart 15), namely **133** [86] and **134** [87,88,89]. In such cases, the existence of the RAE can hardly be detected. As a result, the RAE does not affect 2-aminoaldoses for which the imino group adopts an axial arrangement in the more stable conformer.

**Scheme 10:**
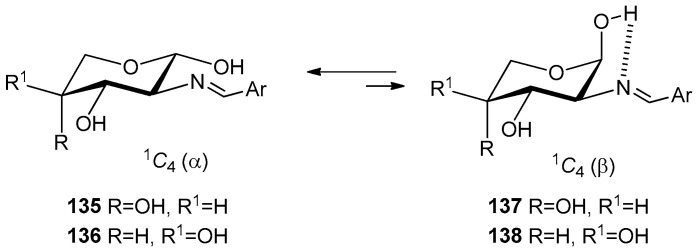
RAE in unprotected pentoses at equilibrium.

Furthermore, the RAE could modify the conformational behavior of imines derived from some 2-aminoaldoses. This is portrayed by imines of 2-amino-2-deoxy-pentopyranoses with d-*arabino* or l-*xylo* configurations. The most abundant species in the tautomeric equilibrium should be the α-anomer in ^1^*C*_4_ conformation (**135**/**136**), since the RAE would be feasible in the β-anomer (**137**/**138**) (Scheme 10).

In addition, the RAE may be present in other aminosugar derivatives and even in unprotected aminoaldoses themselves (Table 12). Thus, by replacing the hydroxyl at C-2 of d-glucose (**139**) by an amino group in d-glucosamine (**140**), the anomeric balance is shifted towards the equatorial anomer (β). However, protonation in the corresponding hydrochloride (**2**) reverses the anomeric ratio, making the α-anomer the more abundant species. The same applies to other protonated 2-aminosugars, such as **6** [16] and **142** [90]. This behavior is completely parallel to that shown by **127** and **128**, suggesting a similar performance of the RAE. Likewise, this trend occurs when replacing the amino group by the *N*-acetyl group, where the lone pair on the nitrogen atom participates in strong delocalization with the amide carbonyl. Thus, the anomeric ratio is reversed in **141** [91,92] with respect to **140** and becomes equal to that observed in compound **2**.

**Chart 16:**
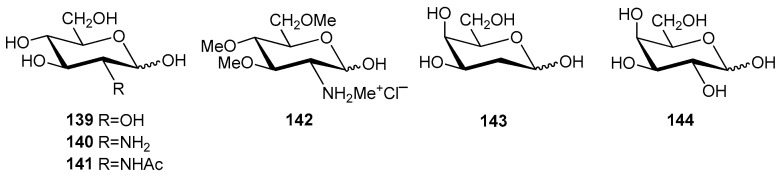
Hexose and 2-aminohexose derivatives exhibiting potential RAE.

As conclusive statement, one may say that whenever there is a heteroatom with at least one lone pair at C-2, a hydrogen bond could form with the hydroxyl of the α-anomer and inhibit the exo-anomeric effect. Such an inhibition should weaken the hydrogen bonding. Apparently, this happens when the presence of the hydroxyl at C-2 decreases the amount of the α-anomer, as illustrated by comparing 2-deoxy-d-glucose (**111**) and 2-deoxy-d-galactose (**143**) with d-glucose (**139**) and d-galactose (**144**), respectively (Chart 16). Overall and despite the intriguing complexity of all factors influencing the anomeric equilibrium, the RAE should invariably be taken into account in further studies.

## 3. Conclusions

New imines have been synthesized by condensation of d-glucosamine and 2-amino-2-deoxy-d-*glycero*-l-*gluco*-heptopyranose with mono- and polycyclic aromatic aldehydes, as well as cinnamylidene aldehydes. They all crystallize as equatorial anomers (β); although in the cases of benzaldehyde, 2-fluorobenzaldehyde, 4-methoxynaphthaldehyde and 2-naphthaldehyde, the corresponding axial anomers (α) could also be isolated. Such imines have been thoroughly characterized by preparing their per-*O*-acetyl derivatives. In solution, the Schiff bases derived from 2-amino-2-deoxyaldoses and alkyl and arylaldehydes establish an equilibrium between both anomers. The prevalence of the β-anomer is independent of the nature of the alkyl or aryl moiety carried by the imine nitrogen. When imines crystallize rapidly, the α-anomer is usually formed; otherwise, the β-anomer or mixtures of both anomers are obtained. This RAE can judiciously be ascribed to the total or partial inhibition of the exo-anomeric effect in the α-anomer, and results from of an intramolecular hydrogen bond between the anomeric hydroxyl and the nitrogen atom. As a key structural prerequisite, the iminic group in the pyranose ring should adopt an equatorial arrangement. The effect is reduced and disappears completely when Schiff bases either adopt enamine structures or undergo protonation of the imino group. Also, the protonation of the starting 2-amino-2-deoxyaldopyranoses modifies the anomeric balance, thus suggesting the action of RAE in such compounds. Theoretical calculations show that the formation of hydrogen bonding between the anomeric hydroxyl and the imine nitrogen is responsible for removal of the exo-anomeric effect on the α-anomer of 2-arylimino-2-deoxyaldopyranoses, which together with solvent effects (in terms of continuous solvation or discrete solvation) provide sufficient evidence supporting the preferential formation of the β-anomer.

## 4. Experimental Section

### 4.1. General Methods

All reagents and solvents were obtained from commercial suppliers and used without further purification. Melting points were determined on Gallenkamp MPA (York, UK) and Electrothermal IA 9000 (York, UK) apparatuses and are uncorrected. Optical rotations were determined on a Perkin-Elmer 241 polarimeter (Waltham, MA, USA) at 22 ± 2 °C, with sodium (D line, λ = 589 nm) and mercury beams (λ = 578, 546, 463 nm). IR spectra were recorded in the range of 4000–600 cm^−1^ on an FT-IR Thermo spectrophotometer (Waltham, MA, USA). Solid samples were recorded on KBr (Merck (Darmstadt, Germany)) pellets. NMR spectra were measured on Bruker 400 and 500 AC/PC instruments (Karlsruhe, Germany) in DMSO-*d*_6_ or CDCl_3_. Structural elucidation was facilitated through (a) distortionless enhancement by polarization transfer (DEPT), (b) 2D correlation spectroscopy (COSY), (c) heteronuclear multiple-quantum correlation (HMQC), (d) heteronuclear multiple bond correlation (HMBC), (e) isotope exchange with deuterium oxide, and (f) nuclear Overhauser effect (NOE). All *J* values are given in hertz. Microanalyses were determined on a Leco^®^ CHNS-932 analyzer (St. Joseph, MI, USA). High-resolution mass spectra (HRMS) were obtained using electrospray ionization (ESI) techniques with a 6520 Accurate-Mass Q-TOF LC/MS system from Agilent Technologies (Santa Clara, CA, USA) at the Servicio de Apoyo a la Investigación (SAIUEX) in the University of Extremadura.

### 4.2. Computational Details

The computational DFT study was initially carried out using the B3LYP [45,46] and the M06-2X [47] hybrid density functionals in conjunction with 6-31G(d,p) and 6-311G(d,p) basis sets [42,43], as implemented in the Gaussian09 package [93]. The M06-2X method was chosen on the basis of previous studies showing its accuracy in determining conformational energies associated with non-covalent interactions. To assess the influence of the level of theory on anomer stability, the def2-TZVP valence-triple-ζ basis set [44] was also employed in combination with the M06-2X functional for geometry optimizations. As mentioned, the latter has proven to be reliable enough for estimating structure and binding in other carbohydrate derivatives [48,49,50]. In all cases, frequency analyses were carried out to confirm the existence of true stationary points on the potential energy surface. All thermal corrections were calculated at the standard values of 1 atm at 298.15 K. Solvent effects were modeled through density-based, self-consistent reaction field (SCRF) theory of bulk electrostatics, i.e., the solvation model based on density (SMD) [51], as implemented in the Gaussian09 suite of programs. This solvation method accounts for long-range electrostatic polarization (bulk solvent) together with short-range effects due to cavitation, dispersion, and solvent structural effects.

We assessed mutarotational equilibria and solvent effects in 2-iminoaldose derivatives using four approaches: (a) gas-phase, as the absence of solvent allows determining the intrinsic stability of each species; (b) continuum solvation: anomerization was studied in solution with a description of the solvent as a continuum dielectric medium, using specifically the SMD model [51]; (c) microsolvation: calculations were conducted in the gas phase, but one or several water molecules were added to the resulting structures of the stationary points in order to determine the stabilization induced by hydrogen bonding, and (d) microsolvation and continuum solvation, which represents the hybrid between methods (b) and (c). Here, the assembly of the imine with one or several water molecules was studied in a continuum and polarizable dielectric medium.

### 4.3. Natural Bond Orbital (NBO) and Steric Analysis

Natural bond orbital analysis was performed with NBO 6.0 [74]. Intramolecular interactions of the stabilization energies were obtained using second-order perturbation theory and listed in the SI. For each donor NBO(i) and acceptor NBO(j), the stabilization energy *E*_2_ associated with electron delocalization between donor and acceptor is estimated as

*E*_2_ = Δ*E_ij_* = −*q_i_* (*F_ij_*)^2^/(*ε_i_* − *ε_j_*)

where *q_i_* is the donor orbital occupancy, *ε_i_*, *ε_j_* are diagonal elements (orbital energies), and *F_ij_* is the off-diagonal NBO Fock matrix element. In the natural bond orbital (NBO) approach, a hydrogen bond is viewed as an interaction between an occupied non-bonded natural orbital n_A_ of the acceptor atom A and the unoccupied antibonding orbital of the DH bond σ_DH_*.

### 4.4. Synthetic Procedures

Compounds **6** [14], **33** [11], **37** [17], **45** [19], **46** [20], **48** [20], **69** [17], **85** [26], **86** [27], **89** [17], **91** [17], **123** [17], **125** [76], **126** [15], **127** [77], and **128** [77] have been synthesized as described.

#### 4.4.1. Synthesis of Schiff Bases

New and reported substances were obtained according to the following general procedures. Method 1: To a solution of **2** or **6** (23.2 mmol) in 1*M* NaOH (25 mL) was added the appropriate aromatic aldehyde (25.0 mmol), and the mixture was stirred at room temperature. A solid precipitated and was collected by filtration and washed successively with cold water, cold ethanol, and ethyl ether, and dried under vacuum on silica gel. Method 2: Sodium hydrogen carbonate (0.50 g, 6.0 mmol) was added to a solution of **2** or **6** (4.7 mmol) in water (6 mL). To the resulting mixture, a solution of the appropriate aromatic aldehyde (4.7 mmol) in methanol (saturated solution) was added dropwise. The mixture was stirred at room temperature until precipitation, and then left in the refrigerator (~5 °C) overnight. The solid was collected, washed with cold water, ethanol, and ethyl ether, and dried in vacuo.

*2-[(*E*)-Benzylidenamino]-2-deoxy-α-d-glucopyranose* (**30**). Method 1 (60%). M.p: 169–171 °C; [α]_D_ +118.4°; [α]_578_ +124.8°; [α]_546_ +142.8°; [α]_436_ +267.4° (*c* 0.5, pyridine); [Lit. [24] m.p. 156 °C (decomp.)]; IR (KBr) $\bar{\nu }$_max_/cm^−1^ 3448, 3250 (OH), 2939, 2868 (CH), 1643 (C=N), 1581 (arom), 1154, 1085, 1010 (C-O), 758, 698 (arom); ^1^H NMR (500 MHz, DMSO-*d*_6_) δ 8.33 (1H, s, N=CH), 7.77 (2H, m, arom), 7.45 (3H, m, arom), 6.22 (1H, d, *J*_1,OH_ 4.0 Hz, C1-OH), 4.95 (1H, t, *J*_1,OH_ = *J*_1,2_ 3.5 Hz, H-1), 4.92 (1H, d, *J*_3,OH_ 5.5 Hz, C3-OH), 4.71 (1H, d, *J*_4,OH_ = 5.5 Hz, C4-OH), 4.46 (1H, t, *J*_6,OH_ = *J*_6′,OH_ 5.5 Hz, C6-OH), 3.83 (1H, dt, *J*_3,OH_ 5.5 Hz, *J*_2,3_ = *J*_3,4_ 9.5 Hz, H-3), 3.76 (1H, ddd, *J*_4,5_ 9.5 Hz, *J*_5,6_ 2.0 Hz, *J*_5,6′_ 5.5 Hz, H-5), 3.68 (1H, ddd, *J*_6,OH_ 5.5 Hz, *J*_5,6′_ 2.0 *J*_6,6′_ 11.5 Hz, H-6), 3.54 (1H, dt, *J*_6′,OH_ = *J*_5,6′_ 5.5 Hz, *J*_6,6_ 11.5 Hz, H-6′), 3.20 (1H, dt, *J*_4,OH_ 5.5 Hz, *J*_3,4_ = *J*_4,5_ 9.5 Hz, H-4), 3.14 (1H, dd, *J*_1,2_ 3.5 Hz, *J*_2,3_ 9.5 Hz, H-2); ^13^C{^1^H} NMR (125 MHz, DMSO-*d*_6_) δ 162.2 (N=C), 136.3, 130.6, 128.6 (2 C-arom), 128.2 (2 C-arom), 93.0 (C-1), 75.1 (C-2), 72.5 (C-5), 70.9 (C-3), 70.8 (C-4), 61.4 (C-6). Anal. Calcd. for C_13_H_17_NO_5_: C, 58.42; H, 6.41; N, 5.24. Found: C, 58.27; H, 6.20; N, 5.11. HRMS [M+H]^+^ calculated for C_13_H_18_NO_5_: 268.1185. Found: 268.1170.

*2-Deoxy-2-[(*E*)-(3-fluorobenzylidene)amino]-α-d-glucopyranose* (**31**). Method 1 (60%). M.p. 183–185 °C; [α]_D_ +154.0°; [α]_578_ +161.0°; [α]_546_ +185.4°; [α]_436_ +350.8° (*c* 0.5, pyridine); IR (KBr) $\bar{\nu }$_max_/cm^−1^ 3449, 3243 (OH), 2940, 2867 (CH), 1644 (C=N) 1614, 1585, 1452, 1152, 1084, 1011 (C-O); ^1^H NMR (500 MHz, DMSO-*d*_6_) δ 8.34 (1H, s, N=CH), 7.59 (2H, m, arom), 7.50 (1H, m, arom), 7.29 (1H, dt, *J* 3.2 Hz, *J* 8.5 Hz, arom), 6.23 (1H, d, *J*_1,OH_ 4.5 Hz, C1-OH), 4.95 (1H, t, *J*_1,OH_ = *J*_1,2_ 3.5 Hz, H-1), 4.92 (1H, d, *J*_3,OH_ 5.5 Hz, C3-OH), 4.74 (1H, d, *J*_4,OH_ = 5.5 Hz, C4-OH), 4.45 (1H, t, *J*_6,OH_ = *J*_6′,OH_ 6.0 Hz, C6-OH), 3.80 (1H, dt, *J*_3,OH_ 5.5 Hz, *J*_2,3_ = *J*_3,4_ 9.5 Hz, H-3), 3.76 (1H, ddd, *J*_4,5_ 9.5 Hz, *J*_5,6_ 2.0 Hz, *J*_5,6′_ 5.5 Hz, H-5), 3.67 (1H, ddd, *J*_6,OH_ 5.5 Hz, *J*_5,6′_ = 2.0, *J*_6,6′_ 11.5 Hz, H-6), 3.54 (1H, dt, *J*_6′,OH_ = *J*_5,6′_ 5.5 Hz, *J*_6,6′_ 12.0 Hz, H-6′), 3.20 (1H, dt, *J*_4,OH_ 5.5 Hz, *J*_3,4_ = *J*_4,5_ 9.9 Hz, H-4), 3.16 (1H, dd, *J*_1,2_ 3.0 Hz, *J*_2,3_ 10.0 Hz, H-2); ^13^C{^1^H} NMR (125 MHz, DMSO-*d*_6_) δ 162.4 (^1^*J*_C,F_ = 242.5 Hz, C3 arom), 161.0 (^4^*J*_C,F_ = 2.5 Hz, N=C), 138.9 (^3^*J*_C,F_ = 7.5 Hz, C1 arom), 130.7 (^4^*J*_C,F_ = 7.5 Hz, C5 arom), 124.7 (^3^*J*_C,F_ = 1.25 Hz, C6 arom), 117.4 (^2^*J*_C,F_ = 22.5 Hz, C2 arom), 113.8 (^2^*J*_C,F_ = 22.5 Hz, C4 arom), 92.8 (C-1), 74.9 (C-2), 72.5(C-5), 70.8 (C-3), 70.7 (C-4), 61.6 (C-6). Anal. Calcd. for C_13_H_16_FNO_5_: C, 54.73; H, 5.65; N, 6.66. Found: C,54.92; H, 5.37; N, 5.58. HRMS [M+H]^+^ calculated for C_13_H_17_FNO_5_: 286.1091. Found: 286.1077.

*2-Deoxy-2-[(*E*)-(3-bromobenzylidene)amino]-β-d-glucopyranose* (**32**). Method 1 (86%). M.p. 167–170 °C; [α]_D_ +51.6°; [α]_578_ +51.6°; [α]_546_ +55.0°; [α]_436_ +113.8° (*c* 0.5, pyridine); IR (KBr) $\bar{\nu }$_max_/cm^−1^ 3484, 3304, 3191 (OH), 1650 (C=N) 1563, 1106, 1070, 1034 (C-O), 989, 880; ^1^H NMR (500 MHz, DMSO-*d*_6_) δ 8.19 (1H, s, CH=N), 7.95 (1H, s, arom), 7.73 (1H, d, *J* 8.0 Hz, H-arom), 7.65 (1H, dd, *J* 8.0 Hz, *J* 1.0 Hz, H-arom), 7.41 (1H, t, *J* 8.0 Hz, H-arom), 6.62 (1H, d, *J*_1,OH_ 7.0 Hz, OH-1), 5.00 (1H, d, *J*_3,OH_ 5.5 Hz, OH-3), 4.92 (1H, d, *J*_4,OH_ 5.5 Hz, OH-4), 4.73 (1H, t, *J*_OH,1_ = *J*_1,2_ 8.0 Hz, H-1), 4.59 (1H, t, *J_6_*_,OH_ 5.5 Hz, OH-6), 3.72 (1H, ddd, *J*_5,6_ 1.5 Hz, *J_6_*_,OH_ 5.5 Hz, *J_6_*_,6′_ 11.5 Hz, H-6), 3.49 (1H, dt, *J*_5,6′_ = *J_6_*_,OH_ 5.5 Hz, *J_6_*_,6′_ 11.5 Hz, H-6′), 3.45 (1H, m, H-3), 3.25 (1H, ddd, *J*_5,6_ 2.0 Hz, *J_5_*_,6′_ 6.0 Hz, *J*_6,6′_ 9.0 Hz, H-5), 3.16 (1H, dt, *J_4_*_,OH_ 5.5 Hz, *J_3_*_,4_ = *J*_4,5_ 9.0 Hz, H-4), 2.85 (1H, dd~t, *J*_1,2_ 8.0 Hz, *J*_2,3_ 9.0 Hz, H-2); ^13^C{^1^H} NMR (125 MHz, DMSO-*d*_6_) δ 160.7 (C=N), 138.5 (C-arom), 133.2 (C-arom), 130.9 (C-arom), 130.1 (C-arom), 127.4 (C-arom), 122.1 (C-arom), 95.5 (C-1), 78.1 (C-2), 77.0 (C-5), 74.4 (C-3), 70.3 (C-4), 61.3 (C-6). Anal. Calcd. for C_13_H_16_BrNO_5_: C, 45.10; H, 4.66; N, 4.05. Found: C, 44.88; H, 4.84; N, 4.21. HRMS [M+H]^+^ calculated for C_13_H_17_BrNO_5_: 346.0290. Found: 346.0285.

*2-Deoxy-2-[(*E*)-(4-chlorobenzylidene)amino]-β-d-glucopyranose* (**33**) [11]. Method 1 (76%). M.p. 178–180 °C; [α]_D_ +38.4°; [α]_578_ +39.2°; [α]_546_ +47.8°; [α]_436_ +113.6° (*c* 0.5, pyridine); IR (KBr) $\bar{\nu }$_max_/cm^−1^ 3494, 3304, 3200 (OH), 1646 (C=N) 1597, 1085, 1068, 1030 (C-O), 987, 830; ^1^H NMR (500 MHz, DMSO-*d*_6_) δ 8.21 (1H, s, CH=N), 7.78 (2H, d, *J* 8.5 Hz, arom), 7.50 (2H, d, *J* 8.5 Hz, arom), 6.59 (1H, d, *J*_1,OH_ 6.5 Hz, OH-1), 4.96 (1H, d, *J*_3,OH_ 5.5 Hz, OH-3), 4.89 (1H, d, *J*_4,OH_ 5.5 Hz, OH-4), 4.73 (1H, t, *J*_OH,1_ = *J*_1,2_ 8.0 Hz, H-1), 4.57 (1H, t, *J*_6,OH_ 6.0 Hz, OH-6), 3.74 (1H, ddd, *J*_5,6_ 2.0 Hz, *J*_6,OH_ 5.5 Hz, *J*_6,6′_ 12.0 Hz, H-6), 3.50 (1H, dt, *J*_6′,OH_ = *J*_5,6′_ 6.0 Hz, *J*_6,6′_ 12.0 Hz, H-6′), 3.45 (1H, dt, *J*_3,OH_ 5.5 Hz, *J*_2,3_ ≈ *J*_3,4_ 9.0 Hz, H-3), 3.26 (1H, ddd, *J*_5,6_ 2.0 Hz, *J*_5,6′_ 6.0 Hz, *J*_4,5_ 9.0 Hz, H-5), 3.18 (1H, dt, *J*_4,OH_ 5.5 Hz, *J*_3,4_ = *J*_4,5_ 9.0 Hz, H-4), 2.86 (1H, t, *J*_1,2_ ≈ *J*_2,3_ 8.0 Hz, H-2); ^13^C{^1^H} NMR (125 MHz, DMSO-*d*_6_) δ 160. 9 (C=N), 135.1 (C-arom), 135.1 (C arom), 129.7 (2 C-arom), 128.7 (2 C- arom), 95.5 (C-1), 78.2 (C-2), 76.9 (C-5), 74.4 (C-3), 70.3 (C-4), 61.3 (C-6). Anal. Calcd. for C_13_H_16_N_2_O_7_: C, 51.71; H, 5.35; N, 4.64. Found: C, 51.56; H, 5.53; N, 4.70. HRMS [M+H]^+^ calculated for C_13_H_17_ClNO_5_: 302.0795. Found: 302.0778.

*2-Deoxy-2-[(*E*)-(4-cyanobenzylidene)amino]-β-d-glucopyranose* (**34**). Method 1 (70%). M.p. 165–167 °C; [α]_D_ +49.8°; [α]_578_ +52.0°; [α]_546_ +61.0°; [α]_436_ +138.0° (*c* 0.5, pyridine); IR (KBr) $\bar{\nu }$_max_/cm^−1^ 3559, 3522, 3466, 3342, 3157 (OH), 2234 (CN), 1641 (C=N) 1369, 1096, 1080, 1048, 1018 (C-O), 835; ^1^H NMR (500 MHz, DMSO-*d*_6_) δ 8.30 (1H, s, CH=N), 7.92 (4H, m, arom), 6.65 (1H, d, *J*_1,OH_ 6.5 Hz, OH-1), 4.99 (1H, d, *J*_3,OH_ 5.5 Hz, OH-3), 4.94 (1H, d, *J*_4,OH_ 5.5 Hz, OH-4), 4.76 (1H, t, *J*_OH,1_ = *J*_1,2_ 7.0 Hz, H-1), 4.59 (1H, t, *J*_6,OH_ 6.0 Hz, OH-6), 3.74 (1H, ddd, *J*_5,6_ 1.5 Hz, *J*_6,OH_ 5.5 Hz, *J*_6,6′_ 11.5 Hz, H-6), 3.50 (1H, dt, *J*_6,OH_ = *J*_5,6_ 6.0 Hz, *J*_6,6′_ 11.5 Hz, H-6′), 3.48 (1H, dt, *J*_6,OH_ 5.5 Hz, *J*_3,4_ = *J*_4,5_ 9.0 Hz, H-3,), 3.27 (1H, ddd, *J*_4,5_ 9.5 Hz, *J*_5,6_ 1.5 Hz, *J*_5,6′_ 6.0 Hz, H-5), 3.18 (1H, dt, *J*_4,OH_ 5.5 Hz, *J*_3,4_ = *J*_4,5_ 9.5 Hz, H-4), 2.91 (1H, dd≈t, *J*_1,2_ ≈ 7.5 *J*_2,3_ 9.5 Hz, H-2); ^13^C{^1^H} NMR (125 MHz, DMSO-*d*_6_) δ 161.0 (C=N), 140.1 (C-arom), 132.7 (2 C-arom), 128.7 (2 C-arom), 118.7 (C≡N), 112.7 (C-arom), 95.4 (C-1), 78.4 (C-2), 77.0 (C-5), 74.3 (C-3), 70.2 (C-4), 61.3 (C-6). Anal. Calcd. for C_14_H_16_N_2_O_5_: C, 57.53; H, 5.52; N, 9.58. Found: C, 57.34; H, 5.28; N, 9.41. HRMS [M+H]^+^ calculated for C_14_H_17_N_2_O_5_: 293.1137. Found: 293.1137.

*2-Deoxy-2-[(*E*)-(4-pyperidinylbenzylidene)amino]-β-d-glucopyranose* (**35**). Method 1 (62%). M.p. 172–174 °C; [α]_D_ +27.8°; [α]_578_ +30.0°; [α]_546_ +35.4°; [α]_436_ +84.0° (*c* 0.5, pyridine); IR (KBr) $\bar{\nu }$_max_/cm^−1^ 3466, 3292, 3178 (OH), 2919, 2838 (CH), 1630 (C=N) 1609 (arom), 1522, 1254, 1191, 1129, 1109, 1089, 1028 (C-O), 807; ^1^H NMR (500 MHz, DMSO-*d*_6_) δ 8.03 (1H, s, CH=N), 7.55 (2H, d, *J* 8.5 Hz, arom), 6.93 (2H, d, *J* 8.5 Hz, arom), 6.50 (1H, d, *J*_1,OH_ 7.0 Hz, OH-1), 4.90 (1H, d, *J*_3,OH_ 5.0 Hz, OH-3), 4.77 (1H, d, *J*_4,OH_ 5.5 Hz, OH-4), 4.68 (1H, t, *J*_OH,1_ = *J*_1,2_ 7.5 Hz, H-1), 4.55 (1H, t, *J*_6,OH_ = *J*_6′,OH_ 6.0 Hz, OH-6), 3.73 (1H, dd, *J*_6,OH_ 5.5 Hz, *J*_6,6′_ 12.0 Hz, H-6), 3.49 (1H, dt, *J*_6′,OH_ = *J*_5,6′_ 6.0 Hz, *J*_6,6′_ 12.0 Hz, H-6′), 3.42 (1H, dt, *J*_3,OH_ 6.0 Hz, *J*_2,3_ ≈ *J*_3,4_ 9.0 Hz, H-3), 3.24 (5H, m, H-5, NCH_2_, piperidine), 3.16 (1H, dt, *J*_4,OH_ 5.0 Hz, *J*_3,4_ = *J*_4,5_ 9.5 Hz, H-4), 2.76 (1H, t, *J*_1,2_ = *J*_2,3_ 8.5 Hz, H-2); 1.57 (6H, s, CH_2_ pyperidine); ^13^C{^1^H} NMR (125 MHz, DMSO-*d*_6_) δ 161.5 (C=N), 152.7 (arom), 129.3 (2 C-arom), 125.9 (C-arom), 114.3 (2 C-arom), 95.8 (C-1), 78.3 (C-2), 76.8 (C-5), 74.8 (C-3), 70.4 (C-4), 61.3 (C-6), 48. 6 (2C, piperidine), 25.0 (2C, piperidine), 24.0 (piperidine). Anal. Calcd. for C_13_H_16_N_2_O_7_: C, 61.70; H, 7.42; N, 7.99. Found: C, 62.01; H, 7.28; N, 8.26. HRMS [M+H]^+^ calculated for C_18_H_27_N_2_O_5_: 351.1920. Found: 351.1918.

*2-Deoxy-2-[(*E*)-(4-morpholinylbenzylidene)amino]-β-d-glucopyranose* (**36**). Method 1 (64%). M.p. 173–175 °C; [α]_D_ +31.4°; [α]_578_ +33.2°; [α]_546_ +38.8°; [α]_436_ +88.8° (*c* 0.5, pyridine); IR (KBr) $\bar{\nu }$_max_/cm^−1^ 3420, 3342, 3080 (OH), 2965, 2910, 2874, 2833 (CH), 1642 (C=N) 1610, 1518, 1117, 1070, 1029, 1011 (C-O), 923, 816; ^1^H NMR (500 MHz, DMSO-*d*_6_) δ 8.06 (1H, s, CH=N), 7.59 (2H, d, *J* 8.5 Hz, arom), 6.96 (2H, d, *J* 8.5 Hz, arom), 6.51 (1H, d, *J*_1,OH_ 6.5 Hz, OH-1), 4.91 (1H, d, *J*_3,OH_ 5.5 Hz, OH-3), 4.78 (1H, d, *J*_4,OH_ 6.0 Hz, OH-4), 4.69 (1H, t, *J*_OH,1_ = *J*_1,2_ 8.5 Hz, H-1), 4.55 (1H, t, *J*_6,OH_ = *J*_6′,OH_ 6.0 Hz, OH-6), 3.73 (5H, m, H-6, OCH_2_, morpholine), 3.49 (1H, dt, *J*_6′,OH_ = *J*_5,6′_ 6.0 Hz, *J*_6,6′_ 12.0 Hz, H-6′), 3.42 (1H, dt, *J*_3,OH_ 5.5 Hz, *J*_2,3_ ≈ *J*_3,4_ 9.0 Hz, H-3), 3.24 (1H, ddd, *J*_5,6_ 2.0 Hz, *J*_5,6′_ 6.0 Hz, *J*_4,5_ 9.0 Hz, H-5), 3.19 (4H, m, NCH_2_, morpholine), 3.15 (1H, dt, *J*_4,OH_ 6.0 Hz, *J*_3,4_ = *J*_4,5_ 9.5 Hz, H-4), 2.77 (1H, t, *J*_1,2_ = *J*_2,3_ 8.5 Hz, H-2); ^13^C{^1^H} NMR (125 MHz, DMSO-*d*_6_) δ 161.5 (C=N), 152.5 (C-arom), 129.3 (2 C-arom), 127.0 (C-arom), 114.1 (2 C-arom), 95.8 (C-1), 78.3 (C-2), 76.9 (C-5), 74.8 (C-3), 70.5 (C-4), 66.0 (2C, morpholine), 61.3 (C-6), 47.6 (2C, morpholine). Anal. Calcd. for C_13_H_16_N_2_O_7_: C, 57.94; H, 6.86; N, 7.95. Found: C, 58.13; H, 6.65; N, 7.92. HRMS [M+H]^+^ calculated for C_17_H_25_N_2_O_6_: 353.1713. Found: 353.1711.

*2-Deoxy-2-[(*E*)-(4-methoxybenzylidene)amino]-β-d-*glycero*-l-*gluco-*heptopyranose* (**37**) [17]. Method 2 (83%). M.p. 189–190 °C (Lit. [17] 190–192 °C); ^1^H NMR (500 MHz, DMSO-*d*_6_) δ 8.11 (1H, s, CH=N), 7.68 (2H, d, *J* 8.0 Hz, H-arom), 6.98 (2H, d, *J* 8.0 Hz, H-arom), 6.46 (1H, d, *J*_1,OH_ 6.5 Hz, OH-1), 4.82 (1H, bs, OH-3), 4.78 (1H, bs, OH-4), 4.66 (1H, t, *J*_1,OH_ = *J*_1,2_ 7.0 Hz, H-1), 4.49 (1H, bs, OH-7), 4.27 (1H, d, *J*_6,OH_ 6.0 Hz, OH-6), 3.79 (4H, m, H-6, OCH_3_), 3.86–3.44 (4H, m, H-3, H-4, H-7, H-7′), 3.27 (1H, d, *J*_4,5_ 8.0 Hz H-5), 2.78 (1H, t, *J*_1,2_ ≈ *J*_2,3_ 7.0 Hz, H-2); ^13^C{^1^H} NMR (125 MHz, DMSO-*d*_6_) δ 161.2 (C=N), 161.1 (C-arom), 131.9 (C-arom), 129.7 (2 C-arom), 113.9 (2 C-arom), 96.0 (C-1), 78.3 (C-2), 74.9 (C-3), 74.5 (C-5), 69.2 (C-4), 68.7 (C-6), 62.5 (C-7), 55.3 (OMe).

*2-[(*E*)-Benzylidenamino]-2-deoxy-β-d-*glycero*-l-*gluco*-heptopyranose* (**38**). Method 2 (37%). M.p. 155–157 °C (dec.); [α]_D_^25^ −49.1°; [α]_578_^25^ −51.9°; [α]_546_^25^ −60.0°; [α]_436_^25^ −120.9° (*c* 0.5, pyridine); IR (KBr) $\bar{\nu }$_max_/cm^−1^ 3375, 3295 (OH), 1643 (C=N) 1090, 1063, 1014 (C-O); ^1^H NMR (500 MHz, DMSO-*d*_6_) δ 8.20 (1H, s, CH=N), 7.75 (2H, m, H-arom), 7.45 (3H, m, H-arom), 6.51 (1H, sa, C1-OH), 4.85 (1H, s, OH), 4.83 (1H, s, OH), 4.70 (1H, t, *J*_1,OH_ ≈ *J*_1,2_ 7.5 Hz, H-1), 4.51 (1H, sa, C7-OH), 4.29 (1H, d, *J*_6,OH_ 6.5 Hz, C6-OH), 3.80 (1H, m, H-6), 3.5 (4H, m, H-3, H-4, H-7, H-7′), 3.29 (1H, d, *J*_4,5_ ≈ *J*_5,6_ 8.0 Hz, H-5), 2.85 (1H, t, *J*_1,2_ ≈ *J*_2,3_ 9.0 Hz, H-2); ^13^C{^1^H} NMR (125 MHz, DMSO-*d*_6_) δ 162.4 (C=N), 136.6, 131.0, 129.0, 128.5 (C-arom), 96.4 (C-1), 78.8 (C-2), 75.2 (C-3), 74.9 (C-5), 69.6 (C-4), 69.1 (C-6), 63.0 (C-7). Anal. Calcd. for C_14_H_19_NO_6_: C, 56.57; H, 6.40; N, 4.71. Found: C, 56.73; H, 6.23; N, 4.77. HRMS [M+H]^+^ calculated for C_14_H_20_NO_6_: 298.1285. Found: 298.1298.

*2-Deoxy-2-[(*E*)-(4-nitrobenzylidene)amino]-β-d-*glycero*-l-*gluco*-heptopyranose* (**39**). Method 2 (73%). M.p. 140–142 °C (dec.); [α]_D_ −61.2°; [α]_578_ −62.3°; [α]_546_ −73.6°; [α]_436_ −170.2° (*c* 0.5, pyridine); IR (KBr) $\bar{\nu }$_max_/cm^−1^ 3527, 3500–3100 (OH), 1649 (C=N), 1602 (arom), 1520 (NO_2_), 1429 (arom), 1345 (NO_2_), 1124, 1061, 1011 (C-O), 989, 838, 693, 682; ^1^H NMR (500 MHz, DMSO-*d*_6_) δ 8.36 (1H, s, CH=N), 8.30 (2H, d, H-arom), 8.03 (2H, d, H-arom), 6.60 (1H, s, C1-OH), 4.92 (1H, d, *J* 3.5 Hz, OH), 4.89 (1H, d, *J* 4.0 Hz, OH), 4.75 (1H, t, *J*_1,OH_ ≈ *J*_1,2_ 7.5 Hz, H-1), 4.51 (1H, t, *J*_7,OH_ 6.5 Hz, C7-OH), 4.27 (1H, d, *J*_6,OH_ 6.5 Hz, C6-OH), 3.80 (1H, c, *J*_6,OH_ 6.5 Hz, H-6), 3.47 (4H, m, H-3, H-4, H-7, H-7′), 3.31 (1H, m, H-5), 2.93 (1H, t, *J*_1,2_ ≈ *J*_2,3_ 8.5 Hz, H-2); ^13^C{^1^H} NMR (125 MHz, DMSO-*d*_6_) δ 160.9 (C=N), 148.9, 142.2, 129.5, 124.3 (C-arom), 96.2 (C-1), 78.9 (C-2), 75.0 (C-3), 74.9 (C-5), 69.5 (C-4), 69.1 (C-6), 62.9 (C-7). Anal. Calcd. for C_14_H_18_N_2_O_8_: C, 49.12; H, 5.26; N, 8.19. Found: C, 48.94; H, 5.23; N, 8.05. HRMS [M+H]^+^ calculated for C_14_H_19_N_2_O_8_: 343.1136. Found: 343.1149.

*2-Deoxy-2-[(*E*)-(4-ethylbenzylidene)amino]-β-d-*glycero*-l-*gluco*-heptopyranose* (**40**). Method 2 (60%). M.p. 182–184 °C (dec.); [α]_D_ −44.0°; [α]_578_ −46.53°; [α]_546_ −54.5°; [α]_436_ −107.4° (*c* 0.5, pyridine); IR (KBr) $\bar{\nu }$_max_/cm^−1^ 3367, 3284 (OH), 1644 (C=N),1609 (arom), 1081, 1014 (C-O); ^1^H NMR (500 MHz, DMSO-*d*_6_) δ 8.16 (1H, s, CH=N), 7.66 (2H, d, H-arom), 7.28 (2H, d, H-arom), 6.48 (1H, d, *J*_1,OH_ 6.5 Hz, C1-OH), 4.82 (1H, d, *J* 3.5 Hz, OH), 4.78 (1H, d, *J* 4.5 Hz, OH), 4.68 (1H, t, *J*_1,OH_ ≈ *J*_1,2_ 7.0 Hz, H-1), 4.51 (1H, t, *J*_7,OH_ 5.0 Hz, C7-OH), 4.27 (1H, d, *J*_6,OH_ 6.5 Hz, C6-OH), 3.79 (1H, m, H-6), 3.45 (4H, m, H-3, H-4, H-7, H-7′), 3.28 (1H, d, *J*_4,5_ ≈ *J*_5,6_ 8.0 Hz, H-5), 2.81 (1H, t, *J*_1,2_ ≈ *J*_2,3_ 8.0 Hz, H-2), 2.64 (2H, c, *J* 7.0 Hz, CH_2_), 1.19 (3H, t, *J* 7.5 Hz, CH_3_); ^13^C{^1^H} NMR (125 MHz, DMSO-*d*_6_) δ 162.2 (C=N), 149.9, 134.4, 128.6, 128.4 (C-arom), 94.4 (C-1), 78.8 (C-2), 75.2 (C-3), 74.9 (C-5), 69.6 (C-4), 69.2 (C-6), 63.0 (C-7), 28.6 (CH_2_), 15.9 (CH_3_). Anal. Calcd. for C_16_H_23_NO_6_: C, 59.08; H, 7.08; N, 4.31. Found: C, 58.86; H, 6.97; N, 4.33. HRMS [M+H]^+^ calculated for C_16_H_24_NO_6_: 326.1598. Found: 326.1612.

*2-Deoxy-2-[(*E*)-(2,4-dimethylbenzylidene)amino]-β-d-*glycero*-l-*gluco*-heptopyranose* (**41**). Method 2 (40%). M.p. 175–177 °C (dec.); [α]_D_ −45.9°; [α]_578_ −46.6°; [α]_546_ −54.1°; [α]_436_ −111.9° (*c* 0.5, pyridine); IR (KBr) $\bar{\nu }$_max_/cm^−1^ 3367, 3294 (OH), 1634 (C=N),1613 (arom), 1084, 1010 (C-O); ^1^H NMR (500 MHz, DMSO-*d*_6_) δ 8.40 (1H, s, CH=N), 7.69 (2H, d, H-arom), 7.04 (2H, d, H-arom), 6.46 (1H, d, *J*_1,OH_ 7.0 Hz, C1-OH), 4.81 (1H, d, *J* 5.0 Hz, OH), 4.76 (1H, d, *J* 5.0 Hz, OH), 4.67 (1H, t, *J*_1,OH_ ≈ *J*_1,2_ 7.5 Hz, H-1), 4.49 (1H, t, *J*_7,OH_ 5.0 Hz, C7-OH), 4.26 (1H, d, *J*_6,OH_ 7.0 Hz, C6-OH), 3.79 (1H, c, *J*_6,OH_ 6.5 Hz, H-6), 3.43 (4H, m, H-3, H-4, H-7, H-7′), 3.28 (1H, d, *J*_4,5_ ≈ *J*_5,6_ 8.5 Hz, H-5), 2.83 (1H, t, *J*_1,2_ ≈ *J*_2,3_ 8.5 Hz, H-2), 2.43, 2.29 (9H, s, CH_3_); ^13^C{^1^H} NMR (125 MHz, DMSO-*d*_6_) δ 160.8 (C=N), 140.0, 137.7, 132.0, 131.8, 128.0, 127.0 (C-arom), 96.5 (C-1), 79.1 (C-2), 75.3 (C-3), 74.9 (C-5), 69.7 (C-4), 69.2 (C-6), 63.0 (C-7), 21.4, 19.4 (CH_3_). Anal. Calcd. for C_16_H_23_NO_6_: C, 59.08; H, 7.08; N, 4.31. Found: C, 59.15; H, 6.88; N, 4.26. HRMS [M+H]^+^ calculated for C_16_H_24_NO_6_: 326.1598. Found: 326.1609.

*2,5-*Bis*(d-*galacto*-pentitol-1-yl)pyrazine* (**43**). The title compound was obtained using Method 1 from 2-amino-2-deoxy-d-*glycero*-l-*gluco*-heptopyranose (**6**) and 2,4,6-trimethyl benzaldehyde. After partial solvent evaporation, a white solid was obtained, then isolated by filtration and washed with cold ethanol and ethyl ether. It decomposes without melting above 200 °C; [α]_D_ −75.4°; [α]_578_ −82.0°; [α]_546_ −77.8°; [α]_436_ −86.8° (*c* 0.4, pyridine); IR(KBr) $\bar{\nu }$_max_/cm^−1^ 3600–3100 (OH), 1637, 1491 (arom), 1043, 1028 (C-O), 870, 848 (arom.); ^1^H NMR (500 MHz, DMSO-*d*_6_) δ 8.63 (2H, s, H-arom), 5.28 (2H, s, C1-OH), 4.99 (2H, d, *J* 4.5 Hz, H-1), 4.45 (2H, s, C5-OH), 4.32 (2H, d, *J* 9.0 Hz, C4-OH), 4.30 (2H, d, *J* 8.5 Hz, C2-OH), 4.19 (2H, s, C3-OH), 3.74 (4H, s, H-3, H-4), 3.63 (2H, t, *J* 7.5 Hz, H-2); 3.44 (4H, t~s, H-5, H-5′); ^13^C{^1^H} NMR (125 MHz, DMSO-*d*_6_) δ 156.3 (C=N arom.), 141.3 (CH=, arom.), 72.7 and 69.9 (C-3, C-4), 71.3 (C-1), 69.4 (C-2), 63.1 (C-5). Anal. Calcd. for C_14_H_24_N_2_O_10_: C, 41.69; H, 5.96; N, 6.95. Found: C, 41.53; H, 5.86; N, 7.02. HRMS [M+H]^+^ calculated for C_14_H_25_N_2_O_10_: 381.1504; Found: 381.1518; [M+Na]^+^ calculated for C_14_H_24_N_2_O_10_Na: 403.1323. Found: 403.1332.

*2,5-*Bis*(1′,2′,3′,4′,5′-penta-*O*-acetyl-d-*galacto*-pentitol-1-yl)pyrazine* (**44**). To a suspension of 2,5-*bis*-(d-*galacto*-pentitol-1-yl)pyrazine (**49**) in pyridine (9.4 mL), acetic anhydride (9.0 mL) was added with stirring and external cooling. The mixture was allowed to warm at room temperature until dissolution. It was then poured into ice-water, and the resulting solid was filtered and washed with cold water and dried in vacuum over silica gel (62%). M.p. 217–219 °C; IR(KBr) $\bar{\nu }$_max_/cm^−1^ 1742 (C=O, acetate), 1373, 1222 and 1206 (C-O-C, acetate), 1029 (C-O); ^1^H NMR (500 MHz, CDCl_3_) δ 8.44 (2H, s, H-arom), 6.02 (2H, d, *J* 1.5 Hz, H-1), 5.65 (2H, dd, *J* 1.5 Hz, *J* 10.0 Hz, H-2), 5.55 (2H, dd, *J* 10.0 Hz, *J* 1.5 Hz, H-3), 5.30 (2H, m, H-4), 4.31 (2H, dd, *J* 5.0 Hz, *J* 11.5 Hz, H-5), 3.90 (2H, dd, *J* 7.5 Hz, *J* 11.5 Hz, H-5′), 2.23 (6H, s, acetate), 2.16 (6H, s, acetate), 2.05 (6H, s, acetate), 2.04 (6H, s, acetate), 1.65 (6H, s, acetate); ^13^C{^1^H} NMR (125 MHz, CDCl_3_) δ 170.4, 170.2, 169.8, 168.6 (C=O), 151.1, 141.3 (C-arom), 71.8 (C-1), 68.9 (C-2), 67.8 (C-3), 67.6 (C-4), 62.0 (C-5), 20.7, 20.6, 20.6, 19.9 (CH_3_). Anal. Calc. for C_34_H_44_N_2_O_20_: C, 51.00; H, 3.50; N, 6.95. Found: C, 49.83; H, 3.60; N, 6.87. HRMS [M+H]^+^ calculated for C_34_H_45_N_2_O_20_: 801.2560; found: 801.2593; [M+Na]^+^ calculated for C_34_H_44_N_2_O_20_Na: 823.2380; found: 823.2399; [M+K]^+^ calculated for C_34_H_44_N_2_O_20_K: 839.2119; found: 839.2129.

*2-[(*E,E*)-Cinnamylideneamino]-2-deoxy-β-d-glucopyranose* (**45**) [19]. Method 2 (40%); M.p. 173–175 °C; [α]_D_ +31.2°; [α]_578_ +31.8°; [α]_546_ +37.8°; [α]_436_ +114.0° (*c* 0.5, pyridine); [Lit. [47] M.p. 187 °C, [α]_546_ +57.6° (pyridine)]; IR (KBr) $\bar{\nu }$_max_/cm^−1^ 3000–2922 (OH), 1632 (C=N), 1630 (C=C), 1096, 1010 (C-O), 758, 694, 631 (arom); ^1^H NMR (400 MHz, DMSO-*d*_6_) δ 7.93 (1H, d, *J*_=C*H*-CH_ 8.8 Hz, N=C*H*-CH), 7.59 (2H, d, *J* 7.3 Hz H-arom), 7.36 (3H, m, H-arom), 7.11 (1H, d, *J*_CH=C*H*_ 16.1 Hz, CH=C*H*-Ar), 6.90 (1H, dd, *J*_C*H*=CH_ 16.1 Hz, *J*_C*H*-CH_ 8.8 Hz, CH-C*H*=CH), 6.56 (1H, d, *J*_C1,OH_ 6.7 Hz C1-OH), 4.95 (1H, d, *J*_C3,OH_ 4.7 Hz, C3-OH), 4.85 (1H, d, *J*_C4,OH_ 5.4 Hz, C4-OH), 4.64 (1H, d, *J*_1,2_ 7.7 Hz, H-1), 4.56 (1H, t, *J*_C6,OH_ 5.3 Hz, C6-OH), 3.72 (1H, dd, *J*_6,6′_ 11.4 Hz, *J*_5,6_ 3.1 Hz, H-6), 3.47 (1H, m, *J*_6,6′_ 11.9 Hz, *J*_5,6′_ 5.8 Hz, H-6′), 3.36 (1H, m, H-3), 3.21 (1H, m, *J*_5,6′_ 1.5 Hz, *J*_5,6_ 5.7 Hz, H-5), 3.13 (1H, dt, *J*_3,4_ ≈ *J*_4,5_ 9.0 Hz, *J*_C4-OH_ 4.2 Hz, H-4), 2.71 (1H, t, *J*_1,2_ ≈ *J*_2,3_ 8.5 Hz, H-2); ^13^C{^1^H} NMR (100 MHz, DMSO-*d*_6_) δ 163.9 (C=N), 141.4 (2C, CH=CH), 136.8, 129.1, 128.6, 127.4 (2 C-arom), 95.8 (C-1), 78.6 (C-2), 77.1 (C-5), 74.8 (C-3), 70.5 (C-4), 61.5 (C-6). Anal. Calcd. for C_15_H_19_NO_5_: C, 61.42, H, 6.53, N, 4.78. Found: C, 61.20, H, 6.59, N, 4.86.

*2-Deoxy-2-[(*E,E*)-(4-nitrocinnamylidene)amino]-β-d-glucopyranose* (**46**) [20]. Method 2 (49%). M.p. 196–198 °C; [α]_D_ +31.2°; [α]_578_ +31.8°; [α]_546_ +37.8°; [α]_436_ +114.0° (*c* 0.5, pyridine); [Lit. [47] M.p. 172–173 °C]; IR (KBr) $\bar{\nu }$_max_/cm^−1^ 3545 (OH), 1634 (C=N), 1510 (arom), 1072, 1013 (C-O), 829, 750 (arom); ^1^H NMR (400 MHz, DMSO-*d*_6_) δ 8.21 (2H, d, *J* 8.7 Hz, H-arom), 7.99 (1H, d, *J*_=CH-CH_ 8.6 Hz, N=CH-CH), 7.87 (2H, d, *J* 8.7 Hz, H-arom), 7.26 (1H, d, *J* 16.1 Hz, CH=CH-Ar), 7.11 (1H, dd, *J* 8.7 Hz, *J*_C**H**=CH_ 16.1 Hz, CH-CH=CH), 6.61 (1H, d, *J*_C1-OH_ 6.7 Hz, C1-OH), 4.98 (1H, d, *J*_C4-OH_ 5.2 Hz, C4-OH), 4.90 (1H, d, *J*_C3-OH_ 5.7 Hz, C3-OH), 4.68 (1H, t, *J*_1,2_ 7.2 Hz, H-1), 4.58 (1H, t, *J*_C6-OH_ 5.7 Hz, C6-OH), 3.72 (1H, dd, *J*_6,6′_ 10.2 Hz, *J*_5,6_ 5.4 Hz, H-6), 3.48 (1H, m, H-6′, H-3), 3.23 (1H, m, H-5), 3.15 (1H, m, H-4), 2.76 (1H, t, *J*_1,2_ ≈ *J*_2,3_ 8.5 Hz, H-2); ^13^C{^1^H} NMR (100 MHz, DMSO-*d*_6_) δ 163.5 (C=N), 147.4, 142.6 (C-arom), 138.9 (CH=CH), 132.7, 128.4 (C-arom), 124.2 (CH=CH), 95.7 (C-1), 78.7 (C-2), 77.1 (C-5), 74.7 (C-3), 70.4 (C-4), 61.4 (C-6). Anal. Calcd. for C_15_H_18_N_2_O_7_: C, 53.25, H, 5.36, N, 8.28. Found: C, 53.03, H, 5.55, N, 8.36.

*2-Deoxy-2-[(*E,E*)-(2-methoxycinnamylidene)amino]-β-d-glucopyranose* (**47**). Method 2 (94%); M.p. 184–185 °C; [α]_D_ −7.4°; [α]_578_ −8.4°; [α]_546_ −7.6°; [α]_436_ +2.4° (*c* 0.5, pyridine); IR (KBr) $\bar{\nu }$_max_/cm^−1^ 3500–2800 (OH), 1635 (C=N), 1598 (CH=CH), 1488, 1462 (arom), 1249, 1160 (C-O-C, ester), 1079, 1024, 985 (C-O), 898, 754 (arom); ^1^H NMR (400 MHz, DMSO-*d*_6_) δ 7.92 (1H, d, *J*_=C*H*-CH_ 8.9 Hz, N=C*H*-CH), 7.60 (1H, dd, *J* 7.7 Hz, *J* 1.2 Hz, H-arom), 7.33 (1H, dt, *J* 7.7 Hz, *J* 1.4 Hz, H-arom), 7.26 (1H, d, *J*_CH=C*H*_ 16.2 Hz, CH=C*H*-Ar), 6.97 (1H, t, *J* 7.5 Hz, H-arom), 6.90 (1H, dd, *J*_C*H*=CH_ 16.2 Hz, *J*_C*H*-CH_ 8.9 Hz, CH-C*H*=CH), 6.55 (1H, d, *J*_C1,OH_ 6.8 Hz, C1-OH), 4.96 (1H, d, *J*_C3-OH_ 5.2 Hz, C3-OH), 4.84 (1H, d, *J*_C4-OH_ 5.8 Hz, C4-OH), 4.63 (1H, d, *J*_1,2_ 7.3 Hz, H-1), 4.57 (1H, t, *J*_C6-OH_ 5.8 Hz, C6-OH), 3.85 (3H, s, OCH_3_), 3.71 (1H, ddd, *J*_6,6′_ 11.5 Hz, *J*_C6,OH_ 5.5 Hz, *J*_5,6_ 1.5 Hz, H-6), 3.47 (1H, dt, *J*_6,6′_ 11.6 Hz, *J*_C6,OH_ ≈ *J*_5,6′_ 5.8 Hz, H-6′), 3.36 (1H, m, H-3), 3.18 (1H, m, *J*_5,6_ 5.8 Hz, *J*_5,6′_ 1.6 Hz, H-5), 3.15 (1H, dt, *J*_3,4_ ≈ *J*_4,5_ 8.9 Hz, *J*_C4-OH_ 5.3 Hz, H-4), 2.70 (1H, t, *J*_1,2_ ≈ *J*_2,3_ 8.5 Hz, H-2); ^13^C{^1^H} NMR (100 MHz, DMSO-*d*_6_) δ 164.4 (C=N), 157.2 (C-arom), 136.1 (CH=CH), 130.6, 129.2, 127.7, 124.3 (C-arom), 120.9 (CH=CH), 95.8 (C-1), 78.5 (C-2), 77.1 (C-5), 74.8 (C-3), 70.5 (C-4), 61.5 (C-6), 55.8 (OCH_3_). Anal. Calcd. for C_16_H_21_NO_6_: C, 59.43, H, 6.55, N, 4.33. Found: C, 58.83, H, 6.41, N, 4.44.

*2-Deoxy-2-[(*E,E*)-(4-hydroxy-3-methoxycinnamylidene)amino]-β-d-glucopyranose* (**48**) [20]. Method 2 (97%). M.p. 202–204 °C; [α]_D_ +21.8°; [α]_578_ +23.6°; [α]_546_ +30.2° (*c* 0.5, DMSO); IR (KBr) $\bar{\nu }$_max_/cm^−1^ 3400–2800 (OH), 1635 (C=N), 1586 (CH=CH), 1520, 1441 (arom), 1294, 1204, 1155 (C-O-C, ester), 1086, 1029, 982 (C-O), 898, 754 (arom); ^1^H NMR (400 MHz, DMSO-*d*_6_) δ 9.41 (1H, bs, OH-arom), 7.88 (1H, d, *J*_=C*H*-CH_ 8.9 Hz, N=C*H*-CH), 7.15 (1H, d, *J* 1.8 Hz H-arom), 6.97 (2H, m, *J*_CH=C*H*_ 16 Hz, H-arom, CH=C*H*-Ar), 6.76 (2H, m, H-arom, CH-C*H*=CH), 6.55 (1H, d, *J*_C1-OH_ 4.3 Hz, C1-OH), 4.97 (1H, bs, C3-OH), 4.85 (1H, bs, C4-OH), 4.62 (1H, bs, H-1), 4.58 (1H, bs, C6-OH), 3.80 (3H, s, OCH_3_), 3.72 (1H, d, *J*_6,6′_ 11.0 Hz, H-6), 3.47 (1H, m, H-6′), 3.36 (1H, t, *J*_2,3_ ≈ *J*_3,4_ 8.9 Hz H-3), 3.20 (1H, m, H-5), 3.15 (1H, q, *J*_3,4_ ≈ *J*_4,5_ 9.1 Hz, H-4), 2.69 (1H, t, *J*_1,2_ ≈ *J*_2,3_ 8.4 Hz, H-2); ^13^C{^1^H} NMR (50.3 MHz, DMSO-*d*_6_) δ 164.2 (C=N), 148.2 (C-arom), 148.2 (C-arom), 141.8 (CH=CH), 127.7, 125., 121.6, 115.9 (C-arom), 110.5 (CH=CH), 95.9 (C-1), 78.4 (C-2), 77.1 (C-5), 75.0 (C-3), 70.5 (C-4), 61.5 (C-6), 55.9 (OCH_3_). Anal. Calcd. for C_16_H_21_NO_7_: C, 56.63, H, 6.50, N, 4.17. Found: C, 56.07, H, 6.50, N, 4.12.

*2-[(*E,E*)-Cinnamylideneamino]-2-deoxy-β-d-*glycero*-l-*gluco*-heptopyranose* (**49**). Method 2 (25%). M.p. 159–161 °C; [α]_D_ −22.0°; [α]_578_ −23.4°; [α]_546_ −27.6°; [α]_436_ −67.4° (*c* 0.5, pyridine); IR (KBr) $\bar{\nu }$_max_/cm^−1^ 3300 (OH), 1634 (C=N, C=C, arom), 1082, 1018 (C-O), 868, 750, 586 (arom); ^1^H NMR (400 MHz, CDCl_3_) δ 7.92 (1H, d, *J*_=C*H*-CH_ 8.8 Hz, N=C*H*-CH), 7.60 (2H, d, *J* 7.2 Hz H-arom), 7.37 (3H, m, H-arom), 7.11 (1H, d, *J*_CH=C*H*_ 16.1 Hz, CH=C*H*-Ar), 6.91 (1H, dd, *J*_C*H*=CH_ 16.1 Hz, *J*_C*H*-CH_ 8.8 Hz, -C*H*=CH-Ar), 6.46 (1H, d, *J*_1,OH_ 7.1, OH-1), 4.81 (1H, d, *J*_3,OH_ 5.1 Hz, OH-3), 4.77 (1H, d, *J*_4,OH_ 5.4 Hz, OH-4), 4.60 (1H, t, *J*_1,2_ ≈ *J*_1,OH_ 7.5 Hz, H-1), 4.46 (1H, t, *J*_7,OH_ 5.7 Hz, OH-7), 4.24 (1H, d, *J*_6,OH_ 6.8 Hz, OH-6), 3.42 (5H, m, H-3, H-4, H-6, H-7, H-7′), 3.24 (1H, dd, *J*_4,5_ 9.1 Hz, *J*_5,6_ 0.82 Hz, H-5), 2.70 (1H, t, *J*_1,2_ ≈ *J*_2,3_ 8.3 Hz, H-2); ^13^C{^1^H} NMR (100 MHz, DMSO-*d*_6_) δ 163.8 (C=N), 141.3 (2C=C), 136.0 (C-arom), 129.3 (C-arom), 129.1 (2 C-arom), 128.6 (C-arom), 127.4 (C-arom), 96.2 (C-1), 78.6 (C-2), 75.1 (C-3), 74.7 (C-5), 69.3 (C-4), 68.9 (C-6), 62.7 (C-7). Anal. Calculated. for C_16_H_21_NO_6_: C, 59.43; H, 6.55; N, 4.33. Found: C, 59.67; H, 6.32; N, 4.21.

*2-Deoxy-2-[(*E*)-(1-naphthylmethylene)amino]-β-d-glucopyranose* (**50**). Method 2 (42%). M.p. 99–100 °C; [α]_D_ +37.0°; [α]_578_ +39.4°; [α]_546_ +46.8°; [α]_436_ +93.8° (*c* 0.5, pyridine); IR (KBr) $\bar{\nu }$_max_/cm^−1^ 3339 (OH), 1638 (C=N, arom), 1512 (arom), 1236 (C-O-C), 1078 (C-O), 874, 773 (arom); ^1^H NMR (400 MHz, DMSO-*d*_6_) δ 9.07 (1H, d, *J* 8.3 Hz, H-arom), 8.81 (1H, s, N=CH), 7.99 (2H, m, H-arom), 7.92 (1H, d, *J* 7.1 Hz, H-arom), 7.59 (3H, m, H-arom), 6.64 (1H, d, *J*_C1-OH_ 4.3 Hz, C1-OH), 5.00 (1H, d, *J*_C4-OH_ 5.1 Hz, C4-OH), 4.94 (1H, d, *J*_C3-OH_ 5.6 Hz, C3-OH), 4.85 (1H, t, *J*_1,2_ 6.3 Hz, H-1), 4.61 (1H, t, *J*_C6-OH_ 5.6 Hz, C6-OH), 3.77 (1H, dd, *J*_6,6′_ 10.9 Hz, *J*_5,6_ 0 Hz, *J*_6,OH_ 4.9 Hz, H-6), 3.53 (2H, m, *J*_2,3_ 9.0 Hz, H-3, H-6′), 3.31 (1H, m, *J*_5,6′_ 1.2 Hz, *J*_6,OH_ 5.5 Hz, H-5), 3.22 (1H, m, *J*_C4-OH_ 4.9 Hz, *J*_3,4_ 8.9 Hz, H-4), 2.98 (1H, t, *J*_1,2_ = *J*_2,3_ 8.5 Hz, H-2); ^13^C{^1^H} NMR (100 MHz, DMSO-*d*_6_) δ 162.38 (N=C), 133.7, 131.7, 131.0, 130.9, 129.5, 128.8, 127.3, 126.4, 125.6, 125.1 (C-arom), 96.0 (C-1), 79.4 (C-2), 77.2 (C-5), 74.9 (C-3), 70.6 (C-4), 61.5 (C-6). Anal. Calculated for C_17_H_19_NO_5_: C, 64.34, H, 6.03, N, 4.41. Found: C, 64.12; H, 5.87; N. 4.37.

*2-Deoxy-2-[(*E*)-(4-methoxy-1-naphthylmethylene)amino]-β-d-glucopyranose* (**51**). Method 2 (15%). M.p. 140–142 °C; [α]_578_ +11.0°; [α]_546_ +12.4° (*c* 0.5, pyridine); IR (KBr) $\bar{\nu }$_max_/cm^−1^ 3379 (OH), 1634 (C=N), 1578 (arom), 1097, 1011 (C-O), 764 (arom); ^1^H NMR (400 MHz, DMSO-*d*_6_) δ 9.24 (1H, s, arom), 8.64 (1H, s, N=CH), 8.22 (1H, d, *J* 8.5 Hz, arom), 7.83 (1H, d, *J* 8.1 Hz, arom), 7.63 (1H, t, *J* 7.7 Hz, arom), 7.55 (1H, t, *J* 7.3 Hz, arom), 7.06 (1H, d, *J* 8.1 Hz, arom), 6.58 (1H, d, *J*_C1-OH_ 6.7 Hz, C1-OH), 4.94 (1H, d, *J*_C4-OH_ 5.3 Hz, C4-OH), 4.87 (1H, d, *J*_C3-OH_ 5.7 Hz, C3-OH), 4.80 (1H, d, *J*_1,2_ 7.2 Hz, H-1), 4.57 (1H, t, *J*_C6-OH_ 5.8 Hz, C6-OH), 4.03 (3H, s, OCH_3_), 3.74 (1H,dd, *J*_6,6′_ 10.2 Hz, *J*_C6-OH_ 5.4 Hz, *J*_6,5_ 1.3 Hz, H-6), 3.53 (2H, m, *J*_6,6′_ 10.1 Hz, *J*_C6-OH_ 5.4 Hz, H-3, H-6′), 3.27 (1H, m, H-5), 3.20 (1H, m, *J*_C4-OH_ 5.3 Hz, H-4), 2.88 (1H, dd~t, *J*_1,2_ = *J*_2,3_ 8.5 Hz, H-2); ^13^C{^1^H} NMR (100 MHz, DMSO-*d*_6_) δ 162.5 (N=C), 156.8 (C-arom), 131.9 (C-arom), 131.9 (C-arom), 126.8 (C-arom), 125.8 (C-arom), 125.5 (C-arom), 125.1 (C-arom), 124.3 (C-arom), 122.0 (C-arom), 104.4 (C-arom), 96.0 (C-1), 79.4 (C-2), 77.2 (C-5), 75.1 (C-3), 70.6 (C-4), 61.5 (C-6), 56.1 (OCH_3_). Anal. Calcd. for C_18_H_21_NO_6_: C, 62.24, H, 6.09, N, 4.03. Found: C, 62.08, H, 6.13, N, 4.10.

*2-Deoxy-2-[(*E*)-(4-methoxy-1-naphthylmethylene)]amino-α-d-glucopyranose* (**52**). Method 2 (57%). [α]_D_ +127.0°; [α]_578_ +134.0°; [α]_546_ +157.4°; [α]_436_ +30.7° (*c* 0.5, pyridine); ^1^H NMR (400 MHz, DMSO-*d*_6_) δ 9.32 (1H, s, arom), 8.99 (1H, s, N=CH), 8.22 (1H, d, *J* 8.2 Hz, arom), 7.85 (1H, d, *J* 8.1 Hz, arom), 7.62 (1H, t, *J* 7.3 Hz, arom), 7.55 (1H, t, *J* 7.4 Hz, arom), 7.23 (1H, d, *J* 10.5 Hz, arom), 6.28 (1H, d, *J*_C1,OH_ 4.3 Hz, C1-OH), 5.00 (1H, t, *J*_1,2_ 3.6 Hz, H-1), 4.91 (1H, d, *J*_C4,OH_ 5.3 Hz, C4-OH), 4.72 (1H, d, *J*_C3,OH_ 5.6 Hz, C3-OH), 4.48 (1H, t, *J*_C6,OH_ 5.8 Hz, C6-OH), 4.03 (3H, s, OCH_3_), 3.89 (1H, dt, *J*_C3,OH_ 5.7 Hz, *J*_3,4_ ≈ *J*_2,3_ 9.2 Hz, H-3), 3.78 (1H, dd, *J*_6,6′_ 9.8 Hz, *J*_C6,OH_ 5.4 Hz, H-6), 3.69 (1H, dd, *J*_6,6′_ 11.6 Hz, *J*_C6,OH_ 5.4 Hz, H-6′), 3.53 (1H, m, H-5), 3.22 (1H, dt, *J*_C4,OH_ 5.4, *J*_4,5_ ≈ *J*_3,4_ 9.2 Hz, H-4), 3.15 (1H, dd, *J*_1,2_ 3.3 Hz, *J*_2,3_ 9.7 Hz, H-2); ^13^C{^1^H} NMR (100 MHz, DMSO-*d*_6_) δ 162.5 (N=C), 156.8 (C-arom), 131.9 (C-arom), 131.9 (C-arom), 126.8 (C-arom), 125.8 (C-arom), 125.5 (C-arom), 125.1 (C-arom), 124.3 (C-arom), 122.0 (C-arom), 104.4 (C-arom), 93.4 (C-1), 75.5 (C-2), 72.7 (C-5), 71.4 (C-3), 71.2 (C-4), 61.5 (C-6), 56.1 (OCH_3_). Anal. Calcd. for C_18_H_21_NO_6_: C, 62.24, H, 6.09, N, 4.03. Found: C, 62.34, H, 6.01, N, 3.87.

*2-Deoxy-2-[(*E*)-(2-naphthylmethylene)amino]-β-d-glucopyranose* (**53**). Method 2 (75%). [α]_D_ +16.0°; [α]_578_ +17.0°; [α]_546_ +20.6°; [α]_436_ +47.6° (*c* 0.5, pyridine); IR (KBr) $\bar{\nu }$_max_/cm^−1^ 3373 (OH), 1635 (C=N, arom), 1014 (C-O), 831, 752 (arom); ^1^H NMR (400 MHz, DMSO-*d*_6_) δ 8.38 (1H, s, N=CH), 8.20 (1H, s, H-arom), 8.00 (2H, m, H-arom), 7.94 (2H, m, H-arom), 7.56 (1H, m, H-arom), 6.65 (1H, d, *J*_C1-OH_ 6.7 Hz, C1-OH), 5.01 (1H, d, *J*_C4-OH_ 5.2 Hz, C4-OH), 4.94 (1H, d, *J*_C4-OH_ 5.6 Hz, C3-OH), 4.80 (1H, t, *J*_1,2_ 7.3 Hz, H-1), 4.62 (1H, t, *J*_C6-OH_ 5.8 Hz, C6-OH), 3.77 (1H, dd, *J*_5,6_ 0 Hz, *J*_6,OH_ 5.3 Hz, *J*_6,6′_ 10.3 Hz, H-6), 3.52 (2H, m, H-3, H-6′), 3.30 (1H, m, *J*_5,6′_ 1.5 Hz, H-5), 3.22 (1H, dd, *J*_C4-OH_ 5.1 Hz, *J*_3,4_ ≈ *J*_4,5_ 8.9 Hz, H-4), 2.95 (1H, t, *J*_1,2_ ≈ *J*_2,3_ 8.5 Hz, H-2); ^13^C{^1^H} NMR (100 MHz, DMSO-*d*_6_) δ 162.3 (N=C), 134.2 (C-arom), 134.1 (C-arom), 132.9 (C-arom), 129.9 (C-arom), 128.7 (C-arom), 128.3 (C-arom), 128.0 (C-arom), 127.4 (C-arom), 126.8 (C-arom), 124.1 (C-arom), 95.8 (C-1), 78.6 (C-2), 77.1 (C-5), 74.7 (C-3), 70.5 (C-4), 61.5 (C-6). Anal. Calcd. for C_17_H_19_NO_5_: C, 64.34, H, 6.03, N, 4.41. Found: C, 64.49; H, 5.97; N. 4.33.

*2-Deoxy-2-[(*E*)-(2-naphthylmethylene)amino]-α-d-glucopyranose* (**54**). Method 2 (46%). M.p. 197–199 °C; [α]_D_ +93.0°; [α]_578_ +97.2°; [α]_546_ +114.4°; [α]_436_ +229.6° (*c* 0.5, pyridine); IR (KBr) $\bar{\nu }$_max_/cm^−1^ 3374 (OH), 1636 (C=N), 1410 (arom), 1148, 1015 (C-O), 831, 752 (arom); ^1^H NMR (400 MHz, DMSO-*d*_6_) δ 8.49 (1H, s, N=CH), 8.20 (1H, s, H-arom), 8.00 (2H, m, H-arom), 7.94 (2H, m, H-arom), 7.56 (1H, m, H-arom), 6.29 (1H, d, *J*_C1,OH_ 4.3 Hz, C1-OH), 5.01 (1H, t, *J*_1,2_ 3.8 Hz, H-1), 4.96 (1H, d, *J*_C4,OH_ 5.4 Hz, C4-OH), 4.78 (1H, d, *J*_C3,OH_ 5.7 Hz, C3-OH), 4.50 (1H, t, *J*_C6,OH_ 5.8 Hz, OH-6), 3.87 (1H, dt, *J*_C3,OH_ 5.6 Hz, *J*_3,4_ ≈ *J*_2,3_ 9.1 Hz, H-3), 3.80 (1H, ddd, *J*_C6,OH_ 5.4 Hz, *J*_5,6_ 1.8 Hz, *J*_6,6′_ 9.8 Hz, H-6), 3.70 (1H, dd, *J*_4,5_ 9.5 Hz, *J*_5,6_ 1.8 Hz, H-5), 3.57 (1H, m, *J*_4,5_ ≈ *J*_3,4_ 9.3 Hz *J*_C4,OH_ 5.6 Hz H-4), 3.22 (1H, dd, *J*_1,2_ 3.3 Hz, *J*_2,3_ 9.9 Hz, H-2); ^13^C{^1^H} NMR (100 MHz, DMSO-*d*_6_) δ 162.3 (N=C), 134.3 (C-arom), 134.2 (C-arom), 132.9 (C-arom), 130.1 (C-arom), 128.7 (C-arom), 128.3 (C-arom), 128.0 (C-arom), 127.4 (C-arom), 126.8 (C-arom), 124.1 (C-arom), 93.1 (C-1), 75.4 (C-2), 72.7 (C-5), 71.1 (C-3), 71.0 (C-4), 61.5 (C-6). Anal. Calcd. for C_17_H_19_NO_5_: C, 64.34, H, 6.03, N, 4.41. Found: C, 64.12, H, 6.09, N, 4.47.

*2-Deoxy-2-[(*E*)-(9-phenantrylmethylene)amino]-β-d-glucopyranose* (**55**). Method 2 (41%). M.p. 156–158 °C; [α]_D_ +41.6°; [α]_578_ +44.2°; [α]_546_ +51.6°; [α]_436_ +115.6° (*c* 0.5, pyridine); IR (KBr) $\bar{\nu }$_max_/cm^−1^ 3358 (OH), 1645 (C=N, arom), 1450 (arom), 1078, 1037 (C-O), 723 (arom); ^1^H NMR (400 MHz, DMSO-*d*_6_) δ 9.26 (1H, dd, *J* 8.0 Hz, *J* 1.4 Hz, H-arom), 8.90 (1H, d, *J* 9.0 Hz, H-arom), 8.85 (1H, d, *J* 8.3 Hz, H-arom), 8.83 (1H, s, H-arom), 8.26 (1H, s, N=CH), 7.73 (4H, m, H-arom), 6.68 (1H, d, *J*_C1-OH_ 6.9 Hz, C1-OH), 5.00 (1H, m, *J*_C4-OH_ 5.31 Hz, C4-OH), 4.98 (1H, d, *J*_C3-OH_ 5.75 Hz, C3-OH), 4.87 (1H, t, *J*_1,2_ 7.3 Hz, H-1), 4.61 (1H, t, *J*_C6-OH_ 5.8 Hz, C6-OH), 3.78 (1H, dd, *J*_6,6′_ 9.9 Hz, *J*_5,6_ 1.4 Hz, H-6), 3.56 (2H, m, H-3, H-6′), 3.32 (1H, m, *J*_5,6_ 1.8 Hz, H-5), 3.25 (1H, m, *J*_C4,OH_ 4.7 Hz, *J*_3,4_ 8.9 Hz, H-4), 3.01 (1H, t, *J*_1,2_ = *J*_2,3_ 8.5 Hz, H-2); ^13^C{^1^H} NMR (50.3 MHz, DMSO-*d*_6_) δ 162.8 (C=N), 131.5, 130.8, 130.4, 130.3, 129.6, 129.5, 128.5, 127.4, 127.4, 127.2, 126.2, 123.1 (C-arom), 95.9 (C-1), 79.5 (C-2), 77.2 (C-5), 74.9 (C-3), 70.6 (C-4), 61.5 (C-6). Anal. Calcd. for C_21_H_21_O_5_N: C, 68.65, H, 5.76, N, 3.81. Found: C, 68.42; H, 5.54; N, 3.85.

*2-[(*E*)-(9-Antrylmethylene)amino]-2-deoxy-β-d-glucopyranose* (**56**). Method 2 (80%). M.p. 162–164 °C; [α]_578_ +3.0°; [α]_546_ –5.0° (*c* 0.5, pyridine); IR (KBr) $\bar{\nu }$_max_/cm^−1^ 3401 (OH), 1649 (C=N), 1570, 1452 (arom), 1099, 1028 (C-O), 897, 731 (arom); ^1^H NMR (400 MHz, DMSO-*d*_6_) δ 9.31 (1H, s, N=CH), 8.66 (1H, s, arom), 8.60 (2H, d, arom), 8.12 (2H, dd, arom), 7.54 (4H, dd, arom), 4.88 (1H, d, *J*_1,2_ 7.7 Hz, H-1), 3.78 (1H, d, *J*_6,6′_ 11.4 Hz, H-6), 3.65–3.38 (4H, m, H-3, 4, 5, 6′), 3.27 (1H, t, *J*_1,2_ = *J*_2,3_ 8.6 Hz, H-2); ^13^C{^1^H} NMR (100 MHz, DMSO-*d*_6_) δ 161.6 (N=C), 131.0 (2C-arom), 129.7 (C-arom), 129.4 (2 C-arom), 128.7 (2 C-arom), 128.6 (C-arom), 126.6 (3 C-arom), 125.7 (3 C-arom), 95.8 (C-1), 79.6 (C-2), 77.3 (C-5), 74.6 (C-3), 70.8 (C-4), 61.4 (C-6). Anal. Calcd. for C_21_H_21_NO_5_: C, 68.65, H, 5.76, N, 3.81. Found: C, 68.39; H, 5.92; N. 3.75.

*2-Deoxy-2-[(*E*)-(10-methyl-9-antrylmethylene)amino]-β-d-glucopyranose* (**57**). Method 2 (35%). M.p. 219–221 °C; [α]_D_ +1.6°; [α]_578_ +0.6°; [α]_546_ +0.4° (*c* 0.5, pyridine); IR (KBr) $\bar{\nu }$_max_/cm^−1^ 3414 (OH), 1659 (C=N), 1444 (arom), 1099, 1036 (C-O), 893, 750 (arom); ^1^H NMR (400 MHz, DMSO-*d*_6_) δ 9.23 (1H, s, CH=N), 8.58 (2H, d, *J* 8.5 Hz, H-arom), 8.39 (2H, d, *J* 8.7 Hz, H-arom), 7.56 (4H, m, H-arom), 6.88 (1H, d, *J*_C1-OH_ 7.2 Hz, C1-OH), 5.19 (1H, d, *J*_C4-OH_ 6.4 Hz, C4-OH), 5.06 (1H, d, *J*_C3-OH_ 4.7 Hz, C3-OH), 4.89 (1H, t, *J*_1,2_ 7.5 Hz, H-1), 4.65 (1H, t, *J*_C6-OH_ 5.8 Hz, C6-OH), 3.80 (1H, dd, *J*_6,6′_ 11.1 Hz, *J*_6,OH_ 5.8 Hz, H-6), 3.64 (1H, m, *J*_2,3_ ≈ *J*_3,4_ 8.3 Hz, H-3), 3.56 (1H, m, *J*_6,6′_ 11.6 Hz, *J*_C6-OH_ 5.8 Hz, H-6′), 3.32 (2H, m, H-4, H-5), 3.26 (1H, t, *J*_1,2_ ≈ *J*_2,3_ 8.5 Hz, H-2), 3.10 (3H, s, CH_3_); ^13^C{^1^H} NMR (50.3 MHz, DMSO-*d*_6_) δ 162.2 (N=C), 132.3, 129.4, 129.0, 128.7, 126.5, 126.0, 125.7, 125.3 (C-arom), 95.9 (C-1), 79.8 (C-2), 77.4 (C-5), 74.7 (C-3), 70.9 (C-4), 61.5 (C-6), 14.4 (CH_3_). Anal. Calcd. for C_22_H_23_NO_5_: C, 69.28, H, 6.08, N, 3.67. Found: C, 69.04, H, 5.97, N, 3.61.

*2-Deoxy-2-[(*E*)-(9-antrylmethylene)amino]-β-d-*glycero*-l-*gluco*-heptopyranose* (**58**). Method 2 (48%). M.p. 161–163 °C; [α]_578_ −5.8°; [α]_546_ 6.3° (*c* 0.5, pyridine); IR (KBr) $\bar{\nu }$_max_/cm^−1^ 3256 (OH), 1644 (C=N), 1445 (arom), 1074, 1018 (C-O), 876, 733 (arom); ^1^H NMR (400 MHz, DMSO-*d*_6_) δ 9.26 (1H, s, CH=N), 8.67 (1H, s, H-arom), 8.59 (2H, m, H-arom), 8.12 (2H, d, *J* 5.0 Hz, H-arom), 7.55 (4H, m, H-arom), 6.77 (1H, d, *J*_1,OH_ 7.3 Hz, OH-1), 5.09 (1H, d, *J*_3,OH_ 5.2 Hz, OH-3), 4.90 (1H, d, *J*_4,OH_ 4.0 Hz, OH-4), 4.84 (1H, t, *J*_1,2_ ≈ *J*_1,OH_ 7.6 Hz, H-1), 4.50 (1H, t, *J*_7,OH_ 5.2 Hz, OH-7), 4.32 (1H, d, *J*_6,OH_ 6.7 Hz, OH-6), 3.85 (1H, c, *J*_6,7_ ≈ *J*_6,7′_ ≈ *J*_6,OH_ 6.5 Hz, H-6), 3.61 (2H, m, H-3, H-4), 3.50 (2H, m, H-7, H-7′), 3.37 (1H, m, H-5), 3.24 (1H, t, *J*_1,2_ ≈ *J*_2,3_ 8.2 Hz, H-2); ^13^C{^1^H} NMR (100 MHz, DMSO-*d*_6_) δ 161.6 (C=N), 131.0 (2 C-arom), 129.7 (2 C-arom), 129.4 (2 C-arom), 128.8 (2 C-arom), 128.6 (2 C-arom), 126.7 (2 C-arom), 125.8 (3 C-arom), 96.2 (C-1), 79.8 (C-2), 75.0 (C-3), 74.9 (C-5), 69.7 (C-4), 69.0 (C-6), 63.0 (C-7). Anal. Calcd. for C_22_H_23_NO_6_·2H_2_O: C, 60.96, H, 6.28, N, 3.23. Found: C, 60.77, H, 6.32, N, 3.33.

#### 4.4.2. Synthesis of Acetyl Derivatives

As general protocol, acetic anhydride (9.0 mL) was added to a suspension of the corresponding 2-(arylmethylene)amino-2-deoxy-β-d-aldopyranose (7.1 mmol) in pyridine (9.4 mL) with stirring and external cooling, and the mixture was left at room temperature until dissolution. Then, it was poured into ice-water (*ca*. 300 mL) with stirring. The solid formed was collected by filtration and washed repeatedly with cold water and dried over silica gel.

*1,3,4,6-Tetra-*O*-acetyl-2-deoxy-2-[(*E*)-(phenylmethylene)amino]-β-d-glucopyranose* (**63**). From **30** (35%). M.p. 162–164 °C; [α]_D_ +89.0°; [α]_578_ +93.2°; [α]_546_ +109.4°, [α]_436_ +216.4° (*c* 0.5, chloroform) (Lit. [13] m.p. 160–162 °C, [α]_D_ +79.0° (c 0.5, chloroform)); IR (KBr) $\bar{\nu }$_max_/cm^−1^ 1752 (C=O), 1646 (C=N), 1581 (arom.), 1216 (C-O-C), 1083, 1055, 1055 (C-O), 755, 690 (arom.); ^1^H NMR (500 MHz, Cl_3_CD) δ 8.24 (1H, s, CH=), 7.71 (2H, d, *J* 7.0 Hz, arom), 7.46 (1H, t, *J* 7.0 Hz, arom), 7.40 (2H, t, *J* 7.0 Hz, arom), 5.97 (1H, d, *J*_1,2_ 8.0 Hz, H-1), 5.46 (1H, t, *J*_2,3_ = *J*_3,4_ 10.0 Hz, H-3), 5.15 (1H, t, *J*_3,4_ = *J*_4,5_ 10.0 Hz, H-4), 4.38 (1H, dd, *J*_5,6_ 4.5 Hz, *J*_6,6′_ 12.5 Hz, H-6), 4.14 (1H, dd, *J*_5,6′_ 2.0 Hz, *J*_6,6′_ 12.5 Hz, H-6′), 3.99 (1H, ddd, *J*_4,5_ 10.0 Hz, *J*_5,6_ 4.5 Hz, *J*_5,6′_ 2.0 Hz, H-5), 3.50 (1H, dd~t, *J*_1,2_ 8.0 Hz, *J*_2,3_ 10.0 Hz, H-2), 2.10, 2.04, 2.02, 1.89 (4 × 3H, s, CH_3_); ^13^C{^1^H} NMR (125 MHz, Cl_3_CD) δ 170.7 (C=O), 169.9 (C=O), 169.5 (C=O), 168.8 (C=O), 165.2 (CH=N), 135.3 (C-arom), 131.6 (C-arom), 128.7 (2 C-arom), 128.6 (2 C-arom), 93.1 (C-1), 73.1, 73.0 and 72.8 (C-2, C-3, C-5), 68.0 (C-4), 61.8 (C-6), 20.8, 20.8, 20.7, 20.5 (CH_3_). Anal. Calc. for C_21_H_25_O_9_N: C, 57.93, H, 5.79, N, 3.22. Found: C, 58.06, H, 5.58, N, 3.01. HRMS [M+H]^+^ calculated for C_21_H_26_NO_9_: 436.1608; Found: 436.1618; [M_2_+Na]^+^ calculated for C_42_H_50_N_2_O_18_Na: 893.2956. Found: 893.2990.

*1,3,4,6-Tetra-*O*-acetyl-2-deoxy-2-[(*E*)-(3-fluorophenylmethylene)amino]-β-d-glucopyranose* (**64**). From **31,** we obtained **64**. This reaction was conducted at −10 °C (37%); M.p.: 150–152 °C; [α]_D_ +87.0°; [α]_578_ +90.4°; [α]_546_ +105.4°, [α]_436_ +211.0° (*c* 0.5, chloroform); IR (KBr) $\bar{\nu }$_max_/cm^−1^ 1755 (C=O), 1648 (C=N), 1587 (arom.), 1216 (C-O-C), 1081, 1057, 1033 (C-O), 788 (arom.); ^1^H NMR (500 MHz, Cl_3_CD) δ 8.22 (1H, s, CH=), 7.45 (2H, m, arom), 7.39 (1H, m, arom), 7.16 (1H, m, arom), 5.96 (1H, d, *J*_1,2_ 8.5 Hz, H-1), 5.45 (1H, t, *J*_2,3_ = *J*_3,4_ 10.0 Hz, H-3), 5.15 (1H, t, *J*_3,4_ = *J*_4,5_ 10.0 Hz, H-4), 4.39 (1H, dd, *J*_5,6_ 4.5 Hz, *J*_6,6′_ 12.5 Hz, H-6), 4.14 (1H, dd, *J*_5,6′_ 2.0 Hz, *J*_6,6′_ 12.5 Hz, H-6′), 3.99 (1H, ddd, *J*_4,5_ 10.0 Hz, *J*_5,6_ 4.5 Hz, *J*_5,6′_ 2.0 Hz, H-5), 3.51 (1H, dd~t, *J*_1,2_ 8.0 Hz, *J*_2,3_ 10.0 Hz, H-2), 2.10, 2.04, 2.03, 1.90 (4 × 3H, s, CH_3_); ^13^C{^1^H} NMR (125 MHz, Cl_3_CD) δ 170.9 (C=O), 167.0 (C=O), 169.5 (C=O), 168.7 (C=O), 163.8 (^4^*J*_C,F_ = 2.5 Hz, CH=N), 163.0 (^1^*J*_C,F_ = 246.3 Hz, C3 arom), 137.5 (^3^*J*_C,F_ = 7.5 Hz, C1 arom), 130.3 (^3^*J*_C,F_ = 7.5 Hz, C5 arom), 124.7 (^4^*J*_C,F_ = 2.5 Hz, C6 arom), 118.6 (^2^*J*_C,F_ = 21.3 Hz, C4 arom), 114.7 (^2^*J*_C,F_ = 21.3 Hz, C2 arom), 93.0 (C-1), 73.0 (C-2), 72.8 (2C, C-3, C-5), 68.0 (C-4), 61.8 (C-6), 20.8, 20.8, 20.7, 20.5 (CH_3_). HRMS [M+H]^+^ calculated for C_21_H_25_NO_9_F: 454.1513; Found: 454.1513; [M_2_+Na]^+^ calculated for C_42_H_50_N_2_O_18_Na: 929.2768. Found: 929.2766.

*1,3,4,6-Tetra-*O*-acetyl-2-deoxy-2-[(*E*)-(3-bromophenylmethylene)amino]-β-d-glucopyranose* (**65**). From **32** we obtained **65** (71%); M.p.: 113–115 °C; [α]_D_ +75.4°; [α]_578_ +78.8°; [α]_546_ +93.0°, [α]_436_ +189.0° (*c* 0.5, chloroform); IR (KBr) $\bar{\nu }$_max_/cm^−1^ 1744 (C=O), 1646 (C=N), 1564, 1381 (arom.), 1223 (C-O-C), 1064, 1034 (C-O), 794 (arom.); ^1^H NMR (500 MHz, Cl_3_CD) δ 8.18 (1H, s, CH=), 7.87 (1H, t, *J* 1.5 Hz, arom), 7.63 (1H, d, *J* 7.5 Hz, arom), 7.58 (1H, d, *J* 7.5 Hz, arom), 7.29 (1H, t, *J* 7.5 Hz, arom), 5.96 (1H, d, *J*_1,2_ 8.5 Hz, H-1), 5.44 (1H, t, *J*_2,3_ = *J*_3,4_ 9.5 Hz, H-3), 5.15 (1H, t, *J*_3,4_ = *J*_4,5_ 9.5 Hz, H-4), 4.39 (1H, dd, *J*_5,6_ 4.5 Hz, *J*_6,6′_ 12.0 Hz, H-6), 4.14 (1H, dd, *J*_5,6′_ 2.0 Hz, *J*_6,6′_ 12.0 Hz, H-6′), 3.99 (1H, ddd, *J*_4,5_ 9.5 Hz, *J*_5,6_ 4.5 Hz, *J*_5,6′_ 2.0 Hz, H-5), 3.50 (1H, dd~t, *J*_1,2_ 8.0 Hz, *J*_2,3_ 9.5 Hz, H-2), 2.10, 2.04, 2.03, 1.90 (4× 3H, s, CH_3_); ^13^C{^1^H} NMR (125 MHz, Cl_3_CD) δ 170. 7 (C=O), 169.9 (C=O), 169.5 (C=O), 168.7 (C=O), 163.6 (CH=N), 137.2 (C-arom), 134.5 (C-arom), 131.3 (2 C-arom), 130.3 (C-arom), 127.2 (C-arom), 123.0 (C-arom), 92.9 (C-1), 73.0, 72.9 and 72.8 (C-2, C-3, C-5), 68.0 (C-4), 61.8 (C-6), 20.8, 20.8, 20.7, 20.5 (CH_3_). HRMS [M+H]^+^ calculated for C_21_H_25_NO_9_Br: 514.0713; Found: 514.0692.

*1,3,4,6-Tetra-*O*-acetyl-2-deoxy-2-[(*E*)-(4-chlorophenylmethylene)amino]-β-d-glucopyranose* (**66**). From **33,** we obtained **66** (76%); M.p.: 177–180 °C; [α]_D_ +96.2°; [α]_578_ +101.4°; [α]_546_ +118.2°, [α]_436_ +242.0° (*c* 0.5, chloroform); IR (KBr) $\bar{\nu }$_max_/cm^−1^ 1752 (C=O), 1643 (C=N), 1597, 1573 (arom.), 1222 (C-O-C), 1088, 1062, 1035 (C-O), 824 (arom.); ^1^H NMR (500 MHz, Cl_3_CD) δ 8.20 (1H, s, CH=), 7.65 (2H, d, *J* 8.5 Hz, arom), 7.39 (2H, d, *J* 8.5 Hz, arom), 5.95 (1H, d, *J*_1,2_ 8.5 Hz, H-1), 5.44 (1H, t, *J*_2,3_ = *J*_3,4_ 10.0 Hz, H-3), 5.15 (1H, t, *J*_3,4_ = *J*_4,5_ 10.0 Hz, H-4), 4.38 (1H, dd, *J*_5,6_ 4.5 Hz, *J*_6,6′_ 12.5 Hz, H-6), 4.14 (1H, dd, *J*_5,6′_ 2.0 Hz, *J*_6,6′_ 12.5 Hz, H-6′), 3.98 (1H, ddd, *J*_4,5_ 10.0 Hz, *J*_5,6_ 4.5 Hz, *J*_5,6′_ 2.0 Hz, H-5), 3.49 (1H, dd~t, *J*_1,2_ 8.5 Hz, *J*_2,3_ 10.0 Hz, H-2), 2.10, 2.04, 2.03, 1.90 (4× 3H, s, CH_3_); ^13^C{^1^H} NMR (125 MHz, Cl_3_CD) δ 170.7 (C=O), 169.9 (C=O), 169.5 (C=O), 168.7 (C=O), 163.7 (CH=N), 137.7 (C-arom), 133.7 (C-arom), 129.8 (2 C-arom), 129.0 (C-arom), 93.0 (C-1), 73.0, 72.9 and 72.8 (C-2, C-3, C-5), 68.0 (C-4), 61.8 (C-6), 20.8, 20.7, 20.6, 20.5 (CH_3_). HRMS [M+H]^+^ calculated for C_21_H_25_NO_9_Cl: 470.1218; Found: 470.1216.

*1,3,4,6-Tetra-*O*-acetyl-2-deoxy-2-[(*E*)-(4-piperidinylbenzylidene)amino]-β-d-glucopyranose* (**67**). From **35,** we obtained **67** (82%); M.p.: 153–155 °C; [α]_D_ +113.6°; [α]_578_ +119.2°; [α]_546_ +142.0°, [α]_436_ +343.4° (*c* 0.5, chloroform); IR (KBr) $\bar{\nu }$_max_/cm^−1^ 2954, 2872, 2811 (CH aliphatic), 1751 (C=O), 1637 (C=N), 1608, 1519 (arom.), 1366 (CH_2_), 1519 (arom.), 1220 (C-O-C), 1127, 1080, 1032 (C-O), 813 (arom.); ^1^H NMR (500 MHz, Cl_3_CD) δ 8.09 (1H, s, CH=), 7.57 (2H, d, *J* 9.0 Hz, arom), 6.87 (2H, d, *J* 9.0 Hz, arom), 5.93 (1H, d, *J*_1,2_ 8.0 Hz, H-1), 5.42 (1H, t, *J*_2,3_ = *J*_3,4_ 9.5 Hz, H-3), 5.14 (1H, t, *J*_3,4_ = *J*_4,5_ 9.5 Hz, H-4), 4.38 (1H, dd, *J*_5,6_ 4.5 Hz, *J*_6,6′_ 12.5 Hz, H-6), 4.13 (1H, dd, *J*_5,6′_ 2.0 Hz, *J*_6,6′_ 12.5 Hz, H-6′), 3.96 (1H, ddd, *J*_4,5_ 10.0 Hz, *J*_5,6_ 4.5 Hz, *J*_5,6′_ 2.0 Hz, H-5), 3.49 (1H, dd~t, *J*_1,2_ 8.0 Hz, *J*_2,3_ 10.0 Hz, H-2), 3.29 (4H, t, *J* 5.5 Hz, NCH_2_ piperidine), 2.10, 2.03, 2.02, 1.88 (4 × 3H, s, CH_3_), 1.68 (6H, m, CH_2_ piperidine); ^13^C{^1^H} NMR (125 MHz, Cl_3_CD) δ 170.7 (C=O), 169.9 (C=O), 169.6 (C=O), 168.8 (C=O), 164.6 (CH=N), 153.8 (C-arom), 130.0 (C-arom), 125.2 (2 C-arom), 114.5 (C-arom), 93.3 (C-1), 73.4 (C-3,), 73.1 (C-2), 72.7 (C-5), 68.1 (C-4), 62.0 (C-6), 49.1 (2C, NCH_2_), 25.5 (2C, CH_2_ piperidine), 24.3 (C, CH_2_ piperidine), 20.9, 20.8, 20.7, 20.6 (CH_3_). HRMS [M+H]^+^ calculated for C_26_H_35_N_2_O_9_: 519.2343; Found: 519.2354.

*1,3,4,6-Tetra-*O*-acetyl-2-deoxy-2-[(*E*)-(4-morpholinylbenzylidene)amino]-β-d-glucopyranose* (**68**). From **36,** we obtained **68** (42%); M.p.: 182–184 °C; [α]_D_ +120.2°; [α]_578_ +126.8°; [α]_546_ +151.4°, [α]_436_ +350.6° (*c* 0.5, chloroform); IR (KBr) $\bar{\nu }$_max_/cm^−1^ 2970, 2917, 2876, 2823 (CH aliphatic), 1761, 1752 (C=O), 1638 (C=N), 1610, 1519 (arom.), 1366 (CH_2_), 1250, 1229 (C-O-C), 1080, 1058, 1035 (C-O), 825 (arom.); ^1^H NMR (500 MHz, Cl_3_CD) δ 8.12 (1H, s, CH=), 7.62 (2H, d, *J* 8.5 Hz, arom), 6.88 (2H, d, *J* 8.5 Hz, arom), 5.94 (1H, d, *J*_1,2_ 8.0 Hz, H-1), 5.42 (1H, t, *J*_2,3_ = *J*_3,4_ 10.0 Hz, H-3), 5.14 (1H, t, *J*_3,4_ = *J*_4,5_ 10.0 Hz, H-4), 4.38 (1H, dd, *J*_5,6_ 4.5 Hz, *J*_6,6′_ 12.5 Hz, H-6), 4.13 (1H, dd, *J*_5,6′_ 2.0 Hz, *J*_6,6′_ 12.5 Hz, H-6′), 3.97 (1H, ddd, *J*_4,5_ 10.0 Hz, *J*_5,6_ 4.5 Hz, *J*_5,6′_ 2.0 Hz, H-5), 3.86 (4H, t, *J* 5.0 Hz, CH_2_O), 3.43 (1H, dd~t, *J*_1,2_ 8.0 Hz, *J*_2,3_ 10.0 Hz, H-2), 3.26 (4H, t, *J* 5.0 Hz, CH_2_N), 2.10, 2.04, 2.02, 1.88 (4 × 3H, s, CH_3_); ^13^C{^1^H} NMR (125 MHz, Cl_3_CD) δ 170.7 (C=O), 169.9 (C=O), 169.6 (C=O), 168.8 (C=O), 164.4 (CH=N), 153.4 (C-arom), 130.0 (2 C-arom), 126.6 (C-arom), 114.3 (2 C-arom), 93.3 (C-1), 73.4 (C-2), 73.0, 72.8 (C-3, C-5), 68.1 (C-4), 66.7 (2C, CH_2_O), 61.9 (C-6), 48.1 (2C, CH_2_N), 20.8, 20.8, 20.7, 20.5 (CH_3_). HRMS [M+H]^+^ calculated for C_25_H_33_N_2_O_10_: 521.2135; Found: 521.2136; [M_2_+Na]^+^ calculated for C_42_H_50_N_2_O_18_Na: 929.2768. Found: 929.2766.

*1,3,4,6,7-Penta-*O*-acetyl-2-deoxy-2-[(*E*)-(4-methoxybenzylidene)amino]-β-d-*glycero*-l-*gluco-*heptopyranose* (**69**) [17]. (89%). M.p. 187–189 °C; ^1^H NMR (500 MHz, CDCl_3_) δ 8.15 (1H, s, CH=N), 7.65 (2H, d, *J* 8.5 Hz, arom), 6.91 (2H, d, *J* 8.5 Hz, arom), 5.87 (1H, d, *J*_1,2_ 8.5 Hz, H-1), 5.42 (1H, t, *J*_2,3_ = *J*_3,4_ 10.0 Hz, H-3), 5.36 (1H, m, H-6), 5.13 (1H, t, *J*_3,4_ = *J*_4,5_ 10.0 Hz, H-4), 4.35 (1H, dd, *J*_6,7_ 5.0 Hz, *J*_7,7_ 11.5 Hz, H-7), 4.17 (1H, dd, *J*_6,7_ 8.0 Hz, *J*_7,7′_ 11.5 Hz, H-7′), 4.01 (1H, dd, *J*_4,5_ 10.0 Hz, *J*_5,6_ 2.0 Hz, H-5), 3.84 (3H, s, OCH_3_), 3.46 (1H, dd, *J*_1,2_ 8.5 Hz, *J*_2,3_ 9.0, H-2), 2.12, 2.06, 2.02, 2.01, 1.88 (5 × 3H, s, CH_3_); ^13^C{^1^H} NMR (125 MHz, CDCl_3_) δ 170.5, 170.3, 167.0, 169.5, 168.6 (acetate), 164.3 (N=C), 162.3 (C-arom), 130.3 (2 C-arom) 128.3, 114.1 (2 C-arom), 93.6 (C-1), 73.4 (C-2), 73.0 (C-3), 72.9 (C-5), 67.2 (C-4), 66.8 (C-6), 62.1 (C-7), 55.4 (OCH_3_), 20.8, 20.7, 20.7, 20.6, 20.5 (CH_3_). HRMS [M+H]^+^ calculated for C_25_H_32_NO_12_: 538.1919. Found: 538.1929.

*1,3,4,6,7-Penta-*O*-acetyl-2-deoxy-2-[(*E*)-(4-nitrobenzylidene)amino]-β-d-*glycero*-l-*gluco-*heptopyranose* (**70**). (63%). M.p. 208–210 °C; [α]_D_ −61.4°; [α]_578_ −64.6°; [α]_546_ −79.4°; [α]_436_ −191.2° (*c* 0.5, chloroform); IR (KBr) $\bar{\nu }$_max_/cm^−1^ 1755 (C=O), 1646 (C=N), 1602, 1522 (arom), 1246, 1221 (C-O-C, ester), 1082, 1062, 1037 (C-O), 835 (arom); ^1^H NMR (500 MHz, CDCl_3_) δ 8.31 (1H, s, CH=N), 8.25 (2H, d, *J* 8.8 Hz, H-arom), 7.08 (2H, d, *J* 8.8 Hz, H-arom), 5.91 (1H, d, *J*_1,2_ 9.0 Hz, H-1), 5.44 (1H, t, *J*_2,3_ = *J*_3,4_ 9.0 Hz, H-3), 5.35 (1H, m, *J*_5,6_ 2.0 Hz, *J*_6,7_ 5.3 Hz, *J*_6,7′_ 7.5 Hz, H-6), 5.13 (1H, t, *J*_3,4_ = *J*_4,5_ 10.0 Hz, H-4), 4.33 (1H, dd, *J*_6,7′_ 5.3 Hz, *J*_7,7′_ 12.5 Hz, H-7), 4.12 (1H, dd, *J*_6,7′_ 8.0 Hz, *J*_7,7′_ 12.5 Hz, H-7′), 3.97 (1H, dd, *J*_4,5_ 10.0 Hz, *J*_5,6_ 2.0 Hz, H-5), 3.56 (1H, t, *J*_1,2_ = *J*_2,3_ 9.0 Hz, H-2), 2.10, 2.05, 2.02, 2.00, 1.87 (5 × 3H, s, CH_3_); ^13^C{^1^H} NMR (125 MHz, CDCl_3_) δ 170.5, 170.1, 169.9, 169.3, 168.4 (C=O), 162.8 (N=CH), 149.6, 140.3, 129.3 (2 C-arom), 123.3, (2 C-arom), 93.2 (C-1), 73.0 (C-2), 72.9 (C-3, C-5), 67.0 (C-4), 66.6 (C-6), 62.0 (C-7), 20.7, 20.6, 20.5, 20.4 (2 CH_3_). Anal. Calcd. for C_24_H_28_N_2_O_13_: C, 57.73, H, 6.32, N, 5.85. Found: C, 57.52; H, 6.16; N, 5.80. HRMS [M+H]^+^ calculated for C_24_H_29_N_2_O_13_: 553.1664. Found: 553.1645.

*1,3,4,6,7-Penta-*O*-acetyl-2-deoxy-2-[(*E*)-(4-ethylbenzylidene)amino]-β-d-*glycero*-l-*gluco-*heptopyranose* (**71**). (54%) M.p. 141–144 °C; [α]_D_ −41.0°; [α]_578_ −43.0°; [α]_546_ −52.0°; [α]_436_ −118.6° (*c* 0.5, chloroform); IR (KBr) $\bar{\nu }$_max_/cm^−1^ 1750 (C=O), 1644 (C=N), 1610 (arom), 1222 (C-O-C), 1078, 1033 (C-O), 895 and 832 (arom); ^1^H NMR (500 MHz, CDCl_3_) δ 8.20 (1H, s, CH=N), 7.63 (2H, d, *J* 8.0 Hz, H-arom), 7.25 (2H, d, *J* 8.0 Hz, H-arom), 5.88 (1H, d, *J*_1,2_ 8.5 Hz, H-1), 5.43 (1H, t, *J*_2,3_ = *J*_3,4_ 9.5 Hz, H-3), 5.36 (1H, m, *J*_6,7_ 5.5 Hz, *J*_6,7′_ 8.0 Hz, H-6), 5.13 (1H, t, *J*_3,4_ = *J*_4,5_ 10.0 Hz, H-4), 4.35 (1H, dd, *J*_6,7′_ 5.5 Hz, *J*_7,7′_ 12.0 Hz, H-7), 4.17 (1H, dd, *J*_6,7′_ 8.0 Hz, *J*_7,7′_ 12.0 Hz, H-7′), 4.02 (1H, dd, *J*_4,5_ 10.0 Hz, *J*_5,6_ 2.0 Hz, H-5), 3.48 (1H, t, *J*_1,2_ = *J*_2,3_ 9.0 Hz, H-2), 2.68 (1H, c, *J* 7.5 Hz, CH_2_), 2.12, 2.06, 2.02, 1.88 (5 × 3H, s, CH_3_), 2.68 (1H, c, *J* 7.5 Hz, CH_3_); ^13^C{^1^H} NMR (125 MHz, CDCl_3_) δ 170.5, 170.2, 170.0, 169.5, 168.5 (C=O), 165.0 (N=CH), 148.3, 132.9, 128.7 (2 C-arom), 128.2, (2 C-arom), 93.5 (C-1), 73.2 (C-2), 73.0 and 72.9 (C-3, C-5), 67.2 (C-4), 66.7 (C-6), 62.1 (C-7), 28.9 (*C*H_2_CH_3_), 20.7, 20.7, 20.6, 20.6, 20.5 (CH_3_ acetyl), 15.3 (CH_2_*C*H_3_). Anal. Calcd. for C_26_H_33_NO_11_: C, 57.73, H, 6.32, N, 5.85. Found: C, 57.58; H, 6.11; N, 5.92. HRMS [M+H]^+^ calculated for C_26_H_34_NO_11_: 536.2126. Found: 536.2112.

*1,3,4,6,7-Penta-*O*-acetyl-2-deoxy-2-[(*E*)-(2,4-dimethylbenzylidene)amino]-β-d-*glycero*-l-*gluco-*heptopyranose* (**72**). (48%); ^1^H NMR (500 MHz, CDCl_3_) δ 8.50 (1H, s, CH=N), 7.65 (2H, d, *J* 7.8 Hz, H-arom), 7.06 (2H, d, *J* 7.8 Hz, H-arom), 7.02 (1H, s, H-arom), 5.91 (1H, d, *J*_1,2_ 9.0 Hz, H-1), 5.47 (1H, t, *J*_2,3_ = *J*_3,4_ 9.5 Hz, H-3), 5.38 (1H, m, *J*_6,7_~*J*_6,7′_ 5.5 Hz, H-6), 5.15 (1H, t, *J*_3,4_ = *J*_4,5_ 10.0 Hz, H-4), 4.36 (1H, dd, *J*_6,7′_ 5.3 Hz, *J*_7,7′_ 11.8 Hz, H-7), 4.33 (1H, dd, *J*_6,7′_ 8.0 Hz, *J*_7,7′_ 11.8 Hz, H-7′), 4.04 (1H, dd, *J*_4,5_ 10.0 Hz, *J*_5,6_ 2.0 Hz, H-5), 3.49 (1H, t, *J*_1,2_ = *J*_2,3_ 9.0 Hz, H-2), 2.46, 2.35 (2xMe, arom), 2.13, 2.08, 2.05, 2.04, 1.92 (5 × 3H, s, CH_3_). Anal. Calcd. for C_26_H_33_NO_11_: C, 57.73, H, 6.32, N, 5.85. Found: C, 57.61; H, 6.07; N, 5.83. HRMS [M+H]^+^ calculated for C_26_H_34_NO_11_: 536.2126. Found: 536.2121.

*1,3,4,6-Tetra-*O*-acetyl-2-[(*E,E*)-cinnamylideneamino]-2-deoxy-β-d-glucopyranose* (**73**). (45%). M.p. 217–218 °C; [α]_D_ +58.8°; [α]_578_ +62.4°; [α]_546_ +75.0°; [α]_436_ +185.2°; [α]_365_ +530.0° (*c* 0.5, chloroform); IR (KBr) $\bar{\nu }$_max_/cm^−1^ 2912 (OCH_3_), 1757 (C=O), 1638 (C=N, arom), 1229 (C-O-C), 1169, 1036 (C-O), 760, 694 (arom); ^1^H NMR (400 MHz, CDCl_3_) δ 7.99 (1H, d, *J*_=CH-CH_ 8.8 Hz, N=C*H*-CH), 7.49 (2H, d, *J* 8.4 Hz, H-arom), 7.36 (3H, m, H-arom), 7.03 (1H, d, *J*_CH=C*H*_ 16.1 Hz, CH=C*H*-Ar), 6.86 (1H, dd, *J*_C*H*=CH_ 16.1 Hz, *J*_C*H*-CH_ 8.8 Hz, CH-C*H*=CH), 5.99 (1H, d, *J*_1,2_ 8.1 Hz, H-1), 5.38 (1H, t, *J*_2,3_ = *J*_3,4_ 9.5 Hz, H-3), 5.13 (1H, t, *J*_3,4_ = *J*_4,5_ 9.8 Hz, H-4), 4.37 (1H, dd, *J*_6,6′_ 12.4 Hz, *J*_5,6_ 4.5 Hz, H-6), 4.12 (1H, dd, *J*_6,6′_ 12.4 Hz, *J*_5,6′_ 2.1 Hz, H-6′), 3.95 (1H, ddd, *J*_4,5_ 10.1 Hz, *J*_5,6_ 4.5 Hz, *J*_5,6′_ 2.1 Hz, H-5), 3.38 (1H, t, *J*_1,2_ = *J*_2,3_ 9.0 Hz, H-2), 2.10, 2.07, 2.04, 1.96 (4 × 3H, s, CH_3_); ^13^C{^1^H} NMR (100 MHz, CDCl_3_) δ 170.7, 169.9. 169.6, 168.7 (4C=O), 166.8 (N=C), 143.9 (2C, CH=CH), 135.1, 129.8, 128.9 (C-arom), 127.5 (2 C-arom), 127.3 (C-arom), 93.1 (C-1), 73.2 (C-2), 73.0 (C-5), 72.7 (C-3), 68.0 (C-4), 61.8 (C-6), 20.8, 20.7, 20.7, 20.5 (CH_3_). Anal. Calcd. for C_23_H_27_NO_9_: C, 59.86, H, 5.90, N, 3.04. Found: C, 59.80, H, 5.94, N, 2.76.

*1,3,4,6-Tetra-*O*-acetyl-2-deoxy-2-[(*E,E*)-(4-nitrocinnamylidene)amino]-β-d-glucopyranose* (**74**). (73%). M.p. 87–89 °C; [α]_D_ +54.0°; [α]_578_ +59.8°; [α]_546_ +71.0°; [α]_436_ +174.0° (*c* 0.5, chloroform); IR (KBr) $\bar{\nu }$_max_/cm^−1^ 2955 (OCH_3_), 1753 (C=O), 1682 (C=N), 1522 (arom), 1219 (C-O-C), 1034 (C-O), 746 (arom); ^1^H NMR (400 MHz, CDCl_3_) δ 8.26 (1H, dd, *J* 9.0 Hz, H-arom), 8.03 (1H, d, *J*_=C*H*-CH_ 8.5 Hz, N=C*H*-CH), 7.63 (2H, dd, *J* 11.1 Hz, *J* 2.2 Hz, H-arom), 7.08 (1H, d, *J*_CH=C*H*_ 16.1 Hz, CH=C*H*-Ar), 6.97 (1H, dd, *J*_C*H*=CH_ 16.2 Hz, *J*_C*H*-CH_ 8.5 Hz, CH-C*H*=CH), 5.91 (1H, d, *J*_1,2_ 8.3 Hz, H-1), 5.41 (1H, t, *J*_2,3_ = *J*_3,4_ 9.7 Hz, H-3), 5.14 (1H, t, *J*_3,4_ = *J*_4,5_ 9.8 Hz, H-4), 4.38 (1H, dd, *J*_6,6′_ 12.5 Hz, *J*_5,6_ 4.5 Hz, H-6), 4.13 (1H, dd, *J*_6,6′_ 12.4 Hz, *J*_5,6′_ 1.9 Hz, H-6′), 3.97 (1H, ddd, *J*_4,5_ 10.1 Hz, *J*_5,6_ 4.5 Hz, *J*_5,6′_ 2.0 Hz, H-5), 3.43 (1H, dd, *J*_1,2_ 8.4 Hz, *J*_2,3_ 9.6 Hz, H-2), 2.10, 2.07, 2.06, 1.97 (4 × 3H, s, CH_3_); ^13^C{^1^H} NMR (100 MHz, CDCl_3_) δ 170.7, 169.9, 169.5, 168.6 (C=O), 165.7 (C=N), 148.0 (C-arom), 140.6 (CH=CH), 131.3, 129.0, 128.0 (C-arom), 124.2 (CH=CH), 92.9 (C-1), 73.0 (C-2), 72.7 (C-5), 67.8 (C-3), 67.8 (C-4), 61.6 (C-6), 20.8, 20.7, 20.6, 20.5 (CH_3_). Anal. Calcd. for C_23_H_26_N_2_O_11_: C, 54.54, H, 5.17, N, 5.53. Found: C, 54.71; H, 5.34; N, 5.38.

*1,3,4,6,7-Penta-*O*-acetyl-2-[(*E,E*)-cinnamylideneamino]-2-deoxy-β-d-glycero-l-gluco-heptopyranose* (**75**). (50%). M.p. 184–187 °C; [α]_D_ −10.0°; [α]_578_ −11.0°; [α]_546_ −14.6°; [α]_436_ −58.2° (*c* 0.5, chloroform); IR (KBr) $\bar{\nu }$_max_/cm^−1^ 1750 (C=O), 1636 (C=N), 1433 (C=C, arom), 1215 (C-O-C), 1036 (C-O), 754, 692 (arom); ^1^H NMR (400 MHz, CDCl_3_) δ 7.98 (1H, s, CH=N), 7.46 (2H, m, H-arom), 7.37 (3H, m, H-arom), 7.02 (1H, d, *J*_CH=C*H*_ 16.0 Hz, CH=C*H*-Ar), 6.85 (1H, dd, *J*_C*H*=CH_ 16.0 Hz, *J*_C*H*-CH_ 8.8 Hz, -C*H*=CH-Ar), 5.64 (1H, d, *J*_1,2_ 8.8 Hz, H-1), 5.36 (2H, m, *J*_2,3_ = *J*_3,4_ 9.6 Hz, H-3 y 6), 5.13 (1H, t, *J*_3,4_ = *J*_4,5_ 9.7 Hz, H-4), 4.33 (1H, dd, *J*_6,7_ 5.1 Hz, *J*_7,7′_ 11.7 Hz, H-7), 4.15 (1H, dd, *J*_6,7′_ 7.9 Hz, *J*_7,7′_ 11.5 Hz, H-7′), 3.98 (1H, dd, *J*_5,6_ 2.0 Hz, *J*_4,5_ 10.1 Hz, H-5), 3.39 (1H, t, *J*_1,2_ ≈ *J*_2,3_ 9.0 Hz, H-2), 2.14, 2.11, 1.95 (4 × 3H, s, CH_3_); ^13^C{^1^H} NMR (100 MHz, CDCl_3_) δ 170.5, 170.2, 169.9, 168.5 (C=O), 166.8 (C=N), 144.0 (2C=C), 135.0 (C-arom), 129.8 (C-arom), 128.9 (C-arom), 127.4 (2 C-arom), 127.2 (C-arom), 93.4 (C-1), 73.2 (C-2), 72.9 (C-3), 72.9 (C-5), 67.1 (C-4), 66.7 (C-6), 62.1 (C-7), 20.6, 20.5, 20.5 (CH_3_, acetate). Anal. Calcd. for C_26_H_31_NO_11_: C, 58.53, H, 5.86, N, 2.63. Found: C, 58.31, H, 5.89, N, 2.73.

*1,3,4,6-Tetra-*O*-acetyl-2-deoxy-2-[(*E*)-(1-naphthylmethylene)amino]-β-d-glucopyranose* (**76**). From **50** as an oil (43%). ^1^H NMR (400 MHz, CDCl_3_) δ 8.89 (1H, s, CH=N), 8.79 (1H, d, *J* 8.8 Hz, H-arom), 7.95 (1H, d, *J* 8.8 Hz, H-arom), 7.90 (1H, d, *J* 8.4 Hz, H-arom), 7.84 (1H, d, *J* 7.2 Hz, H-arom), 7.61 (1H, c, *J* 7.0 Hz, H-arom), 7.53 (1H, c, *J* 8.3 Hz, H-arom), 6.07 (1H, d, *J*_1,2_ 8.3 Hz, H-1), 5.58 (1H, t, *J*_2,3_ = *J*_3,4_ 9.8 Hz, H-3), 5.21 (1H, t, *J*_3,4_ = *J*_4,5_ 9.8 Hz, H-4), 4.42 (1H, dd, *J*_6,6′_ 12.4 Hz, *J*_5,6_ 4.4 Hz, H-6), 4.17 (1H, dd, *J*_6,6′_ 12.6 Hz, *J*_5,6′_ 1.4 Hz, H-6′), 4.04 (1H, ddd, *J*_4,5_ 10.0 Hz, *J*_5,6_ 4.0 Hz, *J*_5,6′_ 2.1 Hz, H-5), 3.61 (1H, t, *J*_1,2_ 8.8 Hz, H-2), 2.12, 2.06, 2.02, 1.89 (4 × 3H, s, CH_3_); ^13^C{^1^H} NMR (100 MHz, CDCl_3_) δ 170.7, 169.9, 169. 6, 168.7 (C=O), 165.1 (C=N), 132.0, 128.7, 127.6, 126.3 (C-arom), 93.1 (C-1), 73.9 (C-2), 73.2 (C-5), 72.8 (C-3), 68.0 (C-4), 61.8 (C-6), 20.7, 20.7, 20.5, 18.4 (CH_3_). Anal. Calcd. for C_25_H_27_NO_9_: C, 61.85, H, 5.61, N, 2.89. Found: C, 62.03; H, 5.50; N, 2.87.

*1,3,4,6-Tetra-*O*-acetyl-2-deoxy-2-[(*E*)-(4-methoxy-1-naphthylmethylene)amino]-β-d-glucopyranose* (**77**). From **51** (50%). M.p. 150–152 °C; [α]_D_ +87.2°; [α]_578_ +91.0°; [α]_546_ +106.4°; [α]_436_ +231.0° (*c* 0.5, chloroform); IR (KBr) $\bar{\nu }$_max_/cm^−1^ 2953 (OCH_3_), 1753 (C=O), 1640 (C=N), 1514 (arom), 1219 (C-O-C), 1032 (C-O), 765 (arom); ^1^H NMR (400 MHz, CDCl_3_) δ 8.88 (1H, d, *J* 8.5 Hz, H-arom), 8.74 (1H, s, CH=N), 8.32 (1H, d, *J* 7.6 Hz, H-arom), 7.77 (1H, d, *J* 8.1 Hz, H-arom), 7.61 (1H, t, *J* 7.8 Hz, H-arom), 7.53 (1H, t, *J* 8.0 Hz, H-arom), 6.84 (1H, d, *J* 8.2 Hz, H-arom), 6.04 (1H, d, *J*_1,2_ 8.5 Hz, H-1), 5.55 (1H, t, *J*_2,3_ = *J*_3,4_ 9.5 Hz, H-3), 5.20 (1H, t, *J*_3,4_ = *J*_4,5_ 9.8 Hz, H-4), 4.40 (1H, dd, *J*_6,6′_ 12.3 Hz, *J*_5,6_ 4.4 Hz, H-6), 4.16 (1H, dd, *J*_6,6′_ 12.3 Hz, *J*_5,6′_ 1.8 Hz, H-6′), 4.05 (1H, ddd, *J*_4,5_ 10.2 Hz, *J*_5,6_ 4.5 Hz, *J*_5,6′_ 2.3 Hz, H-5), 3.53 (1H, dd, *J*_1,2_ 8.4 Hz, *J*_2,3_ 9.6 Hz, H-2), 2.12, 2.05, 2.02, 1.87 (4 × 3H, s, CH_3_); ^13^C{^1^H} NMR (100 MHz, CDCl_3_) δ 170.7, 169.56, 169.0, 160.8 (C=O), 160.8 (C=N), 139.6, 132.5, 127.9, 125.6, 124.5, 123.5, 122.4 (C-arom), 93.3 (C-1), 73.4 (C-2), 72.8 (C-5), 71.7 (C-3), 68.1 (C-4), 61.8 (C-6), 55.9 (OCH_3_), 21.0, 20.7, 20.7, 20.6 (CH_3_). Anal. Calcd. for C_29_H_29_NO_10_: C, 60.58, H, 5.67, N, 2.72. Found: C, 60.29, H, 5.67, N, 2.59.

*1,3,4,6-Tetra-*O*-acetyl-2-deoxy-2-[(*E*)-(4-methoxy-1-naphthylmethylene)amino]-α-d-glucopyranose* (**78**). From **52,** a mixture of **77** and **78** was isolated. Crystallization from ethanol gave **78** (32%); ^1^H NMR (400 MHz, CDCl_3_) δ 9.01 (1H, d, *J* 8.1 Hz, H-arom), 8.74 (1H, s, CH=N), 8.31 (1H, d, *J* 8.1 Hz, H-arom), 7.71 (1H, d, *J* 8.1 Hz, H-arom), 7.56 (1H, dt, *J* 7.6 Hz, *J* 1.5 Hz, H-arom), 7.51 (1H, dt, *J* 8.1 Hz, *J* 1.2 Hz, H-arom), 6.83 (1H, d, *J* 8.1 Hz, H-arom), 6.32 (1H, d, *J*_1,2_ 3.6 Hz, H-1), 5.70 (1H, t, *J*_2,3_ = *J*_3,4_ 9.8 Hz, H-3), 5.23 (1H, t, *J*_3,4_ = *J*_4,5_ 9.9, H-4), 4.38 (1H, dd, *J*_6,6′_ 12.3 Hz, *J*_5,6_ 4.1 Hz, H-6), 4.29 (1H, ddd, *J*_4,5_ 10.2 Hz, *J*_5,6_ 4.0 Hz, *J*_5,6′_ 2.1 Hz, H-5), 4.14 (1H, dd, *J*_5,6′_ 2.0 Hz, *J*_6,6′_ 12.3 Hz, H-6′), 3.73 (1H, dd, *J*_1,2_ 3.6 Hz, *J*_2,3_ 10.1 Hz, H-2), 2.25, 2.13, 2.06, 1.88 (4 × 3H, s, CH_3_); ^13^C{^1^H} NMR (100 MHz, CDCl_3_) δ 170.7, 169.6, 169.0, 160.8 (C=O), 158.3 (CH=N), 139.6, 132.5, 127.9, 125.6, 124.5, 123.5, 122.4 (C-arom), 91.9 (C-1), 71.8 (C-2), 71.3 (C-5), 70.1 (C-3), 68.3 (C-4), 61.9 (C-6), 55.7 (OCH_3_), 21.0, 20.7, 20.7, 20.6 (4 × CH_3_). Anal. Calcd. for C_29_H_29_NO_10_: C, 60.58, H, 5.67, N, 2.72. Found: C, 60.81, H, 5.53, N, 2.75.

*1,3,4,6-Tetra-*O*-acetyl-2-deoxy-2-[(*E*)-(2-naphthylmethylene)amino]-β-d-glucopyranose* (**79**). From **53** (30%); [α]_D_ +86.2°; [α]_578_ +91.0°; [α]_546_ +106.4°; [α]_436_ +224.4°; [α]_365_ +500.4° (*c* 0.5, chloroform); $\bar{\nu }$_max_/cm^−1^ 1752 (C=O), 1641 (C=N), 1430 (arom), 1222 (C-O-C), 1024 (C-O); ^1^H NMR (400 MHz, CDCl_3_) δ 8.55 (1H, s, CH=N), 8.23 (1H, d, arom), 8.00 (1H, m, arom), 7.90 (3H, m, arom), 7.57 (2H, m, arom), 6.18 (1H, d, *J*_1,2_ 8.4 Hz, H-1), 5.56 (1H, t, *J*_3,4_ = *J*_4,5_ 9.8 Hz, H-4), 5.06 (1H, t, *J*_2,3_ = *J*_3,4_ 9.6 Hz, H-3), 4.33 (1H, dd, *J*_5,6_ 3.2 Hz, *J*_4,5_ 8.0 Hz, H-5), 4.27 (1H, dd, *J*_5,6_ 4.4 Hz, *J*_6,6′_ 12.0 Hz, H-6), 4.06 (1H, d, *J*_6,6′_ 11.2 Hz, H-6′), 3.88 (1H, t, *J*_1,2_ = *J*_2,3_ 8.8 Hz, H-2), 2.03, 2.00, 1.98, 1.83 (4 × 3H, s, CH_3_); ^13^C{^1^H} NMR (100 MHz, CDCl_3_) δ 170.5, 169.8, 169.4, 168.6 (C=O), 165.0 (N=CH), 134.9, 132.9, 132.7, 130.7, 128.5, 127.8, 127.5, 126.5, 123.7 (C-arom), 93.0 (C-1), 73.0 (C-2), 73.0 (C-5), 72.7 (C-3), 67.9 (C-4), 61.7 (C-6), 20.7, 20.6, 20.4 (4 CH_3_). Anal. Calcd. for C_25_H_27_NO_9_: C, 61.85, H, 5.61, N, 2.89. Found: C, 61.68; H, 5.73; N, 2.95.

*1,3,4,6-Tetra-*O*-acetyl-2-deoxy-2-(*E*)-(9-phenantrylmethylene)amino]-β-d-glucopyranose* (**81**). From **55** (90%). M.p. 99–100 °C; [α]_D_ +69.6°; [α]_578_ +74.0°; [α]_546_ +87.4°; [α]_436_ +199.8° (*c* 0.5, chloroform); IR (KBr) $\bar{\nu }$_max_/cm^−1^ 1755 (C=O), 1641 (C=N, arom), 1219 (C-O-C), 1036 (C-O), 752, 601 (arom); ^1^H NMR (400 MHz, CDCl_3_) δ 8.96 (1H, dd, *J* 8.0 Hz, *J* 1.6 Hz, H-arom), 8.89 (1H, s, H-arom), 8.74 (1H, dd, H-arom), 8.68 (1H, d, *J* 8.3 Hz, H-arom), 8.09 (1H, s, N=CH), 7.96 (1H, d, *J* 7.6 Hz, H-arom), 7.68 (4H, m, H-arom), 6.10 (1H, d, *J*_1,2_ 6.2 Hz, H-1), 5.61 (1H, t, *J*_2,3_ = *J*_3,4_ 9.6 Hz, H-3), 5.23 (1H, t, *J*_3,4_ = *J*_4,5_ 9.8 Hz, H-4), 4.42 (1H, dd, *J*_6,6′_ 12.4 Hz, *J*_5,6_ 4.5 Hz, H-6), 4.18 (1H, dd, *J*_6,6′_ 12.4 Hz, *J*_5,6′_ 2.1 Hz, H-6′), 4.06 (1H, ddd, *J*_4,5_ 10.1 Hz, *J*_5,6_ 4.4 Hz, *J*_5,6′_ 2.0 Hz, H-5), 3.63 (1H, dd, *J*_1,2_ 8.3 Hz, *J*_2,3_ 9.7 Hz, H-2), 2.11, 2.08, 2.06, 1.90 (4 × 3H, s, CH_3_); ^13^C{^1^H} NMR (100 MHz, DMSO-*d*_6_) δ 170.7, 169.9, 169.6, 168.7 (C=O), 165.7 (C=N), 132.5, 131.6, 130.6, 129.7, 127.4, 127.0 (2 C-arom), 125.2, 123.0, 122.6 (C-arom), 93.1 (C-1), 74.0 (C-2), 73.2 (C-5), 72.8 (C-3), 68.0 (C-4), 61.8 (C-6). 20.8 (2 CH_3_), 20.7 (CH_3_), 20.5 (2 CH_3_) Anal. Calcd. for C_29_H_29_NO_9_: C, 65.04, H, 5.46, N, 2.62. Found: C, 64.82, H, 5.57, N, 2.74.

*1,3,4,5-Tetra-O-acetyl-2-[(*E*)-(9-antrylmethylene)amino]-2-deoxy-β-d-glucopyranose* (**82**). From **56** (68%). M.p. 164–167 °C; [α]_D_ +21.2°; [α]_578_ +21.8°; [α]_546_ +26.2° (*c* 0.5, chloroform); IR (KBr) $\bar{\nu }$_max_/cm^−1^ 1753 (C=O), 1651 (C=N, arom), 1217 (C-O-C), 1041 (C-O), 903, 733 (arom); ^1^H NMR (400 MHz, CDCl_3_) δ 9.46 (1H, s, CH=N), 8.37 (1H, d, arom), 8.08 (1H, s, arom), 8.03 (1H, d, arom), 7.50 (4H, m, arom), 7.30 (2H, m, arom), 6.14 (1H, d, *J*_1,2_ 8.1 Hz, H-1), 5.71 (1H, t, *J*_2,3_ = *J*_3,4_ 9.6 Hz, H-3), 5.27 (1H, t, *J*_3,4_ = *J*_4,5_ 9.8 Hz, H-4), 4.45 (1H, dd, *J*_5,6_ 4.4 Hz, *J*_6,6′_ 12.3 Hz, H-6), 4.20 (1H, dd, *J*_5,6′_ 1.6 Hz, *J*_6,6′_ 12.4 Hz, H-6′), 4.10 (1H, ddd, *J*_4,5_ 9.8 Hz, *J*_5,6_ 4.5 Hz, *J*_5,6′_ 1.7 Hz, H-5), 3.88 (1H, t, *J*_1,2_ = *J*_2,3_ 9.1 Hz, H-2), 2.14, 2.08, 2.07, 2.03 (4 × 3H, s, CH_3_); ^13^C{^1^H} NMR (100 MHz, CDCl_3_) δ 170.6, 170.0, 169.8, 169.7 (C=O), 165.6 (N=CH), 138.1, 137.9, 137.8, 137.7, 130.2, 128.1, 127.6, 127.3, 126.8, 123.5, 121.9, 121.6 (C-arom), 93.0 (C-1), 73.2 (C-2), 72.8 (C-5), 72.8 (C-3), 68.3 (C-4), 61.7 (C-6), 20.7, 20.6 (4 CH_3_). Anal. Calcd. for C_29_H_29_NO_9_: C, 65.04, H, 5.46, N, 2.62; Found: C, 64.79, H, 5.23, N, 2.83.

*1,3,4,6-Tetra-*O*-acetyl-2-deoxy-2-[(*E*)-(10-methyl-9-antrylmethylene)amino]-β-d-glucopyranose* (**83**). From **57** (92%). M.p. 225–226 °C; [α]_D_ +59.2°; [α]_578_ +63.8°; [α]_546_ +80.2° (*c* 0.5, chloroform); IR (KBr) $\bar{\nu }$_max_/cm^−1^ 2915 (OCH_3_), 1746 (C=O), 1647 (C=N, arom), 1215 (C-O-C), 1084, 1032 (C-O), 750 (arom); ^1^H NMR (400 MHz, CDCl_3_) δ 9.43 (1H, s, C=N), 8.34 (4H, m, H-arom), 7.52 (4H, m, H-arom), 6.13 (1H, d, *J*_1,2_ 8.3 Hz, H-1), 5.71 (1H, t, *J*_2,3_ = *J*_3,4_ 9.6 Hz, H-3), 5.28 (1H, t, *J*_3,4_ = *J*_4,5_ 9.7 Hz, H-4), 4.45 (1H, dd, *J*_6,6′_ 12.5 Hz, *J*_5,6_ 4.4 Hz, H-6), 4.19 (1H, dd, *J*_6,6′_ 12.5 Hz, *J*_5,6′_ 1.9 Hz, H-6′), 4.07 (1H, ddd, *J*_4,5_ 10.1 Hz, *J*_5,6_ 4.3 Hz, *J*_5,6′_ 1.9 Hz, H-5), 3.89 (1H, dd, *J*_1,2_ 8.5 Hz, *J*_2,3_ 9.7 Hz, H-2), 2.17, 2.14, 2.08, 2.06 (4 × 3H, s, CH_3_); ^13^C{^1^H} NMR (100 MHz, DMSO-*d*_6_) δ 170.7, 170.0, 169.7, 168.6 (C=O), 166.3 (C=N), 134.2, 129.6, 129.4, 126.3, 125.8, 125.4, 125.3, 124.6 (C-arom), 92.9 (C-1), 74.6 (C-2), 73.2 (C-5), 72.8 (C-3), 68.2 (C-4), 61.7 (C-6), 20.8 (2 CH_3_), 20.7 (2 CH_3_), 14.6 (CH_3_). Anal. Calcd. for C_30_H_31_NO_9_: C, 65.56, H, 5.69, N, 2.55; Found: C, 65.03, H, 5.37, N, 2.39.

*1,3,4,6,7-Penta-O-acetyl-2-deoxy-2-[(*E*)-(9-antrylmethylene)amino]-β-d-*glycero*-l-*gluco*-heptopyranose* (**84**). From **58** (99%). M.p. 176–177 °C; [α]_578_ −25.6°; [α]_546_ −32.6° (*c* 0.5, chloroform); IR (KBr) $\bar{\nu }$_max_/cm^−1^ 1751 (C=O), 1634 (C=N), 1443 (arom), 1219 (C-O-C), 1035 (C-O), 737 (arom); ^1^H NMR (400 MHz, CDCl_3_) δ 9.45 (1H, s, CH=N), 8.53 (1H, s, H-arom), 8.35 (2H, d, *J* 8.2 Hz, H-arom), 8.02 (2H, d, *J* 7.7 Hz, H-arom), 7.52 (4H, m, H-arom), 6.06 (1H, d, *J*_1,2_ 8.3 Hz, H-1), 5.71 (1H, t, *J*_3,4_ = *J*_4,5_ 9.6 Hz, H-4), 5.42 (1H, ddd, *J*_5,6_ 1.4 Hz, *J*_6,7_ 5.4 Hz, *J*_6,7′_ 7.8 Hz, H-6), 5.25 (1H, t, *J*_2,3_ = *J*_3,4_ 9.7, H-3), 4.39 (1H, dd, *J*_6,7_ 5.2 Hz, *J*_7,7′_ 11.4 Hz, H-7), 4.12 (1H, dd, *J*_6,7′_ 7.9 Hz, *J*_7,7′_ 11.4 Hz, H-7′), 4.12 (1H, dd, *J*_4,5_ 10.0 Hz, *J*_5,6_ 1.4 Hz, H-5), 3.89 (1H, dd, *J*_1,2_ ≈ *J*_2,3_ 9.0 Hz, H-2), 2.16, 2.14, 2.08, 2.06, 2.03 (4 × 3H, s, CH_3_); ^13^C{^1^H} NMR (100 MHz, CDCl_3_) δ 170.5, 170.2, 169.6, 168.5 (C=O), 165.7 (C=N), 132.0 (2 C-arom), 131.1, 130.3 (C-arom), 129.2 (2 C-arom), 129.1 (2 C-arom), 127.1, 126.7 (C-arom), 125.4 (2 C-arom), 123.9 (2 C-arom), 93.9 (C-1), 74.5 (C-2), 73.3 (C-3), 73.0 (C-5), 67.5 (C-4), 66.7 (C-6), 62.1 (C-7), 20.7 (3C, CH_3_, acetate), 20.6 (3C, CH_3_, acetate). Anal. Calcd. for C_32_H_33_NO_11_: C, 63.26, H, 5.47, N, 2.31. Found: C, 63.53, H, 5.62, N, 2.45. HRMS [M+H]^+^ calculated for C_32_H_34_NO_11_: 608.2132. Found: 608.2120.

*1,3,4,5-Tetra-*O*-acetyl-2-deoxy-2-[(2-methoxy-1-naphthyl)methylene]amino-β-d-glucopyranose* (**87**). To a solution of 1,3,4,5-tetra-*O*-acetyl-2-amino-2-deoxy-β-d-glucopyranose hydrochloride (**85**) (1.3 g, 3.3 mmol) in ethanol (14 mL), anhydrous sodium acetate (0.25 g) dissolved in water (2 mL) and 2-methoxynaphthaldehyde (0.6 g, 3.0 mmol) were added. The solution was heated in a water bath, filtered to remove impurities, and then cooled. The crystalline solid crystallized was filtered and washed with 96% aqueous ethanol to afford the title compound in 21% yield. M.p.: 196–198 °C; [α]_D_ +73.6°; [α]_578_ +76.4°; [α]_546_ +90.2° (*c* 0.5, chloroform); IR (KBr) $\bar{\nu }$_max_/cm^−1^ 1752 (C=O), 1650 (C=N), 1256, 1218 (C-O-C, ester), 1086, 1033 (C-O), 818 (arom); ^1^H NMR (400 MHz, CDCl_3_) δ 8.97 (1H, s, N=CH), 8.90 (1H, d, *J* 8.8 Hz, H-arom), 7.91 (1H, d, *J* 9.2 Hz, H-arom), 7.49 (1H, t, *J* 8.6 Hz, H-arom), 7.36 (1H, t, *J* 7.0 Hz, H-arom), 7.25 (1H, d, *J* 9.6 Hz, H-arom), 6.10 (1H, d, *J*_1,2_ 7.6 Hz, H-1), 5.58 (1H, t, *J*_3,4_ = *J*_4,5_ 9.4 Hz, H-4), 5.21 (1H, t, *J*_2,3_ = *J*_3,4_ 9.8 Hz, H-3), 4.41 (1H, dd, *J*_5,6_ 4.8 Hz, *J*_6,6′_ 12.4 Hz, H-6), 4.17 (1H, dd, *J*_5,6′_ 2.4 Hz, *J*_6,6′_ 12.4 Hz, H-6′), 4.05 (1H, ddd, *J*_4,5_ 10.0 Hz, *J*_5,6′_ 2.4 Hz, H-5), 3.98 (3H, s, OCH_3_), 3.61 (1H, dd, *J*_1,2_ 8.6 Hz, *J*_2,3_ 9.4 Hz, H-2), 2.12, 2.08, 2.06, 1.97 (4 × 3H, s, CH_3_); ^13^C{^1^H} NMR (100 MHz, CDCl_3_) δ 170.6, 169.9, 169.7, 168.8 (C=O), 163.5 (N=CH), 158.2, 133.3, 131.7, 128.9, 128.2, 128.1, 125.0, 124.1, 116.4, 112.2 (C-arom), 93.2 (C-1), 74.0 (C-2), 73.2 (C-5), 72.8 (C-3), 68.1 (C-4), 61.8 (C-6), 56.3 (OMe), 20.7, 20.4 (4 CH_3_). Anal. Calcd. for C_26_H_29_NO_10_: C, 60.58, H, 5.67, N, 2.72. Found: C, 60.83; H, 5.78; N, 2.67.

*1,3,4,6-Tetra-*O*-acetyl-2-[(*E,E*)-cinnamylideneamino]-2-deoxy-α-d-glucopyranose* (**88**). To a suspension of 1,3,4,6-tetra-O-acetyl-2-amino-2-deoxy-α-d-glucopyranose hydrobromide (**86**) (1.4 g, 3.0 mmol) in 96% aqueous ethanol (14 mL), sodium acetate trihydrate (0.41 g, 3.0 mmol) dissolved in water (2 mL) and pyridine (0.8 mL), and cinnamylidene aldehyde (0.6 mL) were added. The solution was heated in a water bath and filtered to remove impurities, then cooled. The crystalline solid crystallized was collected by filtration and washed with 50% aqueous ethanol to give the title compound in 57% yield. M.p.: 177–179 °C; [α]_D_ +81.8°; [α]_578_ +87.2°; [α]_546_ +93.0° (*c* 0.5, chloroform); IR (KBr) $\bar{\nu }$_max_/cm^−1^ 1747 (C=O), 1635 (C=N), 1251, 1221 (C-O-C, ester), 1027 (C-O); ^1^H NMR (400 MHz, CDCl_3_) δ 8.07 (1H, d, *J*_C*H*=N_ 8.4 Hz, CH-C*H*=N), 7.48 (2H, d, *J* 7.2 Hz, H-arom), 7.38 (3H, m, H-arom), 7.02 (1H, d, *J*_CH=C*H*-Ar_ 16.0 Hz, =CH-Ar), 6.88 (1H, dd, *J*_C*H*-*CH*=CH_ 8.8 Hz, *J*_CH-*CH*=C*H*_ 16.0 Hz, N=CH-C*H*), 6.18 (1H, d, *J*_1,2_ 3.6 Hz, H-1), 5.61 (1H, t, *J*_2,3_ = *J*_3,4_ 9.8 Hz, H-3), 5.17 (1H, t, *J*_3,4_ = *J*_4,5_ 9.8, H-4), 4.36 (1H, dd, *J*_6,6′_ 12.2 Hz, *J*_5,6_ 4.2 Hz, H-6), 4.24 (1H, d, *J*_4,5_ 10.0 Hz, H-5), 4.10 (1H, d, *J*_6,6′_ 12.0 Hz), 3.60 (1H, dd, *J*_1,2_ 3.0 Hz, *J*_2,3_ 10.2 Hz, H-2), 2.22, 2.10, 2.05, 1.95 (4 × 3H, s, CH_3_); ^13^C{^1^H} NMR (100 MHz, CDCl_3_) δ 170.5, 169.6, 168.9, 167.1 (C=O), 167.1 (C=N), 144.0 (C-arom), 135.0, 129.6, 128.7, 127.4, 127.2 (C-arom), 91.4 (C-1), 71.0 (C-2), 70.9 (C-5), 69.8 (C-3), 68.1 (C-4), 61.7 (C-6), 21.0, 20.6, 20.5, 20.4 (CH_3_). Anal. Calcd. for C_23_H_27_NO_9_: C, 59.86, H, 5.90, N, 3.04. Found: C, 59.75, H, 5.83, N, 3.06.

*2-Acetamido-1,3,4,6,7-penta-*O*-acetyl-2-deoxy-α-d-*glycero*-l-*gluco*-heptopyranose* (**90**). To a suspension of 1,3,4,6,7-penta-*O*-acetyl-2-amino-2-deoxy-α-d-*glycero*-l-*gluco*-heptopyranose hydrobromide (**89**) [17] (0.51 g, 0.75 mmol) in pyridine (3.0 mL), acetic anhydride (2.9 mL) was added. The mixture was kept under stirring at room temperature for 30 min until complete dissolution. After 12 h in the refrigerator, it was poured into ice-water and a white solid was filtered, washed with cold water and dried under vacuum over silica gel (0.16 g, 33%). M.p. 90–92 °C (lit. [16] m.p. 82–84 °C); IR (KBr) $\bar{\nu }$_max_/cm^−1^ 3257 (NH), 1751, 1661 (C=O), 1227 (C-O-C), 1047, 1012 (C-O); ^1^H NMR (500 MHz, CDCl_3_) δ 6.15 (1H, d, *J*_1,2_ 3.5 Hz, H-1), 5.67 (1H, d, *J*_2,NH_ 9.0 Hz, NH), 5.20 (1H, t, *J*_2,3_ ≈ *J*_3,4_ 9.5 Hz, H-3), 5.17 (1H, m, *J*_5,6_ 2.0 Hz, *J*_6,7_ 5.0 Hz, *J*_6,7′_ 7.0 Hz, H-6), 5.15 (1H, t, *J*_3,4_ ≈ *J*_4,5_ 10.0 Hz, H-4), 4.47 (1H, td, *J*_1,2_ 4.0 Hz, *J*_2,NH_ 9.5 Hz, *J*_2,3_ 10.5 Hz, H-2), 4.21 (1H, dd, *J*_6,7_ 5.0 Hz, *J*_7,7′_ 11.5 Hz, H-7), 4.10 (1H, dd, *J*_6,7′_ 7.0 Hz, *J*_7,7′_ 11.5 Hz, H-7′), 4.03 (1H, dd, *J*_5,6_ 2.0 Hz, *J*_4,5_ 9.5 Hz, H-5), 2.17, 2.10, 2.02, 2.00, 1.99 (12H, s, OAc), 1.92 (3H, s, NAc); ^13^C{^1^H} NMR (125 MHz, CDCl_3_) δ 171.6, 170.5, 170.3, 169.9, 169.0, 168.4 (C=O), 90.6 (C-1), 70.9 (2C, C-3, C-6), 69.9 (C-5), 66.6 (C-4), 62.1 (C-7), 51.0 (C-2), 23.0, 20.8, 20.6, 20.6, 20.4 (CH_3_). HRMS [M+H]^+^ calculated for C_19_H_28_NO_12_: 462.1606. Found: 462.1618.

*2-Acetamido-1,3,4,6,7-penta-*O*-acetyl-2-deoxy-β-d-*glycero*-l-*gluco*-heptopyranose* (**92**). The title compound was obtained from 1,3,4,6,7-penta-*O*-acetyl-2-amino-2-deoxy-β-d-*glycero*-l-*gluco*-heptopyranose hydrochloride (**91**) [17] (0.26 g, 0.4 mmol) with pyridine (1.5 mL) and acetic anhydride (1.0 mL) using the procedure described for **74** (0.07 g, 27%). M.p. 226–228 °C (Lit. [16] m.p. 221–223 °C); IR (KBr) $\bar{\nu }$_max_/cm^−1^ 3354 (NH), 1754, 1667 (C=O), 1220 (C-O-C), 1073, 1034 (C-O); ^1^H NMR (500 MHz, CDCl_3_) δ 5.76 (1H, d, *J*_2,NH_ 9.0 Hz, NH), 5.66 (1H, d, *J*_1,2_ 9.0 Hz, H-1), 5.24 (1H, ddd, *J*_5,6_ 2.0 Hz, *J*_6,7_ 5.0 Hz, *J*_6,7′_ 7.5 Hz, H-6), 5.16 (1H, t, *J*_2,3_ ≈ *J*_3,4_ 9.5 Hz, H-3), 5.11 (1H, t, *J*_3,4_ ≈ *J*_4,5_ 10.0 Hz, H-4), 4.30 (1H, dd, *J*_6,7_ 5.0 Hz, *J*_7,7′_ 11.5 Hz, H-7), 4.24 (1H, c, *J*_1,2_ = *J*_2,NH_ ≈ *J*_2,3_ 9.5 Hz, H-2), 4.10 (1H, dd, *J*_6,7′_ 8.0 Hz, *J*_7,7′_ 11.5 Hz, 1H, H-7′), 3.85 (1H, dd, *J*_5,6_ 2.0 Hz, *J*_4,5_ 9.5 Hz, H-5), 2.10, 2.09, 2.02, 2.01 (12H, s, OAc), 1.92 (3H, s, NAc); ^13^C{^1^H} NMR (125 MHz, CDCl_3_) δ 171.1, 170.5, 170.3, 170.1, 169.3, 169.2 (C=O), 92.8 (1C, *^1^J* 120 Hz, C-1), 73.0, 72.6 (C-3, C-5), 66.9, 66.5 (C-4, C-6), 62.2 (C-7), 53.1 (C-2), 23.1, 20.8, 20.6, 20.6, 20.5 (CH_3_). HRMS [M+H]^+^ calculated for C_19_H_28_NO_12_: 462.1606; found: 462.1613; [M+Na]^+^ calculated for C_19_H_27_NO_12_Na: 484.1425; found: 484.1435.; [M+K]^+^ calculated for C_19_H_27_NO_12_K: 500.1165, found: 500.1174.

#### 4.4.3. Mutarotational Equilibrium in Schiff Bases Derived from 2-Amino-2-deoxyaldoses

Imine samples (~15 mg) were dissolved in DMSO-*d*_6_ (0.5 mL) or pyridine-*d*_5_ (0.5 mL), and the corresponding ^1^H NMR spectra were immediately recorded, followed by temporal monitoring until equilibration (as inferred from unaltered ^1^H and ^13^C NMR spectra over time).

*2-Deoxy-2-[(*E*)-(3-bromobenzylidene)amino]-α-d-glucopyranose* (**95**). ^13^C{^1^H} NMR (100 MHz, DMSO-*d*_6_) δ 161.0 (C=N), 138.8, 137.4, 132.3, 127.8 (C-arom), 93.1 (C-1), 75.6 (C-2), 72.7 (C-5), 70.9 (C-3, C-4), 61.5 (C-6).

*2-[(*E,E*)-Cinnamylideneamino]-2-deoxy-α-d-glucopyranose* (**105**). ^1^H NMR (400 MHz, DMSO-*d*_6_) δ 8.06 (1H, d, *J*_=C*H*-CH_ 8.7 Hz, N=C*H*-CH), 7.59 (2H, d, *J* 7.3 Hz H-arom), 7.36 (3H, m, H-arom), 7.11 (1H, d, *J*_CH=C*H*_ 16.1 Hz, CH=C*H*-Ar), 6.90 (1H, dd, *J*_C*H*=CH_ 16.1 Hz, *J*_C*H*-CH_ 8.8 Hz, CH-C*H*=CH), 6.24 (1H, d, *J*_C1,OH_ 4.2 Hz, C1-OH), 4.91 (2H, m, *J*_1,2_ ≈ *J*_C1,OH_ 4.0 Hz H-1, C3-OH), 4.71 (1H, d, *J*_C4-OH_ 5.3 Hz, C4-OH), 4.57 (1H, t, *J*_C6-OH_ 5.3 Hz, C6-OH), 3.78 (1H, m, H-3), 3.65 (1H, m, H-6), 3.54 (1H, m, H-6′), 3.00 (1H, t, *J*_1,2_ 3.1 Hz, *J*_2,3_ 9.7 Hz, H-2); ^13^C{^1^H} NMR (100 MHz, DMSO-*d*_6_) δ 164.1 (C=N), 141.6 (2C, CH=CH), 136.0, 129.1, 128.6, 127.4 (2 C-arom), 93.2 (C-1), 75.5 (C-2), 72.6 (C-5), 71.1 (C-3, C-4), 61.5 (C-6).

*2-Deoxy-2-[(*E,E*)-(4-nitrocinnamylidene)amino]-α-d-glucopyranose* (**106**). ^1^H NMR (400 MHz, DMSO-*d*_6_) δ 8.21 (2H, d, *J* 8.7 Hz, H-arom), 8.10 (1H, d, *J*_=CH-CH_ 8.5 Hz, N=C*H*-CH), 7.87 (2H, d, *J* 8.7 Hz, H-arom), 7.25 (1H, d, *J* 16.1 Hz, CH=C*H*-Ar), 7.11 (1H, dd, *J* 8.7 Hz, *J*_C*H*=CH_ 16.1 Hz, CH-C*H*=CH), 6.28 (1H, d, *J*_C1-OH_ 4.4 Hz, C1-OH), 4.93 (1H, d, *J*_1–2_ 5.2 Hz, H-1), 4.74 (1H, m, *J*_C3-OH_ 5.7 Hz, C3-OH), 4.47 (1H, t, *J*_C6-OH_ 5.8 Hz, C6-OH), 4.13 (1H, c, *J*_4,5_ ≈ *J*_5,6_ 5.2 Hz, H-5), 3.78 (1H, m, *J*_3,4_ 9.4 Hz, *J*_2,3_ 5.3 Hz, H-3), 3.66 (1H, ddd, *J*_6,6′_ 11.6 Hz, *J*_5,6_ 2.2 Hz, *J*_C6-OH_ 5.4 Hz, H-6), 3.48 (1H, d, *J*_C6-OH_ 5.9 Hz, H-6′), 3.14 (1H, m, H-4), 3.03 (1H, dd, *J*_1,2_ 3.5 Hz, *J*_2,3_ 9.4 Hz, H-2); ^13^C{^1^H} NMR (100 MHz, DMSO-*d*_6_) δ 163.5 (C=N), 147.4, 142.6 (C-arom), 139.1 (CH=CH), 132.7, 128.4 (C-arom), 124.2 (CH=CH), 93.0 (C-1), 75.6 (C-2), 71.0 (C-3, C-5), 70.4 (C-4), 61.4 (C-6).

*2-Desoxi-2-[(*E,E*)-(2-methoxycinnamylidene)amino]-α-d-glucopyranose* (**107**). ^1^H NMR (400 MHz, DMSO-*d*_6_) δ 8.04 (1H, d, *J*_=C*H*-CH_ 8.8 Hz, N=C*H*-CH), 7.60 (1H, dd, *J* 7.7 Hz, *J* 1.2 Hz, H-arom), 7.33 (1H, dt, *J* 7.7 Hz, *J* 1.4 Hz, H-arom), 7.26 (1H, d, *J*_CH=C*H*_ 16.2 Hz, CH=C*H*-Ar), 6.97 (1H, t, *J* 7.5 Hz, H-arom), 6.90 (1H, dd, *J*_C*H*=CH_ 16.2 Hz, *J*_C*H*-CH_ 8.9 Hz, CH-C*H*=CH), 6.22 (1H, d, *J*_C1,OH_ 4.5 Hz, C1-OH), 4.90 (1H, d, *J*_C3-OH_ 5.4 Hz, C3-OH), 4.88 (1H, t, *J*_1,2_ 4.0 Hz, H-1), 4.68 (1H, d, *J*_C4-OH_ 5.7 Hz, C4-OH), 4.46 (1H, t, *J*_C6-OH_ 5.9 Hz, C6-OH), 3.85 (3H, s, OCH_3_), 3.76 (1H, m, H-3), 3.66 (1H, t, H-4), 3.63 (1H, dd, H-6), 3.52 (1H, dd, *J*_5,6_ 6.2 Hz, *J*_6,6′_ 12.3 Hz, H-6′), 2.97 (1H, dd, *J*_1,2_ 3.1 Hz, *J*_2,3_ 9.7 Hz, H-2); ^13^C{^1^H} NMR (100 MHz, DMSO-*d*_6_) δ 164.7 (C=N), 136.4 (C-arom), 136.1 (CH=CH), 130.6, 129.2, 127.7, 124.3 (C-arom), 120.9 (CH=CH), 93.2 (C-1), 75.5 (C-2), 72.6 (C-5), 71.1 (C-3), 71.0 (C-4), 61.5 (C-6), 55.8 (OCH_3_).

*2-Desoxi-2-[(*E,E*)-(4-hydroxy-3-methoxycinnamylidene)amino]-α-d-glucopyranose* (**108**). ^13^C NMR (50.3 MHz, DMSO-*d*_6_) δ 164.2 (C=N), 148.3 (C-arom), 148.2 (C-arom), 141.8 (CH=CH), 127.7, 126.0, 121.6, 115.9 (C-arom), 111.8 (CH=CH), 93.3 (C-1), 75.4 (C-2), 72.7 (C-5), 71.2 (C-3), 71.1 (C-4), 61.5 (C-6), 55.9 (OCH_3_).

*2-Deoxy-2-[(*E,E*)-(4-nitrocinnamylidene)amino]-β-d-glucopyranose* (**46**). ^1^H NMR (400 MHz, pyridine-*d*_5_) δ 9.06 (1H, d, *J* 5.8 Hz, H-arom), 8.47 (1H, d, *J* 8.7 Hz, H-arom), 8.16 (1H, d, *J*_=C*H*-CH_ 8.7 Hz, N=C*H*-CH), 7.32 (1H, dd, *J* 8.7 Hz, *J*_C*H*=CH_ 16.1 Hz, CH-C*H*=CH), 6.93 (1H, d, *J* 16.1 Hz, CH=C*H*-Ar), 6.71 (1H, t, C6-OH), 5.77 (1H, ddt, *J*_1–2_ ≈ *J*_C1-OH_ 7.3 Hz, H-1), 4.67 (1H, d, *J*_6–6′_ 11.8 Hz, *J*_5–6_ 2.3 Hz, H-6), 4.57 (1H, t, *J*_2–3_ ≈ *J*_3–4_ 8.8 Hz, H-3), 4.50 (1H, dd, *J*_5–6_ 5.4 Hz, *J*_6–6′_ 11.1 Hz, H-6′), 4.41 (1H, t, *J*_2–3_ ≈ *J*_3–4_ 9.0 Hz, H-4), 4.20 (1H, ddd, *J*_4–5_ 8.2 Hz, *J*_5–6_ 5.6 Hz, *J*_5–6′_ 2.5 Hz, H-5), 3.79 (1H, t, *J*_1,2_ ≈ *J*_2,3_ 8.4 Hz, H-2).

*2-Deoxy-2-[(*E*)-(4-methoxy-1-naphthylmethylene)amino]-β-d-glucopyranose* (**51**). ^1^H NMR (400 MHz, pyridine-*d*_5_) δ 9.69 (1H, dd, *J* 2.4 Hz, *J* 6.7 Hz, H-arom), 9.24 (1H, s, CH=N), 8.46 (1H, m, H-arom), 7.93 (1H, d, *J* 8.1 Hz, H-arom), 7.57 (2H, m, H-arom), 7.28 (1H, sa, OH), 6.85 (1H, d, *J* 8.1 Hz, H-arom), 5.85 (1H, d, *J*_1,2_ 7.4 Hz, H-1), 4.72 (1H, dd, *J*_6,6′_ 11.6 Hz, *J*_5,6_ 2.1 Hz, H-6), 4.66 (1H, t, *J*_4,5_ ≈ *J*_3,4_ 8.2 Hz, H-4), 4.53 (1H, dd, *J*_6,6′_ 11.6 Hz, *J*_5,6′_ 5.6 Hz, H-6′), 4.46 (1H, t, *J*_3,4_ ≈ *J*_2,3_ 9.2 Hz, H-3) 4.26 (1H, ddd, *J*_4,5_ 8.9 Hz, *J*_5,6_ 2.5 Hz *J*_5,6′_ 6.0 Hz, H-5), 3.92 (1H, t, *J*_1,2_ ≈ *J*_2,3_ 8.4 Hz, H-2), 3.83 (3H, s, OCH_3_); ^13^C{^1^H} NMR (100 MHz, DMSO-*d*_6_) δ 163.8 (N=C), 157.9 (C-arom), 133.3 (C-arom), 132.6 (C-arom), 128.2 (C-arom), 126.4 (C-arom), 126.1 (C-arom), 122.9 (C-arom), 124.3 (C-arom), 104.3 (C-arom), 98.1 (C-1), 81.9 (C-2), 79.1 (C-5), 77.1 (C-3), 72.4 (C-4), 63.5 (C-6), 56.0 (OCH_3_).

*2-Deoxy-2-[(*E*)-(4-methoxy-1-naphthylmethylene)amino]-α-d-glucopyranose* (**52**). ^1^H NMR (400 MHz, pyridine-*d*_5_) δ 9.83 (1H, m, H-arom), 9.22 (1H, s, CH=N), 8.46 (2H, m, H-arom), 7.98 (1H, d, *J* 8.1 Hz, H-arom), 7.54 (3H, m, H-arom), 7.21 (3H, m, OH), 6.87 (1H, d, *J* 8.08 Hz, H-arom), 6.40 (1H, t, C6-OH), 5.90 (1H, t, *J*_1,2_ ≈ *J*_C1-OH_ 2.8 Hz, H-1), 5.21 (1H, t, *J*_2,3_ ≈ *J*_3,4_ 9.1 Hz, H-3), 5.05 (1H, ddd, *J*_4,5_ 7.9 Hz, *J*_5,6_ 5.4 Hz *J*_5,6_ 2.7 Hz, H-5), 4.68 (1H, dd, *J*_6,6′_ 12.9 Hz, *J*_5,6′_ 1.4 Hz, H-6′), 4.56 (1H, dd, *J*_6,6′_ 11.3 Hz, *J*_5,6_ 4.9 Hz, H-6), 4.48 (1H, t, *J*_3,4_ ≈ *J*_4,5_ 9.3 Hz, H-4), 3.96 (1H, dd, *J*_1,2_ 3.4 Hz, *J*_2,3_ 9.6 Hz, H-2), 3.83 (3H, s, OCH_3_); ^13^C{^1^H} NMR (100 MHz, DMSO-*d*_6_) δ 163.8 (N=C), 157.9 (C-arom), 133.3 (C-arom), 132.6 (C-arom), 128.2 (C-arom), 126.4 (C-arom), 126.1 (C-arom), 122.9 (C-arom), 124.3 (C-arom), 104.3 (C-arom), 95.6 (C-1), 79.0 (C-2), 74.6 (C-5), 73.4 (C-3), 73.2 (C-4), 63.8 (C-6), 56.0 (OCH_3_).

*2-Deoxy-2-[(*E*)-(2-naphthylmethylene)amino]-β-d-glucopyranose* (**53**). ^1^H NMR (400 MHz, pyridine-*d*_5_) δ 9.08 (1H, d, *J* 5.0 Hz, H-arom), 8.90 (1H, s, CH=N), 8.32 (1H, dd, *J* 9.7 Hz, *J* 1.0 Hz, H-arom), 8.12 (1H, s, H-arom), 7.89 (2H, s, H-arom), 7.50 (2H, m, H-arom), 7.34 (1H, sa, OH), 7.23 (2H, sa, OH), 6.78 (1H, sa, C6-OH), 5.86 (1H, t, *J*_1,2_ 7.5 Hz, H-1), 4.70 (1H, d, *J*_6,6′_ 11.8 Hz, H-6), 4.64 (1H, t, *J*_3,4_ = *J*_4,5_ 9.0 Hz, H-4), 4.50 (1H, dd, *J*_6,OH_ 5.5 Hz, *J*_6,6′_ 11.6 Hz, H-6), 4.45 (1H, t, *J*_2,3_ = *J*_3,4_ 9.1 Hz, H-3), 4.25 (1H, m, *J*_4,5_ 8.8 Hz, *J*_5,6_ 2.0 Hz, H-5), 3.96 (1H, t, *J*_1,2_ ≈ *J*_2,3_ 8.4 Hz, H-2).

*2-Deoxy-2-[(*E,E*)-(4-nitrocinnamylidene)amino]-α-d-glucopyranose* (**106**). ^1^H NMR (400 MHz, pyridine-*d*_5_) δ 8.50 (2H, d, *J* 8.7 Hz, H-arom), 8.46 (2H, d, *J* 8.7 Hz, H-arom), 7.59 (1H, d, *J*_=C*H*-CH_ 8.7 Hz, N=C*H*-CH), 7.32 (1H, dd, *J* 8.7 Hz, *J*_C*H*=CH_ 16.0 Hz, CH-C*H*=CH), 7.00 (1H, d, *J* 16.1 Hz, CH=C*H*-Ar), 6.41 (1H, t, *J*_C6-OH_ 5.7 Hz, C6-OH), 5.83 (1H, t, *J*_1–2_ 3.2 Hz, H-1), 4.67 (1H, d, *J*_6–6′_ 11.8 Hz, *J*_5–6_ 2.3 Hz, H-6), 4.57 (1H, t, *J*_2–3_ ≈ *J*_3–4_ 8.8 Hz, H-3), 4.50 (1H, dd, *J*_5–6_ 5.4 Hz, *J*_6–6′_ 11.1 Hz, H-6′), 4.41 (1H, t, *J*_2–3_ ≈ *J*_3–4_ 9.0 Hz, H-4), 4.20 (1H, ddd, *J*_4–5_ 8.2 Hz, *J*_5–6_ 5.6 Hz, *J*_5–6′_ 2.5 Hz, H-5), 3.85 (1H,dd, *J*_1,2_ 3.3 Hz, *J*_2,3_ 9.6 Hz, H-2).

*2-Deoxy-2-[(*E*)-(2-naphthylmethylene)amino]-α-d-glucopyranose* (**54**). ^1^H NMR (400 MHz, pyridine-*d*_5_) δ 8.87 (1H, s, CH=N), 8.51 (1H, d, *J* 3.2 Hz, H-arom), 8.34 (1H, d, *J* 9.6 Hz, H-arom), 8.19 (1H, s, H-arom), 7.88 (2H, m, H-arom), 7.50 (2H, m, arom), 7.26 (2H, sa, OH), 6.48 (1H, t, C6-OH), 5.88 (1H, t, H-1), 5.16 (1H, m, *J*_3,4_ ≈ *J*_2,3_ 9.9 Hz, H-3), 5.05 (1H, dddd, *J*_5,6_ 2.3 Hz, *J*_5,6′_ 5.4 Hz, *J*_4,5_ 7.5 Hz, H-5), 4.66 (1H,d, *J*_6,6′_ 10.3 Hz, H-6), 4.56 (1H, dd, *J*_6,6′_ 11.3 Hz, *J*_5,6_ 4.5 Hz, H-6′), 4.47 (1H, t, *J*_3,4_ ≈ *J*_4,5_ 9.1 Hz, H-4), 4.00 (1H, t, *J*_2,3_ 9.6 Hz, *J*_1,2_ 3.2 Hz, H-2).

## Figures and Tables

**Table 1:** Temporal percentage variation in β-anomers ^a^.

Solvent	Compound	Initial ^b^	2 d	10 d	15 d	Equilibrium
DMSO-*d*_6_	**52**	7.3	17.2	50.1	65.1	83.6
	**54**	13.8	20.3	34.0	44.8	81.4
	**57**	100.0	100	94.7	92.1	84.4
Pyridine-*d*_5_	**51**	100.0	87.8	84.2	---	84.2
	**52**	15.7	73.4	85.3	81.4	88.5
	**54**	19.0	69.8	78.2	85.5	84.1

^a^ At room temperature. ^b^ Just dissolved.

**Table 2:** Relative energy minima found for **56** and **112** ^a^.

		Gas Phase ^b^			DMSO ^b^			Gas Phase ^c^			DMSO ^c^		
		Δ*E*	Δ*G*	θ_N=C-C1-C2_	Δ*E*	Δ*G*	θ_N=C-C1-C2_	Δ*E*	Δ*G*	θ_N=C-C1-C2_	Δ*E*	Δ*G*	θ_N=C-C1-C2_
**56**	Min1	0.00	0.00	50.6	0.00	0.00	56.3	0.00	0.00	52.3	0.00	0.00	52.6
	Min2	0.50	0.90	126.0	1.18	0.57	127.5	0.72	1.17	122.1	1.12	0.80	123.3
**112**	Min1	0.00	0.00	43.9	0.00	0.00	45.7	0.00	0.00	46.0	0.00	0.00	49.2
	Min2	0.62	0.87	141.8	0.35	-0.17	137.9	0.57	0.60	140.4	0.43	0.02	136.4

^a^ In kcal/mol. ^b^ M06-2X/6-311G(d,p). ^c^ M06-2X/def2-TZVP.

**Table 3:** Relative energies (kcal/mol) for species involved in mutarotational equilibria of imines **30** and **93** ^a^.

		30	93	115	116	117e ^b^	117a ^c^	118e ^b^	118a ^c^	119e ^b^	119a ^c^	120e ^b^	120a ^c^
Gas phase	Δ*E*	0.00	1.22	13.56	12.41	8.51	7.51	10.08	7.24	−2.16	3.66	−2.17	2.45
	Δ*G*	0.00	0.21	9.81	7.96	11.28	9.67	11.79	9.28	0.34	5.32	0.96	4.63
DMSO	Δ*E*	0.00	0.99	13.00	11.87	8.00	6.22	7.85	6.44	−1.52	1.38	−1.41	2.48
	Δ*G*	0.00	0.36	10.43	8.92	10.75	8.92	10.42	8.67	0.89	4.09	1.95	4.64

^a^ M06-2X/6-311G(d,p). ^b^ Equatorial NH. ^c^ Axial NH.

**Table 4:** Anomeric stabilization (kcal/mol) of imines from **2** and substituted benzaldehydes ^a^.

	Compound						
	30	31	32	33	34	35	36
α ^b^	11.2	18.9	10.7	11.9	12.3	12.4	12.1
β ^b^	88.8	81.1	89.3	88.1	87.7	87.6	87.9
Δ*G*°_an_	−1.24	−0.87	−1.27	−1.20	−1.18	−1.17	−1.19
*E*_an_ ^c^	0.01	0.38	−0.02	0.05	0.07	0.08	0.06
*E*_an_^THP^	0.69	1.06	0.66	0.73	0.75	0.76	0.74
Δ*G*°_rae_	1.31	0.94	1.34	1.27	1.25	1.24	1.26

^a^ In DMSO-*d*_6_. ^b^ In %. ^c^ Anomeric stabilization referred to cyclohexanol.

**Table 5:** Anomeric stabilization (kcal/mol) of imines from **6** and substituted benzaldehydes ^a^.

	Compound				
	37	38	39	40	41
α ^b^	13.1	12.3	10.8	13.1	10.3
β ^b^	86.9	87.7	89.2	86.9	89.7
Δ*G*°_an_	−1.13	−1.18	−1.26	−1.13	−1.3
*E*_an_ ^c^	0.11	0.07	−0.02	0.11	−0.1
*E*_an_^THP^	0.79	0.75	0.66	0.79	0.6
Δ*G*°_rae_	1.21	1.25	1.30	1.21	1.4

^a^ In DMSO-*d*_6_. ^b^ In %. ^c^ Anomeric stabilization referred to cyclohexanol.

**Table 6:** Anomeric stabilization (kcal/mol) of imines derived from cinnamylidene aldehydes ^a^.

	Compound					
	45	46	46 ^c^	47	48	49
α ^b^	11.4	15.2	13.1	7.5	9.8	12.7
β ^b^	88.6	84.8	86.9	92.5	91.2	87.3
Δ*G*°_an_	−1.23	−1.03	−1.14	−1.51	−1.34	−1.16
*E*_an_ ^d^	0.02	0.22	0.11	−0.26	−0.09	0.09
Δ*G*°_rae_	1.30	1.10	1.21	1.58	1.41	1.23

^a^ In DMSO-*d*_6_. ^b^ In %. ^c^ In pyridine. ^d^ Anomeric stabilization referred to cyclohexanol.

**Table 7:** Anomeric stabilization (kcal/mol) of imines derived from polynuclear aromatic aldehydes ^a^.

	Compound								
	50	51	51 ^c^	53	53 ^c^	55	57	57 ^c^	58
α ^b^	28.6	17.6	16.4	11.7	15.2	10.3	15.6	11.4	10.7
β ^b^	71.4	82.4	83.6	88.3	84.8	89.7	84.4	88.6	89.3
Δ*G*°_an_	−0.55	−0.93	−0.98	−1.21	−1.03	−1.30	−1.01	−1.23	−1.27
*E*_an_ ^d^	0.70	0.32	0.27	0.04	0.22	−0.05	0.24	0.02	−0.02
Δ*G*°_rae_	0.62	1.00	1.05	1.28	1.10	1.37	1.08	1.30	1.34

^a^ In DMSO-*d*_6_. ^b^ In %. ^c^ In pyridine. ^d^ Anomeric stabilization referred to cyclohexanol.

**Table 8:** Calculated stability of imine anomers derived from **2** ^a,b^.

		Gas Phase		DMSO		
Compound	Anomer	Δ*E*	Δ*G*	Δ*E*	Δ*G*	[β] ^c^
**30**	α	0.00	0.00	0.00	0.00	63.0
**93**	β	0.58	−0.30	0.16	−0.32	
**105**	α	0.00	0.00	0.00	0.00	82.0
**45**	β	0.89	0.15	0.33	−0.91	
**110**	α	0.00	0.00	0.00	0.00	76.9
**50**	β	0.59	−0.29	0.15	−0.72	
**110b**	α	−0.34	0.12	−0.04	0.31	73.4
**50b**	β	0.07	−0.36	0.22	−0.30	
**54**	α	0.00	0.00	0.00	0.00	73.1
**53**	β	0.58	−0.01	0.14	−0.36	
**54b**	α	0.98	0.79	0.95	1.65	76.3
**53b**	β	1.63	0.81	1.13	0.95	
**111**	α	0.33	−0.50	−0.01	0.66	85.0
**55**	β	1.12	−0.04	0.23	−0.38	
**111b**	α	0.00	0.00	0.00	0.00	65.3 ^d^
**55b**	β	0.37	−0.11	0.20	0.66	
**112**	α	0.00	0.00	0.00	0.00	63.4
**56**	β	0.57	−0.28	0.09	−0.33	

^a^ In kcal/mol. ^b^ M06-2X/def2-TZVP. ^c^ In %. ^d^ Percentage of β-anomer (**111b** against **55**).

**Table 9:** Calculated stability of hydrated imine anomers with five molecules of water ^a,b^.

		Gas Phase ^b^		DMSO ^b^			Water ^b^		
		Δ*E*	Δ*G*	Δ*E*	Δ*G*	[β] ^c^	Δ*E*	Δ*G*	[β] ^c^
**30.5H_2_O**	α	−2.41	−0.70	0.11	1.17	87.5	1.85	1.88	95.8
**93.5H_2_O**	β	0.00	0.00	0.00	0.00		0.00	0.00	
**105.5H_2_O**	α	−1.86	−0.23	0.38	2.07	96.9	1.56	3.15	99.5
**45.5H_2_O**	β	0.00	0.00	0.00	0.00		0.00	0.00	
**111.5H_2_O**	α	−2.24	−1.49	−1.03	0.92	82.2	1.11	2.43	98.3
**55.5H_2_O**	β	0.00	0.00	0.00	0.00		0.00	0.00	
**112.5H_2_O**	α	−2.15	0.39	−0.20	3.04	99.4	1.00	0.96	83.2
**56.5H_2_O**	β	0.00	0.00	0.00	0.00		0.00	0.00	

^a^ In kcal/mol. ^b^ M06-2X/def2-TZVP. ^c^ In %.

**Table 10:** Calculated geometrical parameters for intramolecular H-bonds in compounds **30**, **54**, **105**, **110**, **111** and **112** ^a^.

		*D*-H···A	*d*(*D*-H) ^b^	*d*(H···*A*) ^b^	*d*(*D*···*A*) ^b^	<(*D*H*A*) ^c^	*E*_HB_ ^d,e^
**30**	Gas phase	O-H···N	0.9663	2.2058	2.7577	115.06	−6.46
	DMSO	O-H···N	0.9688	2.1618	2.7347	116.47	−7.10
**105**	Gas phase	O-H···N	0.9668	2.2097	2.7595	114.87	−6.42
	DMSO	O-H···N	0.9685	2.1724	2.7431	116.34	−6.86
**110**	Gas phase	O-H···N	0.9666	2.2140	2.7598	114.59	−6.40
	DMSO	O-H···N	0.9689	2.1566	2.7306	116.52	−7.22
**110b**	Gas phase	O-H···N	0.9659	2.2362	2.7699	113.74	−6.14
	DMSO	O-H···N	0.9685	2.1864	2.7499	115.80	−6.67
**54**	Gas phase	O-H···N	0.9664	2.2134	2.7621	114.82	−6.34
	DMSO	O-H···N	0.9689	2.1616	2.7354	166.54	−7.08
**54b**	Gas phase	O-H···N	0.9663	2.2115	2.7626	115.01	−6.33
	DMSO	O-H···N	0.9688	2.1572	2.7329	116.68	−7.16
**111**	Gas phase	O-H···N	0.9659	2.2310	2.7673	113.92	−6.21
	DMSO	O-H···N	0.9687	2.1730	2.7406	116.08	−6.93
**111b**	Gas phase	O-H···N	0.9667	2.1964	2.7554	115.58	−6.52
	DMSO	O-H···N	0.9688	2.1534	2.7298	116.72	−7.25
**112**	Gas phase	O-H···N	0.9668	2.2162	2.7561	114.09	−6.50
	DMSO	O-H···N	0.9691	2.1635	2.7339	116.25	−7.13

^a^ M06-2X/def2-TZVP. ^b^ In Å. ^c^ In degrees. ^d^ In kcal/mol. ^e^ Equation (9). Reference [75].

**Table 11:** Anomeric composition (%) at equilibrium for imines and enamines derived from compounds **2** and **6** ^a^.

Compound							
Anomer	123	125	126	127 ^b^	128 ^b^	133 ^b^	134 ^b^
α	84.7	85.0	70.0	33.7	84.8	75.0	71.0
β	15.3	15.0	30.0	66.3	15.2	25.0	29.0
Δ*G*°	1.0	1.0	0.5	−0.4	1.0	0.7	0.5
*E*_an_	2.3	2.3	1.8	0.8	2.3	1.9	1.8
Δ*G*°_rae_	−1.0	−1.0	−0.4	0.5	−1.0	−0.6	−0.5

^a^ In D_2_O. ^b^ In DMSO-*d*_6_.

**Table 12:** Anomeric composition (%) at equilibrium for aldoses and 2-aminoaldoses ^a^.

Compound										
Anomer	2 ^a^	2 ^b^	6	121 ^a^	121 ^b^	139	140 ^a^	141 ^a^	142	143
α	63.3	87.0	83.0	48.8	52.9	36.3	39.0	90.0	47.6	31.0
β	36.7	13.0	17.0	51.2	47.1	63.7	61.0	10.0	52.4	69.0
Δ*G*°	0.3	1.1	0.9	0.0	0.1	−0.3	−0.3	1.3	−0.1	−0.5
*E*_an_	1.6	2.4	2.2	1.2	1.3	0.9	1.0	2.6	1.2	0.8
Δ*G*°_rae_	−0.3	−1.1	−0.9	0.1	0.0	0.4	0.3	−1.2	0.1	0.6

^a^ In D_2_O. ^b^ In DMSO-*d*_6_.

## Data Availability

All data can be obtained from the authors upon reasonable request. This manuscript is part of two Ph.D. theses, by two of us (E.M. and E.M.S.P.) under a Creative Commons Licence, which are available at https://dehesa.unex.es/handle/10662/5152 and https://dialnet.unirioja.es/servlet/tesis?codigo=565, respectively, from institutional repositories. (Accessed on 1 August 2024).

## Supplementary Materials

The following supporting information can be downloaded at: https://www.mdpi.com/article/10.3390/molecules29174131/s1: IR, NMR spectra, and computational data mentioned through the entire manuscript.
