# Supplementary material for: Exploring and Re-Assessing Reverse Anomeric Effect in 2-Iminoaldoses Derived from Mono- and Polynuclear Aromatic Aldehydes
Source: Molecules. 2024 Aug 30;29(17):4131. doi: 10.3390/molecules29174131 (PMC11397646; doi:10.3390/molecules29174131)
Supplement: Supplementary file 1 [file molecules-29-04131-s001.zip › molecules-3168461-supplementary.pdf]

Supporting Information

Exploring and Re-assessing Reverse Anomeric Effect in 2-Iminoaldoses Derived from Mono- and Polynuclear Aromatic Aldehydes

Esther M. S. Pérez,[1] *Esther Matamoros,*[1,2,3] *Pedro Cintas*[1] *and Juan C. Palacios.*[1]

[1] *Departamento de Química Orgánica e Inorgánica, Facultad de Ciencias, and Instituto del Agua, Cambio Climático y Sostenibilidad (IACYS), Universidad de Extremadura, 06006 Badajoz, Spain*

[2] *Departamento de Química Orgánica, Universidad de Málaga, Campus Teatinos s/n, 29071 Málaga, Spain.*

[3] *Instituto de Investigación Biomédica de Málaga y Plataforma en Nanomedicina – IBIMA, Plataforma Bionand, Parque Tecnológico de Andalucía, 29590 Málaga, Spain*

E-mail: esthermc@unex.es; palacios@unex.es

CONTENTS

Tables S1-S29 2

IR Spectra (Figures S1-S42) 8

NMR Spectra (Figures S43-S207) 29

Cartesian Coordinates and Calculated Energies at the M06-2X/6-311G(d,p) and M06-2X/def2-TZVP Level in Gas Phase, DMSO and water (SMD Model) 112

Table S1. ^{1}H NMR data (δ, ppm) of **30-36**[a]

Comp	CH=N	C1OH	H-1	H-2	H-3	H-4	H-5	H-6	H-6´
**30**	8.33 s	6.22 d d	4.95 t	3.14 dd	3.83 dt	3.20 dt	3.76 ddd	3.68 dd	3.54 dt
**31**	8.34 s	6.23 d	4.95 t	3.16 dd	3.80 dt	3.20 dt	3.76 ddd	3.67 dd	3.54 dt
**32**	8.19 s	6.62 d	4.73 t	2.85 t	3.45 m	3.16 m	3.25 m	3.72 m	3.49 m
**33**	8.21 s	6.59 d	4.73 t	2.86 t	3.45 dt	3.18 dt	3.26 ddd	3.74 ddd	3.50 dt
**34**	8.30 s	6.65 d	4.76 t	2.91 t	3.48 dt	3.18 dt	3.27 ddd	3.74 ddd	3.69 dd
**35**	8.03 s	6.50 d	4.68 t	2.76 t	3.42 dt	3.16 dt	3.24 m	3.73 dd	3.49 dt
**36**	8.06 s	6.69 d	4.69 t	2.77 t	3.42 dt	3.15 dt	3.24 ddd	3.73 m	3.49 dt

[a] In DMSO-d_6 at 500 MHz.

Table S2. Coupling constants (Hz) of **30-36**[a]

Comp	$J_{1,2}$	$J_{2,3}$	$J_{3,4}$	$J_{4,5}$	$J_{5,6}$	$J_{5,6´}$	$J_{6,6´}$	$J_{C1,OH}$
**30**	3.5	9.5	9.5	9.5	2.0	5.5	11.5	4.0
**31**	3.5	9.5	9.5	9.5	2.0	5.5	11.5	4.5
**32**	8.0	9.0	9.0	9.0	2.0	6.0	11.5	7.0
**33**	8.0	9.0	9.0	9.0	2.0	6.0	12.0	6.5
**34**	7.5	9.5	9.0	9.0	1.5	6.0	11.5	6.5
**35**	7.5	9.0	9.0	9.5	----	6.0	12.0	7.2
**36**	8.5	9.0	9.0	9.5	2.0	6.0	12.0	6.5

[a] In DMSO-d_6 at 500 MHz.

Table S3. ^{13}C NMR data (δ, ppm) of **30-36**[a]

Comp	ArCHN	C-1	C-2	C-3	C-4	C-5	C-6
**30**	162.2	93.0	75.1	70.9	70.8	72.5	61.4
**31**	162.2	93.0	75.1	70.9	70.8	72.5	61.4
**32**	160.7	95.5	78.1	74.4	70.3	77.0	61.3
**33**	160.9	95.5	78.2	74.4	70.3	76.9	61.3
**34**	161.0	95.4	78.4	74.3	70.2	77.0	61.3
**35**	161.5	95.8	78.3	74.8	70.4	76.8	61.3
**36**	161.5	95.8	78.3	74.8	70.5	76.9	61.3

[a] In DMSO-d_6 at 125 MHz.

Table S4. ^{1}H NMR data (δ, ppm) of **37-41.**[a]

Comp	CH=	C1OH	H-1	H-2	H-3	H-4	H-5	H-6	H-7	H-7´
**37**	8.11 d	6.46 d	4.66 t	2.78 t	3.55 m	3.55 m	3.27 d	3.79 m	3.55 m	3.55 m
**38**	8.20 d	6.51 bs	4.70 t	2.85 t	3.52 m	3.52 t	3.29 d	3.80 dd	3.52 dd	3.52 dd
**39**	8.36 d	6.60 s	4.75 t	2.93 t	3.47 m	3.47 m	3.31 m	3.80 c	3.47 dt	3.47 dt
**40**	8.16 d	6.48 d	4.68 t	2.81 t	3.45 m	3.45 m	3.28 d	3.79 m	3.45 m	3.45 m
**41**	8.40 d	6.46 d	4.67 t	2.83 t	3.43 m	3.43 m	3.28 d	3.79 c	3.43 m	3.43 m

[a]In DMSO-d_6 at 500 MHz.

Table S5. Coupling constants (Hz) of **37-41**[a]

Comp	$J_{1,2}$	$J_{2,3}$	$J_{3,4}$	$J_{4,5}$	$J_{5,6}$	$J_{1,OH}$
**37**	7.0	7.0	-	8.0	-	6.5
**38**	7.5	9.0	-	8.0	8.0	-
**39**	7.5	8.5	-	8.0	-	-
**40**	8.0	8.0	-	8.5	8.5	6.5
**41**	8.5	8.5	-	8.5	8.5	7.0

[a]In DMSO-d_6 at 500 MHz.

Table S6. ^{13}C NMR data (δ, ppm) of **37-41**[a]

Comp	C-1	C-2	C-3	C-4	C-5	C-6	C-7	CH=N
**37**	96.0	78.3	74.9	69.2	74.5	68.7	62.5	161.1
**38**	96.4	78.8	74.9	69.6	74.9	69.1	63.0	162.4
**39**	96.2	78.9	75.0	69.5	74.9	69.1	62.9	160.9
**40**	94.4	78.8	75.2	69.6	74.9	69.2	63.0	162.2
**41**	96.5	79.1	75.3	69.7	74.9	69.2	63.0	160.8

[a]In DMSO-d_6 at 125 MHz.

Table S7. ^{1}H NMR data (δ, ppm) of **45-49**[a]

Comp	CH=N	C1OH	H-1	H-2	H-3	H-4	H-5	H-6	H-6´
**45**	7.93 d	6.56 d	4.64 d	2.71 t	3.36 m	3.13 dt	3.21 m	3.72 dd	3.47 m
**46**	7.99 d	6.61 d	4.68 t	2.76 t	3.48 m	3.15 m	3.23 m	3.72 dd	3.48 m
**47**	7.92 d	6.55 d	4.63 d	2.70 t	3.36 m	3.15 dt	3.18 m	3.71 ddd	3.47 dt
**48**	7.88 d	6.55 d	4.62 bs	2.69 t	3.36 t	3.15 q	3.20m	3.72d	3.47m
**49**[b]	7.92 d	6.46 d	4.60 t	2.70 t	3.42 m	3.42 m	3.24 dd	3.42 m	---

[a] In DMSO-d_6 at 400 MHz. [b]H-7 and H-7" appear as a multiplet at 3.42 ppm.

Table S8. Coupling constants (Hz) of **45-49**[a]

Comp	$J_{1,2}$	$J_{2,3}$	$J_{3,4}$	$J_{4,5}$	$J_{5,6}$	$J_{5,6'}$	$J_{6,6'}$	$J_{C1,OH}$
**45**	8.5	8.5	8.9	---	3.1	5.8	11.4	6.7
**46**	8.5	8.5	---	---	5.4	---	10.2	6.7
**47**	8.5	8.5	---	---	5.8	1.6	11.5	6.8
**48**	8.4	8.9	8.9	9.1	1.5	---	11.0	4.3
**49**	8.3	8.3	--	9.1	0.8			7.1

[a] In DMSO-d_6 at 100 MHz.

Table S9. ^{13}C NMR data (δ, ppm) of **45-49**[a]

Comp	ArCHN	C-1	C-2	C-3	C-4	C-5	C-6	C-7
**45**	163.9	95.8	78.6	74.8	70.5	77.1	61.5	
**46**	163.5	95.7	78.7	74.7	70.4	77.1	61.4	
**47**	164.4	95.8	78.5	74.8	70.5	77.1	61.5	
**48**	164.2	95.9	78.4	75.0	70.5	77.1	61.5	
**49**	163.8	96.2	78.6	75.1	69.3	74.7	68.9	62.7

[a] In DMSO-d_6 at 100 MHz.

Table S10. ^{1}H NMR data (δ, ppm) of **50-58**[a]

Comp	CH=N	C1OH	H-1	H-2	H-3	H-4	H-5	H-6	H-6´
**50**	8.81 s	6.64 d	4.85 t	2.98 dd	3.53 m	3.22 m	3.31 m	3.77 dd	3.53 m
**51**	8.64 s	6.58 d	4.80 d	2.88 t	3.53 m	3.20 m	3.27 m	3.74 dd	3.53 m
**52**	8.99 s	6.28 d	5.00 t	3.15 dd	3.89 dt	3.22 dt	3.53 dd	3.78 dd	3.69 dd
**53**	8.38 s	6.65 d	4.80 t	2.95 t	3.52 m	3.22dd	3.30m	3.77dd	3.52 m
**54**	8.49 s	6.29 d	5.01 t	3.22 dd	3.87 dt	3.57 m	3.70 dd	3.80 ddd	
**55**	8.26 s	6.68 d	4.87 t	3.01 t	3.56 m	3.25 m	3.32 m	3.78 dd	3.56 m
**56**	9.31 s	---	4.88 d	3.27 t	3.51 m	3.51 m	3.51 m	3.78 d	3.51 m
**57**	9.23 s	6.88 d	4.89 t	3.26 t	3.64 m	3.32 m	3.32 m	3.80 dd	3.56 m
**58**[b]	9.26 s	6.77 d	4.84 t	3.24 t	3.61 m	3.61 m	3.37 m	3.85 c	---

[a] In DMSO-d_6 at 400 MHz. [b]H-7 and H-7" appear as a multiplet at 3.50 ppm.

Table S11. Coupling constants (Hz) of **50-58**[a]

Comp	$J_{1,2}$	$J_{2,3}$	$J_{3,4}$	$J_{4,5}$	$J_{5,6}$	$J_{5,6'}$	$J_{6,6'}$	$J_{6,7}$	$J_{6,7'}$	$J_{C1,OH}$
**50**	8.5	8.5	8.9	---	1.2	0	10.9	---	---	4.3
**51**	7.2	8.5	--	--	1.3	0	10.2	---	---	6.7
**52**	3.3	9.7	9.2	9.2	---	0	11.6	---	---	4.3
**53**	8.5	8.5	8.9	8.9	1.5	0	10.3	---	---	6.7
**54**	3.3	9.9	9.3	9.3	1.8	---	11.6	---	---	4.3
**55**	8.5	8.5	8.9	---	1.4	0	9.9	---	---	6.9
**56**	8.6	8.6	--	--	---	---	11.4	---	---	----
**57**	8.5	8.5	8.3	---	----	---	11.6	---	---	7.2
**58**	8.2	8.2	--	--	6.5	--	--	6.5	6.5	7.3

[a] In DMSO-d_6 at 400 MHz.

Table S12. ^{13}C NMR data (δ, ppm) of **50-58**[a]

Comp	ArCHN	C-1	C-2	C-3	C-4	C-5	C-6	C-7
**50**	162.4	96.0	79.4	74.9	70.6	77.2	61.5	
**51**	162.5	96.0	79.4	75.1	70.6	77.2	61.5	
**52**	162.5	93.4	75.1	71.4	71.2	72.7	61.5	
**53**	162.3	95.8	78.6	74.7	70.5	77.1	61.5	
**54**	162.3	93.1	75.4	71.1	71.0	72.7	61.5	
**55**	162.8	95.9	79.5	74.9	70.6	77.2	61.5	
**56**	161.6	95.8	79.6	74.6	70.8	77.3	61.4	
**57**	162.2	95.9	79.8	74.7	70.9	77.4	61.5	
**58**	161.6	96.2	79.8	75.0	69.7	74.9	69.0	62.8

[a] In DMSO-d_6 at 100 MHz.

Table S13. ^{1}H NMR data (δ, ppm) of **63-68**[a]

Comp	CH=N	H-1	H-2	H-3	H-4	H-5	H-6	H-6´
**63**	8.24 s	5.97 d	3.50 t	5.46 t	5.15 t	3.99 ddd	4.38 dd	4.14 dd
**64**	8.22 s	5.96 d	3.51 t	5.45 t	5.15 t	3.99 ddd	4.39 dd	4.14 dd
**65**	8.18 s	5.96 d	3.50 t	5.44 t	5.15 t	3.99 ddd	4.39 dd	4.14 dd
**66**	8.20 s	5.95 d	3.49 t	5.44 t	5.15 t	3.98 ddd	4.38 dd	4.14 dd
**67**	8.09 s	5.93 d	3.49 t	5.42 t	5.14 t	3.96 ddd	4.38 dd	4.13 dd
**68**	8.12 s	5.94 d	3.43 t	5.42 t	5.14 t	3.97 ddd	4.38 dd	4.13 dd

[a] In $CDCl_3$ at 500 MHz.

Table S14. Coupling constants (Hz) of **63-68**[a]

Comp	$J_{1,2}$	$J_{2,3}$	$J_{3,4}$	$J_{4,5}$	$J_{5,6}$	$J_{5,6'}$	$J_{6,6'}$
**63**	8.0	10.0	10.0	10.0	4.5	2.0	12.5
**64**	8.5	10.0	10.0	10.0	4.5	2.0	12.5
**65**	8.5	9.5	9.5	9.5	4.5	2.0	12.0
**66**	8.5	10.0	10.0	10.0	4.5	2.0	12.5
**67**	8.0	9.5	9.5	9.5	4.5	2.0	12.5
**68**	8.0	10.0	10.0	10.0	4.5	2.0	12.5

[a] In $CDCl_3$ at 500 MHz.

Table S15. ^{13}C NMR data (δ, ppm) of **63-68**[a]

Comp	ArCHN	C-1	C-2	C-3	C-4	C-5	C-6
**63**	165.2	93.1	73.1	73.0	68.0	72.8	61.8
**64**	163.8	93.0	73.0	72.8	68.0	72.8	61.8
**65**	163.6	92.9	73.0	72.9	68.0	72.8	61.8
**66**	163.7	93.0	73.0	72.9	68.0	72.8	61.8
**67**	164.6	93.3	73.4	73.1	68.1	72.7	62.0
**68**	164.4	93.3	73.4	73.0	68.1	72.8	61.9

[a]In $CDCl_3$ at 125 MHz.

Table S16. ^1H NMR data (δ, ppm) of **69-72**[a]

Comp	CH=N	H-1	H-2	H-3	H-4	H-5	H-6	H-7	H-7´
**69**	8.15 s	5.87 d	3.46 t	5.42 t	5.13 t	4.01 dd	5.36 m	4.35 dd	4.17 dd
**70**	8.31 s	5.91 d	3.56 t	5.44 t	5.13 t	3.97 dd	5.35 m	4.33 dd	4.12 dd
**71**	8.20 s	5.88 d	3.48 t	5.43 t	5.13 t	4.02 dd	5.36 m	4.37 dd	4.17 dd
**72**	8.50 s	5.91 d	3.49 t	5.47 t	5.15 t	4.04 dd	5.38 m	4.36 dd	4.33 dd

[a]In $CDCl_3$ at 500 MHz.

Table S17. Coupling constants (Hz) of **69-72**[a]

Comp	$J_{1,2}$	$J_{2,3}$	$J_{3,4}$	$J_{4,5}$	$J_{5,6}$	$J_{6,7}$	$J_{6,7'}$	$J_{7,7'}$
**69**	8.5	10.0	10.0	10.0	2.0	5.0	8.0	11.5
**70**	9.0	9.0	10.0	10.0	2.0	5.3	8.0	12.5
**71**	9.0	9.0	10.0	10.0	2.0	5.5	8.0	12.0
**72**	9.0	9.0	10.0	10.0	2.0	5.3	8.0	11.8

[a] In $CDCl_3$ at 500 MHz.

Table S18. ^{13}C NMR data (δ, ppm) of **69-72**[a]

Comp	ArCHN	C-1	C-2	C-3	C-4	C-5	C-6	C-7
**69**	164.3	93.6	73.4	73.0	67.2	72.9	66.8	62.1
**70**	162.8	93.2	73.0	72.9	67.0	72.9	66.6	62.0
**71**	165.0	93.5	73.2	73.0	67.2	72.9	66.7	62.1

[a] In $CDCl_3$ at 125 MHz.

Table S19. ^1H NMR data (δ, ppm) of **73-75**[a]

Comp	CH=	H-1	H-2	H-3	H-4	H-5	H-6	H-6´
**73**	7.99 d	5.99 d	3.38 t	5.38 t	5.13 t	3.95 ddd	4.37 dd	4.12 dd
**74**	8.03 d	5.91 d	3.43 dd	5.41 t	5.14 t	3.97 ddd	4.38 dd	4.13 dd
**75**[b]	7.98 d	5.64 d	3.39 t	5.36 m	5.13 t	3.98 dd	5.36 m	---

[a]In $CDCl_3$ at 400 MHz. [b]H-7 and H-7' appear as dd at 4.33 and 4.15 ppm, respectively.

Table S20. Coupling constants (Hz) of **73-75**[a]

Comp	$J_{1,2}$	$J_{2,3}$	$J_{3,4}$	$J_{4,5}$	$J_{5,6}$	$J_{5,6'}$	$J_{6,6'}$
**73**	8.1	9.5	9.5	10.1	4.5	2.1	12.4
**74**	8.3	9.7	9.8	9.8	4.5	1.9	12.4
**75**	8.8	9.6	9.7	9.7	2.0	--	--

[a]In $CDCl_3$ at 400 MHz.

Table S21. ^{13}C NMR data (δ, ppm) of **73-75**[a]

Comp	C-1	C-2	C-3	C-4	C-5	C-6	C-7	CH=N
**73**	93.1	73.2	72.7	68.0	73.0	61.8		166.8
**74**	92.9	73.0	67.8	67.8	72.7	61.6		165.7
**75**	93.4	73.0	72.9	67.1	72.9	66.7	62.1	166.8

[a]In $CDCl_3$ at 100 MHz.

Table S22. ^{1}H NMR data (δ, ppm) of **76-84**[a]

Comp	CH=N	H-1	H-2	H-3	H-4	H-5	H-6	H-6´
**76**	8.89 s	6.07 d	3.61 t	5.21 t	5.58 t	4.04 ddd	4.42 dd	4.17 dd
**77**	8.74 s	6.04 d	3.53 dd	5.20 t	5.55 t	4.05 ddd	4.40 dd	4.16 dd
**78**	8.74 s	6.32 d	3.73 ddd	5.23 t	5.70 t	4.29 ddd	4.38 dd	4.14 dd
**79**	8.55 s	6.18 d	3.88 t	5.06 d	5.56 t	4.33 dd	4.27 dd	4.06 dd
**81**	8.09 s	6.10 d	3.63 dd	5.23 t	5.61 t	4.06 ddd	4.42 dd	4.18 dd
**82**	9.46 s	6.14 d	3.88 t	5.27 t	5.71 t	4.10 ddd	4.45 dd	4.20 dd
**83**	9.43 s	6.13 d	3.89 dd	5.28 t	5.71 t	4.07 ddd	4.45 dd	4.19 dd
**84**[b]	9.45 s	6.06 d	3.89 dd	5.25 t	5.71 t	4.12 dd	5.42 ddd	---

[a] In $CDCl_3$ at 400 MHz. [b] H-7 and H-7' appears as dd at 4.39 and 4.12 ppm, respectively.

Table S23. Coupling constants (Hz) of **76-84**[a]

Comp	$J_{1,2}$	$J_{2,3}$	$J_{3,4}$	$J_{4,5}$	$J_{5,6}$	$J_{5,6'}$	$J_{6,6'}$	$J_{6,7}$	$J_{6,7'}$	$J_{7,7'}$
**76**	8.4	9.8	9.8	10.0	4.4	1.8	12.4	---	---	---
**77**	8.5	9.5	9.5	10.2	4.5	1.8	12.3	---	---	---
**78**	3.6	9.8	9.8	9.9	4.1	2.1	12.3	---	---	---
**79**	8.4	9.6	9.6	10.2	4.4	1.8	12.3	---	---	---
**81**	6.2	9.7	9.7	10.1	4.4	2.0	12.4	---	---	---
**82**	8.1	9.6	9.1	9.8	4.4	1.7	12.3	---	---	---
**83**	8.3	9.7	9.7	10.1	4.3	1.9	12.5	---	---	---
**84**	8.3	9.0	9.6	9.6	1.4			5.2	7.9	11.4

[a] In $CDCl_3$ at 400 MHz.

Table S24. ^{13}C NMR data (δ, ppm) of **76-84**[a]

Comp	ArCHN	C-1	C-2	C-3	C-4	C-5	C-6	C-7
**76**	165.1	93.1	73.9	72.8	68.0	73.2	61.8	---
**77**	160.8	93.3	73.4	71.7	68.1	72.8	61.8	---
**78**	158.3	91.9	71.8	70.1	68.3	71.3	61.9	---
**79**	165.0	93.0	73.0	72.7	67.9	73.0	61.7	---
**81**	165.7	93.1	74.0	72.8	68.0	73.2	65.0	---
**82**	165.6	93.0	73.2	72.8	68.3	72.8	61.7	---
**83**	166.3	92.9	74.6	72.8	68.2	73.2	61.8	---
**84**	165.7	93.9	74.5	73.3	67.5	73.0	66.7	62.1

[a] In $CDCl_3$ at 100 MHz.

Table S25. ^{1}H NMR data (δ, ppm) of **90** and **92**[a]

Comp	NH	AcN	H-1	H-2	H-3	H-4	H-5	H-6	H-7	H-7´
**90**	5.67 d	1.92	6.15 d	4.47 ddd	5.20 t	5.15 t	4.03 dd	5.17 ddd	4.21 dd	4.10 dd
**92**	5.75 d	1.92	5.66 d	4.24 c	5.16 t	5.11 t	3.85 dd	5.24 ddd	4.30 dd	4.11 dd

[a] In $CDCl_3$ at 500 MHz.

Table S26. Coupling constants (Hz) of **90** and **92**[a]

Comp	$J_{1,2}$	$J_{2,3}$	$J_{3,4}$	$J_{4,5}$	$J_{5,6}$	$J_{6,7}$	$J_{6,7'}$	$J_{7,7'}$	$J_{2,NH}$
**90**	3.5	9.5	9.5	10.0	2.0	5.0	7.0	11.5	9.0
**92**	9.5	9.5	9.5	9.5	2.0	5.0	8.0	11.5	9.0

[a] In $CDCl_3$ at 500 MHz.

Table S27. ^{13}C NMR data (δ, ppm) of **90** and **92**[a]

Comp	AcN	C-1	C-2	C-3	C-4	C-5	C-6	C-7
**90**	23.0	90.6	51.0	70.9	66.6	69.9	70.9	62.1
**92**	23.1	92.3	53.1	72.6	66.9	73.0	66.5	62.2

[a] In $CDCl_3$ at 125 MHz.

Table S28. NBO stabilizing interactions of some representative α-anomers[a]

Donor	Acceptor	30		105		110		54		111		112	
		Gas phase	DMSO	Gas phase	DMSO	Gas phase	DMSO	Gas phase	DMSO	Gas phase	DMSO	Gas phase	DMSO
LP (1) N20	BD*(1) C2 – H9	6.66	6.36	6.62	6.31	6.79	6.33	6.44	6.03	6.60	5.92	6.74	6.13
LP (1) N20	BD*(1) C32- H33	12.79	11.92	12.79	11.87	12.27	11.45	12.73	11.89	12.21	11.51	12.27	11.52
LP (1) N20	BD*(1) O35- H36	2.11	2.80	2.25	2.91	2.11	2.73	2.25	2.86	2.26	2.87	1.91	2.58
LP (1) O35	BD*(1) C1 –O10	3.11	2.54	3.09	2.51	3.15	2.57	3.06	2.59	2.86	2.53	3.37	2.68
LP (2) O35	BD*(1) C1 - O10	3.39	4.67	3.45	4.72	3.29	4.59	3.52	4.53	4.01	4.73	2.79	4.28
LP (2) O35	BD*(1) C1 – H34	10.40	9.87	10.46	9.87	10.47	9.93	10.39	9.88	10.37	9.86	10.44	9.89
LP (2) O10	BD*(1) C1 - C2	6.51	5.98	6.47	5.95	6.51	5.94	6.48	5.96	6.47	5.95	6.43	5.94
LP (2) O10	BD*(1) C1 – O35	13.47	13.64	13.48	13.64	13.49	13.66	13.51	13.66	13.51	13.66	13.53	13.69
LP (2) O10	BD*(1) C4 - C5	6.01	5.89	6.01	5.88	6.02	5.85	6.01	5.88	6.03	5.94	5.98	5.84
LP (2) O10	BD*(1) C5 – H8	7.37	7.62	7.42	7.65	7.33	7.64	7.39	7.64	7.38	7.57	7.47	7.70

[a] M06-2X/6-311G(d,p).

Table S29. NBO stabilizing interactions of some representative β-anomers[a]

Donor	Acceptor	93		45		50		53		55		56	
		Gas phase	DMSO	Gas phase	DMSO	Gas phase	DMSO	Gas phase	DMSO	Gas phase	DMSO	Gas phase	DMSO
LP (1) N20	BD*(1) C2 – H9	6.53	7.18	6.29	7.22	5.71	7.01	6.93	7.13	5.30	7.07	7.43	6.97
LP (1) N20	BD*(1) C32- H33	12.92	12.44	12.89	12.37	12.19	11.87	12.96	12.40	12.16	11.94	12.77	12.10
LP (2) O35	BD*(1) C1 – H34	7.09	5.71	7.07	5.68	6.85	5.60	7.15	5.68	6.92	5.70	7.23	5.27
LP (2) O35	BD*(1) C1 - O10	15.34	17.13	15.27	17.14	15.44	17.29	15.42	17.16	15.20	17.21	14.93	17.07
LP (1) O10	BD*(1) C1 – O35	5.31	5.01	5.31	4.99	5.32	4.96	5.27	5.01	5.31	4.99	5.19	4.95
LP (2) O10	BD*(1) C1 - C2	7.26	7.04	7.26	7.04	7.24	7.02	7.24	7.02	7.32	6.98	7.14	7.09
LP (2) O10	BD*(1) C1 – H34	6.88	6.35	6.84	6.29	6.94	6.39	6.84	6.29	6.87	6.37	6.89	6.36
LP (2) O10	BD*(1) C4 - C5	7.26	7.36	7.27	7.37	7.22	7.36	7.27	7.36	7.25	7.33	7.32	7.35
LP (2) O10	BD*(1) C5 – H8	6.47	6.31	6.45	6.36	6.52	6.34	6.51	6.39	6.50	6.39	6.70	6.39

[a] M06-2X/6-311G(d,p).

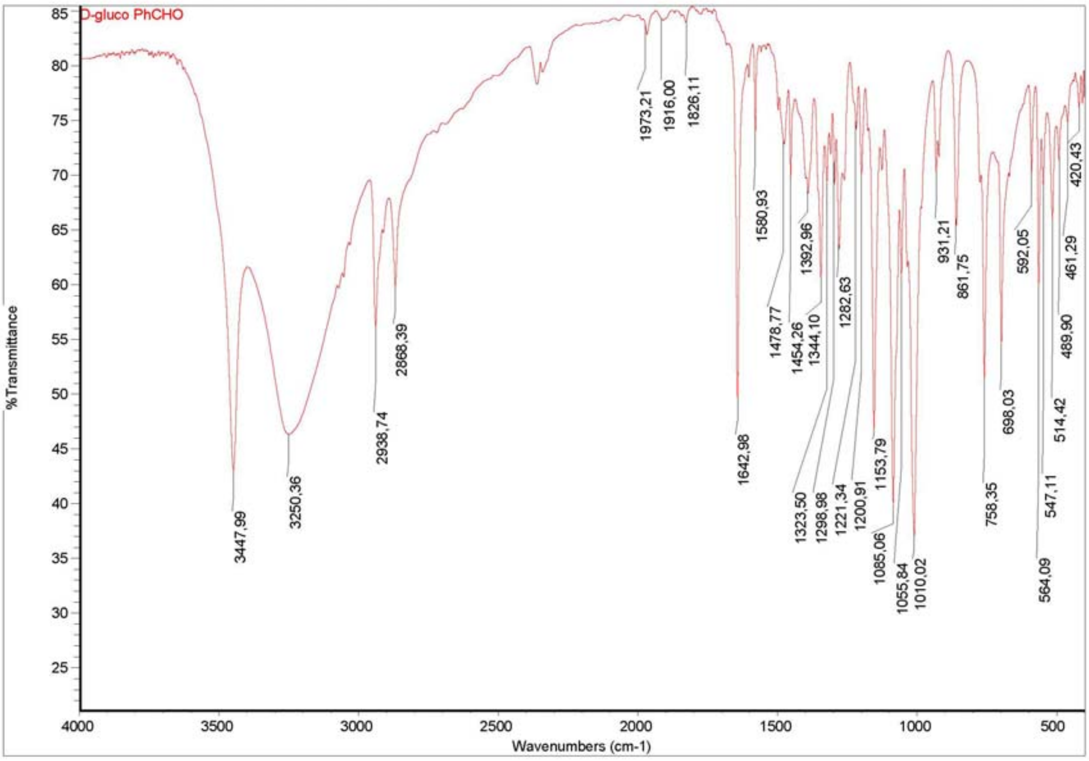

Figure S1. IR spectrum of **30**

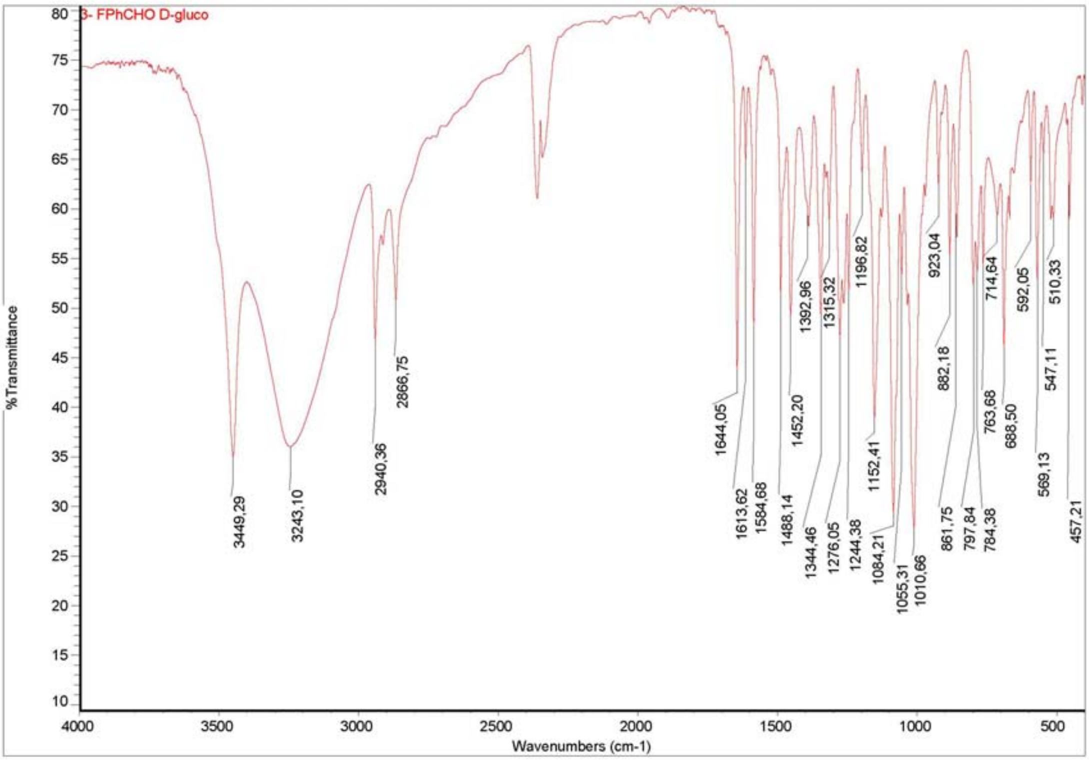

Figure S2. IR spectrum of **31**

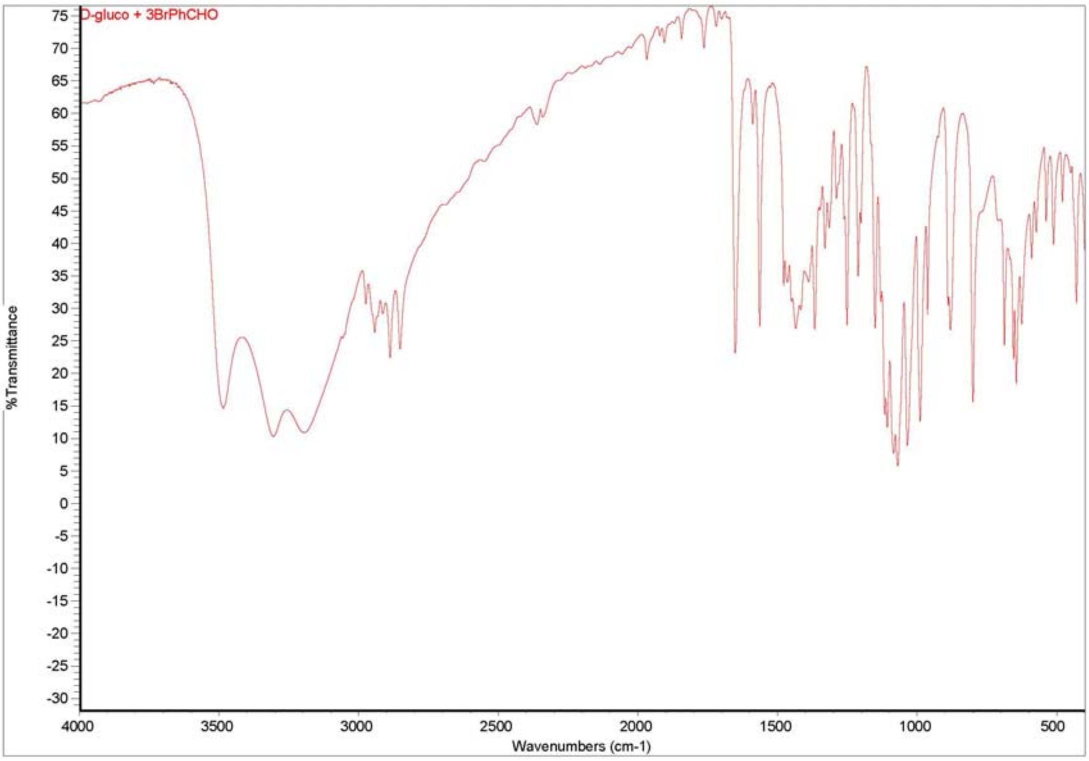

Figure S3. IR spectrum of **32**

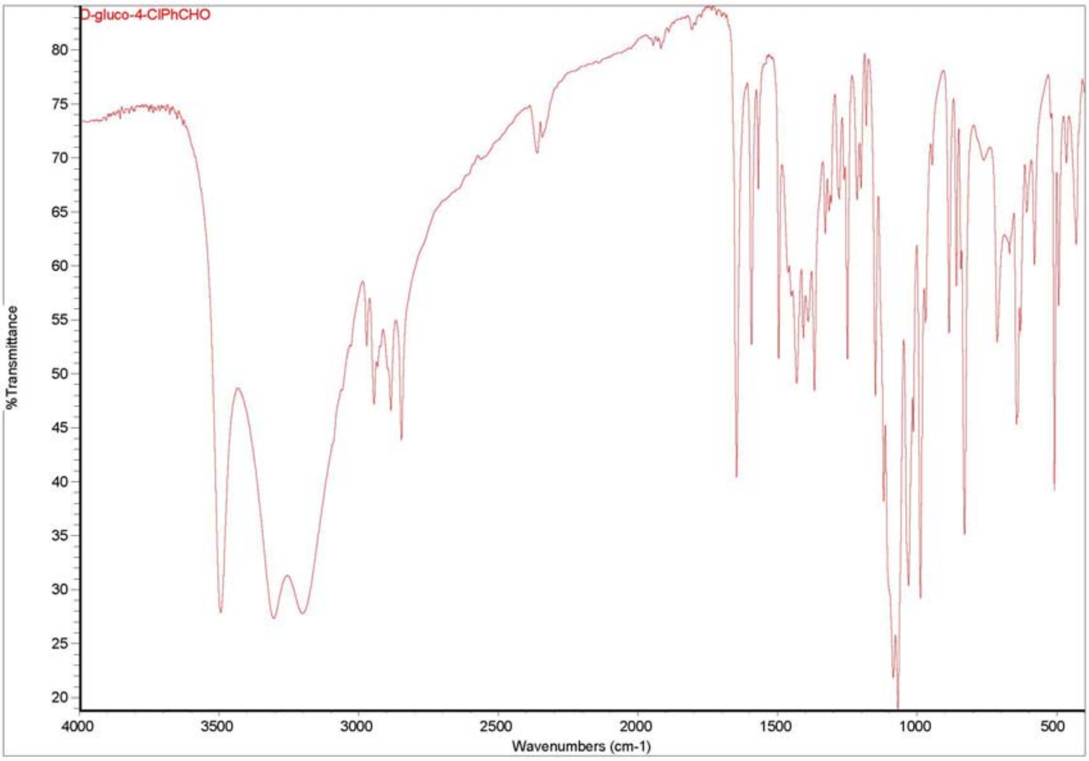

Figure S4. IR spectrum of **33**

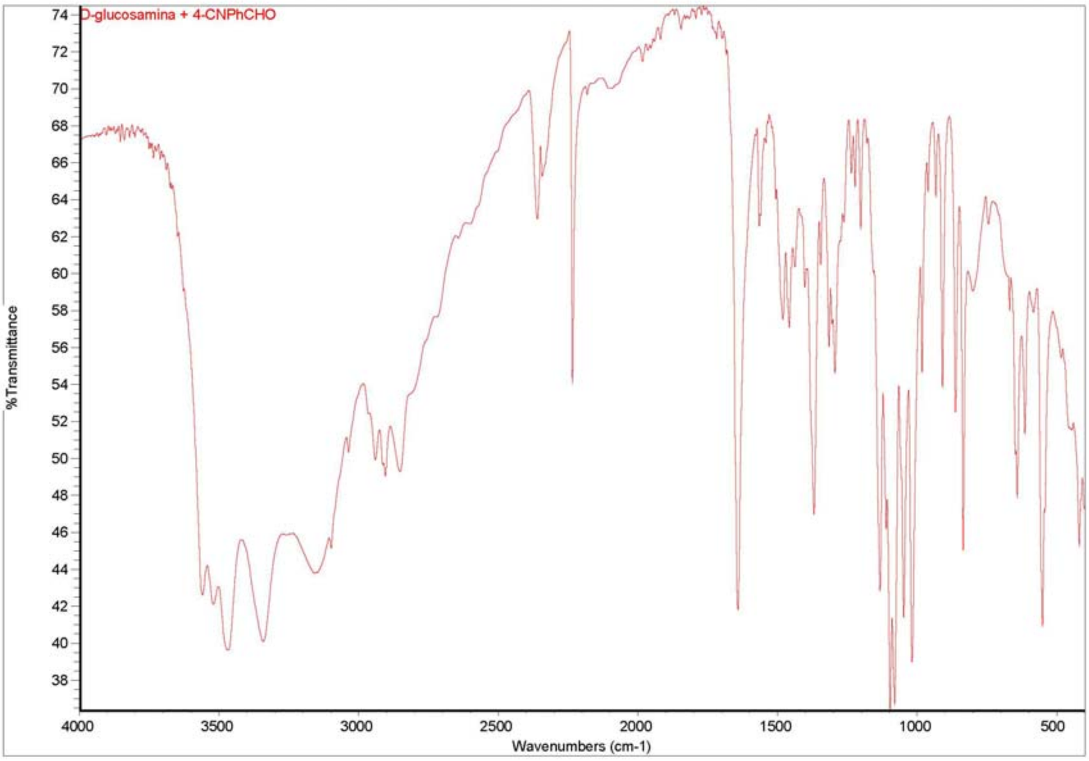

Figure S5. IR spectrum of **34**

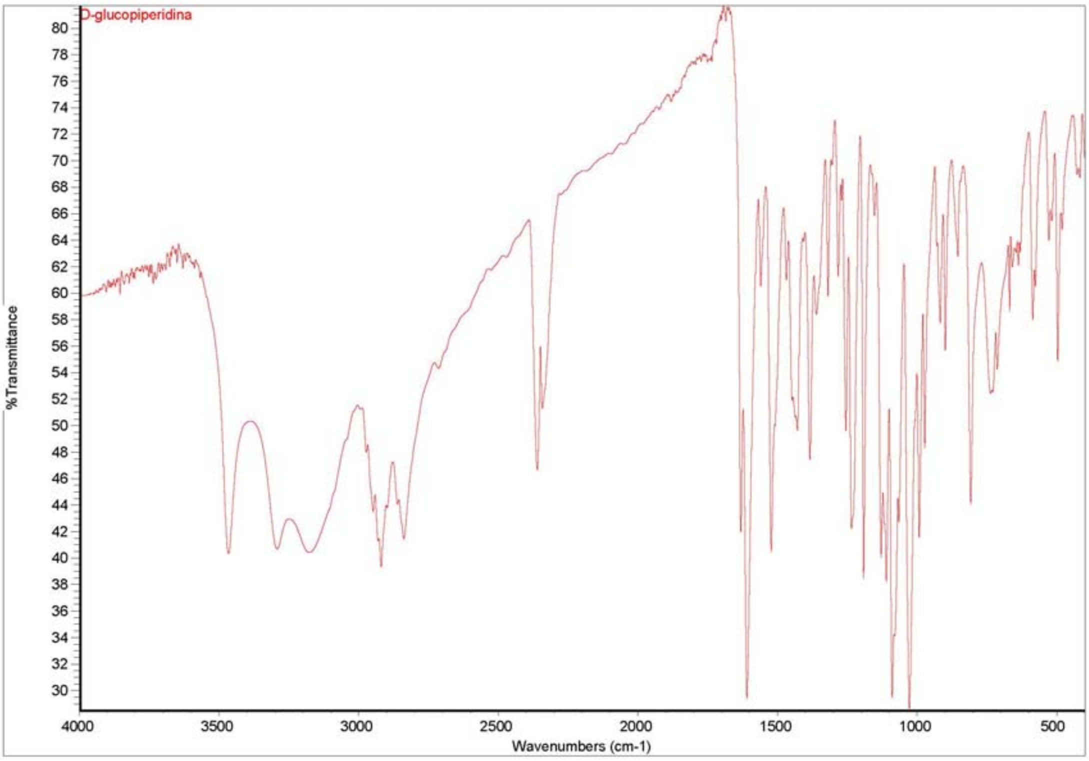

Figure S6. IR spectrum of **35**

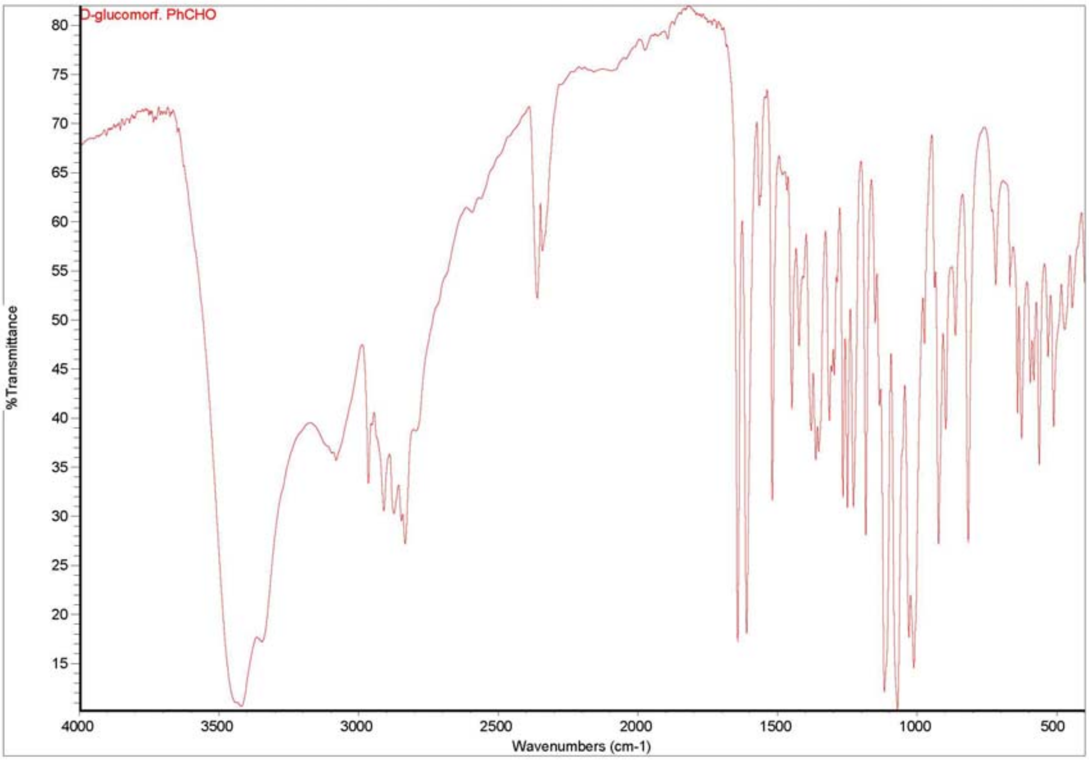

Figure S7. IR spectrum of **36**

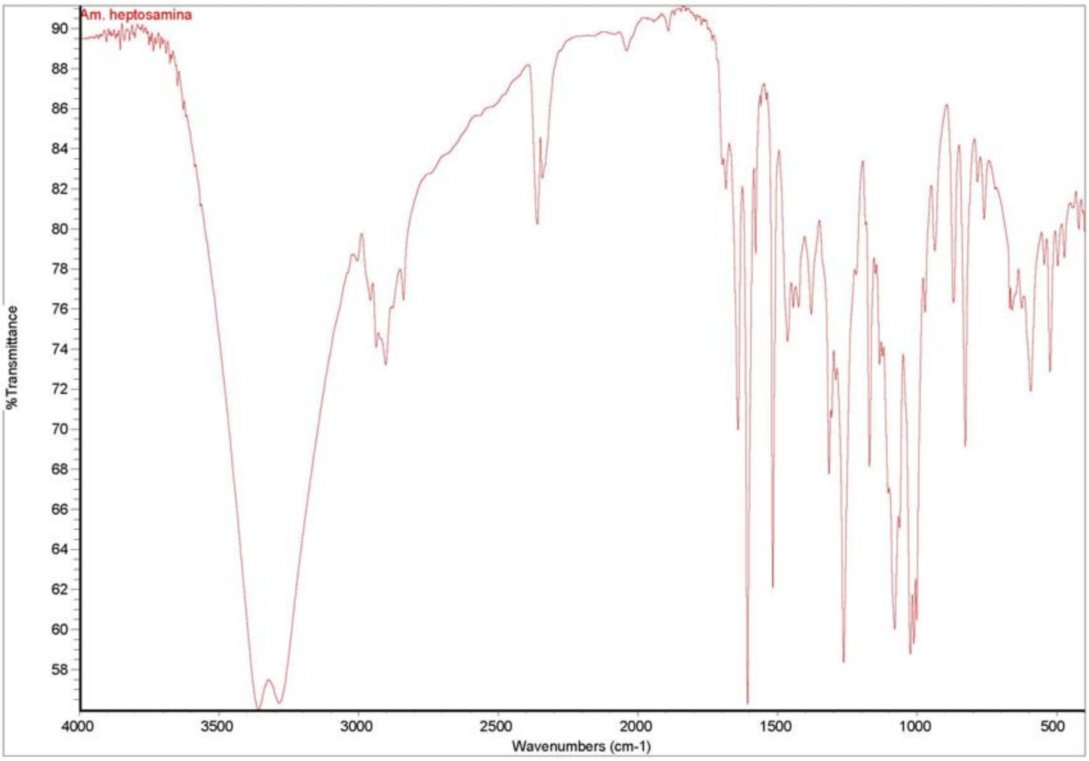

Figure S8. IR spectrum of **37**

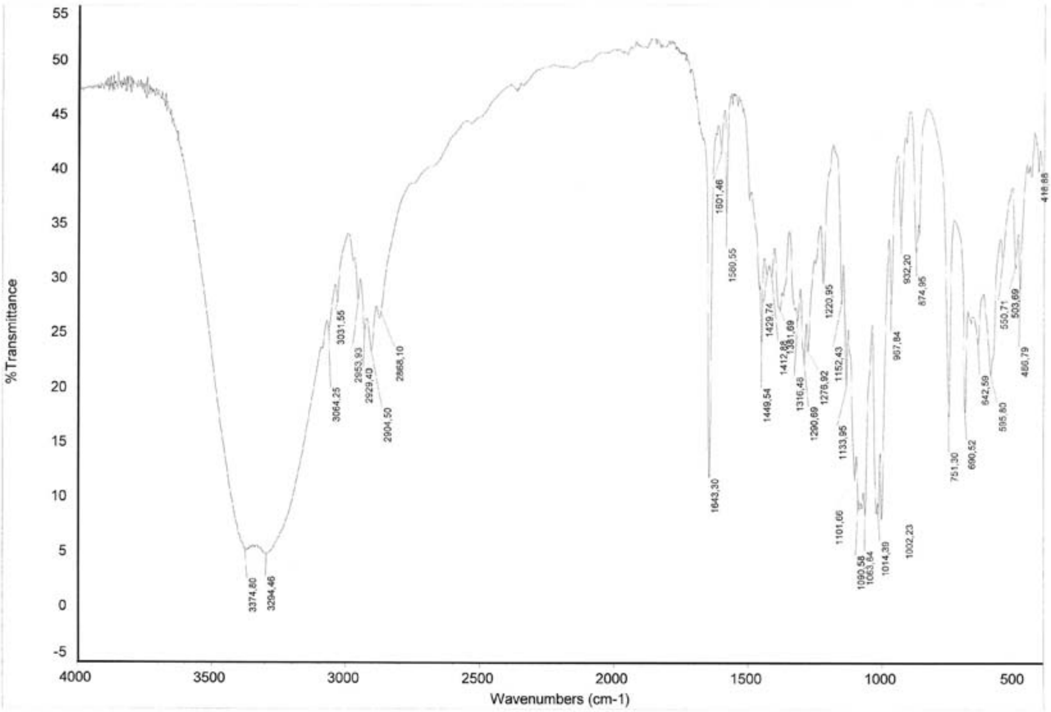

Figure S9. IR spectrum of **38**

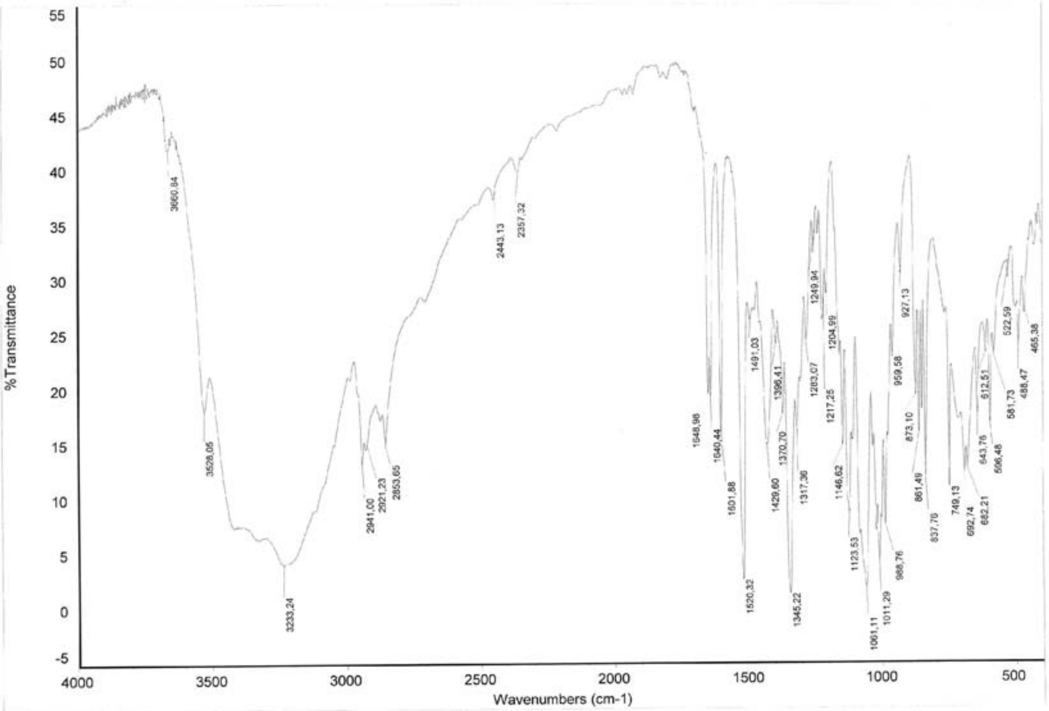

Figure S10. IR spectrum of **39**

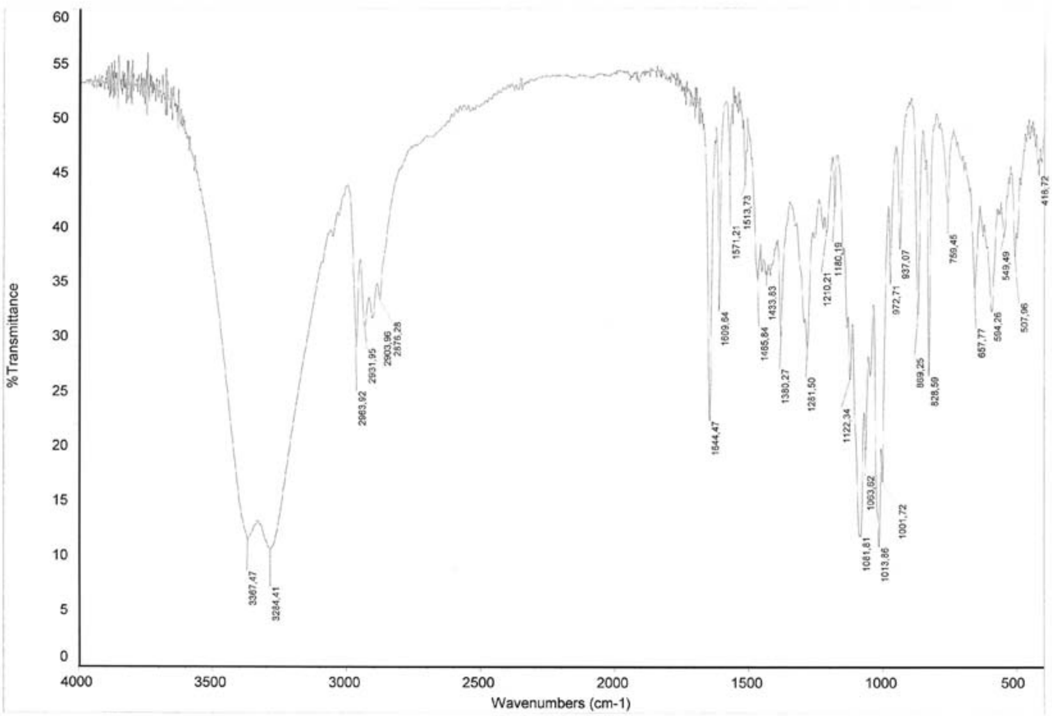

Figure S11. IR spectrum of **40**

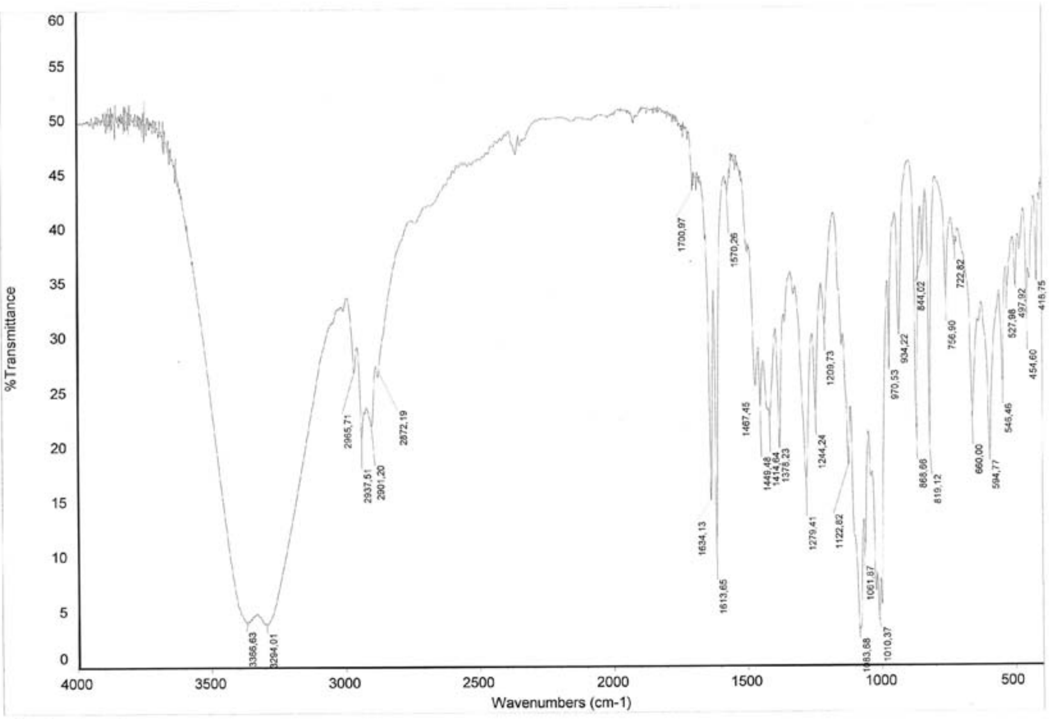

Figure S12. IR spectrum of **41**

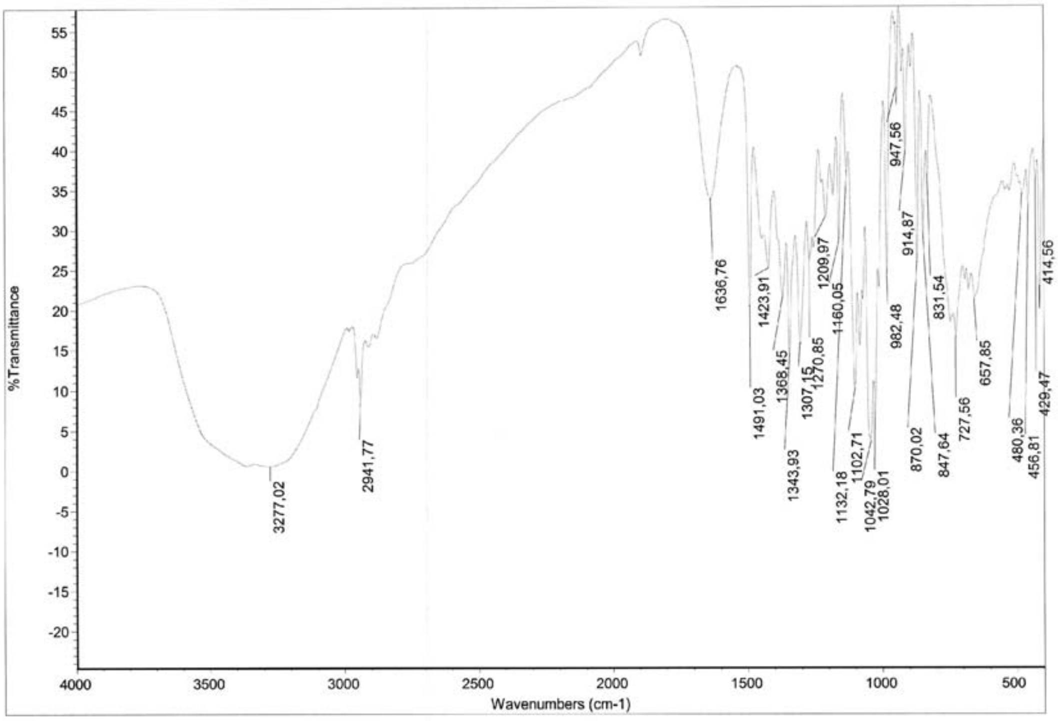

Figure S13. IR spectrum of **43**

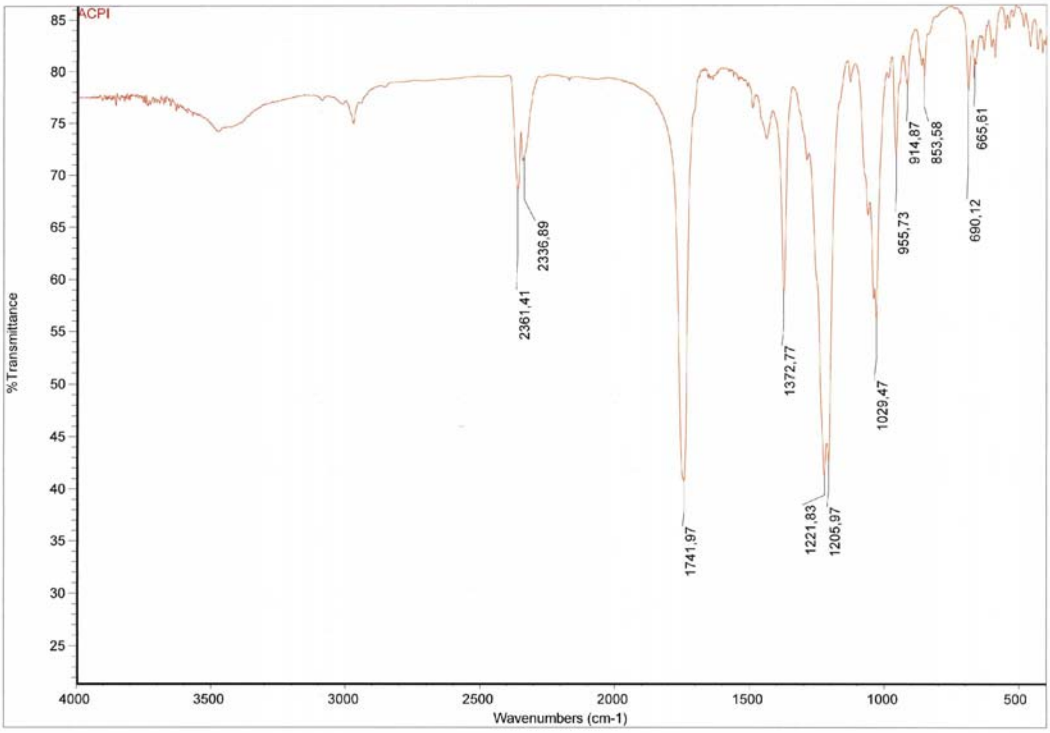

Figure S14. IR spectrum of **44**

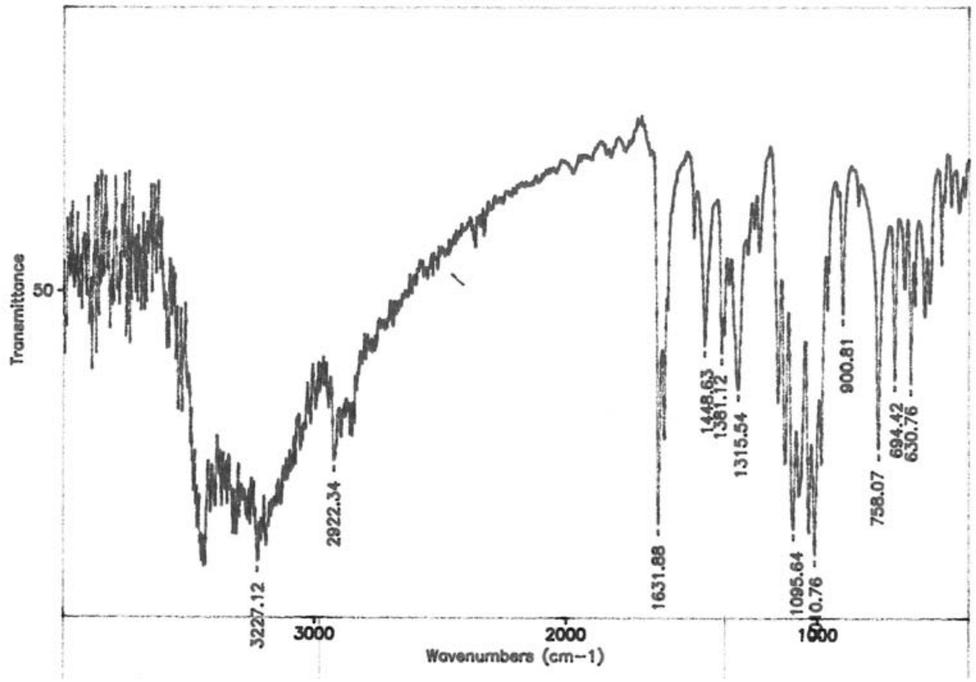

Figure S15. IR spectrum of **45**

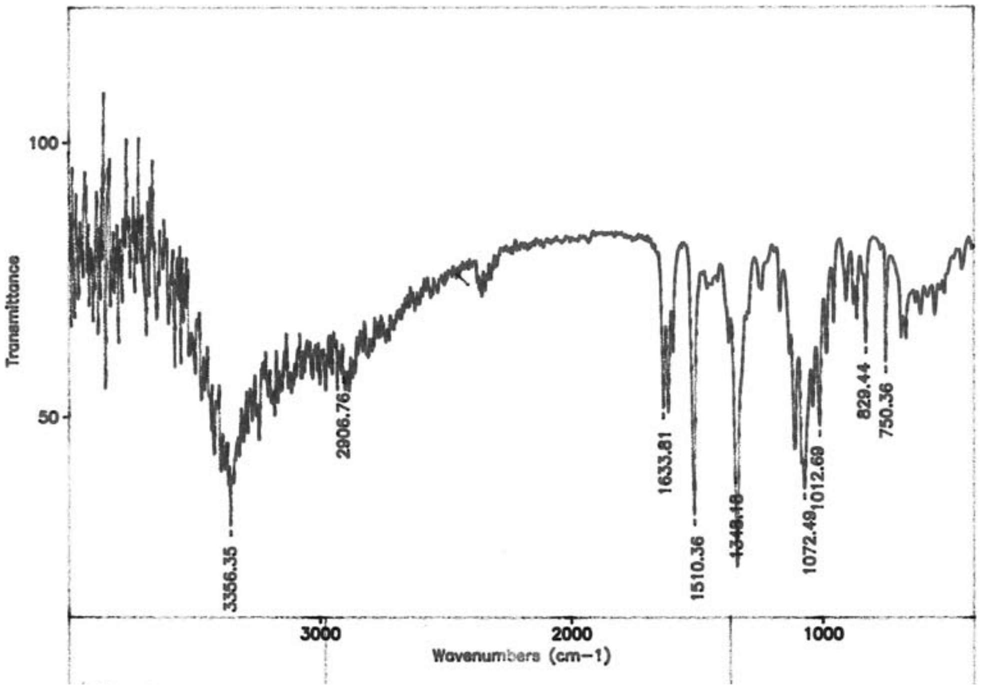

Figure S16. IR spectrum of **46**

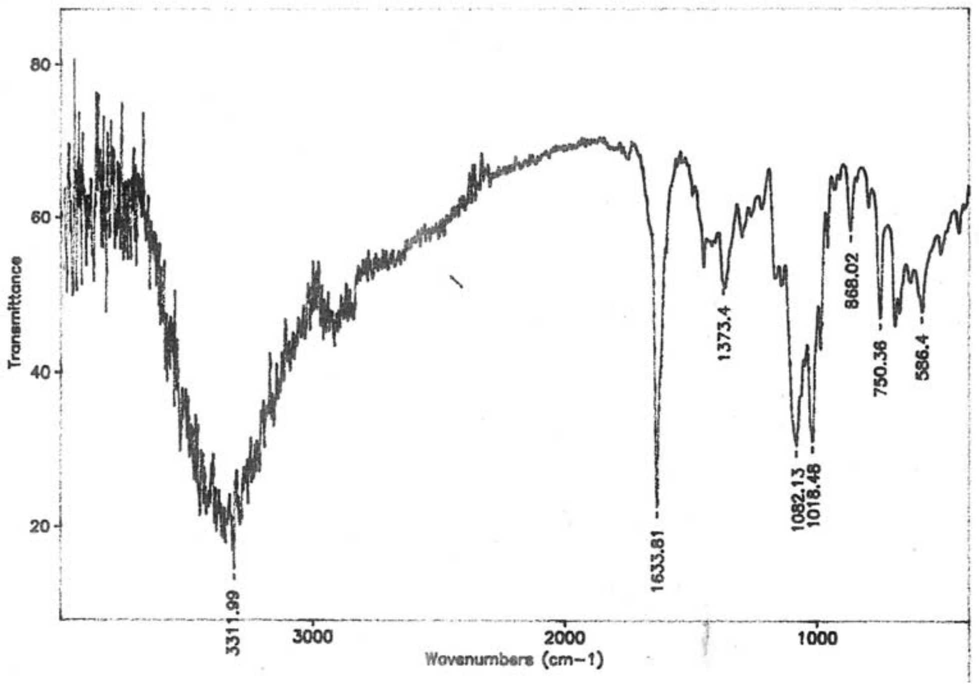

Figure S17. IR spectrum of **49**

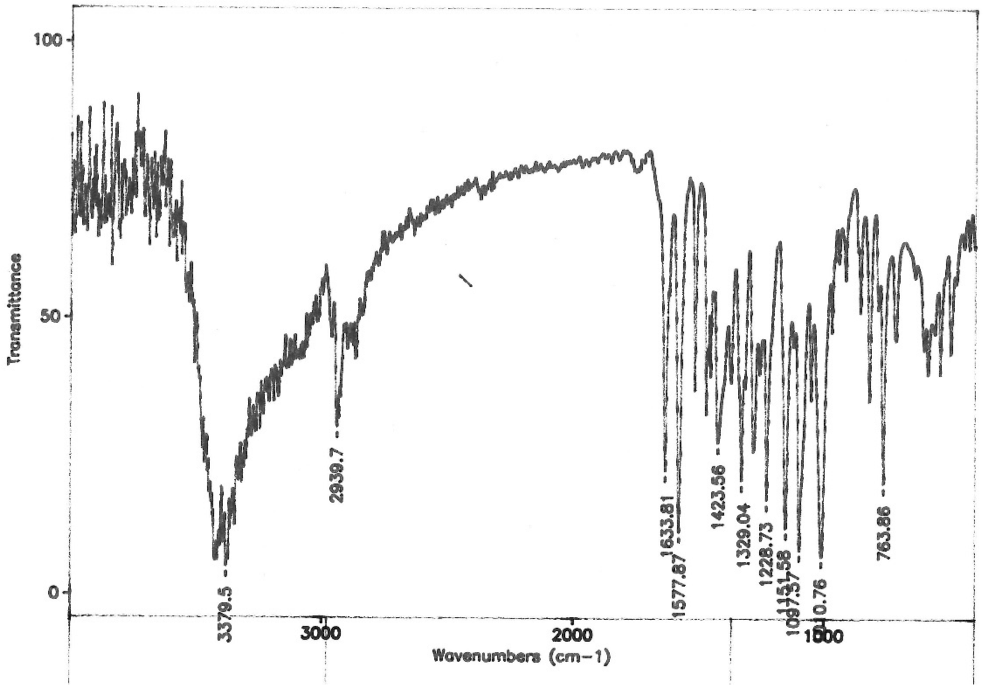

Figure S18. IR spectrum of **51**

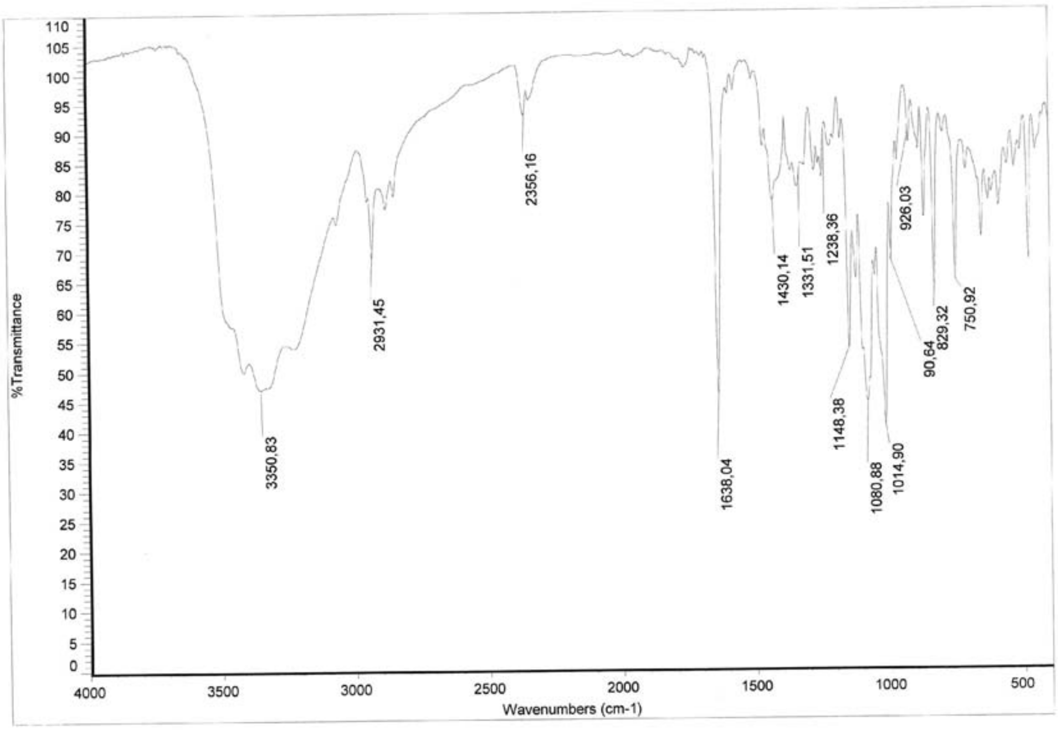

Figure S19. IR spectrum of **54**

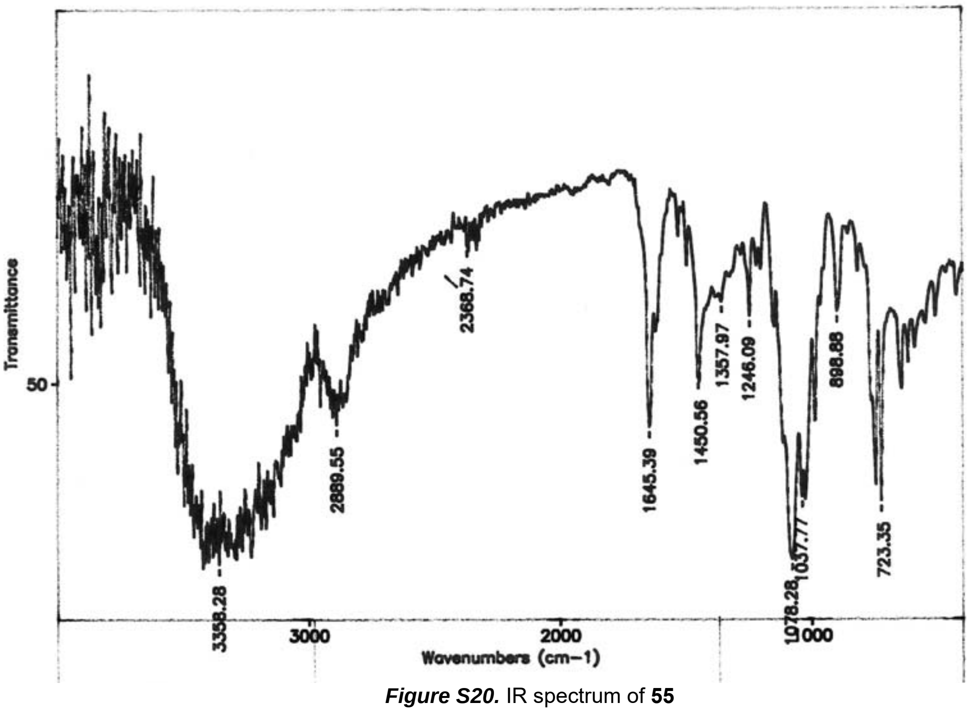

Figure S20. IR spectrum of **55**

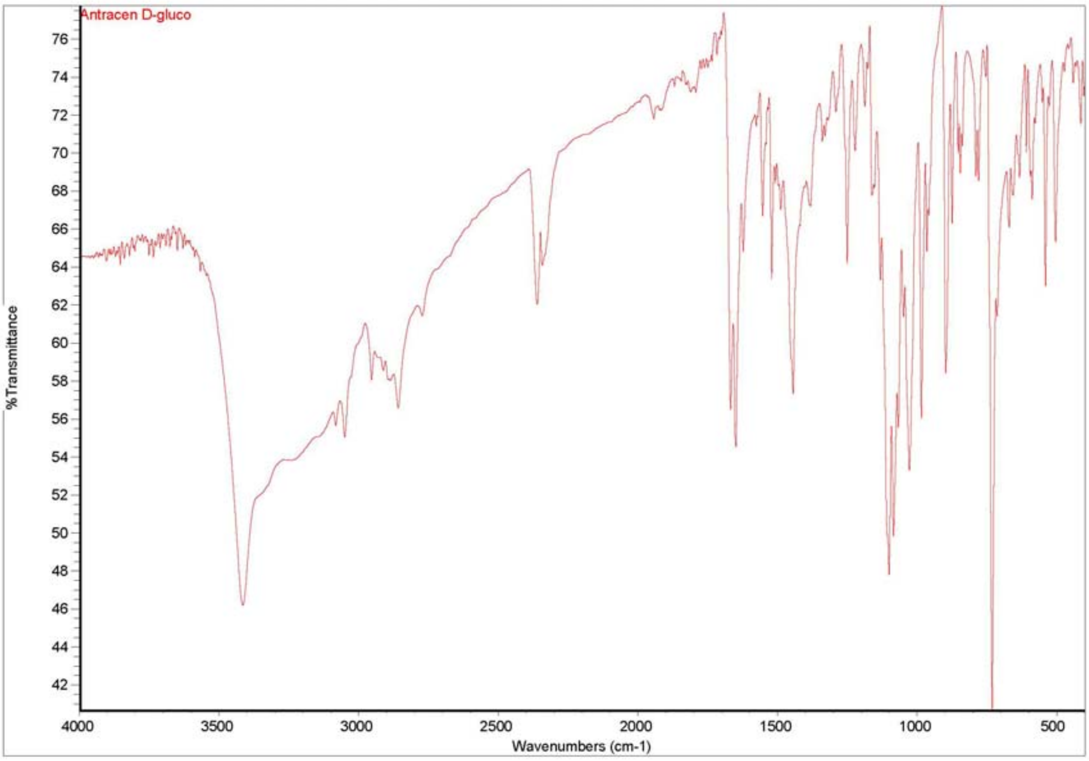

Figure S21. IR spectrum of **56**

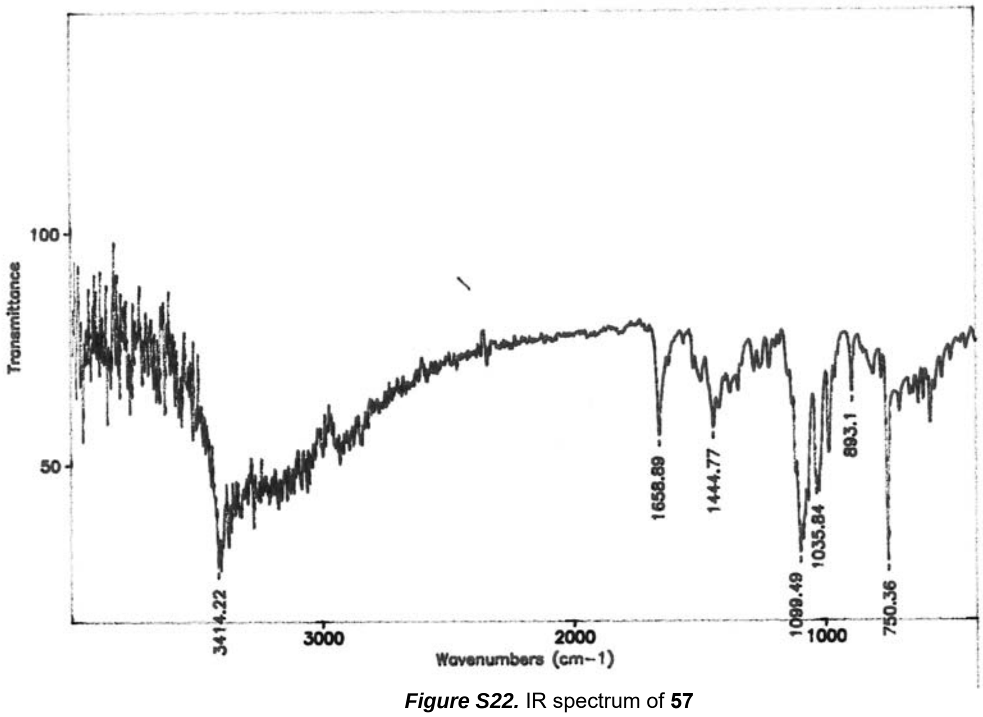

Figure S22. IR spectrum of **57**

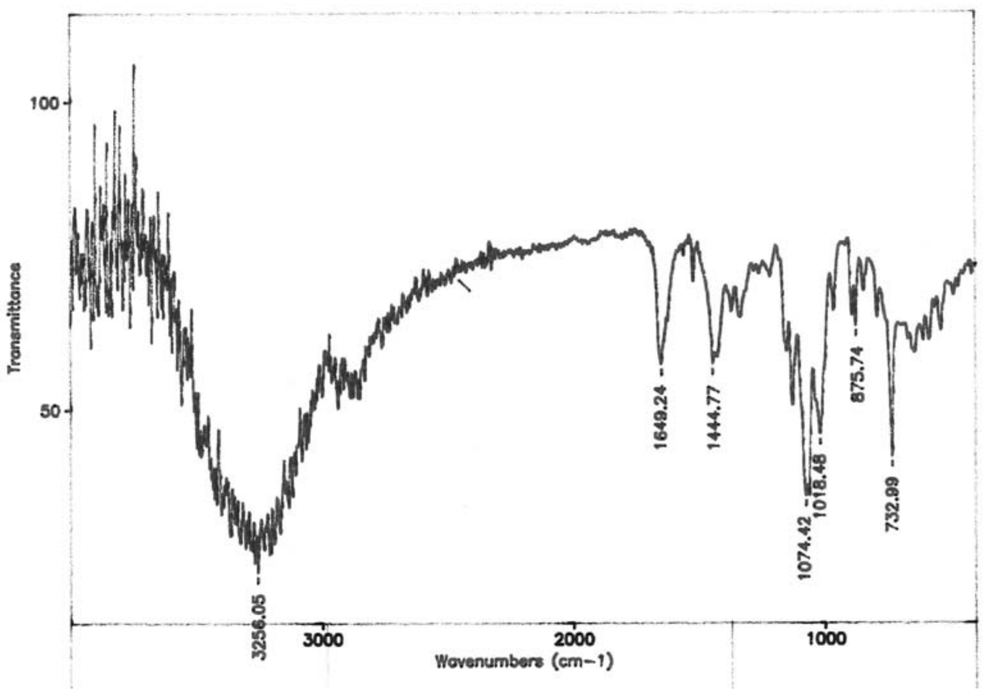

Figure S23. IR spectrum of **58**

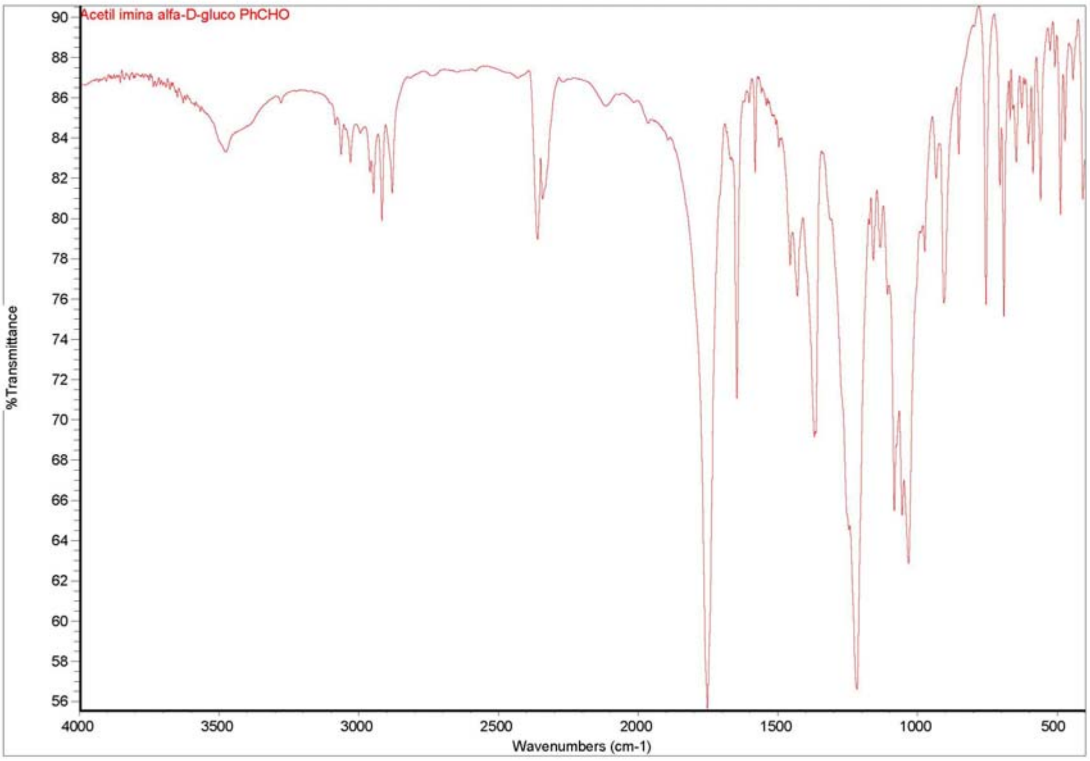

Figure S24. IR spectrum of **63**

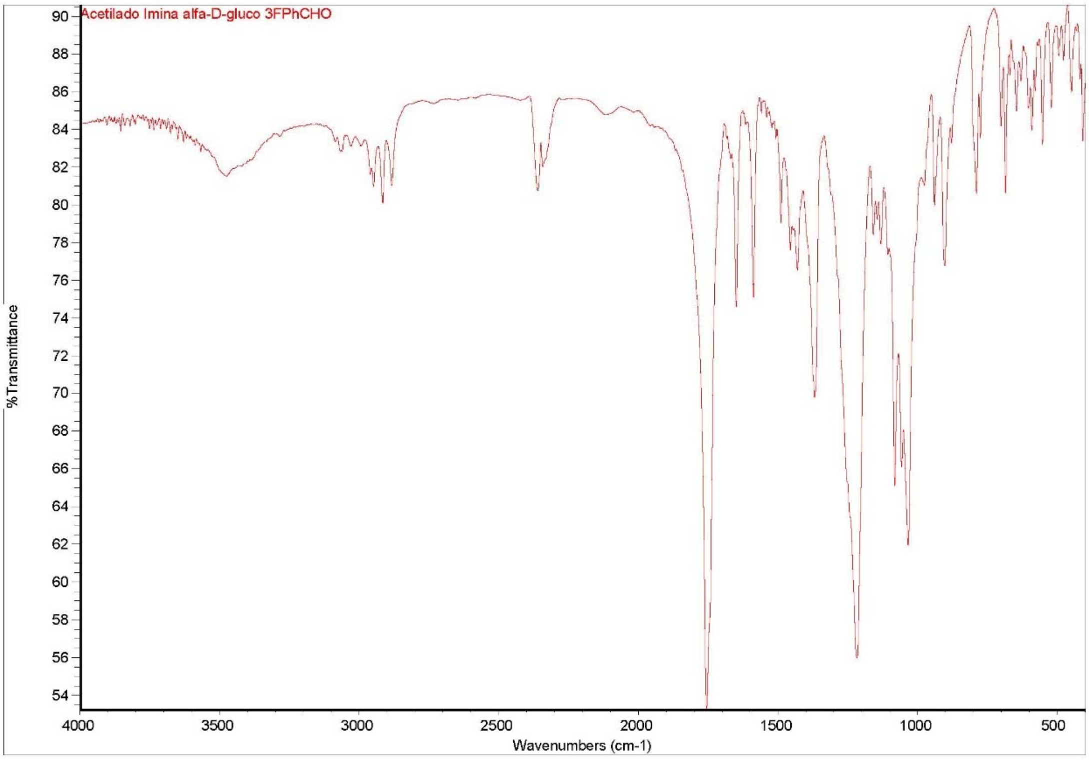

Figure S25. IR spectrum of **64**

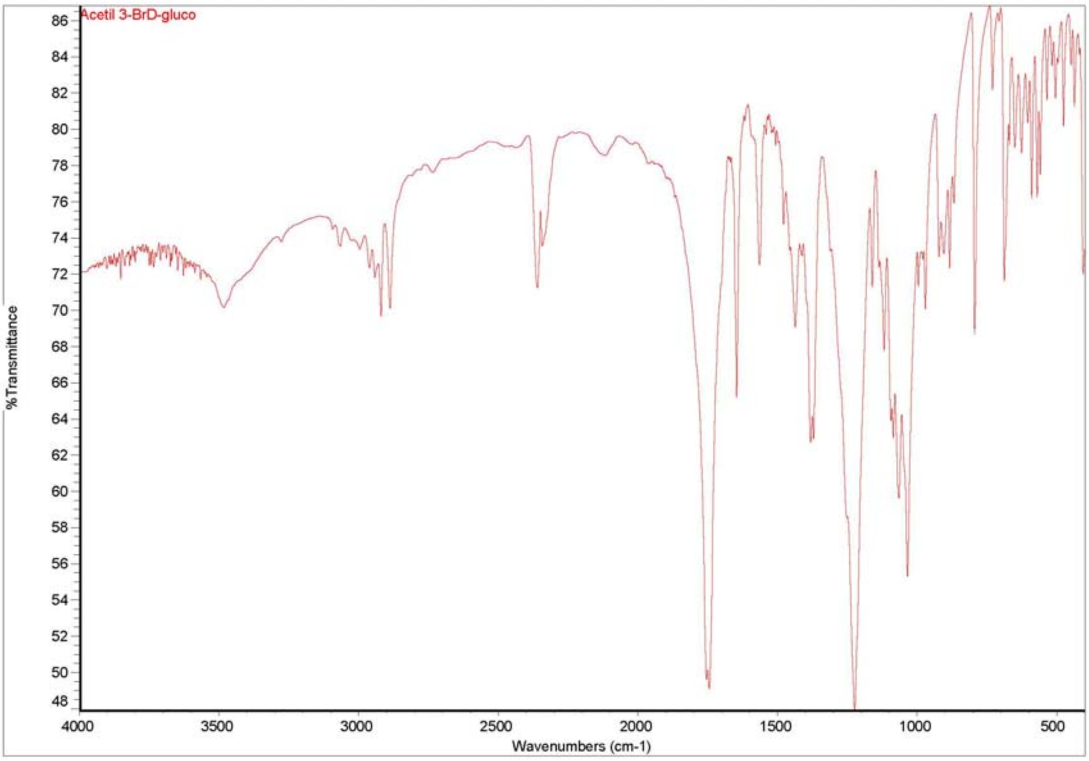

Figure S26. IR spectrum of **65**

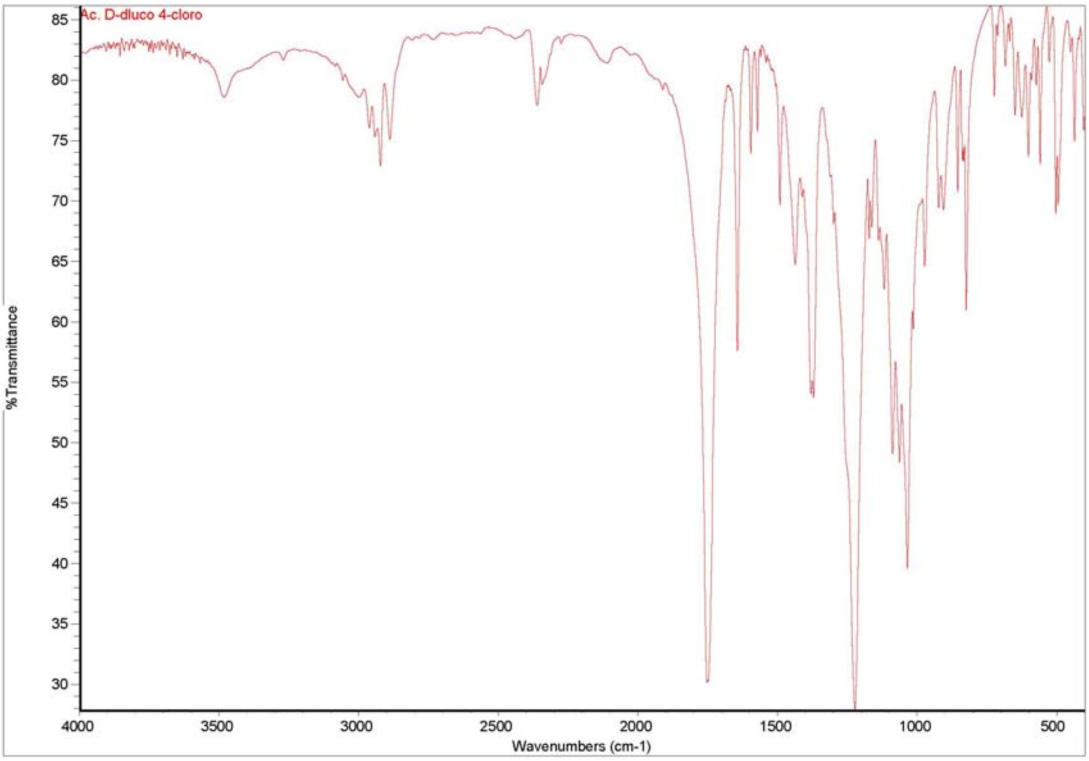

Figure S27. IR spectrum of **66**

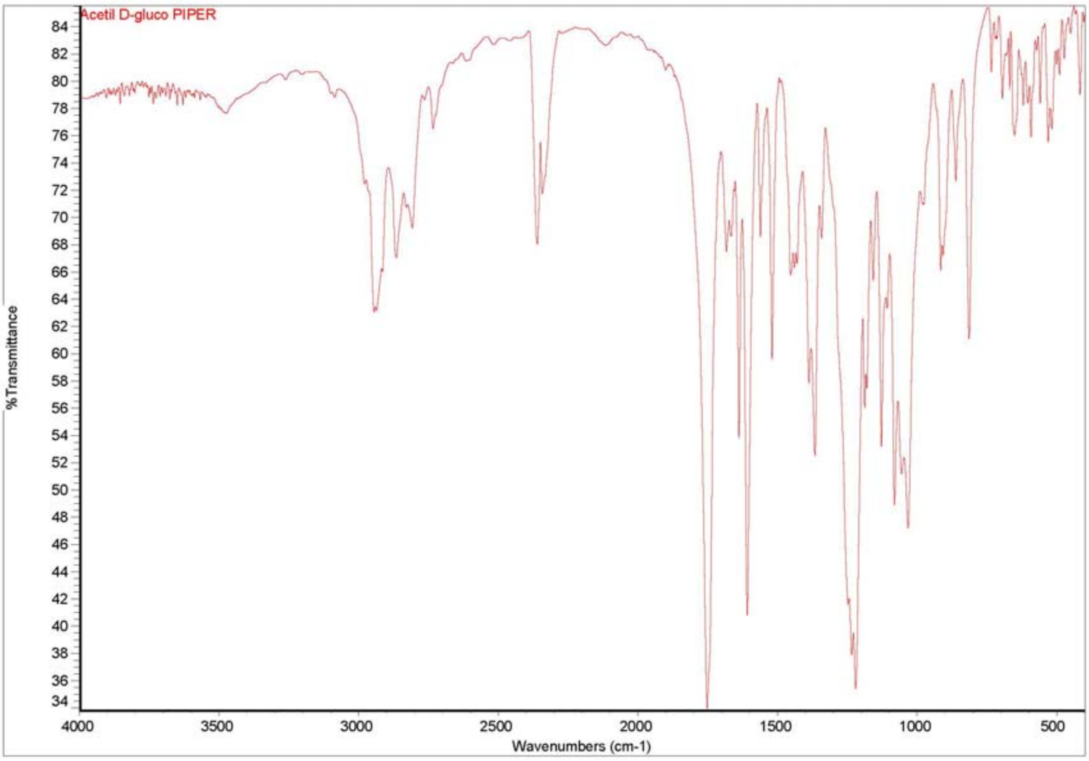

Figure S28. IR spectrum of **67**

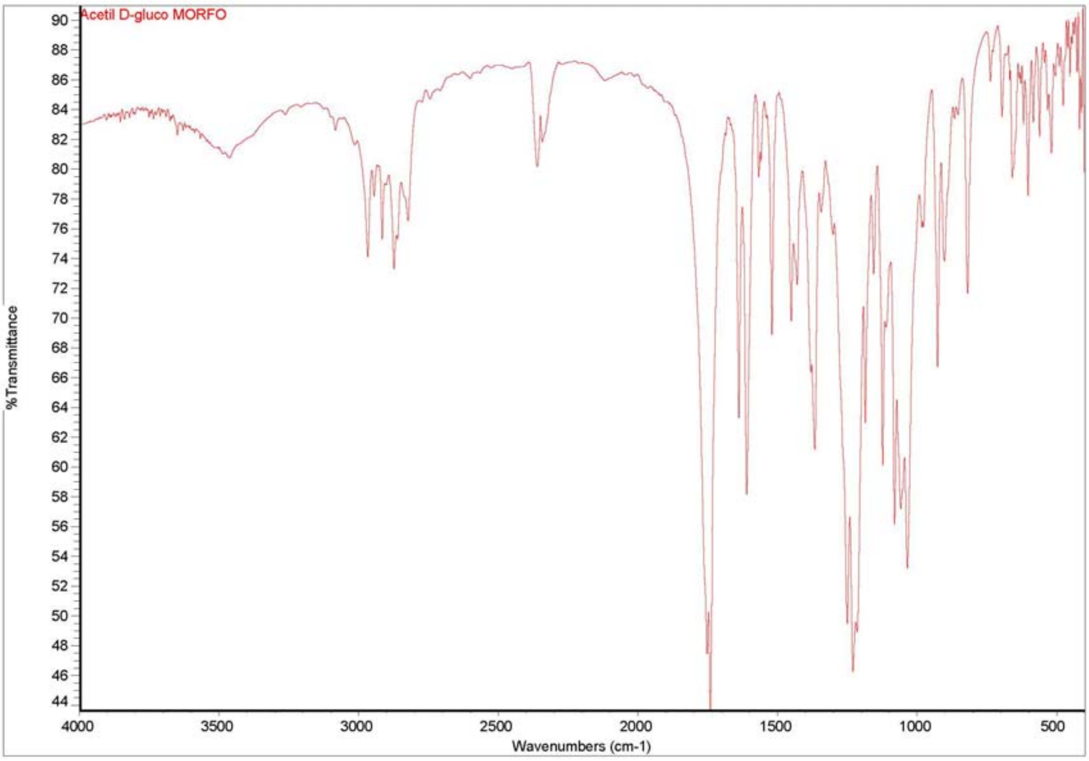

Figure S29. IR spectrum of **68**

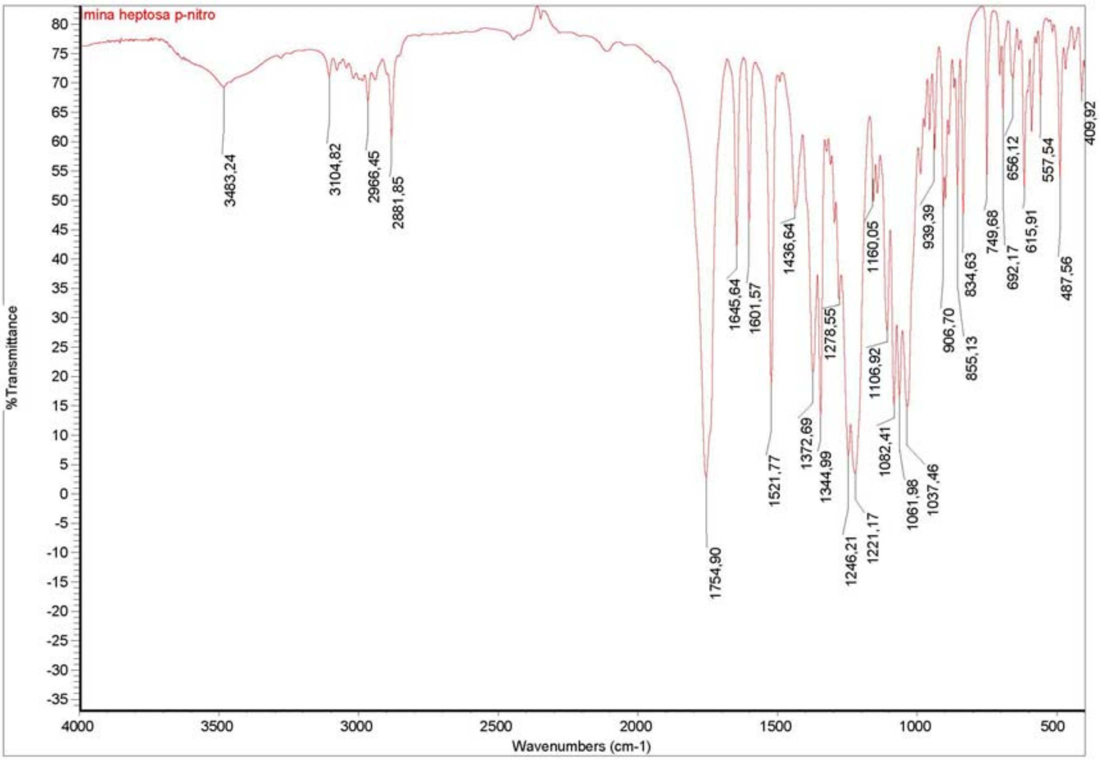

Figure S30. IR spectrum of **70**

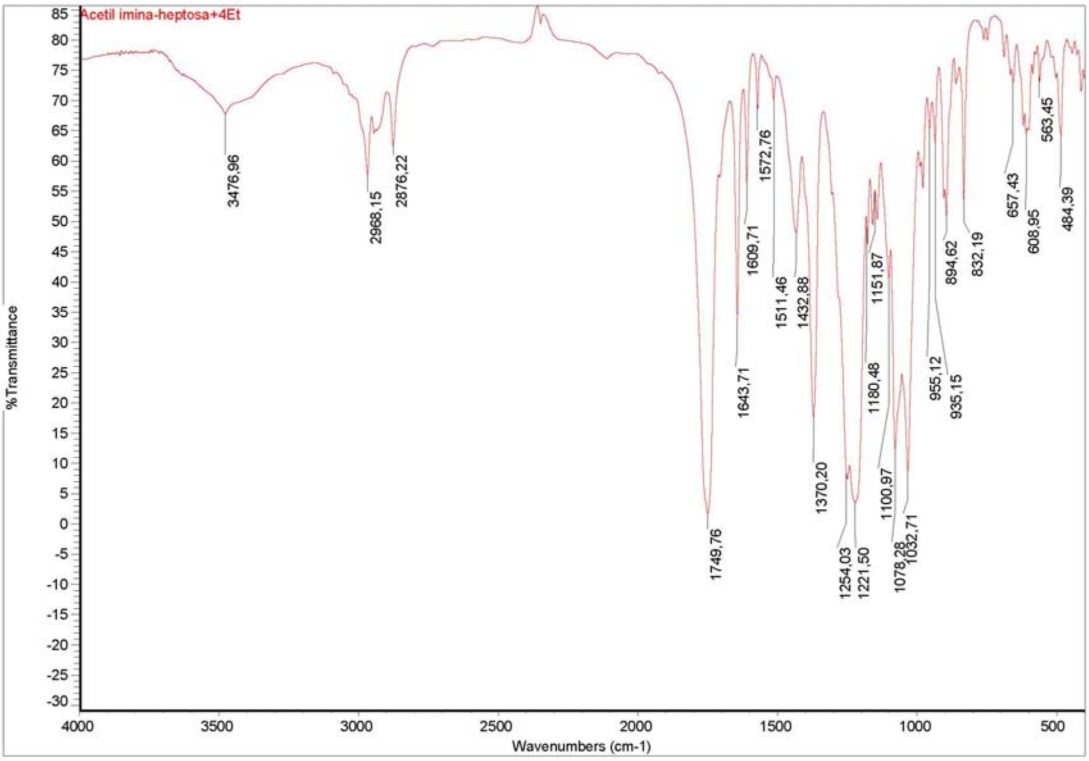

Figure S31. IR spectrum of **71**

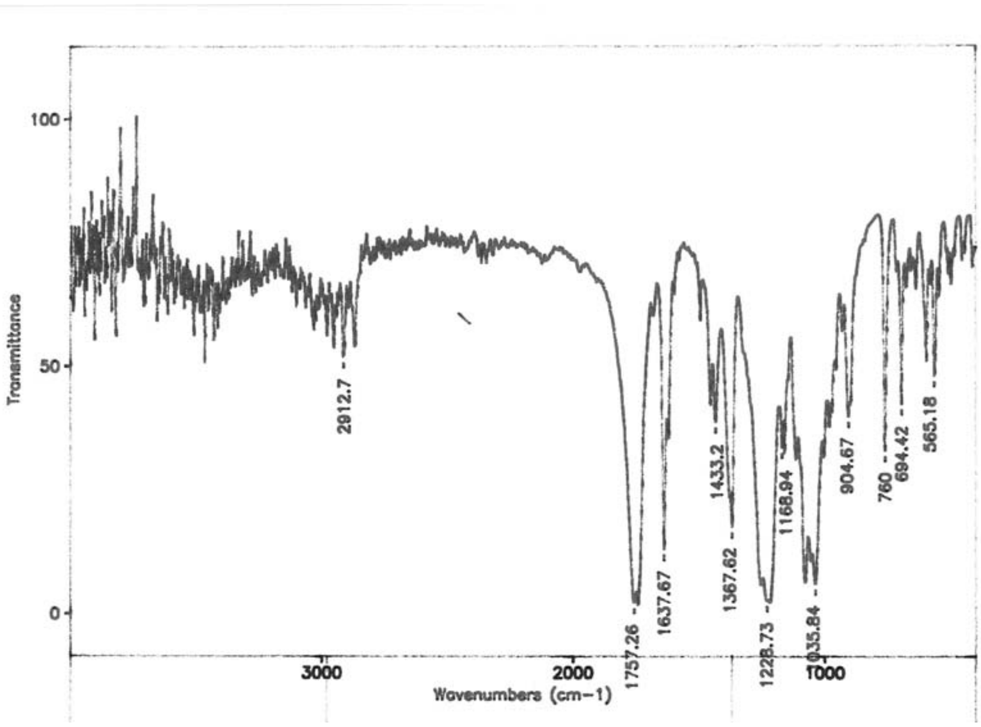

Figure S32. IR spectrum of **73**

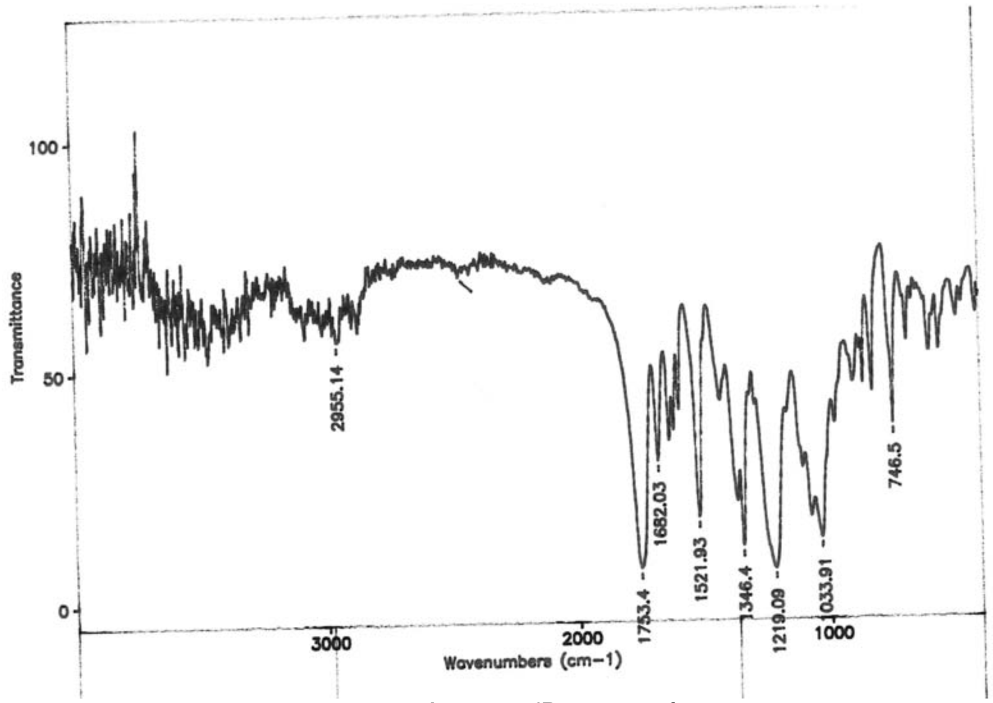

Figure S33. IR spectrum of **74**

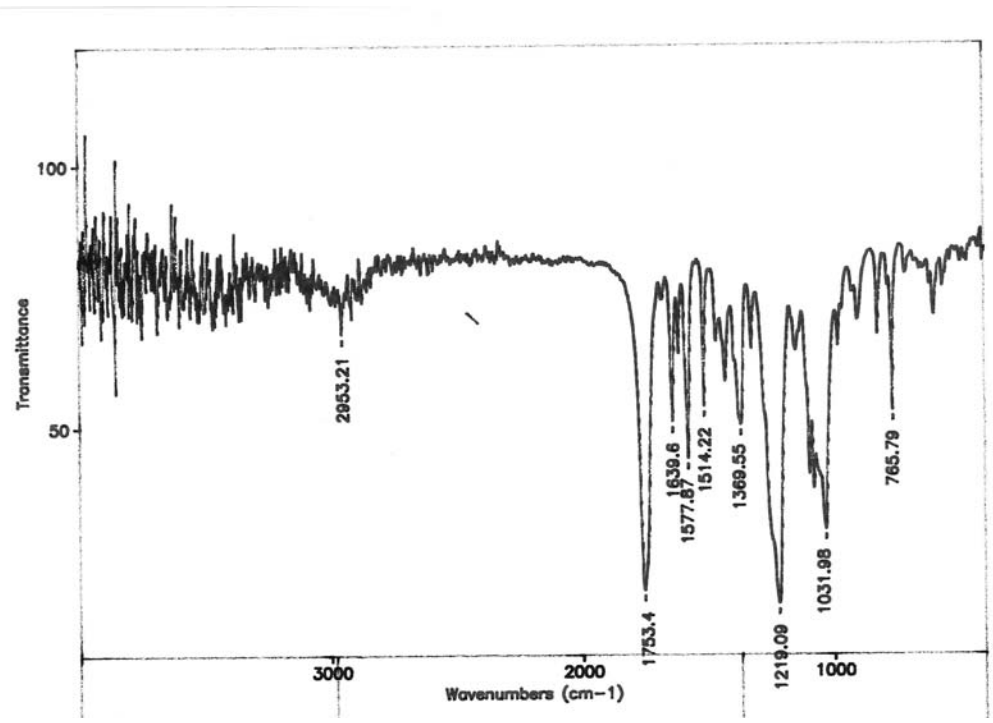

Figure S34. IR spectrum of **77**

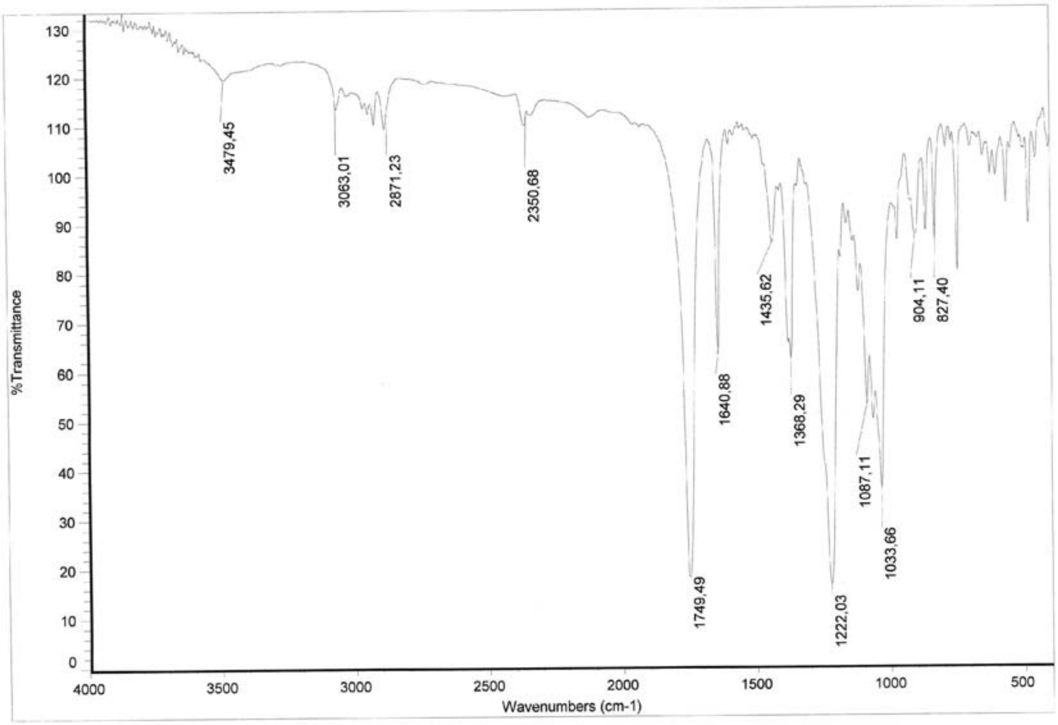

Figure S35. IR spectrum of **79**

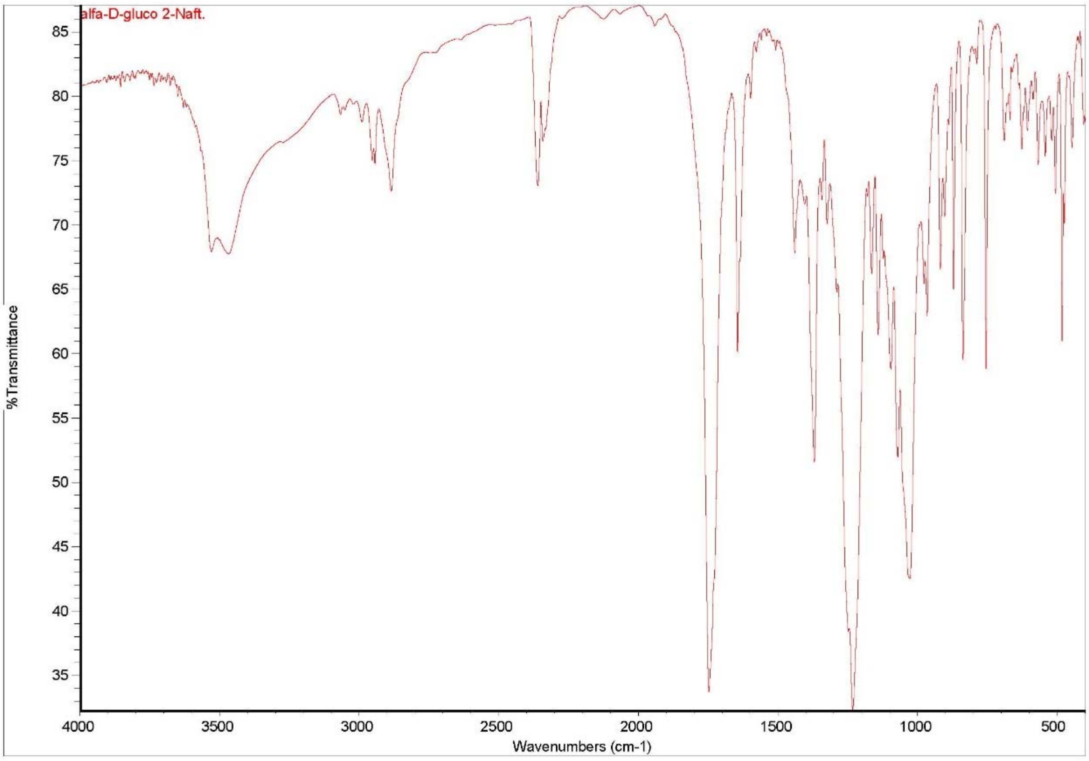

Figure S36. IR spectrum of **80**

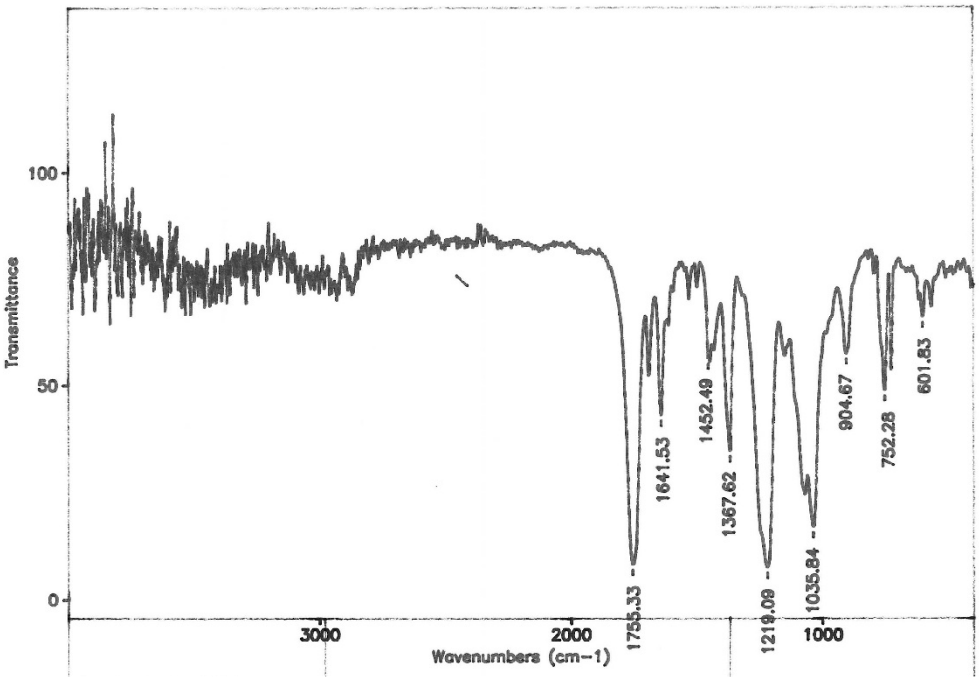

Figure S37. IR spectrum of **81**

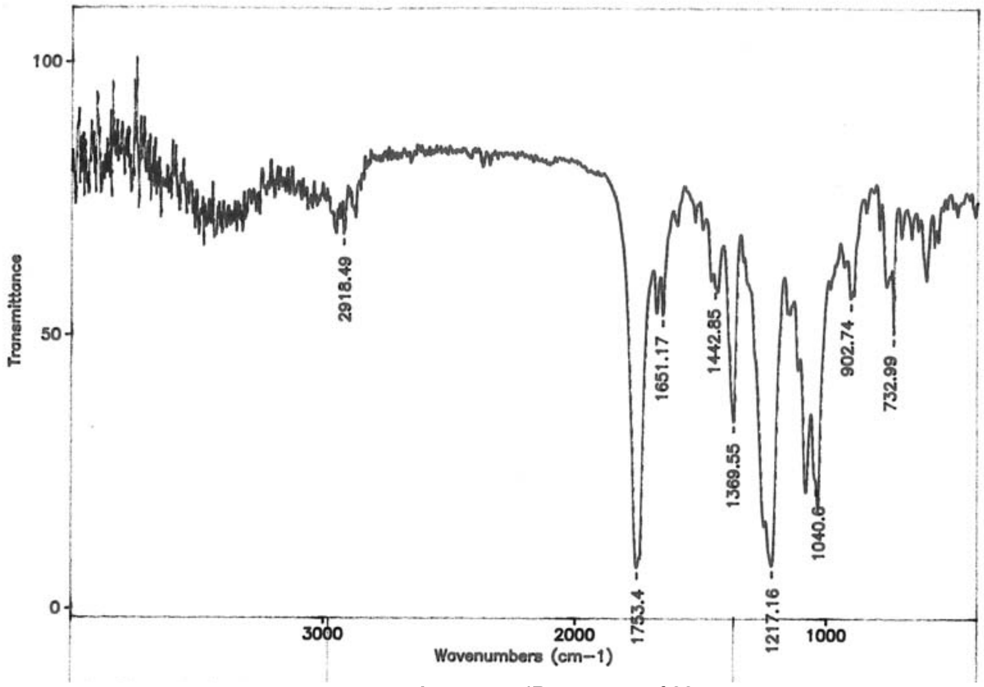

Figure S38. IR spectrum of **82**

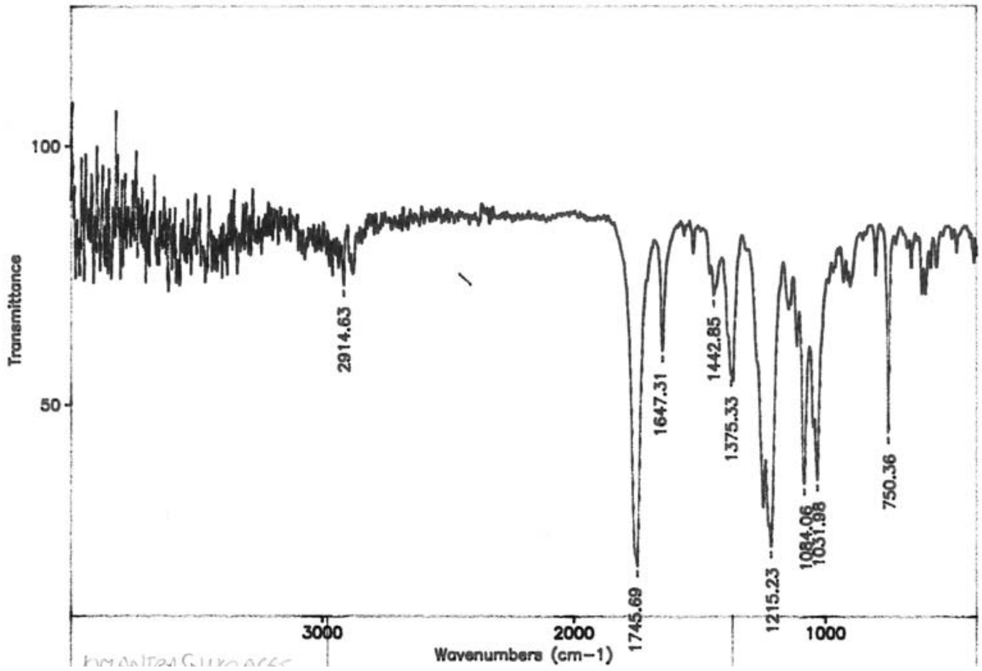

Figure S39. IR spectrum of **83**

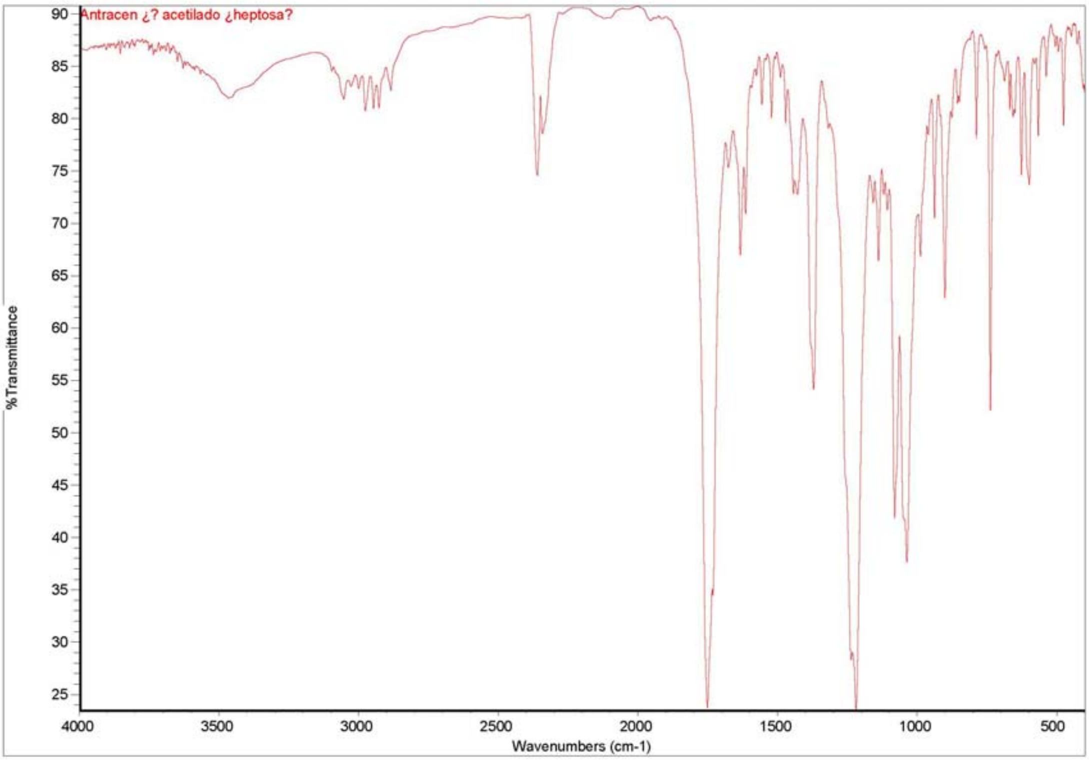

Figure S40. IR spectrum of **84**

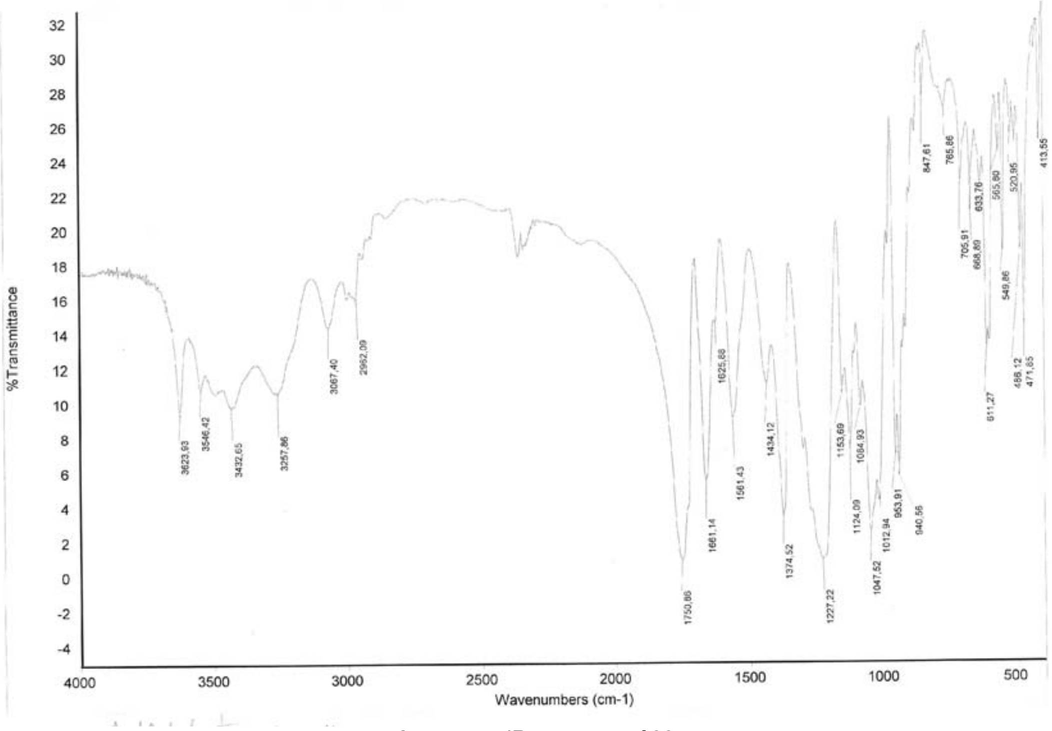

Figure S41. IR spectrum of **90**

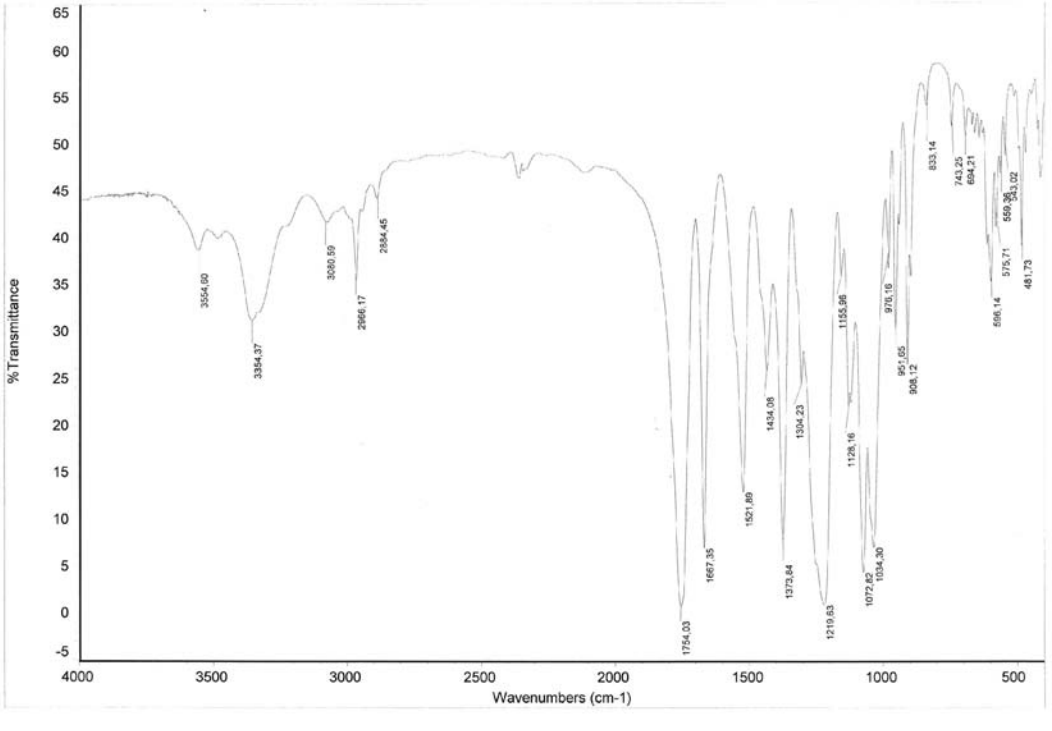

Figure S42. IR spectrum of **92**

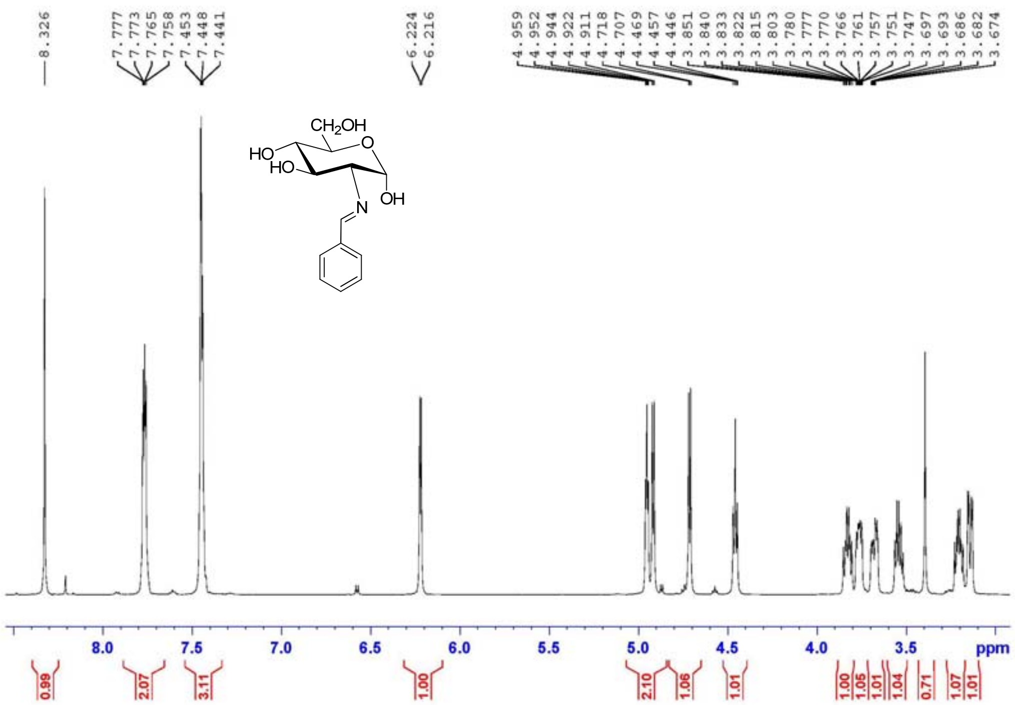

Figure S43. ^{1}H NMR spectrum of **30** in DMSO-d_6.

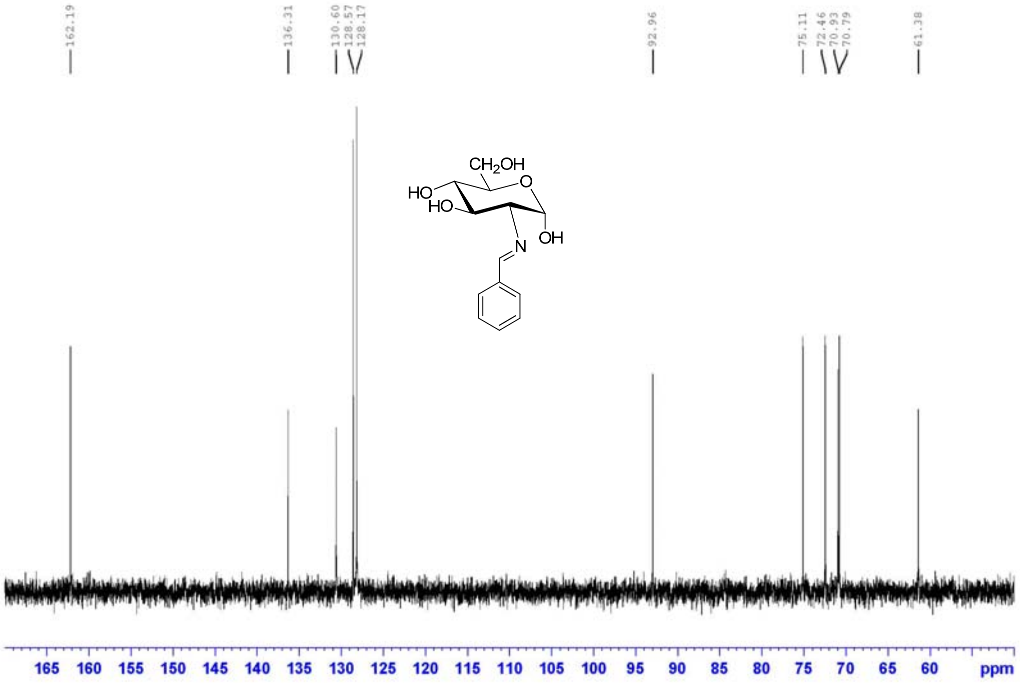

Figure S44. ^{13}C {^{1}H} NMR spectrum of **30** in DMSO-d_6.

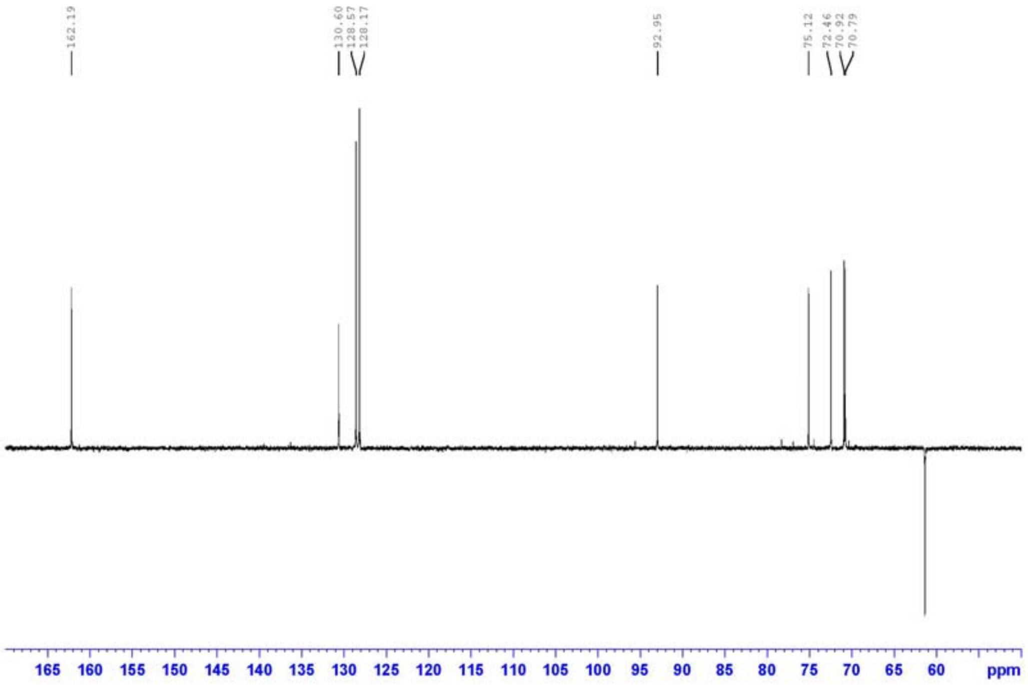

Figure S45. DEPT spectrum of **30** in DMSO-*d*6.

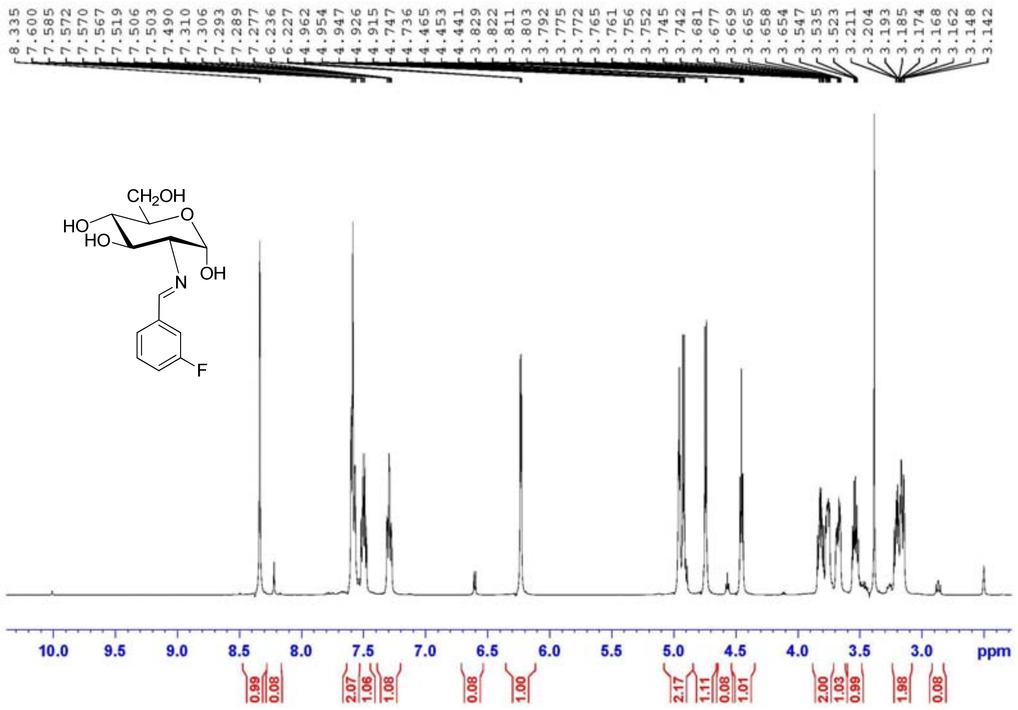

Figure S46. ^{1}H NMR spectrum of **31** in DMSO-*d*6.

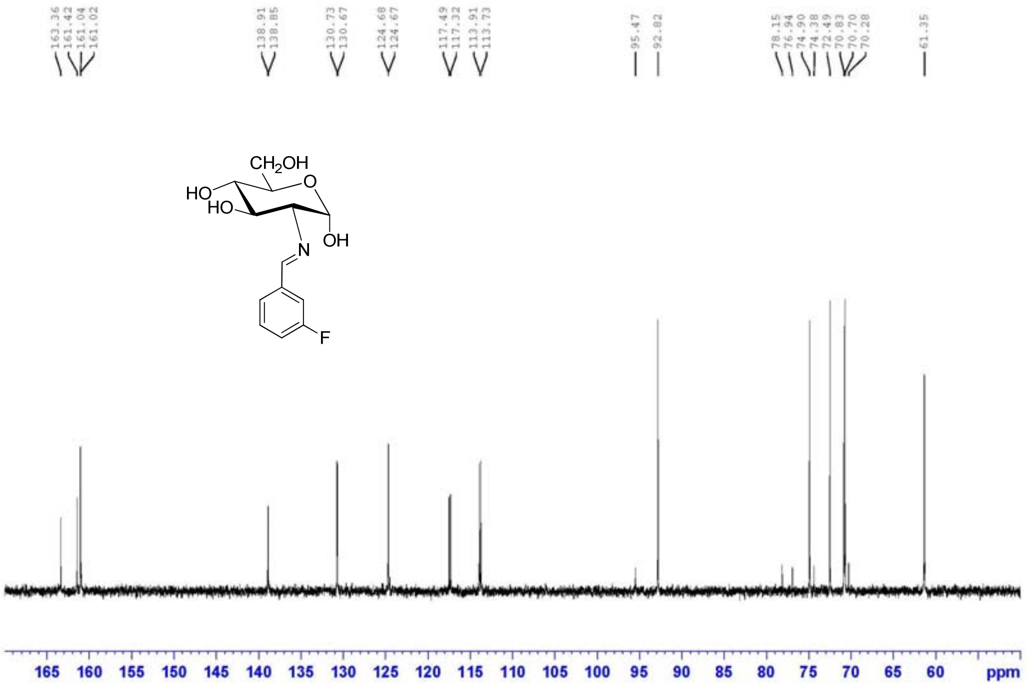

Figure S47. ^{13}C {^{1}H} NMR spectrum of **31** in DMSO-*d6*.

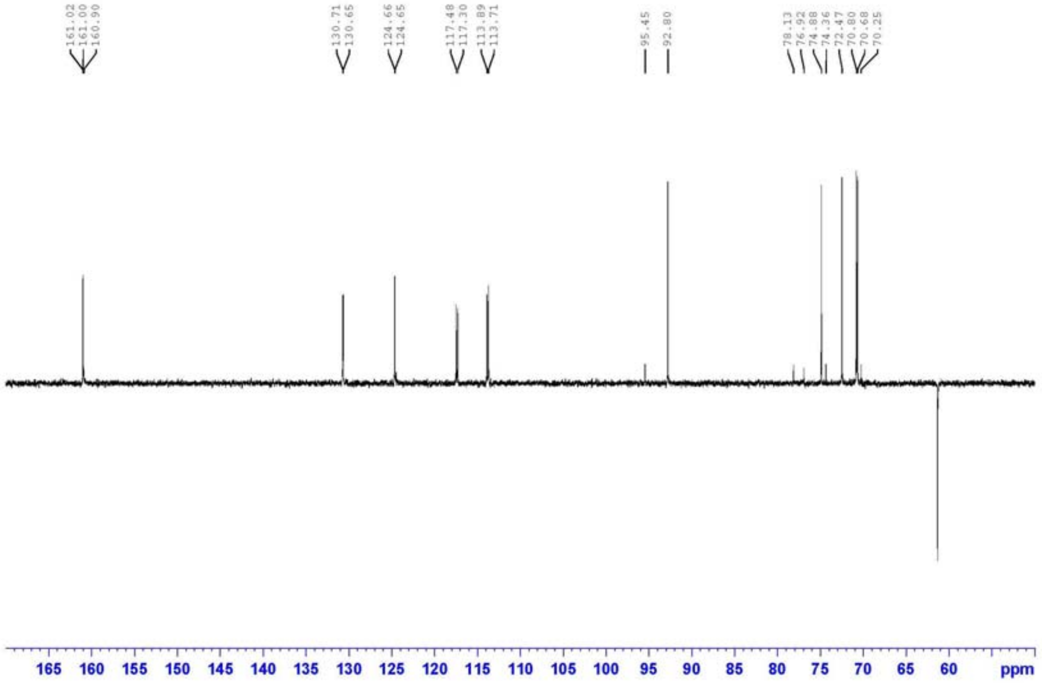

Figure S48. DEPT spectrum of **31** in DMSO-*d6*.

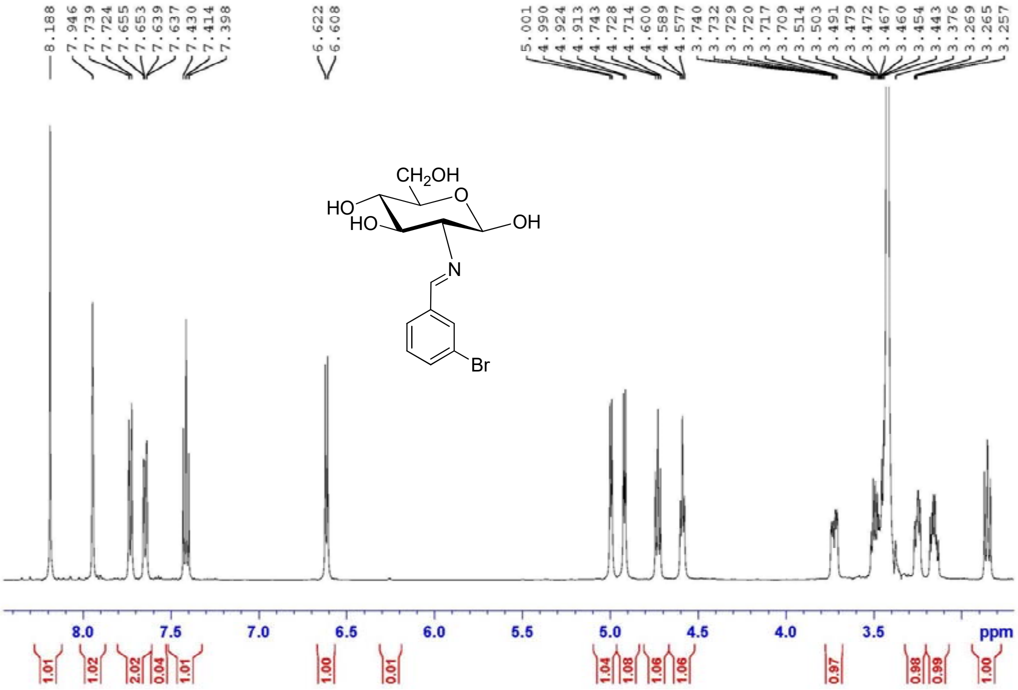

Figure S49. ^{1}H NMR spectrum of **32** in DMSO-d_6.

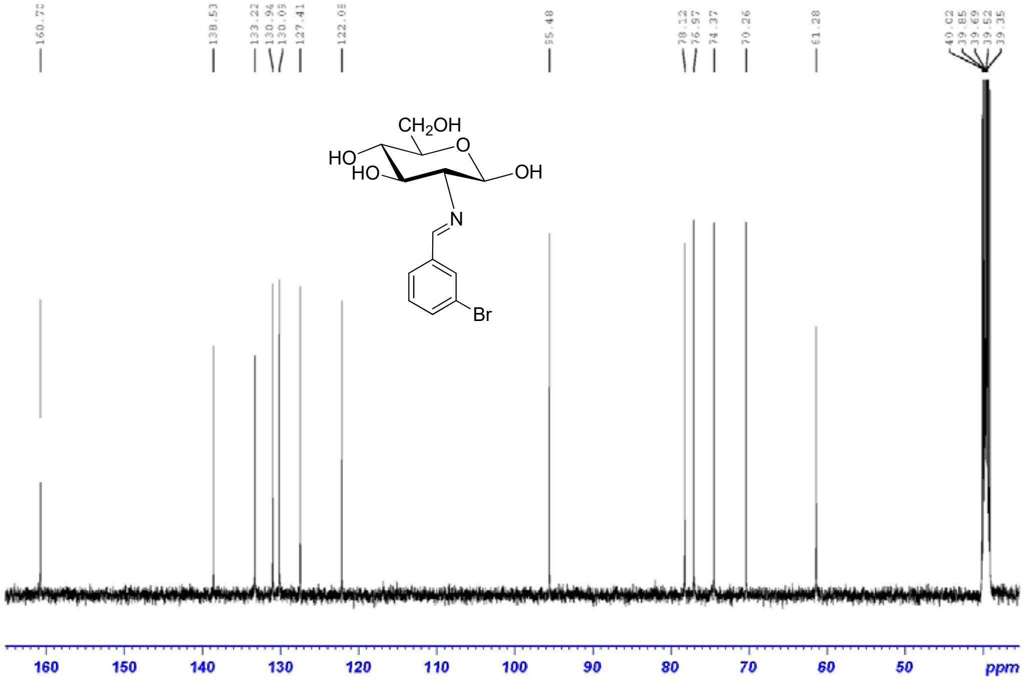

Figure S50. ^{13}C{^{1}H} NMR spectrum of **32** in DMSO-d_6.

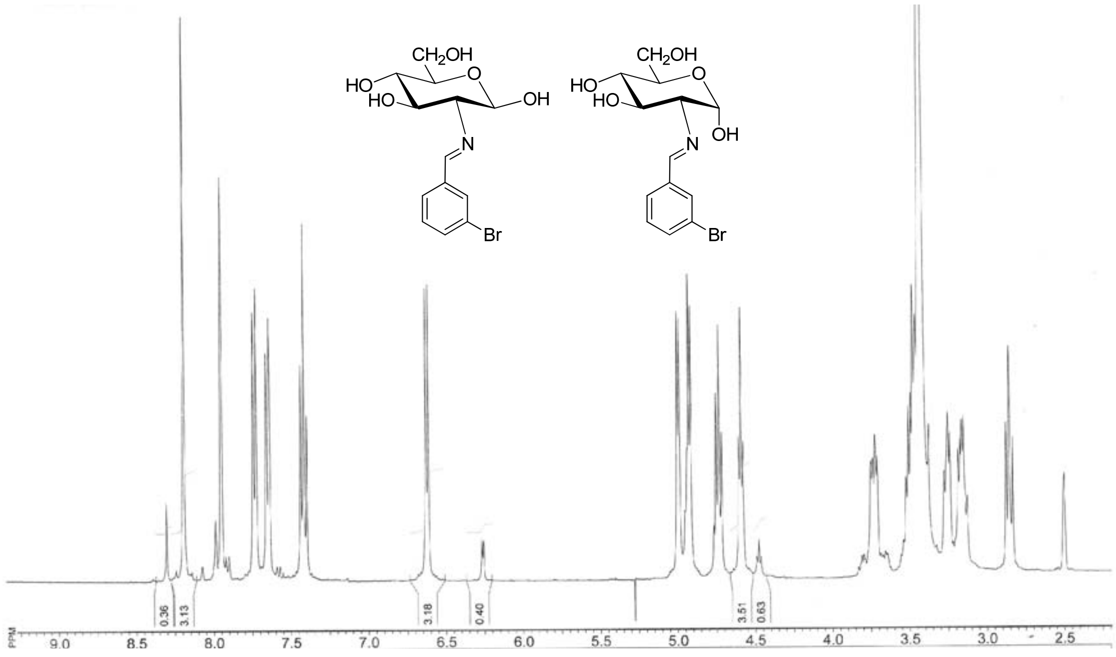

Figure S51. ^{1}H NMR spectrum of **32** and **95** in DMSO-d_6.

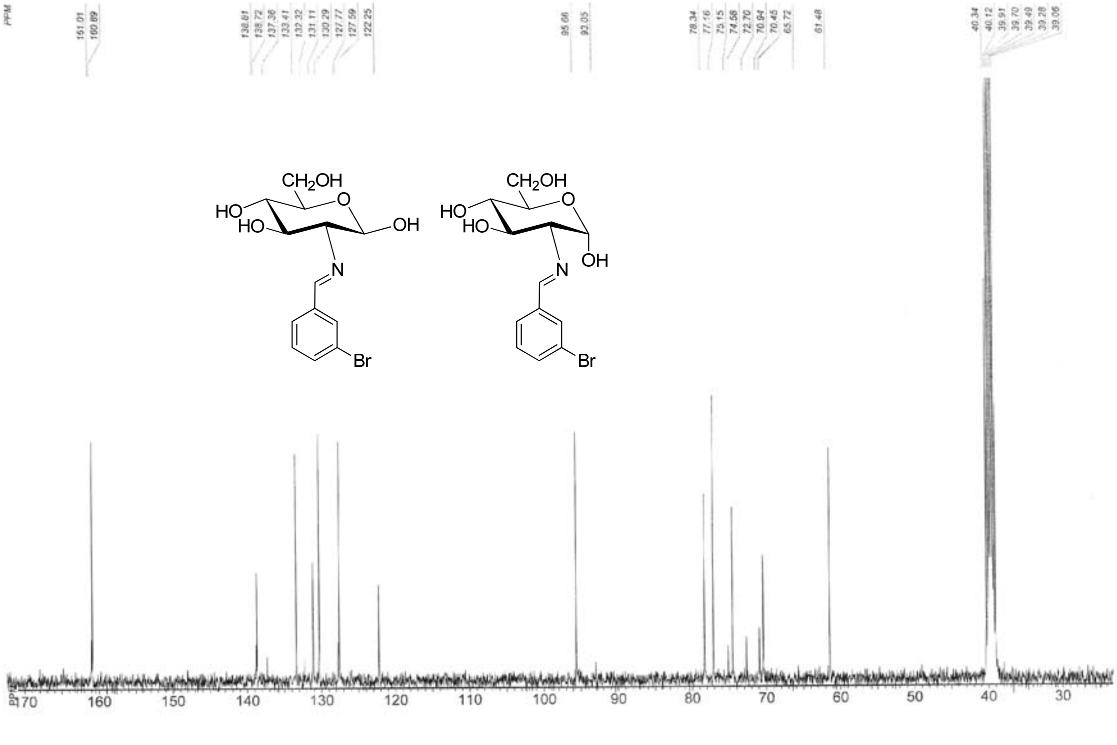

Figure S52. ^{13}C {^{1}H} NMR spectrum of **32** and **95** in DMSO-d_6.

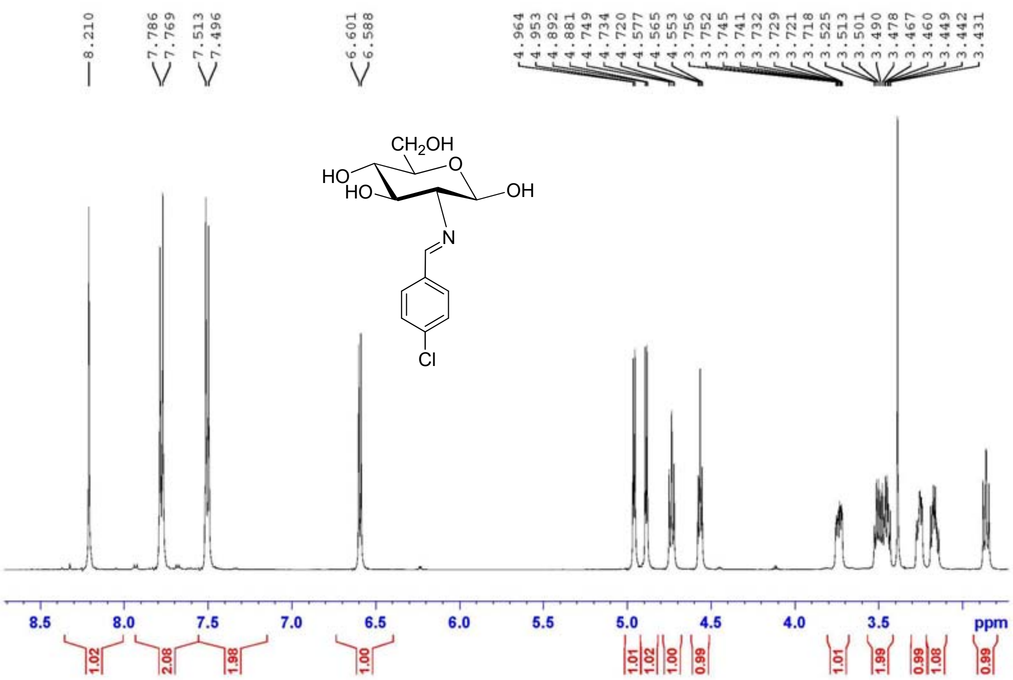

Figure S53. ^{1}H NMR spectrum of **33** in DMSO-*d*6.

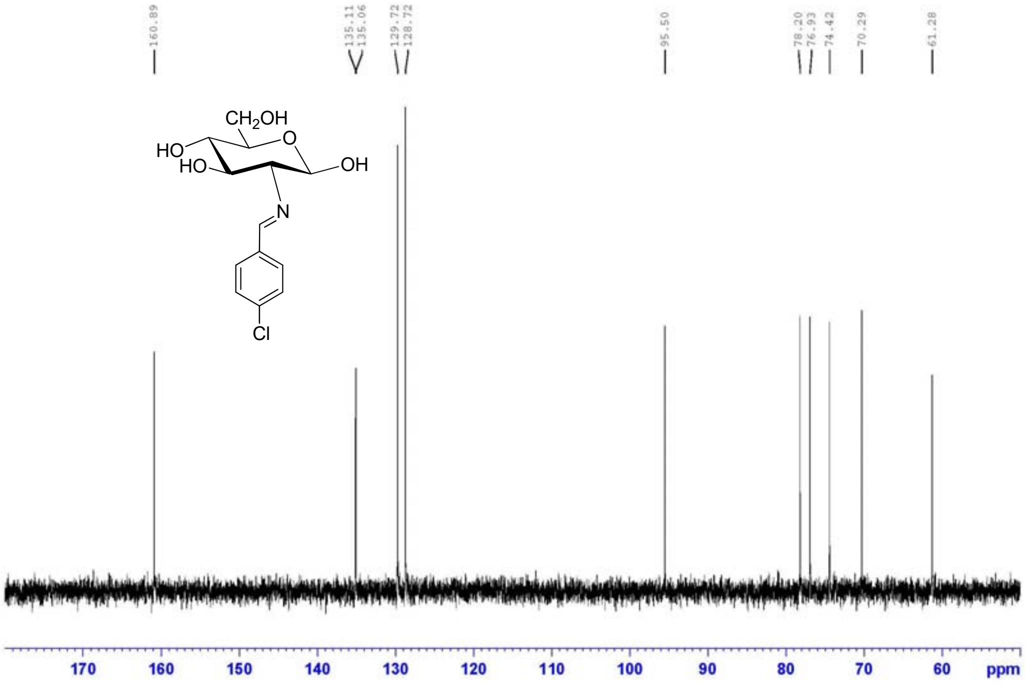

Figure S54. ^{13}C {^{1}H} NMR spectrum of **33** in DMSO-*d*6.

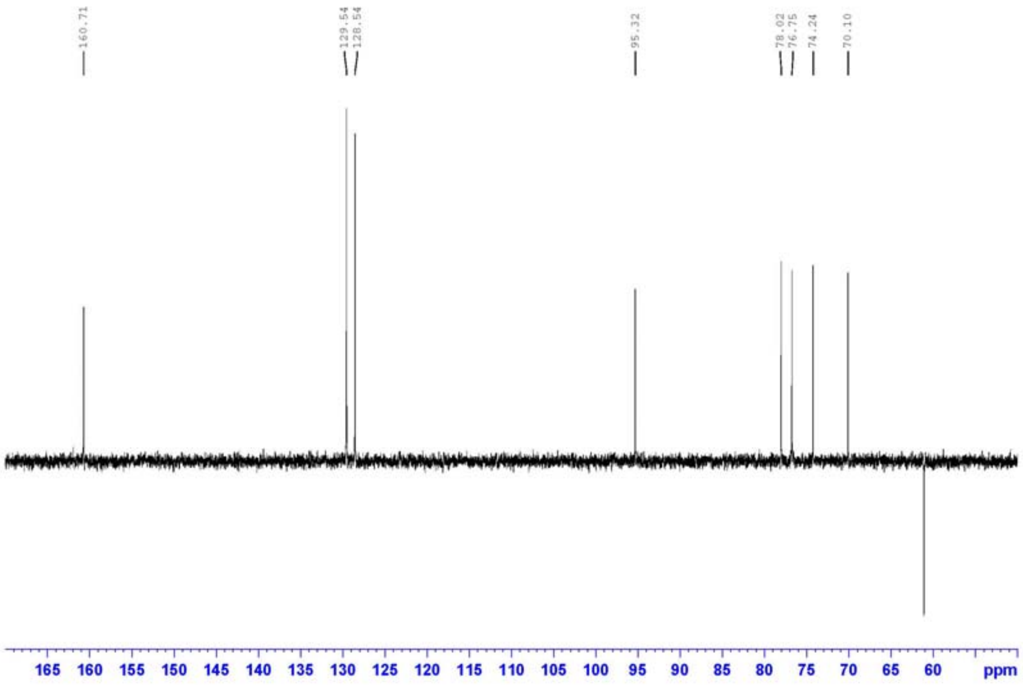

Figure S55. DEPT spectrum of **33** in DMSO-*d*6.

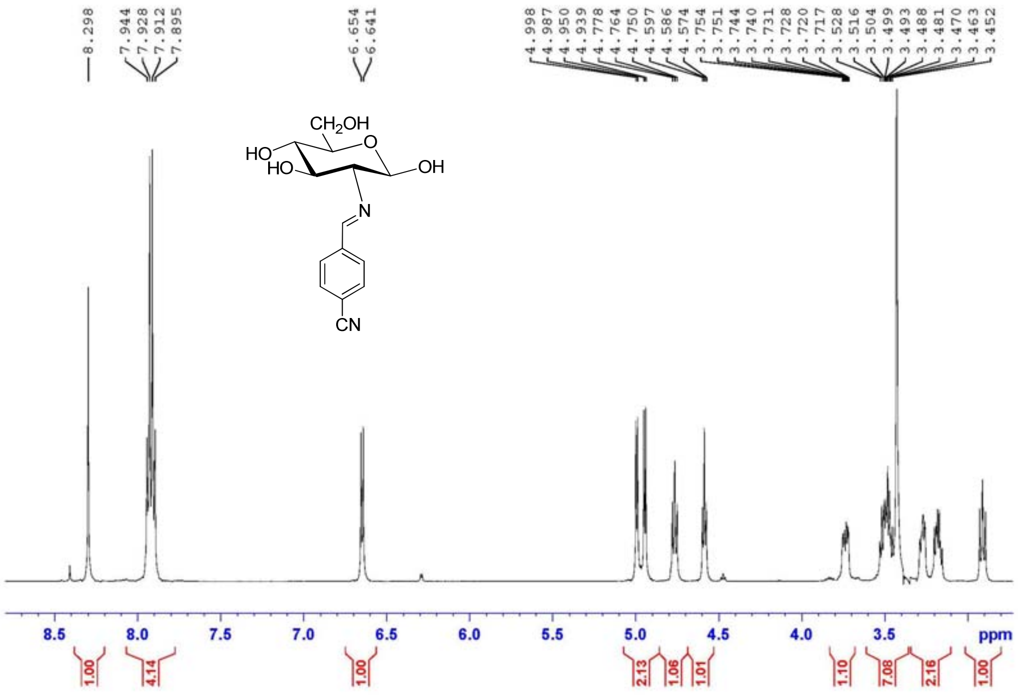

Figure S56. ^{1}H NMR spectrum of **34** in DMSO-*d*6.

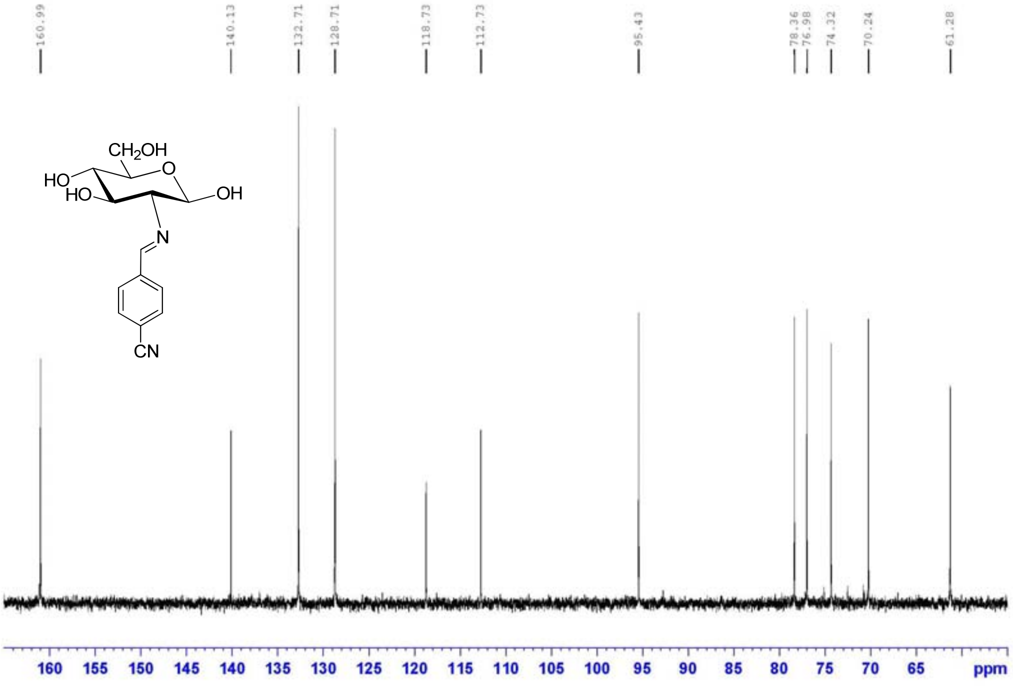

Figure S57. ^{13}C {^{1}H} NMR spectrum of **34** in DMSO-*d*$_6$.

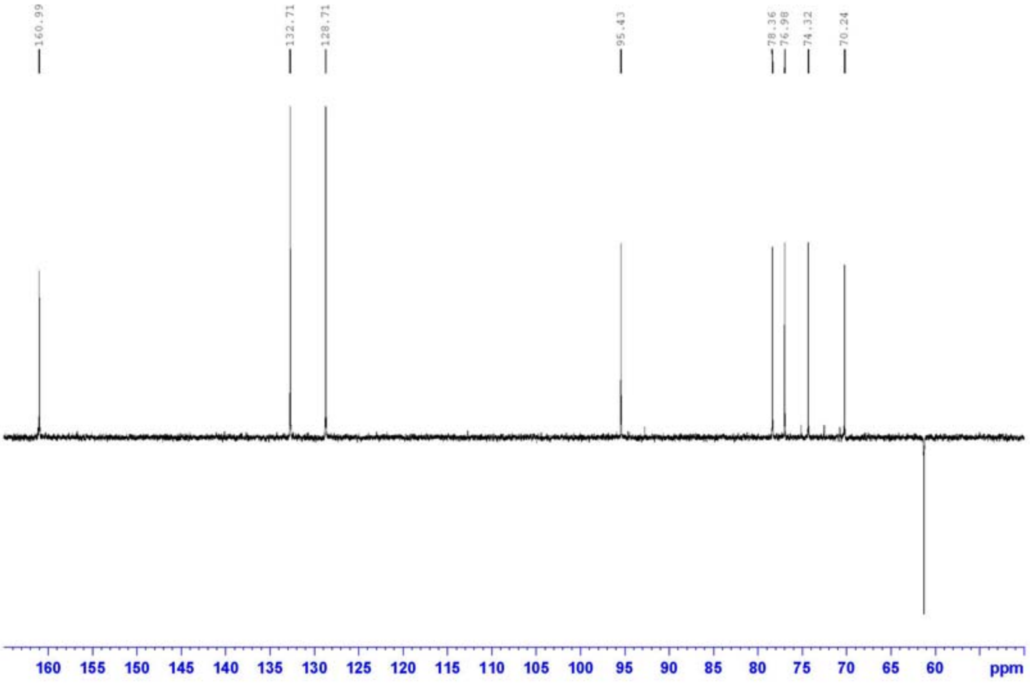

Figure S58. DEPT spectrum of **34** in DMSO-*d*$_6$.

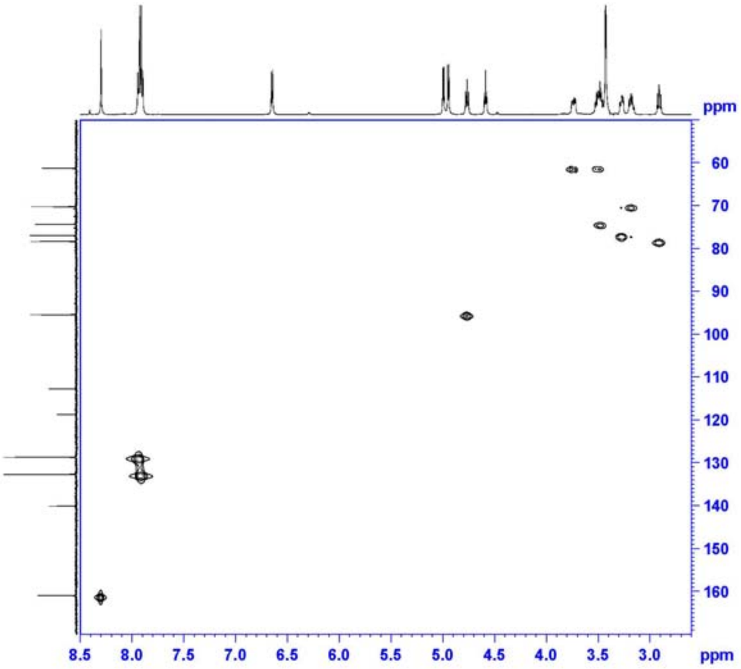

Figure S59. HMQC spectrum of **34** in DMSO-d_6

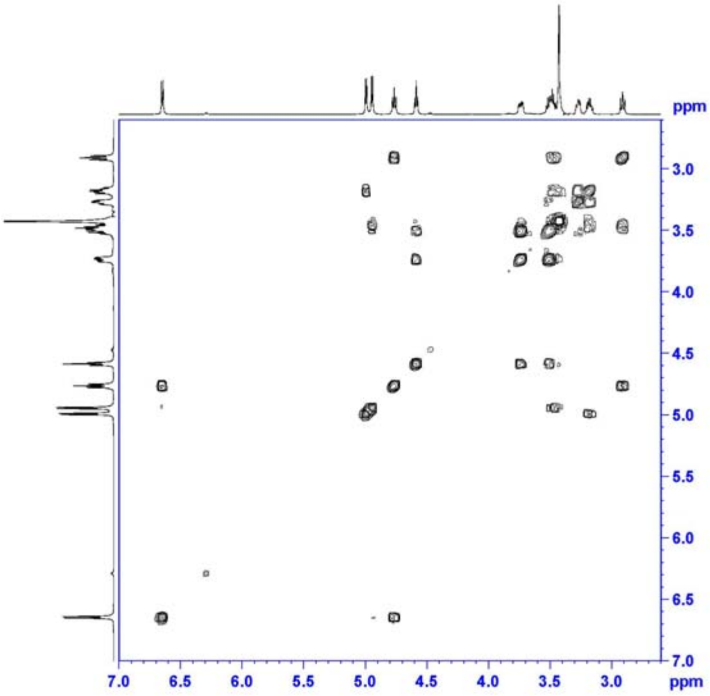

Figure S60. COSY spectrum of **34** in DMSO-d_6

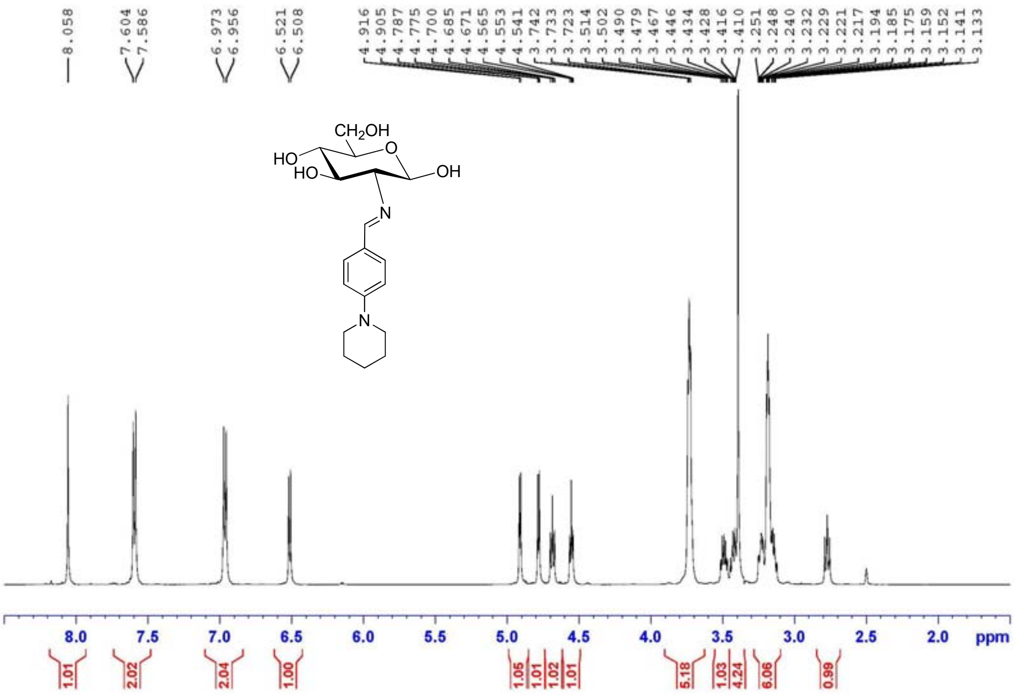

Figure S61. ^{1}H NMR spectrum of **35** in DMSO-*d6*.

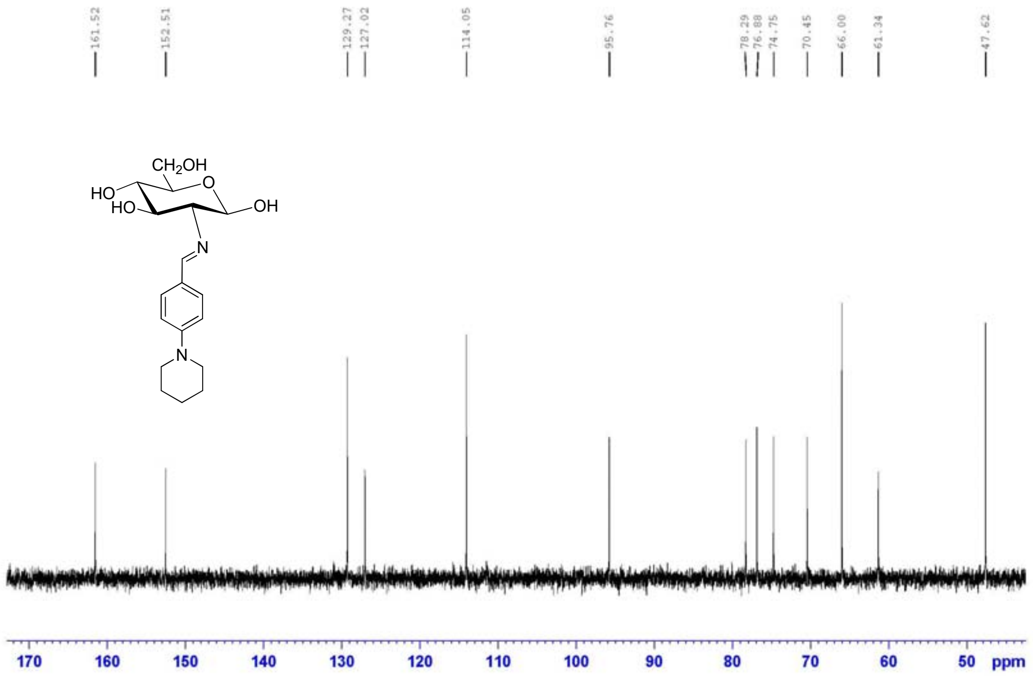

Figure S62. ^{13}C {^{1}H} NMR spectrum of **35** in DMSO-*d6*.

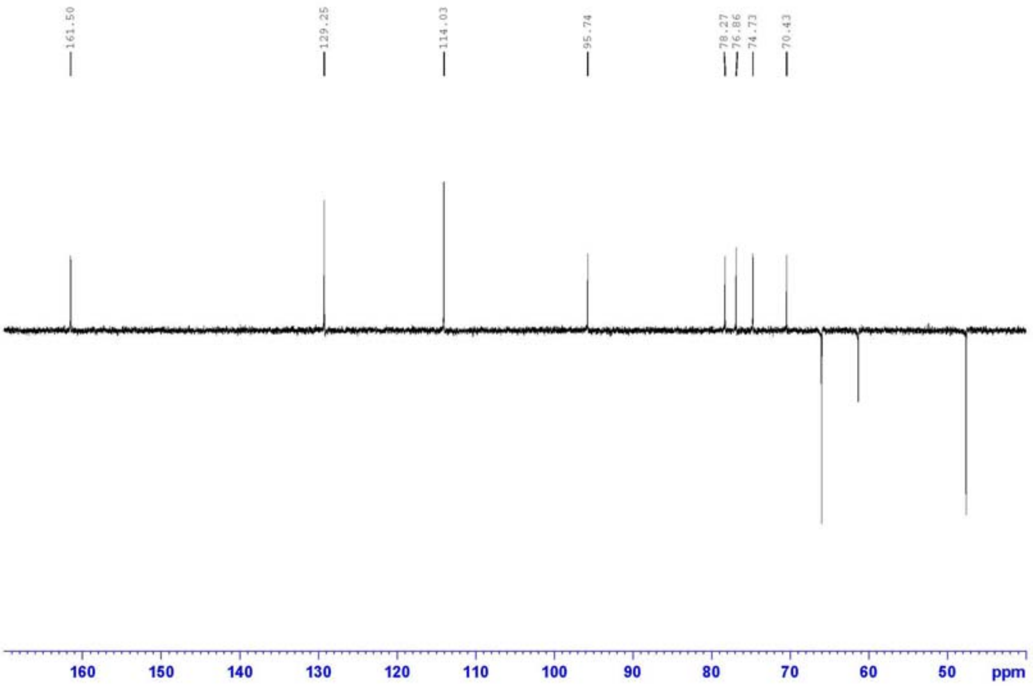

Figure S63. DEPT spectrum of **35** in DMSO-d_6.

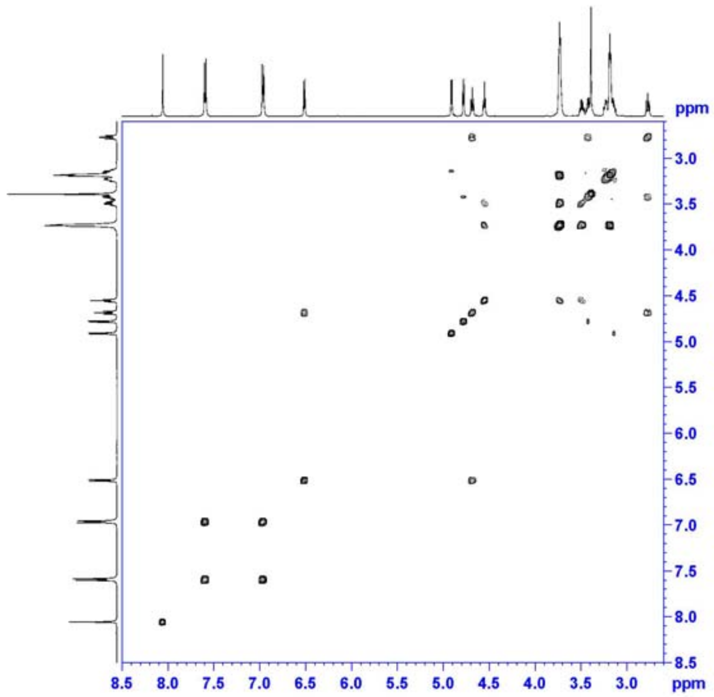

Figure S64. COSY spectrum of **35** in DMSO-d_6

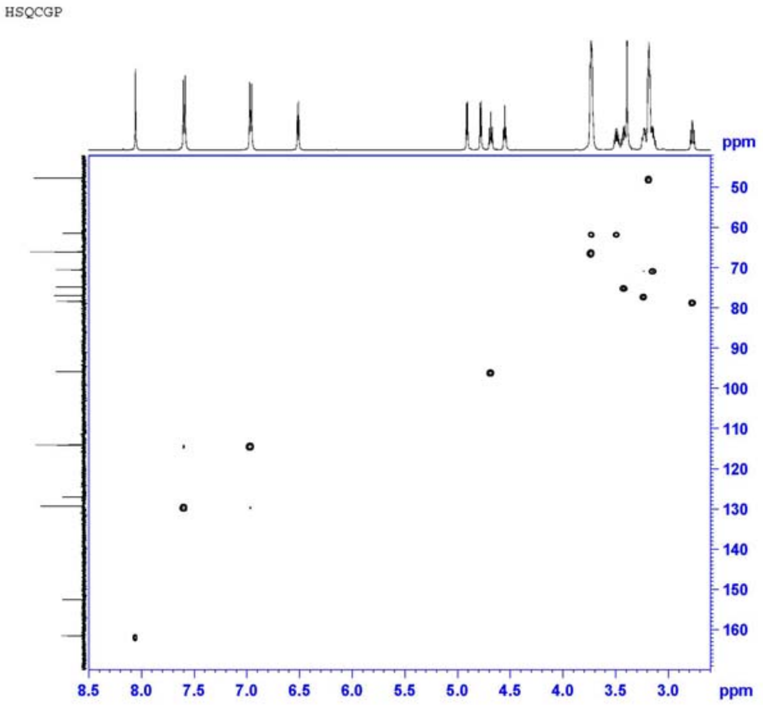

Figure S65. HMQC spectrum of **35** in DMSO-d_6

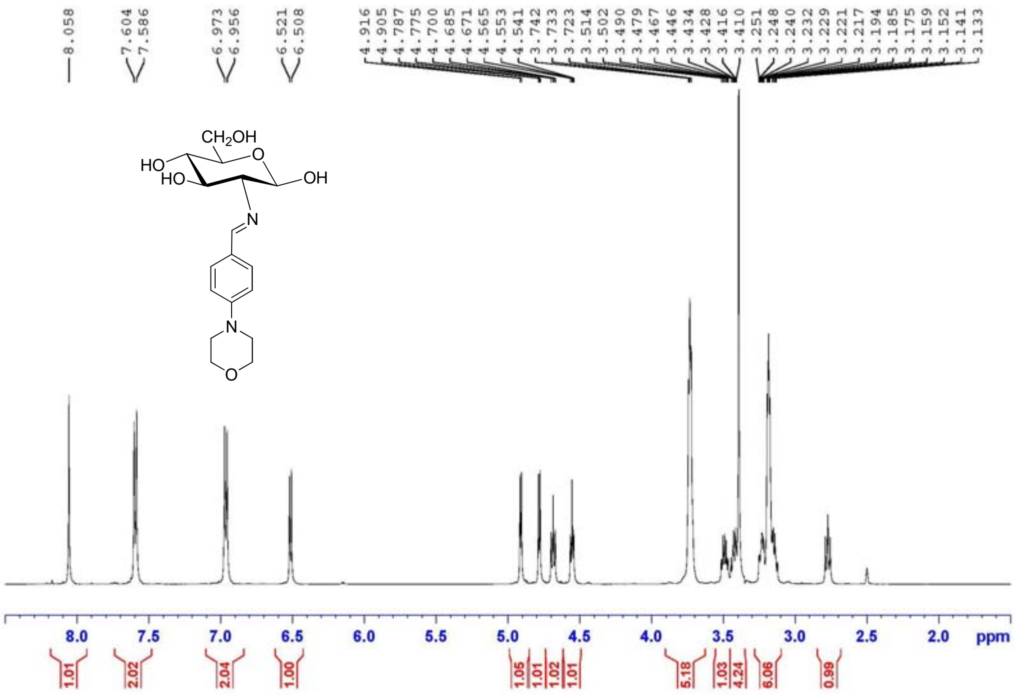

Figure S66. ^{1}H NMR spectrum of **36** in DMSO-d_6

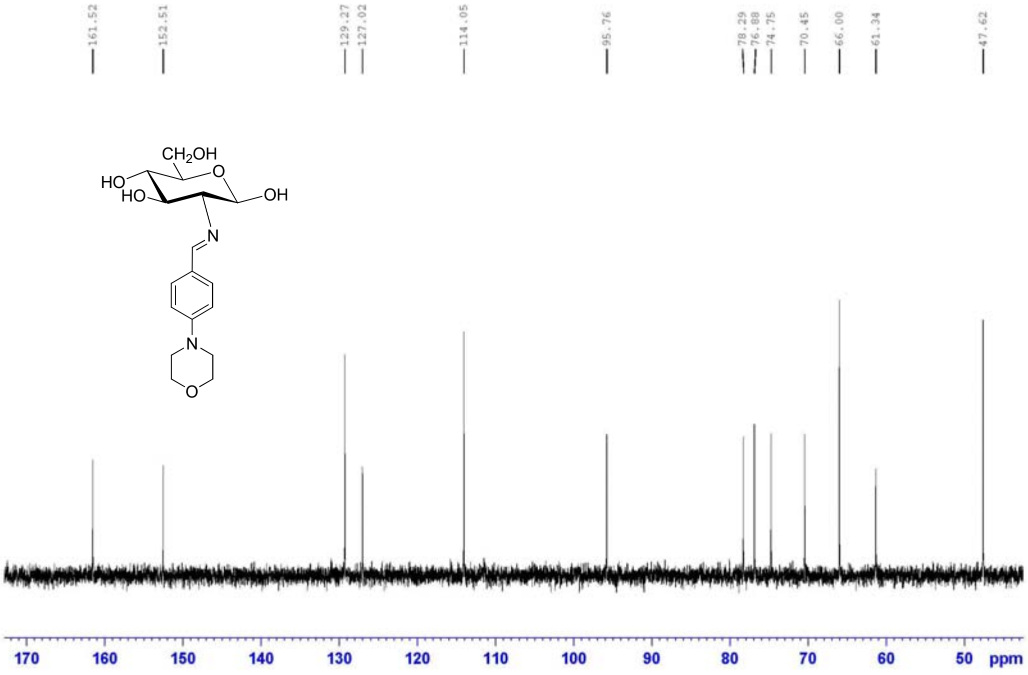

Figure S67. ^{13}C {^{1}H} NMR spectrum of **36** in DMSO-*d₆*

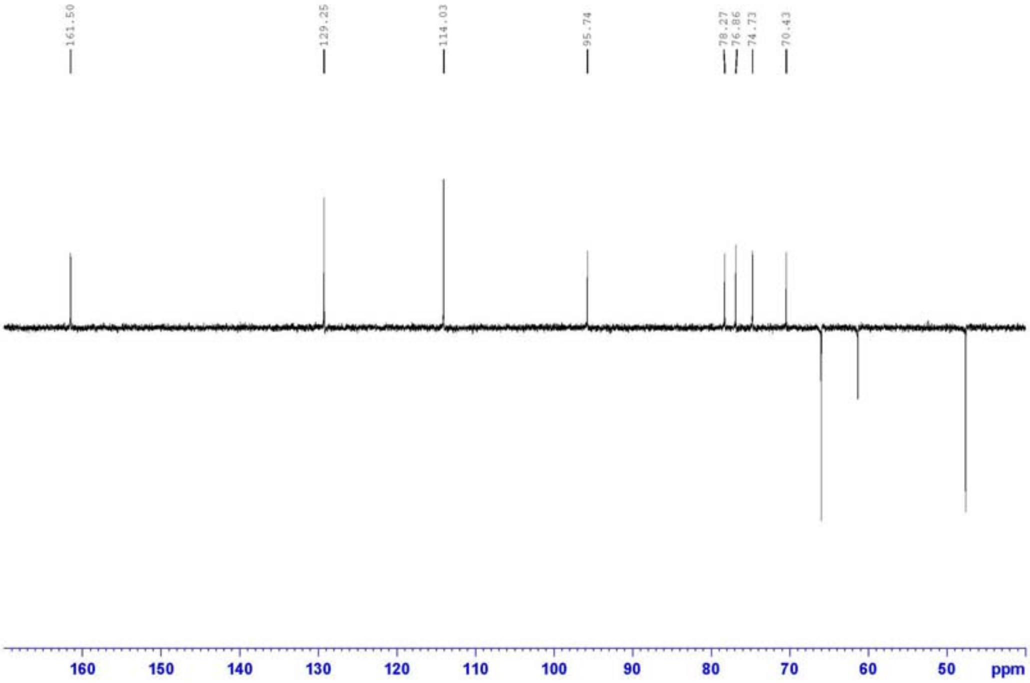

Figure S68. DEPT spectrum of **36** in DMSO-d_6

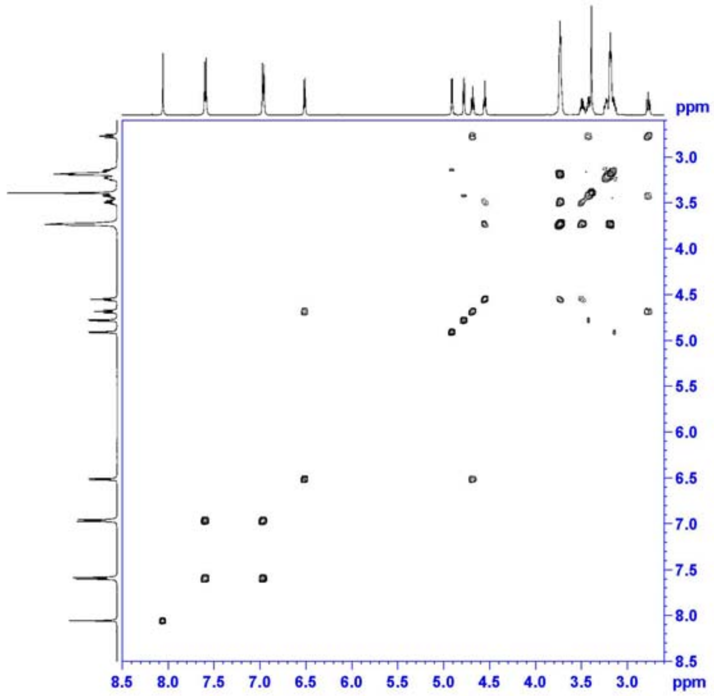

Figure S69. COSY spectrum of **35** in DMSO-d_6

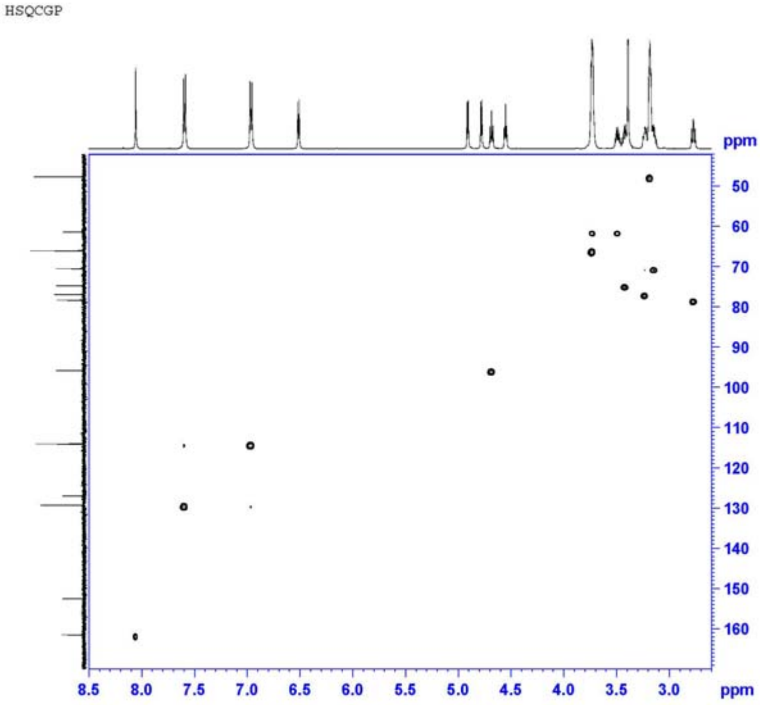

Figure S70. HSQC spectrum of **35** in DMSO-d_6

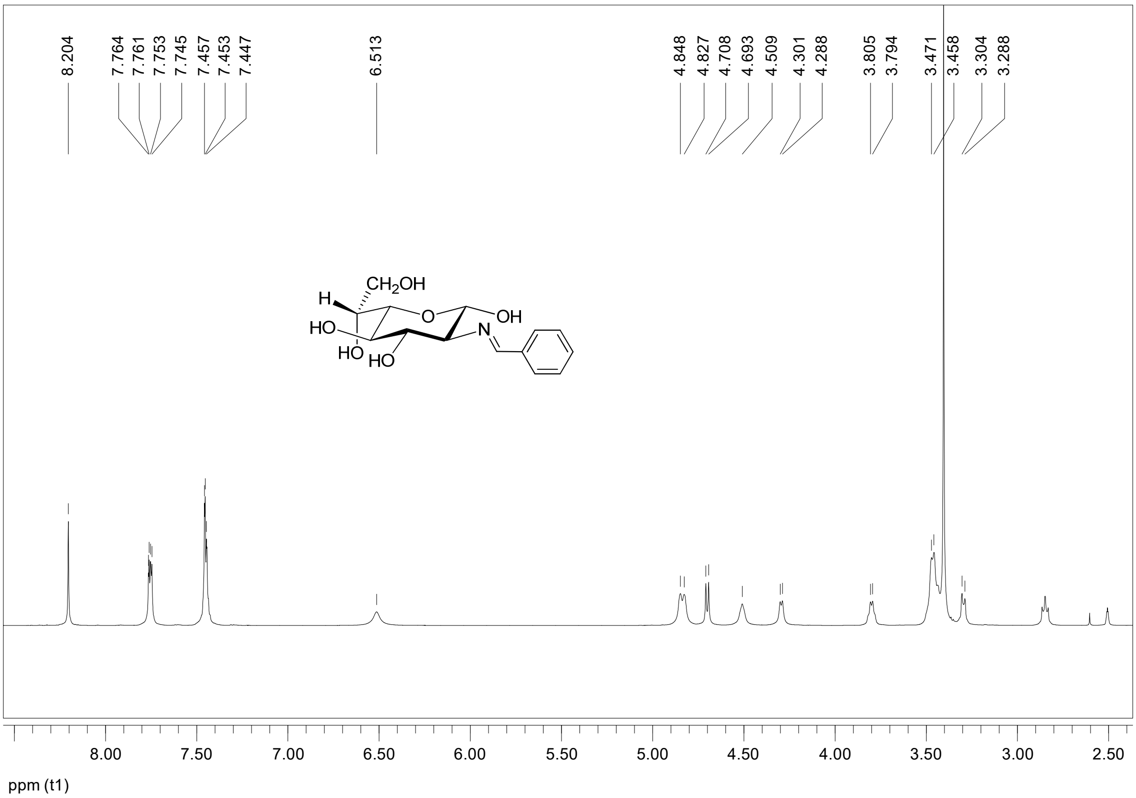

Figure S71. ^{1}H NMR spectrum of **38** in DMSO-d_6

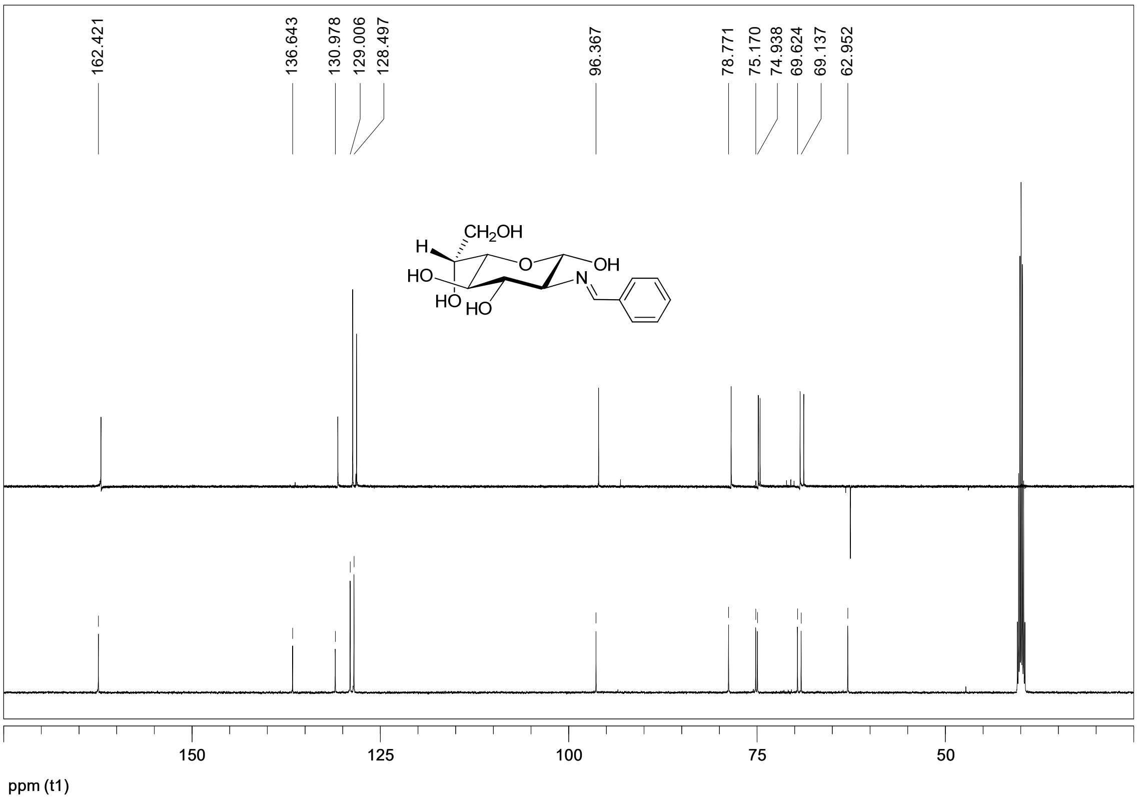

Figure S72. ^{13}C{^{1}H} NMR (top: DEPT) spectra of **38** in DMSO-d_6

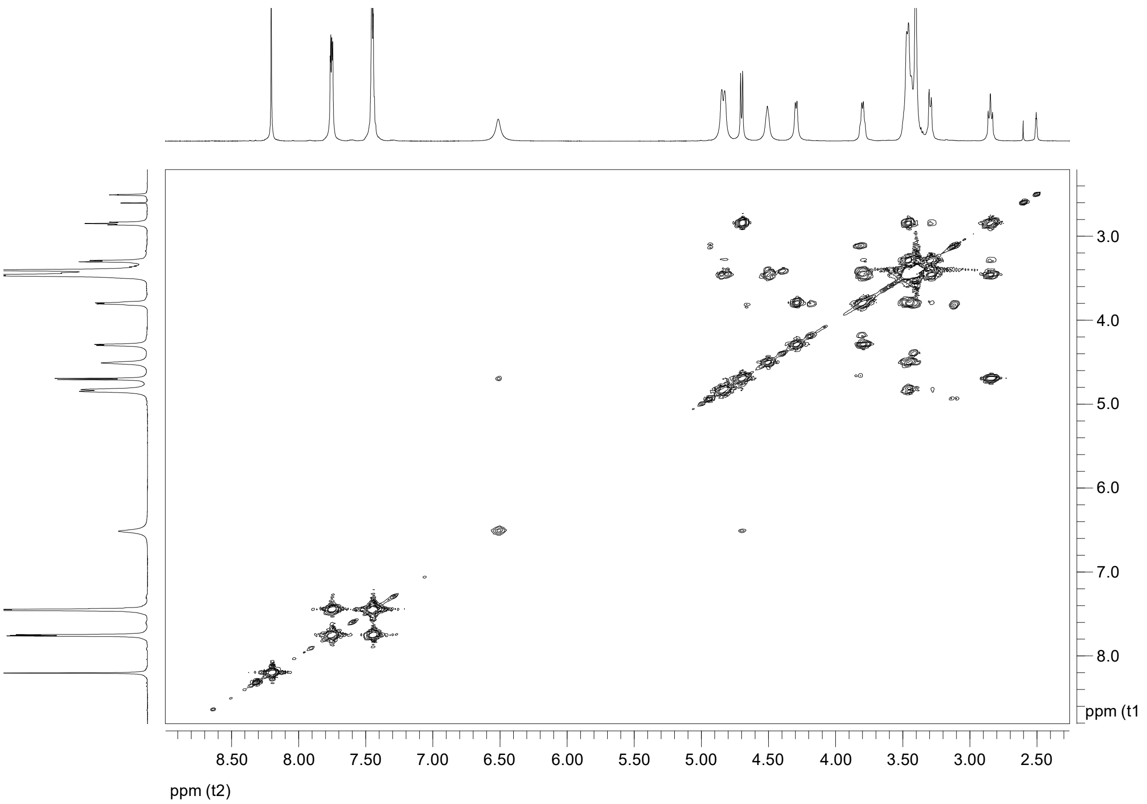

Figure S73. COSY spectrum of **38** in DMSO-d_6

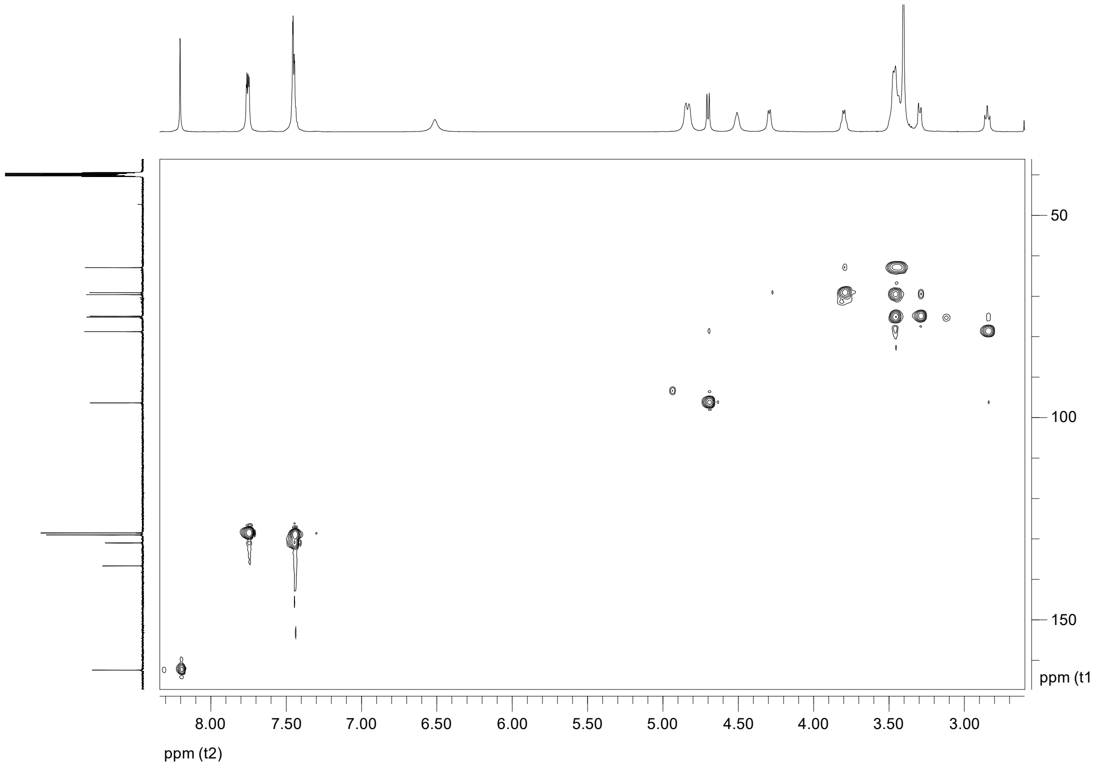

Figure S74. HMQC spectrum of **38** inDMSO-d_6

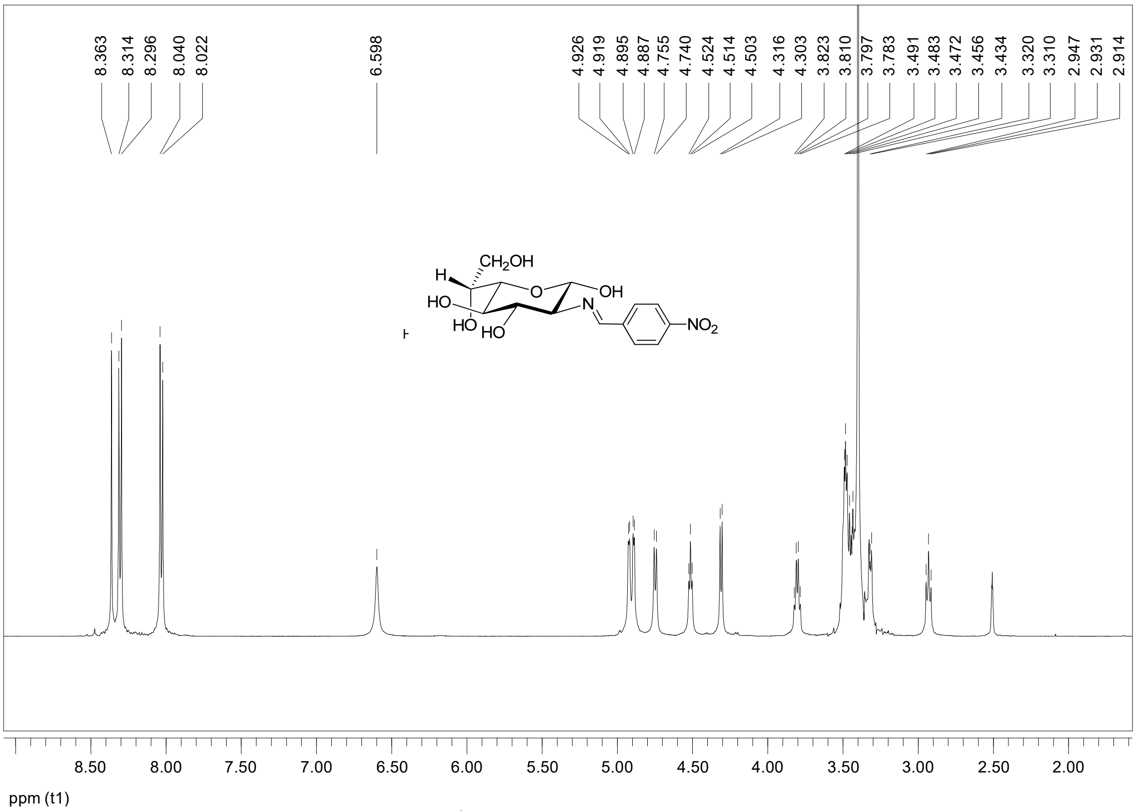

Figure S75. ^{1}H NMR spectrum of **39** in DMSO-d_6

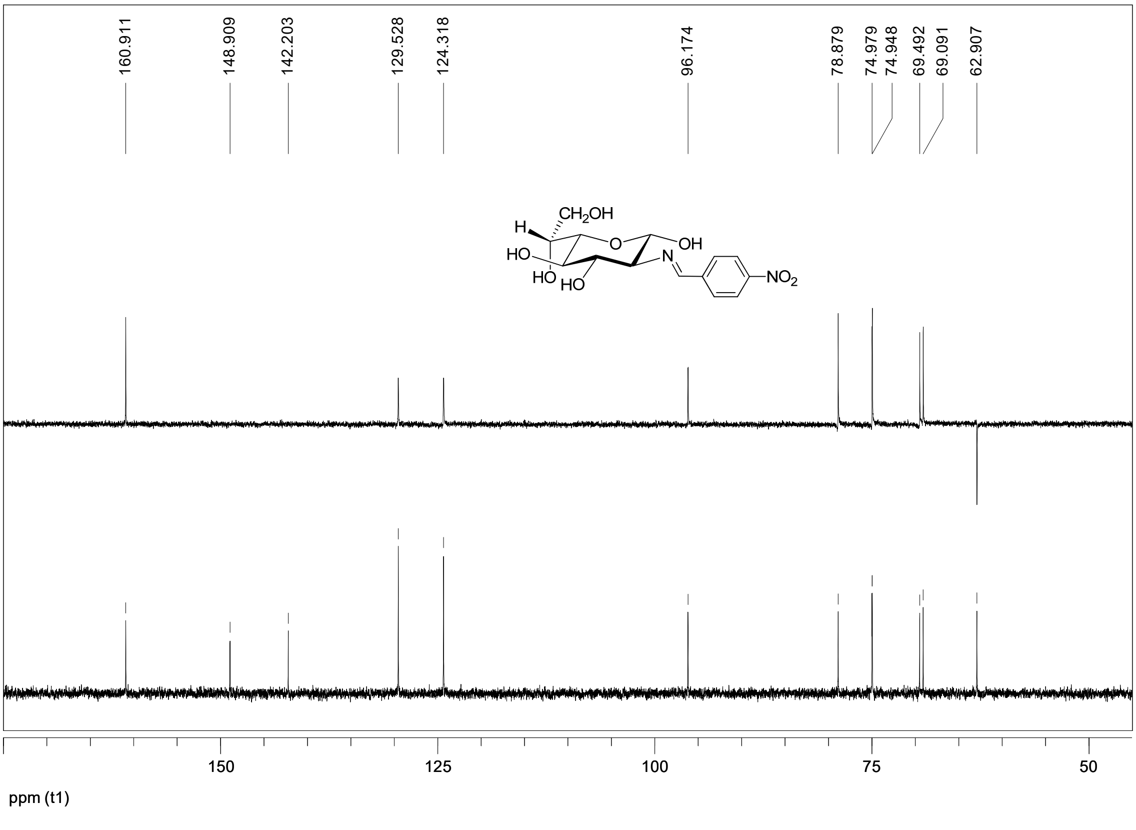

Figure S76. $^{13}C\{^1H\}$ NMR (top: DEPT) spectra of **39** in DMSO-d_6

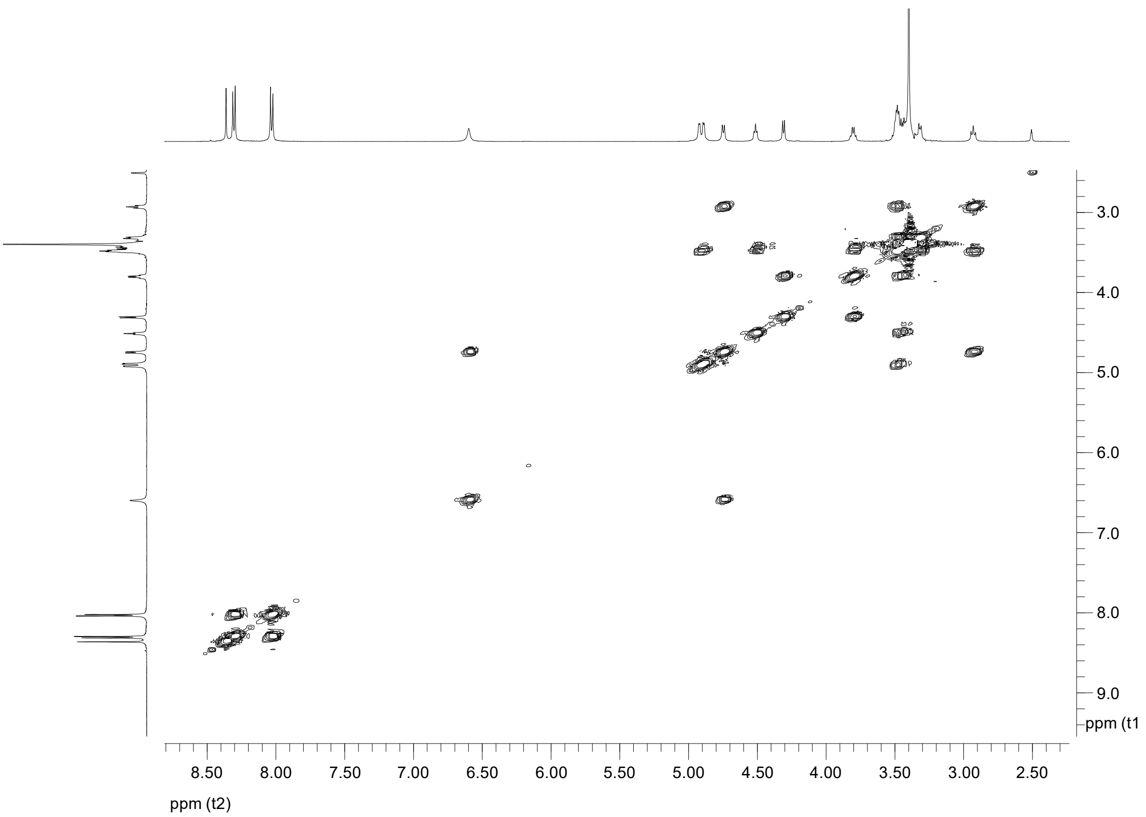

Figure S77. COSY spectrum of **39** in DMSO-d_6

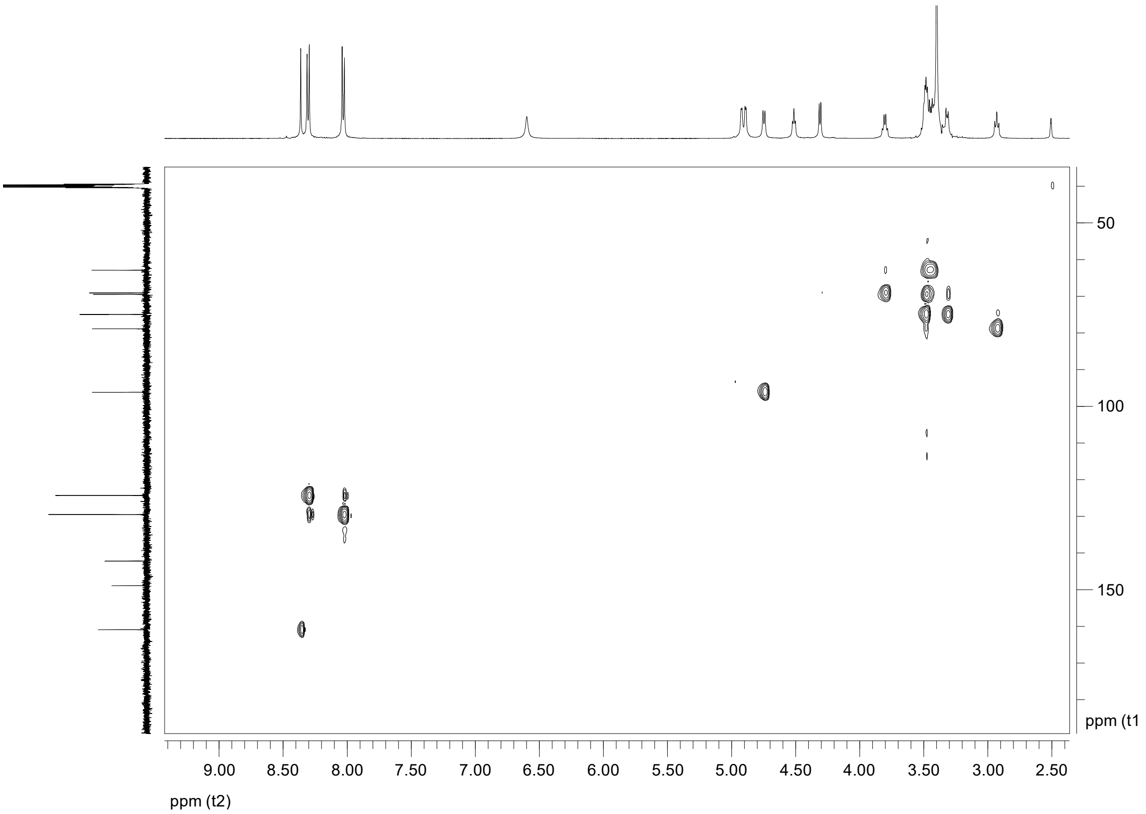

Figure S78. HMQC spectrum of **39** in DMSO-d_6

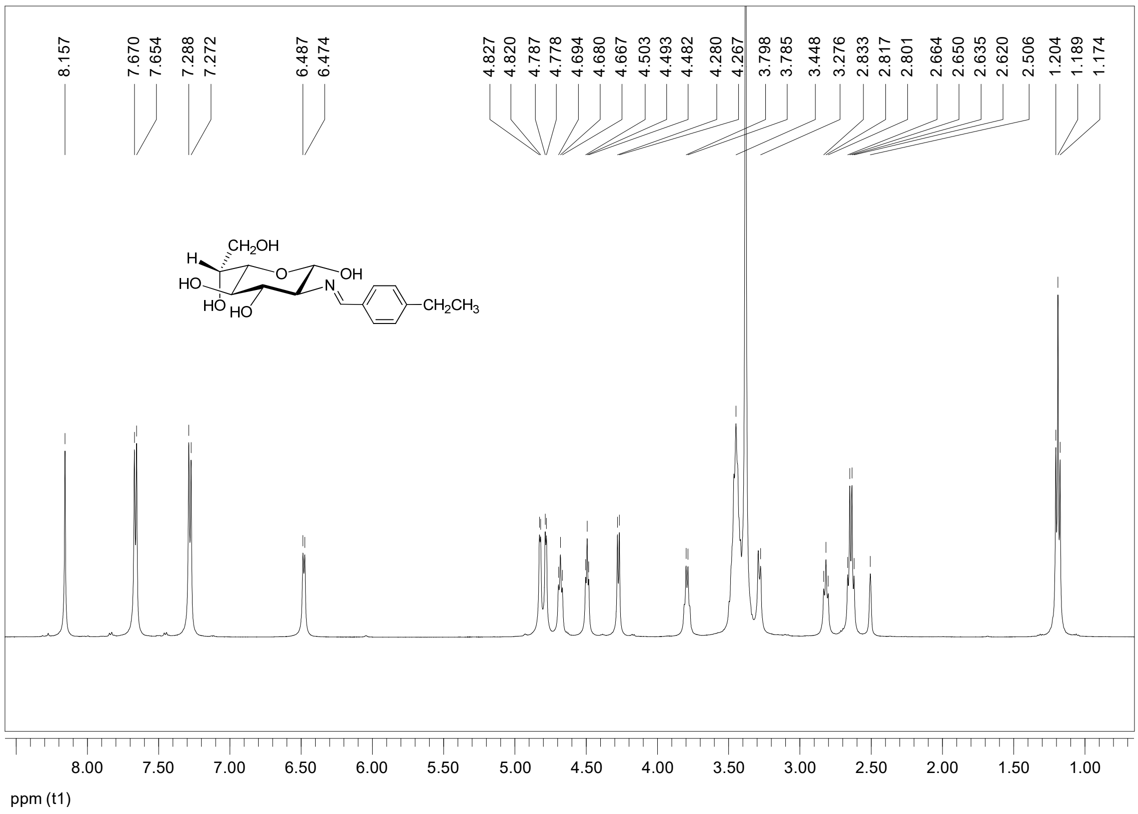

Figure S79. ^{1}H NMR spectrum of **40** in DMSO-*d*$_6$

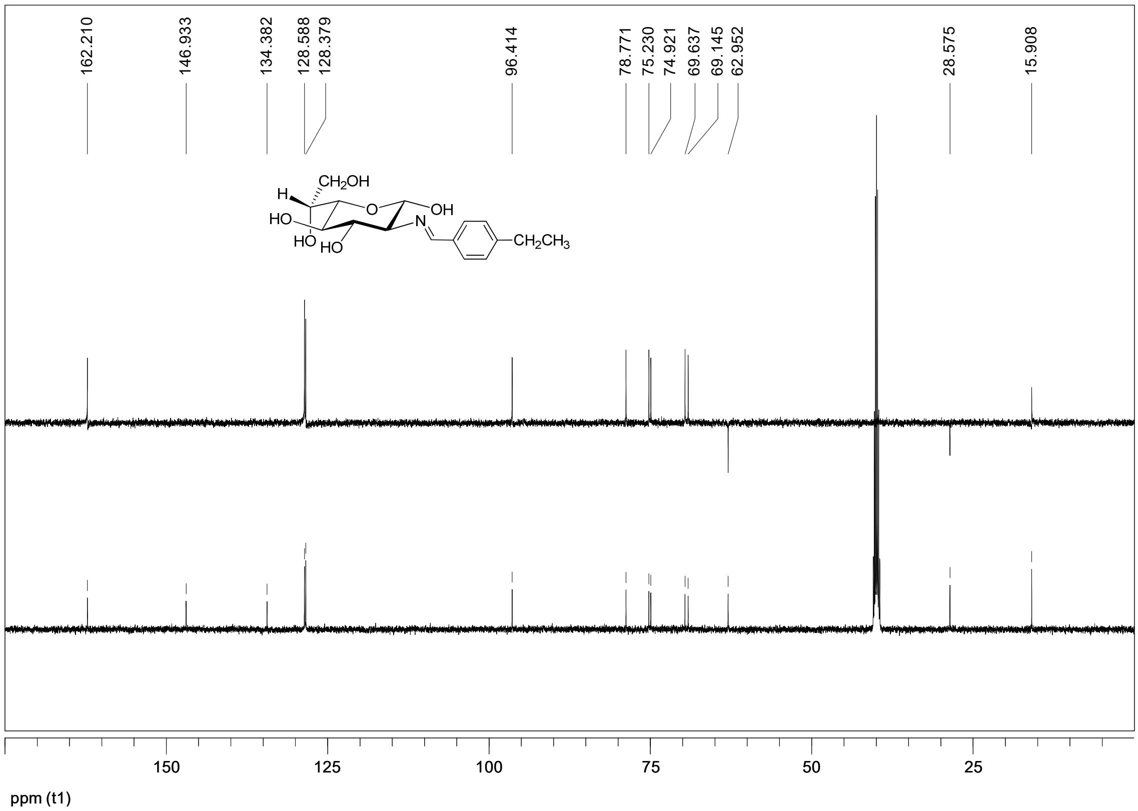

Figure S80. $^{13}C\{^{1}H\}$ NMR (top: DEPT) spectra of **40** in DMSO-*d*$_6$

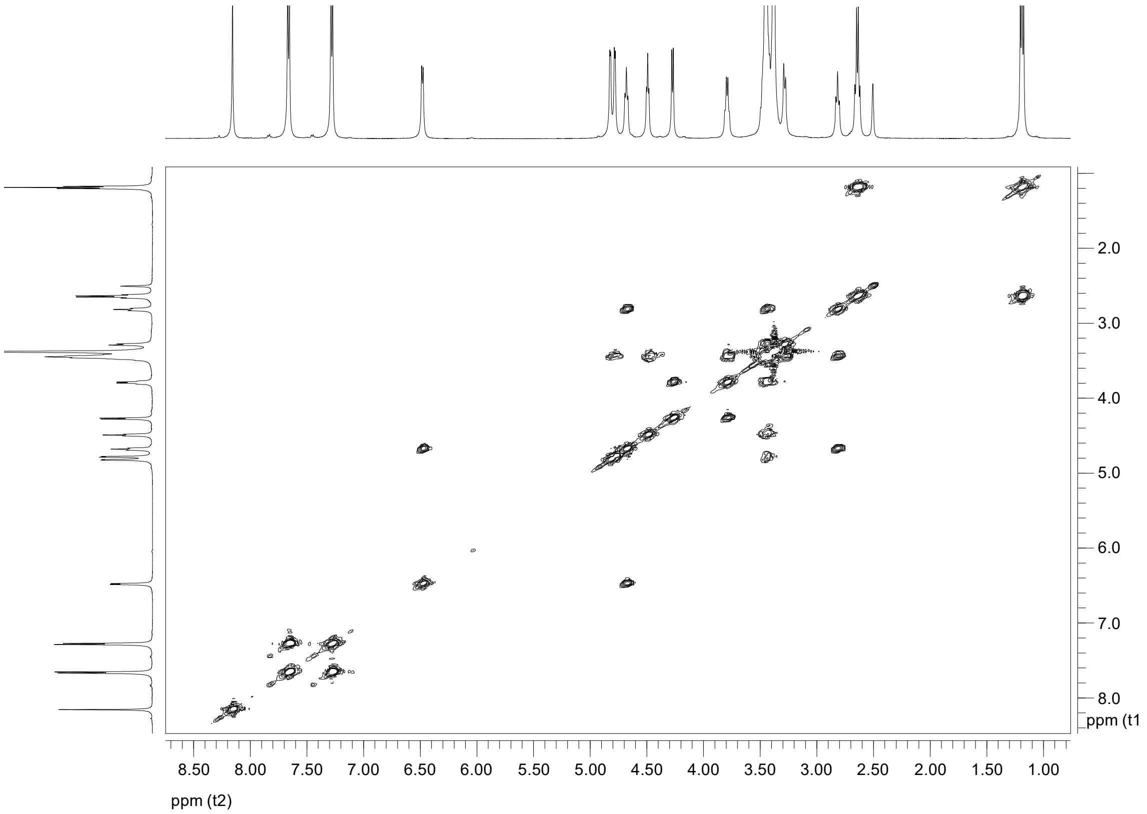

Figure S81. COSY spectrum of **40** DMSO-d_6

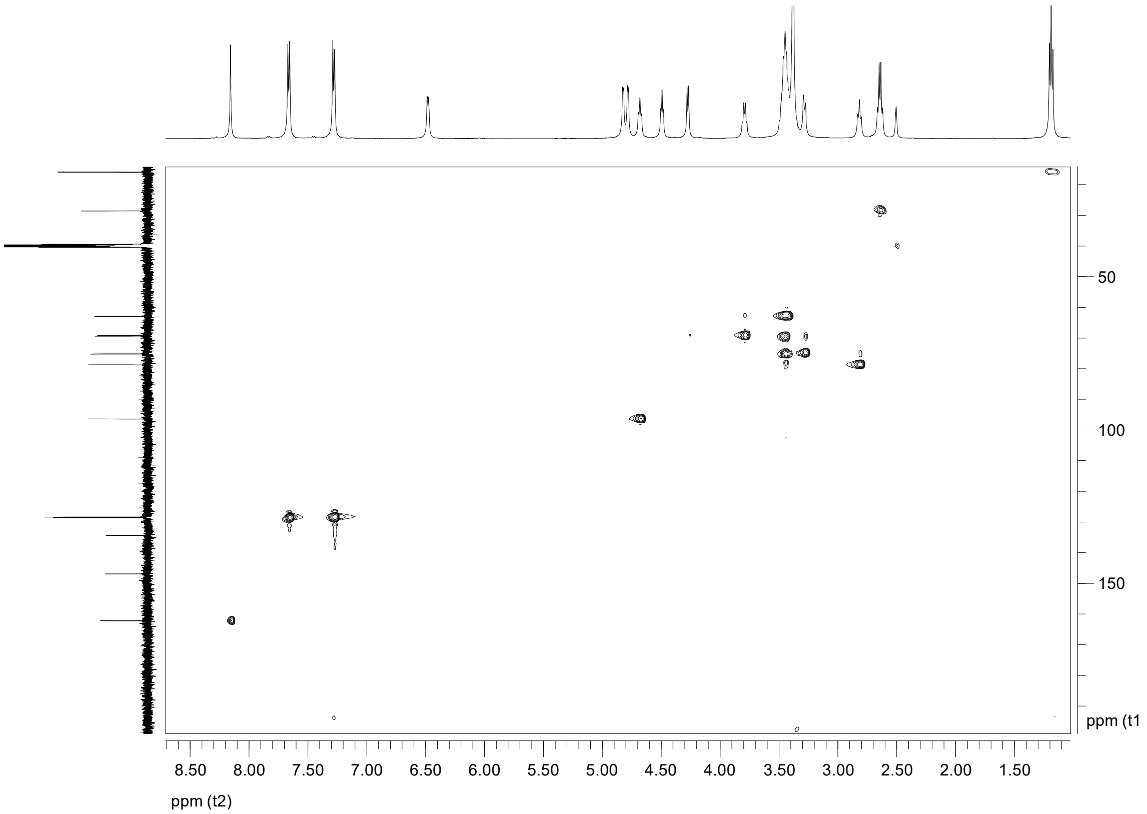

Figure S82. HMQC spectrum of **40** DMSO-d_6

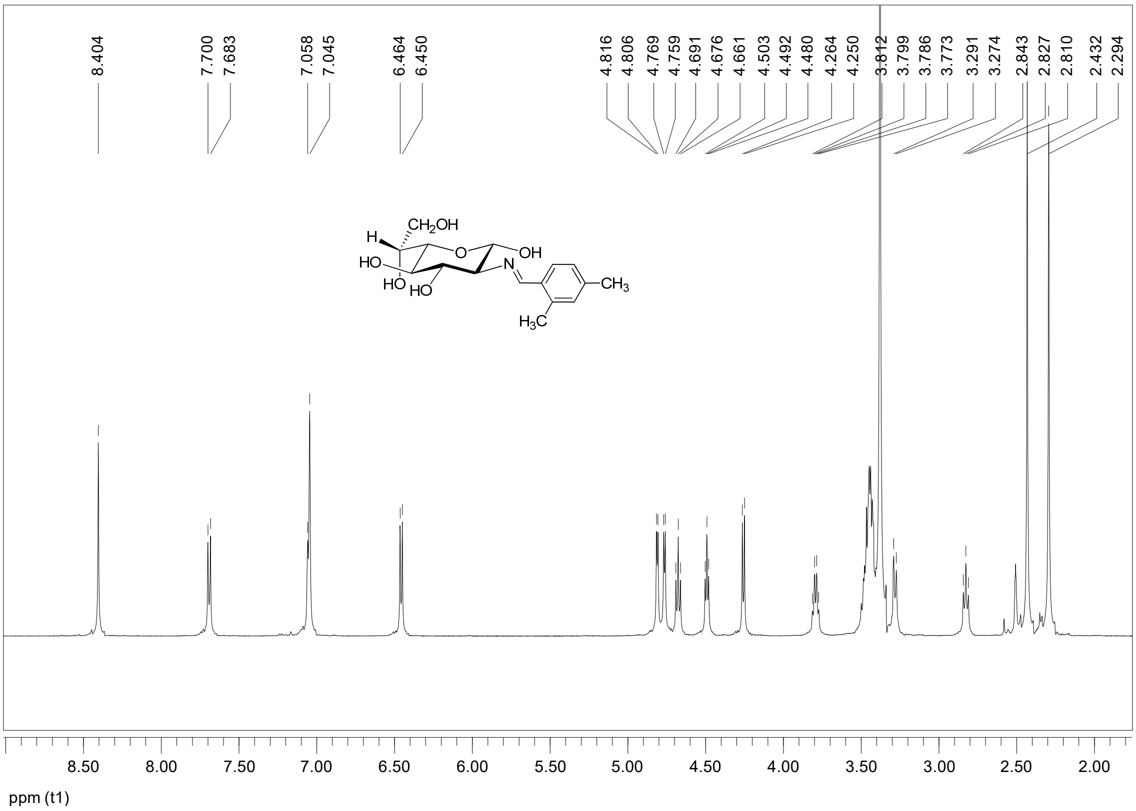

Figure S83. ^{1}H NMR spectrum of **41** in DMSO-d_6

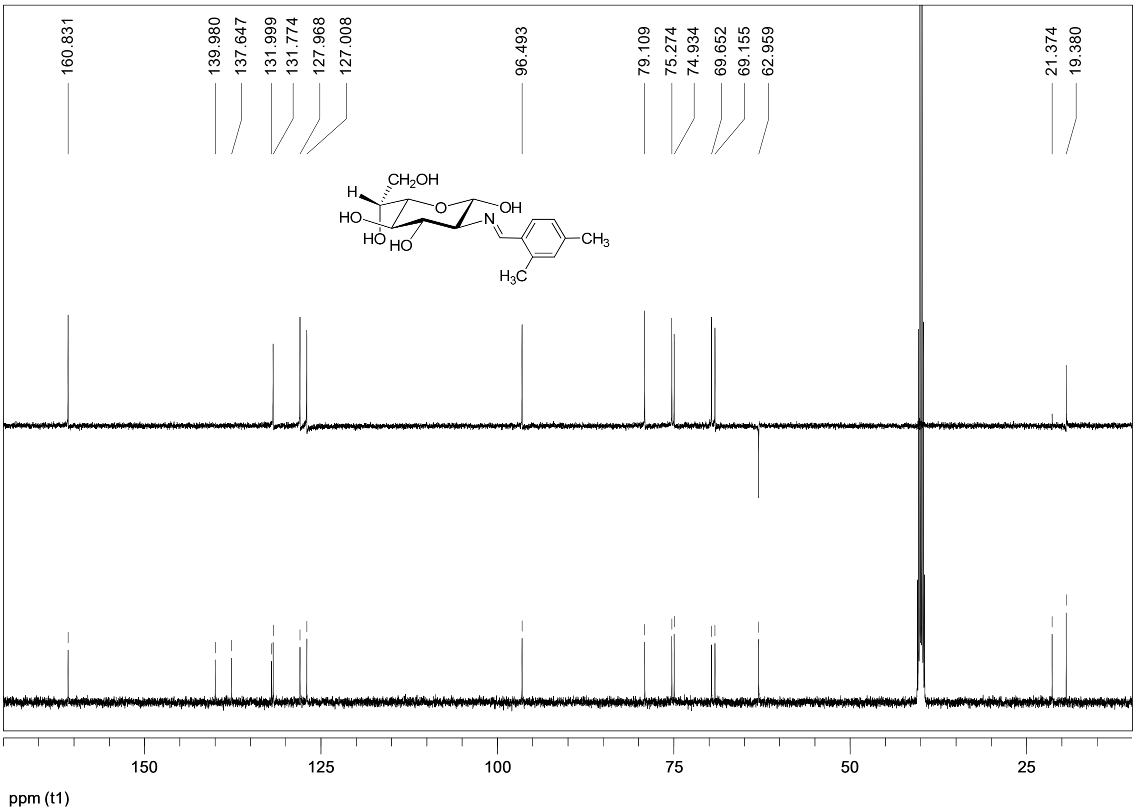

Figure S84. ^{13}C{^{1}H} NMR (top: DEPT) spectra of **41** in DMSO-d_6

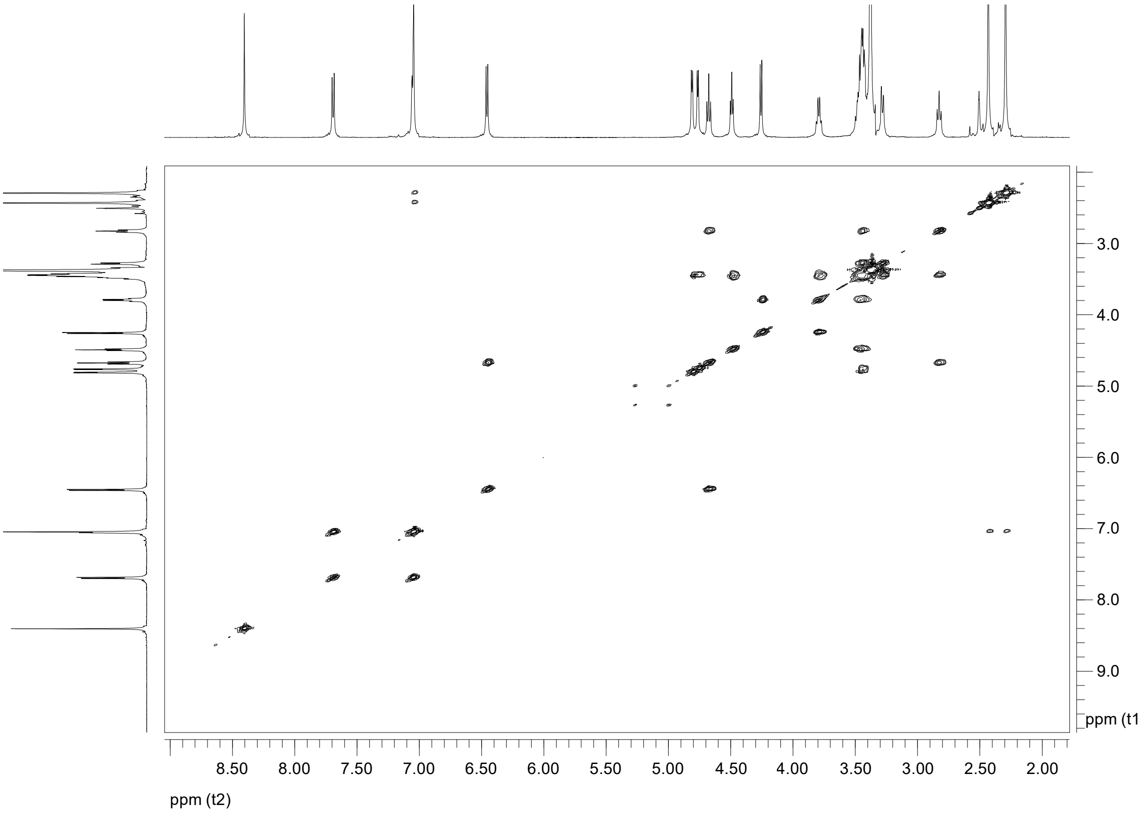

Figure S85. COSY spectrum of **41** in DMSO-d_6

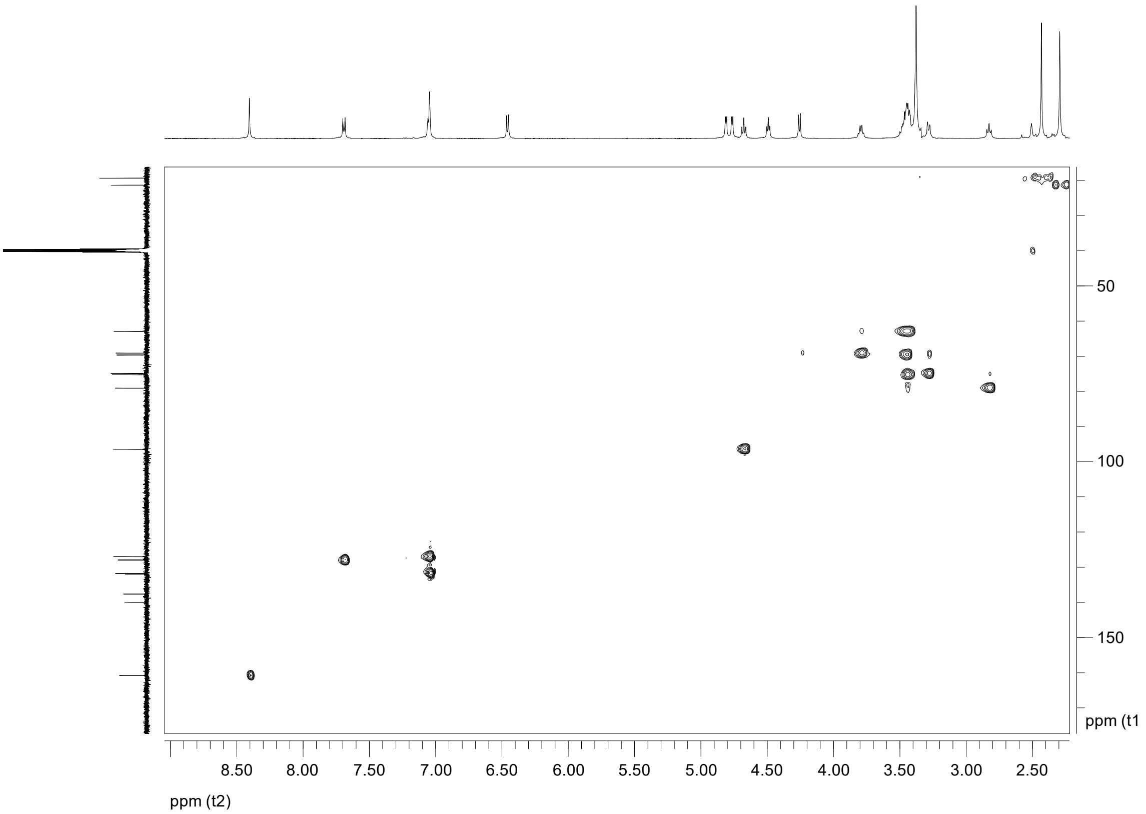

Figure S86. HMQC spectrum of **41** in DMSO-d_6

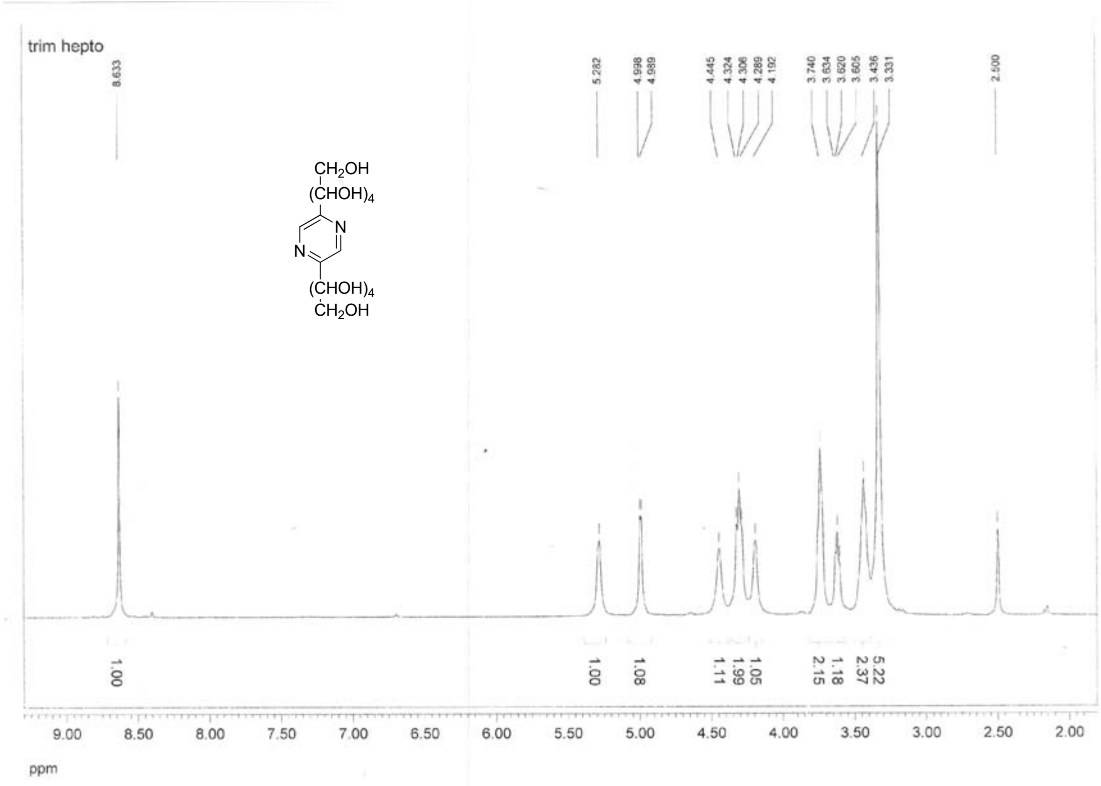

Figure S87. ^{1}H NMR spectrum of **43** in DMSO-*d*$_6$.

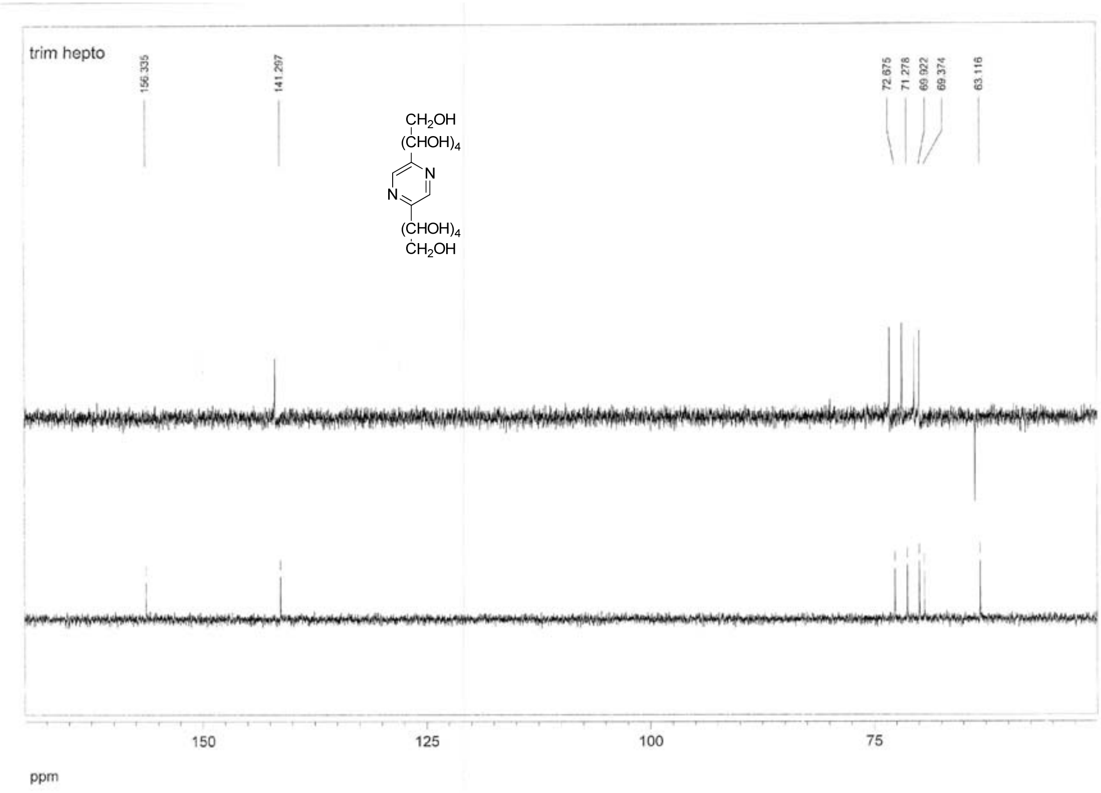

Figure S88. $^{13}C\{^{1}H\}$ NMR (top: DEPT) spectra of **43** in DMSO-*d*$_6$.

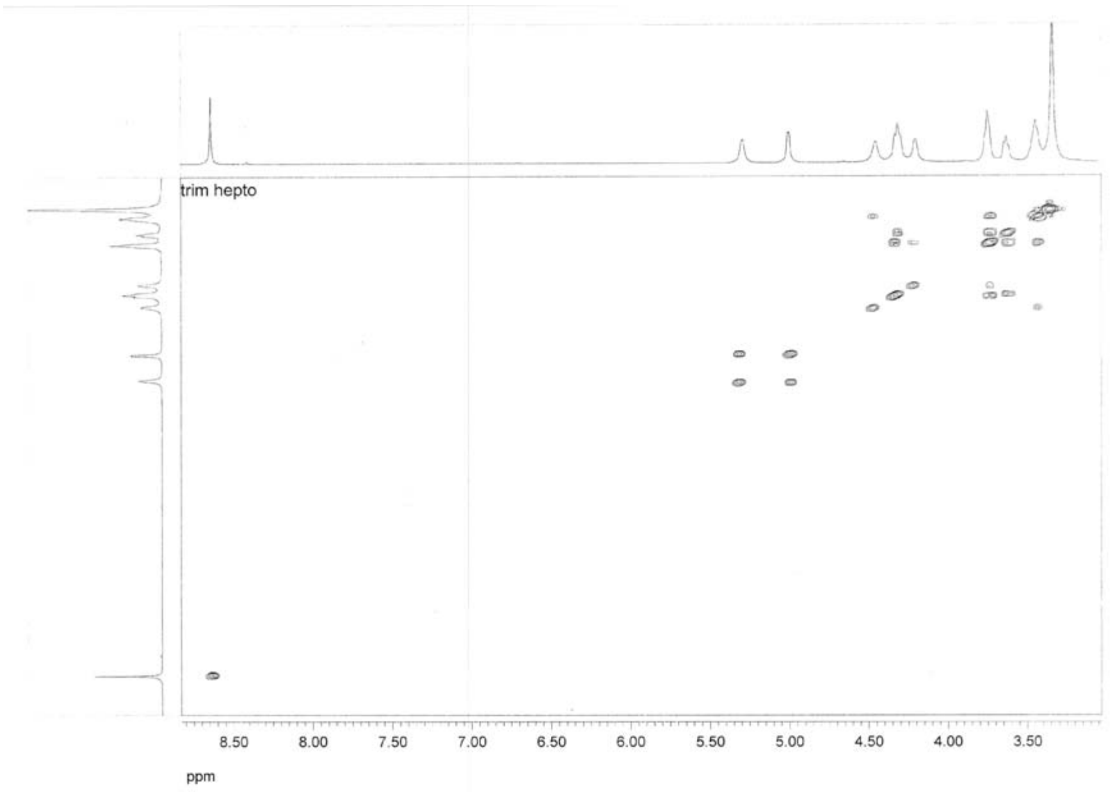

Figure S89. COSY spectrum of **43** in DMSO-d_6

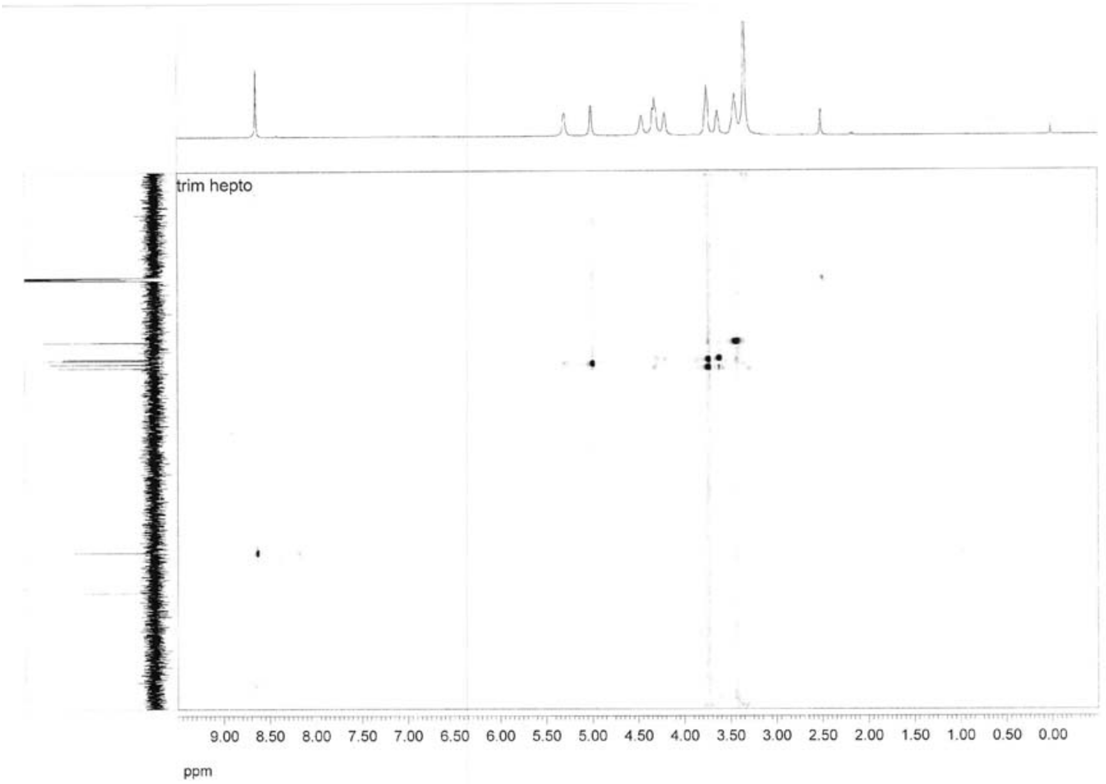

Figure S90. HMQC spectrum of **43** in DMSO-d_6

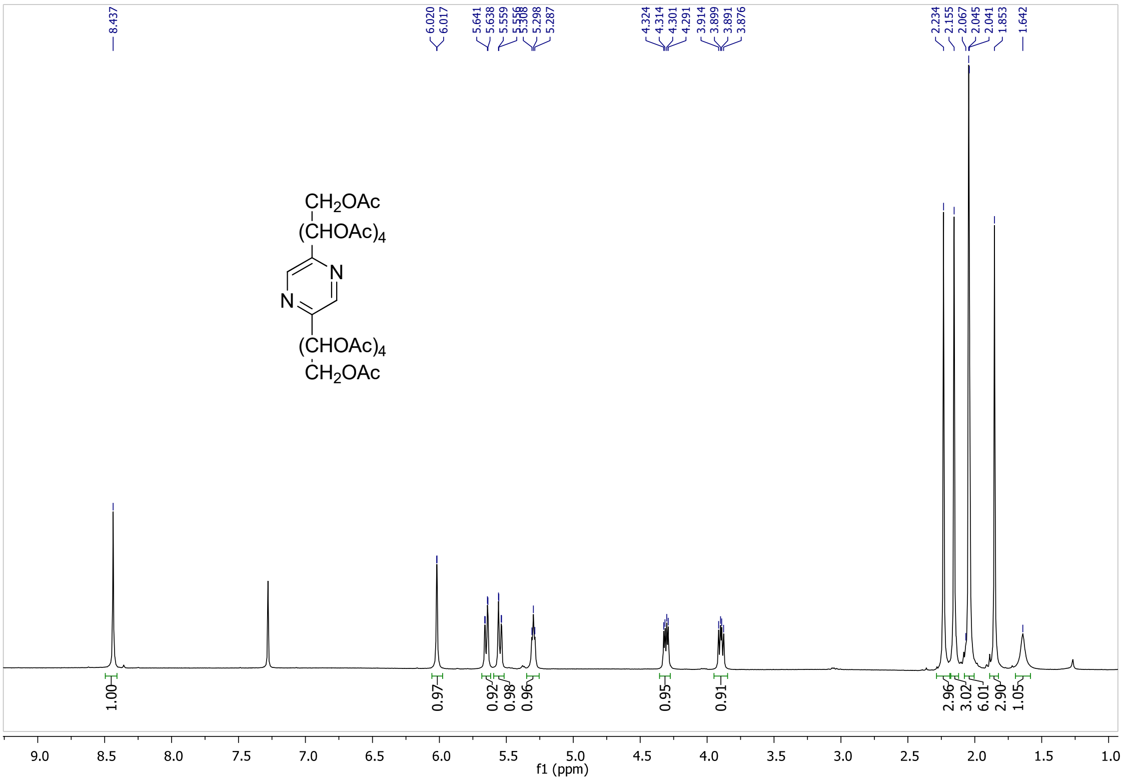

Figure S91. ^{1}H NMR spectrum of **44** in $CDCl_3$.

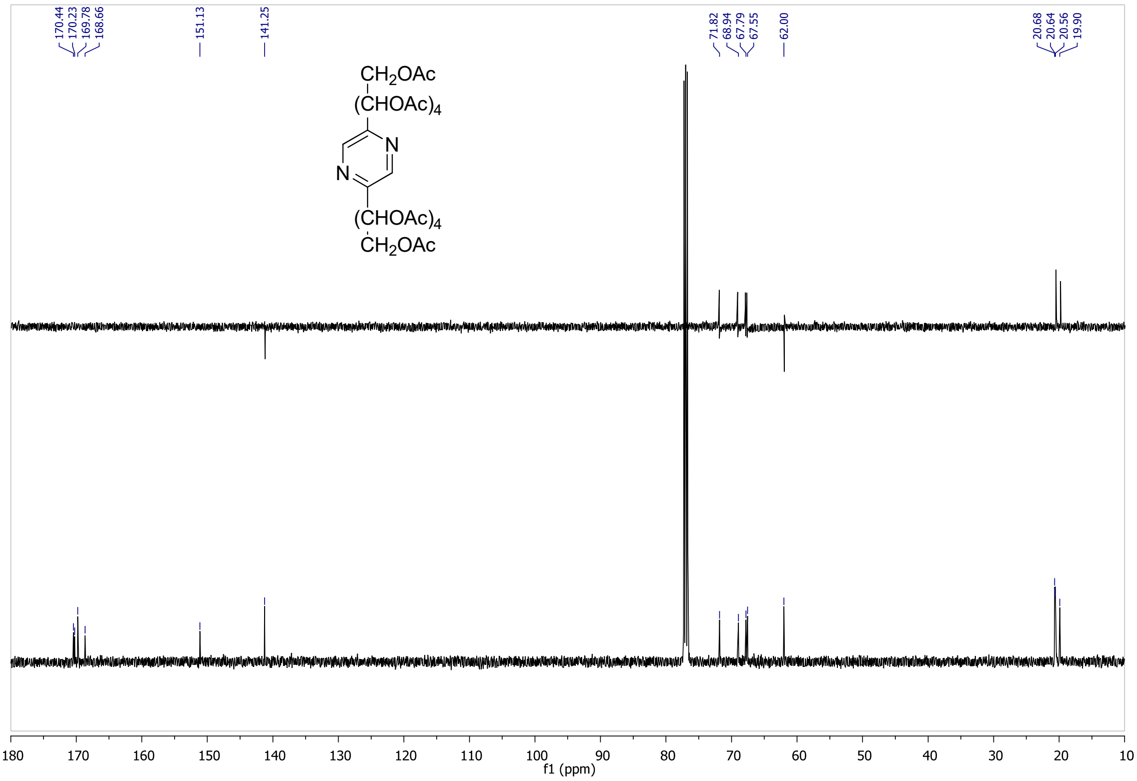

Figure S92. $^{13}C\{^{1}H\}$ NMR (top: DEPT) spectra of **44** in $CDCl_3$.

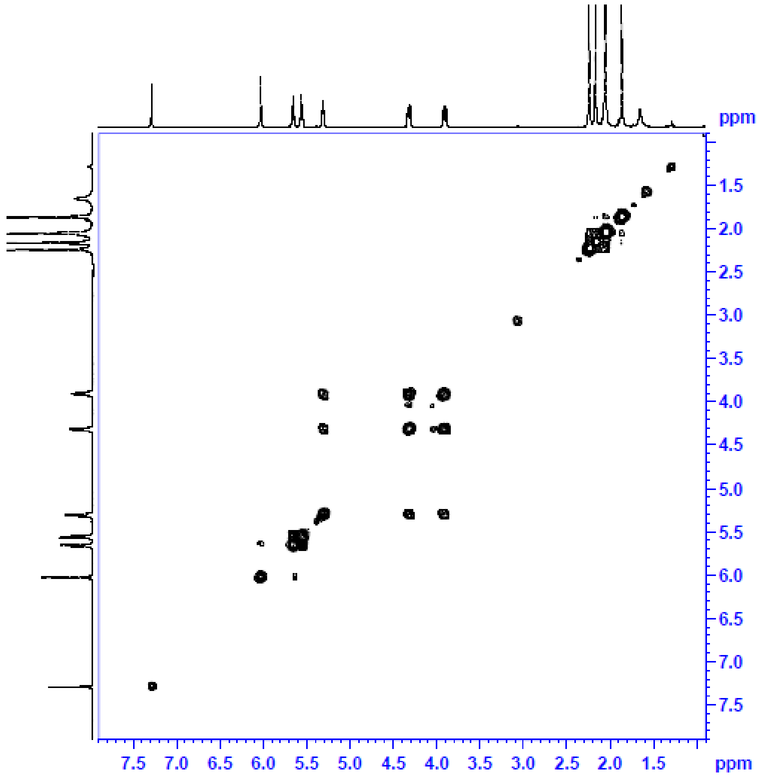

Figure S93. COSY spectrum of **44** in $CDCl_3$

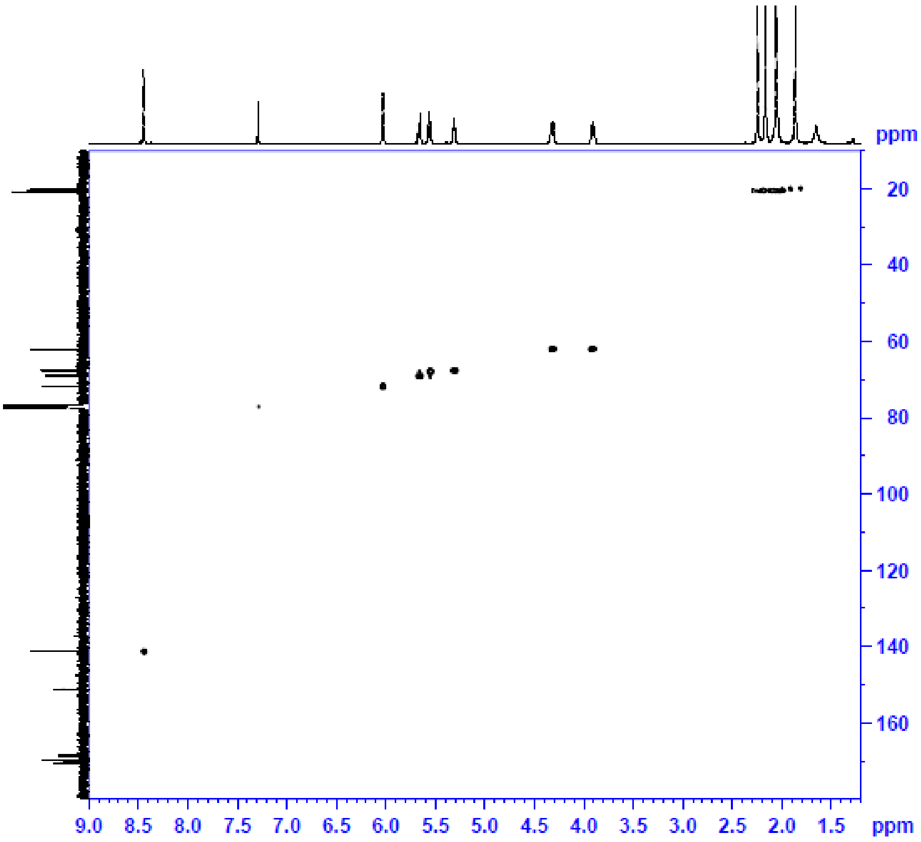

Figure S94. HMQC spectrum of **44** in $CDCl_3$

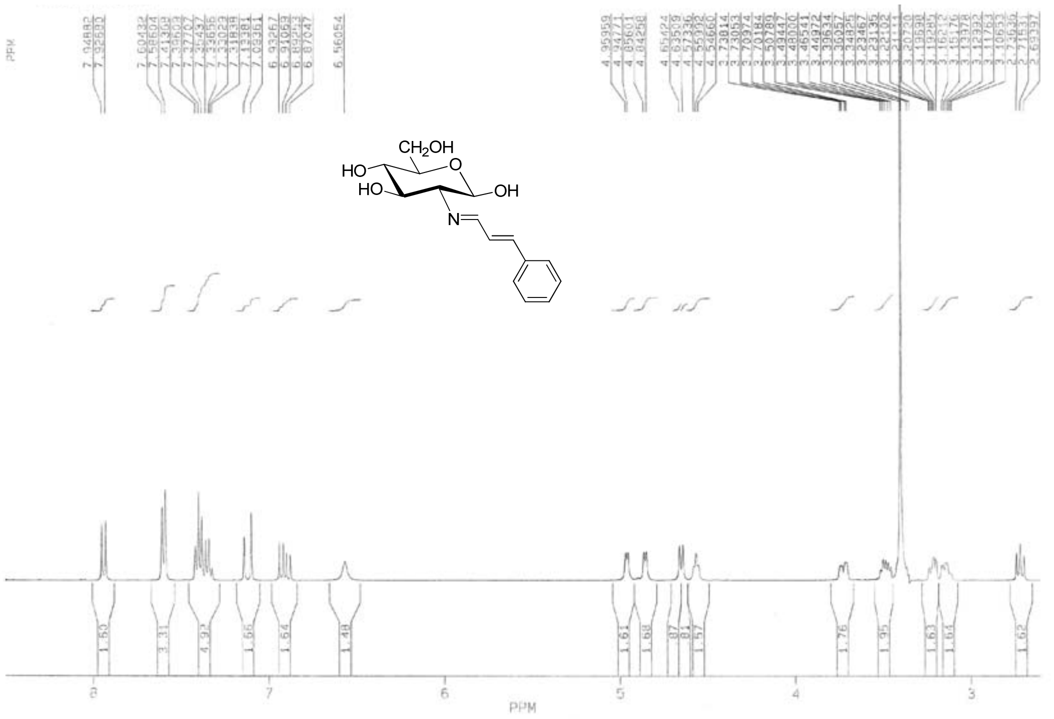

Figure S95. ^{1}H NMR spectrum of **45** in DMSO-d_6.

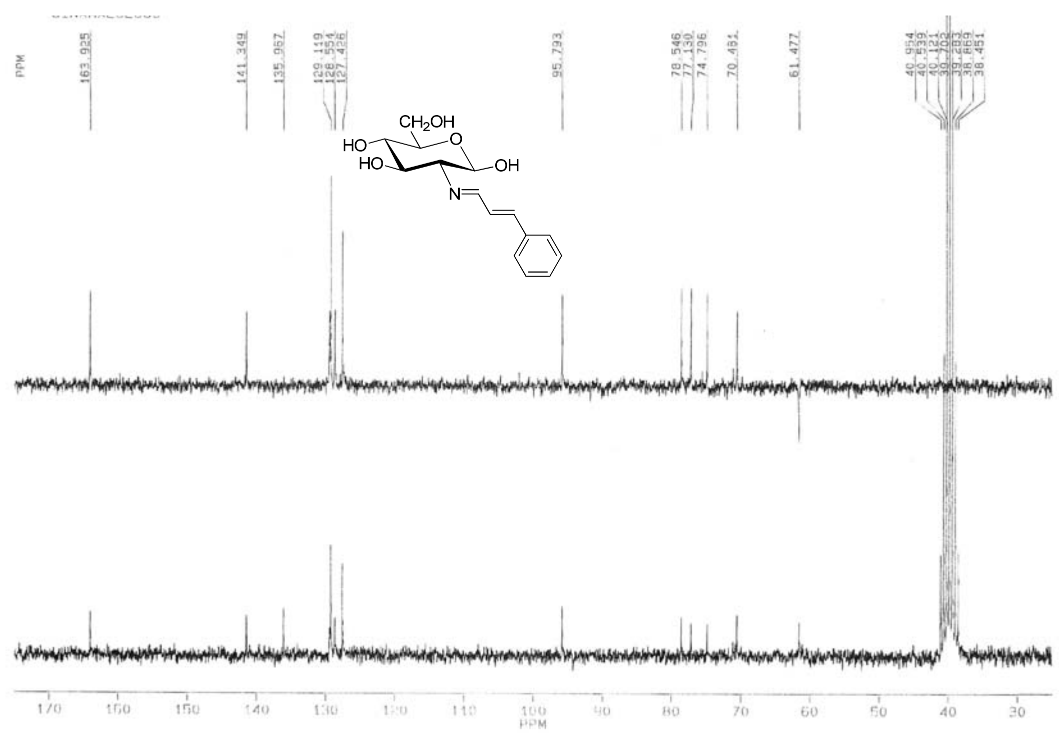

Figure S96. ^{13}C{^{1}H} NMR (top: DEPT) spectra of **45** in DMSO-d_6.

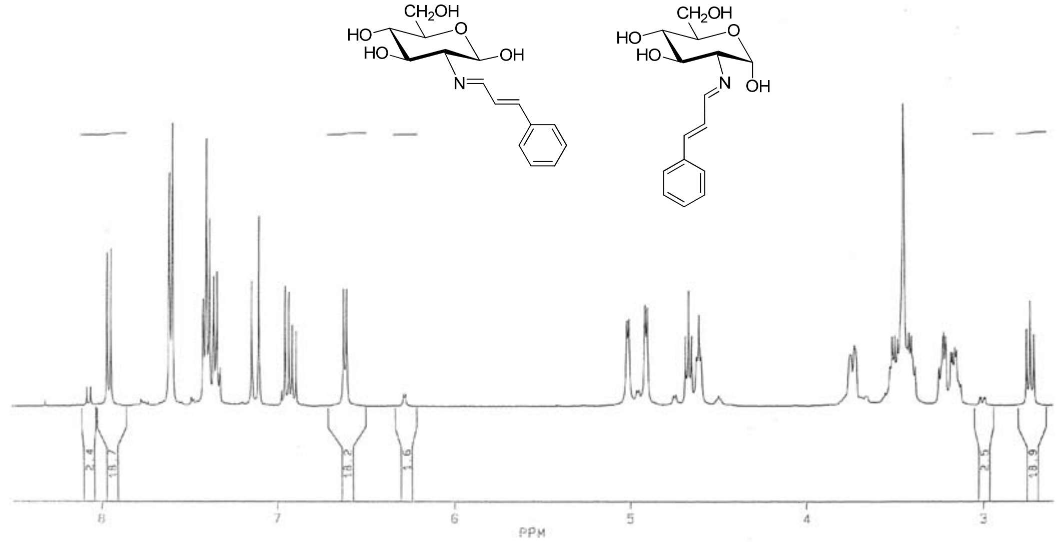

Figure S97. ^{1}H NMR spectrum of **45** and **105** in DMSO-*d*$_6$.

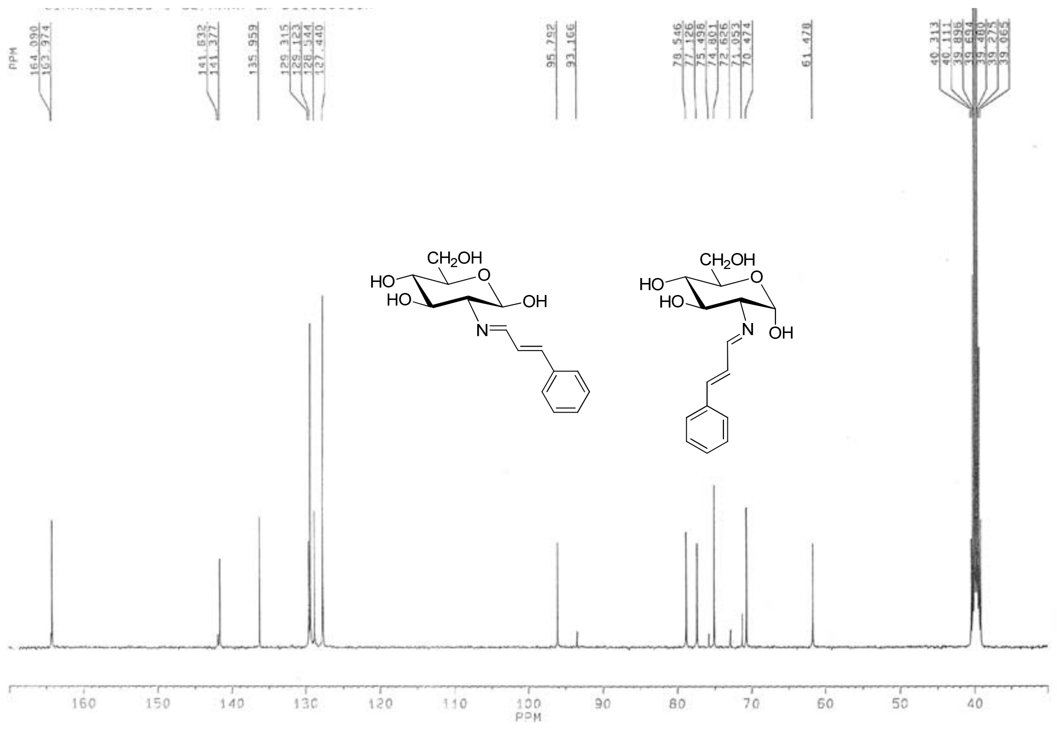

Figure S98. ^{13}C {^{1}H} NMR spectrum of **45** and **105** in DMSO-*d*$_6$.

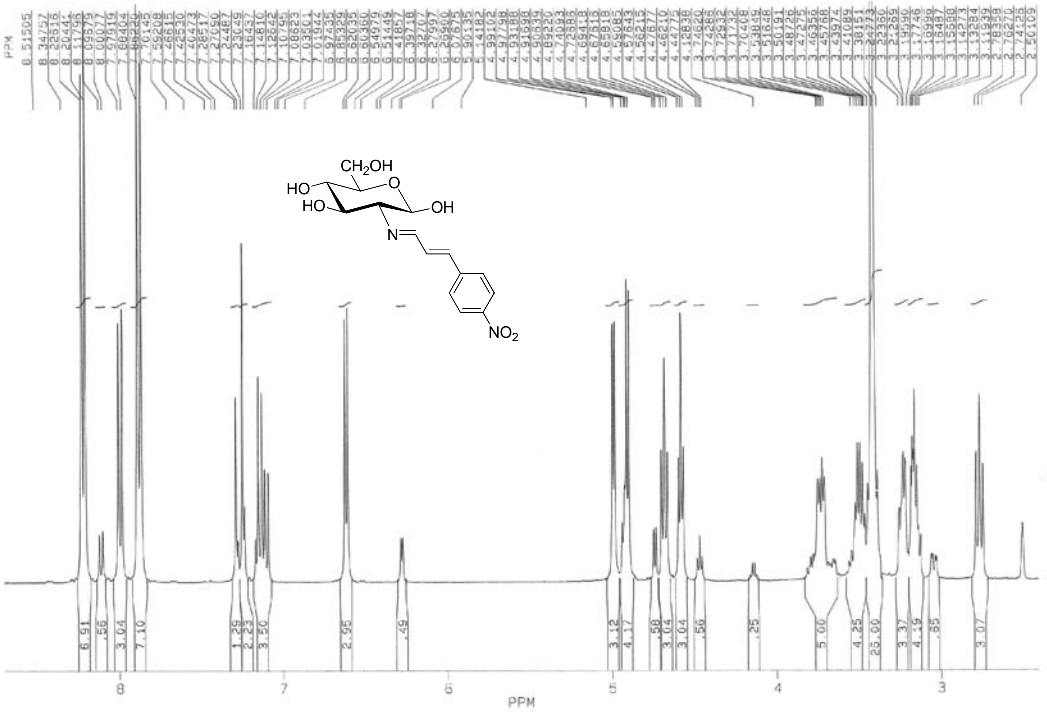

Figure S99. ^{1}H NMR spectrum of **46** in DMSO-d_6.

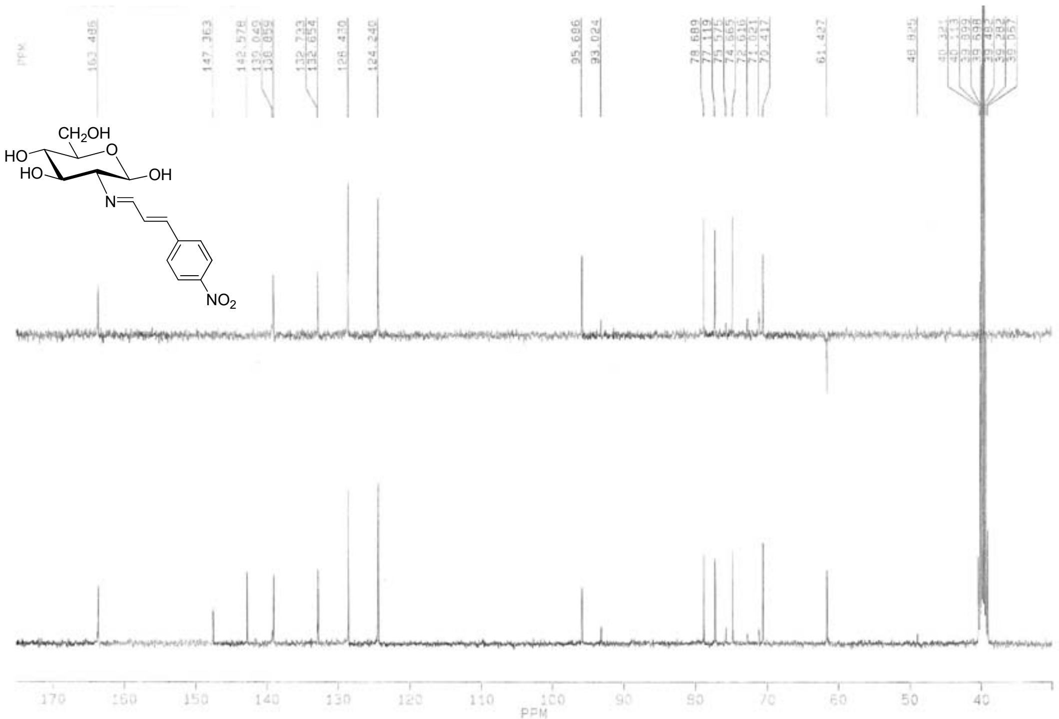

Figure S100. ^{13}C{^{1}H} NMR (top: DEPT) spectra of **46** in DMSO-d_6.

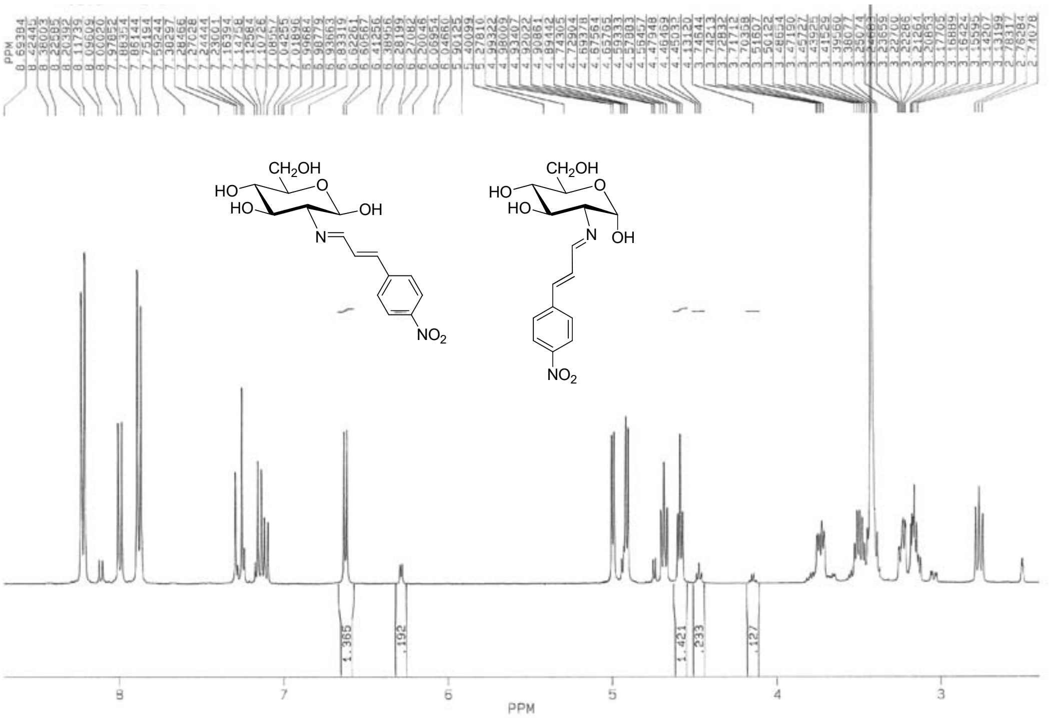

Figure S101. ^{1}H NMR spectrum of **46** and **106** in DMSO-*d*₆.

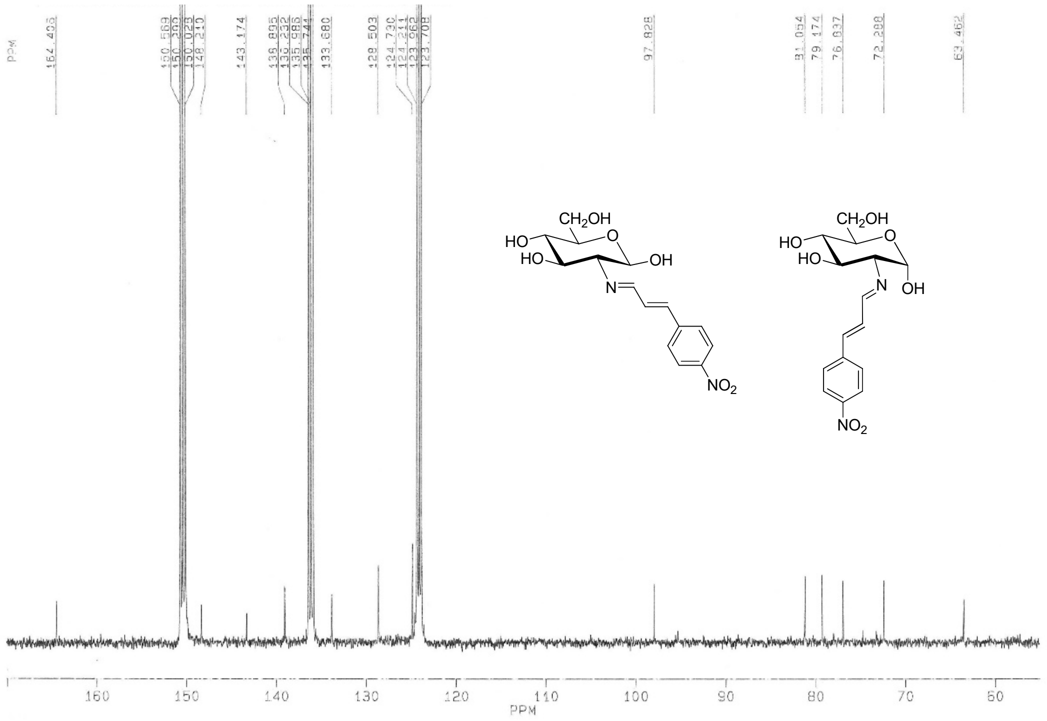

Figure S102. ^{13}C {^{1}H} NMR spectrum of **46** and **106** in DMSO-*d*₆.

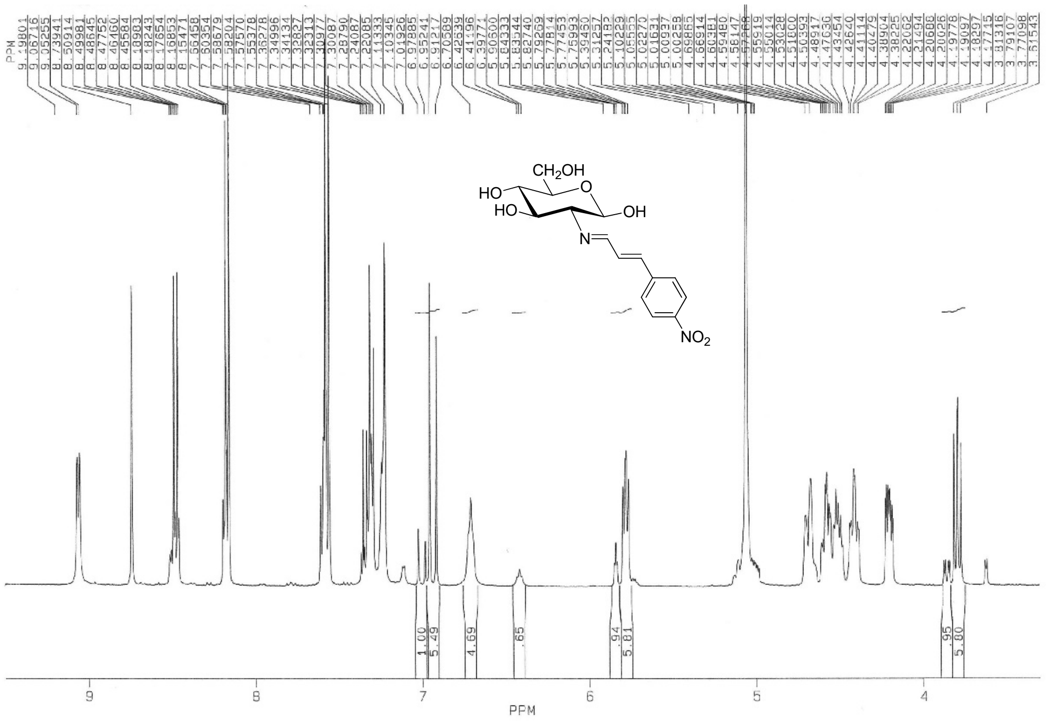

Figure S103. ¹H NMR spectrum of **46** in pyridine-d_5.

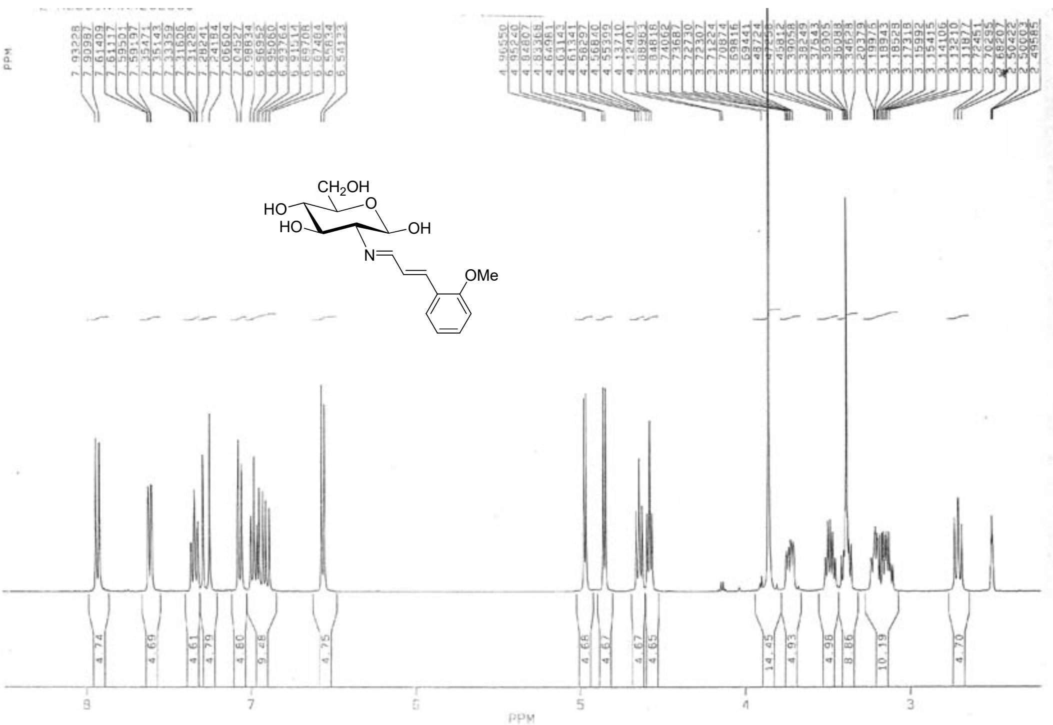

Figure S104. ¹H NMR spectrum of **47** in DMSO-d_6.

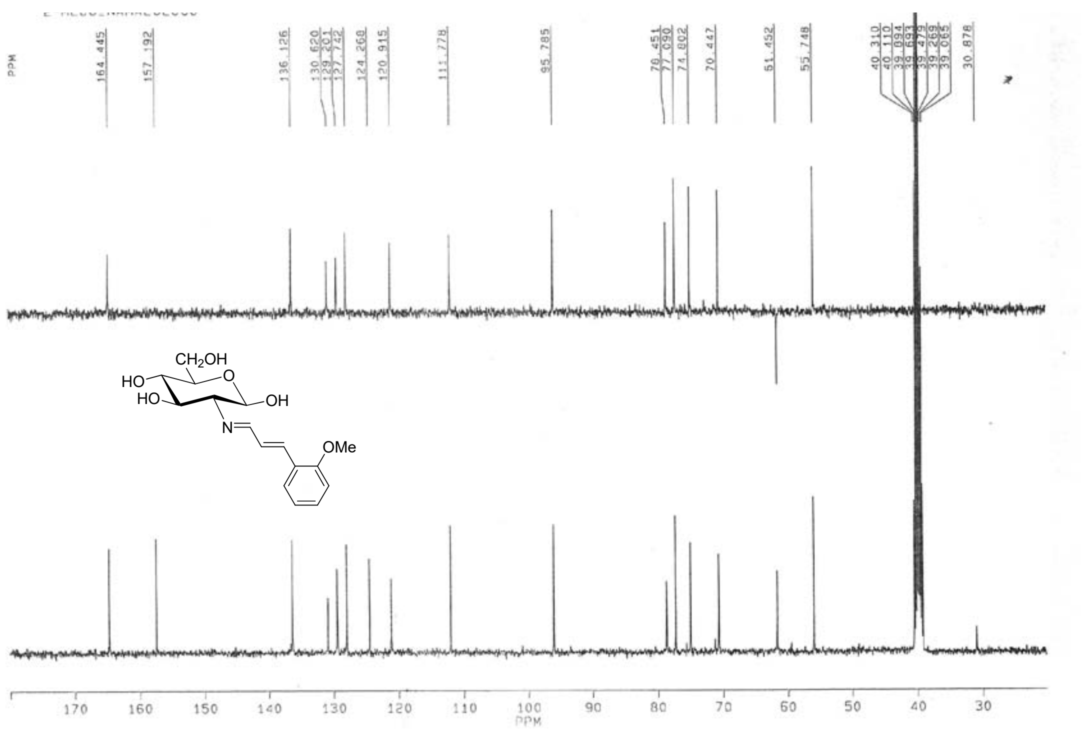

Figure S105. $^{13}C\{^{1}H\}$ NMR (top: DEPT) spectra of **47** in DMSO-d_6.

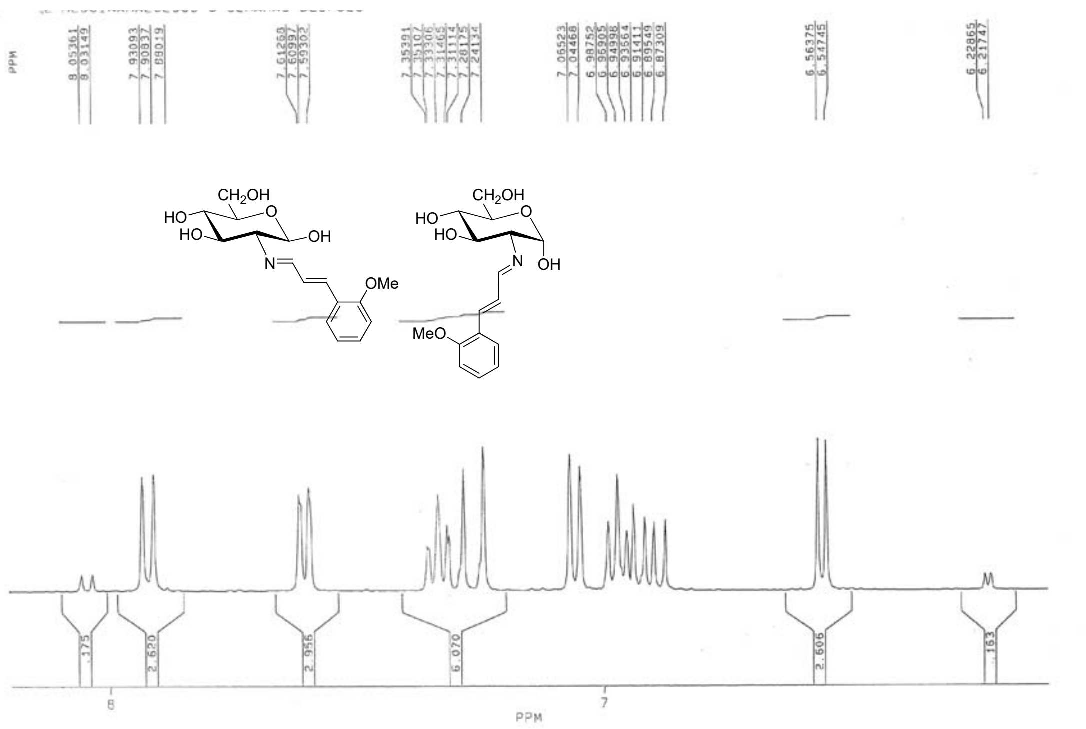

Figure S106. ^{1}H NMR spectrum of **47** and **107** in DMSO-d_6.

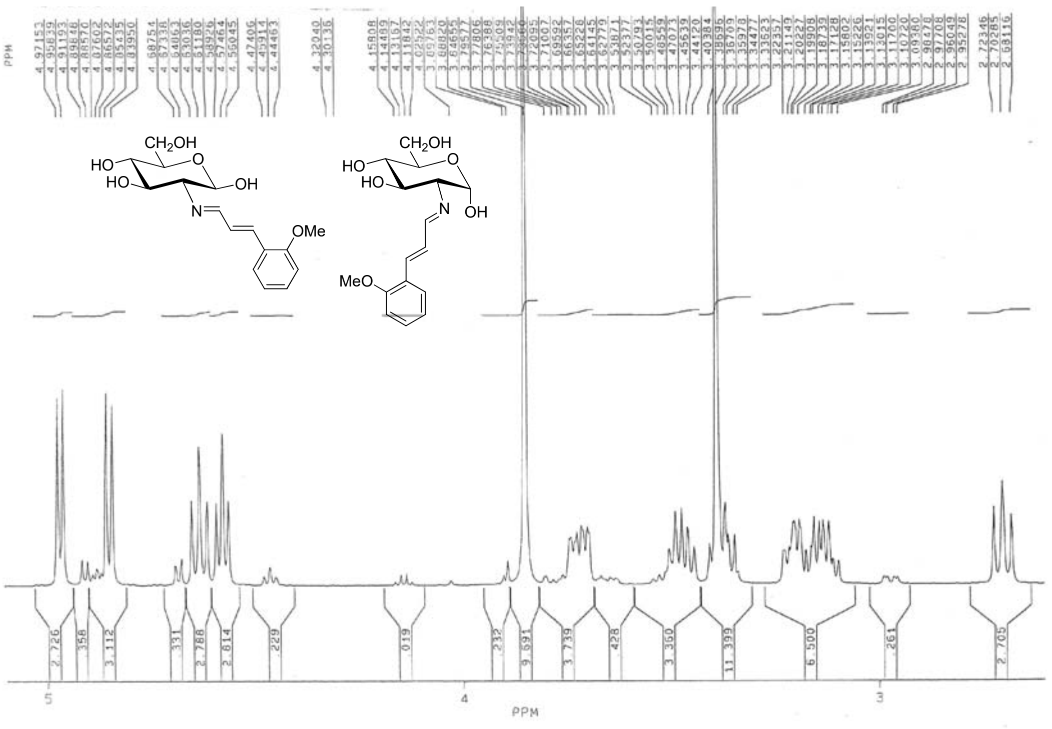

Figure S107. ^{1}H NMR spectrum of **47** and **107** in DMSO-*d*$_6$.

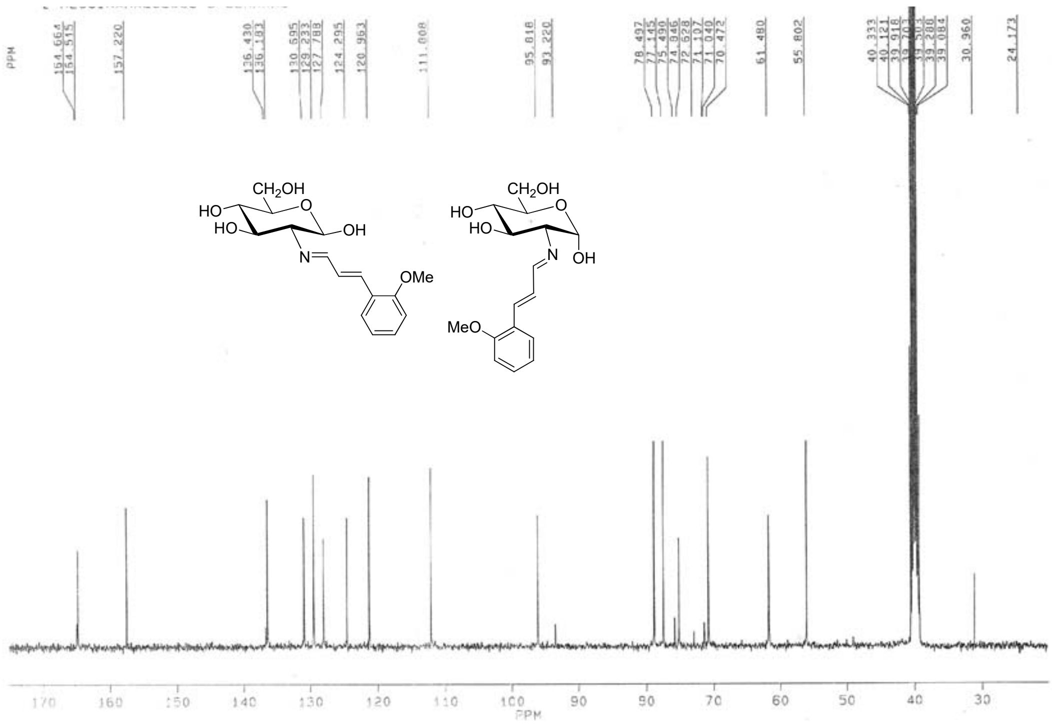

Figure S108. ^{13}C {^{1}H} NMR spectrum of **47** and **107** in DMSO-*d*$_6$.

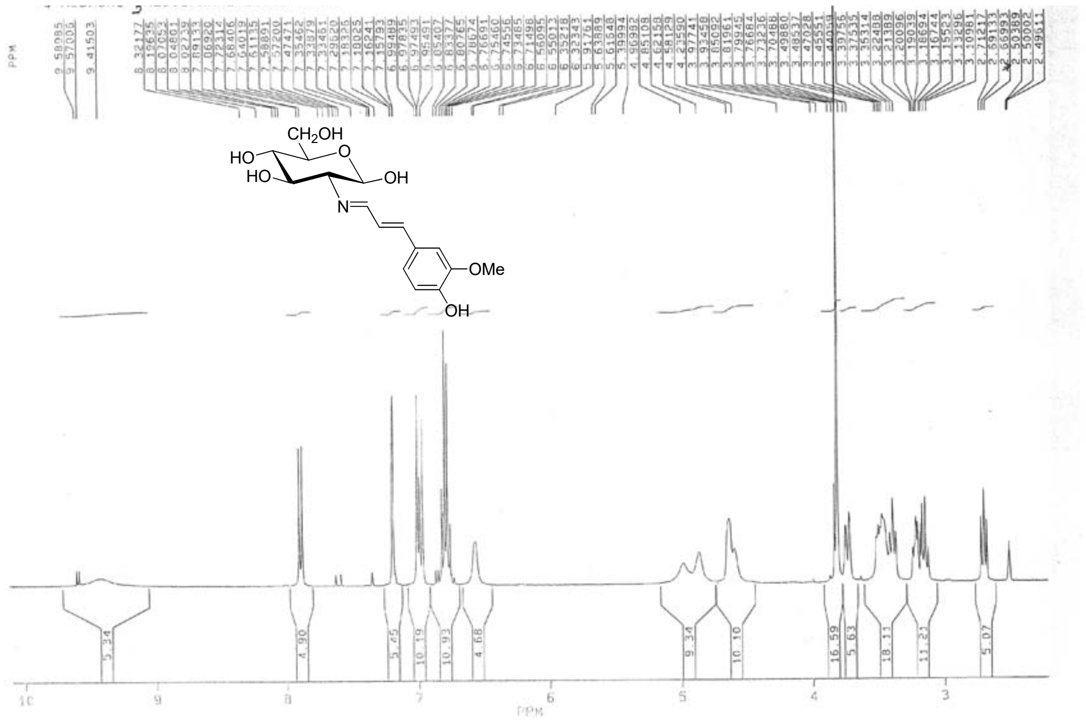

Figure S109. ^1H NMR spectrum of **48** in DMSO-*d*6.

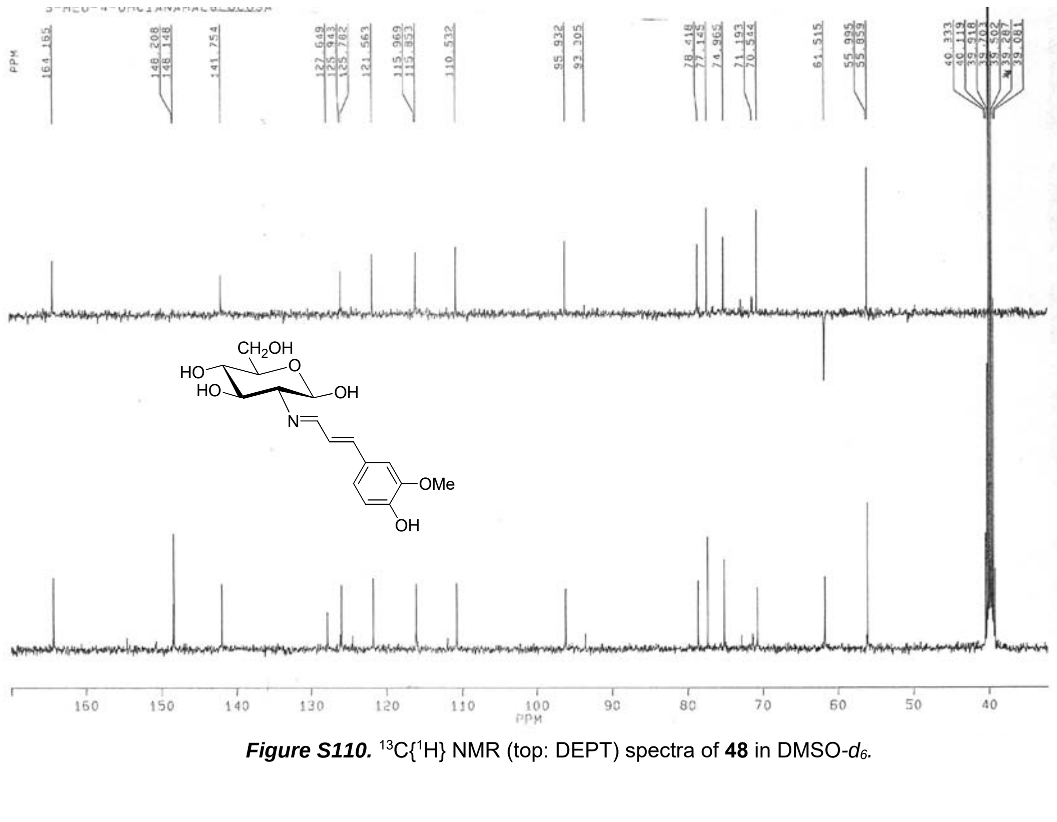

Figure S110. $^{13}C\{^1H\}$ NMR (top: DEPT) spectra of **48** in DMSO-*d*6.

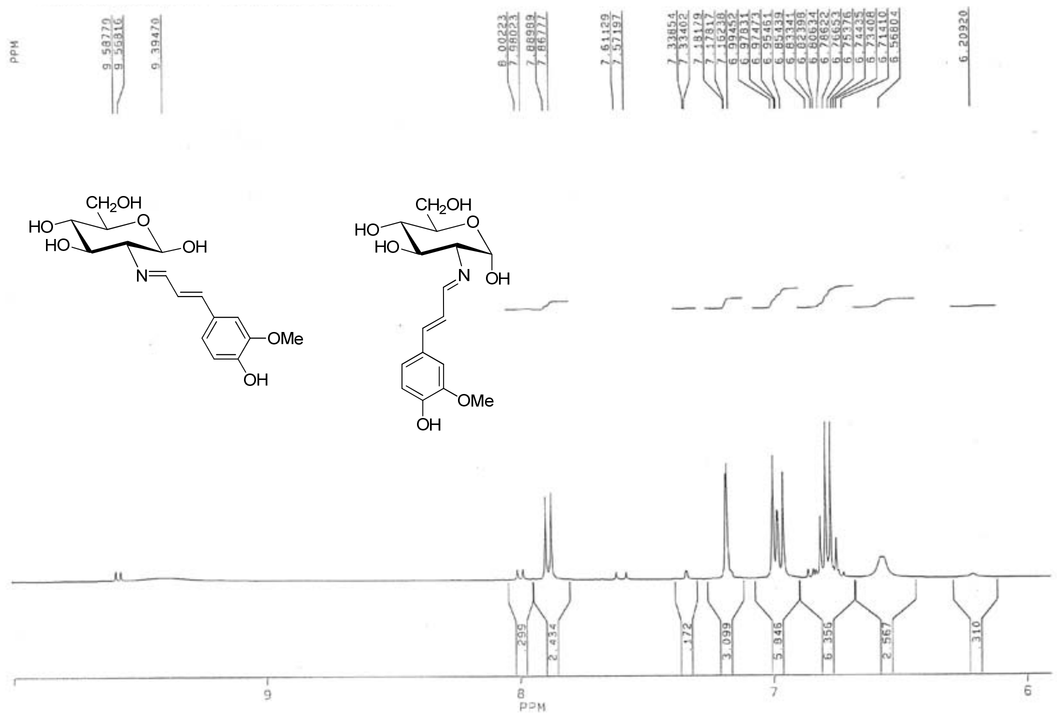

Figure S111. ^{1}H NMR spectrum of **48** and **108** in DMSO-*d*$_6$.

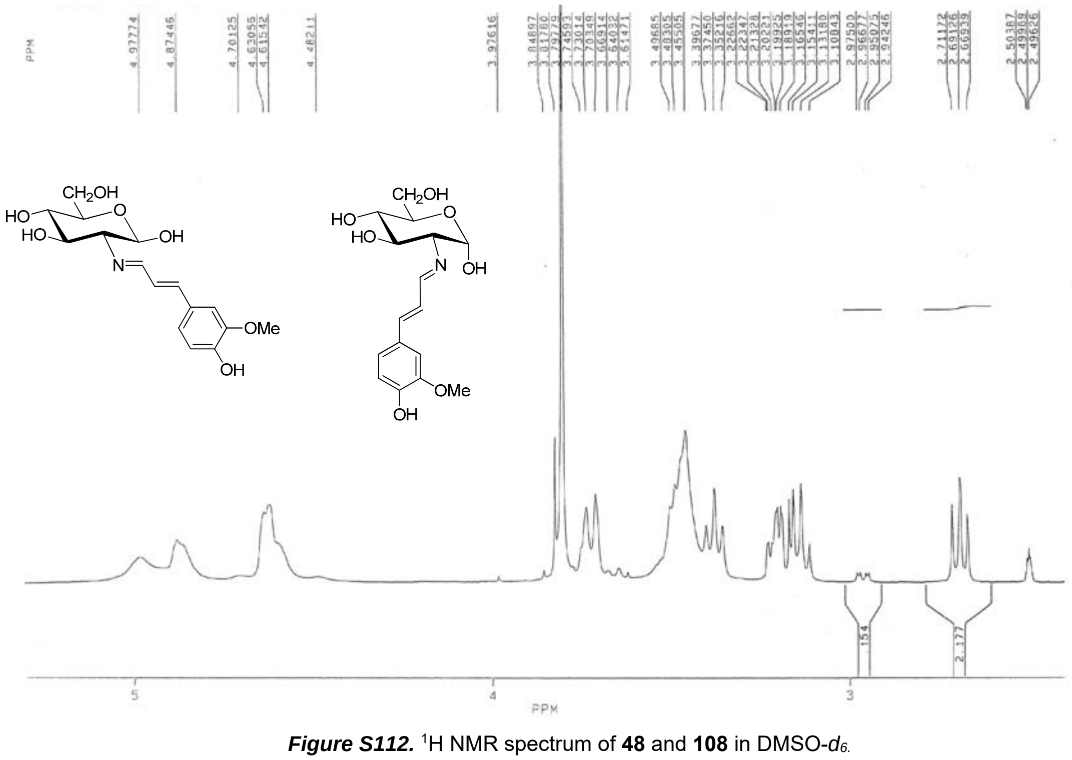

Figure S112. ^{1}H NMR spectrum of **48** and **108** in DMSO-*d*$_6$.

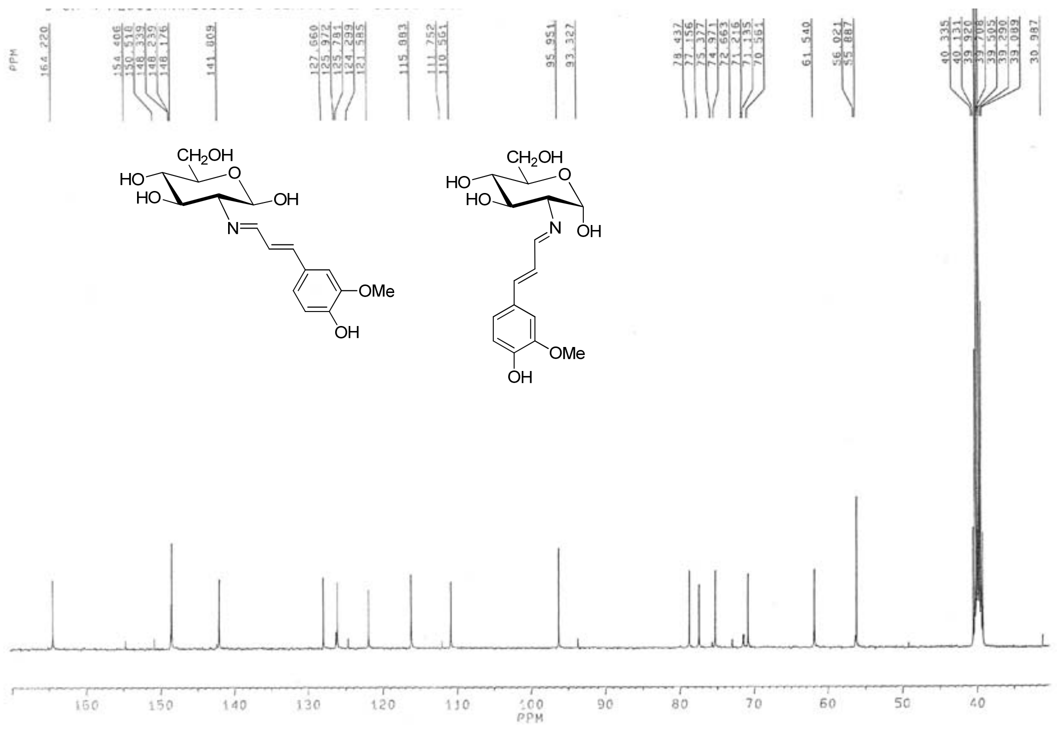

Figure S113. $^{13}C\{^1H\}$ NMR spectrum of **48** and **108** in DMSO-d_6.

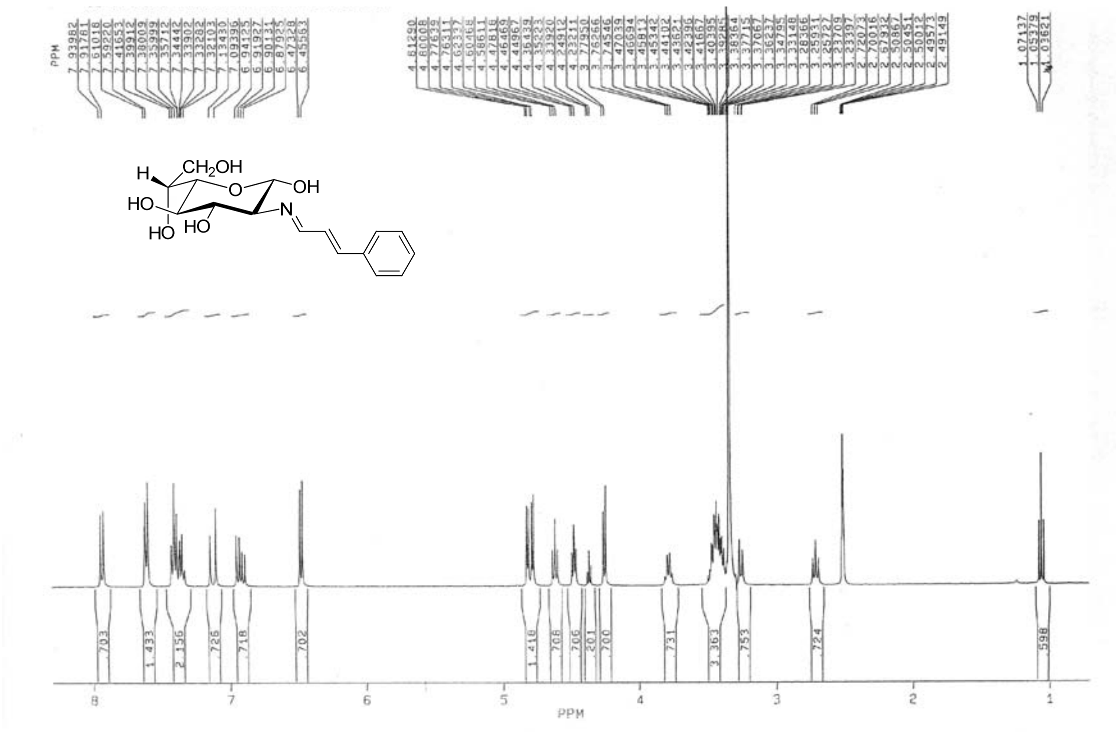

Figure S114. ^1H NMR spectrum of **49** in DMSO-d_6.

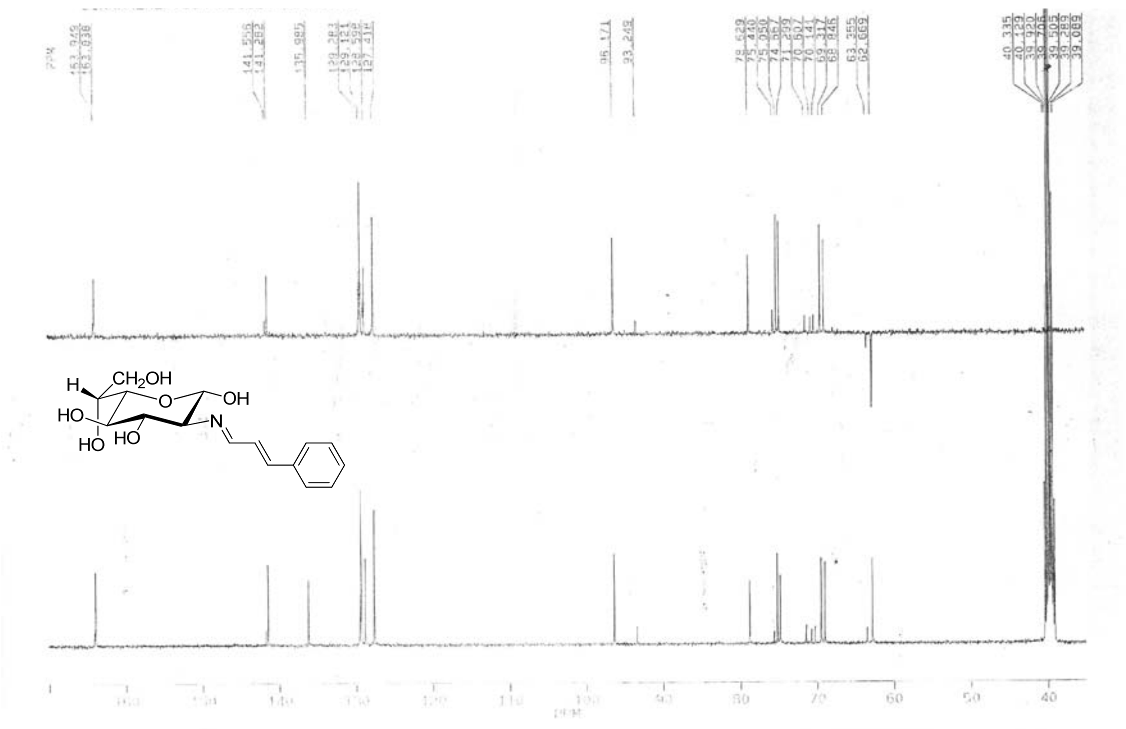

Figure S115. $^{13}C\{^1H\}$ NMR (top: DEPT) spectra of **49** in DMSO-d_6.

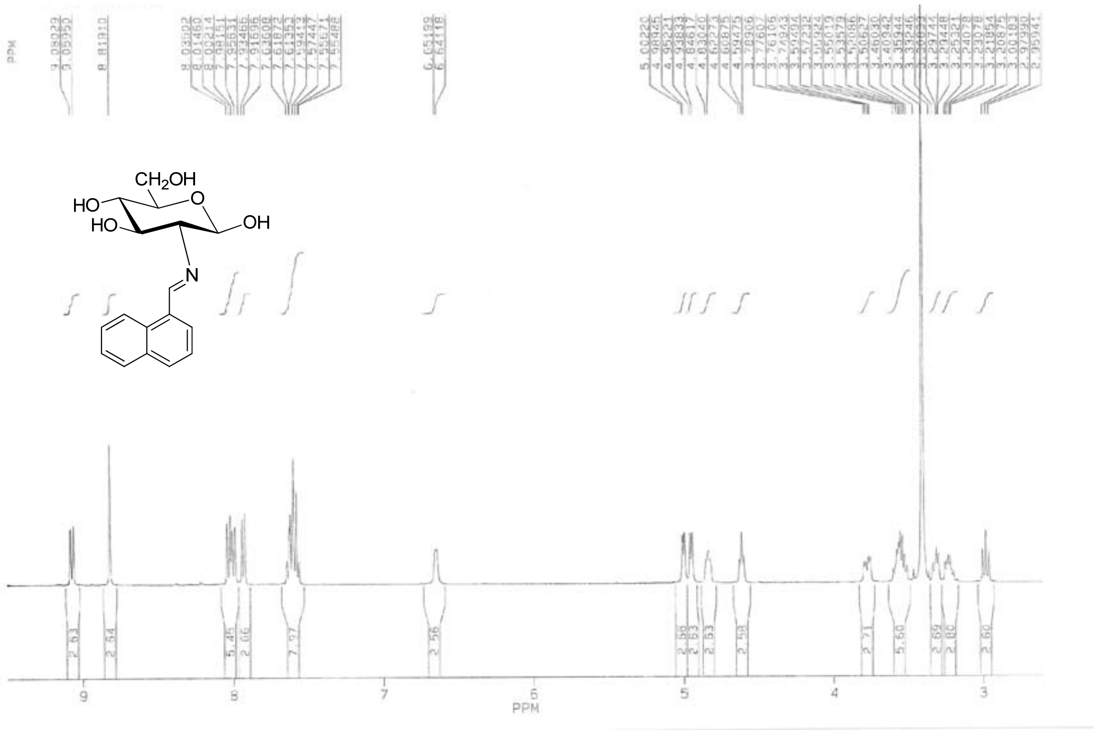

Figure S116. ^1H NMR spectrum of **50** in DMSO-d_6.

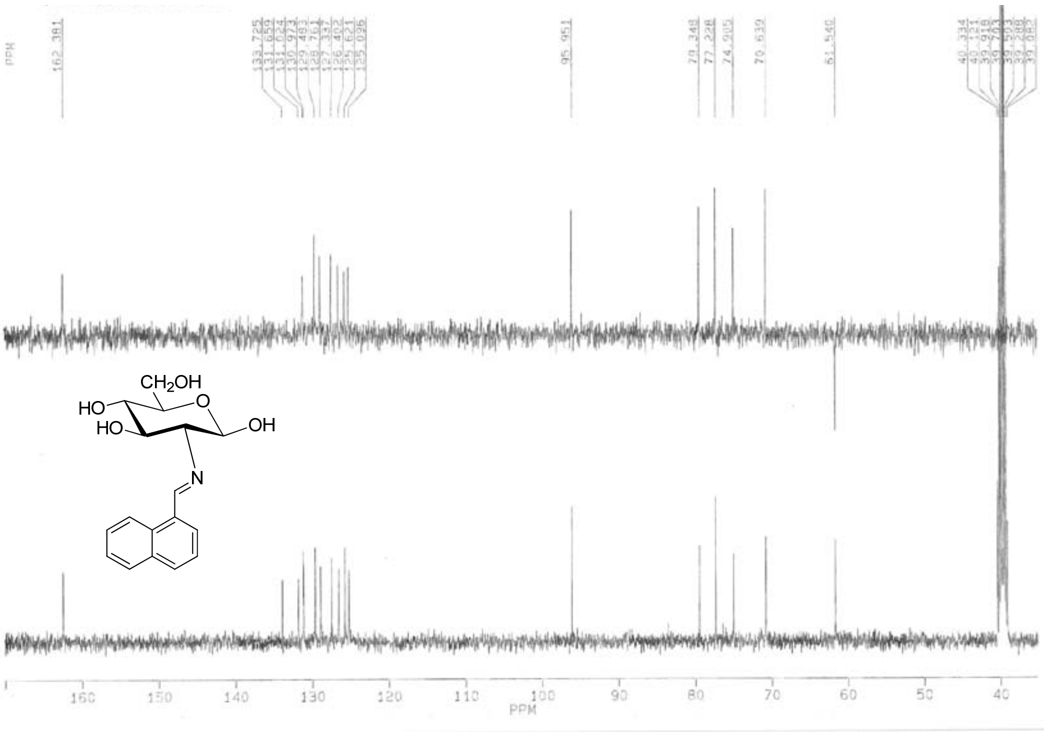

Figure S117. $^{13}C\{^{1}H\}$ NMR (top: DEPT) spectra of **50** in DMSO-*d₆*.

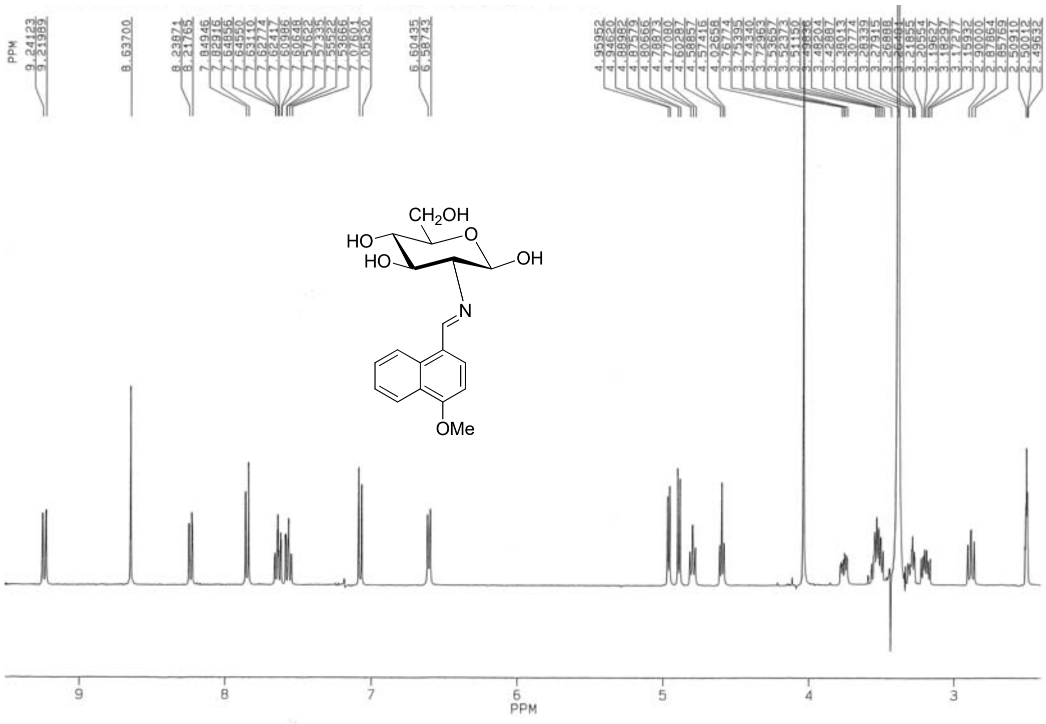

Figure S118. ^{1}H NMR spectrum of **51** in DMSO-*d₆*.

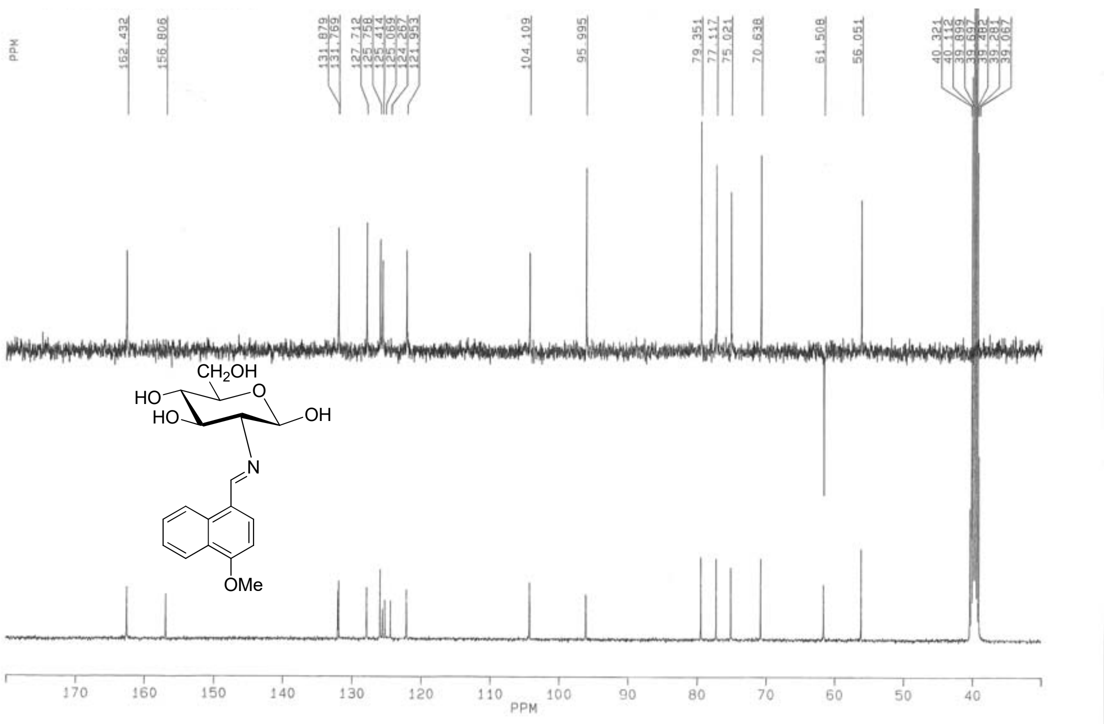

Figure S119. $^{13}C\{^1H\}$ NMR (top: DEPT) spectra of **51** in DMSO-*d*$_6$

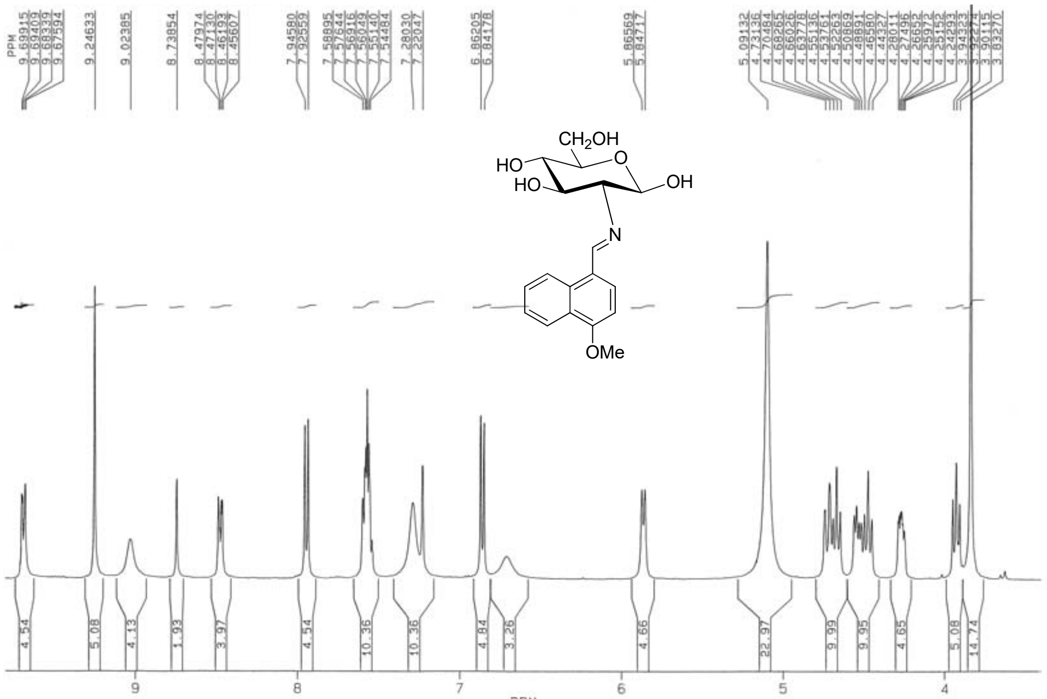

Figure S120. ^1H NMR spectrum of **51** in Pyridine-*d*$_5$.

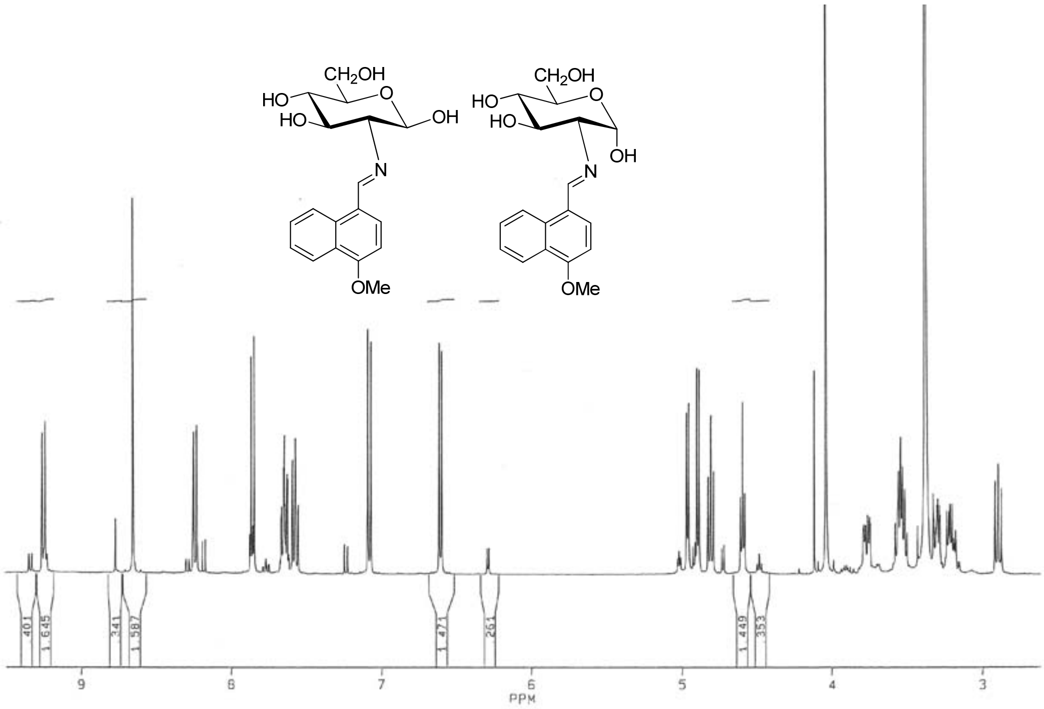

Figure S121. ^{1}H NMR spectrum of **51 and 52** in DMSO-d_6.

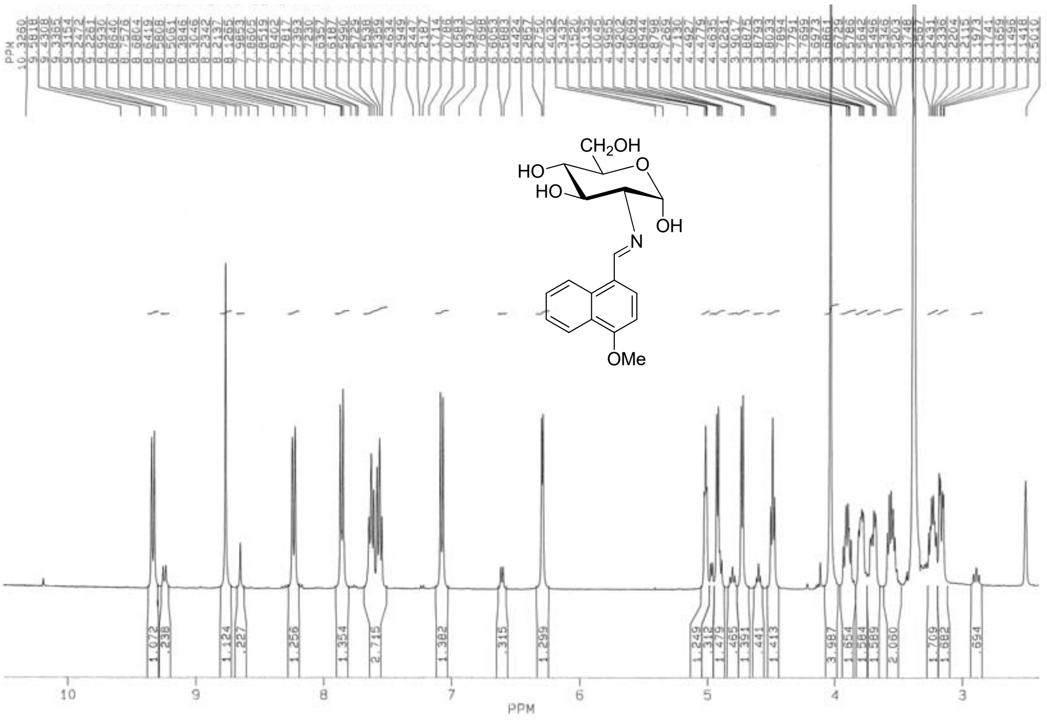

Figure S122. ^{1}H NMR spectrum of **52** in DMSO-d_6.

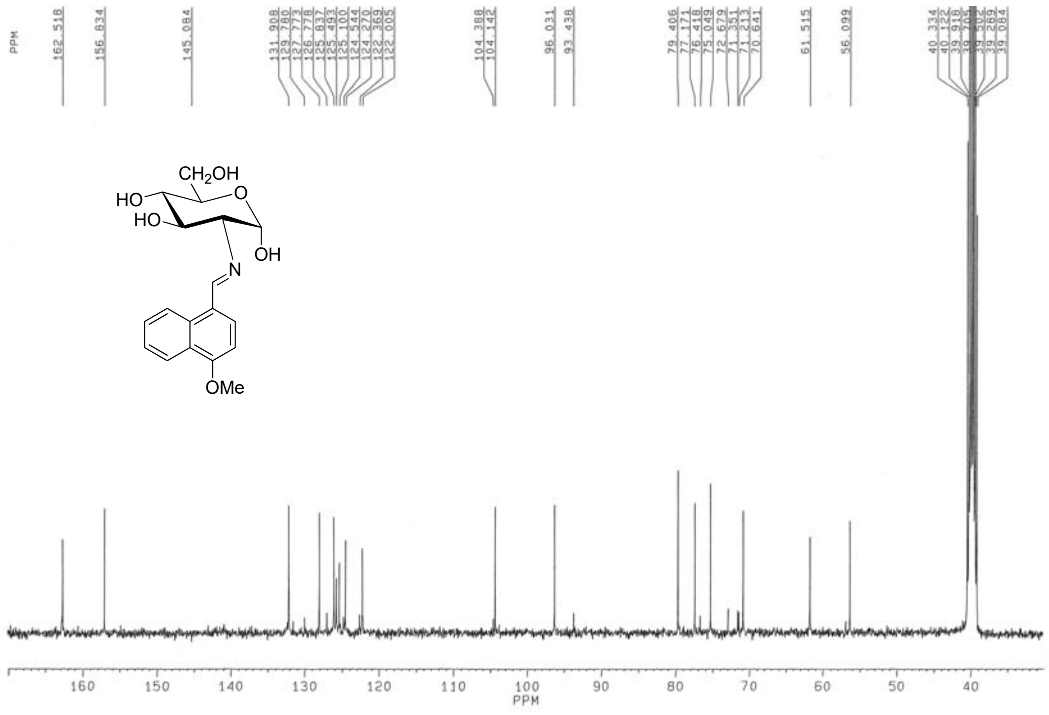

Figure S123. $^{13}C\{^{1}H\}$ NMR spectrum of **52** in DMSO-d_6

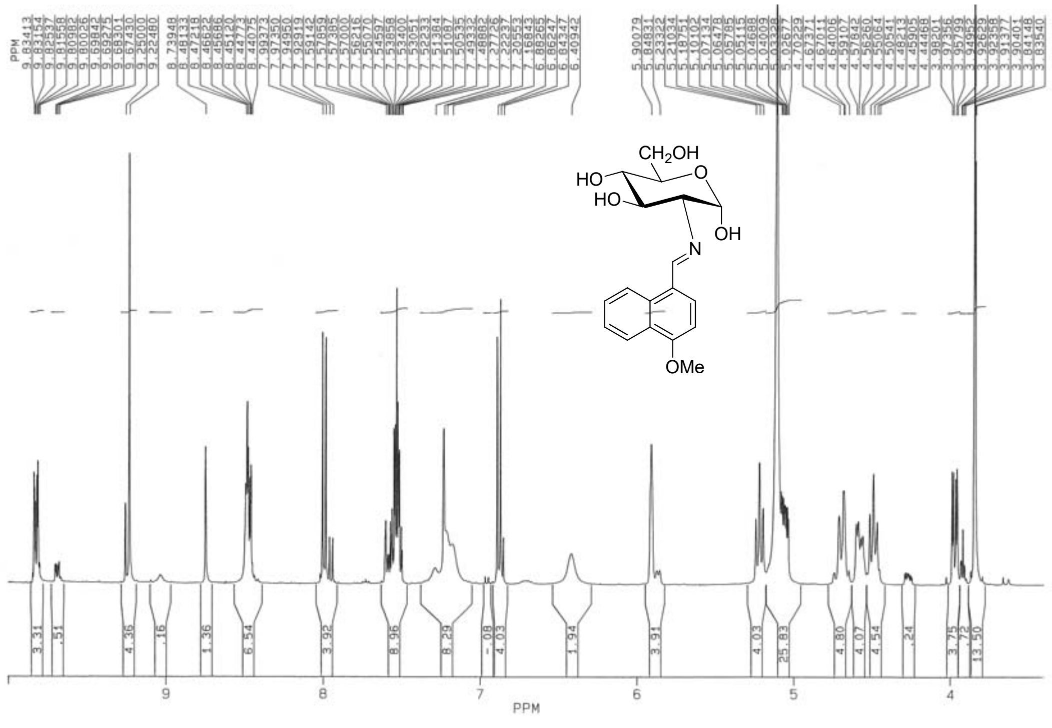

Figure S124. ^{1}H NMR spectrum of **52** in Pyridine-d_5.

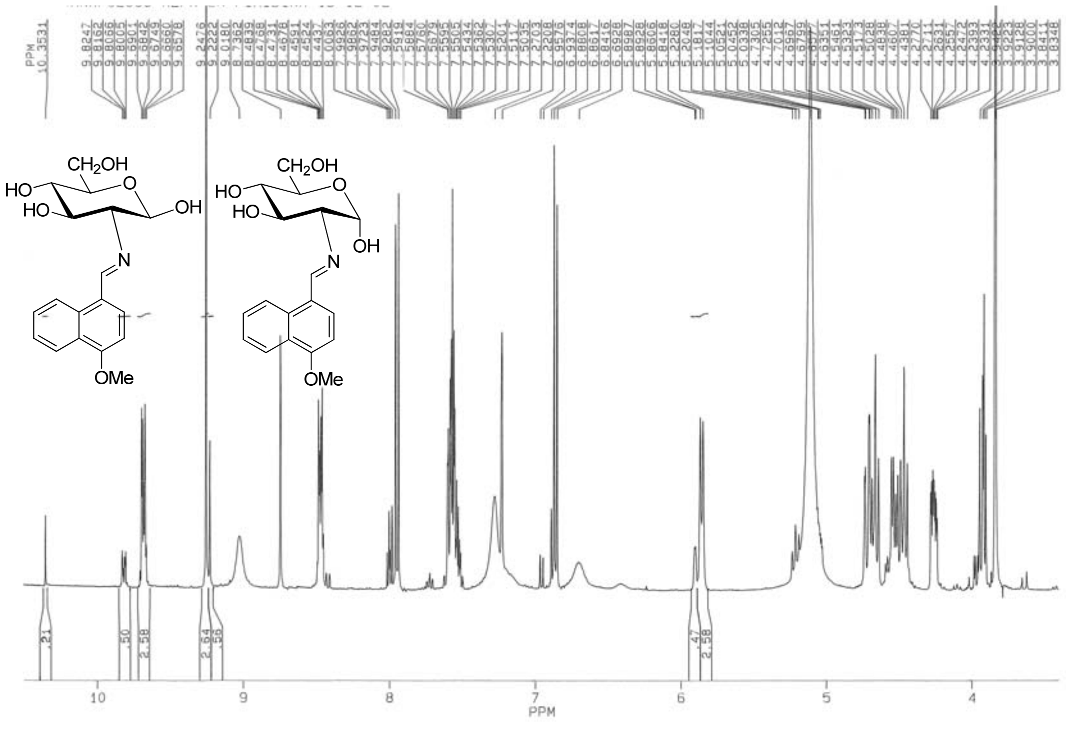

Figure S125. ^{1}H NMR spectrum of **51** and **52** in Pyridine-d_5.

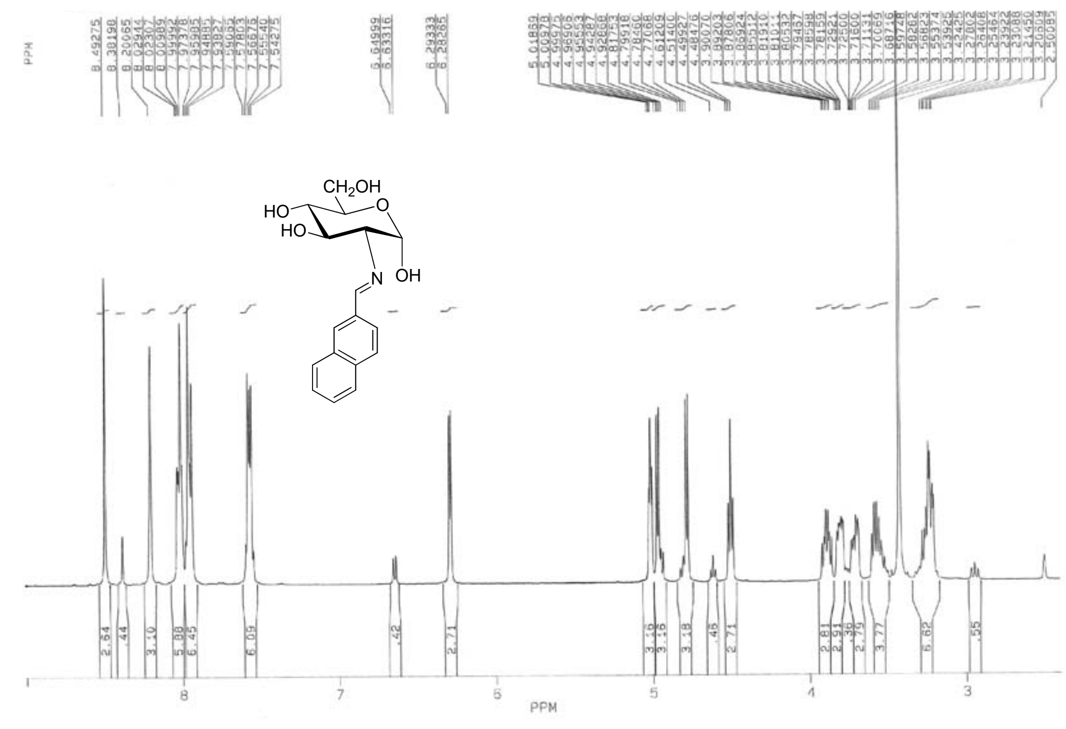

Figure S126. ^{1}H NMR spectrum of **54** in DMSO-d_6

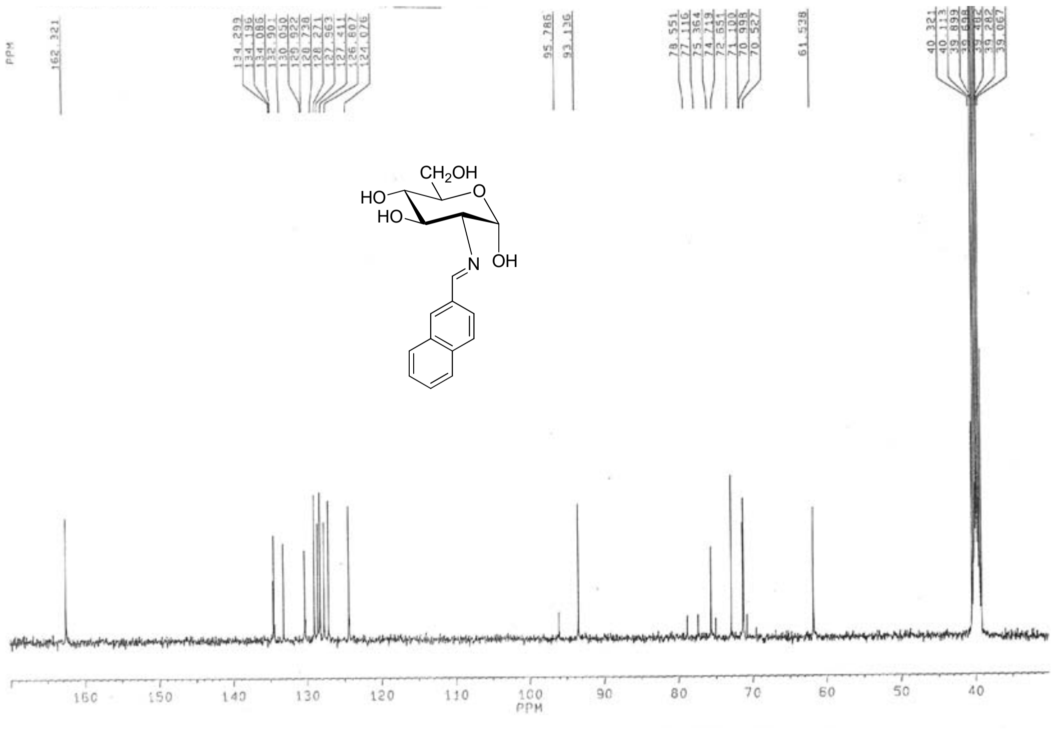

Figure S127. ^{13}C {^{1}H} NMR spectrum of **54** in DMSO-*d*$_6$.

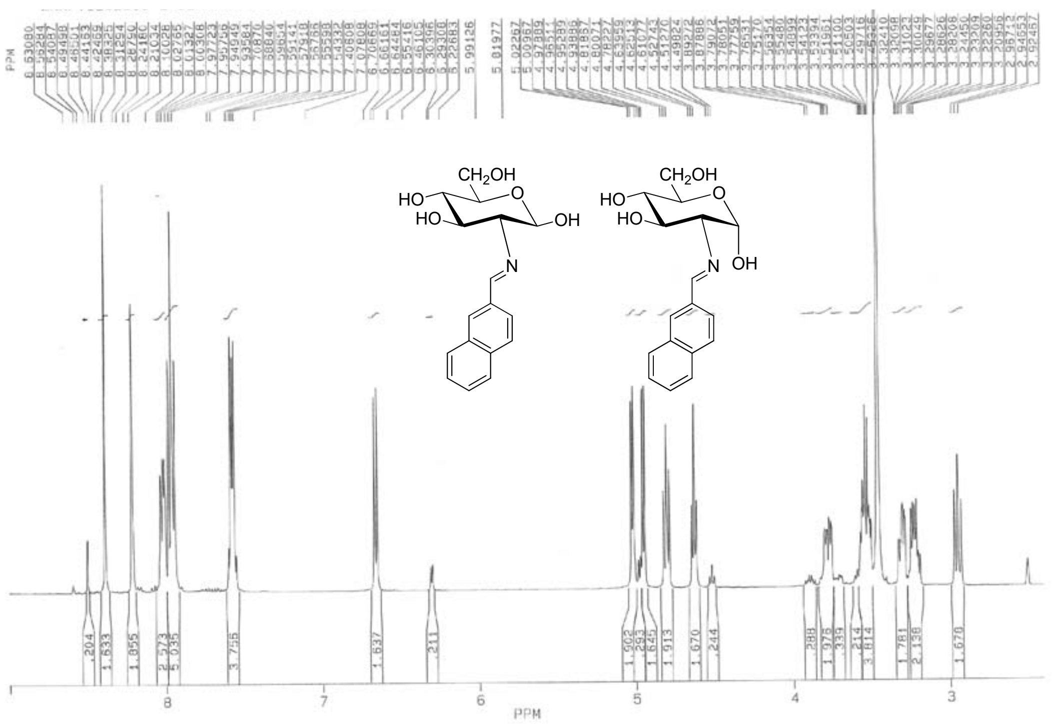

Figure S128. ^{1}H NMR spectrum of **53** and **54** in DMSO-*d*$_6$

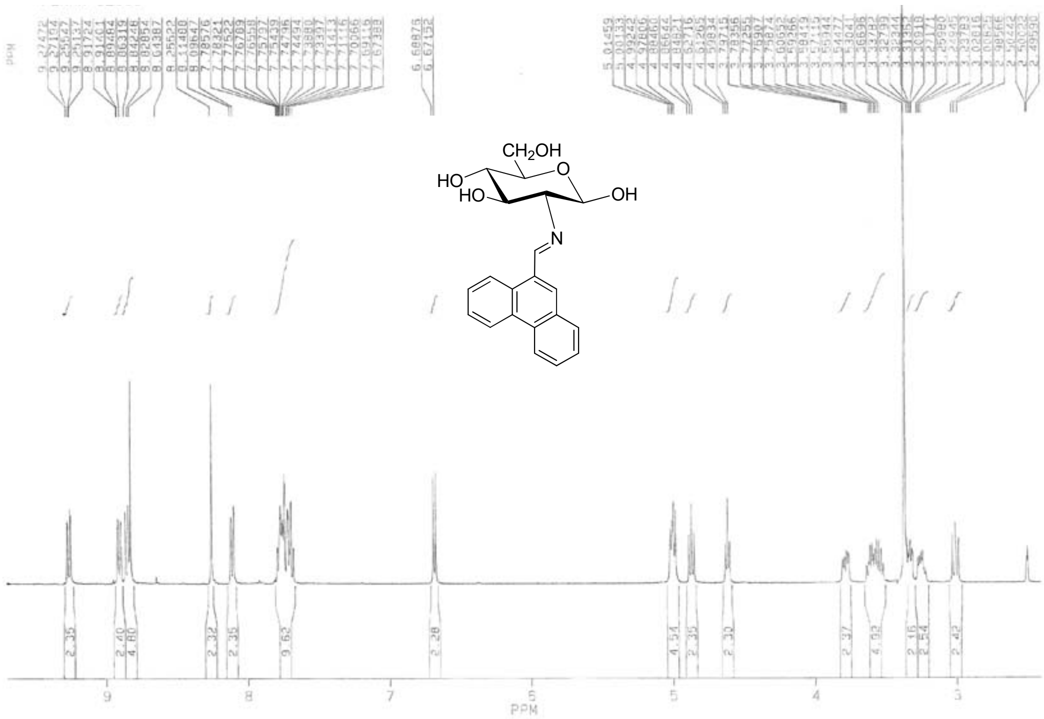

Figure S129. ^{1}H NMR spectrum of **55** in DMSO-*d₆*

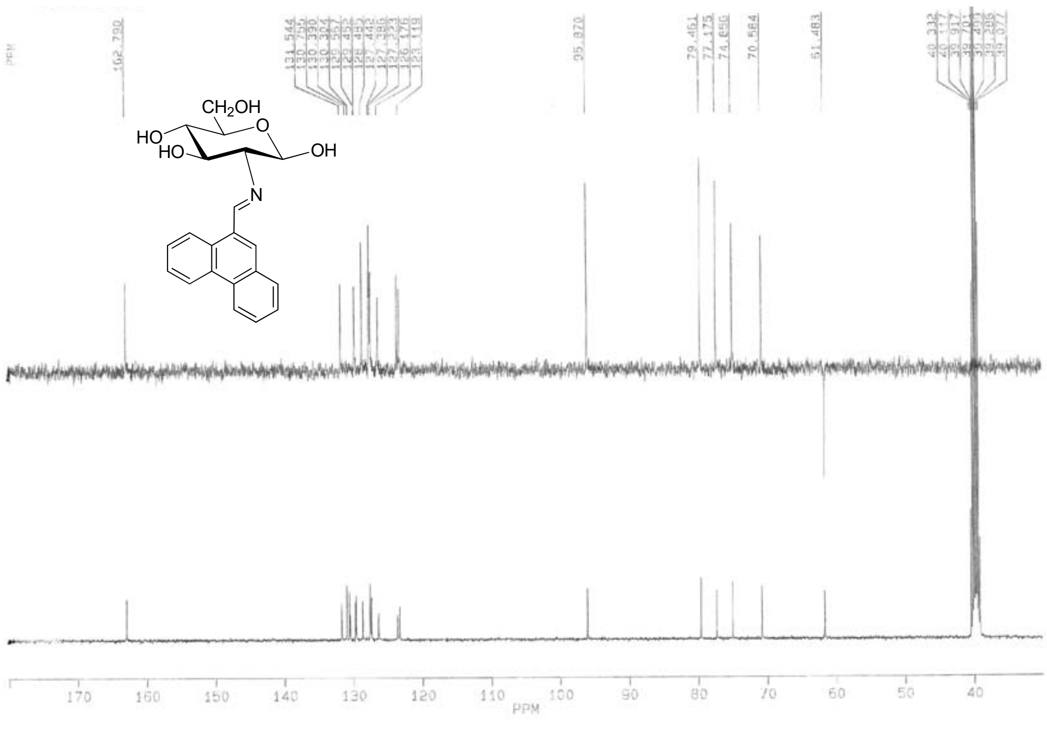

Figure S130. $^{13}C\{^{1}H\}$ NMR (top: DEPT) spectra of **55** in DMSO-*d₆*.

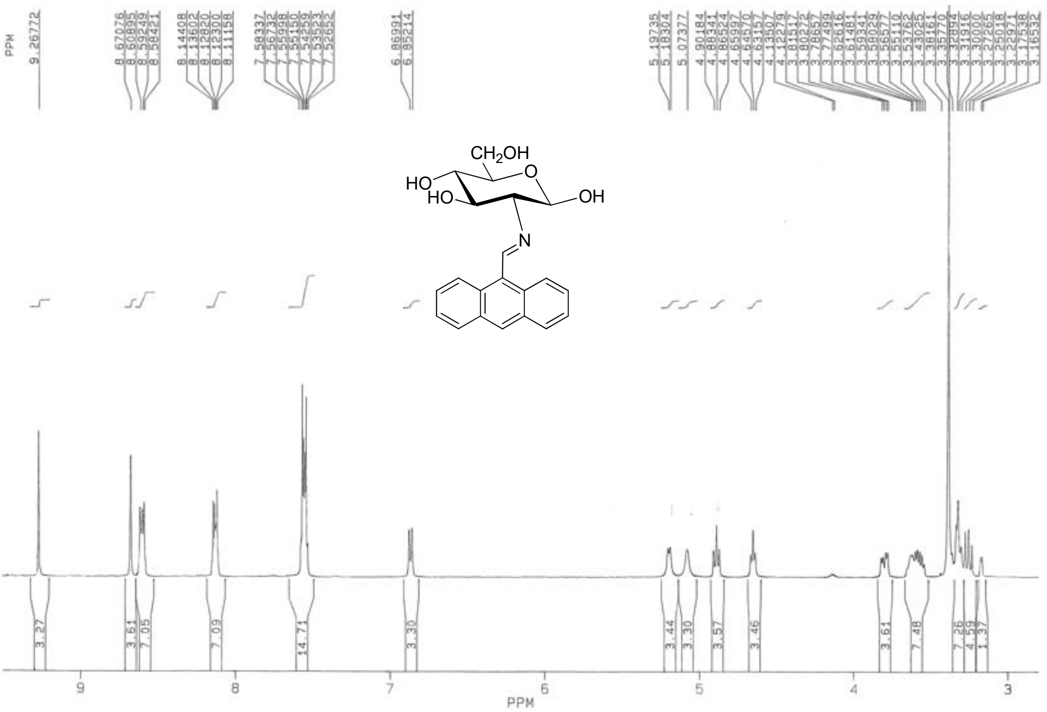

Figure S131. ^{1}H NMR spectrum of **56** in DMSO-*d₆*

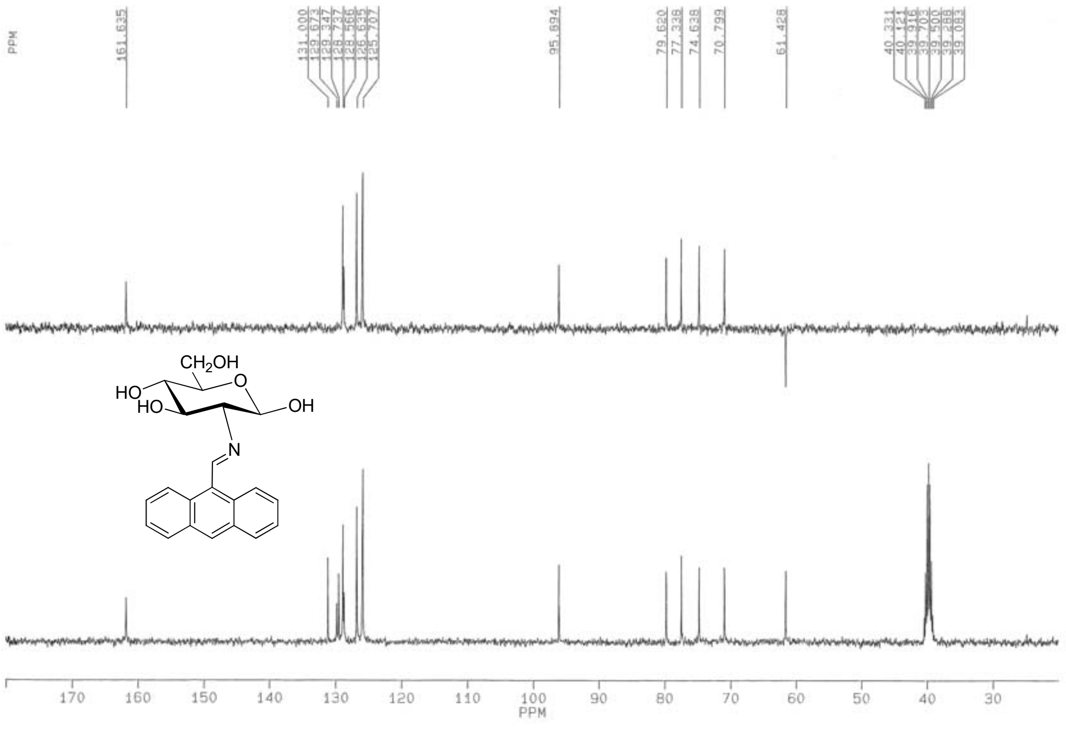

Figure S132. ^{13}C{^{1}H} NMR (top: DEPT) spectra of **56** in DMSO-*d₆*.

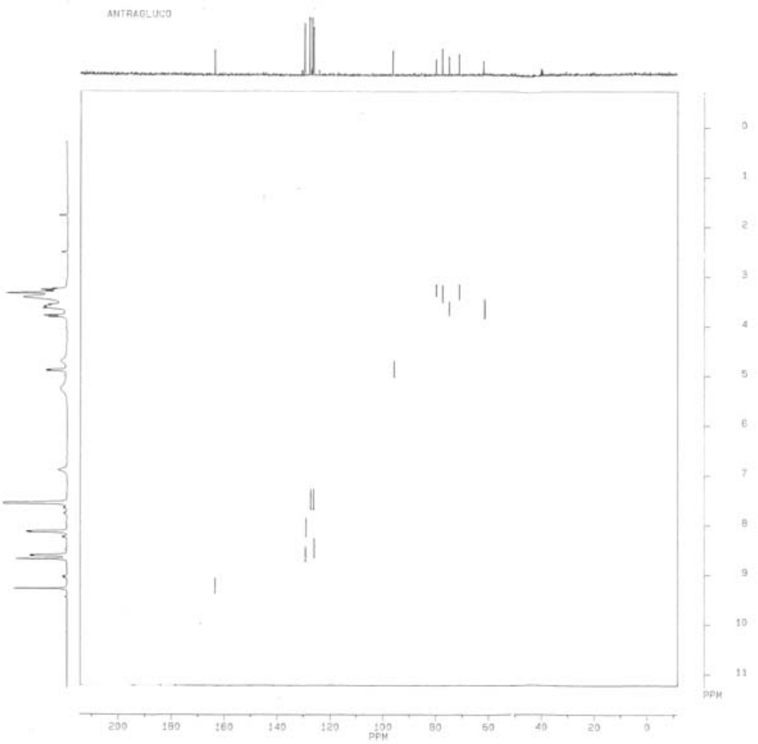

Figure S133. HMQC spectrum of **56** in DMSO-d_6

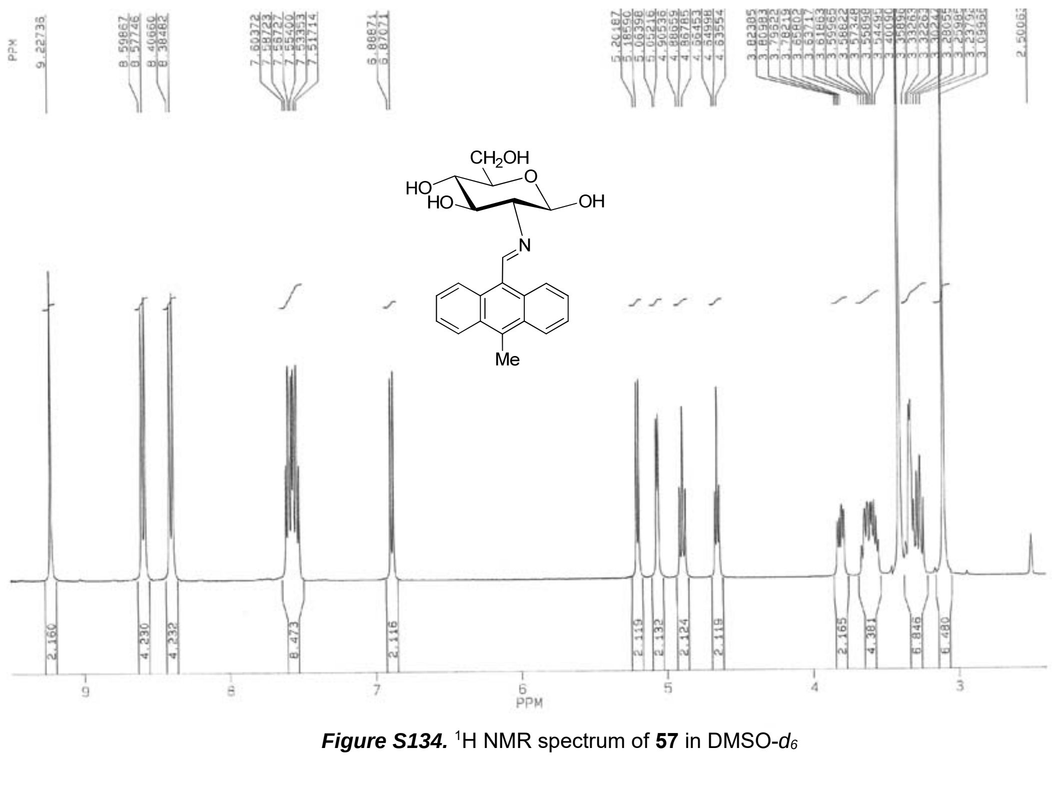

Figure S134. ^{1}H NMR spectrum of **57** in DMSO-d_6

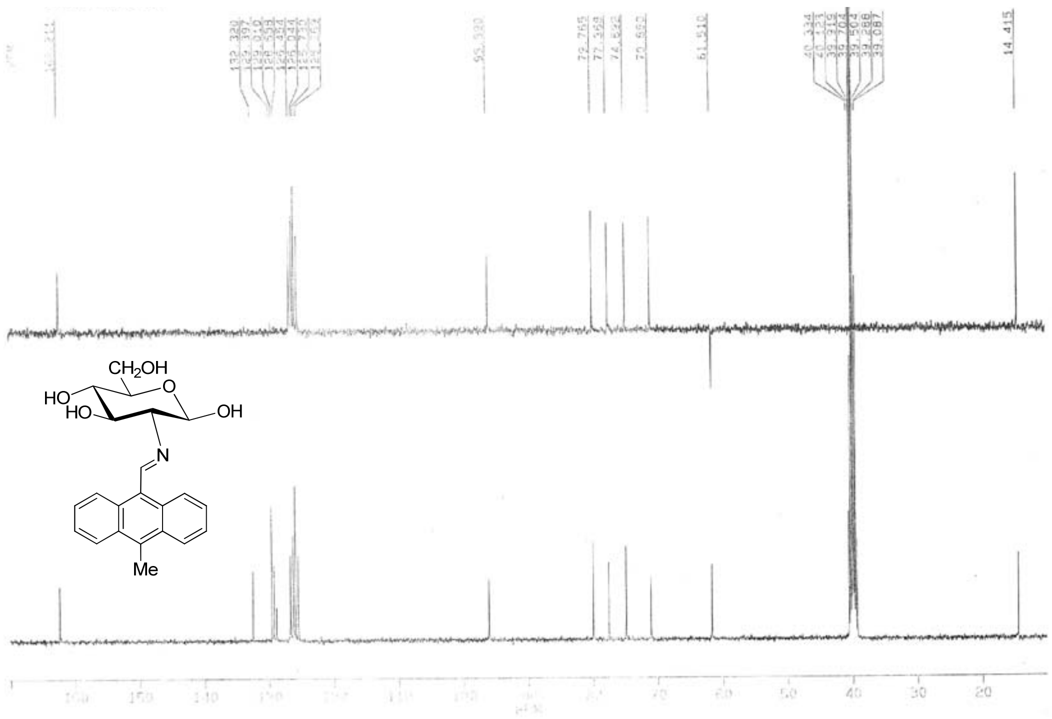

Figure S135. $^{13}C\{^1H\}$ NMR (top: DEPT) spectra of **57** in DMSO-d_6.

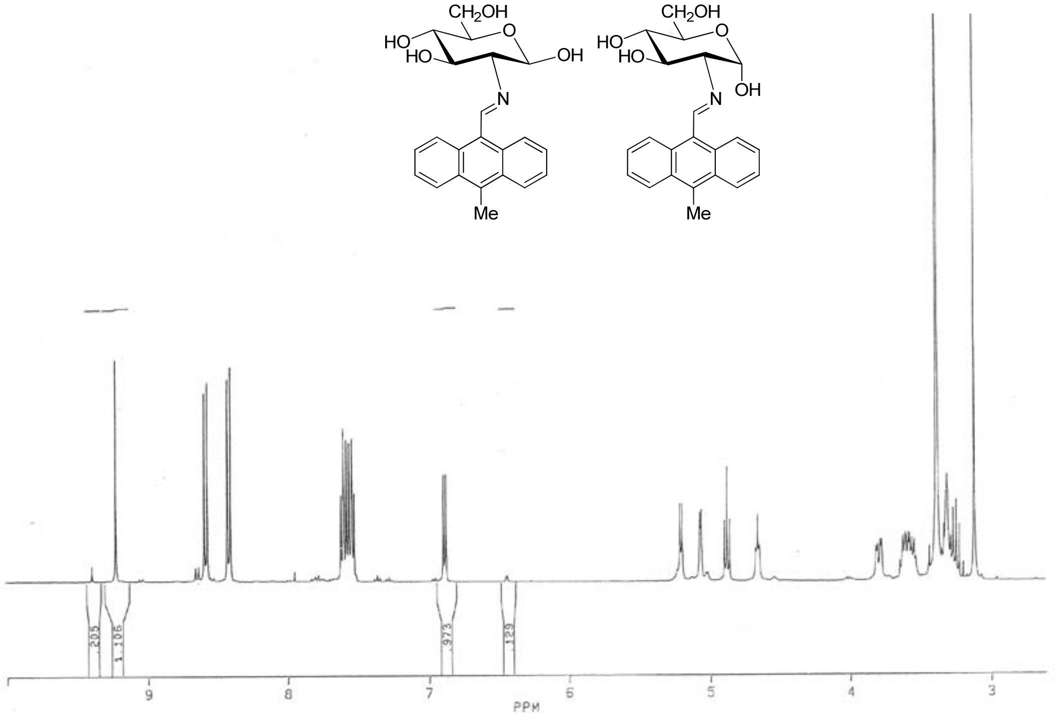

Figure S136. ^1H NMR spectrum of **57** and **113** in DMSO-d_6

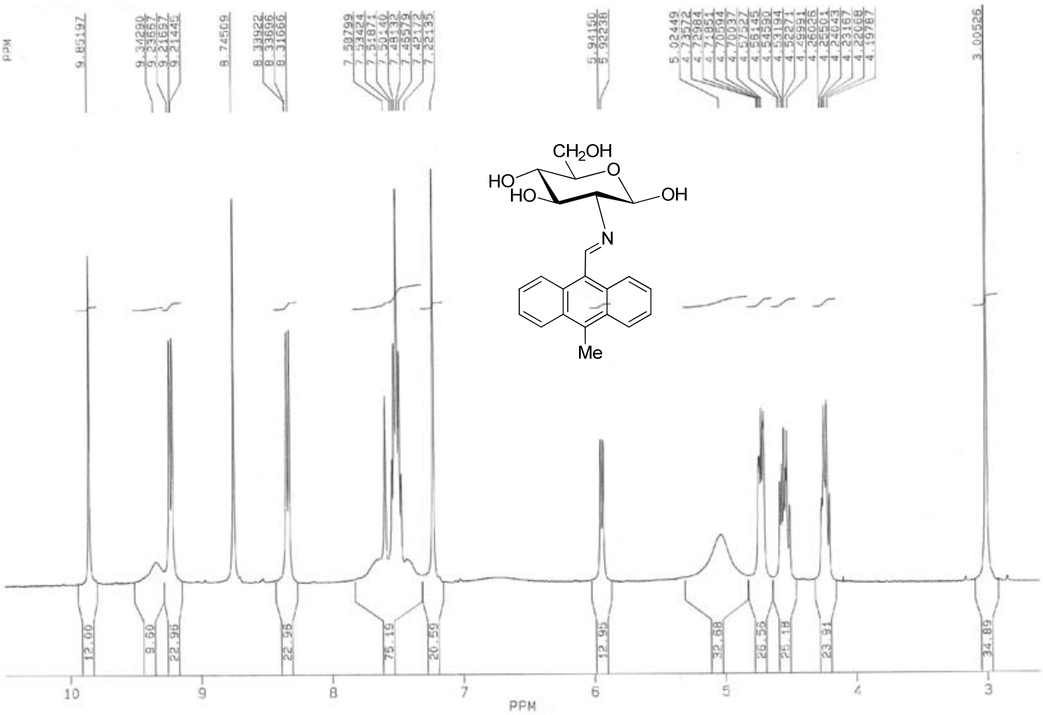

Figure S137. ^{1}H NMR spectrum of **57** in pyridine-*d*$_5$

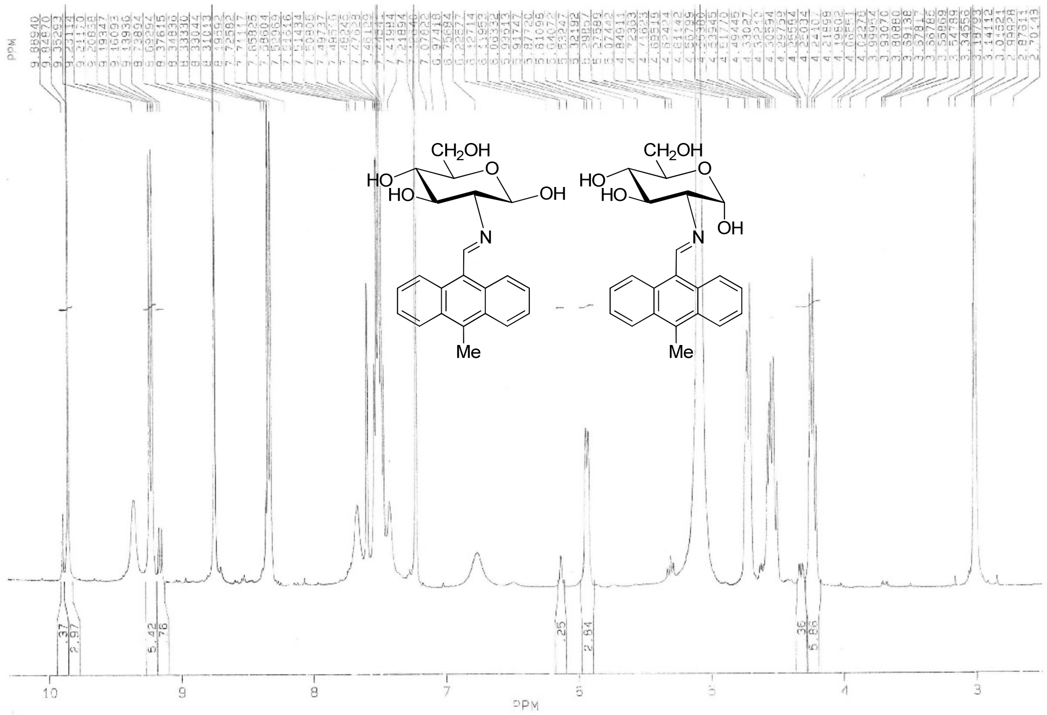

Figure S138. ^{1}H NMR spectrum of **57** and **113** in pyridine-*d*$_5$

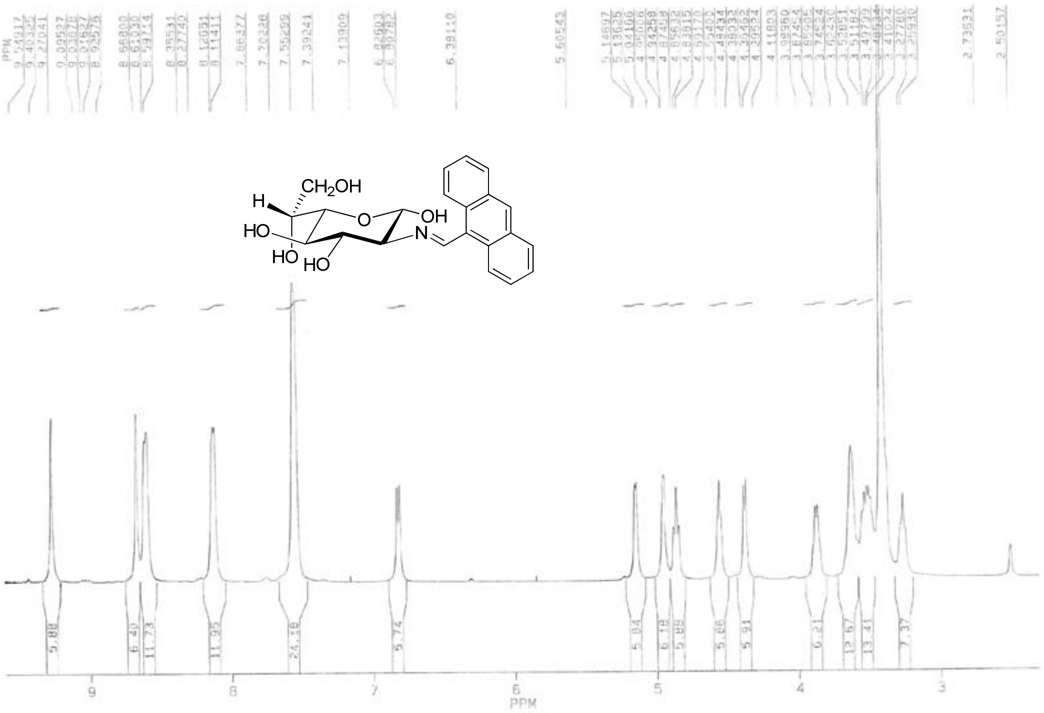

***Figure* S139.** ^{1}H NMR spectrum of **58** in DMSO-d_6

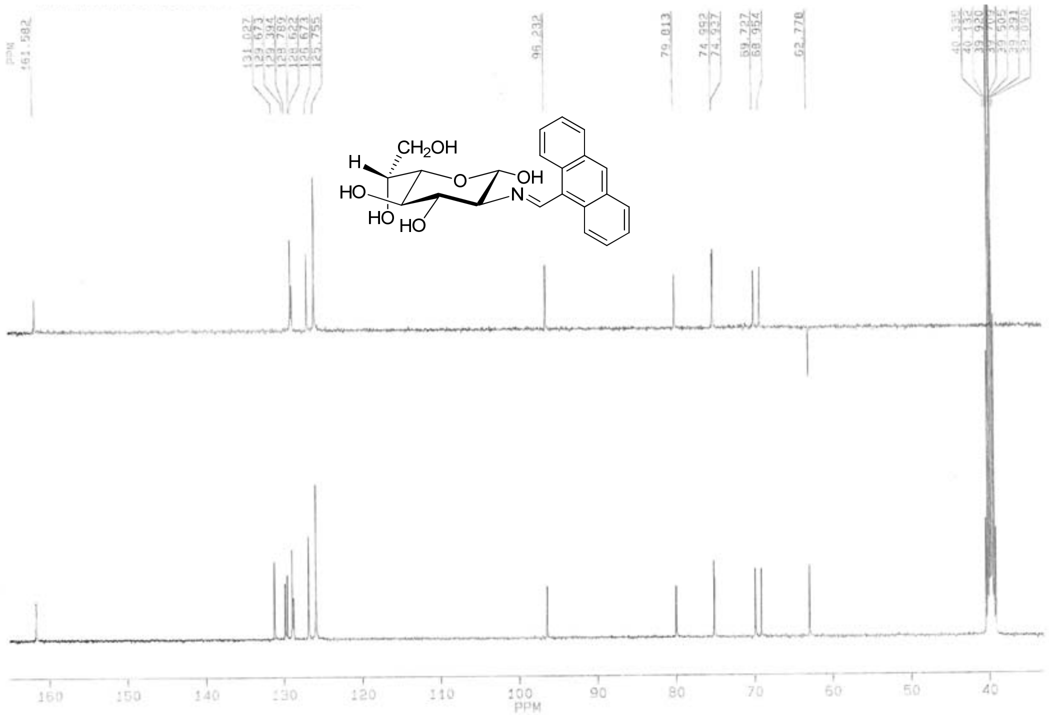

Figure S140. ^{13}C{^{1}H} NMR (top: DEPT) spectra of **58** in DMSO-d_6.

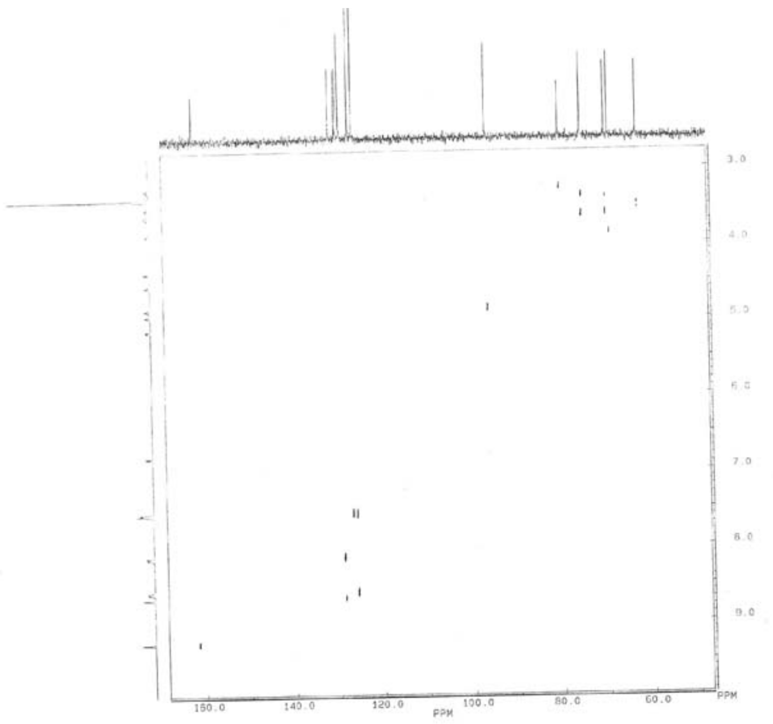

Figure S141. HMQC spectrum of **58** in DMSO-d_6

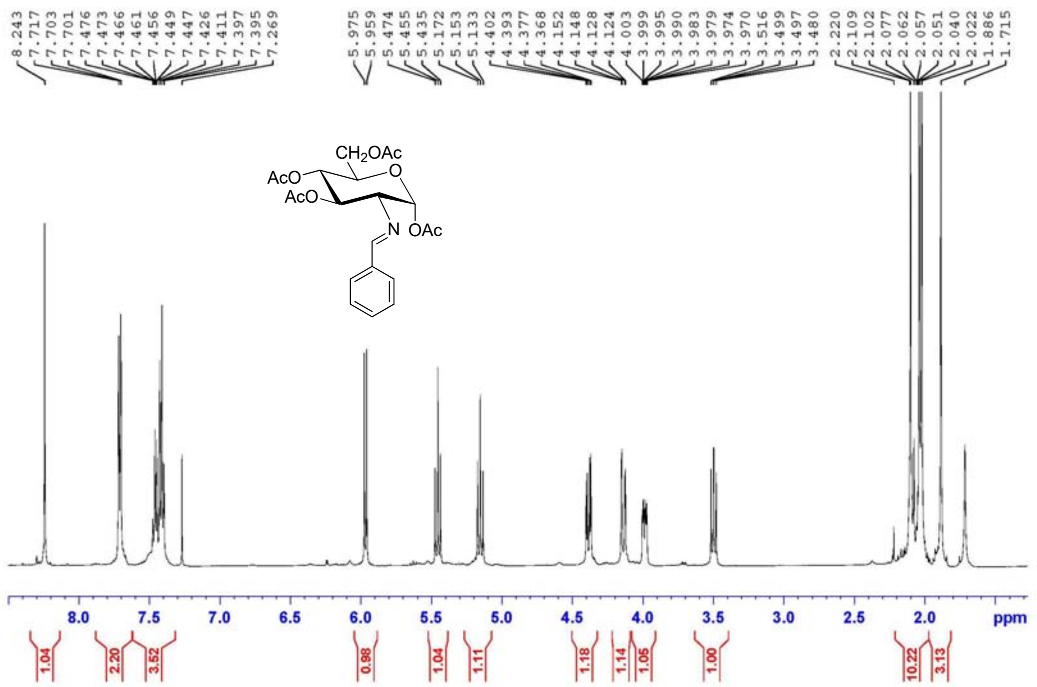

Figure S142. ^{1}H NMR spectrum of **63** in $CDCl_3$

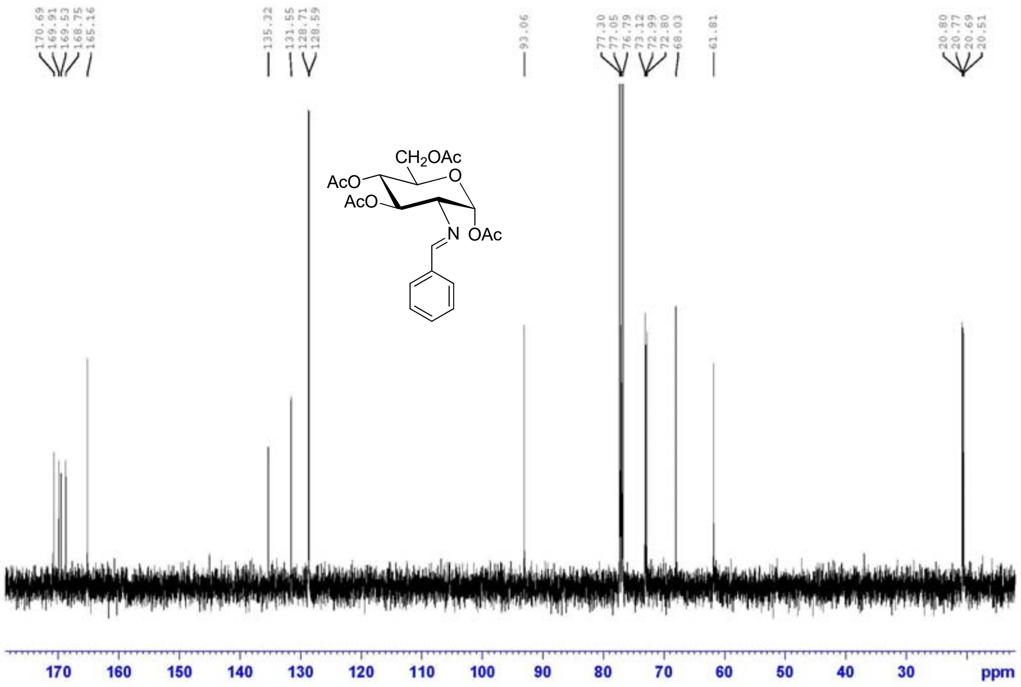

Figure S143. ^{13}C {^{1}H} NMR spectrum of **63** in $CDCl_3$

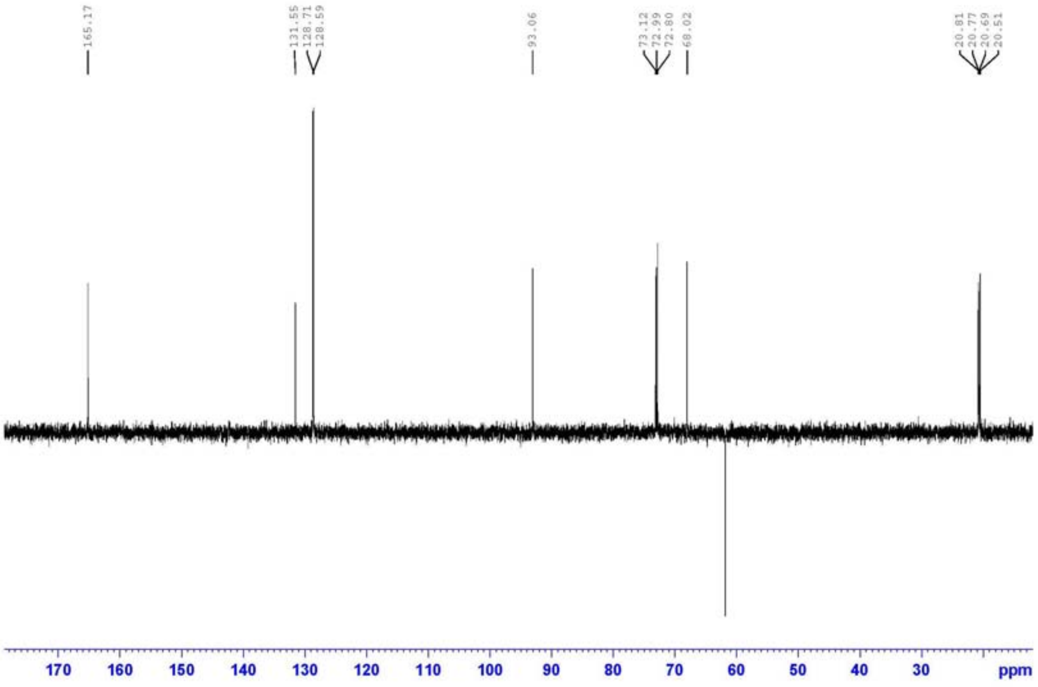

Figure S144. DEPT spectrum of **63** in $CDCl_3$

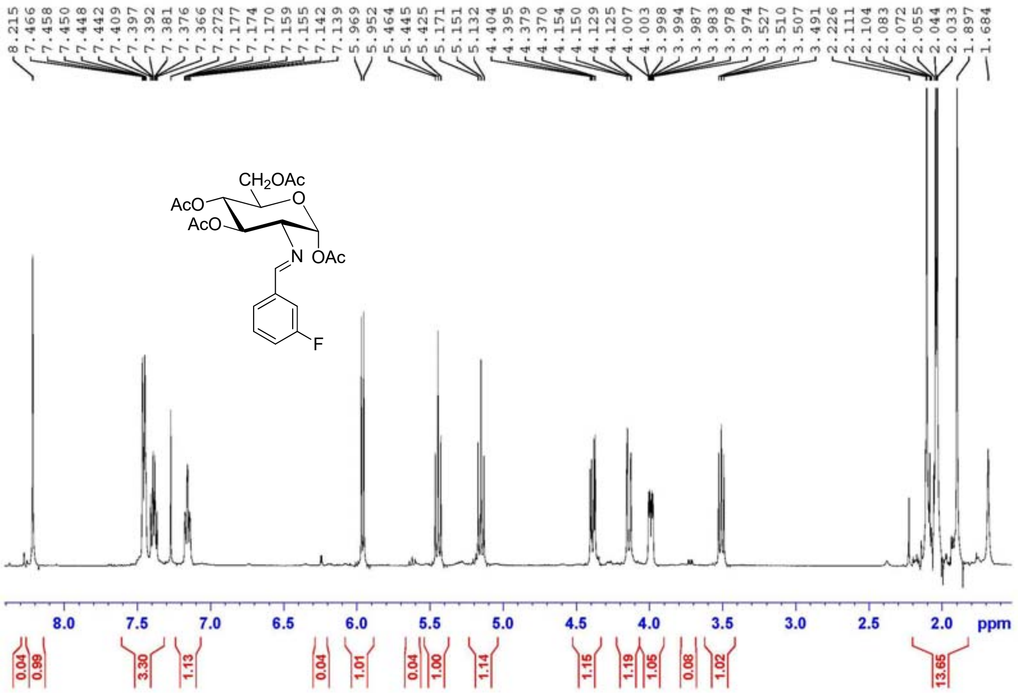

Figure S145. ^{1}H NMR spectrum of **64** in $CDCl_3$

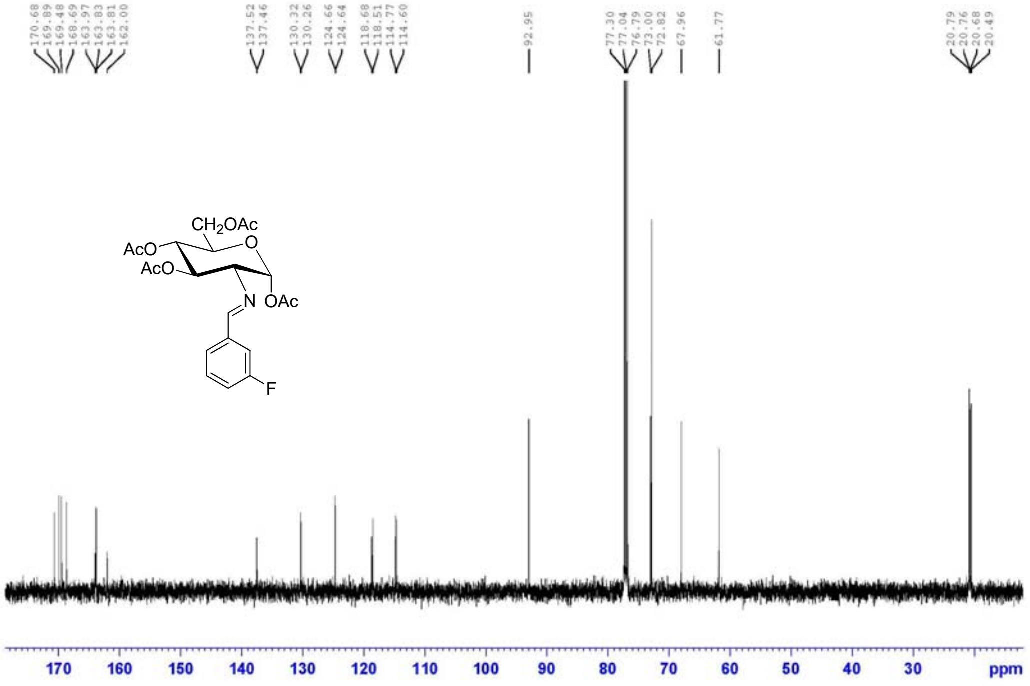

Figure S146. ^{13}C {^{1}H} NMR spectrum of **64** in $CDCl_3$

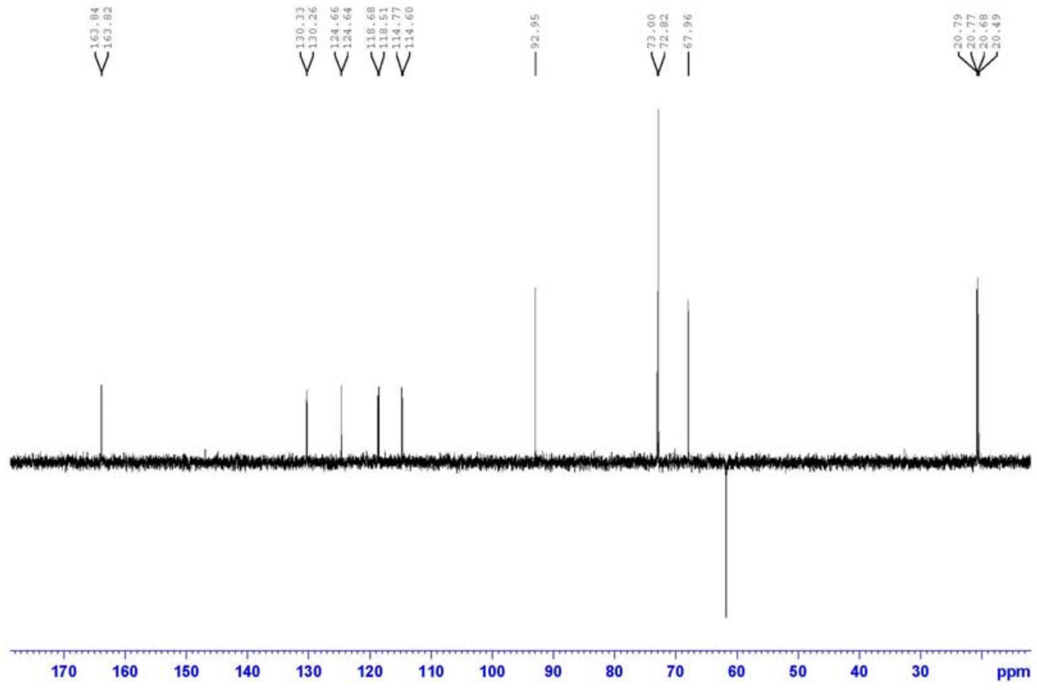

Figure S147. DEPT spectrum of **64** in $CDCl_3$

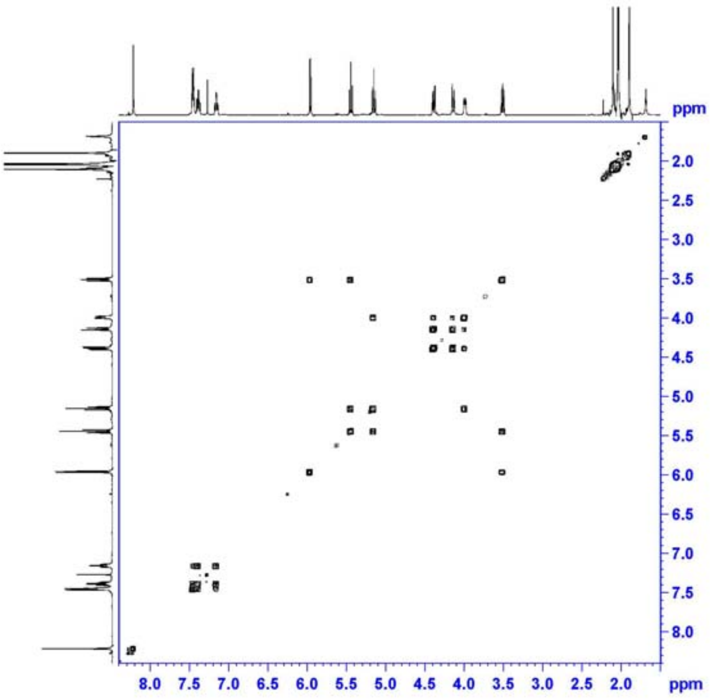

Figure S148. COSY spectrum of **64** in $CDCl_3$

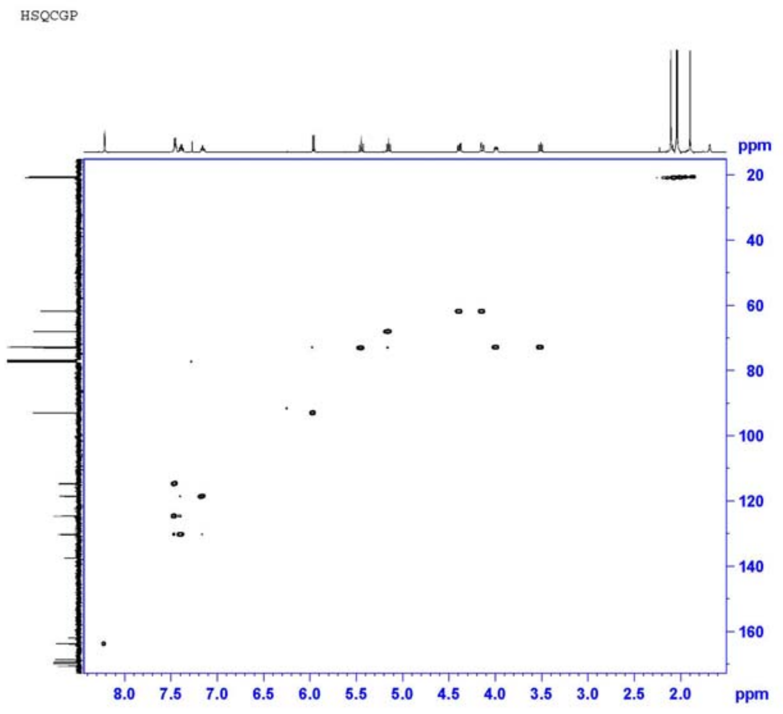

Figure S149. HSQC spectrum of **64** in $CDCl_3$

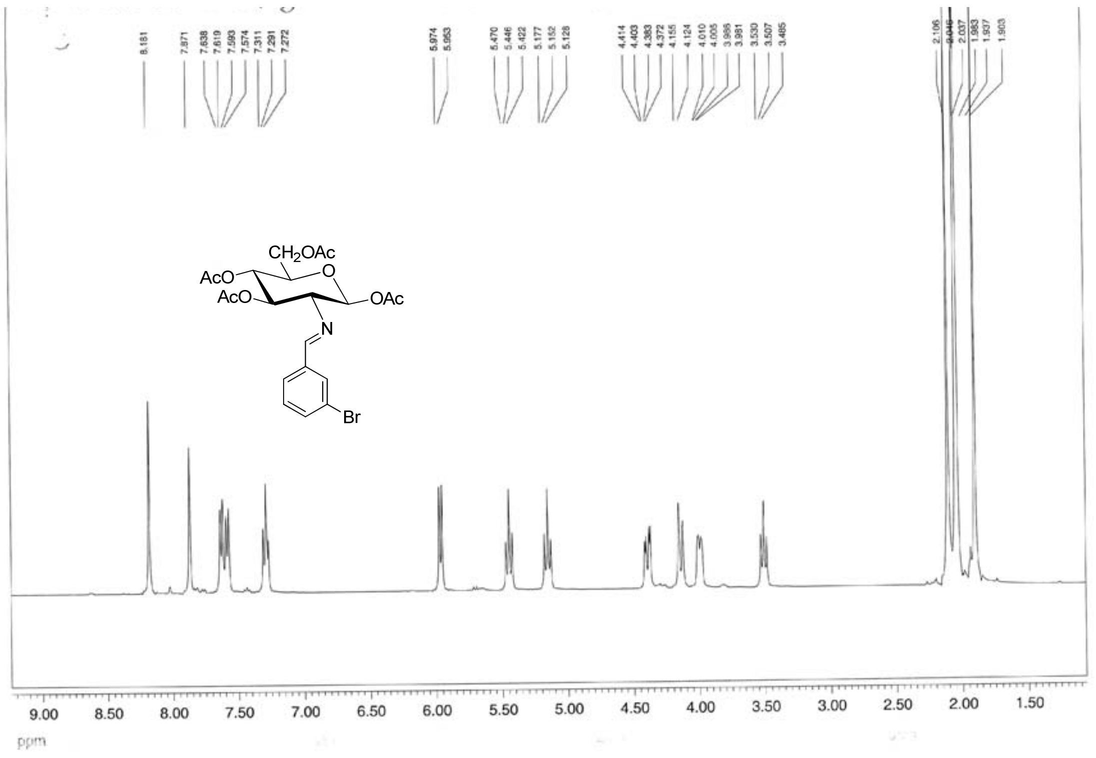

Figure S150. ^{1}H NMR spectrum of **65** in $CDCl_3$

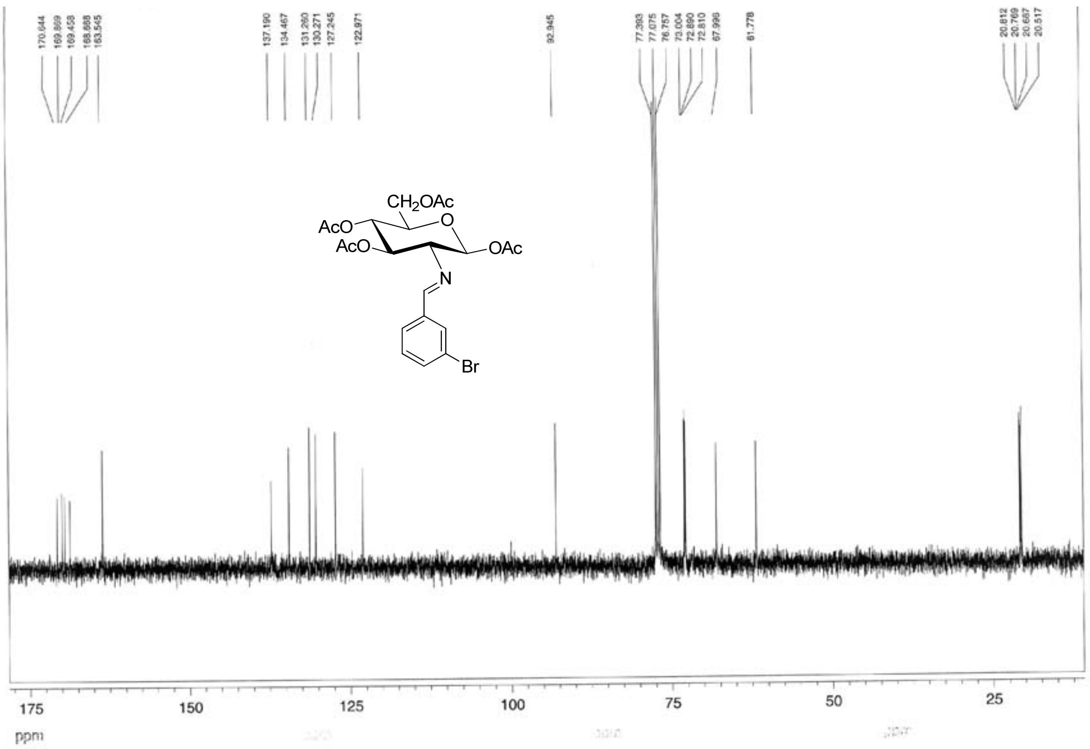

Figure S151. $^{13}C\ \{^{1}H\}$ NMR spectrum of **65** in $CDCl_3$.

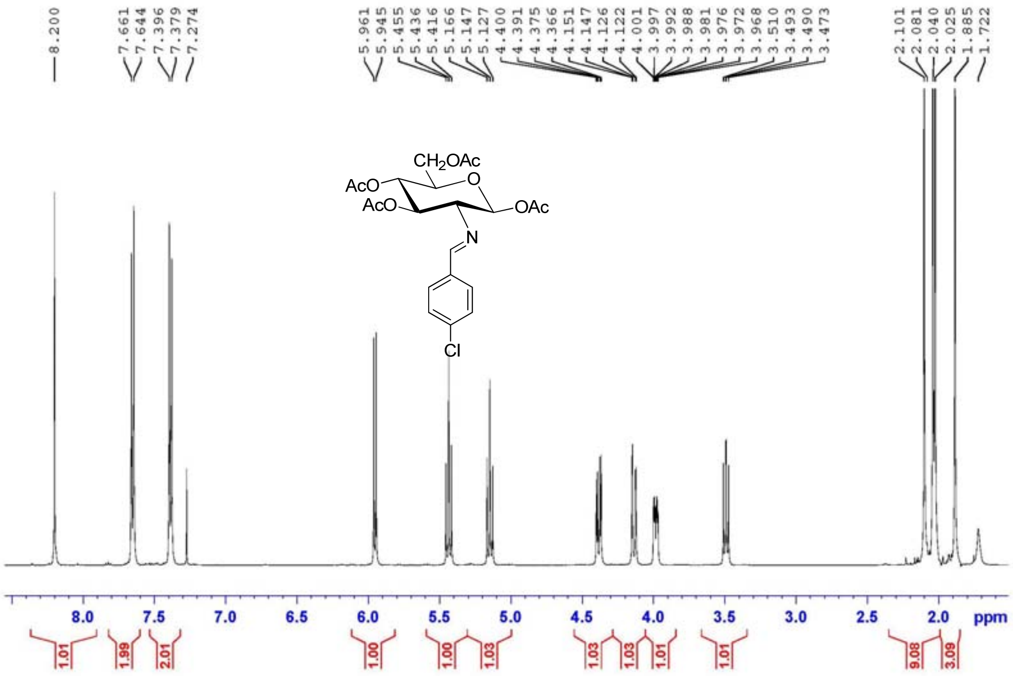

Figure S152. ^{1}H NMR spectrum of **66** in $CDCl_3$

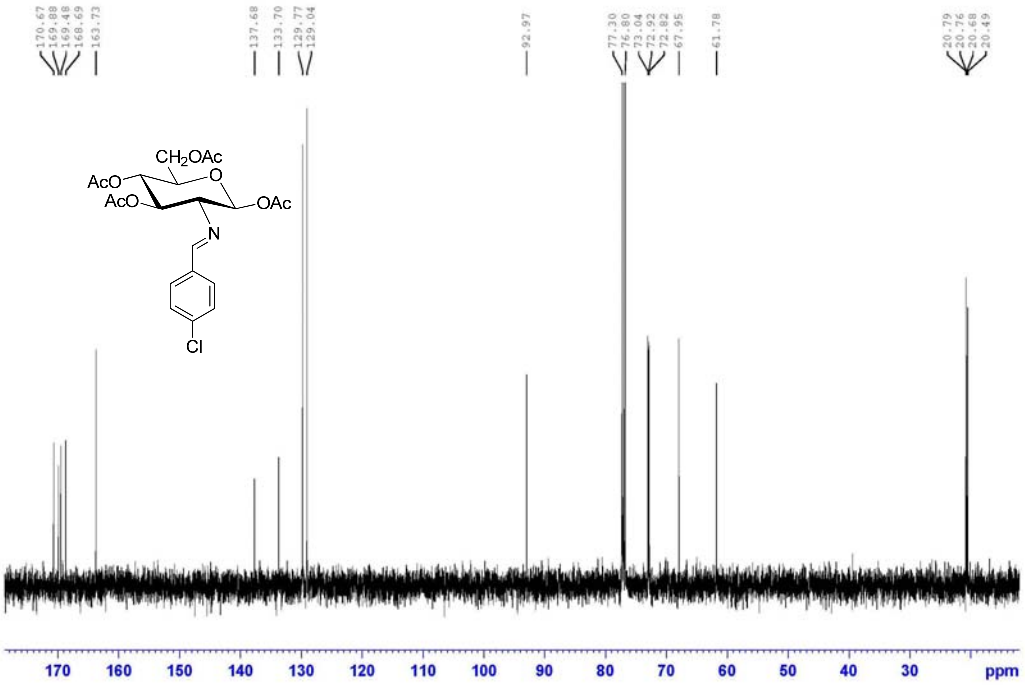

Figure S153. ^{13}C {^{1}H} NMR spectrum of **66** in $CDCl_3$

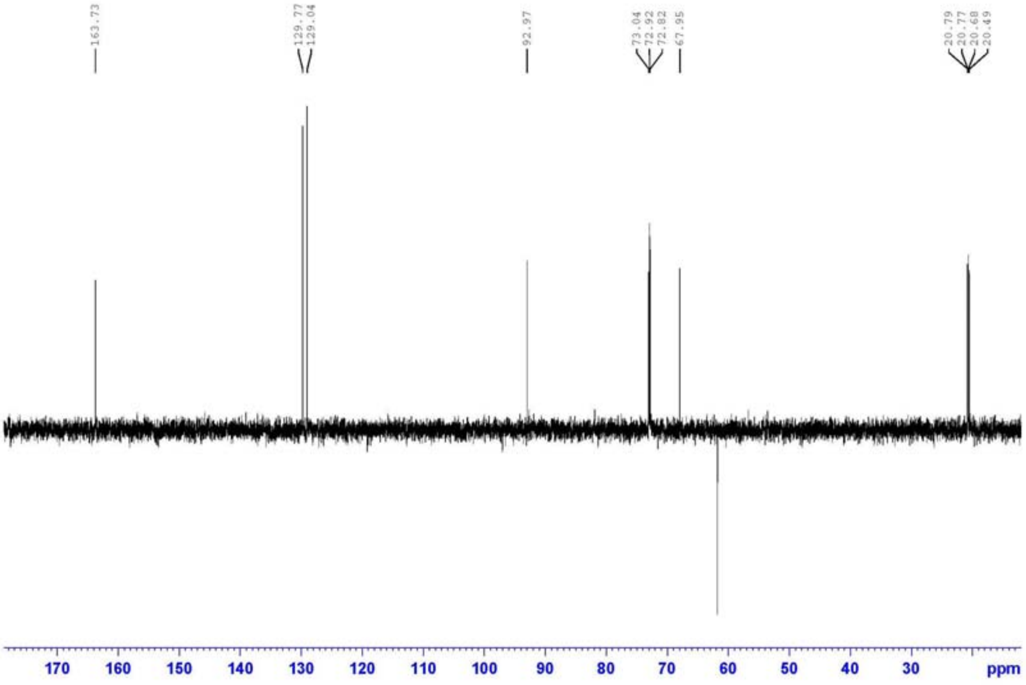

Figure S154. DEPT spectrum of **66** in $CDCl_3$

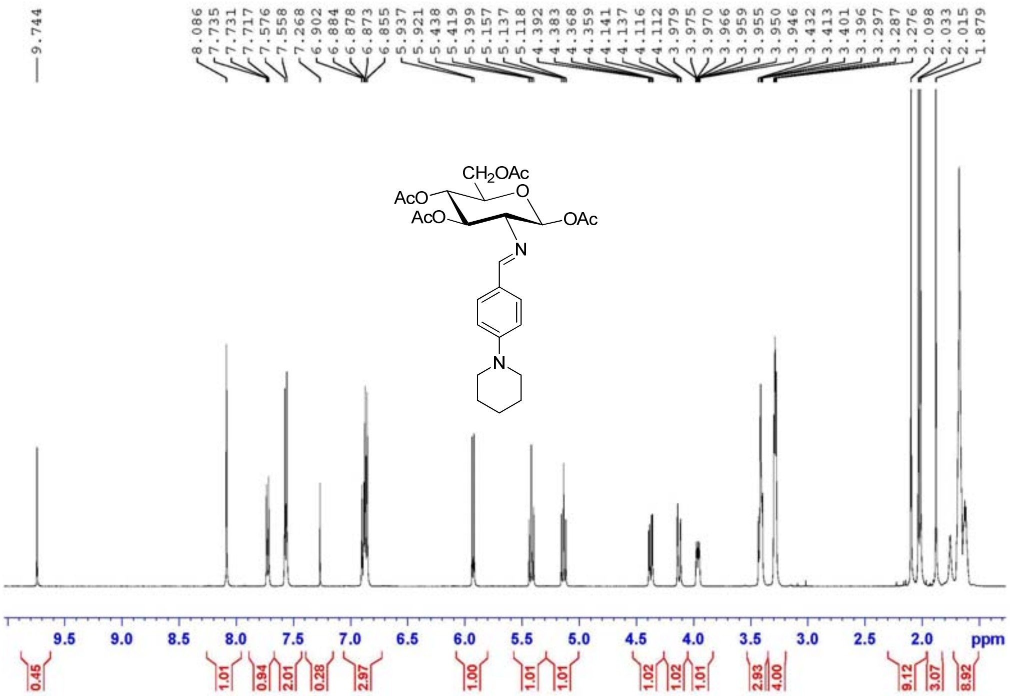

Figure S155. ^{1}H NMR spectrum of **67** in $CDCl_3$

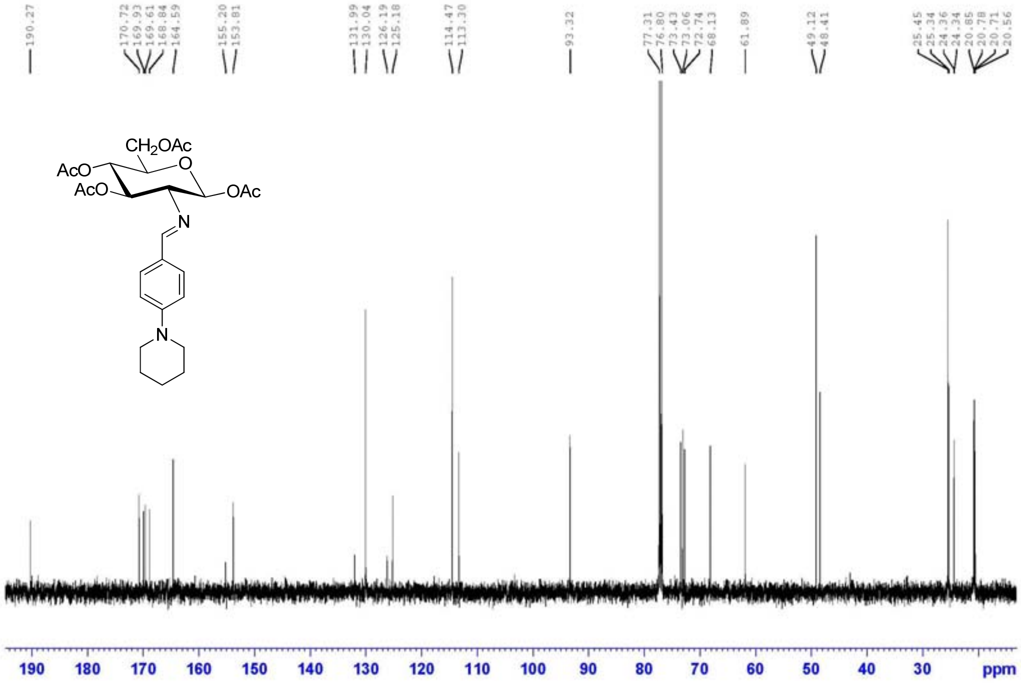

Figure S156. ^{13}C {^{1}H} NMR spectrum of **67** in $CDCl_3$

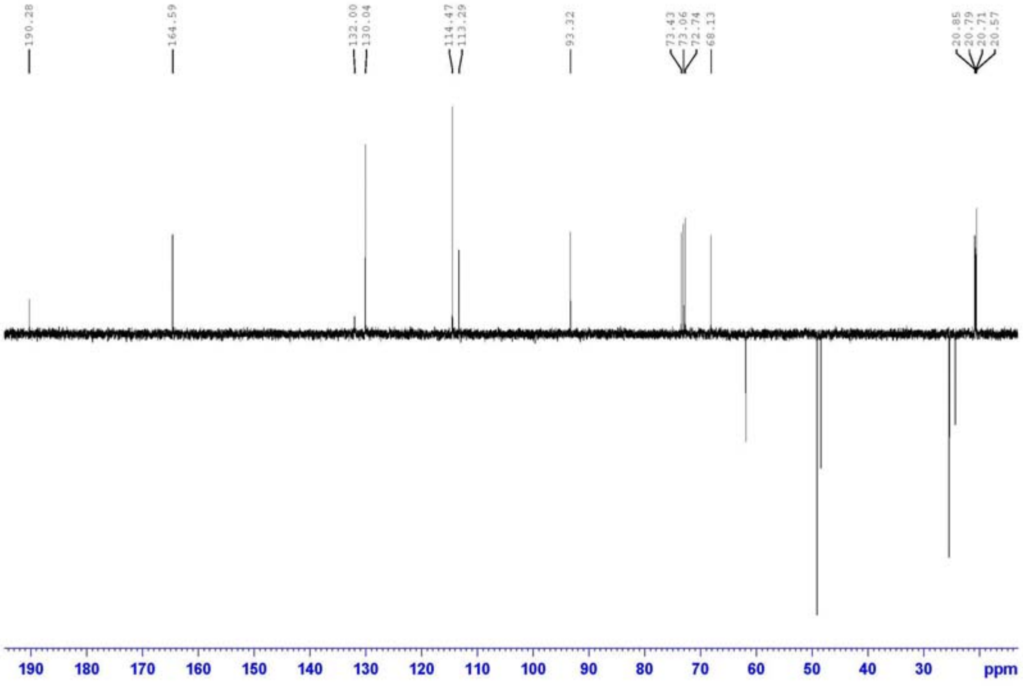

Figure S157. DEPT spectrum of **67** in $CDCl_3$

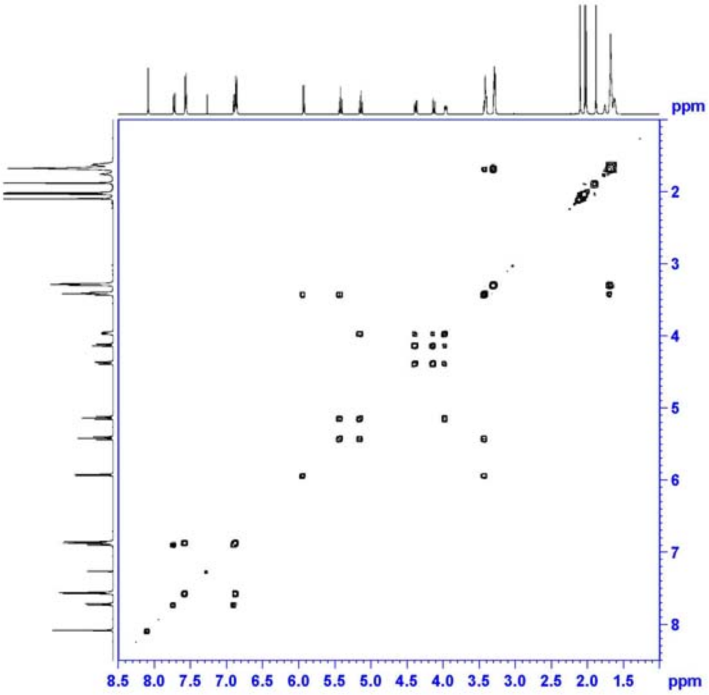

Figure S158. COSY spectrum of **67** in $CDCl_3$

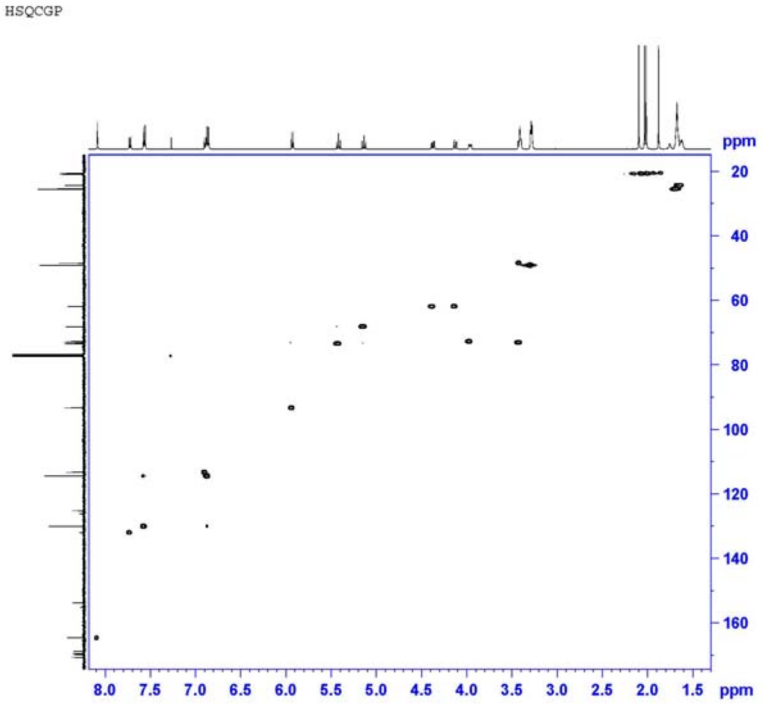

Figure S159. HSQC spectrum of **67** in $CDCl_3$

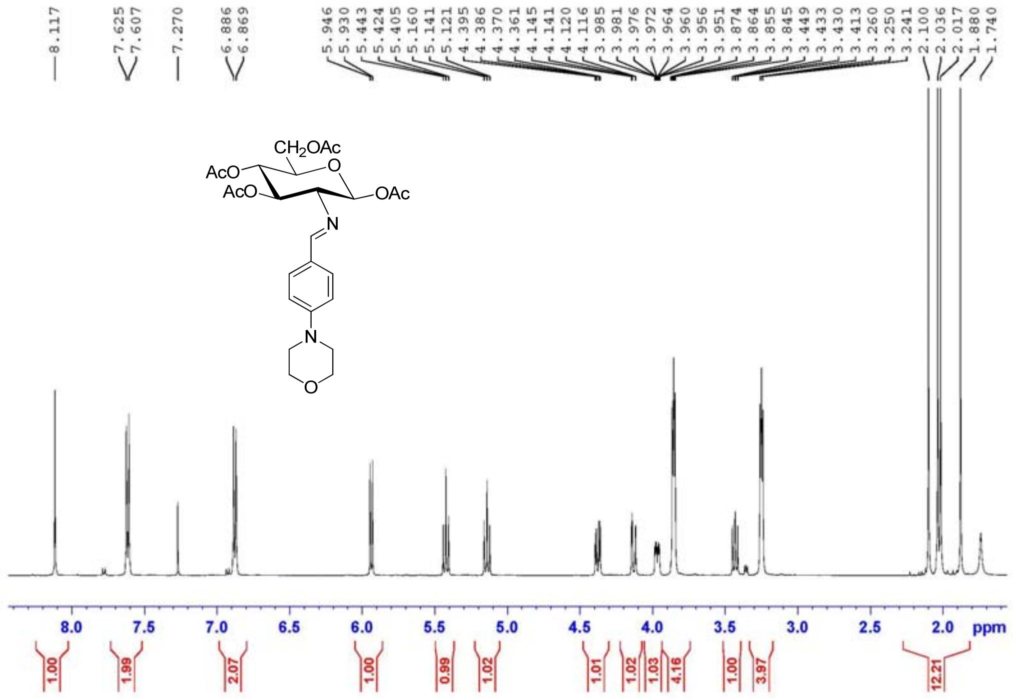

Figure S160. ^{1}H NMR spectrum of **68** in $CDCl_3$

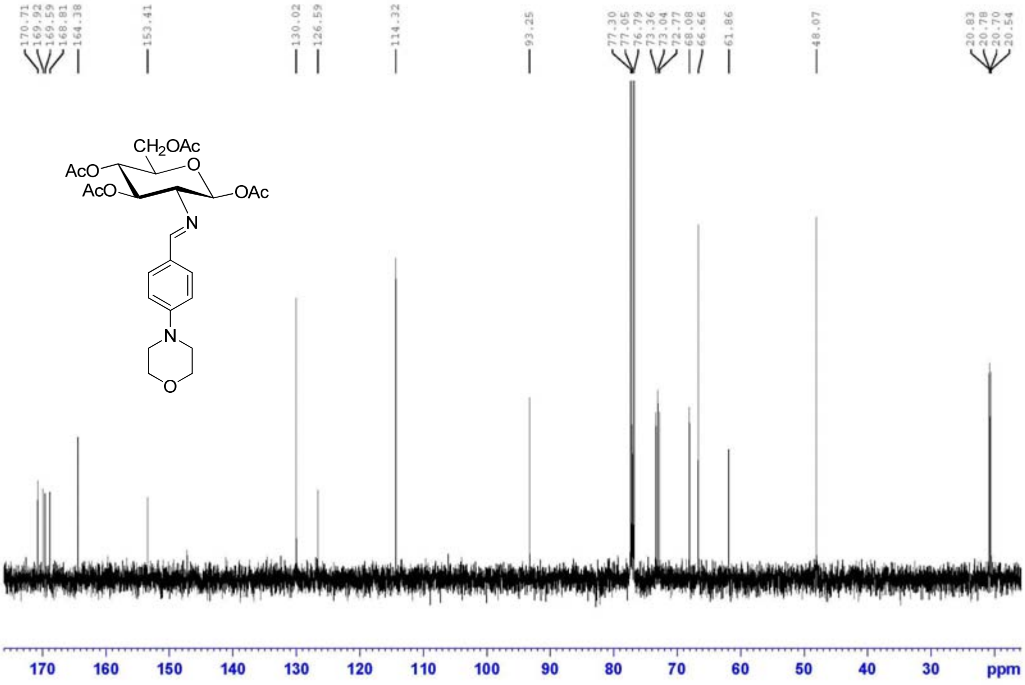

Figure S161. ^{13}C {^{1}H} NMR spectrum of **68** in $CDCl_3$

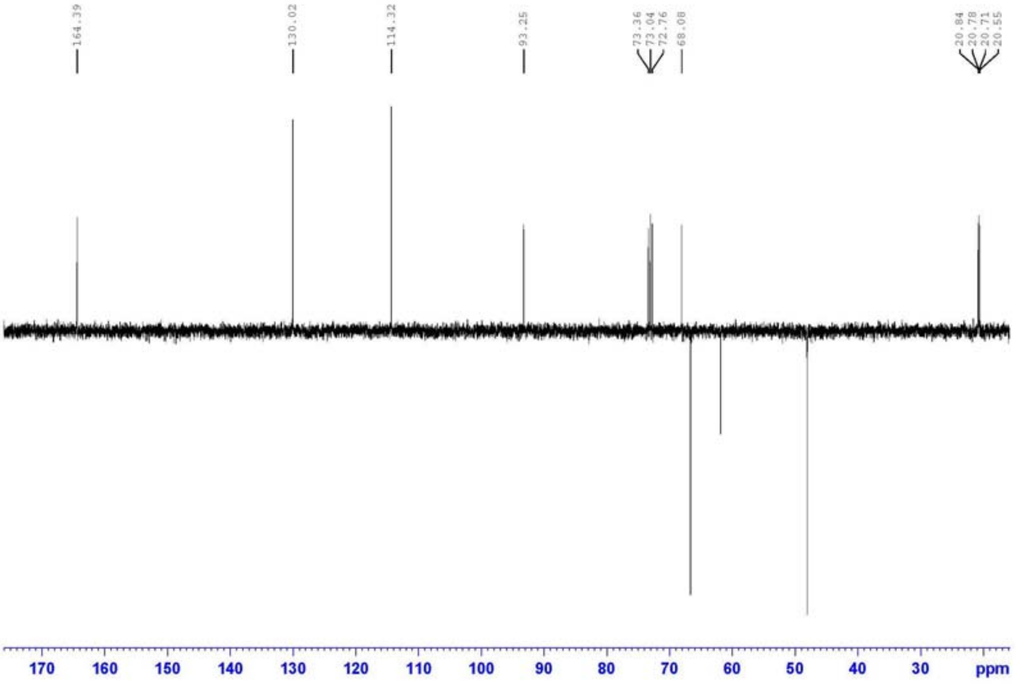

Figure S162. DEPT spectrum of **68** in $CDCl_3$

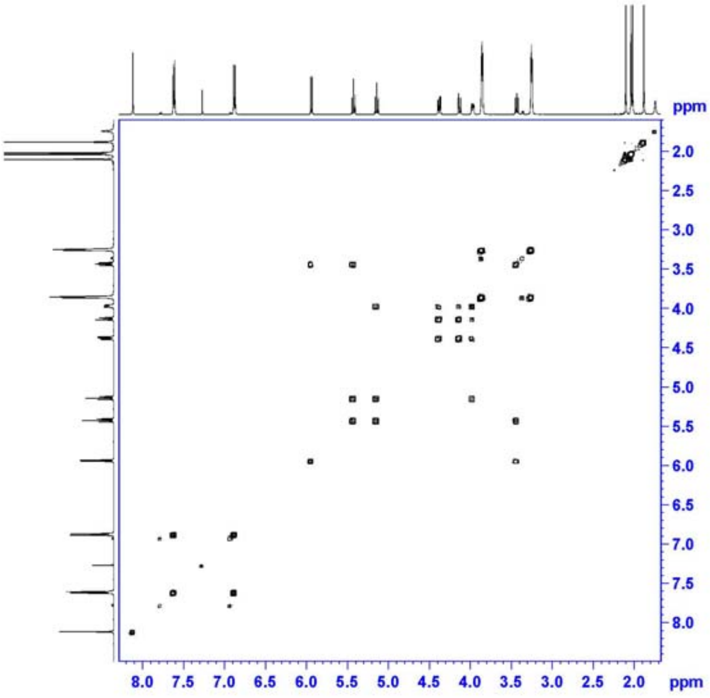

Figure S163. COSY spectrum of **68** in $CDCl_3$

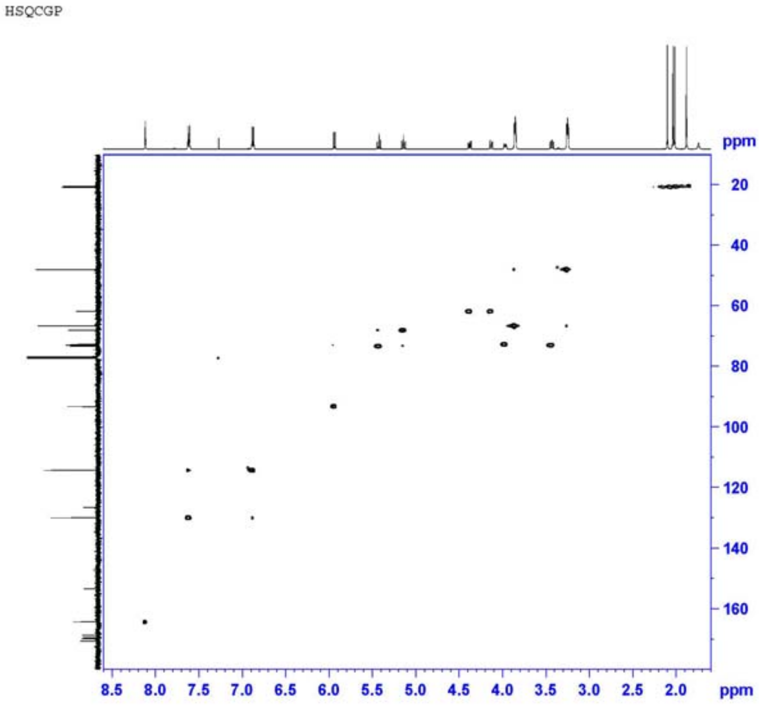

Figure S164. HSQC spectrum of **68** in $CDCl_3$

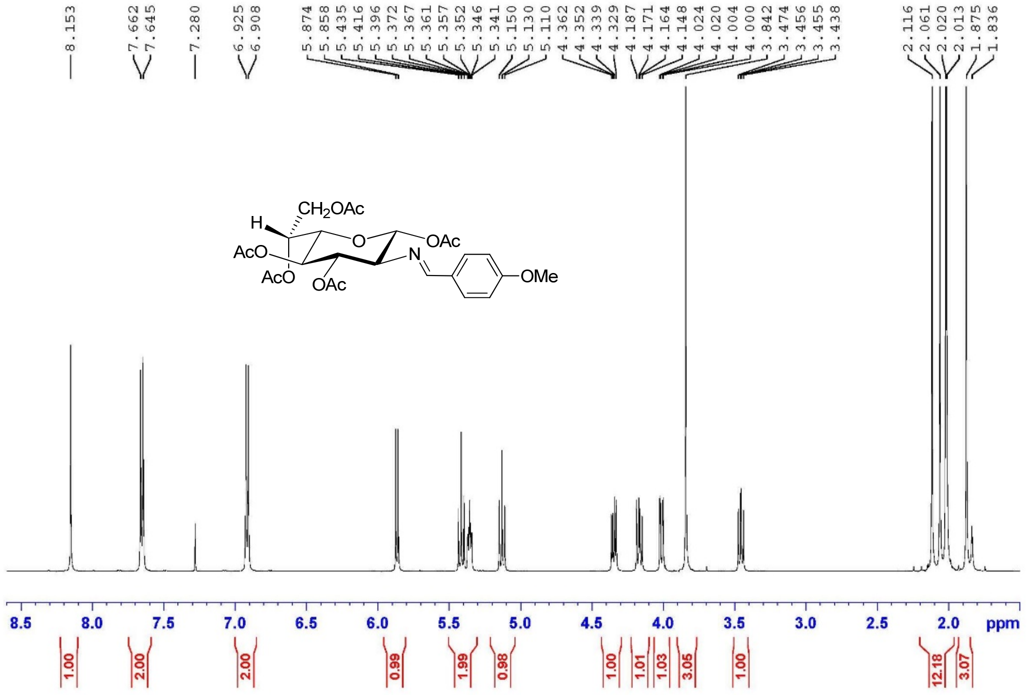

Figure S165. ^{1}H NMR spectrum of **69** in $CDCl_3$.

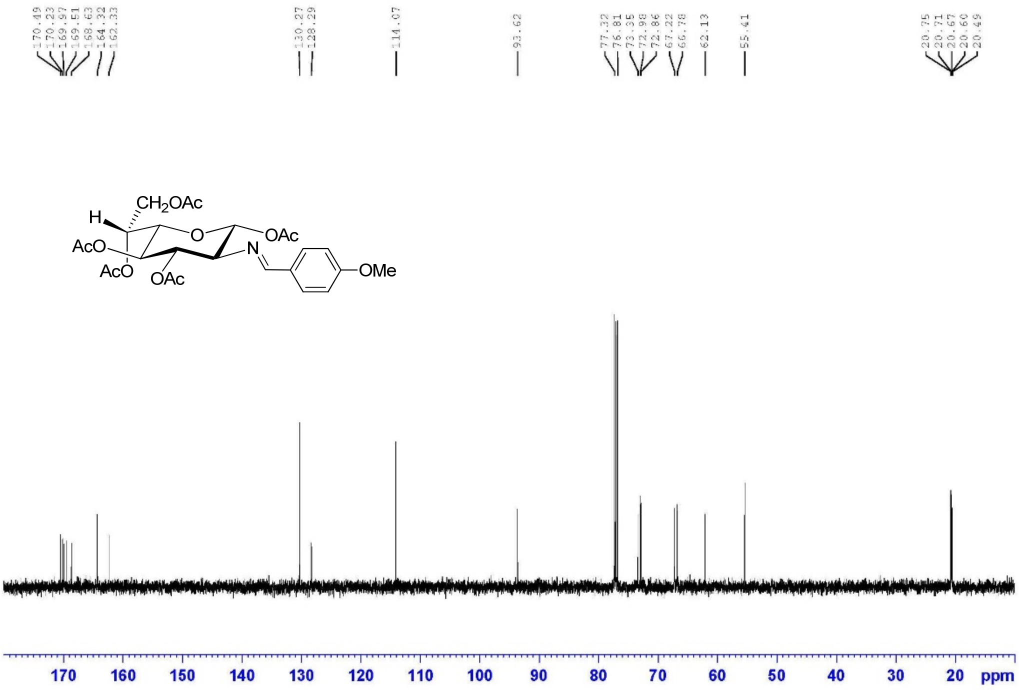

Figure S166. ^{13}C {^{1}H} NMR spectrum of **69** in $CDCl_3$.

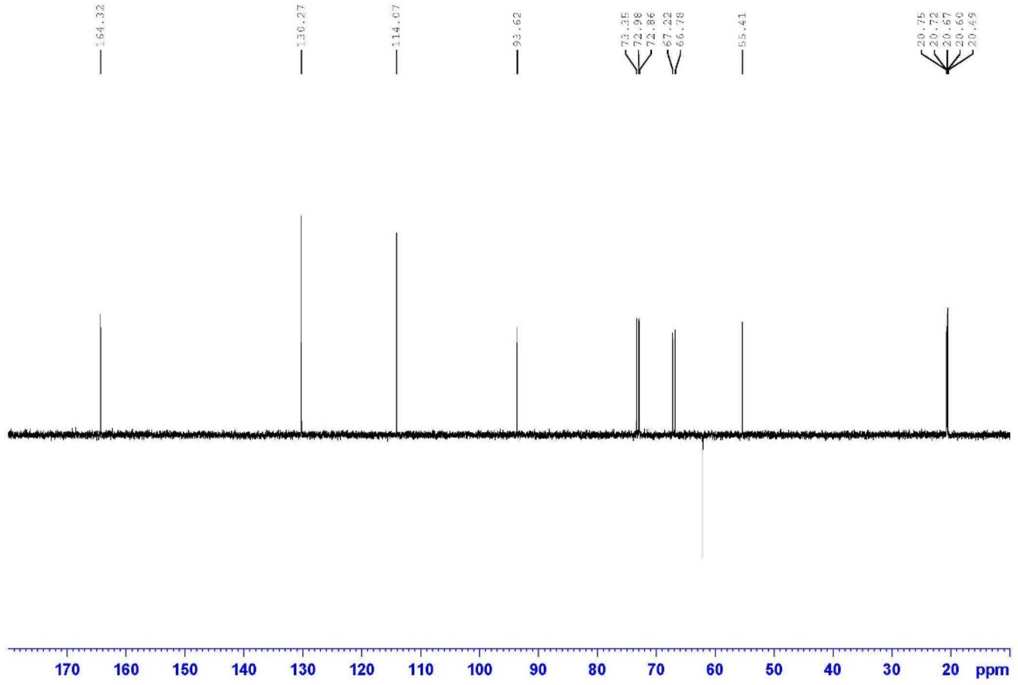

Figure S167. DEPT spectrum of **69** in $CDCl_3$.

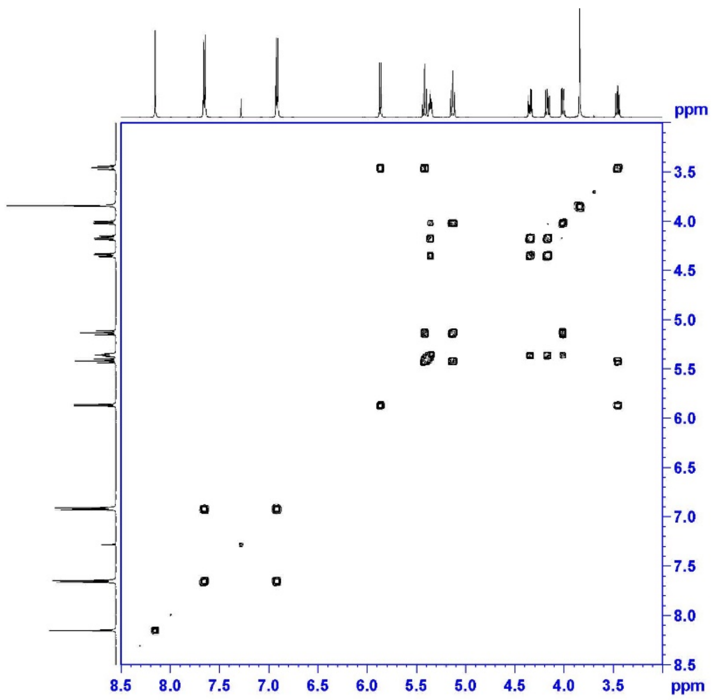

Figure S168. HMQC spectrum of **69** in $CDCl_3$.

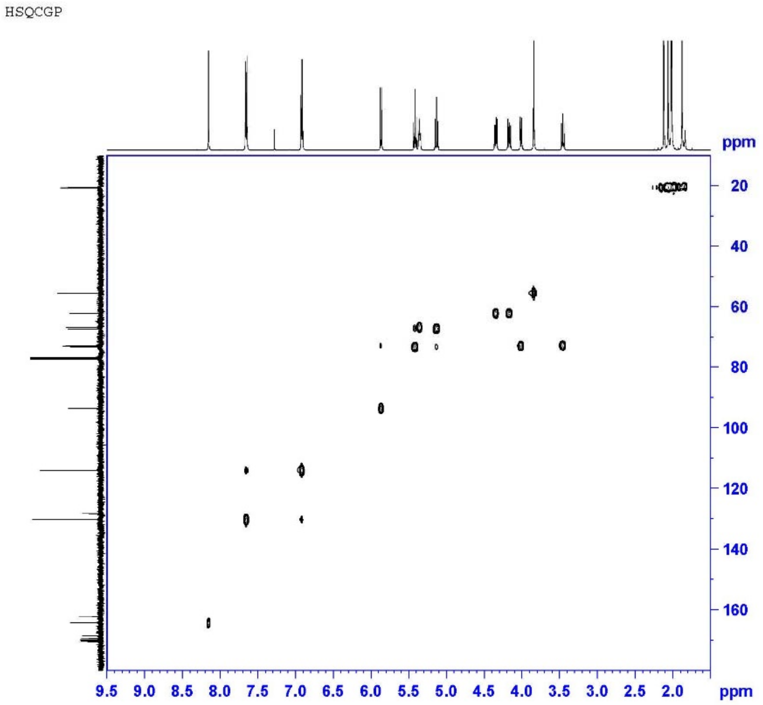

Figure S169. HMQC spectrum of **69** in $CDCl_3$.

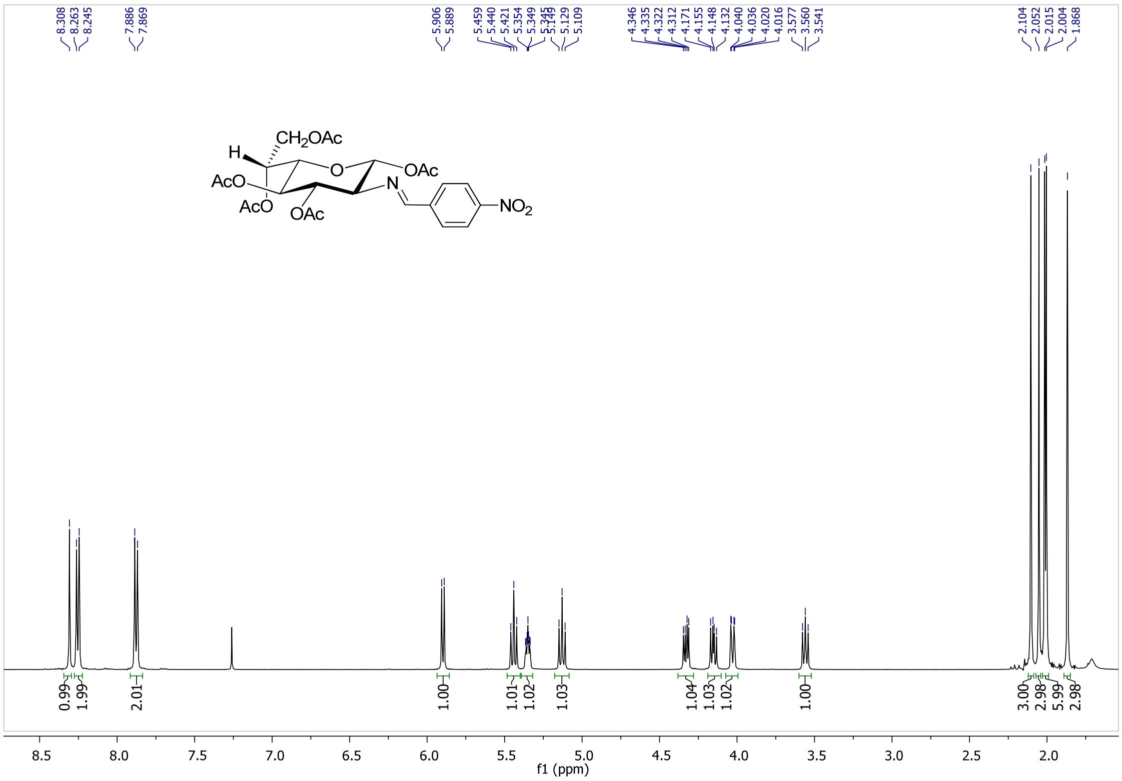

Figure S170. ^{1}H NMR spectrum of **70** in $CDCl_3$

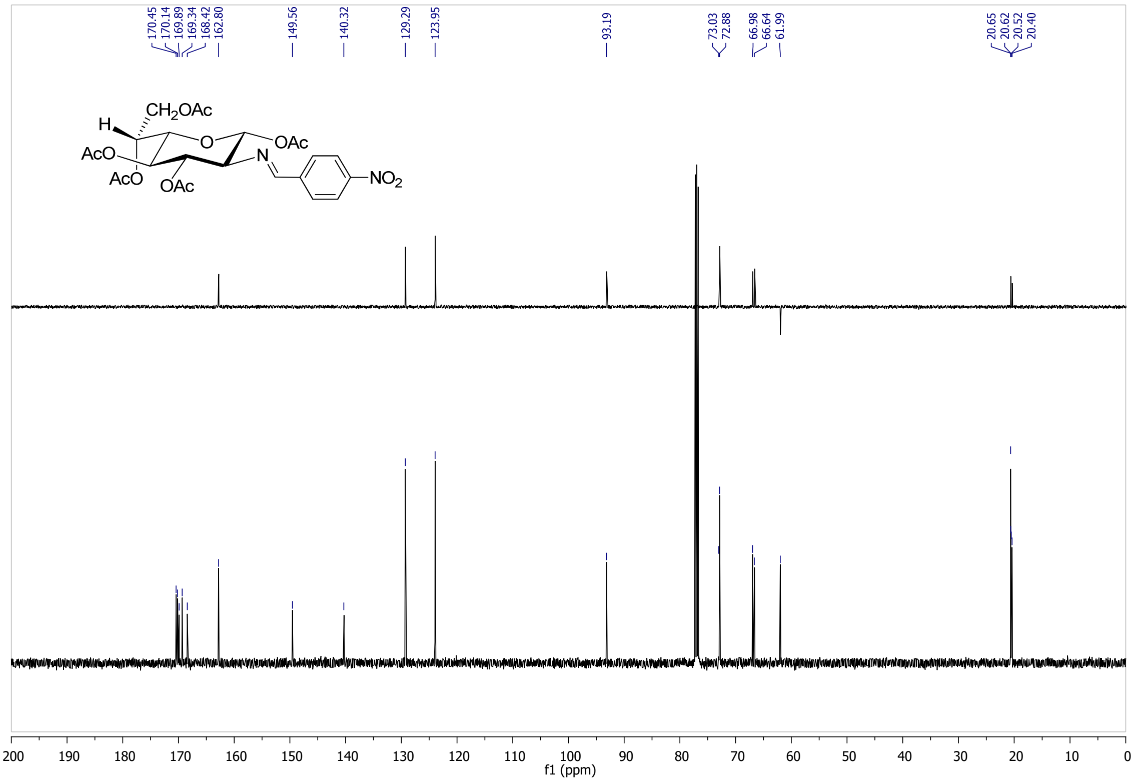

Figure S171. $^{13}C\{^1H\}$ NMR (top: DEPT) spectra of **70** in $CDCl_3$.

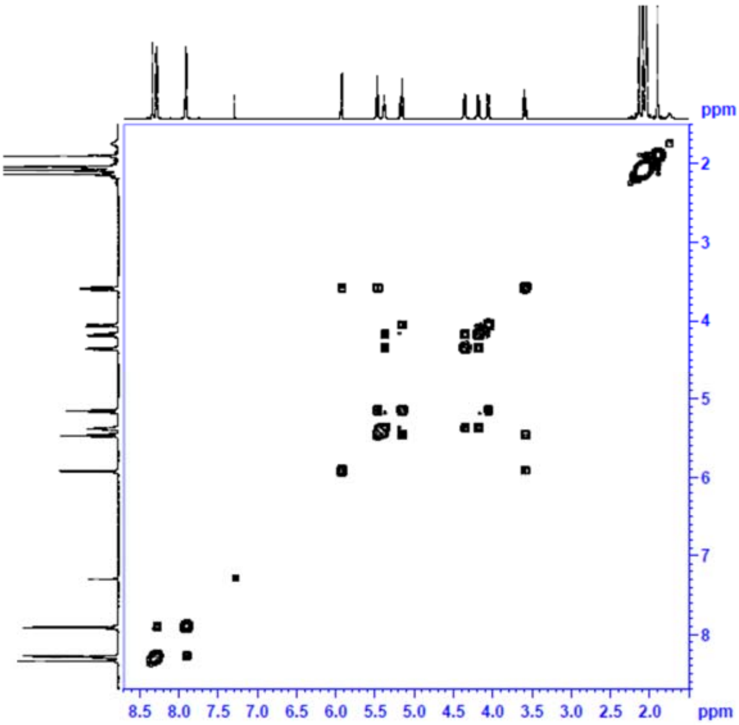

Figure S172. COSY spectrum of **70** in $CDCl_3$

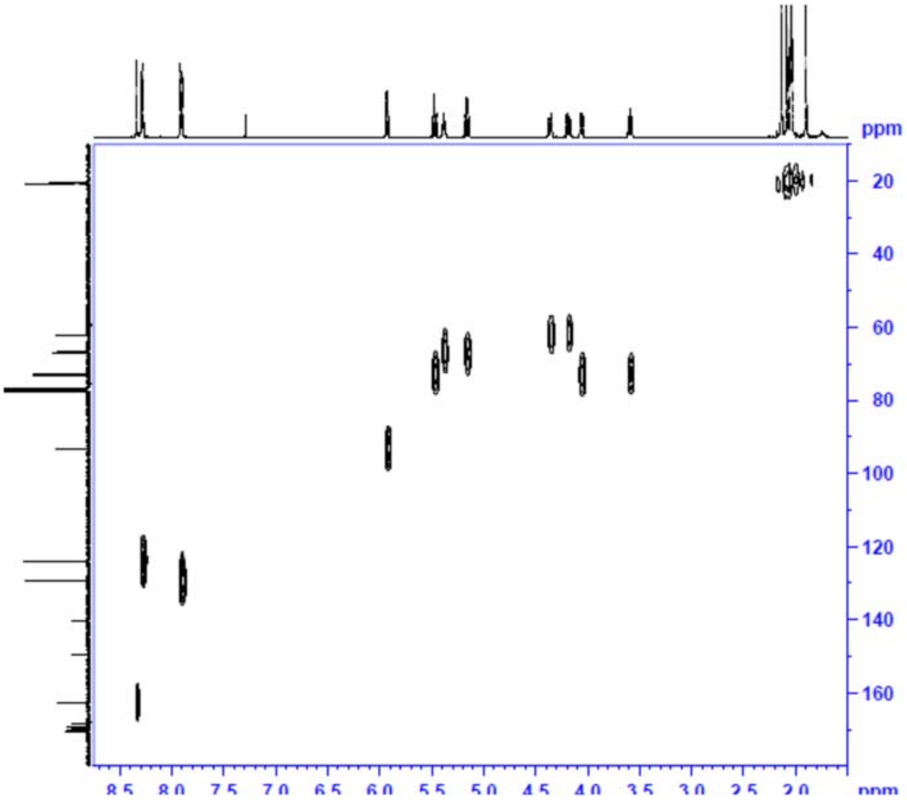

Figure S173. HMQC spectrum of **70** in $CDCl_3$

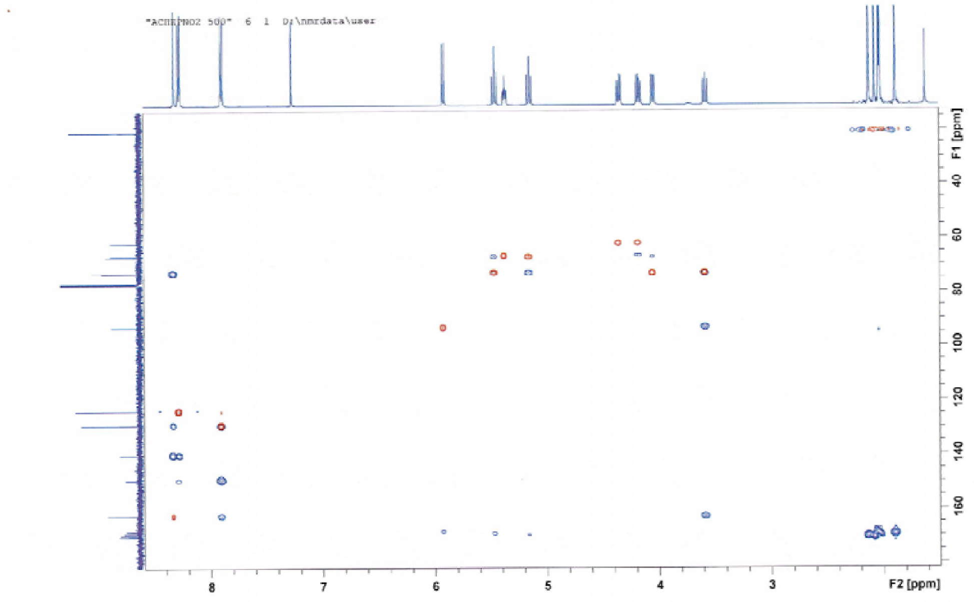

Figure S174. HMBC spectrum of **70** in $CDCl_3$

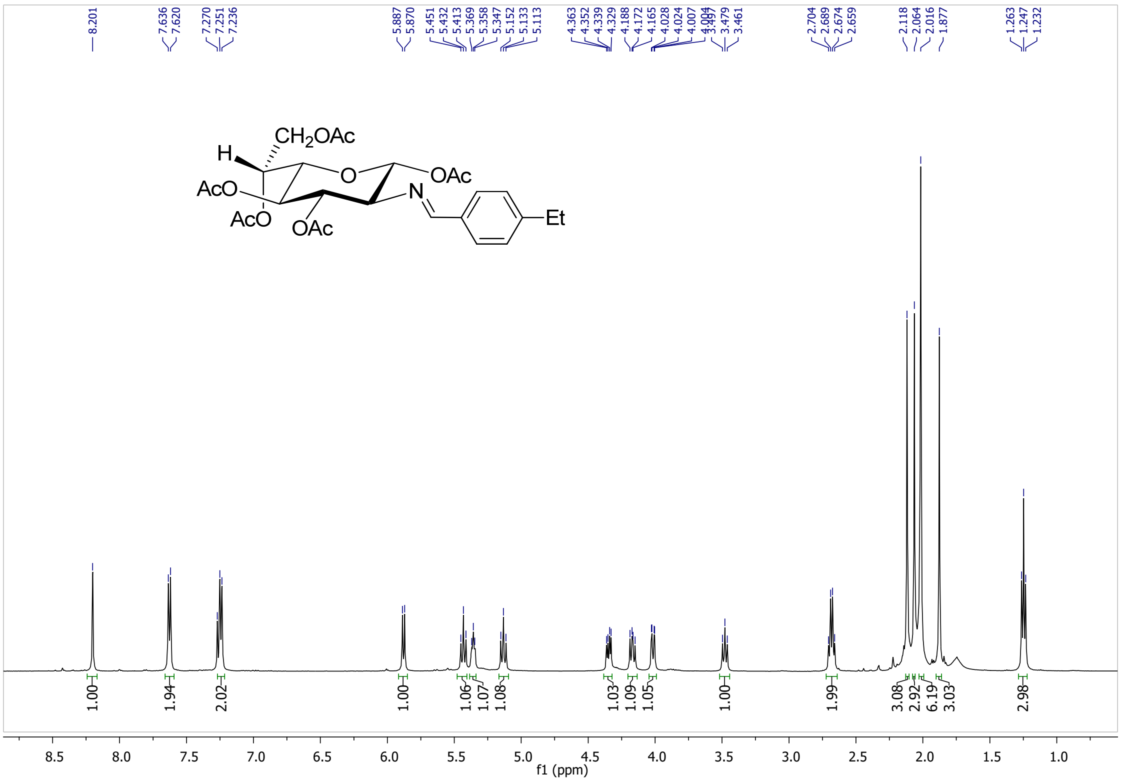

Figure S175. ^{1}H NMR spectrum of **71** in $CDCl_3$

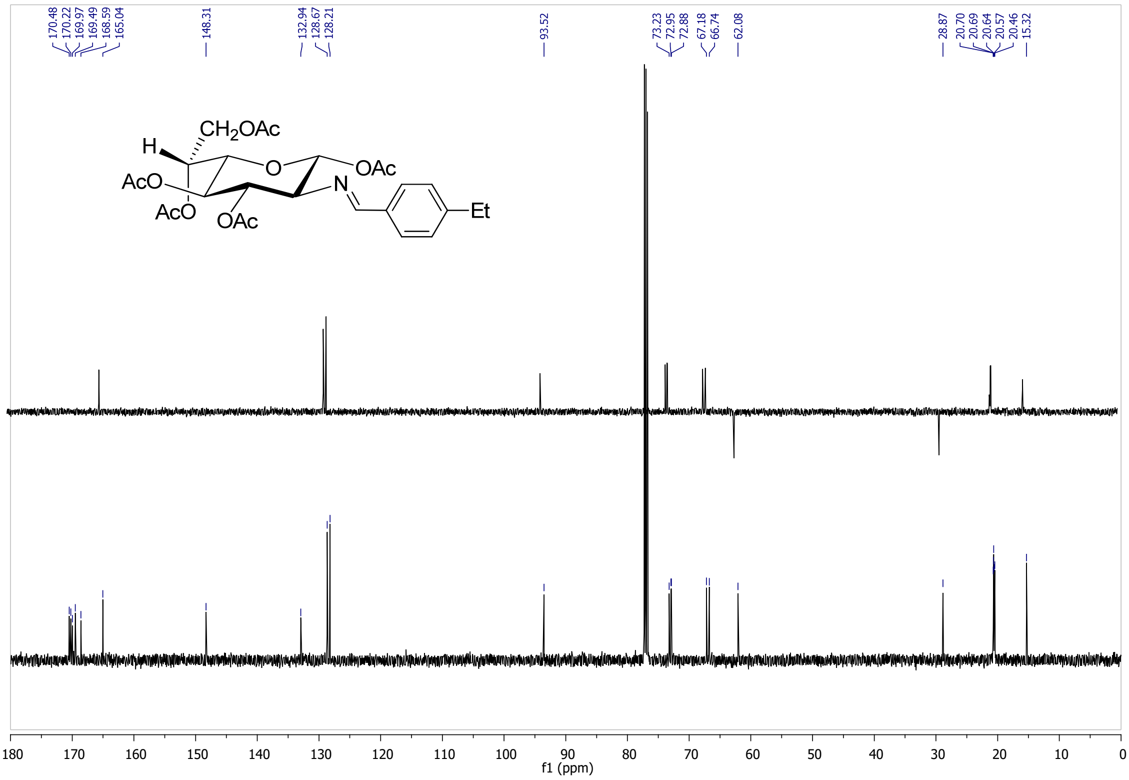

Figure S176. $^{13}C\{^{1}H\}$ NMR (top: DEPT) spectra of **71** in $CDCl_3$

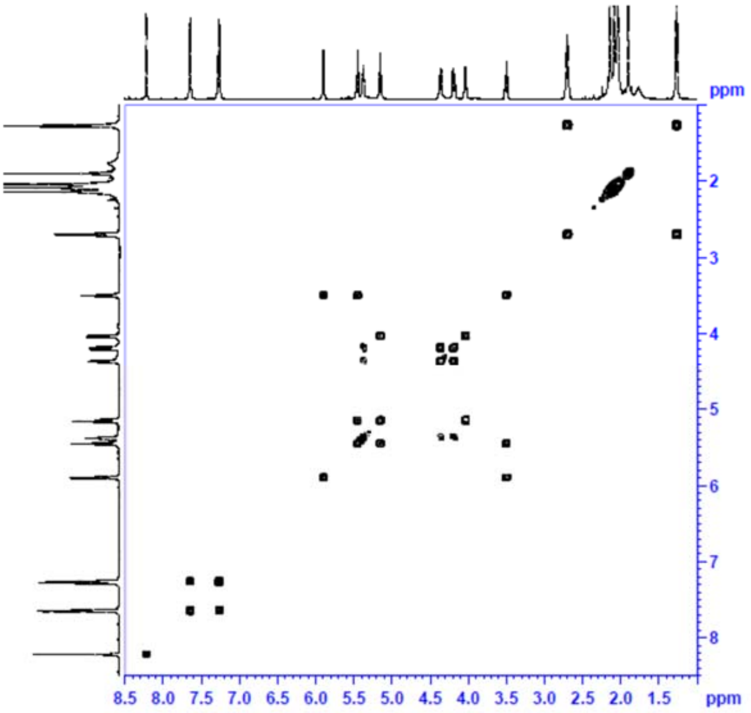

Figure S177. COSY spectrum of **71** in $CDCl_3$

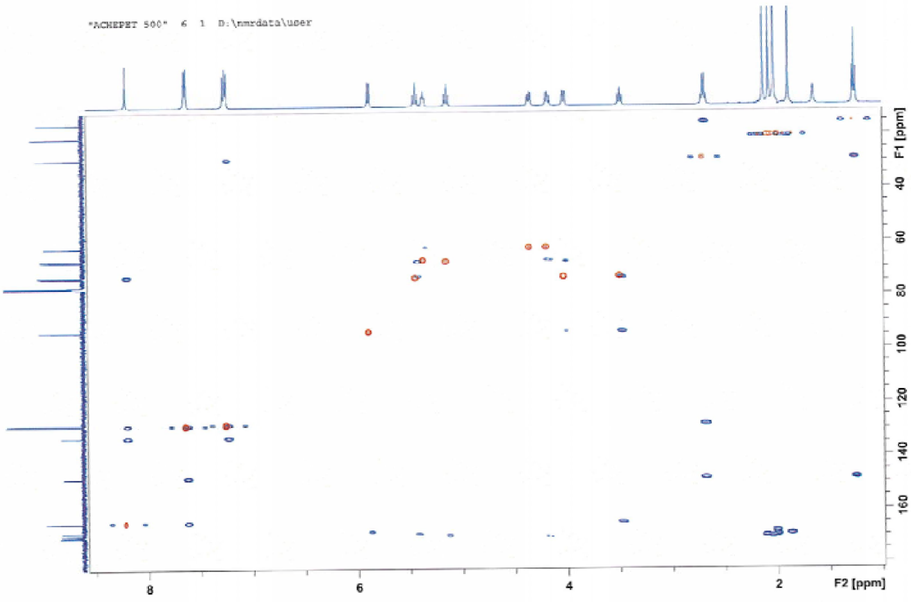

Figure S178. HMBC spectrum of **71** in $CDCl_3$

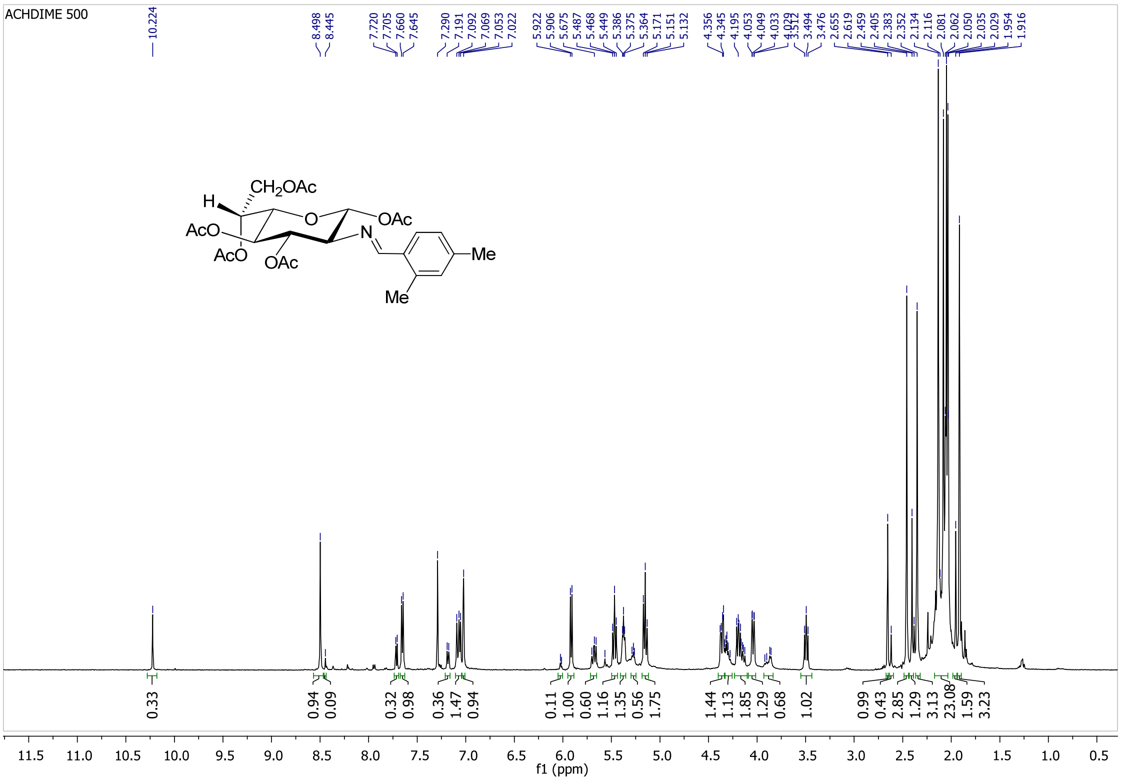

Figure S179. ^{1}H NMR spectrum of **72** in $CDCl_3$

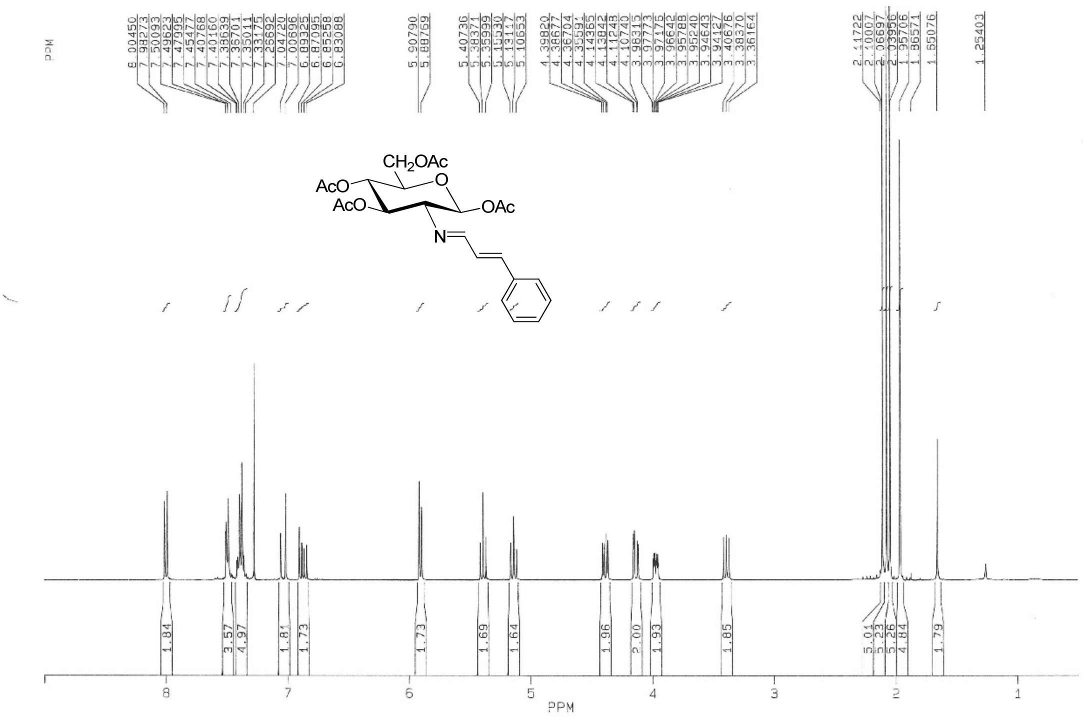

Figure S180. ^{1}H NMR spectrum of **73** in $CDCl_3$

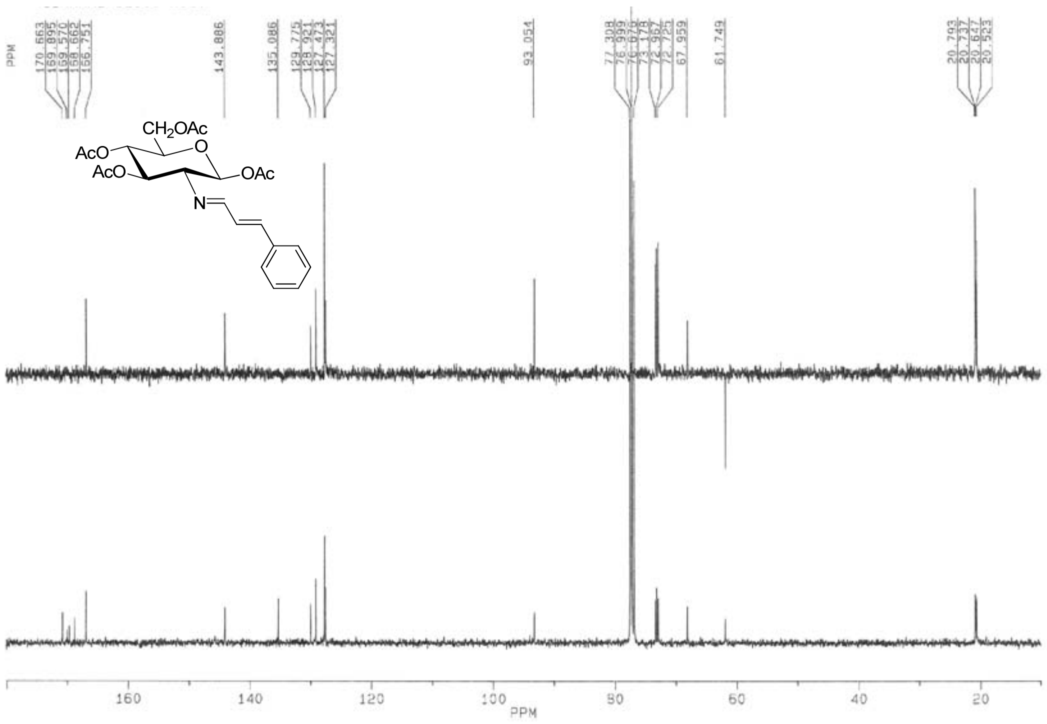

Figure S181. $^{13}C\{^1H\}$ NMR (top: DEPT) spectra of **73** in $CDCl_3$

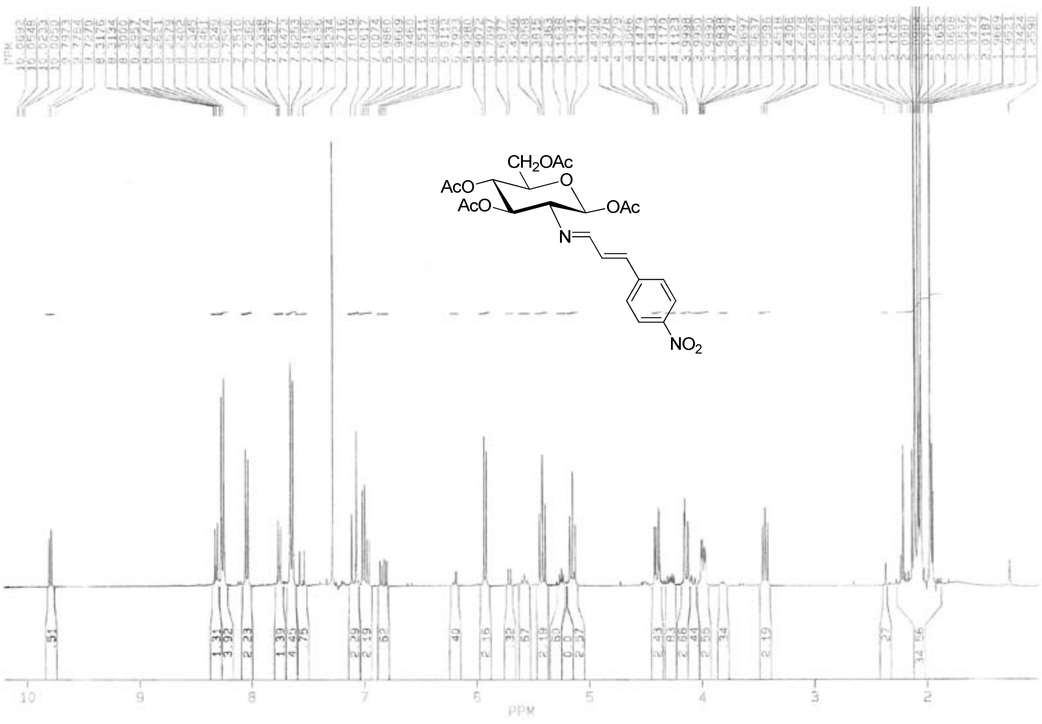

Figure S182. ^1H NMR spectrum of **74** in $CDCl_3$

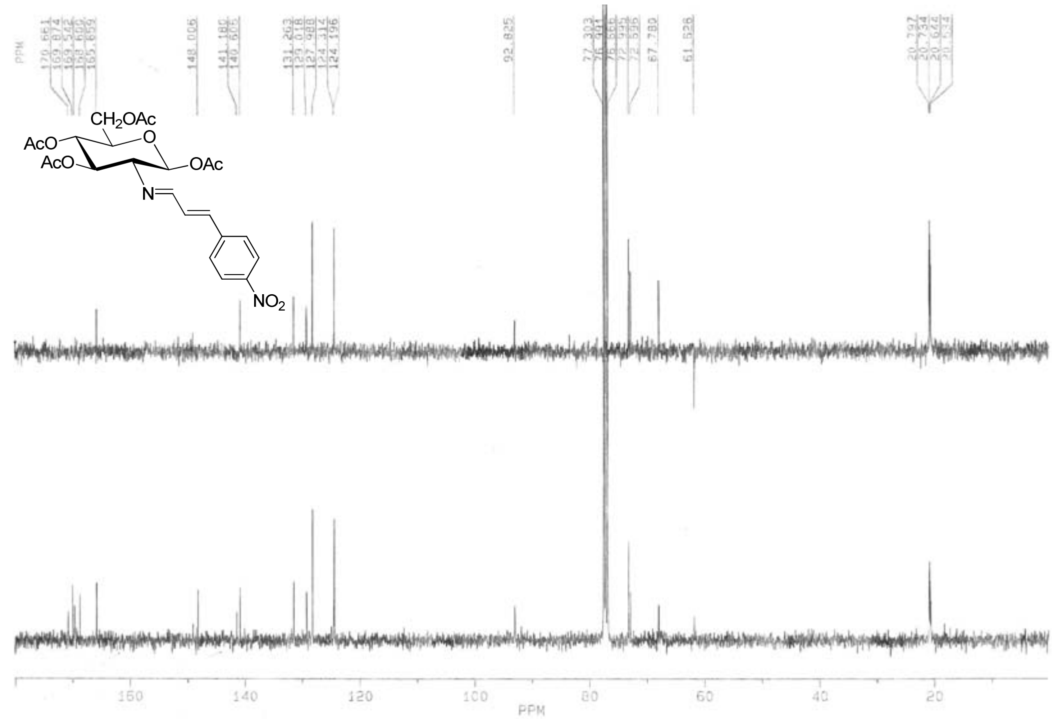

Figure S183. $^{13}C\{^1H\}$ NMR (top: DEPT) spectra of **74** in $CDCl_3$.

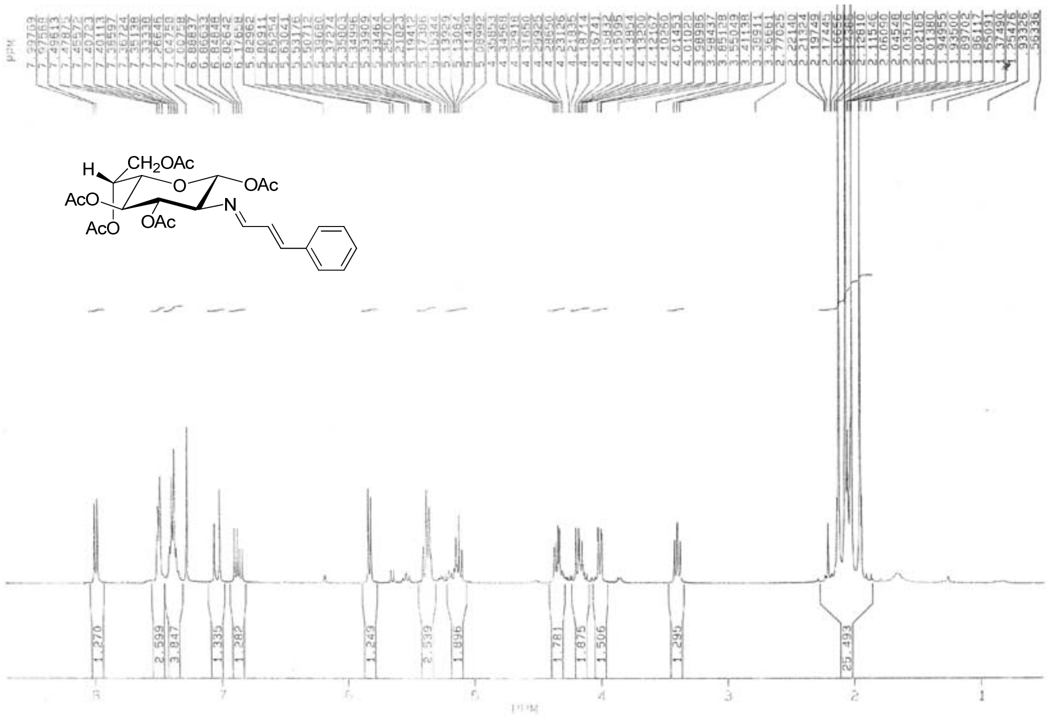

Figure S184. ^1H NMR spectrum of **75** in $CDCl_3$

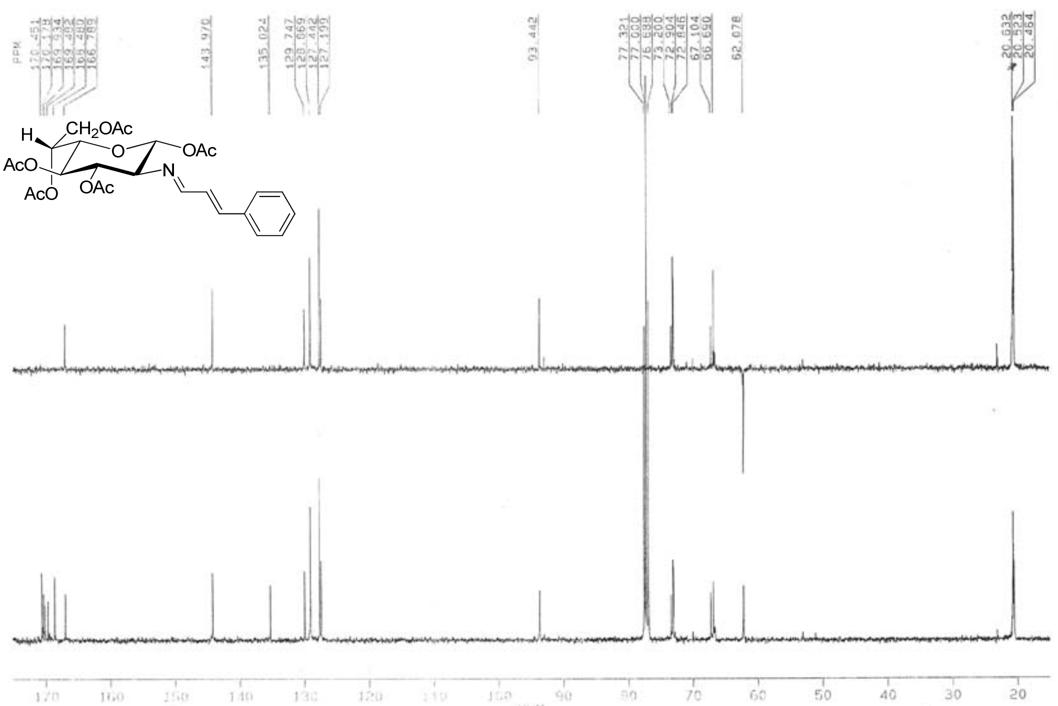

Figure S185. $^{13}C\{^{1}H\}$ NMR (top: DEPT) spectra of **75** in $CDCl_3$.

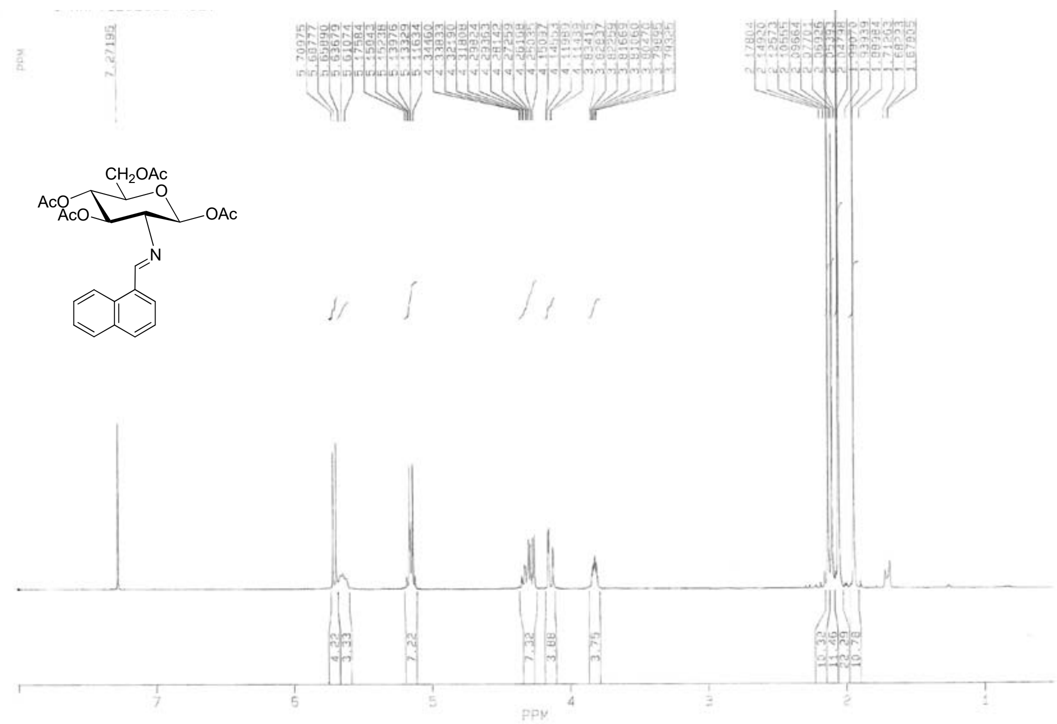

Figure S186. ^{1}H NMR spectrum of **76** in $CDCl_3$

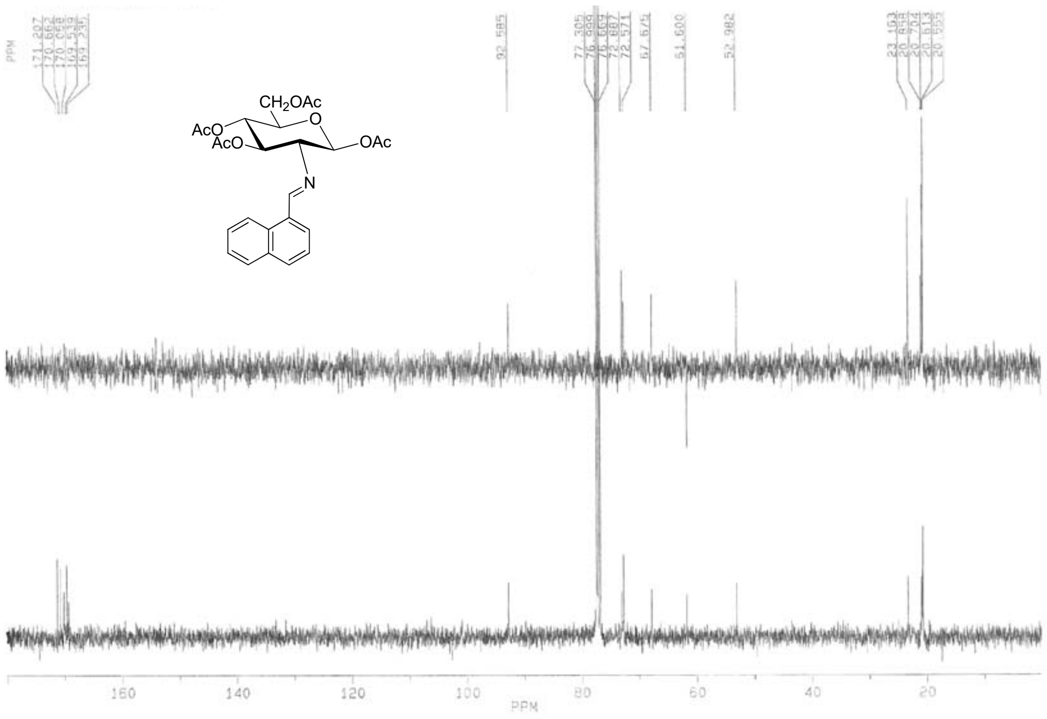

Figure S187. $^{13}C\{^1H\}$ NMR (top: DEPT) spectra of **76** in $CDCl_3$

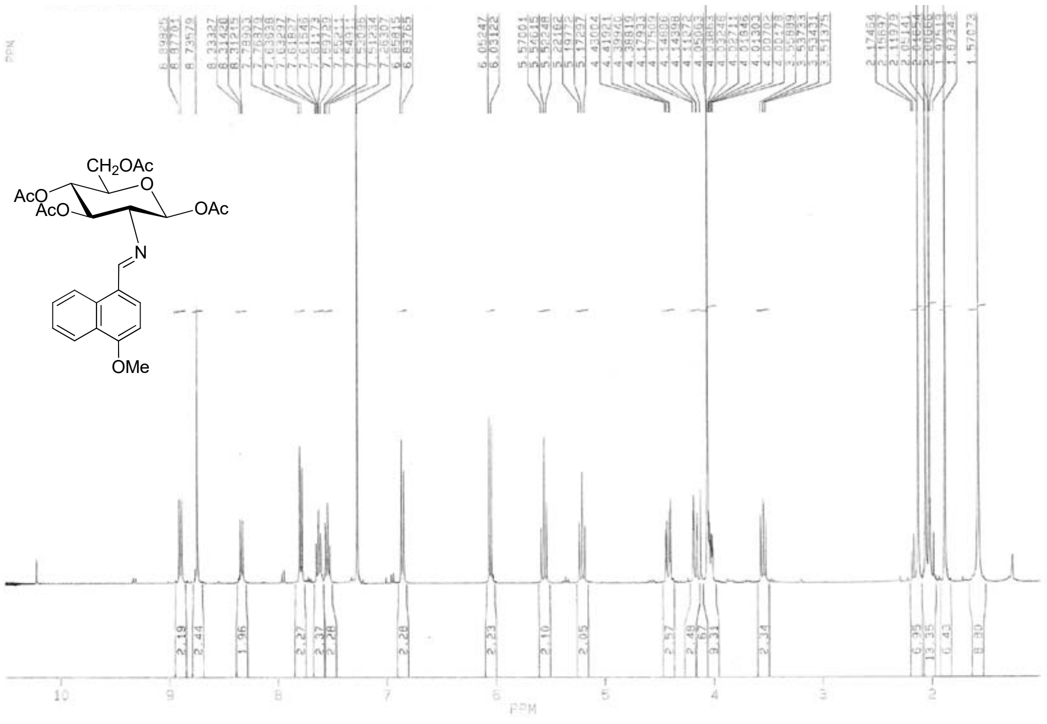

Figure S188. ^1H NMR spectrum of **77** in $CDCl_3$

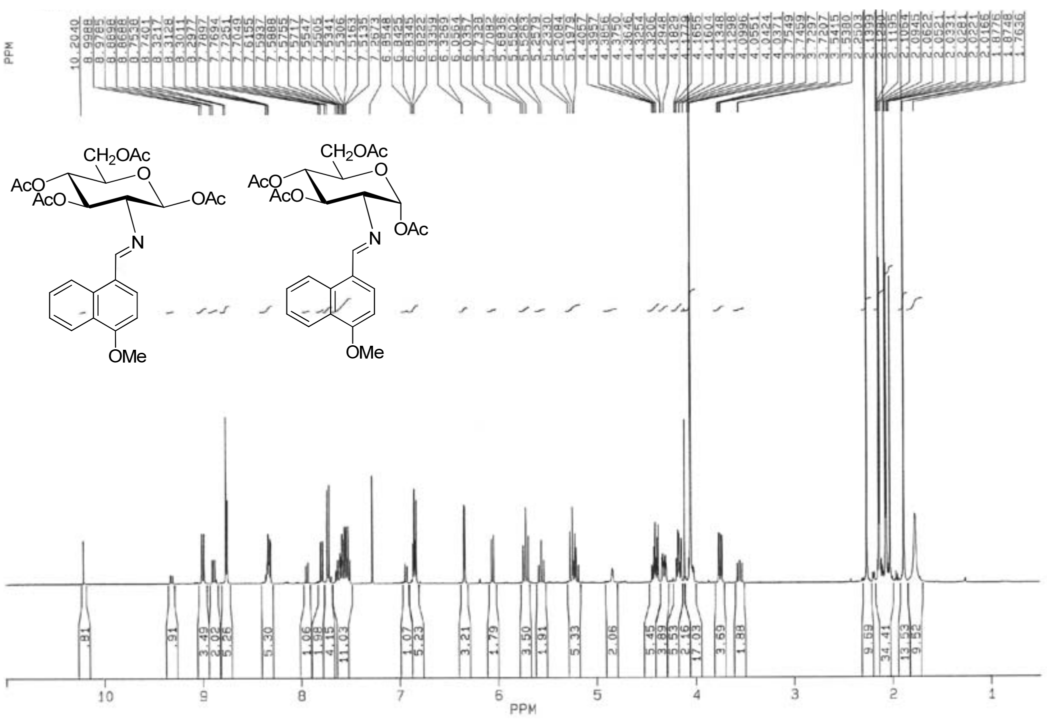

Figure S189. ^{1}H NMR spectrum of **77** and **78** in $CDCl_3$

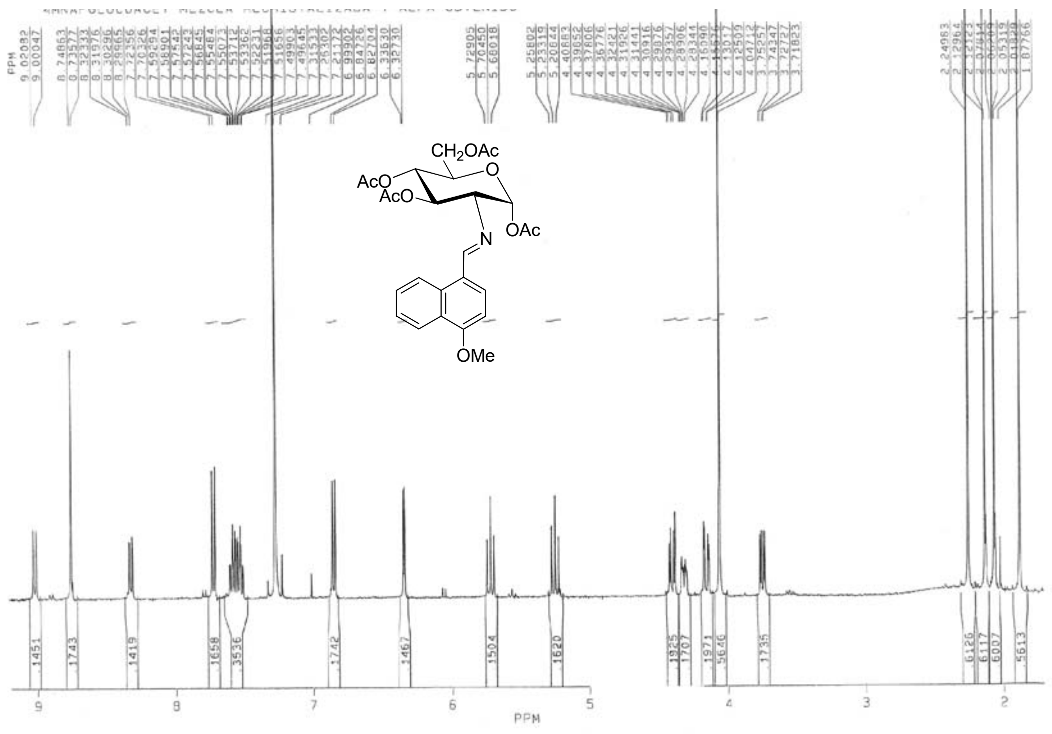

Figure S190. ^{1}H NMR spectrum of **78** in $CDCl_3$

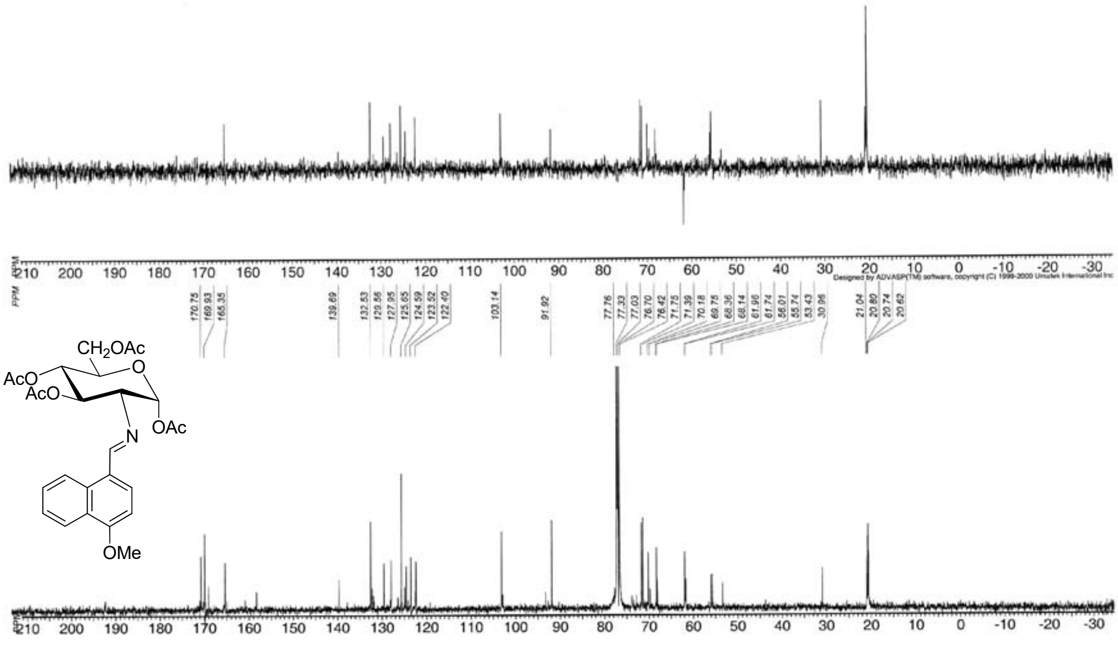

Figure S191. $^{13}C\{^1H\}$ NMR (top: DEPT) spectra of **78** in $CDCl_3$

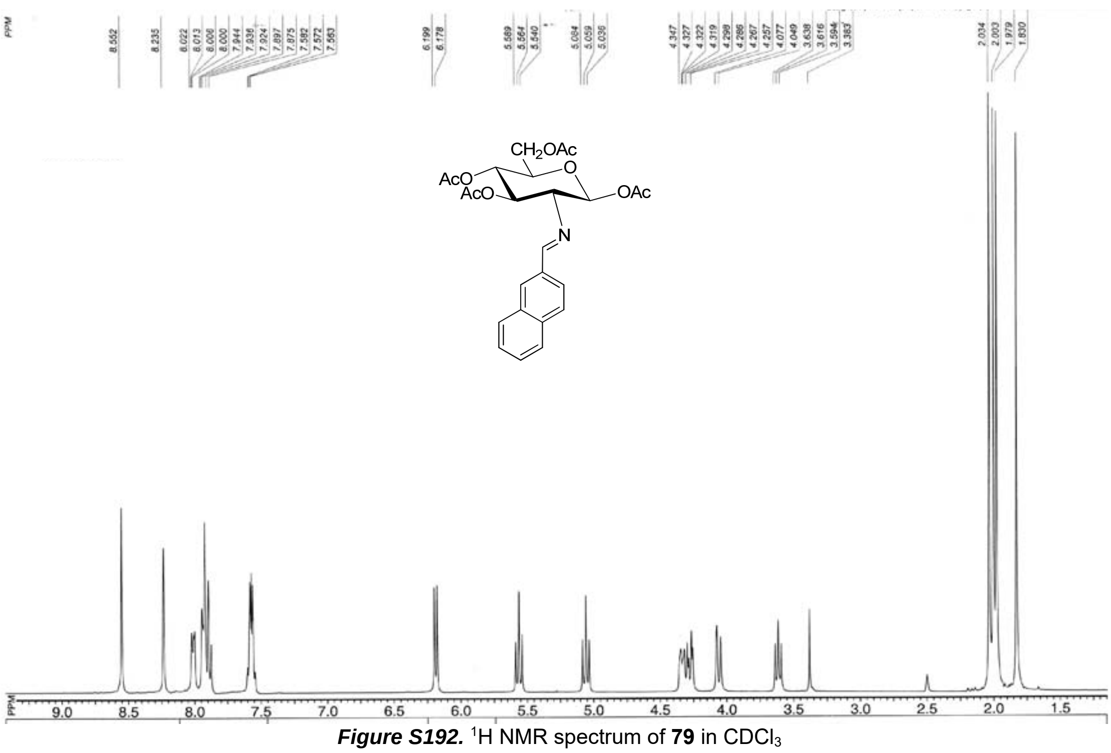

Figure S192. ^1H NMR spectrum of **79** in $CDCl_3$

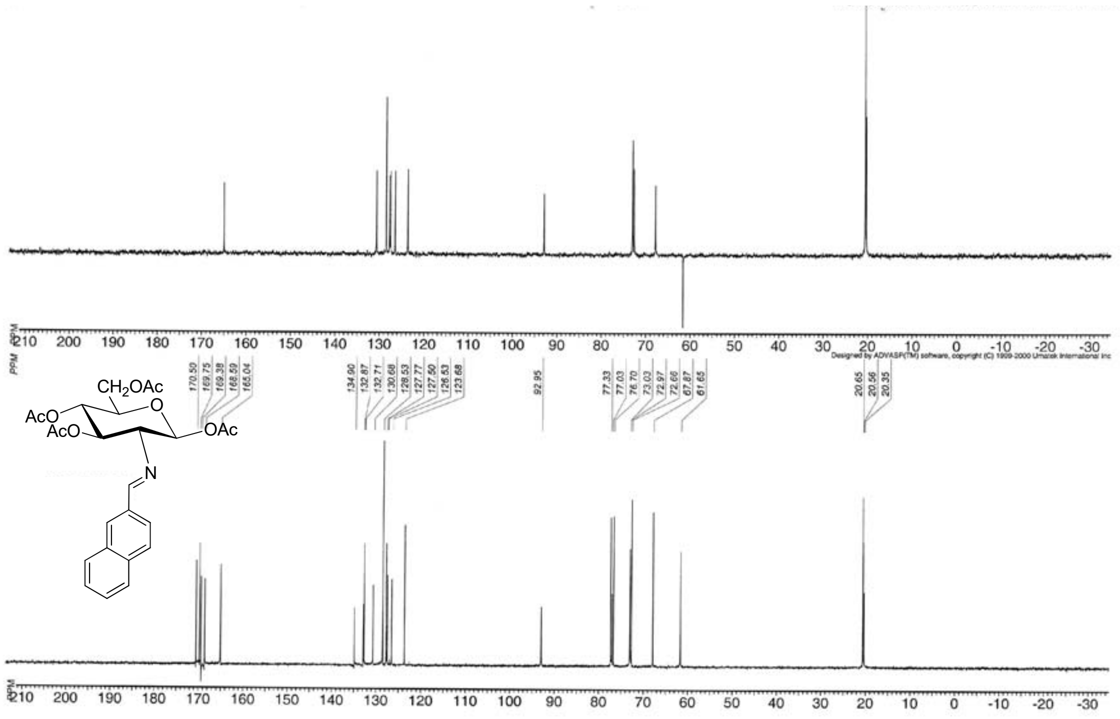

Figure S193. $^{13}C\{^1H\}$ NMR (top: DEPT) spectra of **79** in $CDCl_3$

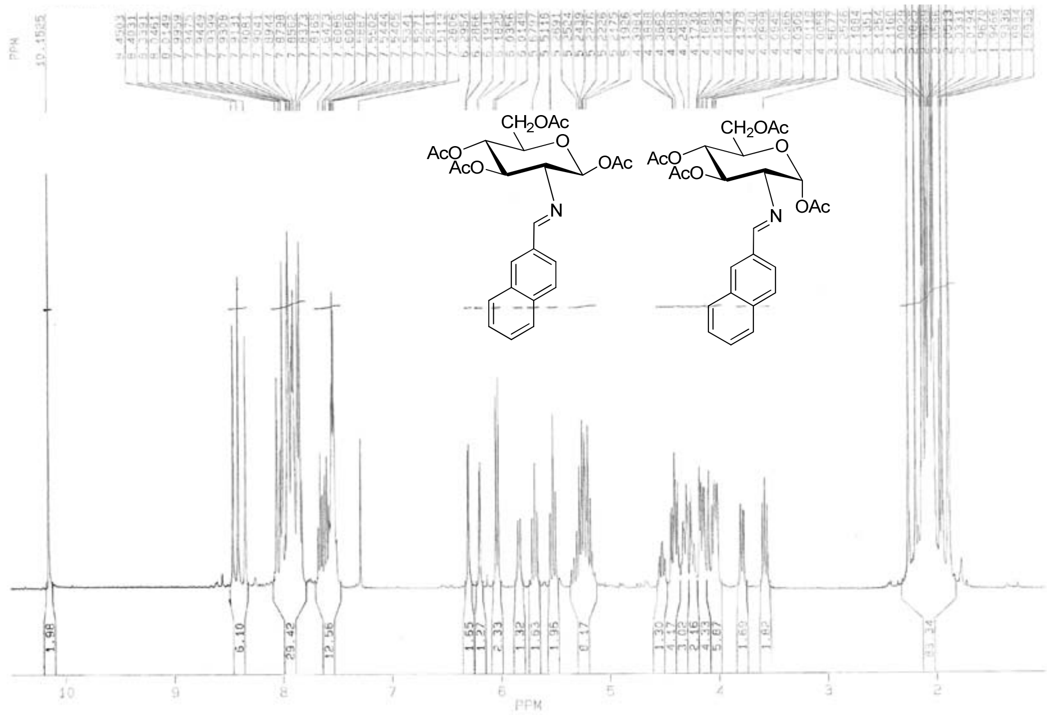

Figure S194. ^1H NMR spectrum of **79** and **80** in $CDCl_3$

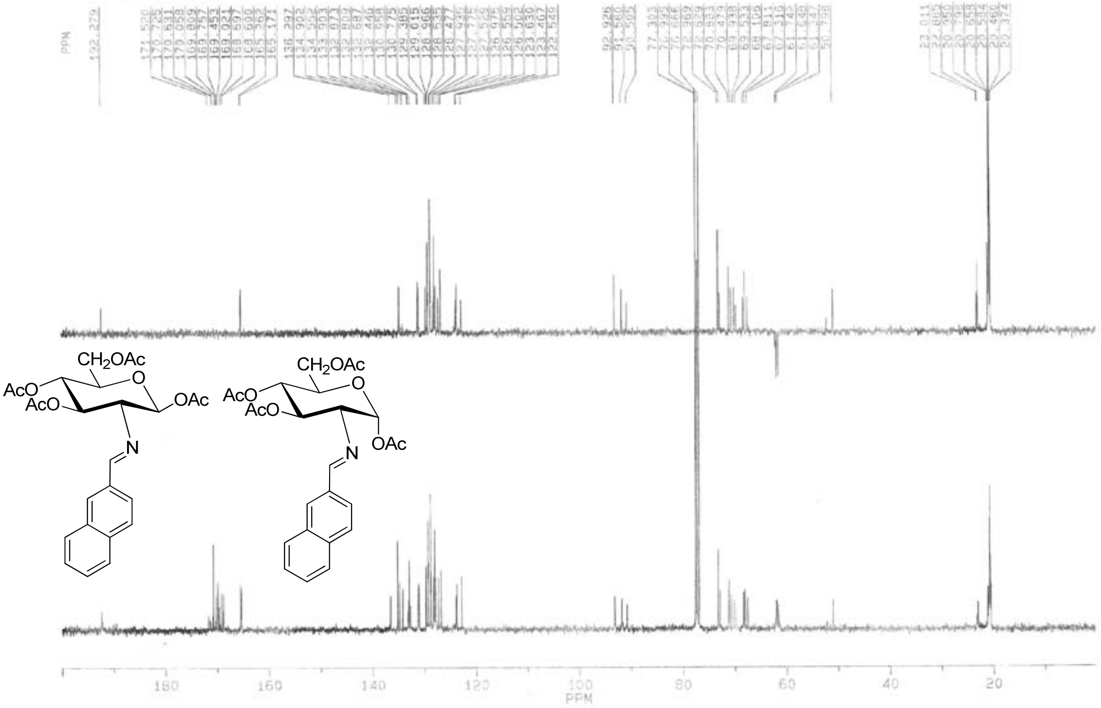

Figure S195. $^{13}C\{^1H\}$ NMR (top: DEPT) spectra of **79** and **80** in $CDCl_3$

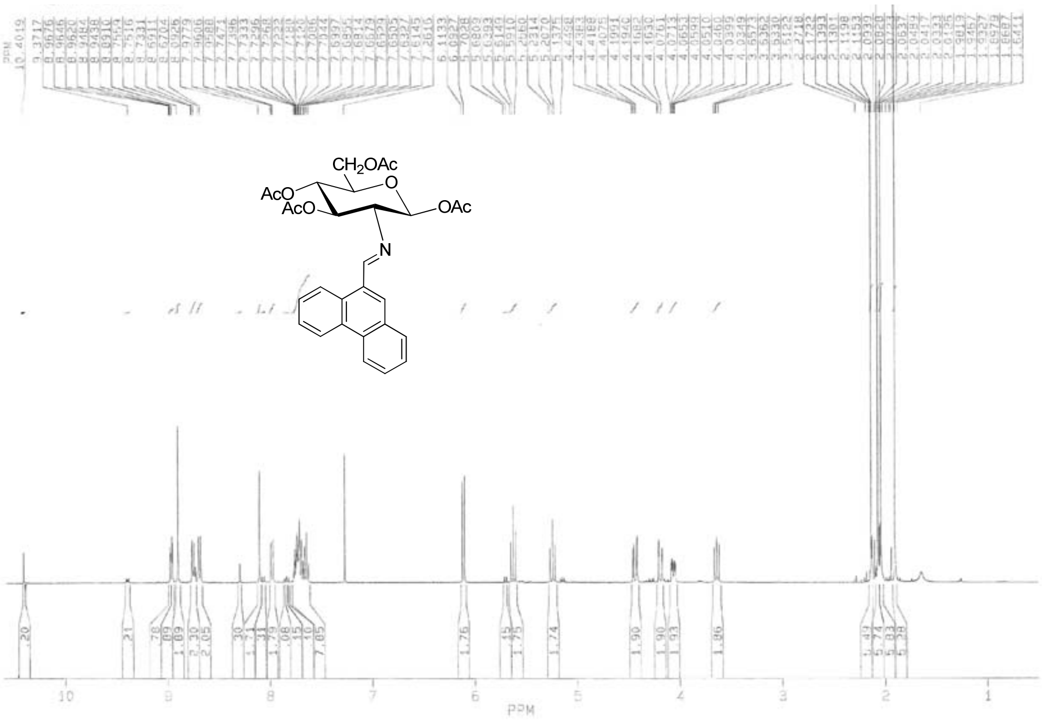

Figure S196. ^1H NMR spectrum of **81** in $CDCl_3$

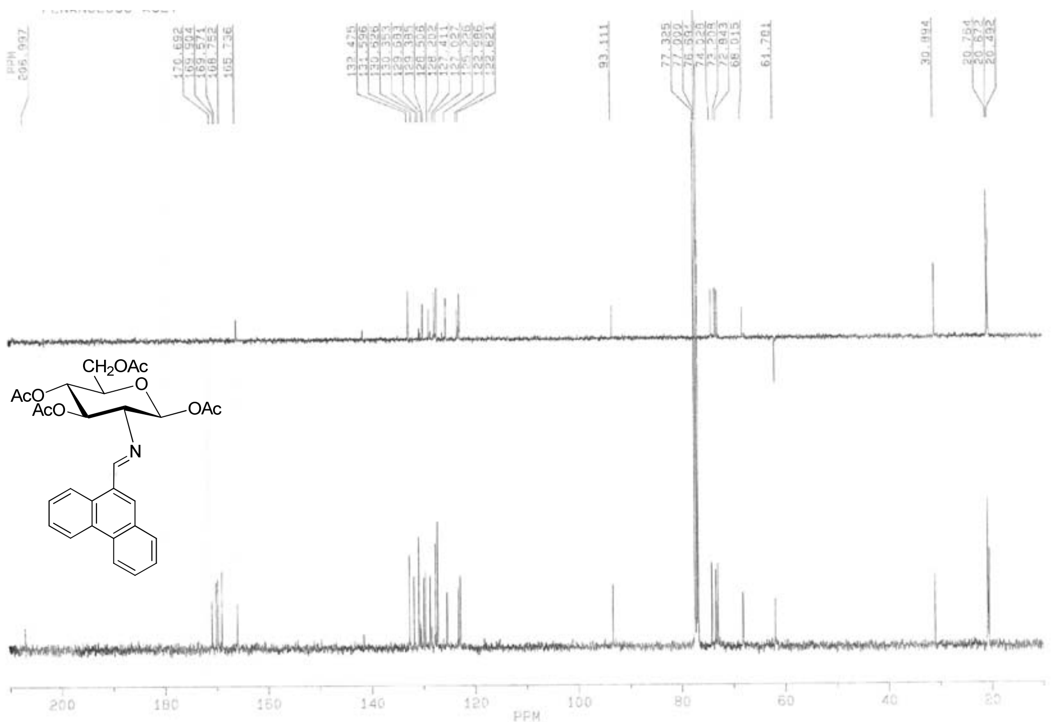

Figure S197. $^{13}C\{^1H\}$ NMR (top: DEPT) spectra of **81** in $CDCl_3$

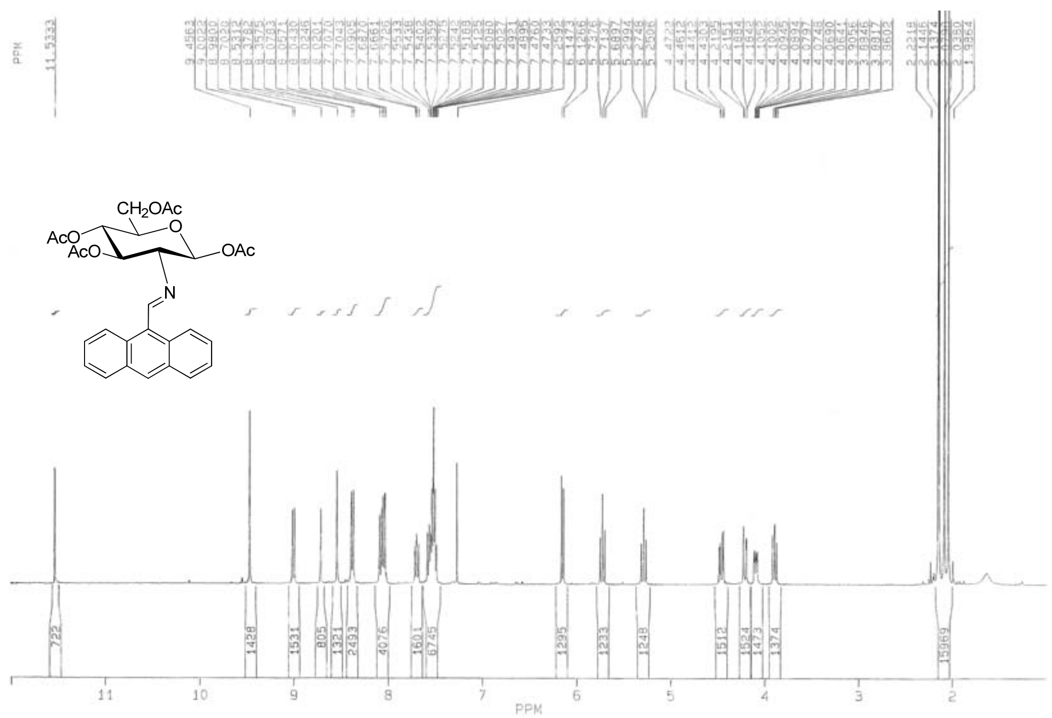

Figure S198. ^1H NMR spectrum of **82** in $CDCl_3$

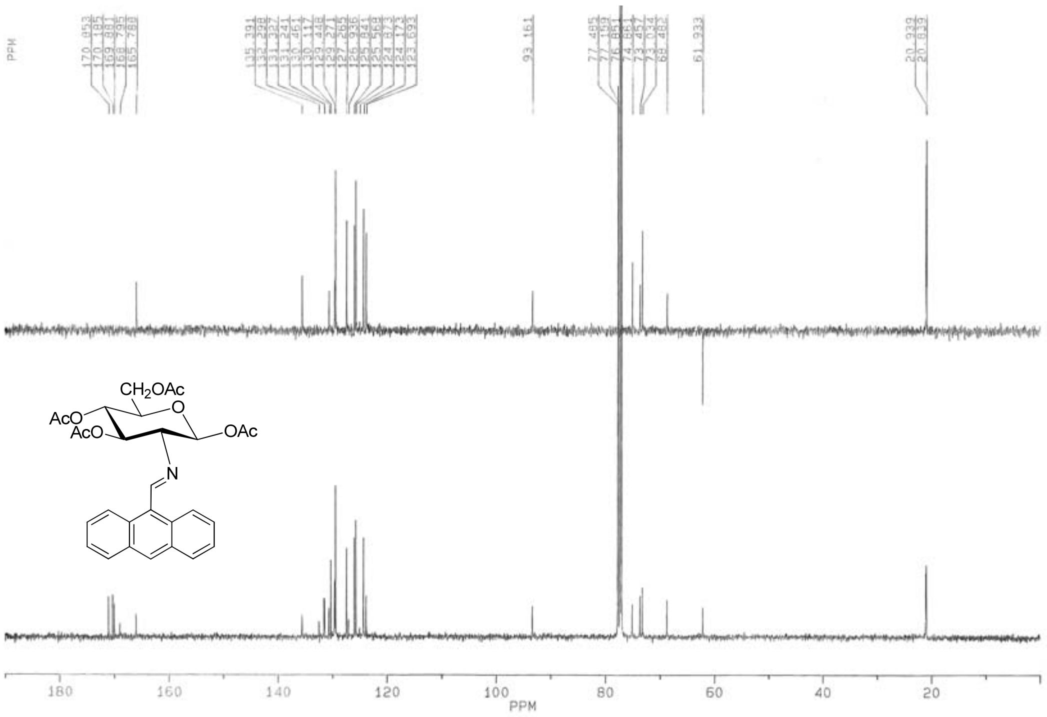

Figure S199. $^{13}C\{^1H\}$ NMR (top: DEPT) spectra of **82** in $CDCl_3$

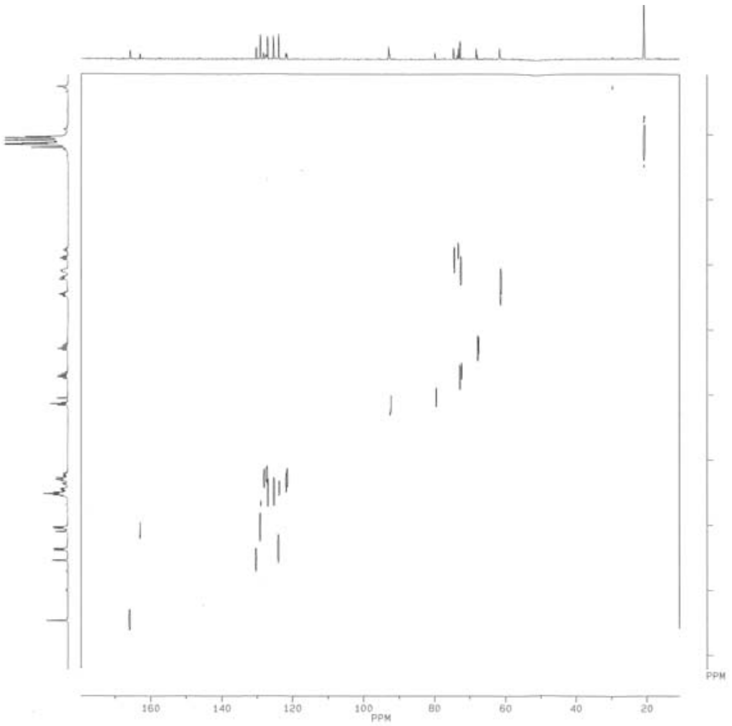

Figure S200. HMQC spectrum of **82** in $CDCl_3$

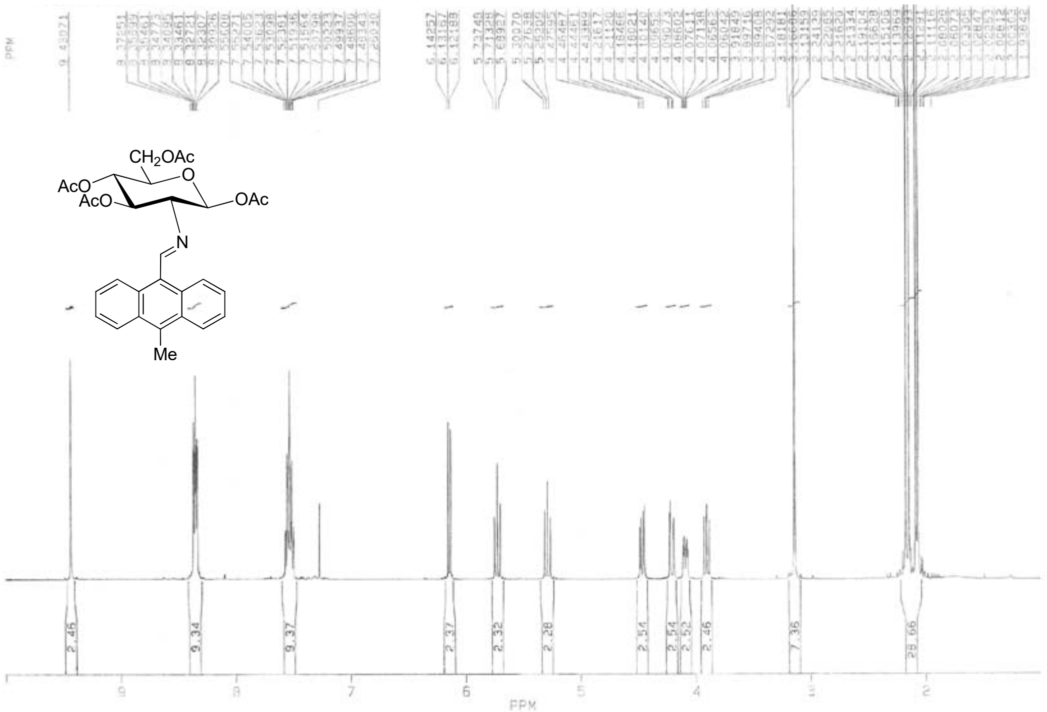

Figure S201. ^{1}H NMR spectrum of **83** in $CDCl_3$

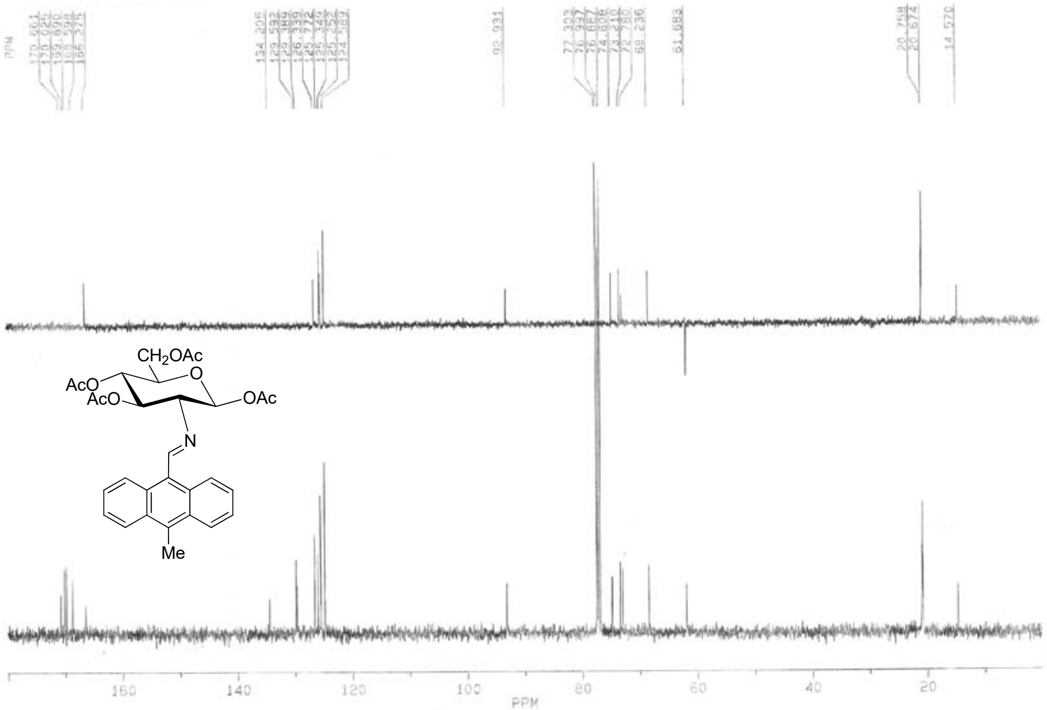

Figure S202. $^{13}C\{^{1}H\}$ NMR (top: DEPT) spectra of **83** in $CDCl_3$

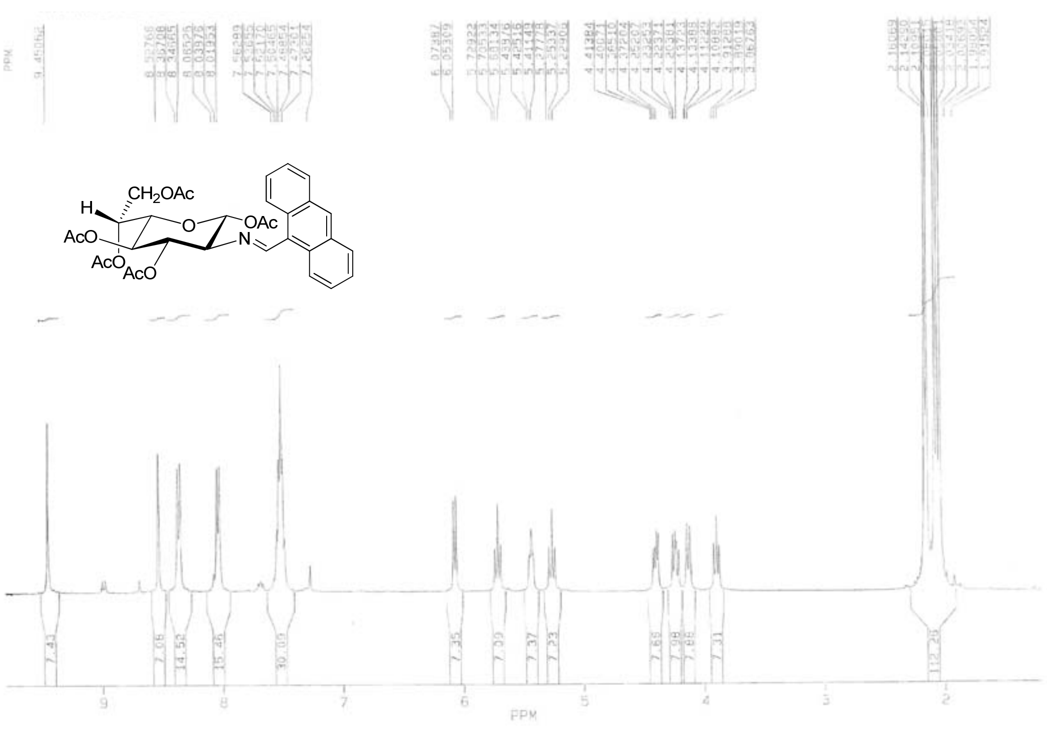

Figure S203. ^{1}H NMR spectrum of **84** in $CDCl_3$

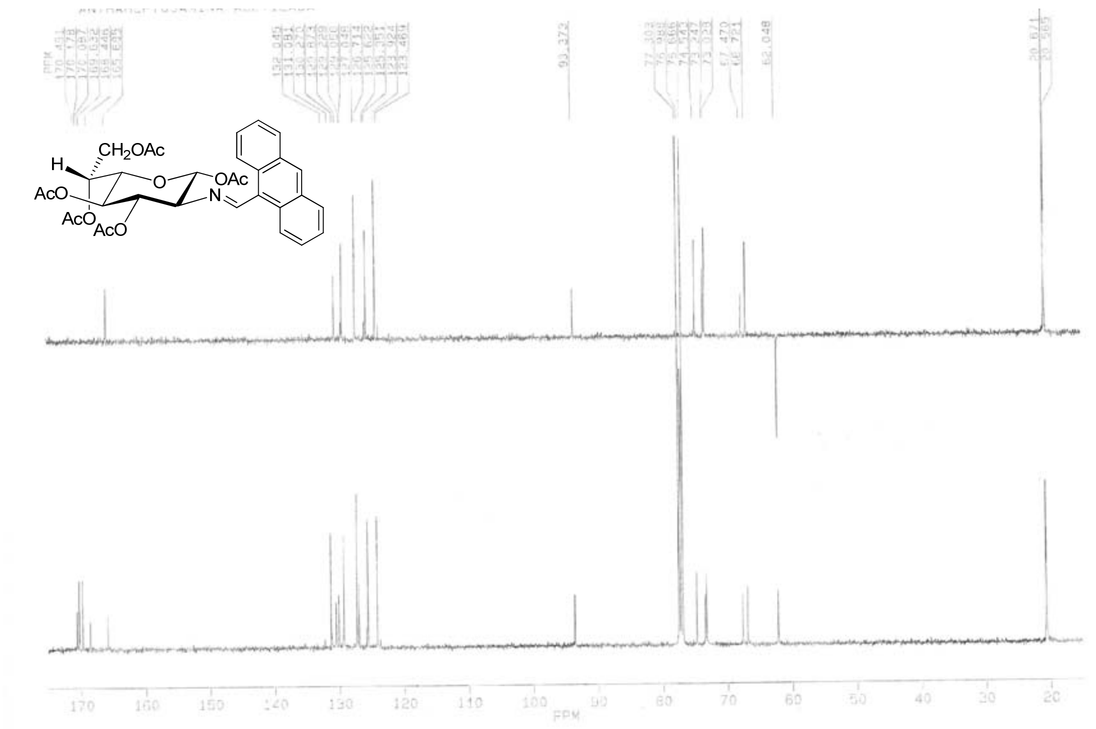

Figure S204. $^{13}C\{^{1}H\}$ NMR (top: DEPT) spectra of **84** in $CDCl_3$

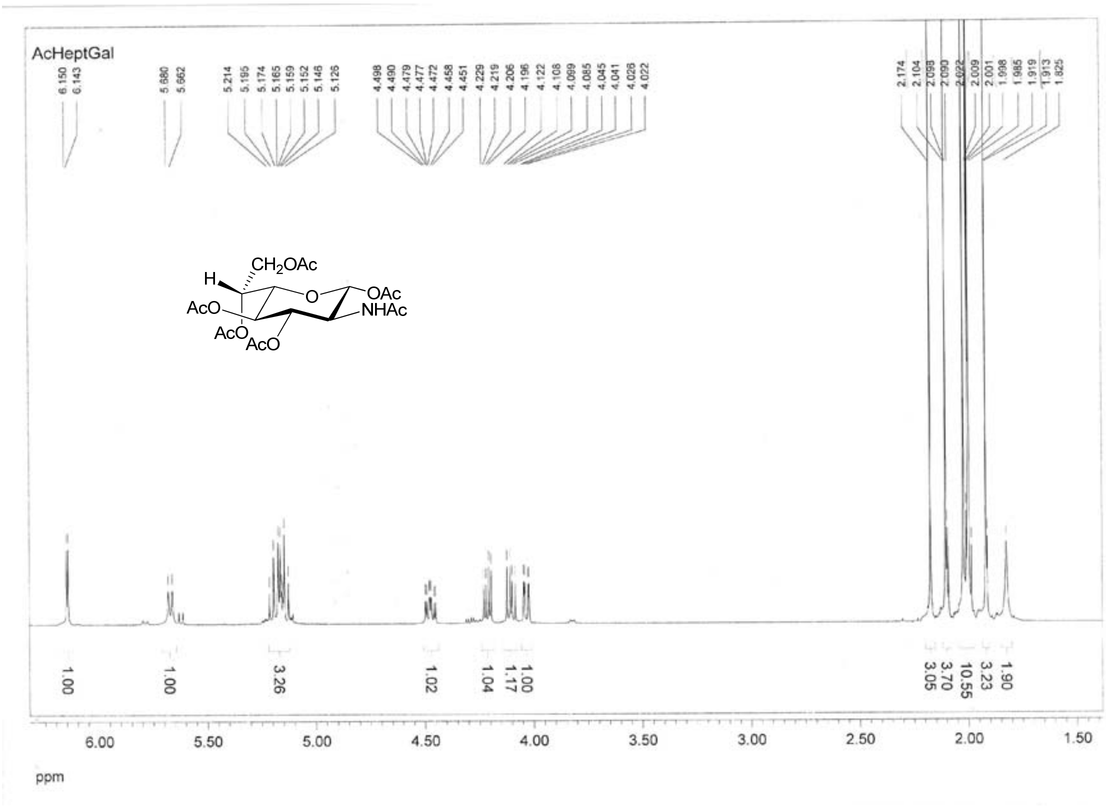

Figure S205. ^{1}H NMR spectrum of **90** in $CDCl_3$

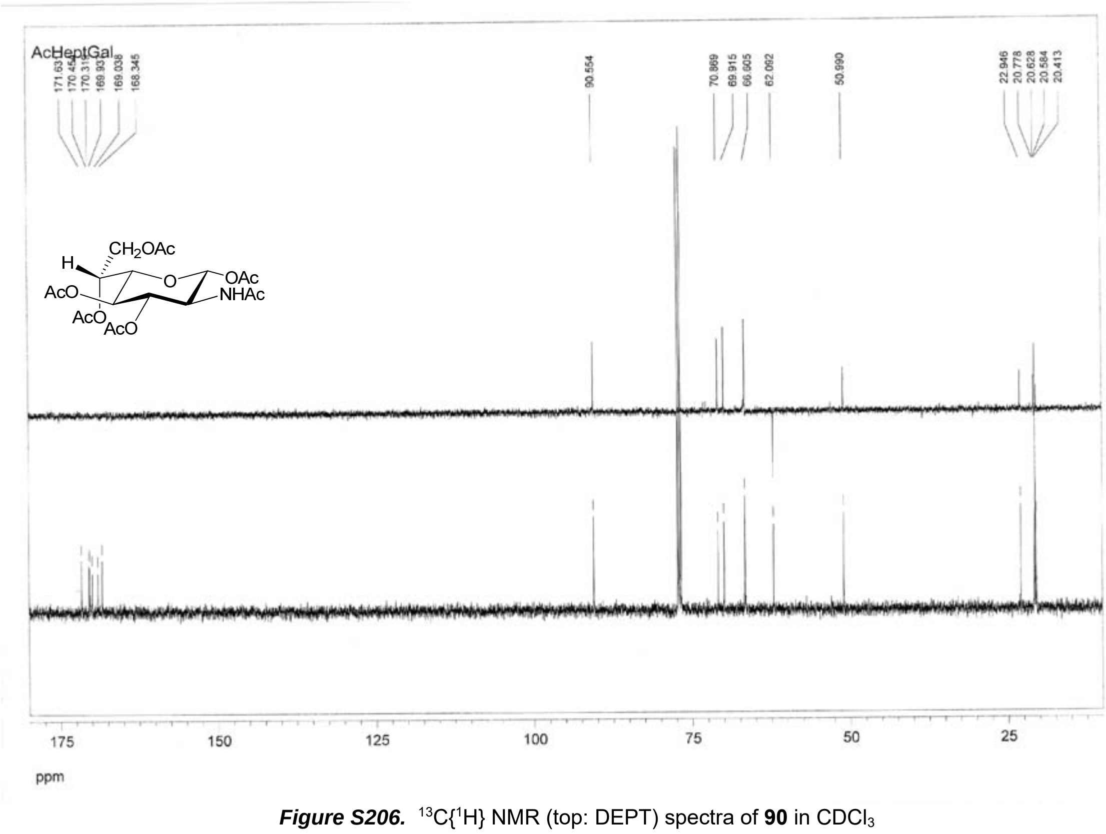

Figure S206. $^{13}C\{^1H\}$ NMR (top: DEPT) spectra of **90** in $CDCl_3$

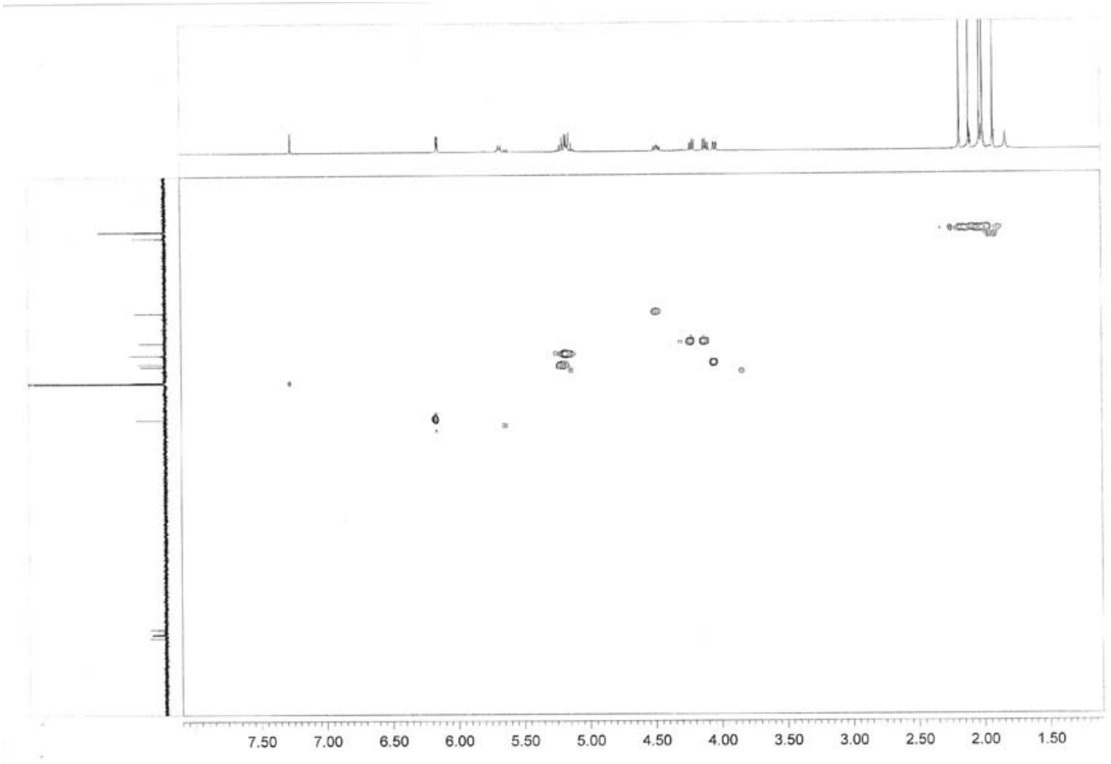

Figure S207. [1]HSQC spectrum of **90** in $CDCl_3$

Structure 30 (M06-2X/6-311G(d,p), Gas Phase)

Energy (Hartrees): = - 936.3487594
No imaginary frequencies

Standard orientation:

Center Number	Atomic Number	Atomic Type	Coordinates (Angstroms) X	Y	Z
1	6	0	1.199145	-1.515244	0.586850
2	6	0	0.436237	-0.176718	0.597702
3	6	0	1.143742	0.809017	-0.335712
4	6	0	2.603524	0.913273	0.058016
5	6	0	3.247672	-0.468522	0.008532
6	1	0	1.088461	0.441080	-1.366571
7	1	0	2.663068	1.291745	1.089492
8	1	0	3.198108	-0.857132	-1.014016
9	1	0	0.456928	0.239268	1.614305
10	8	0	2.543636	-1.325604	0.902158
11	6	0	4.702041	-0.451963	0.458164
12	1	0	4.771469	0.062033	1.427780
13	1	0	5.025515	-1.484013	0.600052
14	8	0	5.543532	0.129051	-0.509401
15	1	0	5.156104	0.977194	-0.750722
16	8	0	3.296453	1.776436	-0.823756
17	1	0	2.783479	2.589900	-0.878115
18	8	0	0.593586	2.112486	-0.239857
19	1	0	-0.236941	2.130249	-0.722686
20	7	0	-0.912295	-0.395869	0.118458
21	6	0	-3.267283	0.097595	0.283775
22	6	0	-3.639178	-0.703406	-0.799675
23	6	0	-4.243683	0.805836	0.983990
24	6	0	-4.970789	-0.791942	-1.171471
25	1	0	-2.870066	-1.247670	-1.333740
26	6	0	-5.579250	0.717685	0.609060
27	1	0	-3.954550	1.425931	1.826394
28	6	0	-5.942871	-0.081396	-0.468074
29	1	0	-5.257190	-1.414123	-2.010799
30	1	0	-6.333856	1.269070	1.156471
31	1	0	-6.983269	-0.152915	-0.761914
32	6	0	-1.859806	0.209151	0.705169
33	1	0	-1.669039	0.857768	1.571244
34	1	0	0.810605	-2.176094	1.368175
35	8	0	1.094318	-2.116407	-0.675442
36	1	0	0.188033	-1.969102	-0.972128

Structure 30 (M06-2X/6-311G(d,p), DMSO)

Energy (Hartrees): = - 936.3746193
No imaginary frequencies

Standard orientation:

Center Number	Atomic Number	Atomic Type	Coordinates (Angstroms) X	Y	Z
1	6	0	1.174856	-1.497436	0.601255
2	6	0	0.436880	-0.147494	0.602498
3	6	0	1.156257	0.831607	-0.329996
4	6	0	2.619003	0.906415	0.057694
5	6	0	3.239478	-0.485117	0.004517
6	1	0	1.085173	0.476490	-1.364787
7	1	0	2.694848	1.287216	1.086007
8	1	0	3.180061	-0.868632	-1.020193
9	1	0	0.452646	0.266348	1.618644
10	8	0	2.532444	-1.340526	0.897053
11	6	0	4.694730	-0.489053	0.449383
12	1	0	4.775340	0.015096	1.422300
13	1	0	5.017049	-1.524379	0.576508
14	8	0	5.541317	0.103346	-0.515963
15	1	0	5.139433	0.948989	-0.747919
16	8	0	3.331706	1.751274	-0.832987
17	1	0	2.849405	2.586067	-0.875251
18	8	0	0.632682	2.143925	-0.220443
19	1	0	-0.197129	2.183148	-0.708952
20	7	0	-0.910190	-0.363790	0.114123
21	6	0	-3.267147	0.107586	0.275521

22	6	0	-3.632192	-0.691551	-0.813344
23	6	0	-4.250827	0.800774	0.983003
24	6	0	-4.964884	-0.794249	-1.182931
25	1	0	-2.863456	-1.226279	-1.359119
26	6	0	-5.587091	0.698055	0.609819
27	1	0	-3.964189	1.419342	1.827597
28	6	0	-5.944559	-0.099806	-0.471954
29	1	0	-5.245449	-1.414328	-2.026530
30	1	0	-6.347381	1.237740	1.162262
31	1	0	-6.985211	-0.182119	-0.764098
32	6	0	-1.861651	0.234422	0.703603
33	1	0	-1.682880	0.877155	1.574039
34	1	0	0.780391	-2.135916	1.397556
35	8	0	1.042757	-2.125873	-0.648006
36	1	0	0.158305	-1.901502	-0.968565

Structure 30 (M06-2X/def2-TZVP, Gas Phase)

Energy (Hartrees): = -936.464964
No imaginary frequencies

Standard orientation:

Center Number	Atomic Number	Atomic Type	Coordinates (Angstroms) X	Y	Z
1	6	0	1.176840	-1.499022	0.571540
2	6	0	0.433815	-0.151316	0.575400
3	6	0	1.161132	0.828998	-0.344065
4	6	0	2.618954	0.910681	0.057250
5	6	0	3.243374	-0.478167	0.004262
6	1	0	1.104582	0.471795	-1.377874
7	1	0	2.679040	1.280377	1.090582
8	1	0	3.199072	-0.858484	-1.021362
9	1	0	0.452668	0.257502	1.594015
10	8	0	2.522358	-1.330322	0.883981
11	6	0	4.690497	-0.487833	0.473033
12	1	0	4.755229	0.013827	1.447759
13	1	0	4.997137	-1.525009	0.609289
14	8	0	5.561225	0.088440	-0.469613
15	1	0	5.199246	0.946060	-0.721959
16	8	0	3.329304	1.770723	-0.811561
17	1	0	2.830646	2.593711	-0.875029
18	8	0	0.625498	2.136608	-0.242854
19	1	0	-0.220902	2.164018	-0.699704
20	7	0	-0.911704	-0.349574	0.089207
21	6	0	-3.272276	0.093837	0.284515
22	6	0	-3.639369	-0.656081	-0.833356
23	6	0	-4.252436	0.751644	1.021969
24	6	0	-4.969157	-0.743588	-1.201816
25	1	0	-2.868745	-1.163811	-1.398233
26	6	0	-5.586589	0.664567	0.650883
27	1	0	-3.966532	1.333509	1.890869
28	6	0	-5.944889	-0.083255	-0.460659
29	1	0	-5.251819	-1.327625	-2.068037
30	1	0	-6.344289	1.178108	1.227857
31	1	0	-6.984855	-0.154136	-0.752332
32	6	0	-1.867159	0.204155	0.706587
33	1	0	-1.685000	0.804758	1.607149
34	1	0	0.778327	-2.145504	1.359288
35	8	0	1.059339	-2.118083	-0.680310
36	1	0	0.169920	-1.928730	-1.007123

Structure 30 (M06-2X/def2-TZVP, DMSO)

Energy (Hartrees): = -936.490747
No imaginary frequencies

Standard orientation:

Center Number	Atomic Number	Atomic Type	Coordinates (Angstroms) X	Y	Z
1	6	0	1.154699	-1.482129	0.588092
2	6	0	0.435357	-0.124362	0.580667
3	6	0	1.173011	0.849145	-0.338869
4	6	0	2.632886	0.905147	0.056912

5	6	0	3.235446	-0.492424	-0.001845
6	1	0	1.102864	0.502330	-1.375902
7	1	0	2.708199	1.276063	1.087642
8	1	0	3.180433	-0.867321	-1.029693
9	1	0	0.449416	0.283314	1.598426
10	8	0	2.513035	-1.344200	0.877315
11	6	0	4.683493	-0.522134	0.460804
12	1	0	4.759918	-0.031905	1.439475
13	1	0	4.990344	-1.562249	0.579520
14	8	0	5.557580	0.068280	-0.479628
15	1	0	5.181041	0.925405	-0.717572
16	8	0	3.362287	1.748689	-0.819653
17	1	0	2.897642	2.594602	-0.863908
18	8	0	0.659283	2.163874	-0.227590
19	1	0	-0.194688	2.203052	-0.675816
20	7	0	-0.908710	-0.321008	0.085685
21	6	0	-3.270966	0.100623	0.278113
22	6	0	-3.634276	-0.658051	-0.836440
23	6	0	-4.255048	0.757693	1.012988
24	6	0	-4.964853	-0.755453	-1.204459
25	1	0	-2.866396	-1.168032	-1.404642
26	6	0	-5.589502	0.660913	0.641580
27	1	0	-3.969526	1.345433	1.878292
28	6	0	-5.944765	-0.095941	-0.466315
29	1	0	-5.244245	-1.345872	-2.068169
30	1	0	-6.350326	1.174255	1.215886
31	1	0	-6.984898	-0.174156	-0.757795
32	6	0	-1.868114	0.224210	0.707120
33	1	0	-1.696683	0.817349	1.612371
34	1	0	0.753113	-2.104000	1.393239
35	8	0	1.005508	-2.130728	-0.648593
36	1	0	0.139016	-1.871955	-0.996035

Structure 30.5H_2O (M06-2X/def2-TZVP, Gas Phase)

Energy (Hartrees): = -1318.686836
No imaginary frequencies

Standard orientation:

Center Number	Atomic Number	Atomic Type	Coordinates (Angstroms) X	Y	Z
1	6	0	4.875034	-0.194168	-1.037055
2	6	0	3.741764	-0.720844	-0.424879
3	6	0	3.869332	-1.406460	0.784328
4	6	0	5.114412	-1.556863	1.368221
5	6	0	6.243480	-1.025596	0.751719
6	6	0	6.123600	-0.345153	-0.450418
7	6	0	2.427306	-0.514209	-1.054343
8	7	0	1.347117	-0.888955	-0.514529
9	6	0	0.093649	-0.540175	-1.150925
10	6	0	-0.401654	0.769968	-0.536692
11	6	0	-1.849915	1.067993	-0.905559
12	6	0	-2.704649	-0.151929	-0.587517
13	8	0	-2.195664	-1.288876	-1.278104
14	6	0	-0.911635	-1.672781	-0.918064
15	8	0	0.501873	1.770127	-0.971074
16	8	0	-0.905254	-2.069201	0.432729
17	6	0	-4.142345	0.003637	-1.032029
18	8	0	-4.976226	-0.997957	-0.489129
19	8	0	-2.351521	2.138383	-0.130277
20	1	0	2.429740	0.016985	-2.012792
21	1	0	0.202378	-0.384873	-2.230988
22	1	0	0.142750	2.664502	-0.818068
23	1	0	-0.360989	0.673573	0.557047
24	1	0	-2.009592	2.983421	-0.457119
25	1	0	-1.915236	1.290301	-1.979149
26	1	0	-4.484296	-1.839173	-0.428421
27	1	0	-4.517250	0.968026	-0.690361
28	1	0	-4.172153	-0.020938	-2.127666
29	1	0	-2.681658	-0.327088	0.495116
30	1	0	0.004302	-1.985114	0.751571
31	1	0	-0.670951	-2.524865	-1.559315
32	1	0	4.774303	0.340162	-1.974687
33	1	0	7.000405	0.069585	-0.929866
34	1	0	7.216060	-1.144334	1.211677
35	1	0	5.210787	-2.090484	2.304822
36	1	0	2.983161	-1.820398	1.248586
37	1	0	-3.942274	-2.603254	1.597252

38	8	0	-3.633518	-3.091500	0.818859
39	1	0	-2.680262	-2.937167	0.780581
40	1	0	-0.441780	4.082154	0.649799
41	8	0	-0.562841	4.261577	-0.314014
42	1	0	-0.273285	5.155058	-0.508295
43	8	0	-0.409805	3.114953	2.063548
44	1	0	0.400350	2.573543	2.082892
45	1	0	-1.145531	2.514138	1.888621
46	1	0	1.717092	1.722947	0.490837
47	8	0	1.965729	1.768772	1.430197
48	1	0	2.448712	0.957786	1.616211
49	8	0	-4.793904	-0.950316	2.267062
50	1	0	-5.080155	-0.822844	1.343341
51	1	0	-5.565671	-0.817187	2.820963

Structure 30.5H_2O (M06-2X/def2-TZVP, DMSO)

Energy (Hartrees): = -1318.723120
No imaginary frequencies

Standard orientation:

Center Number	Atomic Number	Atomic Type	Coordinates (Angstroms) X	Y	Z
1	6	0	4.947151	-0.401813	-1.166506
2	6	0	3.813430	-0.689417	-0.410098
3	6	0	3.957313	-1.131569	0.906792
4	6	0	5.220166	-1.279382	1.453876
5	6	0	6.350147	-0.988693	0.693314
6	6	0	6.213410	-0.550693	-0.616544
7	6	0	2.489942	-0.497905	-1.023978
8	7	0	1.408084	-0.703572	-0.399050
9	6	0	0.156401	-0.433502	-1.076377
10	6	0	-0.434491	0.848963	-0.496869
11	6	0	-1.877877	1.063915	-0.929020
12	6	0	-2.676030	-0.193869	-0.613480
13	8	0	-2.083993	-1.323903	-1.248977
14	6	0	-0.784962	-1.618976	-0.845173
15	8	0	0.425274	1.903188	-0.894619
16	8	0	-0.803202	-1.992551	0.515559
17	6	0	-4.099466	-0.134244	-1.114081
18	8	0	-4.863712	-1.231769	-0.636531
19	8	0	-2.463269	2.122369	-0.187211
20	1	0	2.490732	-0.152523	-2.062789
21	1	0	0.284003	-0.302569	-2.156970
22	1	0	0.003177	2.771207	-0.755796
23	1	0	-0.438778	0.756806	0.598296
24	1	0	-2.165316	2.975401	-0.536860
25	1	0	-1.920051	1.268626	-2.005557
26	1	0	-4.282424	-2.007743	-0.545996
27	1	0	-4.574314	0.779255	-0.757226
28	1	0	-4.093070	-0.121664	-2.209232
29	1	0	-2.682461	-0.338687	0.474367
30	1	0	0.073423	-1.801620	0.883494
31	1	0	-0.471257	-2.466282	-1.458995
32	1	0	4.831511	-0.056769	-2.187805
33	1	0	7.090616	-0.322207	-1.208599
34	1	0	7.336272	-1.105036	1.125721
35	1	0	5.329195	-1.623618	2.474675
36	1	0	3.074947	-1.362090	1.492183
37	1	0	-3.871643	-2.696503	1.537655
38	8	0	-3.416432	-3.220561	0.859234
39	1	0	-2.506875	-2.887842	0.839031
40	1	0	-0.687402	4.251859	0.623171
41	8	0	-0.811405	4.362255	-0.341398
42	1	0	-0.538390	5.251981	-0.590883
43	8	0	-0.666698	3.138006	2.069655
44	1	0	0.162852	2.624549	2.057195
45	1	0	-1.350336	2.545453	1.725607
46	1	0	1.596005	1.840907	0.614388
47	8	0	1.798108	1.822036	1.566781
48	1	0	1.957854	0.888655	1.757229
49	8	0	-5.004052	-1.243482	2.139762
50	1	0	-5.177317	-1.092318	1.192375
51	1	0	-5.863285	-1.333824	2.565493

Structure 30.5H_2O (M06-2X/def2-TZVP, H_2O)

Energy (Hartrees): = -1318.739212
No imaginary frequencies

Standard orientation:

Center Number	Atomic Number	Atomic Type	Coordinates (Angstroms) X	Y	Z
1	6	0	4.925048	-0.476376	-1.219968
2	6	0	3.826442	-0.683734	-0.388330
3	6	0	4.031848	-0.980544	0.960428
4	6	0	5.319234	-1.079418	1.459977
5	6	0	6.413109	-0.881248	0.621655
6	6	0	6.215743	-0.578347	-0.718430
7	6	0	2.479937	-0.566151	-0.968538
8	7	0	1.419947	-0.862011	-0.337770
9	6	0	0.156712	-0.612572	-1.002446
10	6	0	-0.417717	0.705758	-0.489575
11	6	0	-1.842062	0.914610	-0.982257
12	6	0	-2.693014	-0.305886	-0.655004
13	8	0	-2.090716	-1.492574	-1.168894
14	6	0	-0.798482	-1.766082	-0.701329
15	8	0	0.456667	1.731090	-0.931645
16	8	0	-0.855101	-2.032529	0.682909
17	6	0	-4.058322	-0.218192	-1.299173
18	8	0	-4.903604	-1.288719	-0.897938
19	8	0	-2.444345	2.019494	-0.323065
20	1	0	2.442764	-0.202980	-2.000533
21	1	0	0.267328	-0.539203	-2.090081
22	1	0	0.072326	2.612996	-0.752782
23	1	0	-0.446540	0.681388	0.608023
24	1	0	-2.092779	2.845848	-0.687527
25	1	0	-1.831349	1.073894	-2.066763
26	1	0	-4.427814	-2.119861	-1.030499
27	1	0	-4.550439	0.703039	-0.992514
28	1	0	-3.941033	-0.214033	-2.387092
29	1	0	-2.800979	-0.386623	0.433188
30	1	0	-0.017872	-1.750664	1.081609
31	1	0	-0.484718	-2.663015	-1.238075
32	1	0	4.760717	-0.237094	-2.264454
33	1	0	7.063694	-0.419194	-1.372080
34	1	0	7.417865	-0.959914	1.017362
35	1	0	5.475849	-1.307600	2.506579
36	1	0	3.181900	-1.125796	1.613947
37	1	0	-3.929982	-2.108938	1.848050
38	8	0	-3.359668	-2.889023	1.744331
39	1	0	-2.518789	-2.548848	1.399668
40	1	0	-0.660794	4.155942	0.505032
41	8	0	-0.669268	4.255317	-0.465257
42	1	0	-0.144688	5.036691	-0.673778
43	8	0	-0.849526	3.044648	2.037234
44	1	0	0.024922	2.620006	1.949107
45	1	0	-1.448319	2.506696	1.495877
46	1	0	1.568303	1.866190	0.629405
47	8	0	1.767093	1.966402	1.577604
48	1	0	2.423157	2.671836	1.626214
49	8	0	-5.221942	-0.697357	1.802218
50	1	0	-5.279725	-0.920746	0.853484
51	1	0	-6.050204	-1.000835	2.190989

Structure 45 (M06-2X/def2-TZVP, Gas Phase)

Energy (Hartrees): = - 1013.8594083
No imaginary frequencies

Standard orientation:

Center Number	Atomic Number	Atomic Type	Coordinates (Angstroms) X	Y	Z
1	6	0	-2.029211	-1.302366	0.475879
2	6	0	-1.416210	-0.078380	-0.203605
3	6	0	-2.325519	1.118012	0.042511
4	6	0	-3.739921	0.806910	-0.392609
5	6	0	-4.228957	-0.444830	0.328205
6	1	0	-2.047142	-1.153374	1.566955
7	1	0	-2.332096	1.334916	1.119097
8	1	0	-3.741355	0.605574	-1.472373

9	1	0	-4.241269	-0.252394	1.411110
10	1	0	-1.381679	-0.299271	-1.279468
11	8	0	-3.348089	-1.509350	0.019660
12	6	0	-5.626491	-0.863975	-0.102631
13	1	0	-5.661845	-0.914031	-1.198458
14	1	0	-5.819996	-1.864527	0.285168
15	8	0	-6.618751	-0.013099	0.416003
16	1	0	-6.368635	0.895112	0.205876
17	8	0	-4.608878	1.881889	-0.092657
18	1	0	-4.189081	2.690304	-0.409804
19	8	0	-1.906166	2.259148	-0.678239
20	1	0	-1.022506	2.495871	-0.375513
21	8	0	-1.271979	-2.405893	0.124669
22	1	0	-1.664884	-3.187857	0.526168
23	7	0	-0.112697	0.211436	0.346368
24	6	0	0.902795	-0.130195	-0.331728
25	1	0	0.803470	-0.619887	-1.308550
26	6	0	2.255462	0.103869	0.147693
27	6	0	3.323447	-0.250005	-0.573111
28	6	0	4.731648	-0.071638	-0.212425
29	6	0	5.135445	0.540672	0.977769
30	6	0	5.715005	-0.528886	-1.090869
31	6	0	6.478262	0.687070	1.274458
32	1	0	4.395367	0.907663	1.676779
33	6	0	7.061693	-0.383167	-0.794488
34	1	0	5.414351	-1.005401	-2.016683
35	6	0	7.447386	0.225833	0.389949
36	1	0	6.774500	1.164305	2.199684
37	1	0	7.808531	-0.746036	-1.488679
38	1	0	8.497192	0.342800	0.625297
39	1	0	3.149895	-0.725444	-1.535740
40	1	0	2.337967	0.576515	1.119105

Structure 45 (M06-2X/def2-TZVP, DMSO)

Energy (Hartrees): = - 1013.8887671
No imaginary frequencies

Standard orientation:

Center Number	Atomic Number	Atomic Type	Coordinates (Angstroms) X	Y	Z
1	6	0	2.190886	1.499982	0.279155
2	6	0	1.421423	0.267878	-0.189237
3	6	0	2.190876	-0.982414	0.235739
4	6	0	3.624325	-0.910359	-0.244561
5	6	0	4.271531	0.379238	0.248962
6	1	0	2.235149	1.519786	1.377734
7	1	0	2.193525	-1.040045	1.331580
8	1	0	3.632976	-0.903090	-1.342301
9	1	0	4.290076	0.374777	1.348326
10	1	0	1.370796	0.301713	-1.286142
11	8	0	3.511700	1.473889	-0.229126
12	6	0	5.688984	0.557730	-0.270319
13	1	0	5.685551	0.454235	-1.362539
14	1	0	6.024747	1.566676	-0.026878
15	8	0	6.597024	-0.343742	0.329241
16	1	0	6.200408	-1.223378	0.278814
17	8	0	4.374564	-2.011415	0.242992
18	1	0	3.890625	-2.817547	0.020747
19	8	0	1.628173	-2.158507	-0.314938
20	1	0	0.770638	-2.313411	0.101119
21	8	0	1.560419	2.624671	-0.222544
22	1	0	1.963002	3.406431	0.176604
23	7	0	0.113649	0.237177	0.422489
24	6	0	-0.896775	0.143217	-0.341825
25	1	0	-0.790179	0.104424	-1.432310
26	6	0	-2.251131	0.089714	0.185160
27	6	0	-3.306698	0.008110	-0.632547
28	6	0	-4.717143	-0.045336	-0.239234
29	6	0	-5.132778	0.018839	1.095406
30	6	0	-5.688124	-0.164091	-1.236769
31	6	0	-6.478435	-0.035621	1.415805
32	1	0	-4.401822	0.114941	1.888494
33	6	0	-7.037158	-0.219187	-0.915203
34	1	0	-5.374900	-0.212878	-2.273558
35	6	0	-7.436093	-0.154446	0.412486
36	1	0	-6.785859	0.015941	2.453000
37	1	0	-7.775804	-0.312435	-1.701638
38	1	0	-8.487756	-0.195204	0.667717
39	1	0	-3.121479	-0.023313	-1.703886
40	1	0	-2.352422	0.123450	1.264600

Structure 45.5H_2O (M06-2X/def2-TZVP, Gas Phase)

Energy (Hartrees): = - 1396.0791632
No imaginary frequencies

Standard orientation:

Center Number	Atomic Number	Atomic Type	Coordinates (Angstroms) X	Y	Z
1	6	0	-1.544765	-0.371086	0.449896
2	7	0	-0.542401	0.335296	0.126945
3	6	0	0.774166	-0.129173	0.502769
4	6	0	1.394040	-0.916788	-0.663664
5	8	0	2.720023	-1.282108	-0.326505
6	6	0	3.623113	-0.200826	-0.101221
7	6	0	3.111129	0.606297	1.085429
8	6	0	1.678855	1.051801	0.820691
9	8	0	0.706455	-2.078101	-0.961646
10	6	0	4.983433	-0.824568	0.141507
11	8	0	5.464349	-1.515822	-0.975730
12	8	0	3.912009	1.717950	1.398145
13	8	0	1.173127	1.748561	1.945374
14	8	0	3.709823	3.061626	-0.989271
15	8	0	1.366889	4.357227	-1.355521
16	8	0	-0.567347	3.180403	0.123684
17	8	0	2.103761	-3.542148	1.086007
18	8	0	3.709763	-3.809739	-1.383157
19	1	0	-1.427226	-1.299892	1.021541
20	1	0	1.687476	1.718033	-0.054651
21	1	0	1.920472	2.248443	2.303666
22	1	0	3.968776	2.283120	0.601726
23	1	0	3.114229	-0.038351	1.973424
24	1	0	3.661231	0.432082	-0.996333
25	1	0	5.691778	-0.030308	0.379176
26	1	0	4.911435	-1.478090	1.024431
27	1	0	4.942589	-2.325869	-1.102663
28	1	0	0.767812	-2.679829	-0.202034
29	1	0	1.396750	-0.301275	-1.569283
30	1	0	0.735182	-0.803717	1.371912
31	1	0	-0.709499	2.258699	-0.165408
32	1	0	-0.169377	3.043254	0.996257
33	1	0	2.598077	-2.728531	0.886086
34	1	0	2.215527	-3.734838	2.020174
35	1	0	0.613455	3.962685	-0.859149
36	1	0	1.235925	5.307514	-1.346830
37	1	0	2.903864	3.597232	-1.157745
38	1	0	4.331821	3.243294	-1.696569
39	1	0	2.988773	-3.239791	-1.678175
40	1	0	3.354310	-4.228516	-0.589737
41	6	0	-2.900091	0.005755	0.091180
42	6	0	-3.943494	-0.766249	0.410059
43	6	0	-5.352584	-0.508431	0.110160
44	6	0	-5.803554	0.717343	-0.388396
45	6	0	-6.285135	-1.523109	0.330556
46	6	0	-7.144317	0.912037	-0.666908
47	1	0	-5.103298	1.527045	-0.546828
48	6	0	-7.628648	-1.329984	0.048051
49	1	0	-5.946245	-2.475223	0.721938
50	6	0	-8.061361	-0.111286	-0.452850
51	1	0	-7.480313	1.867345	-1.048809
52	1	0	-8.337360	-2.129459	0.220282
53	1	0	-9.109517	0.045702	-0.671794
54	1	0	-3.743654	-1.697382	0.935328
55	1	0	-3.011726	0.936367	-0.453122

Structure 45.5H_2O (M06-2X/def2-TZVP, DMSO)

Energy (Hartrees): = - 1396.1218781
No imaginary frequencies

Standard orientation:

Center Number	Atomic Number	Atomic Type	Coordinates (Angstroms) X	Y	Z
1	6	0	-1.551355	-0.351819	0.396834
2	7	0	-0.535695	0.303859	0.003416
3	6	0	0.771481	-0.159813	0.415464
4	6	0	1.438223	-0.905604	-0.750130
5	8	0	2.748891	-1.288827	-0.367978
6	6	0	3.641923	-0.221961	-0.064522
7	6	0	3.076786	0.569595	1.110547
8	6	0	1.658396	1.019435	0.787704
9	8	0	0.769777	-2.059772	-1.115220

10	6	0	4.974669	-0.869066	0.252287
11	8	0	5.499331	-1.582601	-0.842359
12	8	0	3.869698	1.679158	1.459033
13	8	0	1.098134	1.703302	1.897933
14	8	0	3.884682	3.383391	-0.695640
15	8	0	1.420707	4.331642	-1.348730
16	8	0	-0.583111	3.148489	0.073764
17	8	0	2.036973	-3.631580	0.948829
18	8	0	3.685327	-3.804496	-1.431867
19	1	0	-1.448438	-1.225255	1.050543
20	1	0	1.703050	1.697488	-0.077833
21	1	0	1.819649	2.210812	2.298144
22	1	0	3.914879	2.283705	0.687276
23	1	0	3.043529	-0.086512	1.987877
24	1	0	3.740238	0.429186	-0.944218
25	1	0	5.692510	-0.092193	0.516663
26	1	0	4.843903	-1.521595	1.126360
27	1	0	4.938147	-2.359324	-1.003411
28	1	0	0.785032	-2.672923	-0.360629
29	1	0	1.483336	-0.260378	-1.633784
30	1	0	0.708658	-0.855167	1.265407
31	1	0	-0.716051	2.233339	-0.243761
32	1	0	-0.159398	2.984950	0.931120
33	1	0	2.515190	-2.788655	0.983860
34	1	0	1.928000	-3.933619	1.858429
35	1	0	0.657497	3.939566	-0.869655
36	1	0	1.285452	5.285279	-1.345037
37	1	0	3.004680	3.743498	-0.941972
38	1	0	4.289251	3.075392	-1.514053
39	1	0	3.038864	-3.116076	-1.640208
40	1	0	3.331467	-4.178355	-0.612272
41	6	0	-2.901522	0.016019	0.008864
42	6	0	-3.948824	-0.700937	0.433827
43	6	0	-5.361151	-0.468880	0.125458
44	6	0	-5.797799	0.570885	-0.702954
45	6	0	-6.313398	-1.326137	0.682755
46	6	0	-7.147073	0.742891	-0.961010
47	1	0	-5.081052	1.249253	-1.148688
48	6	0	-7.665607	-1.153053	0.424111
49	1	0	-5.983236	-2.135413	1.324125
50	6	0	-8.086084	-0.117534	-0.398737
51	1	0	-7.471219	1.552013	-1.603655
52	1	0	-8.389662	-1.827911	0.863398
53	1	0	-9.140242	0.021684	-0.603872
54	1	0	-3.750778	-1.554789	1.077868
55	1	0	-3.008660	0.881207	-0.637049

Structure 45.5H_2O (M06-2X/def2-TZVP, H_2O)

Energy (Hartrees): = - 1396.1406726
No imaginary frequencies

Standard orientation:

Center Number	Atomic Number	Atomic Type	Coordinates (Angstroms) X	Y	Z
1	6	0	-1.644746	-0.456076	0.353348
2	7	0	-0.625714	0.229963	0.020721
3	6	0	0.685444	-0.267158	0.374903
4	6	0	1.427579	-0.680324	-0.897174
5	8	0	2.750035	-1.073615	-0.567948
6	6	0	3.547374	-0.039011	0.009762
7	6	0	2.912993	0.391451	1.330609
8	6	0	1.473954	0.821508	1.086389
9	8	0	0.822040	-1.760333	-1.533903
10	6	0	4.942202	-0.593784	0.200125
11	8	0	5.575004	-0.900589	-1.029153
12	8	0	3.635316	1.434427	1.953821
13	8	0	0.814466	1.110342	2.306030
14	8	0	3.892635	3.427889	0.056457
15	8	0	1.946390	2.969324	-1.848986
16	8	0	-0.600320	2.926419	-0.763554
17	8	0	2.437267	-3.302117	1.269958
18	8	0	4.172720	-3.345849	-1.582501
19	1	0	-1.540735	-1.399369	0.899166
20	1	0	1.492925	1.717259	0.450166
21	1	0	1.362943	1.744333	2.786849
22	1	0	3.709526	2.177793	1.315490
23	1	0	2.918267	-0.461129	2.018407
24	1	0	3.576696	0.814403	-0.681197
25	1	0	5.548230	0.158173	0.704973
26	1	0	4.888555	-1.481445	0.840868
27	1	0	5.220939	-1.751625	-1.338031
28	1	0	0.653085	-2.453134	-0.877207
29	1	0	1.461174	0.144849	-1.615050
30	1	0	0.630296	-1.152785	1.025198

31	1	0	-0.664739	1.971537	-0.517062
32	1	0	-0.530191	3.393503	0.078047
33	1	0	2.606324	-2.556248	0.671143
34	1	0	2.015345	-2.899003	2.038114
35	1	0	1.037919	2.979544	-1.477590
36	1	0	1.957355	3.657187	-2.524831
37	1	0	3.198797	3.267196	-0.619127
38	1	0	4.720377	3.174375	-0.369279
39	1	0	3.434415	-2.725406	-1.478595
40	1	0	4.228876	-3.788899	-0.726249
41	6	0	-2.991223	-0.024142	0.030304
42	6	0	-4.049846	-0.761538	0.384883
43	6	0	-5.456997	-0.450059	0.126468
44	6	0	-5.865622	0.723588	-0.517044
45	6	0	-6.430984	-1.361773	0.540088
46	6	0	-7.209513	0.970575	-0.738787
47	1	0	-5.131462	1.448019	-0.846344
48	6	0	-7.778068	-1.114062	0.316463
49	1	0	-6.121794	-2.271630	1.041408
50	6	0	-8.170723	0.053119	-0.323917
51	1	0	-7.512419	1.882011	-1.238500
52	1	0	-8.519652	-1.831334	0.644517
53	1	0	-9.220455	0.250604	-0.500275
54	1	0	-3.868867	-1.692885	0.916224
55	1	0	-3.087089	0.913312	-0.506573

Structure 50 (M06-2X/def2-TZVP, Gas Phase)

Energy (Hartrees): = - 1090.0946489
No imaginary frequencies

Standard orientation:

Center Number	Atomic Number	Atomic Type	Coordinates (Angstroms) X	Y	Z
1	6	0	2.003819	0.686240	-1.247561
2	6	0	1.315692	-0.090497	-0.125962
3	6	0	2.224138	-0.086175	1.095908
4	6	0	3.598310	-0.603296	0.733807
5	6	0	4.168700	0.225493	-0.412246
6	1	0	2.118855	1.742332	-0.956270
7	1	0	2.324031	0.947340	1.453968
8	1	0	3.506159	-1.643804	0.393884
9	1	0	4.278233	1.268029	-0.079208
10	1	0	1.191309	-1.120593	-0.487831
11	8	0	3.278468	0.142251	-1.510357
12	6	0	5.526220	-0.276246	-0.881379
13	1	0	5.461324	-1.355330	-1.071330
14	1	0	5.767814	0.216092	-1.823747
15	8	0	6.550310	0.035710	0.030206
16	1	0	6.272550	-0.267161	0.903457
17	8	0	4.475221	-0.524067	1.840510
18	1	0	4.018037	-0.904049	2.600124
19	8	0	1.725158	-0.910203	2.129915
20	1	0	0.868599	-0.563584	2.403837
21	8	0	1.231963	0.553081	-2.387904
22	1	0	1.672162	1.009800	-3.112459
23	7	0	0.059428	0.526669	0.228236
24	6	0	-2.338434	0.542547	0.083111
25	6	0	-2.437290	1.861903	0.454425
26	6	0	-3.521248	-0.245610	-0.066641
27	6	0	-3.685348	2.463240	0.687569
28	1	0	-1.524754	2.435010	0.553403
29	6	0	-4.780636	0.372282	0.164840
30	6	0	-4.832037	1.734258	0.540747
31	1	0	-3.731987	3.505836	0.973732
32	6	0	-1.003426	-0.027639	-0.178312
33	1	0	-0.953573	-0.947410	-0.766936
34	6	0	-5.968217	-0.386607	0.022809
35	6	0	-3.510322	-1.621084	-0.416074
36	6	0	-4.673830	-2.326458	-0.543942
37	6	0	-5.920897	-1.704420	-0.326229
38	1	0	-2.572561	-2.132713	-0.576914
39	1	0	-4.639044	-3.374536	-0.812243
40	1	0	-6.833844	-2.275765	-0.431516
41	1	0	-5.801288	2.188618	0.709665
42	1	0	-6.918146	0.103455	0.200562

Structure 50 (M06-2X/def2-TZVP, DMSO)

Energy (Hartrees): = - 1090.1244797
No imaginary frequencies

Standard orientation:

Center Number	Atomic Number	Atomic Type	Coordinates (Angstroms) X	Y	Z
1	6	0	-2.225672	1.480826	0.516353
2	6	0	-1.330527	0.258838	0.338955
3	6	0	-2.001181	-0.704834	-0.640491
4	6	0	-3.414095	-1.012731	-0.191931
5	6	0	-4.191258	0.286641	-0.012489
6	1	0	-2.318028	2.016480	-0.439664
7	1	0	-2.045045	-0.228375	-1.628017
8	1	0	-3.375748	-1.531298	0.774857
9	1	0	-4.245759	0.809268	-0.978097
10	1	0	-1.230330	-0.233579	1.315480
11	8	0	-3.515791	1.084737	0.942458
12	6	0	-5.602588	0.060145	0.504692
13	1	0	-5.561413	-0.564514	1.405607
14	1	0	-6.030121	1.024515	0.782547
15	8	0	-6.446534	-0.506305	-0.476998
16	1	0	-5.980560	-1.267258	-0.847509
17	8	0	-4.084183	-1.815041	-1.150934
18	1	0	-3.524487	-2.580424	-1.336180
19	8	0	-1.311581	-1.937616	-0.721341
20	1	0	-0.467113	-1.790083	-1.165763
21	8	0	-1.675704	2.286528	1.497071
22	1	0	-2.151881	3.126517	1.507941
23	7	0	-0.056645	0.659440	-0.211533
24	6	0	2.340437	0.621402	-0.113421
25	6	0	2.506119	1.799380	-0.801648
26	6	0	3.473284	-0.204832	0.164895
27	6	0	3.776118	2.218756	-1.238482
28	1	0	1.639371	2.419382	-0.993527
29	6	0	4.753667	0.226730	-0.278652
30	6	0	4.876633	1.450796	-0.979038
31	1	0	3.875001	3.156442	-1.770517
32	6	0	0.993335	0.247360	0.364192
33	1	0	0.930843	-0.379174	1.258211
34	6	0	5.890916	-0.578112	-0.018527
35	6	0	3.381711	-1.452497	0.835995
36	6	0	4.497769	-2.208603	1.067741
37	6	0	5.769495	-1.767544	0.641906
38	1	0	2.418973	-1.823195	1.160771
39	1	0	4.405703	-3.158250	1.580154
40	1	0	6.642582	-2.377446	0.837460
41	1	0	5.861677	1.765131	-1.304249
42	1	0	6.858223	-0.228961	-0.361121

Structure 50.5H_2O (M06-2X/def2-TZVP, Gas Phase)

Energy (Hartrees): = - 1472.3143964
No imaginary frequencies

Standard orientation:

Center Number	Atomic Number	Atomic Type	Coordinates (Angstroms) X	Y	Z
1	6	0	-3.261119	1.878042	0.504292
2	6	0	-2.964680	0.543471	0.387496
3	6	0	-3.982579	-0.391146	0.032778
4	6	0	-5.304353	0.084619	-0.163611
5	6	0	-5.572035	1.467414	-0.019637
6	6	0	-4.574084	2.344292	0.296662
7	6	0	-1.603618	0.060347	0.681936
8	7	0	-0.559562	0.678165	0.323881
9	6	0	0.719329	0.078679	0.661177
10	6	0	1.109800	-0.928520	-0.435395
11	8	0	2.406480	-1.431700	-0.174602
12	6	0	3.459469	-0.468482	-0.166897
13	6	0	3.179740	0.527467	0.951400
14	6	0	1.800570	1.142259	0.750214
15	8	0	0.254412	-2.015225	-0.504167
16	6	0	4.746889	-1.244504	0.030129
17	8	0	5.020907	-2.109417	-1.034552
18	8	0	4.147101	1.540244	1.061901
19	8	0	1.508938	2.031007	1.814961
20	8	0	3.896838	2.624875	-1.441810
21	8	0	1.786343	4.284388	-1.722278
22	8	0	-0.151976	3.505271	0.004528
23	8	0	1.659877	-3.424203	1.552508
24	8	0	2.959432	-4.165205	-1.003764
25	1	0	-1.532678	-0.872211	1.251495
26	1	0	1.814198	1.695926	-0.200391
27	1	0	2.351023	2.446585	2.050257
28	1	0	4.202332	2.002111	0.200715

29	1	0	3.183920	-0.011493	1.907551
30	1	0	3.485783	0.049621	-1.133665
31	1	0	5.569835	-0.533716	0.110727
32	1	0	4.682111	-1.784082	0.987480
33	1	0	4.393582	-2.851406	-1.016945
34	1	0	0.365385	-2.543293	0.304262
35	1	0	1.089130	-0.437797	-1.414531
36	1	0	0.669728	-0.471088	1.612523
37	1	0	-2.481179	2.575155	0.784663
38	1	0	-4.785420	3.400114	0.402516
39	1	0	-0.474851	2.612043	-0.215285
40	1	0	0.321625	3.336029	0.834159
41	1	0	2.248681	-2.718583	1.235668
42	1	0	1.797778	-3.523803	2.497818
43	1	0	1.028403	4.044608	-1.142522
44	1	0	1.809550	5.242444	-1.766073
45	1	0	3.182280	3.282154	-1.592646
46	1	0	4.490247	2.662621	-2.194671
47	1	0	2.287418	-3.523550	-1.265308
48	1	0	2.652338	-4.477314	-0.144141
49	1	0	-6.586331	1.818085	-0.169472
50	6	0	-3.719875	-1.769063	-0.171821
51	6	0	-6.324249	-0.831749	-0.515423
52	6	0	-6.045820	-2.158474	-0.681083
53	1	0	-6.831917	-2.850778	-0.952844
54	6	0	-4.726254	-2.628306	-0.515321
55	1	0	-4.508542	-3.676519	-0.674078
56	1	0	-2.706458	-2.141161	-0.089924
57	1	0	-7.330629	-0.456211	-0.657694

Structure 50.5H_2O (M06-2X/def2-TZVP, DMSO)

Energy (Hartrees): = -1472.3553417
No imaginary frequencies

Standard orientation:

Center Number	Atomic Number	Atomic Type	Coordinates (Angstroms) X	Y	Z
1	6	0	-3.179009	1.933294	0.208630
2	6	0	-2.961512	0.578372	0.261299
3	6	0	-4.052001	-0.327111	0.078196
4	6	0	-5.347788	0.208432	-0.154074
5	6	0	-5.526309	1.612503	-0.200641
6	6	0	-4.465711	2.455918	-0.025277
7	6	0	-1.611560	0.060697	0.555372
8	7	0	-0.554416	0.624757	0.142509
9	6	0	0.705147	0.001573	0.505302
10	6	0	1.198785	-0.878330	-0.653982
11	8	0	2.440705	-1.455517	-0.288250
12	6	0	3.506514	-0.536766	-0.064040
13	6	0	3.117929	0.401644	1.075001
14	6	0	1.774962	1.050730	0.766717
15	8	0	0.343326	-1.918932	-0.963414
16	6	0	4.724337	-1.374004	0.268841
17	8	0	5.098500	-2.226857	-0.786919
18	8	0	4.086859	1.392872	1.322754
19	8	0	1.376284	1.890622	1.838570
20	8	0	4.231221	2.717275	-1.073484
21	8	0	2.116757	4.368829	-1.555916
22	8	0	-0.022026	3.456587	-0.107390
23	8	0	1.335033	-3.584487	1.141982
24	8	0	2.927076	-4.137766	-1.231182
25	1	0	-1.555340	-0.838740	1.175062
26	1	0	1.892194	1.651873	-0.147402
27	1	0	2.186444	2.297779	2.180210
28	1	0	4.216074	1.905665	0.496880
29	1	0	3.020125	-0.185366	1.995681
30	1	0	3.685474	0.042320	-0.979726
31	1	0	5.564015	-0.708476	0.472046
32	1	0	4.515343	-1.943267	1.184937
33	1	0	4.416755	-2.911484	-0.889056
34	1	0	0.297428	-2.512530	-0.193993
35	1	0	1.318986	-0.272750	-1.558898
36	1	0	0.596430	-0.641426	1.390088
37	1	0	-2.354130	2.612864	0.380191
38	1	0	-4.607842	3.528689	-0.053109
39	1	0	-0.327364	2.568144	-0.370981
40	1	0	0.371264	3.267032	0.759916
41	1	0	1.968559	-2.852197	1.186736
42	1	0	1.156991	-3.860596	2.049232
43	1	0	1.310625	4.075480	-1.079705
44	1	0	2.213234	5.308767	-1.367983
45	1	0	3.509313	3.353417	-1.267924
46	1	0	5.033923	3.073308	-1.468687

47	1	0	2.422348	-3.345942	-1.462974
48	1	0	2.517392	-4.406221	-0.396852
49	1	0	-6.522260	2.003581	-0.374276
50	6	0	-3.897559	-1.737305	0.090221
51	6	0	-6.441885	-0.671980	-0.342048
52	6	0	-6.262691	-2.025995	-0.309869
53	1	0	-7.102752	-2.693489	-0.454816
54	6	0	-4.974027	-2.561138	-0.095347
55	1	0	-4.836804	-3.635195	-0.082905
56	1	0	-2.917443	-2.173128	0.234509
57	1	0	-7.423677	-0.245609	-0.513590

Structure 50.5H_2O (M06-2X/def2-TZVP, H_2O)

Energy (Hartrees): = - 1472.3756944
No imaginary frequencies

Standard orientation:

Center Number	Atomic Number	Atomic Type	Coordinates (Angstroms) X	Y	Z
1	6	0	-3.178498	1.828870	-0.033232
2	6	0	-3.041447	0.475205	0.160584
3	6	0	-4.193966	-0.369249	0.139848
4	6	0	-5.467374	0.224998	-0.068585
5	6	0	-5.563285	1.625114	-0.255844
6	6	0	-4.443832	2.409146	-0.244107
7	6	0	-1.710599	-0.090812	0.440615
8	7	0	-0.646920	0.409914	-0.034543
9	6	0	0.615718	-0.176841	0.358556
10	6	0	1.376800	-0.615104	-0.891317
11	8	0	2.646032	-1.123646	-0.514932
12	6	0	3.502321	-0.181582	0.135530
13	6	0	2.831897	0.294963	1.422552
14	6	0	1.447526	0.848717	1.112502
15	8	0	0.715393	-1.623831	-1.586758
16	6	0	4.813693	-0.885970	0.407932
17	8	0	5.518261	-1.203715	-0.778713
18	8	0	3.609151	1.263612	2.095820
19	8	0	0.758190	1.181369	2.303367
20	8	0	3.880431	3.403494	0.382330
21	8	0	2.587134	2.725438	-1.942608
22	8	0	-0.156697	2.553245	-1.789206
23	8	0	2.041679	-3.414776	1.172763
24	8	0	3.923636	-3.395383	-1.694876
25	1	0	-1.661806	-0.958357	1.102367
26	1	0	1.563255	1.741486	0.480401
27	1	0	1.337536	1.756830	2.820641
28	1	0	3.715932	2.039410	1.501679
29	1	0	2.723469	-0.558790	2.100560
30	1	0	3.668218	0.672296	-0.534257
31	1	0	5.447016	-0.227449	1.001381
32	1	0	4.610650	-1.789837	0.994001
33	1	0	5.099753	-1.987350	-1.174573
34	1	0	0.475497	-2.325059	-0.962024
35	1	0	1.508647	0.224116	-1.581801
36	1	0	0.474656	-1.062475	0.995273
37	1	0	-2.301159	2.462437	0.004943
38	1	0	-4.522219	3.479360	-0.384805
39	1	0	-0.395945	1.825200	-1.166907
40	1	0	-0.557732	3.348001	-1.417991
41	1	0	2.314690	-2.643726	0.649173
42	1	0	1.618378	-3.035799	1.952078
43	1	0	1.607802	2.684565	-1.883919
44	1	0	2.781438	3.338828	-2.660605
45	1	0	3.391696	3.182625	-0.440447
46	1	0	3.436929	4.179573	0.743610
47	1	0	3.234246	-2.749830	-1.471817
48	1	0	3.904404	-4.029946	-0.967852
49	1	0	-6.543926	2.060920	-0.407871
50	6	0	-4.124015	-1.778558	0.288043
51	6	0	-6.623224	-0.594372	-0.094930
52	6	0	-6.523933	-1.947161	0.066581
53	1	0	-7.411006	-2.567185	0.045083
54	6	0	-5.257889	-2.542949	0.254162
55	1	0	-5.185389	-3.617121	0.368692
56	1	0	-3.165191	-2.262484	0.416519
57	1	0	-7.586431	-0.122584	-0.250562

Structure 50b (M06-2X/def2-TZVP, Gas Phase)

Energy (Hartrees): = - 1090.0954866
No imaginary frequencies

Standard orientation:

Center Number	Atomic Number	Atomic Type	Coordinates (Angstroms) X	Y	Z
1	6	0	1.831922	-0.690037	-1.261934
2	6	0	1.232233	-0.582545	0.139093
3	6	0	1.982091	0.495579	0.910050
4	6	0	3.469306	0.222963	0.886692
5	6	0	3.943301	0.114269	-0.558539
6	1	0	1.658892	0.247387	-1.814184
7	1	0	1.799143	1.465058	0.427149
8	1	0	3.660075	-0.736801	1.385527
9	1	0	3.763976	1.073080	-1.066936
10	1	0	1.387566	-1.555632	0.625674
11	8	0	3.220652	-0.928396	-1.187477
12	6	0	5.423812	-0.217408	-0.669108
13	1	0	5.643207	-1.090477	-0.041314
14	1	0	5.640080	-0.485896	-1.703501
15	8	0	6.237583	0.881831	-0.341730
16	1	0	5.948056	1.219979	0.514689
17	8	0	4.182476	1.261121	1.529859
18	1	0	3.751352	1.427803	2.376535
19	8	0	1.587882	0.548468	2.266059
20	1	0	0.651540	0.774125	2.301273
21	8	0	1.240463	-1.769891	-1.892120
22	1	0	1.624362	-1.861801	-2.770412
23	7	0	-0.162447	-0.215914	0.065635
24	6	0	-2.481316	-0.966058	0.319122
25	6	0	-3.176257	-2.077886	0.737789
26	6	0	-3.204602	0.209139	-0.073071
27	6	0	-4.580569	-2.101540	0.807065
28	1	0	-2.620668	-2.962880	1.025136
29	6	0	-4.624528	0.177317	0.001105
30	6	0	-5.287057	-0.992439	0.447547
31	1	0	-5.087851	-2.995664	1.143931
32	6	0	-1.016201	-1.112773	0.329731
33	1	0	-0.682801	-2.120425	0.609728
34	6	0	-5.371988	1.317769	-0.376213
35	6	0	-2.589040	1.397479	-0.538733
36	6	0	-3.342746	2.482345	-0.898860
37	6	0	-4.747999	2.449615	-0.816440
38	1	0	-1.514064	1.432472	-0.607556
39	1	0	-2.850824	3.378882	-1.254417
40	1	0	-5.327274	3.317632	-1.103597
41	1	0	-6.369680	-0.987208	0.494136
42	1	0	-6.452563	1.271947	-0.309816

Structure 50b (M06-2X/def2-TZVP, DMSO)

Energy (Hartrees): = - 1090.1243634
No imaginary frequencies

Standard orientation:

Center Number	Atomic Number	Atomic Type	Coordinates (Angstroms) X	Y	Z
1	6	0	-1.883754	0.717000	1.305908
2	6	0	-1.238857	-0.384385	0.470015
3	6	0	-1.941279	-0.440617	-0.886416
4	6	0	-3.434609	-0.594410	-0.694277
5	6	0	-3.959620	0.531103	0.189833
6	1	0	-1.727947	1.692348	0.822629
7	1	0	-1.752669	0.498120	-1.422252
8	1	0	-3.631931	-1.550604	-0.192425
9	1	0	-3.783954	1.493865	-0.310792
10	1	0	-1.394049	-1.339610	0.990531
11	8	0	-3.274964	0.485834	1.428707
12	6	0	-5.442454	0.393881	0.493693
13	1	0	-5.632587	-0.608122	0.898099
14	1	0	-5.709758	1.123934	1.258997
15	8	0	-6.248781	0.655037	-0.636772
16	1	0	-5.889015	0.137234	-1.368815
17	8	0	-4.109361	-0.549062	-1.941252
18	1	0	-3.685028	-1.190666	-2.525850
19	8	0	-1.507857	-1.543307	-1.659594
20	1	0	-0.596706	-1.386737	-1.937906
21	8	0	-1.330230	0.687745	2.573612
22	1	0	-1.634313	1.465737	3.058148
23	7	0	0.160164	-0.093922	0.258194
24	6	0	2.452408	-0.960365	0.339848
25	6	0	3.111535	-2.137149	0.618213
26	6	0	3.209389	0.189711	-0.067280

27	6	0	4.509546	-2.257439	0.508557
28	1	0	2.533467	-2.999651	0.929686
29	6	0	4.623064	0.059461	-0.171402
30	6	0	5.248616	-1.178810	0.120156
31	1	0	4.986207	-3.202911	0.732981
32	6	0	0.988925	-1.020383	0.506283
33	1	0	0.635815	-1.986644	0.885643
34	6	0	5.405305	1.172317	-0.564389
35	6	0	2.637041	1.452271	-0.366308
36	6	0	3.422225	2.509764	-0.744488
37	6	0	4.820903	2.374966	-0.846977
38	1	0	1.568280	1.573691	-0.292923
39	1	0	2.960617	3.464458	-0.965617
40	1	0	5.425274	3.222335	-1.145691
41	1	0	6.326776	-1.246777	0.029539
42	1	0	6.479618	1.045987	-0.634756

Structure 53 (M06-2X/def2-TZVP, Gas Phase)

Energy (Hartrees): = - 1090.1002247
No imaginary frequencies

Standard orientation:

Center Number	Atomic Number	Atomic Type	Coordinates (Angstroms) X	Y	Z
1	6	0	2.273759	1.306173	0.573281
2	6	0	1.624097	0.201464	-0.258270
3	6	0	2.479373	-1.054113	-0.152880
4	6	0	3.911915	-0.750480	-0.530470
5	6	0	4.439326	0.381319	0.344928
6	1	0	2.267797	1.024494	1.637787
7	1	0	2.460126	-1.404511	0.887760
8	1	0	3.938481	-0.417643	-1.576811
9	1	0	4.426811	0.056056	1.395527
10	1	0	1.614872	0.552459	-1.299460
11	8	0	3.607463	1.512231	0.161408
12	6	0	5.859791	0.790142	-0.015336
13	1	0	5.915318	0.973788	-1.096051
14	1	0	6.088519	1.725823	0.495535
15	8	0	6.806950	-0.160480	0.404682
16	1	0	6.522720	-1.024479	0.081802
17	8	0	4.731024	-1.890088	-0.356532
18	1	0	4.282688	-2.635382	-0.773971
19	8	0	2.028262	-2.077844	-1.016753
20	1	0	1.137328	-2.329029	-0.749126
21	8	0	1.565892	2.473518	0.350144
22	1	0	1.975902	3.183371	0.855172
23	7	0	0.301535	-0.097075	0.237332
24	6	0	-2.079745	0.054157	-0.041449
25	6	0	-3.095879	0.488734	-0.851192
26	6	0	-2.381644	-0.629611	1.165060
27	6	0	-4.450725	0.264098	-0.511506
28	1	0	-2.866894	1.014581	-1.772379
29	6	0	-3.676390	-0.859403	1.516575
30	1	0	-1.560037	-0.955551	1.788817
31	6	0	-4.747639	-0.423904	0.691837
32	1	0	-3.910779	-1.381426	2.436757
33	6	0	-0.688072	0.297959	-0.443718
34	1	0	-0.552438	0.848151	-1.383241
35	6	0	-5.514096	0.701584	-1.336056
36	6	0	-6.100146	-0.654161	1.031997
37	6	0	-7.108401	-0.221362	0.215635
38	1	0	-8.140720	-0.404043	0.484860
39	6	0	-6.812571	0.464840	-0.981492
40	1	0	-7.620326	0.802189	-1.617805
41	1	0	-5.280164	1.228246	-2.253614
42	1	0	-6.324380	-1.180875	1.951859

Structure 53 (M06-2X/def2-TZVP, DMSO)

Energy (Hartrees): = - 1090.1298748
No imaginary frequencies

Standard orientation:

Center Number	Atomic Number	Atomic Type	Coordinates (Angstroms) X	Y	Z
1	6	0	2.383771	1.494529	0.199637
2	6	0	1.630007	0.254853	-0.273329

3	6	0	2.393796	-0.987688	0.182238
4	6	0	3.834760	-0.914940	-0.275727
5	6	0	4.470248	0.383261	0.210829
6	1	0	2.409668	1.523043	1.298584
7	1	0	2.377141	-1.031911	1.278536
8	1	0	3.859672	-0.921518	-1.373257
9	1	0	4.476623	0.390900	1.310258
10	1	0	1.606238	0.277292	-1.371528
11	8	0	3.712356	1.470353	-0.287382
12	6	0	5.892640	0.561043	-0.295378
13	1	0	5.902014	0.439047	-1.385674
14	1	0	6.220130	1.575795	-0.065263
15	8	0	6.798635	-0.325104	0.329302
16	1	0	6.409175	-1.208239	0.285314
17	8	0	4.582888	-2.005865	0.236606
18	1	0	4.109976	-2.817721	0.011611
19	8	0	1.845172	-2.171973	-0.364732
20	1	0	0.983130	-2.328752	0.041159
21	8	0	1.755967	2.611751	-0.321306
22	1	0	2.148495	3.398748	0.077641
23	7	0	0.305856	0.226175	0.303621
24	6	0	-2.073591	0.075476	-0.030827
25	6	0	-3.067929	-0.018924	-0.970456
26	6	0	-2.407815	0.123658	1.349233
27	6	0	-4.430274	-0.069554	-0.590480
28	1	0	-2.815656	-0.053429	-2.025485
29	6	0	-3.711347	0.075944	1.742936
30	1	0	-1.611632	0.199937	2.078799
31	6	0	-4.759579	-0.021968	0.788421
32	1	0	-3.970439	0.112266	2.794960
33	6	0	-0.677448	0.130011	-0.488229
34	1	0	-0.535750	0.087069	-1.574213
35	6	0	-5.469789	-0.165489	-1.547229
36	6	0	-6.120795	-0.071260	1.170562
37	6	0	-7.105754	-0.162155	0.224328
38	1	0	-8.145452	-0.197780	0.525555
39	6	0	-6.777452	-0.210372	-1.148888
40	1	0	-7.568462	-0.283797	-1.884814
41	1	0	-5.208659	-0.201096	-2.598690
42	1	0	-6.368047	-0.034921	2.225395

Structure 53.5H_2O (M06-2X/def2-TZVP, Gas Phase)

Energy (Hartrees): = - 1472.3188717
No imaginary frequencies

Standard orientation:

Center Number	Atomic Number	Atomic Type	Coordinates (Angstroms) X	Y	Z
1	6	0	-3.072851	0.954071	-0.659359
2	6	0	-2.730039	-0.176300	0.128055
3	6	0	-3.716406	-1.018026	0.574215
4	6	0	-5.077379	-0.779415	0.273964
5	6	0	-5.413989	0.356486	-0.504771
6	6	0	-4.375066	1.209013	-0.961231
7	6	0	-1.337861	-0.475408	0.478996
8	7	0	-0.360207	0.269728	0.177801
9	6	0	0.968244	-0.182665	0.534270
10	6	0	1.589579	-0.917373	-0.666763
11	8	0	2.921714	-1.280664	-0.354045
12	6	0	3.813615	-0.198319	-0.093997
13	6	0	3.302157	0.552160	1.129214
14	6	0	1.862403	0.993060	0.896348
15	8	0	0.912688	-2.073261	-1.005856
16	6	0	5.184084	-0.813003	0.111839
17	8	0	5.665670	-1.448402	-1.037682
18	8	0	4.092354	1.658690	1.483739
19	8	0	1.362625	1.632431	2.057497
20	8	0	3.870517	3.093491	-0.847942
21	8	0	1.503285	4.349286	-1.202311
22	8	0	-0.370103	3.130124	0.320873
23	8	0	2.345679	-3.608971	0.963549
24	8	0	3.944346	-3.747794	-1.521711
25	1	0	-1.183726	-1.407781	1.035749
26	1	0	1.857717	1.698888	0.052564
27	1	0	2.111567	2.115869	2.434818
28	1	0	4.137031	2.257636	0.711597
29	1	0	3.319430	-0.130022	1.988712
30	1	0	3.835580	0.470508	-0.963218
31	1	0	5.883099	-0.019898	0.379291
32	1	0	5.127199	-1.505215	0.965866
33	1	0	5.157619	-2.262332	-1.192754
34	1	0	0.988887	-2.705768	-0.273067
35	1	0	1.579008	-0.266654	-1.547463
36	1	0	0.941775	-0.891693	1.375963

37	1	0	-2.289224	1.603667	-1.023143
38	1	0	-4.638179	2.071780	-1.561332
39	1	0	-3.456954	-1.886603	1.170717
40	1	0	-0.507434	2.207488	0.032516
41	1	0	0.032720	2.996991	1.191256
42	1	0	2.829843	-2.781807	0.798391
43	1	0	2.466547	-3.844099	1.886785
44	1	0	0.772426	3.942273	-0.683438
45	1	0	1.354036	5.296923	-1.193672
46	1	0	3.057223	3.619044	-1.011939
47	1	0	4.496255	3.301635	-1.544515
48	1	0	3.217370	-3.175330	-1.796315
49	1	0	3.592296	-4.200948	-0.745831
50	6	0	-6.773184	0.600842	-0.806025
51	6	0	-7.749660	-0.244023	-0.356214
52	1	0	-8.787622	-0.047733	-0.592120
53	6	0	-6.108532	-1.636981	0.725268
54	6	0	-7.414124	-1.376331	0.417340
55	1	0	-8.197126	-2.037080	0.765785
56	1	0	-5.844004	-2.504140	1.318749
57	1	0	-7.028056	1.470126	-1.400325

Structure 53.5H_2O (M06-2X/def2-TZVP, DMSO)

Energy (Hartrees): = - 1472.3626133
No imaginary frequencies

Standard orientation:

Center Number	Atomic Number	Atomic Type	Coordinates (Angstroms) X	Y	Z
1	6	0	-3.029643	0.287925	-1.181867
2	6	0	-2.710752	-0.435314	-0.001069
3	6	0	-3.716786	-0.926196	0.791425
4	6	0	-5.076142	-0.702765	0.466515
5	6	0	-5.389357	0.030914	-0.706816
6	6	0	-4.329991	0.510728	-1.522286
7	6	0	-1.316346	-0.659931	0.405206
8	7	0	-0.341759	-0.068470	-0.146833
9	6	0	0.990081	-0.358376	0.335809
10	6	0	1.814538	-0.976467	-0.800537
11	8	0	3.146088	-1.177845	-0.356870
12	6	0	3.857810	0.000078	0.008721
13	6	0	3.128705	0.666851	1.169888
14	6	0	1.681765	0.926303	0.772665
15	8	0	1.335966	-2.203605	-1.221411
16	6	0	5.256816	-0.451711	0.373205
17	8	0	5.915796	-1.069282	-0.706675
18	8	0	3.736475	1.862293	1.595362
19	8	0	0.971427	1.500088	1.860127
20	8	0	3.643538	3.643152	-0.483929
21	8	0	1.108318	4.436561	-1.072030
22	8	0	-0.784702	2.831787	0.089194
23	8	0	2.712392	-3.633702	0.870632
24	8	0	4.460579	-3.514357	-1.440668
25	1	0	-1.165999	-1.362290	1.232193
26	1	0	1.678652	1.623851	-0.078205
27	1	0	1.593828	2.090024	2.311591
28	1	0	3.744496	2.495384	0.844847
29	1	0	3.139696	-0.017895	2.025825
30	1	0	3.896133	0.680957	-0.852945
31	1	0	5.844399	0.417588	0.669331
32	1	0	5.190480	-1.126698	1.237495
33	1	0	5.463082	-1.904989	-0.908939
34	1	0	1.393754	-2.825783	-0.476713
35	1	0	1.810622	-0.309974	-1.669429
36	1	0	0.977645	-1.065374	1.179203
37	1	0	-2.227658	0.642228	-1.817857
38	1	0	-4.576837	1.057483	-2.425050
39	1	0	-3.475426	-1.487208	1.688472
40	1	0	-0.872925	2.003231	-0.408366
41	1	0	-0.341923	2.527205	0.900349
42	1	0	3.056308	-2.730524	0.951246
43	1	0	2.627338	-3.986050	1.764650
44	1	0	0.386807	3.897175	-0.683339
45	1	0	0.869171	5.358316	-0.927092
46	1	0	2.738004	3.950218	-0.707879
47	1	0	4.065470	3.411705	-1.318504
48	1	0	3.740543	-2.932411	-1.719344
49	1	0	4.101400	-3.915768	-0.636181
50	6	0	-6.747221	0.258032	-1.032518
51	6	0	-7.744579	-0.220371	-0.226621
52	1	0	-8.781654	-0.040085	-0.481173
53	6	0	-6.128684	-1.186479	1.281064
54	6	0	-7.432562	-0.950323	0.942052
55	1	0	-8.233364	-1.321869	1.569062

56	1	0	-5.880417	-1.745997	2.175580
57	1	0	-6.982246	0.818726	-1.929863

Structure 53.5H_2O (M06-2X/def2-TZVP, H_2O)

Energy (Hartrees): = - 1472.3797902
No imaginary frequencies

Standard orientation:

Center Number	Atomic Number	Atomic Type	Coordinates (Angstroms) X	Y	Z
1	6	0	-3.094732	1.083187	-0.575783
2	6	0	-2.738901	-0.106606	0.115068
3	6	0	-3.718041	-0.983625	0.510044
4	6	0	-5.083941	-0.719987	0.250726
5	6	0	-5.433532	0.471955	-0.433763
6	6	0	-4.402221	1.360306	-0.839124
7	6	0	-1.344628	-0.443768	0.421411
8	7	0	-0.363158	0.329975	0.191702
9	6	0	0.959794	-0.180955	0.501355
10	6	0	1.597501	-0.758237	-0.767782
11	8	0	2.904876	-1.212153	-0.463375
12	6	0	3.808201	-0.201705	-0.024255
13	6	0	3.271441	0.430573	1.259303
14	6	0	1.850946	0.927037	1.033298
15	8	0	0.894673	-1.849138	-1.271109
16	6	0	5.149177	-0.868017	0.193909
17	8	0	5.722700	-1.346877	-1.009517
18	8	0	4.092071	1.491622	1.706368
19	8	0	1.285073	1.416414	2.236361
20	8	0	4.210332	3.293034	-0.397867
21	8	0	1.809291	3.302006	-1.771700
22	8	0	-0.288360	3.186756	0.046266
23	8	0	1.756405	-3.519943	0.953767
24	8	0	4.019708	-3.603405	-1.524588
25	1	0	-1.190344	-1.426550	0.878646
26	1	0	1.895407	1.732584	0.287696
27	1	0	1.896599	2.071825	2.599232
28	1	0	4.128605	2.166669	0.993688
29	1	0	3.262552	-0.326636	2.050959
30	1	0	3.894537	0.567883	-0.803827
31	1	0	5.837598	-0.137867	0.618340
32	1	0	5.021894	-1.683606	0.915179
33	1	0	5.263638	-2.167686	-1.256022
34	1	0	0.882859	-2.534741	-0.577197
35	1	0	1.642476	-0.000684	-1.556927
36	1	0	0.912332	-0.993731	1.237800
37	1	0	-2.320968	1.764378	-0.901984
38	1	0	-4.676267	2.264404	-1.369945
39	1	0	-3.449179	-1.895666	1.032513
40	1	0	-0.383002	2.207568	0.047642
41	1	0	0.109858	3.387280	0.902923
42	1	0	2.437593	-2.834281	0.875529
43	1	0	1.268394	-3.289980	1.754380
44	1	0	1.056139	3.326383	-1.144765
45	1	0	1.738657	4.109836	-2.293261
46	1	0	3.352987	3.290523	-0.875662
47	1	0	4.823577	2.832635	-0.983321
48	1	0	3.369994	-2.931430	-1.261588
49	1	0	4.019367	-4.241906	-0.801341
50	6	0	-6.797534	0.738126	-0.697387
51	6	0	-7.766528	-0.142071	-0.297922
52	1	0	-8.808561	0.068106	-0.503421
53	6	0	-6.107318	-1.612551	0.652253
54	6	0	-7.418272	-1.329632	0.383505
55	1	0	-8.196901	-2.014989	0.693142
56	1	0	-5.831196	-2.520344	1.175670
57	1	0	-7.059162	1.649486	-1.221966

Structure 53b (M06-2X/def2-TZVP, Gas Phase)

Energy (Hartrees): = - 1090.0985564
No imaginary frequencies

Standard orientation:

Center Number	Atomic Number	Atomic Type	Coordinates (Angstroms) X	Y	Z
1	6	0	-2.160157	1.109203	-0.846684
2	6	0	-1.590379	0.388150	0.375437
3	6	0	-2.347418	-0.917390	0.568641

4	6	0	-3.836129	-0.663121	0.630125
5	6	0	-4.276527	0.074600	-0.629231
6	1	0	-1.963537	0.516917	-1.754210
7	1	0	-2.141037	-1.570280	-0.290221
8	1	0	-4.051458	-0.023773	1.496795
9	1	0	-4.070256	-0.557890	-1.505022
10	1	0	-1.762755	1.046072	1.238177
11	8	0	-3.552597	1.289602	-0.709963
12	6	0	-5.758566	0.418578	-0.621549
13	1	0	-6.004388	0.916311	0.325366
14	1	0	-5.952988	1.123229	-1.430479
15	8	0	-6.564951	-0.711449	-0.844938
16	1	0	-6.291383	-1.397680	-0.223892
17	8	0	-4.551857	-1.879246	0.727363
18	1	0	-4.137774	-2.403820	1.422951
19	8	0	-1.982445	-1.568082	1.769029
20	1	0	-1.043106	-1.778673	1.722905
21	8	0	-1.566536	2.357316	-0.914196
22	1	0	-1.927282	2.828324	-1.672369
23	7	0	-0.191067	0.088067	0.178779
24	6	0	2.112189	0.676982	0.546959
25	6	0	2.625375	-0.342598	-0.215362
26	6	0	2.990790	1.578408	1.194422
27	6	0	4.017928	-0.508018	-0.361239
28	1	0	1.945333	-1.024257	-0.712489
29	6	0	4.343238	1.443201	1.070121
30	1	0	2.574409	2.381833	1.791052
31	6	0	4.895050	0.398823	0.290749
32	1	0	5.013509	2.135234	1.565703
33	6	0	0.659953	0.860789	0.705232
34	1	0	0.349724	1.722079	1.309465
35	6	0	4.568689	-1.554200	-1.144231
36	6	0	6.292806	0.229914	0.138781
37	6	0	6.792815	-0.788016	-0.622016
38	1	0	7.862926	-0.908386	-0.732046
39	6	0	5.920312	-1.691364	-1.271427
40	1	0	6.331945	-2.492425	-1.871536
41	1	0	3.893909	-2.242149	-1.639553
42	1	0	6.958945	0.924084	0.637220

Structure 53b (M06-2X/def2-TZVP, DMSO)

Energy (Hartrees): = -1090.1282963
No imaginary frequencies

Standard orientation:

Center Number	Atomic Number	Atomic Type	Coordinates (Angstroms) X	Y	Z
1	6	0	-2.290064	1.484142	-0.356605
2	6	0	-1.599870	0.372203	0.426507
3	6	0	-2.255204	-0.961407	0.066135
4	6	0	-3.752660	-0.881740	0.274830
5	6	0	-4.321599	0.287631	-0.521276
6	1	0	-2.133151	1.336112	-1.434858
7	1	0	-2.059765	-1.176313	-0.991947
8	1	0	-3.954739	-0.706540	1.339533
9	1	0	-4.137671	0.117338	-1.591799
10	1	0	-1.753719	0.567983	1.496487
11	8	0	-3.681164	1.475485	-0.092679
12	6	0	-5.813819	0.475203	-0.300440
13	1	0	-6.015067	0.522980	0.777086
14	1	0	-6.115044	1.425335	-0.743738
15	8	0	-6.577278	-0.538852	-0.921587
16	1	0	-6.191010	-1.385069	-0.660675
17	8	0	-4.387250	-2.076183	-0.152660
18	1	0	-3.939832	-2.816358	0.278007
19	8	0	-1.778687	-2.017928	0.877344
20	1	0	-0.860789	-2.199871	0.639202
21	8	0	-1.780003	2.697897	0.067957
22	1	0	-2.112418	3.392475	-0.514861
23	7	0	-0.200044	0.304724	0.077397
24	6	0	2.095028	0.184029	0.813831
25	6	0	2.645732	0.052924	-0.437660
26	6	0	2.936914	0.203828	1.953974
27	6	0	4.042518	-0.065970	-0.603927
28	1	0	2.005825	0.036339	-1.312458
29	6	0	4.291382	0.091280	1.822799
30	1	0	2.487602	0.309858	2.934948
31	6	0	4.881356	-0.048611	0.542894
32	1	0	4.933693	0.105627	2.695593
33	6	0	0.640609	0.298213	1.023647
34	1	0	0.320643	0.377559	2.069068
35	6	0	4.632174	-0.207403	-1.887077

36	6	0	6.282515	-0.173477	0.372120
37	6	0	6.820971	-0.308828	-0.876841
38	1	0	7.892830	-0.404195	-0.999416
39	6	0	5.986459	-0.325984	-2.019672
40	1	0	6.429828	-0.435141	-3.001647
41	1	0	3.986463	-0.220111	-2.757577
42	1	0	6.917023	-0.160632	1.250831

Structure 54 (M06-2X/def2-TZVP, Gas Phase)

Energy (Hartrees): = - 1090.1011475
No imaginary frequencies

Standard orientation:

Center Number	Atomic Number	Atomic Type	Coordinates (Angstroms) X	Y	Z
1	6	0	-2.384612	1.325783	0.880145
2	6	0	-1.613800	0.036269	0.545351
3	6	0	-2.351623	-0.719660	-0.557586
4	6	0	-3.790999	-0.937872	-0.143144
5	6	0	-4.448882	0.404742	0.152267
6	1	0	-2.340140	-0.127503	-1.478907
7	1	0	-3.803483	-1.541698	0.775068
8	1	0	-4.452526	1.016120	-0.755623
9	1	0	-1.591445	-0.599144	1.440226
10	8	0	-3.714113	1.049516	1.184431
11	6	0	-5.876721	0.257877	0.655282
12	1	0	-5.891885	-0.457917	1.487962
13	1	0	-6.205048	1.224050	1.038496
14	8	0	-6.767342	-0.113466	-0.368511
15	1	0	-6.392913	-0.876316	-0.824961
16	8	0	-4.513610	-1.591520	-1.167678
17	1	0	-3.996307	-2.357693	-1.442761
18	8	0	-1.785250	-1.998256	-0.784921
19	1	0	-0.946422	-1.890968	-1.244230
20	7	0	-0.288921	0.380061	0.084192
21	6	0	2.090181	0.028466	0.180034
22	6	0	2.393488	0.961867	-0.845230
23	6	0	3.103328	-0.648890	0.807220
24	6	0	3.686461	1.186929	-1.205659
25	1	0	1.577375	1.482077	-1.328312
26	6	0	4.456343	-0.432601	0.455993
27	1	0	2.874212	-1.363752	1.591109
28	6	0	4.754428	0.501062	-0.568114
29	1	0	3.921401	1.898193	-1.988642
30	6	0	0.702569	-0.225954	0.586604
31	1	0	0.565858	-0.983905	1.368214
32	1	0	-1.971294	1.779322	1.786279
33	8	0	-2.325737	2.224882	-0.193272
34	1	0	-1.443832	2.147284	-0.580873
35	6	0	5.516754	-1.117283	1.095703
36	6	0	6.105054	0.720430	-0.922014
37	6	0	7.110245	0.044249	-0.287677
38	6	0	6.813516	-0.884449	0.732644
39	1	0	7.619462	-1.410667	1.227368
40	1	0	5.282546	-1.828381	1.879049
41	1	0	8.141455	0.220460	-0.565149
42	1	0	6.329471	1.434969	-1.704962

Structure 54 (M06-2X/def2-TZVP, DMSO)

Energy (Hartrees): = - 1090.130096
No imaginary frequencies

Standard orientation:

Center Number	Atomic Number	Atomic Type	Coordinates (Angstroms) X	Y	Z
1	6	0	-2.379747	1.319225	0.899263
2	6	0	-1.617552	0.024979	0.574488
3	6	0	-2.344467	-0.743845	-0.528816
4	6	0	-3.792483	-0.939936	-0.136304
5	6	0	-4.439976	0.413884	0.126706
6	1	0	-2.307255	-0.172822	-1.463668
7	1	0	-3.833111	-1.533604	0.786641
8	1	0	-4.414169	1.011879	-0.790581
9	1	0	-1.594931	-0.599929	1.475060
10	8	0	-3.726950	1.069800	1.166765
11	6	0	-5.879321	0.292137	0.599635
12	1	0	-5.921854	-0.396614	1.452843
13	1	0	-6.219051	1.271525	0.939363

14	8	0	-6.749653	-0.117999	-0.435518
15	1	0	-6.350837	-0.893047	-0.852067
16	8	0	-4.511634	-1.593706	-1.169169
17	1	0	-4.016298	-2.386860	-1.412566
18	8	0	-1.787914	-2.031883	-0.720463
19	1	0	-0.939985	-1.940845	-1.172718
20	7	0	-0.292953	0.373740	0.112439
21	6	0	2.084615	0.033371	0.197434
22	6	0	2.380469	0.979343	-0.820325
23	6	0	3.101731	-0.653349	0.809388
24	6	0	3.670923	1.208606	-1.191346
25	1	0	1.565314	1.510059	-1.295394
26	6	0	4.451951	-0.434323	0.444839
27	1	0	2.878446	-1.377124	1.586691
28	6	0	4.743075	0.511750	-0.571157
29	1	0	3.900883	1.928804	-1.968236
30	6	0	0.701028	-0.229963	0.615455
31	1	0	0.573659	-0.985549	1.398235
32	1	0	-1.984065	1.755344	1.820811
33	8	0	-2.276432	2.237939	-0.157819
34	1	0	-1.417302	2.085533	-0.578934
35	6	0	5.516834	-1.130490	1.067032
36	6	0	6.091327	0.732423	-0.937944
37	6	0	7.101064	0.044793	-0.320689
38	6	0	6.811384	-0.895663	0.692711
39	1	0	7.621894	-1.429414	1.173066
40	1	0	5.286191	-1.850502	1.843826
41	1	0	8.130883	0.220462	-0.606179
42	1	0	6.307785	1.455654	-1.715852

Structure 54.5H_2O (M06-2X/def2-TZVP, Gas Phase)

Energy (Hartrees): = - 1472.3230037
No imaginary frequencies

Standard orientation:

Center Number	Atomic Number	Atomic Type	Coordinates (Angstroms) X	Y	Z
1	6	0	3.879023	-0.039298	-1.112910
2	6	0	2.778309	-0.593921	-0.513765
3	6	0	2.924040	-1.310251	0.703914
4	6	0	4.150805	-1.442843	1.279472
5	6	0	5.305774	-0.875368	0.678696
6	6	0	5.166149	-0.163150	-0.538869
7	6	0	1.455827	-0.399732	-1.122139
8	7	0	0.390868	-0.815508	-0.580191
9	6	0	-0.877581	-0.468801	-1.186647
10	6	0	-1.393390	0.802981	-0.511491
11	6	0	-2.850473	1.086242	-0.855738
12	6	0	-3.676977	-0.163882	-0.582976
13	8	0	-3.151045	-1.261025	-1.323398
14	6	0	-1.855782	-1.631504	-0.991067
15	8	0	-0.513460	1.838847	-0.908801
16	8	0	-1.827359	-2.082812	0.342232
17	6	0	-5.120860	-0.020218	-1.010940
18	8	0	-5.930107	-1.059787	-0.503153
19	8	0	-3.368840	2.110827	-0.031442
20	1	0	1.435095	0.161579	-2.062785
21	1	0	-0.786086	-0.265750	-2.260453
22	1	0	-0.883389	2.717966	-0.702756
23	1	0	-1.340989	0.660009	0.576578
24	1	0	-3.037009	2.975315	-0.314686
25	1	0	-2.929809	1.352813	-1.918326
26	1	0	-5.420416	-1.892248	-0.479802
27	1	0	-5.512866	0.921915	-0.628811
28	1	0	-5.158163	-0.001600	-2.106487
29	1	0	-3.641708	-0.382762	0.491346
30	1	0	-0.916445	-1.992816	0.655559
31	1	0	-1.603724	-2.451362	-1.669133
32	1	0	3.769701	0.512147	-2.041056
33	1	0	4.265074	-1.989967	2.207785
34	1	0	2.043621	-1.749090	1.155183
35	1	0	-4.842976	-2.720608	1.513277
36	8	0	-4.533447	-3.174622	0.714827
37	1	0	-3.583196	-3.003379	0.674391
38	1	0	-1.485126	4.041524	0.846126
39	8	0	-1.615059	4.270782	-0.105931
40	1	0	-1.342484	5.178677	-0.251988
41	8	0	-1.416052	3.007269	2.209883
42	1	0	-0.594166	2.483655	2.184448
43	1	0	-2.142673	2.399386	2.022831
44	1	0	0.724487	1.738239	0.538360
45	8	0	0.977322	1.753921	1.477516
46	1	0	1.489535	0.953805	1.629109
47	8	0	-5.719343	-1.110514	2.250360

48	1	0	-6.017251	-0.955864	1.334441
49	1	0	-6.485815	-1.002835	2.816971
50	6	0	6.313413	0.405543	-1.140531
51	6	0	6.589446	-0.999145	1.256769
52	6	0	7.542424	0.270520	-0.558302
53	6	0	7.681689	-0.439989	0.652926
54	1	0	6.200008	0.949348	-2.070757
55	1	0	8.415478	0.708566	-1.024033
56	1	0	8.660730	-0.540466	1.103285
57	1	0	6.692591	-1.543844	2.187577

Structure 54.5H_2O (M06-2X/def2-TZVP, DMSO)

Energy (Hartrees): = - 1472.3636238
No imaginary frequencies

Standard orientation:

Center Number	Atomic Number	Atomic Type	Coordinates (Angstroms) X	Y	Z
1	6	0	3.885267	-0.365306	-1.157915
2	6	0	2.779025	-0.557096	-0.370595
3	6	0	2.934279	-0.840408	1.013267
4	6	0	4.178201	-0.919252	1.563172
5	6	0	5.340014	-0.723194	0.768955
6	6	0	5.189559	-0.442157	-0.613247
7	6	0	1.447927	-0.449511	-0.981469
8	7	0	0.379454	-0.562078	-0.309851
9	6	0	-0.885871	-0.419278	-0.999187
10	6	0	-1.559591	0.878677	-0.557116
11	6	0	-3.001620	0.942008	-1.052363
12	6	0	-3.726296	-0.327105	-0.621480
13	8	0	-3.053274	-1.480860	-1.117191
14	6	0	-1.749131	-1.637906	-0.662557
15	8	0	-0.755210	1.928190	-1.076252
16	8	0	-1.775734	-1.872193	0.729800
17	6	0	-5.136589	-0.414894	-1.154380
18	8	0	-5.834756	-1.514972	-0.587047
19	8	0	-3.721049	2.014880	-0.484136
20	1	0	1.428600	-0.251967	-2.058205
21	1	0	-0.762267	-0.384686	-2.088039
22	1	0	-1.159781	2.792457	-0.869564
23	1	0	-1.578852	0.925395	0.540678
24	1	0	-3.214177	2.840506	-0.525760
25	1	0	-2.995843	1.005736	-2.149379
26	1	0	-5.202680	-2.234630	-0.413463
27	1	0	-5.684356	0.491293	-0.899036
28	1	0	-5.100941	-0.509094	-2.245099
29	1	0	-3.753976	-0.354610	0.474743
30	1	0	-0.914790	-1.608531	1.088624
31	1	0	-1.358755	-2.517803	-1.179831
32	1	0	3.767401	-0.148501	-2.214716
33	1	0	4.301918	-1.136696	2.617835
34	1	0	2.051259	-1.000351	1.620791
35	1	0	-4.817373	-2.674268	1.753575
36	8	0	-4.306775	-3.242109	1.154362
37	1	0	-3.422170	-2.849693	1.112474
38	1	0	-1.579025	4.268872	0.682277
39	8	0	-1.994833	4.230821	-0.211858
40	1	0	-1.930582	5.106157	-0.610259
41	8	0	-0.660721	4.000177	2.094291
42	1	0	0.016405	3.330814	1.863090
43	1	0	-1.111281	3.677273	2.882543
44	1	0	0.628405	2.026884	0.194930
45	8	0	1.116509	2.200437	1.021647
46	1	0	1.299055	1.324131	1.383568
47	8	0	-6.093791	-1.273747	2.168256
48	1	0	-6.229123	-1.232915	1.203336
49	1	0	-6.959530	-1.430785	2.560080
50	6	0	6.344242	-0.245645	-1.408752
51	6	0	6.642057	-0.802080	1.315689
52	6	0	7.591388	-0.326648	-0.852567
53	6	0	7.741266	-0.608579	0.523310
54	1	0	6.219695	-0.030413	-2.463755
55	1	0	8.471273	-0.174467	-1.465159
56	1	0	8.734962	-0.671862	0.949130
57	1	0	6.752664	-1.018465	2.371982

Structure 54.5H_2O (M06-2X/def2-TZVP, H_2O)

Energy (Hartrees): = - 1472.3778507
No imaginary frequencies

Standard orientation:

Center Number	Atomic Number	Atomic Type	Coordinates (Angstroms) X	Y	Z
1	6	0	3.903959	-0.090861	-1.125833
2	6	0	2.812254	-0.524068	-0.417471
3	6	0	2.985911	-1.090050	0.874704
4	6	0	4.232233	-1.206334	1.412298
5	6	0	5.379166	-0.769653	0.696166
6	6	0	5.210594	-0.204396	-0.594000
7	6	0	1.479842	-0.363040	-1.012419
8	7	0	0.422064	-0.800223	-0.464330
9	6	0	-0.844355	-0.481374	-1.094807
10	6	0	-1.411011	0.773211	-0.434346
11	6	0	-2.855697	1.023025	-0.842195
12	6	0	-3.688990	-0.235704	-0.639253
13	8	0	-3.095890	-1.342746	-1.315600
14	6	0	-1.793496	-1.664845	-0.915858
15	8	0	-0.562543	1.846850	-0.809475
16	8	0	-1.821352	-2.091591	0.428703
17	6	0	-5.075004	-0.083348	-1.224132
18	8	0	-5.909169	-1.190699	-0.908247
19	8	0	-3.434598	2.035474	-0.030904
20	1	0	1.448655	0.166721	-1.969531
21	1	0	-0.738839	-0.288700	-2.168087
22	1	0	-0.958040	2.705857	-0.558314
23	1	0	-1.387658	0.637212	0.655215
24	1	0	-3.146420	2.905507	-0.344965
25	1	0	-2.886811	1.313574	-1.898582
26	1	0	-5.445148	-2.003275	-1.151622
27	1	0	-5.554665	0.800169	-0.806986
28	1	0	-4.994691	0.035730	-2.308906
29	1	0	-3.762386	-0.452045	0.433151
30	1	0	-0.943839	-1.933267	0.809380
31	1	0	-1.489573	-2.490483	-1.561903
32	1	0	3.772493	0.348183	-2.109347
33	1	0	4.369456	-1.633445	2.398858
34	1	0	2.113905	-1.419518	1.425141
35	1	0	-4.827750	-2.442849	1.621483
36	8	0	-4.278135	-3.201453	1.362213
37	1	0	-3.435761	-2.820878	1.067431
38	1	0	-1.611994	4.152653	0.814958
39	8	0	-1.719698	4.316658	-0.139827
40	1	0	-1.220299	5.115237	-0.345150
41	8	0	-1.655322	2.899378	2.268622
42	1	0	-0.794131	2.473854	2.103832
43	1	0	-2.292139	2.401256	1.732896
44	1	0	0.649186	1.859823	0.637983
45	8	0	0.976503	1.950641	1.551808
46	1	0	1.300115	1.073101	1.790056
47	8	0	-6.085777	-1.023702	1.866411
48	1	0	-6.173615	-1.070865	0.894988
49	1	0	-6.914106	-1.370405	2.217461
50	6	0	6.349990	0.234325	-1.311157
51	6	0	6.683078	-0.878095	1.233159
52	6	0	7.599442	0.117142	-0.766929
53	6	0	7.767042	-0.443989	0.518857
54	1	0	6.211385	0.664343	-2.296236
55	1	0	8.467688	0.453311	-1.319328
56	1	0	8.762008	-0.531401	0.936717
57	1	0	6.806623	-1.309989	2.219382

Structure 54b (M06-2X/def2-TZVP, Gas Phase)

Energy (Hartrees): = - 1090.099592
No imaginary frequencies

Standard orientation:

Center Number	Atomic Number	Atomic Type	Coordinates (Angstroms) X	Y	Z
1	6	0	2.264825	1.068938	-1.209384
2	6	0	1.590965	-0.210426	-0.686010
3	6	0	2.256108	-0.634094	0.624155
4	6	0	3.750760	-0.750701	0.414444
5	6	0	4.302298	0.576131	-0.092802
6	1	0	2.071291	0.124602	1.391781
7	1	0	3.939021	-1.520936	-0.346584
8	1	0	4.130521	1.352923	0.659201
9	1	0	1.743733	-1.012122	-1.419860
10	8	0	3.643351	0.904070	-1.308761
11	6	0	5.789944	0.510820	-0.402583
12	1	0	5.985358	-0.355978	-1.047797
13	1	0	6.062059	1.408834	-0.957174

14	8	0	6.576660	0.480902	0.763327
15	1	0	6.229702	-0.212651	1.336911
16	8	0	4.401628	-1.088407	1.623467
17	1	0	3.941306	-1.848745	1.997326
18	8	0	1.794612	-1.901119	1.057958
19	1	0	0.908858	-1.804722	1.421552
20	7	0	0.190507	0.048565	-0.443813
21	6	0	-2.100505	-0.701521	-0.573384
22	6	0	-2.941174	-1.770799	-0.967045
23	6	0	-2.647506	0.414978	0.008787
24	6	0	-4.289755	-1.704952	-0.768772
25	1	0	-2.499045	-2.646740	-1.427468
26	6	0	-4.037802	0.513415	0.221687
27	1	0	-1.999839	1.230933	0.306441
28	6	0	-4.875782	-0.564286	-0.170581
29	1	0	-4.930249	-2.525580	-1.068348
30	6	0	-0.652276	-0.825280	-0.801775
31	1	0	-0.328446	-1.743002	-1.309680
32	1	0	1.927326	1.276246	-2.229396
33	8	0	1.980192	2.151577	-0.366153
34	1	0	1.067940	2.043201	-0.066405
35	6	0	-4.624124	1.659120	0.816927
36	6	0	-6.270948	-0.462819	0.047656
37	6	0	-6.805788	0.653406	0.624427
38	6	0	-5.972856	1.727799	1.013090
39	1	0	-6.412244	2.606265	1.467245
40	1	0	-3.980558	2.479054	1.112137
41	1	0	-7.873683	0.721516	0.786781
42	1	0	-6.906429	-1.287615	-0.251687

Structure 54b (M06-2X/def2-TZVP, DMSO)

Energy (Hartrees): = - 1090.128578
No imaginary frequencies

Standard orientation:

Center Number	Atomic Number	Atomic Type	Coordinates (Angstroms) X	Y	Z
1	6	0	2.240359	1.058952	-1.210794
2	6	0	1.594473	-0.238927	-0.703113
3	6	0	2.267076	-0.684374	0.597465
4	6	0	3.765326	-0.751120	0.395229
5	6	0	4.280310	0.602448	-0.076750
6	1	0	2.053002	0.041386	1.389826
7	1	0	3.987792	-1.500731	-0.375720
8	1	0	4.074725	1.355383	0.691979
9	1	0	1.745053	-1.020830	-1.456650
10	8	0	3.629203	0.945024	-1.292898
11	6	0	5.771508	0.589803	-0.371722
12	1	0	5.998771	-0.243578	-1.048536
13	1	0	6.034238	1.518482	-0.880142
14	8	0	6.548525	0.522360	0.806741
15	1	0	6.190712	-0.198336	1.341287
16	8	0	4.423552	-1.085810	1.606329
17	1	0	4.005165	-1.883176	1.956297
18	8	0	1.840635	-1.976306	0.989733
19	1	0	0.946187	-1.914771	1.347507
20	7	0	0.194167	0.012501	-0.444622
21	6	0	-2.099146	-0.713730	-0.575209
22	6	0	-2.946018	-1.790704	-0.937121
23	6	0	-2.636348	0.418225	-0.012569
24	6	0	-4.293062	-1.718841	-0.724056
25	1	0	-2.507279	-2.675986	-1.383155
26	6	0	-4.025534	0.523415	0.212677
27	1	0	-1.991659	1.245671	0.261305
28	6	0	-4.869872	-0.562519	-0.144494
29	1	0	-4.939238	-2.544938	-0.997006
30	6	0	-0.653145	-0.850955	-0.819142
31	1	0	-0.343956	-1.757894	-1.350513
32	1	0	1.910056	1.255300	-2.234850
33	8	0	1.909306	2.139114	-0.376912
34	1	0	1.023725	1.959585	-0.027479
35	6	0	-4.602544	1.685528	0.787268
36	6	0	-6.263539	-0.454712	0.087669
37	6	0	-6.789793	0.676928	0.645010
38	6	0	-5.950214	1.760060	0.997544
39	1	0	-6.383969	2.649984	1.436224
40	1	0	-3.953230	2.510959	1.055445
41	1	0	-7.855926	0.751122	0.820103
42	1	0	-6.902129	-1.287042	-0.184880

Structure 55 (M06-2X/def2-TZVP, Gas Phase)

Energy (Hartrees): = - 1243.732541
No imaginary frequencies

Standard orientation:

Center Number	Atomic Number	Atomic Type	Coordinates (Angstroms) X	Y	Z
1	6	0	-2.721388	-0.772240	-1.220856
2	6	0	-2.098852	0.217677	-0.238119
3	6	0	-2.923675	0.215671	1.042701
4	6	0	-4.380563	0.477476	0.731600
5	6	0	-4.878571	-0.550730	-0.278528
6	1	0	-2.654816	-1.793748	-0.813917
7	1	0	-2.839227	-0.773067	1.512703
8	1	0	-4.471778	1.474774	0.280590
9	1	0	-4.800929	-1.554131	0.165158
10	1	0	-2.155987	1.209571	-0.708069
11	8	0	-4.078283	-0.456076	-1.442864
12	6	0	-6.324386	-0.316685	-0.690041
13	1	0	-6.447120	0.733913	-0.983286
14	1	0	-6.539884	-0.934850	-1.561918
15	8	0	-7.228520	-0.687459	0.321100
16	1	0	-6.951311	-0.256042	1.138524
17	8	0	-5.169965	0.393301	1.901890
18	1	0	-4.738971	0.925973	2.580788
19	8	0	-2.502259	1.219107	1.944484
20	1	0	-1.599814	1.020849	2.218033
21	8	0	-2.046709	-0.656943	-2.423031
22	1	0	-2.468175	-1.235685	-3.067000
23	7	0	-0.746169	-0.169906	0.082338
24	6	0	1.614408	0.194609	-0.159605
25	6	0	2.643953	1.210573	-0.196667
26	6	0	1.948901	-1.104712	0.029934
27	6	0	4.000850	0.830478	-0.066560
28	6	0	3.303953	-1.523797	0.181329
29	1	0	1.162432	-1.849753	0.049607
30	6	0	4.341978	-0.570032	0.122676
31	6	0	0.195707	0.541701	-0.372551
32	1	0	-0.020133	1.427210	-0.978043
33	6	0	3.608494	-2.885388	0.373741
34	6	0	4.907365	-3.302455	0.508159
35	6	0	5.943397	-2.360208	0.447973
36	6	0	5.666220	-1.027711	0.259245
37	1	0	5.133852	-4.349785	0.658506
38	1	0	6.971077	-2.683817	0.550853
39	1	0	6.490602	-0.331012	0.217104
40	1	0	2.792969	-3.597406	0.414961
41	6	0	2.329414	2.578159	-0.329440
42	6	0	4.984974	1.835896	-0.108772
43	6	0	4.653993	3.159843	-0.257975
44	1	0	5.432561	3.910952	-0.287308
45	6	0	3.311086	3.536518	-0.362024
46	1	0	3.045523	4.580549	-0.464319
47	1	0	1.296312	2.889449	-0.392396
48	1	0	6.028349	1.572088	-0.016878

Structure 55 (M06-2X/def2-TZVP, DMSO)

Energy (Hartrees): = - 1243.7658215
No imaginary frequencies

Standard orientation:

Center Number	Atomic Number	Atomic Type	Coordinates (Angstroms) X	Y	Z
1	6	0	2.818244	-1.347219	0.836489
2	6	0	2.101020	-0.063553	0.430652
3	6	0	2.822018	0.538984	-0.773396
4	6	0	4.293692	0.715160	-0.467486
5	6	0	4.892403	-0.617952	-0.032061
6	1	0	2.754219	-2.085213	0.023616
7	1	0	2.720368	-0.146741	-1.623944
8	1	0	4.402336	1.431404	0.357408
9	1	0	4.803788	-1.337955	-0.857995
10	1	0	2.160729	0.637961	1.273801
11	8	0	4.182532	-1.079844	1.103639
12	6	0	6.354095	-0.500888	0.369215
13	1	0	6.456809	0.290662	1.121883
14	1	0	6.668018	-1.441631	0.823372
15	8	0	7.196933	-0.268002	-0.740734
16	1	0	6.808455	0.458814	-1.245340
17	8	0	4.993148	1.177492	-1.611627

18	1	0	4.533761	1.962029	-1.938204
19	8	0	2.312602	1.815702	-1.108677
20	1	0	1.418365	1.710255	-1.457725
21	8	0	2.236940	-1.822318	1.998151
22	1	0	2.597034	-2.698561	2.185656
23	7	0	0.738122	-0.354601	0.048684
24	6	0	-1.609530	0.079151	0.290468
25	6	0	-2.564262	1.165497	0.342475
26	6	0	-2.023802	-1.177416	-0.002190
27	6	0	-3.935703	0.899894	0.111361
28	6	0	-3.396477	-1.481462	-0.254399
29	1	0	-1.303328	-1.987047	-0.026080
30	6	0	-4.365861	-0.457564	-0.188213
31	6	0	-0.183828	0.316198	0.598787
32	1	0	0.046834	1.078647	1.348380
33	6	0	-3.784602	-2.802585	-0.553834
34	6	0	-5.101369	-3.110264	-0.786198
35	6	0	-6.069949	-2.097692	-0.719591
36	6	0	-5.710801	-0.802845	-0.426342
37	1	0	-5.393517	-4.126495	-1.019071
38	1	0	-7.110774	-2.335796	-0.900548
39	1	0	-6.484976	-0.049756	-0.380993
40	1	0	-3.020201	-3.569921	-0.598739
41	6	0	-2.154311	2.492933	0.586450
42	6	0	-4.845523	1.974092	0.163233
43	6	0	-4.424217	3.256355	0.421296
44	1	0	-5.144737	4.063929	0.455468
45	6	0	-3.065017	3.520304	0.628750
46	1	0	-2.730738	4.532817	0.817177
47	1	0	-1.105418	2.716001	0.730054
48	1	0	-5.898446	1.799784	-0.007777

Structure 55.5H_2O (M06-2X/def2-TZVP, Gas Phase)

Energy (Hartrees): = - 1625.9530683
No imaginary frequencies

Standard orientation:

Center Number	Atomic Number	Atomic Type	Coordinates (Angstroms) X	Y	Z
1	6	0	-2.832391	0.954697	0.459903
2	6	0	-2.282632	-0.276609	0.342225
3	6	0	-3.100425	-1.412702	-0.013138
4	6	0	-4.492573	-1.242886	-0.184312
5	6	0	-5.077218	0.079773	-0.029262
6	6	0	-4.233048	1.169866	0.271184
7	6	0	-0.854411	-0.476881	0.648664
8	7	0	0.050282	0.317402	0.262243
9	6	0	1.418248	0.004612	0.633263
10	6	0	2.031128	-0.926693	-0.426735
11	8	0	3.397756	-1.141187	-0.129355
12	6	0	4.226054	0.020726	-0.146623
13	6	0	3.725871	0.971425	0.934055
14	6	0	2.251100	1.274132	0.700987
15	8	0	1.420774	-2.168601	-0.478908
16	6	0	5.644106	-0.461363	0.086977
17	8	0	6.107725	-1.284814	-0.944495
18	8	0	4.455687	2.169175	1.016689
19	8	0	1.761729	2.110530	1.735636
20	8	0	4.008791	3.100545	-1.522638
21	8	0	1.631914	4.362643	-1.753184
22	8	0	-0.157434	3.173987	-0.094329
23	8	0	3.049712	-3.215748	1.624262
24	8	0	4.517594	-3.722346	-0.893599
25	1	0	-0.607738	-1.351886	1.259574
26	1	0	2.161577	1.790861	-0.266102
27	1	0	2.491211	2.706165	1.960480
28	1	0	4.426912	2.601946	0.139446
29	1	0	3.828812	0.477237	1.908544
30	1	0	4.156854	0.500616	-1.130765
31	1	0	6.299514	0.408032	0.146996
32	1	0	5.680253	-0.970388	1.062447
33	1	0	5.643653	-2.138006	-0.910658
34	1	0	1.619974	-2.644589	0.344882
35	1	0	1.930994	-0.476004	-1.419896
36	1	0	1.468119	-0.517151	1.600153
37	1	0	-2.214494	1.800558	0.738667
38	1	0	-0.284535	2.237035	-0.328849
39	1	0	0.336870	3.095522	0.737372
40	1	0	3.486140	-2.407833	1.305422
41	1	0	3.188277	-3.273907	2.572923
42	1	0	0.921231	3.962452	-1.203648
43	1	0	1.476773	5.309219	-1.762198
44	1	0	3.188329	3.622015	-1.662428
45	1	0	4.574832	3.238705	-2.284620

46	1	0	3.730730	-3.247498	-1.188711
47	1	0	4.261838	-4.069563	-0.030670
48	6	0	-2.524757	-2.680117	-0.231375
49	6	0	-5.262602	-2.370334	-0.525835
50	6	0	-4.685563	-3.603868	-0.705862
51	1	0	-5.301968	-4.452881	-0.971351
52	6	0	-3.302071	-3.760243	-0.567528
53	1	0	-2.844625	-4.726417	-0.735866
54	1	0	-1.450105	-2.797839	-0.166384
55	1	0	-6.330031	-2.273272	-0.661694
56	6	0	-4.772946	2.462986	0.407423
57	6	0	-6.453983	0.332638	-0.174431
58	6	0	-6.118094	2.680933	0.254787
59	6	0	-6.963191	1.601584	-0.036030
60	1	0	-4.101116	3.282520	0.633407
61	1	0	-6.527047	3.677263	0.359569
62	1	0	-7.134514	-0.475855	-0.398567
63	1	0	-8.026292	1.767608	-0.153834

Structure 55.5H_2O (M06-2X/def2-TZVP, DMSO)

Energy (Hartrees): = - 1625.9968617
No imaginary frequencies

Standard orientation:

Center Number	Atomic Number	Atomic Type	Coordinates (Angstroms) X	Y	Z
1	6	0	-2.779569	0.976744	0.301824
2	6	0	-2.296840	-0.289106	0.316253
3	6	0	-3.192895	-1.409241	0.120232
4	6	0	-4.575066	-1.171841	-0.073364
5	6	0	-5.078506	0.193580	-0.079559
6	6	0	-4.166606	1.255098	0.101734
7	6	0	-0.865698	-0.522894	0.593896
8	7	0	0.047850	0.249586	0.176056
9	6	0	1.412789	-0.098009	0.529215
10	6	0	2.064237	-0.858967	-0.636776
11	8	0	3.411431	-1.146222	-0.303528
12	6	0	4.252944	-0.016694	-0.087932
13	6	0	3.693380	0.796222	1.075304
14	6	0	2.240423	1.150459	0.787159
15	8	0	1.450607	-2.066190	-0.916363
16	6	0	5.633020	-0.572134	0.196302
17	8	0	6.147037	-1.312697	-0.885379
18	8	0	4.432798	1.964717	1.337945
19	8	0	1.685142	1.879137	1.870042
20	8	0	4.300638	3.342547	-1.024413
21	8	0	1.847652	4.371971	-1.629226
22	8	0	-0.026218	3.128355	-0.064153
23	8	0	2.889570	-3.470928	1.140062
24	8	0	4.427610	-3.654157	-1.312546
25	1	0	-0.617424	-1.391896	1.209170
26	1	0	2.218884	1.768903	-0.122551
27	1	0	2.395457	2.443002	2.210940
28	1	0	4.444940	2.510349	0.522989
29	1	0	3.732152	0.183937	1.983587
30	1	0	4.275012	0.599182	-0.996473
31	1	0	6.316662	0.256014	0.385248
32	1	0	5.582444	-1.182500	1.108311
33	1	0	5.614928	-2.118824	-0.990155
34	1	0	1.554326	-2.646735	-0.142631
35	1	0	2.026317	-0.254122	-1.548898
36	1	0	1.444824	-0.748983	1.414139
37	1	0	-2.116149	1.814293	0.480977
38	1	0	-0.142482	2.190426	-0.309212
39	1	0	0.406440	3.043001	0.800534
40	1	0	3.330051	-2.607200	1.141922
41	1	0	2.827884	-3.760131	2.058426
42	1	0	1.131795	3.954145	-1.104190
43	1	0	1.699657	5.322519	-1.578656
44	1	0	3.447970	3.767855	-1.259270
45	1	0	5.001136	3.899110	-1.380294
46	1	0	3.736910	-3.011973	-1.525909
47	1	0	4.129545	-4.001963	-0.459966
48	6	0	-2.713493	-2.734919	0.090516
49	6	0	-5.424501	-2.278129	-0.267549
50	6	0	-4.935760	-3.562589	-0.277354
51	1	0	-5.611808	-4.394401	-0.429987
52	6	0	-3.566466	-3.794318	-0.101013
53	1	0	-3.179141	-4.805093	-0.121038
54	1	0	-1.655662	-2.928263	0.208670
55	1	0	-6.483551	-2.125368	-0.418193
56	6	0	-4.623420	2.587882	0.100679
57	6	0	-6.439060	0.512924	-0.258592
58	6	0	-5.953999	2.871242	-0.078368

59	6	0	-6.865759	1.820597	-0.258626
60	1	0	-3.900580	3.382964	0.243785
61	1	0	-6.300974	3.896755	-0.079642
62	1	0	-7.172519	-0.268963	-0.398431
63	1	0	-7.917063	2.039157	-0.399003

Structure 55.5H_2O (M06-2X/def2-TZVP, H2O)

Energy (Hartrees): = - 1626.0152944
No imaginary frequencies

Standard orientation:

Center Number	Atomic Number	Atomic Type	Coordinates (Angstroms) X	Y	Z
1	6	0	-2.712342	0.981493	0.272523
2	6	0	-2.327911	-0.317524	0.313982
3	6	0	-3.305485	-1.372180	0.162794
4	6	0	-4.670791	-1.038874	-0.000392
5	6	0	-5.072126	0.359406	-0.020979
6	6	0	-4.080599	1.356433	0.102662
7	6	0	-0.914580	-0.640095	0.584703
8	7	0	0.031680	0.050047	0.101255
9	6	0	1.387167	-0.274297	0.493570
10	6	0	2.170026	-0.737916	-0.738012
11	8	0	3.523854	-0.958754	-0.380060
12	6	0	4.217172	0.189808	0.105816
13	6	0	3.522690	0.696341	1.367642
14	6	0	2.054240	0.962495	1.071143
15	8	0	1.681245	-1.934466	-1.256388
16	6	0	5.645008	-0.232199	0.375255
17	8	0	6.355601	-0.545744	-0.809450
18	8	0	4.137985	1.861054	1.879051
19	8	0	1.356803	1.329470	2.247964
20	8	0	4.031905	3.764209	-0.114022
21	8	0	2.546049	2.738111	-2.174305
22	8	0	-0.104398	2.189281	-1.701222
23	8	0	2.685406	-3.455474	0.974451
24	8	0	5.124628	-3.071385	-1.391358
25	1	0	-0.707009	-1.481083	1.251341
26	1	0	1.993224	1.772840	0.329802
27	1	0	1.841329	2.061032	2.653881
28	1	0	4.095108	2.559569	1.188990
29	1	0	3.594065	-0.073158	2.143737
30	1	0	4.197763	0.975083	-0.662434
31	1	0	6.166907	0.590206	0.863056
32	1	0	5.634187	-1.089033	1.058985
33	1	0	6.074279	-1.428813	-1.102607
34	1	0	1.758352	-2.610502	-0.556549
35	1	0	2.113249	0.014679	-1.531714
36	1	0	1.414853	-1.081927	1.237932
37	1	0	-1.980158	1.768961	0.410784
38	1	0	-0.109277	1.447594	-1.047226
39	1	0	-0.602543	2.901565	-1.283033
40	1	0	3.279677	-2.690958	0.931169
41	1	0	2.169292	-3.316713	1.777995
42	1	0	1.592203	2.581611	-2.001668
43	1	0	2.586354	3.256891	-2.985925
44	1	0	3.468418	3.420760	-0.841480
45	1	0	3.596485	4.568261	0.191562
46	1	0	4.360404	-2.521688	-1.150037
47	1	0	5.268926	-3.639461	-0.624889
48	6	0	-2.919165	-2.727732	0.143379
49	6	0	-5.601486	-2.084929	-0.153379
50	6	0	-5.203708	-3.400459	-0.153698
51	1	0	-5.939635	-4.184783	-0.275935
52	6	0	-3.849722	-3.726554	-0.008636
53	1	0	-3.535447	-4.762259	-0.022041
54	1	0	-1.872978	-2.988715	0.237159
55	1	0	-6.651300	-1.862539	-0.281131
56	6	0	-4.440696	2.718246	0.079641
57	6	0	-6.411698	0.771788	-0.158222
58	6	0	-5.753523	3.092355	-0.060287
59	6	0	-6.744355	2.106292	-0.176814
60	1	0	-3.659097	3.462454	0.178413
61	1	0	-6.026306	4.139705	-0.076968
62	1	0	-7.202615	0.041263	-0.250876
63	1	0	-7.781776	2.397020	-0.282492

Structure 55b (M06-2X/def2-TZVP, Gas Phase)

Energy (Hartrees): = - 1243.7337425
No imaginary frequencies

Standard orientation:

Center Number	Atomic Number	Atomic Type	Coordinates (Angstroms) X	Y	Z
1	6	0	2.678023	-0.641033	-1.285927
2	6	0	2.063070	-0.502106	0.106158
3	6	0	2.937459	0.427755	0.935326
4	6	0	4.372169	-0.050461	0.932028
5	6	0	4.862820	-0.173083	-0.506537
6	1	0	2.654680	0.330983	-1.803728
7	1	0	2.905440	1.430052	0.486574
8	1	0	4.415119	-1.044711	1.397009
9	1	0	4.833494	0.819541	-0.979619
10	1	0	2.066046	-1.505889	0.553460
11	8	0	4.015838	-1.078278	-1.191203
12	6	0	6.282349	-0.712330	-0.600631
13	1	0	6.353521	-1.632064	-0.005821
14	1	0	6.484759	-0.969642	-1.640629
15	8	0	7.238844	0.241305	-0.210272
16	1	0	6.978022	0.586681	0.652306
17	8	0	5.206449	0.854307	1.629021
18	1	0	4.781253	1.049398	2.472559
19	8	0	2.518362	0.487978	2.283385
20	1	0	1.619236	0.834348	2.304681
21	8	0	1.956109	-1.600929	-1.972679
22	1	0	2.346963	-1.714037	-2.845433
23	7	0	0.734515	0.061195	0.023284
24	6	0	-1.675132	-0.408009	0.064136
25	6	0	-2.240435	0.909055	-0.171603
26	6	0	-2.506374	-1.465741	0.258807
27	6	0	-3.649521	1.062324	-0.177294
28	6	0	-3.926890	-1.349675	0.257074
29	1	0	-2.082521	-2.450094	0.427542
30	6	0	-4.514485	-0.086887	0.045749
31	6	0	-0.241251	-0.739091	0.125756
32	1	0	-0.054824	-1.809058	0.282801
33	6	0	-4.735274	-2.482094	0.469998
34	6	0	-6.102088	-2.375424	0.477058
35	6	0	-6.694328	-1.122334	0.270033
36	6	0	-5.919292	-0.006740	0.059675
37	1	0	-6.719987	-3.248136	0.642263
38	1	0	-7.772735	-1.029990	0.275559
39	1	0	-6.413502	0.940815	-0.095666
40	1	0	-4.256280	-3.440993	0.628841
41	6	0	-1.433180	2.040476	-0.403209
42	6	0	-4.185629	2.342489	-0.406433
43	6	0	-3.378256	3.431308	-0.624565
44	1	0	-3.819836	4.404327	-0.797543
45	6	0	-1.990013	3.276860	-0.625233
46	1	0	-1.349044	4.130732	-0.803573
47	1	0	-0.362606	1.918275	-0.407555
48	1	0	-5.256061	2.484490	-0.415223

Structure 55b (M06-2X/def2-TZVP, DMSO)

Energy (Hartrees): = - 1243.7658698
No imaginary frequencies

Standard orientation:

Center Number	Atomic Number	Atomic Type	Coordinates (Angstroms) X	Y	Z
1	6	0	2.766756	-0.404090	-1.464879
2	6	0	2.060260	-0.563085	-0.122026
3	6	0	2.844239	0.210558	0.937453
4	6	0	4.295950	-0.218854	0.935454
5	6	0	4.877587	-0.056657	-0.464924
6	1	0	2.757962	0.652678	-1.768153
7	1	0	2.797265	1.280601	0.698589
8	1	0	4.354926	-1.278478	1.215942
9	1	0	4.836473	1.004303	-0.749401
10	1	0	2.062739	-1.630982	0.136666
11	8	0	4.110455	-0.837803	-1.363245
12	6	0	6.316107	-0.538328	-0.562652
13	1	0	6.382075	-1.559290	-0.166569
14	1	0	6.604095	-0.563889	-1.614426
15	8	0	7.215666	0.328564	0.097754
16	1	0	6.856620	0.493332	0.979478
17	8	0	5.055947	0.568814	1.838145
18	1	0	4.610426	0.544907	2.695018
19	8	0	2.342612	-0.022628	2.239378
20	1	0	1.465235	0.375309	2.306557
21	8	0	2.125153	-1.202912	-2.394171
22	1	0	2.476477	-0.998186	-3.269935

23	7	0	0.725714	-0.015368	-0.198300
24	6	0	-1.668069	-0.417293	0.131163
25	6	0	-2.222116	0.912296	-0.062524
26	6	0	-2.506635	-1.473841	0.300363
27	6	0	-3.630724	1.070984	-0.115050
28	6	0	-3.926986	-1.351025	0.249140
29	1	0	-2.091460	-2.463298	0.461521
30	6	0	-4.505361	-0.085239	0.025153
31	6	0	-0.235696	-0.766090	0.143741
32	1	0	-0.046493	-1.800435	0.455294
33	6	0	-4.743561	-2.487736	0.406566
34	6	0	-6.109983	-2.380161	0.340724
35	6	0	-6.692353	-1.125494	0.109935
36	6	0	-5.909553	-0.004650	-0.045460
37	1	0	-6.735931	-3.255206	0.461578
38	1	0	-7.770058	-1.035750	0.051831
39	1	0	-6.397526	0.942570	-0.226013
40	1	0	-4.269949	-3.447729	0.576989
41	6	0	-1.407725	2.058382	-0.172269
42	6	0	-4.158814	2.364517	-0.289328
43	6	0	-3.342412	3.463853	-0.405126
44	1	0	-3.776732	4.446967	-0.537460
45	6	0	-1.954635	3.308771	-0.341069
46	1	0	-1.306985	4.173197	-0.419593
47	1	0	-0.336766	1.942976	-0.123397
48	1	0	-5.228739	2.510781	-0.325614

Structure 56 (M06-2X/def2-TZVP, Gas Phase)

Energy (Hartrees): = - 1243.7180576
No imaginary frequencies

Standard orientation:

Center Number	Atomic Number	Atomic Type	Coordinates (Angstroms) X	Y	Z
1	6	0	2.217620	-1.219769	-0.635845
2	6	0	1.718542	-0.144928	0.330980
3	6	0	2.677722	1.034550	0.296060
4	6	0	4.097195	0.570233	0.537039
5	6	0	4.463090	-0.505500	-0.479346
6	1	0	2.168902	-0.842164	-1.668926
7	1	0	2.625873	1.499987	-0.697426
8	1	0	4.157771	0.128059	1.540692
9	1	0	4.406724	-0.078198	-1.491198
10	1	0	1.725799	-0.594094	1.333544
11	8	0	3.547657	-1.577623	-0.338820
12	6	0	5.862788	-1.063313	-0.268939
13	1	0	5.978086	-1.351512	0.783901
14	1	0	5.969028	-1.962885	-0.875924
15	8	0	6.859217	-0.159609	-0.677769
16	1	0	6.682586	0.687978	-0.251424
17	8	0	5.007053	1.646447	0.418883
18	1	0	4.663128	2.376513	0.947034
19	8	0	2.382372	1.990388	1.295994
20	1	0	1.486240	2.314860	1.157114
21	8	0	1.419951	-2.340938	-0.467369
22	1	0	1.729699	-3.030020	-1.064402
23	7	0	0.397504	0.293081	-0.070678
24	6	0	-2.005546	-0.024756	0.188549
25	6	0	-2.851794	-1.151586	0.196054
26	6	0	-2.530075	1.260031	-0.049661
27	6	0	-4.258405	-0.983478	0.000066
28	6	0	-3.942632	1.410044	-0.241829
29	6	0	-4.767909	0.292528	-0.205840
30	6	0	-0.582322	-0.261595	0.506908
31	1	0	-0.403936	-0.999344	1.296665
32	6	0	-5.111846	-2.126978	0.013067
33	6	0	-2.358499	-2.486219	0.351136
34	6	0	-3.204596	-3.550227	0.354465
35	6	0	-4.605692	-3.372697	0.192933
36	1	0	-2.806482	-4.550289	0.468902
37	1	0	-1.293685	-2.660102	0.429840
38	1	0	-5.258852	-4.235398	0.200232
39	1	0	-6.174829	-1.973664	-0.130421
40	6	0	-1.732215	2.445816	-0.085031
41	6	0	-4.489283	2.710689	-0.459419
42	6	0	-2.291632	3.666405	-0.294245
43	1	0	-1.662736	4.547243	-0.319750
44	6	0	-3.692866	3.807754	-0.486388
45	1	0	-4.115885	4.789942	-0.652364
46	1	0	-5.559881	2.797304	-0.601985
47	1	0	-0.664139	2.352540	0.026604
48	1	0	-5.835828	0.420327	-0.347603

Structure 56 (M06-2X/def2-TZVP, DMSO)

Energy (Hartrees): = - 1243.751181
No imaginary frequencies

Standard orientation:

Center Number	Atomic Number	Atomic Type	Coordinates (Angstroms) X	Y	Z
1	6	0	2.446708	-1.032569	-1.262926
2	6	0	1.698005	-0.568403	-0.018652
3	6	0	2.455580	0.603114	0.608397
4	6	0	3.903890	0.224775	0.838422
5	6	0	4.530627	-0.253046	-0.466105
6	1	0	2.449069	-0.230515	-2.015580
7	1	0	2.421937	1.457773	-0.078733
8	1	0	3.943636	-0.599753	1.562361
9	1	0	4.509296	0.566772	-1.198455
10	1	0	1.676208	-1.400423	0.698052
11	8	0	3.785352	-1.360070	-0.940111
12	6	0	5.965400	-0.722947	-0.286749
13	1	0	5.998452	-1.468002	0.518071
14	1	0	6.292469	-1.206024	-1.208442
15	8	0	6.852976	0.347376	-0.035625
16	1	0	6.462378	0.881243	0.668703
17	8	0	4.641779	1.335561	1.321060
18	1	0	4.172291	1.686906	2.088838
19	8	0	1.923579	0.962830	1.869708
20	1	0	1.074138	1.403426	1.739362
21	8	0	1.829051	-2.174220	-1.741257
22	1	0	2.209818	-2.391804	-2.601550
23	7	0	0.370856	-0.124733	-0.385383
24	6	0	-1.998265	-0.095068	0.131916
25	6	0	-2.986970	-1.091745	0.257563
26	6	0	-2.362315	1.240698	-0.132425
27	6	0	-4.368217	-0.742566	0.121602
28	6	0	-3.752316	1.570048	-0.269242
29	6	0	-4.717129	0.577632	-0.137968
30	6	0	-0.590836	-0.499446	0.347693
31	1	0	-0.412945	-1.171670	1.193937
32	6	0	-5.367888	-1.754573	0.247182
33	6	0	-2.671475	-2.469672	0.484148
34	6	0	-3.652176	-3.405926	0.594102
35	6	0	-5.024026	-3.047007	0.481274
36	1	0	-3.388614	-4.442535	0.764118
37	1	0	-1.638103	-2.782443	0.549965
38	1	0	-5.785555	-3.810882	0.575575
39	1	0	-6.406516	-1.461732	0.145805
40	6	0	-1.420639	2.313684	-0.241147
41	6	0	-4.137411	2.921209	-0.528994
42	6	0	-1.828698	3.588866	-0.480741
43	1	0	-1.093270	4.380434	-0.556834
44	6	0	-3.207203	3.903475	-0.635134
45	1	0	-3.504411	4.925913	-0.831805
46	1	0	-5.194353	3.137630	-0.634054
47	1	0	-0.366765	2.104249	-0.144249
48	1	0	-5.764914	0.839555	-0.240902

Structure 56.5H_2O (M06-2X/def2-TZVP, Gas Phase)

Energy (Hartrees): = - 1625.9400225
No imaginary frequencies

Standard orientation:

Center Number	Atomic Number	Atomic Type	Coordinates (Angstroms) X	Y	Z
1	6	0	-3.465590	-0.577578	-0.951958
2	6	0	-2.569250	0.366952	-0.425196
3	6	0	-3.015403	1.395588	0.421644
4	6	0	-4.411061	1.469626	0.741048
5	6	0	-5.291507	0.536159	0.205994
6	6	0	-4.855343	-0.483480	-0.632312
7	6	0	-1.149999	0.266562	-0.826146
8	7	0	-0.203850	0.212067	0.008711
9	6	0	1.140681	0.085441	-0.497329
10	6	0	2.029375	1.163005	0.126551
11	8	0	3.360065	0.977939	-0.323670
12	6	0	3.972947	-0.257289	0.044578
13	6	0	3.160164	-1.401588	-0.555284
14	6	0	1.707505	-1.275169	-0.118289

15	8	0	1.647441	2.449241	-0.204605
16	6	0	5.394144	-0.200962	-0.480943
17	8	0	6.150449	0.809090	0.122356
18	8	0	3.654548	-2.675449	-0.230268
19	8	0	0.918771	-2.288191	-0.720983
20	8	0	3.411778	-2.529764	2.494447
21	8	0	0.918604	-3.170984	3.306710
22	8	0	-0.896841	-2.212445	1.527035
23	8	0	3.062610	2.314648	-2.706194
24	8	0	4.990862	3.344823	-0.706820
25	1	0	-0.949921	0.221066	-1.904254
26	1	0	1.665581	-1.377188	0.975562
27	1	0	1.474422	-3.080668	-0.746588
28	1	0	3.679873	-2.751325	0.745182
29	1	0	3.199814	-1.322754	-1.649154
30	1	0	3.985867	-0.343544	1.138331
31	1	0	5.878436	-1.156311	-0.276209
32	1	0	5.351617	-0.079360	-1.574187
33	1	0	5.834351	1.675137	-0.185560
34	1	0	1.715001	2.558986	-1.167227
35	1	0	1.992250	1.088069	1.218611
36	1	0	1.183159	0.197074	-1.592850
37	1	0	-0.972639	-1.247856	1.512615
38	1	0	-0.508503	-2.413940	0.660381
39	1	0	3.413425	1.627412	-2.114619
40	1	0	3.082934	1.969945	-3.602307
41	1	0	0.209307	-2.814308	2.727729
42	1	0	0.620514	-4.030468	3.611426
43	1	0	2.544313	-2.807188	2.862213
44	1	0	4.045475	-2.523621	3.214561
45	1	0	4.185024	3.168435	-0.205122
46	1	0	4.676108	3.439397	-1.613632
47	1	0	-6.346108	0.604635	0.450737
48	6	0	-2.150408	2.401850	0.957322
49	6	0	-4.876534	2.512028	1.597801
50	6	0	-4.020086	3.437076	2.097812
51	1	0	-4.381438	4.224310	2.746540
52	6	0	-2.638552	3.381487	1.762570
53	1	0	-1.966692	4.134093	2.154673
54	1	0	-1.096969	2.378069	0.717261
55	1	0	-5.933165	2.546162	1.834929
56	6	0	-5.760704	-1.444874	-1.172017
57	6	0	-3.036975	-1.665967	-1.773582
58	6	0	-3.931155	-2.562332	-2.267755
59	6	0	-5.317098	-2.448650	-1.970761
60	1	0	-1.982218	-1.795168	-1.982649
61	1	0	-6.811634	-1.356156	-0.923783
62	1	0	-6.011107	-3.174177	-2.374515
63	1	0	-3.587324	-3.381801	-2.885632

Structure 56.5H_2O (M06-2X/def2-TZVP, DMSO)

Energy (Hartrees): = - 1625.9843601
No imaginary frequencies

Standard orientation:

Center Number	Atomic Number	Atomic Type	Coordinates (Angstroms) X	Y	Z
1	6	0	-3.507194	-0.435465	-1.067730
2	6	0	-2.564225	0.383452	-0.423267
3	6	0	-2.959521	1.276373	0.588327
4	6	0	-4.341528	1.327715	0.968633
5	6	0	-5.265825	0.507954	0.330286
6	6	0	-4.882825	-0.367776	-0.680338
7	6	0	-1.151025	0.289916	-0.854749
8	7	0	-0.214206	0.068520	-0.034664
9	6	0	1.137255	-0.008621	-0.544427
10	6	0	1.964641	1.130819	0.060776
11	8	0	3.310002	1.006639	-0.366696
12	6	0	3.978367	-0.184446	0.041002
13	6	0	3.237930	-1.385216	-0.541622
14	6	0	1.773413	-1.327515	-0.129008
15	8	0	1.527703	2.385789	-0.324744
16	6	0	5.396798	-0.069600	-0.478581
17	8	0	6.079640	1.036623	0.062469
18	8	0	3.800656	-2.617789	-0.159981
19	8	0	1.051563	-2.401181	-0.713951

20	8	0	3.496207	-2.570178	2.559164
21	8	0	0.952190	-3.114391	3.372743
22	8	0	-0.837405	-2.349278	1.436962
23	8	0	2.940154	2.267288	-2.824396
24	8	0	4.728875	3.464517	-0.855848
25	1	0	-0.950334	0.401793	-1.925726
26	1	0	1.721719	-1.402555	0.967344
27	1	0	1.642220	-3.169237	-0.701587
28	1	0	3.766446	-2.677017	0.818191
29	1	0	3.295957	-1.333408	-1.635060
30	1	0	3.980913	-0.242475	1.137552
31	1	0	5.948043	-0.968630	-0.201870
32	1	0	5.362570	-0.017984	-1.575533
33	1	0	5.685966	1.848658	-0.297169
34	1	0	1.627677	2.459645	-1.289650
35	1	0	1.915865	1.088571	1.154145
36	1	0	1.170322	0.083559	-1.640278
37	1	0	-0.852740	-1.382739	1.339277
38	1	0	-0.379106	-2.619076	0.623166
39	1	0	3.283988	1.490283	-2.357454
40	1	0	2.827623	2.013815	-3.748375
41	1	0	0.268480	-2.843628	2.723415
42	1	0	0.717053	-4.003975	3.657806
43	1	0	2.604995	-2.807331	2.894883
44	1	0	4.133151	-2.856571	3.222101
45	1	0	3.976841	3.112364	-0.359989
46	1	0	4.427344	3.387943	-1.771698
47	1	0	-6.308530	0.553155	0.626662
48	6	0	-2.055130	2.175509	1.239021
49	6	0	-4.753183	2.231277	1.996006
50	6	0	-3.857917	3.053441	2.600212
51	1	0	-4.177249	3.737130	3.376717
52	6	0	-2.490594	3.028752	2.204137
53	1	0	-1.790091	3.706003	2.677155
54	1	0	-1.013654	2.178868	0.949811
55	1	0	-5.800807	2.245086	2.273932
56	6	0	-5.834524	-1.210514	-1.330161
57	6	0	-3.141096	-1.372434	-2.084460
58	6	0	-4.078100	-2.157672	-2.681182
59	6	0	-5.447663	-2.076133	-2.303063
60	1	0	-2.102773	-1.467779	-2.376067
61	1	0	-6.871805	-1.143793	-1.022659
62	1	0	-6.174328	-2.713768	-2.790559
63	1	0	-3.781327	-2.859743	-3.450505

Structure 56.5H_2O (M06-2X/def2-TZVP, H_2O)

Energy (Hartrees): = - 1626.0007891
No imaginary frequencies

Standard orientation:

Center Number	Atomic Number	Atomic Type	Coordinates (Angstroms) X	Y	Z
1	6	0	-3.376604	-0.654750	-0.850312
2	6	0	-2.520298	0.377475	-0.428471
3	6	0	-3.021783	1.477028	0.288654
4	6	0	-4.419742	1.522507	0.605687
5	6	0	-5.256873	0.490715	0.194303
6	6	0	-4.769751	-0.592989	-0.528499
7	6	0	-1.089452	0.281833	-0.798691
8	7	0	-0.154084	0.333605	0.054160
9	6	0	1.195757	0.174286	-0.448761
10	6	0	2.101796	1.220042	0.194254
11	8	0	3.440472	1.008367	-0.222345
12	6	0	3.995163	-0.250664	0.153776
13	6	0	3.173284	-1.370323	-0.482117
14	6	0	1.710088	-1.212759	-0.097906
15	8	0	1.757432	2.518733	-0.174525
16	6	0	5.430596	-0.264686	-0.323800
17	8	0	6.243022	0.671020	0.361713
18	8	0	3.636960	-2.650115	-0.103594
19	8	0	0.905975	-2.168482	-0.776184
20	8	0	3.193251	-2.895422	2.610123
21	8	0	0.648741	-2.010559	3.234586
22	8	0	-1.108926	-2.645733	1.159482
23	8	0	2.826179	2.070429	-2.875832
24	8	0	5.280580	3.116311	-0.777485
25	1	0	-0.875091	0.142393	-1.863740
26	1	0	1.624225	-1.363555	0.986125
27	1	0	1.346726	-3.027174	-0.695404
28	1	0	3.493491	-2.754669	0.863237
29	1	0	3.265597	-1.301455	-1.571239
30	1	0	3.962153	-0.347838	1.247428
31	1	0	5.849234	-1.255050	-0.147617
32	1	0	5.441537	-0.071701	-1.402912

33	1	0	6.045604	1.555637	0.009207
34	1	0	1.835858	2.580466	-1.144059
35	1	0	2.042271	1.161211	1.285272
36	1	0	1.250438	0.302582	-1.538806
37	1	0	-1.824043	-1.997288	1.126416
38	1	0	-0.543919	-2.437822	0.391182
39	1	0	3.279260	1.462352	-2.271492
40	1	0	2.207625	1.514043	-3.364619
41	1	0	0.002523	-2.240517	2.535832
42	1	0	0.296956	-2.391009	4.047754
43	1	0	2.276604	-2.640458	2.848376
44	1	0	3.292747	-3.811419	2.893462
45	1	0	4.444737	2.646721	-0.625227
46	1	0	5.391193	3.117752	-1.735902
47	1	0	-6.312400	0.533559	0.440595
48	6	0	-2.209382	2.585009	0.690536
49	6	0	-4.936670	2.637770	1.333818
50	6	0	-4.126116	3.659205	1.711240
51	1	0	-4.524868	4.502691	2.260252
52	6	0	-2.743093	3.632173	1.374071
53	1	0	-2.112182	4.463134	1.663331
54	1	0	-1.157939	2.585933	0.440421
55	1	0	-5.994257	2.646582	1.570162
56	6	0	-5.631958	-1.650882	-0.948999
57	6	0	-2.903108	-1.798235	-1.569506
58	6	0	-3.757263	-2.784623	-1.953554
59	6	0	-5.144374	-2.711695	-1.643070
60	1	0	-1.848456	-1.887608	-1.799119
61	1	0	-6.683677	-1.584840	-0.696143
62	1	0	-5.802959	-3.510793	-1.958156
63	1	0	-3.381178	-3.642124	-2.497155

Structure 93 (M06-2X/6-311G(d,p), Gas Phase)

Energy (Hartrees): = - 936.3468146
No imaginary frequencies

Standard orientation:

Center Number	Atomic Number	Atomic Type	Coordinates (Angstroms) X	Y	Z
1	6	0	1.033456	1.340609	0.371620
2	6	0	0.443288	0.127985	-0.350309
3	6	0	1.316650	-1.087389	-0.065326
4	6	0	2.760298	-0.795476	-0.411935
5	6	0	3.223194	0.440192	0.355408
6	1	0	0.983018	1.187589	1.462523
7	1	0	1.258030	-1.316463	1.008427
8	1	0	2.830712	-0.582489	-1.488345
9	1	0	3.155675	0.236745	1.435084
10	1	0	0.472880	0.360351	-1.424455
11	8	0	2.383405	1.525729	-0.004420
12	6	0	4.659690	0.830348	0.034558
13	1	0	4.778294	0.888637	-1.056447
14	1	0	4.845061	1.823251	0.447404
15	8	0	5.582517	-0.053664	0.623418
16	1	0	5.322342	-0.946369	0.369629
17	8	0	3.590413	-1.889664	-0.069073
18	1	0	3.175486	-2.679675	-0.432313
19	8	0	0.925371	-2.214102	-0.825169
20	1	0	0.024369	-2.436849	-0.570767
21	8	0	0.315767	2.454540	-0.026217
22	1	0	0.697017	3.223396	0.407319
23	7	0	-0.897773	-0.148096	0.121821
24	6	0	-3.271552	0.076370	-0.192337
25	6	0	-3.605969	-0.580956	0.994883
26	6	0	-4.283754	0.543031	-1.029638
27	6	0	-4.936694	-0.768719	1.332055
28	1	0	-2.806096	-0.931890	1.635469
29	6	0	-5.618815	0.354416	-0.690410
30	1	0	-4.022553	1.057166	-1.948812
31	6	0	-5.945449	-0.302219	0.489872
32	1	0	-5.194153	-1.278819	2.252511
33	1	0	-6.400879	0.719365	-1.344965
34	1	0	-6.985028	-0.451070	0.756525
35	6	0	-1.863330	0.280254	-0.577871
36	1	0	-1.695634	0.830413	-1.513259

Structure 93 (M06-2X/6-311G(d,p), DMSO)

Energy (Hartrees): = - 936.3730451
No imaginary frequencies

Standard orientation:

Center Number	Atomic Number	Atomic Type	Coordinates (Angstroms) X	Y	Z
1	6	0	1.148351	1.485583	0.194567
2	6	0	0.447767	0.239314	-0.342539
3	6	0	1.222645	-1.000315	0.107543
4	6	0	2.681860	-0.875748	-0.279180
5	6	0	3.255857	0.420855	0.285866
6	1	0	1.121456	1.483468	1.295246
7	1	0	1.157865	-1.082496	1.200772
8	1	0	2.758305	-0.843450	-1.374928
9	1	0	3.190217	0.392616	1.383784
10	1	0	0.464100	0.295226	-1.440304
11	8	0	2.501603	1.506057	-0.229529
12	6	0	4.707759	0.639848	-0.113875
13	1	0	4.797211	0.542200	-1.204514
14	1	0	4.995094	1.656697	0.160441
15	8	0	5.579143	-0.244852	0.562321
16	1	0	5.209012	-1.129157	0.452074
17	8	0	3.431095	-1.966776	0.234596
18	1	0	2.980808	-2.773972	-0.042365
19	8	0	0.736586	-2.180909	-0.505997
20	1	0	-0.128516	-2.380245	-0.130377
21	8	0	0.514678	2.596428	-0.332039
22	1	0	0.888637	3.378202	0.090886
23	7	0	-0.896113	0.165093	0.189732
24	6	0	-3.262312	-0.020837	-0.216187
25	6	0	-3.624813	0.011730	1.134580
26	6	0	-4.253419	-0.134962	-1.192426
27	6	0	-4.961073	-0.068925	1.497300
28	1	0	-2.850908	0.103386	1.887860
29	6	0	-5.593527	-0.216204	-0.827224
30	1	0	-3.969577	-0.158320	-2.239891
31	6	0	-5.948008	-0.182104	0.517213
32	1	0	-5.239072	-0.042294	2.544753
33	1	0	-6.358280	-0.305406	-1.590053
34	1	0	-6.991407	-0.243261	0.804938
35	6	0	-1.851123	0.070700	-0.638841
36	1	0	-1.676777	0.057955	-1.722036

Structure 93 (M06-2X/def2-TZVP, Gas Phase)

Energy (Hartrees): = -936.464045
No imaginary frequencies

Standard orientation:

Center Number	Atomic Number	Atomic Type	Coordinates (Angstroms) X	Y	Z
1	6	0	1.051054	1.354870	0.361005
2	6	0	0.442268	0.139439	-0.336804
3	6	0	1.302431	-1.079148	-0.031002
4	6	0	2.746948	-0.811150	-0.389281
5	6	0	3.229993	0.434886	0.345476
6	1	0	1.004184	1.220634	1.453155
7	1	0	1.244300	-1.285747	1.046144
8	1	0	2.814654	-0.622223	-1.469185
9	1	0	3.175632	0.255261	1.429290
10	1	0	0.468991	0.347806	-1.415248
11	8	0	2.398148	1.519759	-0.023530
12	6	0	4.661579	0.809243	-0.008202
13	1	0	4.763027	0.842192	-1.100511
14	1	0	4.860084	1.809666	0.377369
15	8	0	5.596633	-0.060844	0.579947
16	1	0	5.335563	-0.964682	0.364636
17	8	0	3.567080	-1.906193	-0.030486
18	1	0	3.145770	-2.705891	-0.367167
19	8	0	0.892452	-2.215047	-0.765197
20	1	0	-0.010022	-2.431980	-0.506113
21	8	0	0.344782	2.472797	-0.045880

22	1	0	0.733792	3.250009	0.368434
23	7	0	-0.896434	-0.105396	0.146588
24	6	0	-3.270796	0.057153	-0.199325
25	6	0	-3.614693	-0.490420	1.036625
26	6	0	-4.274666	0.439052	-1.083759
27	6	0	-4.945645	-0.654031	1.374686
28	1	0	-2.823065	-0.777727	1.716301
29	6	0	-5.610079	0.275271	-0.743834
30	1	0	-4.005273	0.867749	-2.042365
31	6	0	-5.945678	-0.272245	0.485046
32	1	0	-5.210891	-1.078701	2.334332
33	1	0	-6.386015	0.575092	-1.435961
34	1	0	-6.986293	-0.401666	0.753475
35	6	0	-1.863596	0.239387	-0.590547
36	1	0	-1.696889	0.700371	-1.572038

Structure 93 (M06-2X/def2-TZVP, DMSO)

Energy (Hartrees): = -936.490491
No imaginary frequencies

Standard orientation:

Center Number	Atomic Number	Atomic Type	Coordinates (Angstroms) X	Y	Z
1	6	0	1.149463	1.481145	0.201544
2	6	0	0.447430	0.238331	-0.338120
3	6	0	1.220943	-0.999975	0.113309
4	6	0	2.679312	-0.879402	-0.273127
5	6	0	3.256139	0.415782	0.288632
6	1	0	1.119130	1.480146	1.300867
7	1	0	1.154702	-1.078890	1.205777
8	1	0	2.756213	-0.847091	-1.367744
9	1	0	3.198614	0.388000	1.386214
10	1	0	0.465143	0.292691	-1.435181
11	8	0	2.500711	1.500826	-0.218225
12	6	0	4.701417	0.640708	-0.126067
13	1	0	4.781598	0.543647	-1.215984
14	1	0	4.987888	1.657824	0.144568
15	8	0	5.590355	-0.235957	0.535419
16	1	0	5.233393	-1.128588	0.438990
17	8	0	3.429373	-1.969389	0.237877
18	1	0	2.988942	-2.783895	-0.037301
19	8	0	0.726843	-2.178850	-0.493803
20	1	0	-0.154512	-2.359693	-0.143303
21	8	0	0.521767	2.597429	-0.321812
22	1	0	0.875556	3.382035	0.115936
23	7	0	-0.895174	0.164573	0.188915
24	6	0	-3.261542	-0.020671	-0.213092
25	6	0	-3.630271	0.006493	1.133164
26	6	0	-4.246673	-0.128473	-1.192050
27	6	0	-4.965798	-0.073640	1.488098
28	1	0	-2.863004	0.093446	1.892294
29	6	0	-5.586315	-0.208661	-0.835150
30	1	0	-3.957527	-0.147440	-2.237019
31	6	0	-5.946454	-0.180353	0.504822
32	1	0	-5.248857	-0.051687	2.533246
33	1	0	-6.346558	-0.292728	-1.601585
34	1	0	-6.990355	-0.240853	0.787051
35	6	0	-1.852285	0.070378	-0.633474
36	1	0	-1.678728	0.058623	-1.715814

Structure 93.5H_2O (M06-2X/def2-TZVP, Gas Phase)

Energy (Hartrees): = -1318.683000
No imaginary frequencies

Standard orientation:

Center Number	Atomic Number	Atomic Type	Coordinates (Angstroms) X	Y	Z
1	6	0	-4.063549	-0.006630	-0.932881

2	6	0	-3.633216	-0.785347	0.141238
3	6	0	-4.567470	-1.491859	0.893392
4	6	0	-5.918640	-1.410483	0.591542
5	6	0	-6.340611	-0.626165	-0.471633
6	6	0	-5.410768	0.072141	-1.235250
7	6	0	-2.212621	-0.878022	0.506809
8	7	0	-1.317767	-0.132774	0.014842
9	6	0	0.048518	-0.336323	0.436113
10	6	0	0.849879	-0.956618	-0.718124
11	8	0	2.206132	-1.077415	-0.327694
12	6	0	2.881407	0.144326	-0.037953
13	6	0	2.174928	0.810663	1.136289
14	6	0	0.697160	0.988603	0.811421
15	8	0	0.407321	-2.214943	-1.075961
16	6	0	4.320233	-0.228224	0.261879
17	8	0	4.965045	-0.800110	-0.839835
18	8	0	2.737870	2.042595	1.509968
19	8	0	0.022804	1.547811	1.925512
20	8	0	2.388008	3.399249	-0.851181
21	8	0	-0.126692	4.320845	-1.213698
22	8	0	-1.858495	2.709115	0.108988
23	8	0	1.944803	-3.450424	1.022174
24	8	0	3.656980	-3.349780	-1.399107
25	1	0	-1.964999	-1.637987	1.258762
26	1	0	0.617061	1.667901	-0.049709
27	1	0	0.649279	2.164328	2.331620
28	1	0	2.728352	2.630111	0.727737
29	1	0	2.258102	0.153381	2.011362
30	1	0	2.844963	0.798296	-0.918206
31	1	0	4.863562	0.674737	0.541720
32	1	0	4.326844	-0.901454	1.132832
33	1	0	4.589453	-1.679835	-1.013031
34	1	0	0.516305	-2.811403	-0.317795
35	1	0	0.774851	-0.322097	-1.607567
36	1	0	0.113239	-1.024829	1.293445
37	1	0	-3.332220	0.518476	-1.533399
38	1	0	-5.740337	0.675991	-2.070542
39	1	0	-7.394131	-0.561868	-0.711505
40	1	0	-6.639757	-1.958369	1.183625
41	1	0	-4.232126	-2.103021	1.723508
42	1	0	-1.876878	1.814199	-0.269344
43	1	0	-1.448414	2.544978	0.972673
44	1	0	2.307376	-2.564058	0.853315
45	1	0	2.062024	-3.645302	1.955174
46	1	0	-0.807926	3.762677	-0.775048
47	1	0	-0.407317	5.232051	-1.106984
48	1	0	1.510668	3.806757	-1.020662
49	1	0	2.985376	3.695846	-1.540611
50	1	0	2.843847	-2.913277	-1.682728
51	1	0	3.375481	-3.878734	-0.642924

Structure 93.5H_2O (M06-2X/def2-TZVP, DMSO)

Energy (Hartrees): = -1318.723303
No imaginary frequencies

Standard orientation:

Center Number	Atomic Number	Atomic Type	Coordinates (Angstroms) X	Y	Z
1	6	0	-4.059875	-0.048776	-0.961306
2	6	0	-3.628587	-0.770548	0.153329
3	6	0	-4.564111	-1.424328	0.952270
4	6	0	-5.917797	-1.343882	0.656137
5	6	0	-6.341441	-0.616364	-0.447537
6	6	0	-5.410119	0.027483	-1.257839
7	6	0	-2.206272	-0.864110	0.517924
8	7	0	-1.309682	-0.145283	-0.014333
9	6	0	0.058055	-0.343092	0.413025
10	6	0	0.861015	-0.960627	-0.740016
11	8	0	2.216268	-1.089020	-0.346387
12	6	0	2.890477	0.128795	-0.048089
13	6	0	2.176762	0.809517	1.114965
14	6	0	0.703227	0.987873	0.774239
15	8	0	0.422104	-2.221600	-1.099946
16	6	0	4.317704	-0.254599	0.284751
17	8	0	4.965160	-0.890575	-0.791062

18	8	0	2.746672	2.047159	1.467523
19	8	0	0.015195	1.567650	1.872587
20	8	0	2.481994	3.707786	-0.701919
21	8	0	-0.118677	4.285393	-1.252373
22	8	0	-1.891527	2.686786	0.087433
23	8	0	1.918831	-3.521261	0.985189
24	8	0	3.592177	-3.417323	-1.379027
25	1	0	-1.962764	-1.592120	1.299522
26	1	0	0.631397	1.653520	-0.098799
27	1	0	0.626554	2.205174	2.271165
28	1	0	2.700494	2.645525	0.690772
29	1	0	2.255291	0.161200	1.995522
30	1	0	2.874235	0.778602	-0.933989
31	1	0	4.880620	0.647344	0.526516
32	1	0	4.306474	-0.894708	1.177495
33	1	0	4.538997	-1.750415	-0.944893
34	1	0	0.547950	-2.814461	-0.339610
35	1	0	0.789008	-0.323570	-1.628047
36	1	0	0.121619	-1.022771	1.275731
37	1	0	-3.332082	0.433596	-1.602582
38	1	0	-5.740246	0.584762	-2.125625
39	1	0	-7.396725	-0.554916	-0.683131
40	1	0	-6.640086	-1.849457	1.284529
41	1	0	-4.225584	-1.992368	1.811596
42	1	0	-1.901187	1.799197	-0.311254
43	1	0	-1.422814	2.513354	0.920865
44	1	0	2.240206	-2.608513	1.045858
45	1	0	1.862536	-3.862402	1.885920
46	1	0	-0.799401	3.741752	-0.799955
47	1	0	-0.406728	5.201496	-1.177010
48	1	0	1.550684	3.938711	-0.911050
49	1	0	2.892609	3.457156	-1.536607
50	1	0	2.837042	-2.878416	-1.651512
51	1	0	3.279882	-3.804298	-0.548362

Structure 93.5H_2O (M06-2X/def2-TZVP, H_2O)

Energy (Hartrees): = -1318.742159
No imaginary frequencies

Standard orientation:

Center Number	Atomic Number	Atomic Type	Coordinates (Angstroms) X	Y	Z
1	6	0	-4.133722	0.486590	-0.677365
2	6	0	-3.663101	-0.571061	0.103558
3	6	0	-4.567813	-1.496699	0.621511
4	6	0	-5.927403	-1.364188	0.376384
5	6	0	-6.389330	-0.307525	-0.395613
6	6	0	-5.489997	0.615112	-0.924214
7	6	0	-2.237835	-0.754764	0.411136
8	7	0	-1.337282	0.093653	0.125944
9	6	0	0.026866	-0.258659	0.473980
10	6	0	0.754235	-0.807440	-0.758173
11	8	0	2.093979	-1.105386	-0.402731
12	6	0	2.868575	0.019985	0.004705
13	6	0	2.237981	0.639565	1.250275
14	6	0	0.775570	0.961781	0.979075
15	8	0	0.184458	-1.983046	-1.236856
16	6	0	4.273235	-0.477905	0.267412
17	8	0	4.916205	-0.938840	-0.907237
18	8	0	2.924054	1.805770	1.660669
19	8	0	0.129566	1.427587	2.150980
20	8	0	2.865303	3.526904	-0.516239
21	8	0	0.441403	3.262882	-1.849659
22	8	0	-1.612941	2.944097	0.005602
23	8	0	1.109480	-3.518587	1.014492
24	8	0	3.484268	-3.378043	-1.383603
25	1	0	-1.985329	-1.685137	0.930833
26	1	0	0.745157	1.739233	0.203418
27	1	0	0.640618	2.177362	2.484751
28	1	0	2.897016	2.446980	0.917258
29	1	0	2.298161	-0.079009	2.074589
30	1	0	2.877940	0.761058	-0.806810
31	1	0	4.867644	0.343125	0.666997
32	1	0	4.227578	-1.271739	1.022099
33	1	0	4.546375	-1.810099	-1.129899
34	1	0	0.231116	-2.644691	-0.520409

35	1	0	0.743035	-0.077061	-1.573679
36	1	0	0.054828	-1.045315	1.239921
37	1	0	-3.436991	1.200530	-1.096878
38	1	0	-5.850529	1.434722	-1.532545
39	1	0	-7.448933	-0.201583	-0.591359
40	1	0	-6.623964	-2.084592	0.785548
41	1	0	-4.198784	-2.320330	1.221914
42	1	0	-1.602514	1.961453	-0.018010
43	1	0	-1.195858	3.158971	0.850095
44	1	0	1.742433	-2.784986	0.987466
45	1	0	0.583667	-3.358435	1.808068
46	1	0	-0.307729	3.221445	-1.219478
47	1	0	0.278369	4.039941	-2.396887
48	1	0	2.008023	3.428954	-0.982385
49	1	0	3.514684	3.111101	-1.096188
50	1	0	2.766352	-2.770793	-1.139597
51	1	0	3.538839	-4.002115	-0.649803

Structure 105 (M06-2X/def2-TZVP, Gas Phase)

Energy (Hartrees): = - 1013.860834
No imaginary frequencies

Standard orientation:

Center Number	Atomic Number	Atomic Type	Coordinates (Angstroms) X	Y	Z
1	6	0	2.260233	-1.563729	0.533556
2	6	0	1.414381	-0.280324	0.474643
3	6	0	2.098216	0.729679	-0.446392
4	6	0	3.524171	0.942784	0.013201
5	6	0	4.263139	-0.390679	0.026106
6	1	0	2.116492	0.338760	-1.469228
7	1	0	3.506311	1.342263	1.037089
8	1	0	4.299248	-0.795512	-0.990523
9	1	0	1.364214	0.154466	1.481354
10	8	0	3.575108	-1.281392	0.893900
11	6	0	5.681561	-0.266801	0.560544
12	1	0	5.656178	0.258068	1.524830
13	1	0	6.069397	-1.270410	0.735314
14	8	0	6.544080	0.364164	-0.354465
15	1	0	6.119526	1.179066	-0.647940
16	8	0	4.200603	1.837161	-0.848419
17	1	0	3.637385	2.611781	-0.960281
18	8	0	1.454785	1.991380	-0.409794
19	1	0	0.623183	1.931523	-0.890240
20	7	0	0.107122	-0.594954	-0.052812
21	6	0	-2.259569	-0.301449	0.021650
22	6	0	-3.310807	0.278658	0.609828
23	1	0	-3.117209	0.929904	1.459414
24	6	0	-0.907483	-0.073708	0.503836
25	1	0	-0.802731	0.578891	1.380525
26	1	0	1.882794	-2.222450	1.321647
27	8	0	2.242228	-2.216590	-0.705894
28	1	0	1.349847	-2.116597	-1.064275
29	6	0	-4.719988	0.147572	0.237240
30	6	0	-5.157327	-0.709223	-0.777039
31	6	0	-5.668979	0.907970	0.922043
32	6	0	-6.500522	-0.795142	-1.094667
33	1	0	-4.444258	-1.317614	-1.317794
34	6	0	-7.015544	0.823863	0.603400
35	1	0	-5.341713	1.572936	1.712722
36	6	0	-7.434866	-0.028038	-0.407096
37	1	0	-6.824533	-1.464223	-1.881193
38	1	0	-7.736402	1.422312	1.144914
39	1	0	-8.485107	-0.098343	-0.658502
40	1	0	-2.356595	-0.956537	-0.835896

Structure 105 (M06-2X/def2-TZVP, DMSO)

Energy (Hartrees): = - 1013.8892944
No imaginary frequencies

Standard orientation:

Center Number	Atomic Number	Atomic Type	Coordinates (Angstroms) X	Y	Z
1	6	0	2.175782	-1.528661	0.478697
2	6	0	1.402500	-0.201803	0.430649
3	6	0	2.157395	0.804609	-0.436982

4	6	0	3.585228	0.919602	0.050360
5	6	0	4.248656	-0.452002	0.033166
6	1	0	2.168262	0.458624	-1.476674
7	1	0	3.580162	1.292763	1.083080
8	1	0	4.279009	-0.826289	-0.995998
9	1	0	1.337219	0.198122	1.449585
10	8	0	3.504585	-1.336610	0.860386
11	6	0	5.661280	-0.422184	0.594353
12	1	0	5.646512	0.063152	1.578401
13	1	0	6.005331	-1.448716	0.728452
14	8	0	6.571399	0.214960	-0.278993
15	1	0	6.173454	1.056051	-0.538504
16	8	0	4.334299	1.792461	-0.780314
17	1	0	3.843267	2.621465	-0.850383
18	8	0	1.583605	2.096989	-0.358022
19	1	0	0.738416	2.089101	-0.824359
20	7	0	0.100109	-0.446211	-0.148114
21	6	0	-2.267467	-0.188760	-0.055079
22	6	0	-3.328074	0.266374	0.622262
23	1	0	-3.148024	0.783048	1.562240
24	6	0	-0.922727	0.000991	0.460740
25	1	0	-0.829824	0.543697	1.408519
26	1	0	1.747118	-2.174084	1.250796
27	8	0	2.139714	-2.172119	-0.769082
28	1	0	1.292000	-1.942700	-1.177392
29	6	0	-4.736271	0.137472	0.241267
30	6	0	-5.147069	-0.473302	-0.948968
31	6	0	-5.710629	0.641737	1.106232
32	6	0	-6.492841	-0.574991	-1.257187
33	1	0	-4.412926	-0.871234	-1.638480
34	6	0	-7.059433	0.538788	0.797181
35	1	0	-5.399941	1.117365	2.029687
36	6	0	-7.454184	-0.070432	-0.385813
37	1	0	-6.797528	-1.050310	-2.181386
38	1	0	-7.800482	0.934569	1.480459
39	1	0	-8.505718	-0.152707	-0.631251
40	1	0	-2.358768	-0.718526	-0.997044

Structure 105.5H_2O (M06-2X/def2-TZVP, Gas Phase)

Energy (Hartrees): = - 1013.860834
No imaginary frequencies

Standard orientation:

Center Number	Atomic Number	Atomic Type	Coordinates (Angstroms) X	Y	Z
1	6	0	3.023521	-0.593387	-0.157740
2	6	0	1.735652	-0.451016	-0.812879
3	7	0	0.643102	-0.711937	-0.221095
4	6	0	-0.595247	-0.512664	-0.945988
5	6	0	-1.249294	0.780371	-0.465421
6	6	0	-2.679756	0.918590	-0.970477
7	6	0	-3.452745	-0.348974	-0.627495
8	8	0	-2.791016	-1.485536	-1.176217
9	6	0	-1.511182	-1.715313	-0.693455
10	8	0	-0.399612	1.829321	-0.890879
11	8	0	-1.579118	-2.008401	0.682606
12	6	0	-4.852252	-0.357619	-1.201336
13	8	0	-5.650782	-1.378648	-0.641015
14	8	0	-3.338607	1.985906	-0.317805
15	1	0	1.747673	-0.098941	-1.850824
16	1	0	-0.433468	-0.425090	-2.027565
17	1	0	-0.838229	2.696288	-0.810065
18	1	0	-1.303217	0.753235	0.631002
19	1	0	-3.025209	2.837687	-0.655365
20	1	0	-2.672740	1.062827	-2.059278
21	1	0	-5.101602	-2.166300	-0.463416
22	1	0	-5.332125	0.596028	-0.982369
23	1	0	-4.779765	-0.476561	-2.288756
24	1	0	-3.513104	-0.439478	0.463794
25	1	0	-0.701283	-1.845412	1.054735
26	1	0	-1.143213	-2.585775	-1.243648
27	1	0	-4.650241	-2.690565	1.658088
28	8	0	-4.247749	-3.220963	0.953639
29	1	0	-3.308377	-2.994047	0.966731
30	1	0	-1.659551	4.109671	0.554818
31	8	0	-1.716688	4.248635	-0.420743
32	1	0	-1.512158	5.163738	-0.622867
33	8	0	-1.662161	3.185430	2.005474
34	1	0	-0.832386	2.682657	2.104431
35	1	0	-2.352407	2.542242	1.798222
36	1	0	0.696265	1.906312	0.709684
37	8	0	0.808968	1.843769	1.673857

38	1	0	0.954823	0.904158	1.826766
39	8	0	-5.680422	-1.066087	2.103553
40	1	0	-5.914395	-1.049685	1.156775
41	1	0	-6.493017	-0.939885	2.597451
42	6	0	4.161029	-0.303063	-0.797784
43	1	0	3.006085	-0.946330	0.866764
44	1	0	4.085458	0.047766	-1.824410
45	6	0	5.523698	-0.399657	-0.274469
46	6	0	6.585722	-0.022923	-1.098115
47	6	0	5.808810	-0.855407	1.015825
48	6	0	7.895892	-0.095628	-0.650750
49	1	0	6.376340	0.331477	-2.100635
50	6	0	7.115355	-0.929237	1.462829
51	1	0	5.005047	-1.155540	1.675165
52	6	0	8.163907	-0.549528	0.631561
53	1	0	8.706462	0.201540	-1.303142
54	1	0	7.320655	-1.284441	2.464305
55	1	0	9.184799	-0.608703	0.985677

Structure 105.5H₂O (M06-2X/def2-TZVP, DMSO)

Energy (Hartrees): = - 1396.1212683
No imaginary frequencies

Standard orientation:

Center Number	Atomic Number	Atomic Type	Coordinates (Angstroms) X	Y	Z
1	6	0	3.017414	-0.575296	-0.181076
2	6	0	1.738004	-0.418836	-0.850198
3	7	0	0.639523	-0.641235	-0.250035
4	6	0	-0.597554	-0.428701	-0.973293
5	6	0	-1.258070	0.840954	-0.443227
6	6	0	-2.696205	0.986834	-0.920682
7	6	0	-3.452456	-0.296645	-0.606486
8	8	0	-2.796023	-1.413277	-1.200681
9	6	0	-1.499457	-1.646120	-0.750433
10	8	0	-0.430872	1.920474	-0.843127
11	8	0	-1.546007	-1.996940	0.615794
12	6	0	-4.859916	-0.304913	-1.153145
13	8	0	-5.591021	-1.428032	-0.682792
14	8	0	-3.347909	2.030983	-0.214255
15	1	0	1.761654	-0.095671	-1.896191
16	1	0	-0.439414	-0.314075	-2.051657
17	1	0	-0.890561	2.772923	-0.731280
18	1	0	-1.291889	0.774387	0.653242
19	1	0	-3.070300	2.890655	-0.564163
20	1	0	-2.715048	1.172503	-2.001328
21	1	0	-4.979422	-2.175994	-0.561396
22	1	0	-5.385252	0.592092	-0.826535
23	1	0	-4.818326	-0.308221	-2.247612
24	1	0	-3.488325	-0.421997	0.483097
25	1	0	-0.688214	-1.765036	1.004437
26	1	0	-1.131944	-2.490381	-1.338461
27	1	0	-4.614536	-2.810586	1.551368
28	8	0	-4.112559	-3.327853	0.901259
29	1	0	-3.217795	-2.956552	0.905637
30	1	0	-1.670485	4.242371	0.606000
31	8	0	-1.785460	4.334555	-0.361681
32	1	0	-1.544162	5.230870	-0.620177
33	8	0	-1.617990	3.162352	2.073188
34	1	0	-0.769702	2.680301	2.088519
35	1	0	-2.273997	2.531574	1.744113
36	1	0	0.713427	1.930021	0.690975
37	8	0	0.895270	1.909597	1.647421
38	1	0	1.037178	0.972813	1.835291
39	8	0	-5.841746	-1.407940	2.084580
40	1	0	-5.979437	-1.278451	1.128024
41	1	0	-6.711614	-1.562231	2.468356
42	6	0	4.162408	-0.320782	-0.825749
43	1	0	2.989447	-0.901180	0.853385
44	1	0	4.102913	0.007398	-1.860955
45	6	0	5.518371	-0.432390	-0.284457
46	6	0	6.594253	-0.077350	-1.101963
47	6	0	5.781633	-0.880015	1.014878
48	6	0	7.899191	-0.162452	-0.637707
49	1	0	6.398258	0.268418	-2.110548
50	6	0	7.083693	-0.965641	1.477233
51	1	0	4.965941	-1.165376	1.667148
52	6	0	8.147079	-0.606991	0.653429
53	1	0	8.721756	0.117353	-1.283897
54	1	0	7.273627	-1.314586	2.484699
55	1	0	9.163764	-0.675989	1.019779

Structure 105.5H_2O (M06-2X/def2-TZVP, H_2O)

Energy (Hartrees): = - 1396.1381933
No imaginary frequencies

Standard orientation:

Center Number	Atomic Number	Atomic Type	Coordinates (Angstroms) X	Y	Z
1	6	0	2.974698	-0.670674	-0.094975
2	6	0	1.673134	-0.566258	-0.729494
3	7	0	0.589571	-0.796238	-0.101051
4	6	0	-0.650414	-0.614618	-0.827499
5	6	0	-1.277710	0.725621	-0.450556
6	6	0	-2.674354	0.846912	-1.046203
7	6	0	-3.517180	-0.367065	-0.671653
8	8	0	-2.859731	-1.578171	-1.039403
9	6	0	-1.590834	-1.765800	-0.479644
10	8	0	-0.404678	1.730175	-0.945411
11	8	0	-1.721803	-1.905793	0.918650
12	6	0	-4.836975	-0.373338	-1.410588
13	8	0	-5.666042	-1.452117	-0.997520
14	8	0	-3.365889	1.976298	-0.542626
15	1	0	1.666705	-0.280168	-1.786065
16	1	0	-0.496848	-0.623469	-1.912394
17	1	0	-0.773663	2.616339	-0.754795
18	1	0	-1.358993	0.801611	0.641999
19	1	0	-2.870413	2.788969	-0.732611
20	1	0	-2.583340	0.901417	-2.138508
21	1	0	-5.156788	-2.270918	-1.068038
22	1	0	-5.383689	0.543399	-1.198026
23	1	0	-4.646468	-0.431993	-2.486571
24	1	0	-3.698118	-0.358656	0.409834
25	1	0	-0.888102	-1.625243	1.325689
26	1	0	-1.218832	-2.695830	-0.913016
27	1	0	-4.871424	-2.067780	1.945304
28	8	0	-4.221681	-2.790724	1.976939
29	1	0	-3.399725	-2.420291	1.618947
30	1	0	-1.408892	4.025556	0.635157
31	8	0	-1.603575	4.164757	-0.316462
32	1	0	-1.116692	4.948564	-0.595115
33	8	0	-1.083014	3.250387	2.217391
34	1	0	-0.245408	2.794093	1.999755
35	1	0	-1.750479	2.553308	2.237801
36	1	0	0.878167	1.893874	0.432884
37	8	0	1.299431	2.105874	1.287074
38	1	0	1.544014	1.251028	1.662423
39	8	0	-6.216350	-0.774685	1.640424
40	1	0	-6.147125	-1.031939	0.700355
41	1	0	-7.080996	-1.087974	1.929795
42	6	0	4.088853	-0.424758	-0.794839
43	1	0	2.992392	-0.949895	0.953081
44	1	0	3.981160	-0.148523	-1.841230
45	6	0	5.468109	-0.487732	-0.307805
46	6	0	6.502157	-0.193282	-1.199751
47	6	0	5.794788	-0.832814	1.008446
48	6	0	7.828085	-0.241226	-0.793557
49	1	0	6.256431	0.073816	-2.221114
50	6	0	7.117880	-0.880611	1.413072
51	1	0	5.012387	-1.064906	1.719868
52	6	0	8.139425	-0.585270	0.514521
53	1	0	8.617005	-0.011298	-1.498315
54	1	0	7.357033	-1.148231	2.434584
55	1	0	9.172381	-0.623718	0.836350

Structure 110 (M06-2X/def2-TZVP, Gas Phase)

Energy (Hartrees): = - 1090.095597
No imaginary frequencies

Standard orientation:

Center Number	Atomic Number	Atomic Type	Coordinates (Angstroms) X	Y	Z
1	6	0	2.107099	0.397935	-1.562719
2	6	0	1.294508	-0.186735	-0.394054
3	6	0	2.082830	-0.018567	0.904392
4	6	0	3.462892	-0.617111	0.735146
5	6	0	4.170259	0.046669	-0.440310
6	1	0	2.190601	1.047262	1.132559
7	1	0	3.355100	-1.688977	0.516779
8	1	0	4.292832	1.115144	-0.235463

9	1	0	1.152931	-1.261320	-0.569134
10	8	0	3.385072	-0.150708	-1.608556
11	6	0	5.534933	-0.563246	-0.720924
12	1	0	5.431211	-1.653851	-0.796615
13	1	0	5.882579	-0.192376	-1.685124
14	8	0	6.492752	-0.195148	0.241275
15	1	0	6.118366	-0.368135	1.113306
16	8	0	4.236253	-0.432141	1.904405
17	1	0	3.705686	-0.730782	2.652310
18	8	0	1.464290	-0.697417	1.982990
19	1	0	0.680691	-0.209180	2.254653
20	7	0	0.040056	0.520592	-0.287083
21	6	0	-2.341700	0.473404	0.061013
22	6	0	-2.409269	1.807432	0.385269
23	6	0	-3.543853	-0.289250	-0.072139
24	6	0	-3.641344	2.447105	0.597110
25	1	0	-1.484216	2.360437	0.482903
26	6	0	-4.786647	0.364554	0.151623
27	6	0	-4.804314	1.738044	0.486488
28	1	0	-3.662026	3.497974	0.854076
29	1	0	-5.761659	2.217700	0.652663
30	6	0	-1.019842	-0.153659	-0.121769
31	1	0	-0.970778	-1.245479	-0.090602
32	1	0	1.641457	0.120151	-2.513304
33	8	0	2.198301	1.791334	-1.446323
34	1	0	1.343330	2.103910	-1.121464
35	6	0	-3.571436	-1.660915	-0.435182
36	6	0	-5.993760	-0.365937	0.027496
37	6	0	-5.982119	-1.686824	-0.311907
38	1	0	-6.909481	-2.236462	-0.405091
39	6	0	-4.753474	-2.337216	-0.549361
40	1	0	-4.747646	-3.382911	-0.828917
41	1	0	-2.651563	-2.188768	-0.640368
42	1	0	-6.929733	0.150068	0.205065

Structure 110 (M06-2X/def2-TZVP, DMSO)

Energy (Hartrees): = - 1090.1247119
No imaginary frequencies

Standard orientation:

Center Number	Atomic Number	Atomic Type	Coordinates (Angstroms) X	Y	Z
1	6	0	2.025340	-0.653561	-1.449726
2	6	0	1.286060	-0.421833	-0.122561
3	6	0	2.117928	0.483365	0.786694
4	6	0	3.515552	-0.080482	0.922598
5	6	0	4.143548	-0.229641	-0.457714
6	1	0	2.185670	1.484111	0.345362
7	1	0	3.456476	-1.071433	1.392059
8	1	0	4.215024	0.755998	-0.930242
9	1	0	1.153380	-1.389519	0.375373
10	8	0	3.332782	-1.095978	-1.240650
11	6	0	5.528288	-0.854538	-0.407103
12	1	0	5.485383	-1.785872	0.171431
13	1	0	5.834692	-1.103115	-1.424162
14	8	0	6.495201	0.032639	0.117625
15	1	0	6.134967	0.392556	0.938792
16	8	0	4.332935	0.779838	1.699652
17	1	0	3.863937	0.963321	2.524158
18	8	0	1.570168	0.559454	2.090236
19	1	0	0.772929	1.103479	2.063124
20	7	0	0.027981	0.231477	-0.406638
21	6	0	-2.329368	0.458336	-0.035938
22	6	0	-2.361303	1.805368	-0.308266
23	6	0	-3.551760	-0.270586	0.097054
24	6	0	-3.579711	2.492349	-0.458066
25	1	0	-1.425545	2.344089	-0.391416
26	6	0	-4.779001	0.431780	-0.053019
27	6	0	-4.763012	1.820053	-0.329701
28	1	0	-3.572037	3.554668	-0.666267
29	1	0	-5.709620	2.337123	-0.437463
30	6	0	-1.024736	-0.208145	0.144941
31	1	0	-0.988555	-1.088619	0.791741
32	1	0	1.537002	-1.460231	-2.003481
33	8	0	2.049318	0.520502	-2.219578
34	1	0	1.233127	1.003019	-2.020133
35	6	0	-3.603954	-1.667197	0.344578
36	6	0	-6.004373	-0.269777	0.067834
37	6	0	-6.019339	-1.612264	0.319079
38	1	0	-6.959537	-2.141681	0.409466
39	6	0	-4.803116	-2.316002	0.455002
40	1	0	-4.821149	-3.381936	0.645150
41	1	0	-2.688044	-2.234557	0.438266

42	1	0	-6.929454	0.283285	-0.047676

Structure 110.5H₂O (M06-2X/def2-TZVP, Gas Phase)

Energy (Hartrees): = - 1472.3185026
No imaginary frequencies

Standard orientation:

Center Number	Atomic Number	Atomic Type	Coordinates (Angstroms) X	Y	Z
1	6	0	4.091439	-0.344321	-0.310813
2	6	0	2.932217	-1.172884	-0.227783
3	6	0	2.993594	-2.382316	0.420494
4	6	0	4.196043	-2.838500	0.990750
5	6	0	5.326877	-2.076602	0.899670
6	6	0	5.304206	-0.816985	0.256048
7	6	0	1.670607	-0.753908	-0.864760
8	7	0	0.540193	-1.083942	-0.401168
9	6	0	-0.645445	-0.587699	-1.071384
10	6	0	-1.061856	0.722294	-0.402977
11	6	0	-2.452099	1.168267	-0.836563
12	6	0	-3.428951	0.015269	-0.642216
13	8	0	-2.977393	-1.128917	-1.360951
14	6	0	-1.752805	-1.638929	-0.953025
15	8	0	-0.049501	1.662942	-0.717339
16	8	0	-1.856808	-2.100061	0.373079
17	6	0	-4.814113	0.322834	-1.166820
18	8	0	-5.768034	-0.624066	-0.734475
19	8	0	-2.909088	2.233080	-0.026665
20	1	0	1.743293	-0.140869	-1.767032
21	1	0	-0.469598	-0.382922	-2.134045
22	1	0	-0.336913	2.573920	-0.518034
23	1	0	-1.100812	0.558002	0.682441
24	1	0	-2.470879	3.060583	-0.272875
25	1	0	-2.429890	1.455621	-1.896264
26	1	0	-5.354309	-1.507046	-0.678155
27	1	0	-5.125624	1.299765	-0.797765
28	1	0	-4.770656	0.354883	-2.261812
29	1	0	-3.494207	-0.213906	0.428424
30	1	0	-0.963818	-2.108667	0.745980
31	1	0	-1.547614	-2.476115	-1.625696
32	1	0	4.222502	-3.800262	1.485694
33	1	0	2.102069	-2.994914	0.462856
34	1	0	-5.001220	-2.383618	1.338870
35	8	0	-4.690066	-2.869189	0.559637
36	1	0	-3.726034	-2.807010	0.585037
37	1	0	-0.894403	3.936269	1.014166
38	8	0	-0.931364	4.190537	0.060301
39	1	0	-0.581859	5.077557	-0.044765
40	8	0	-1.039735	2.876867	2.351633
41	1	0	-0.289207	2.255109	2.377045
42	1	0	-1.818941	2.366132	2.096443
43	1	0	1.052372	1.391217	0.824543
44	8	0	1.228217	1.319465	1.778095
45	1	0	1.510481	0.410821	1.921255
46	8	0	-5.768817	-0.699651	2.026505
47	1	0	-5.985865	-0.512818	1.094041
48	1	0	-6.566002	-0.544485	2.537113
49	1	0	6.261192	-2.425447	1.323445
50	6	0	4.079565	0.944024	-0.902566
51	6	0	6.465965	-0.011171	0.179994
52	6	0	5.217324	1.699407	-0.956267
53	6	0	6.428036	1.215793	-0.416830
54	1	0	3.156728	1.349230	-1.297067
55	1	0	5.188673	2.682262	-1.408466
56	1	0	7.320350	1.825928	-0.467056
57	1	0	7.385926	-0.387136	0.611587

Structure 110.5H₂O (M06-2X/def2-TZVP, DMSO)

Energy (Hartrees): = - 1472.3571281
No imaginary frequencies

Standard orientation:

Center Number	Atomic Number	Atomic Type	Coordinates (Angstroms) X	Y	Z
1	6	0	4.187245	-0.384315	-0.370189
2	6	0	2.949703	-1.052506	-0.115401
3	6	0	2.907541	-2.111198	0.760468
4	6	0	4.069364	-2.566803	1.409915
5	6	0	5.268490	-1.953994	1.173869

6	6	0	5.356857	-0.853902	0.287414
7	6	0	1.711467	-0.630514	-0.797913
8	7	0	0.570882	-0.822501	-0.281528
9	6	0	-0.602386	-0.373111	-1.003492
10	6	0	-1.135220	0.882238	-0.316586
11	6	0	-2.530560	1.254360	-0.801910
12	6	0	-3.431946	0.033180	-0.683189
13	8	0	-2.883879	-1.056602	-1.419508
14	6	0	-1.639899	-1.499603	-0.981160
15	8	0	-0.177731	1.901587	-0.553597
16	8	0	-1.776875	-2.039090	0.315724
17	6	0	-4.815293	0.257293	-1.246125
18	8	0	-5.677758	-0.830272	-0.944686
19	8	0	-3.096283	2.260431	0.018357
20	1	0	1.807296	-0.153211	-1.775361
21	1	0	-0.384853	-0.130823	-2.049813
22	1	0	-0.533507	2.776649	-0.310072
23	1	0	-1.213969	0.678373	0.760678
24	1	0	-2.659392	3.109744	-0.144645
25	1	0	-2.484429	1.577307	-1.849598
26	1	0	-5.158723	-1.654191	-0.939035
27	1	0	-5.249639	1.154892	-0.807550
28	1	0	-4.741424	0.397236	-2.329986
29	1	0	-3.513738	-0.237498	0.376878
30	1	0	-0.917447	-1.960040	0.758107
31	1	0	-1.343819	-2.284951	-1.680613
32	1	0	4.007865	-3.409226	2.086955
33	1	0	1.962294	-2.611434	0.932099
34	1	0	-4.935630	-2.669249	1.038711
35	8	0	-4.474336	-3.124650	0.316038
36	1	0	-3.546004	-2.858395	0.392360
37	1	0	-1.158940	4.059698	1.261872
38	8	0	-1.249177	4.326618	0.322650
39	1	0	-0.920788	5.226446	0.219673
40	8	0	-1.164473	2.842418	2.574392
41	1	0	-0.370584	2.280652	2.481633
42	1	0	-1.910288	2.294288	2.295946
43	1	0	0.954786	1.580965	0.927167
44	8	0	1.176082	1.444753	1.866388
45	1	0	1.273537	0.487424	1.950884
46	8	0	-5.997098	-1.229296	1.786156
47	1	0	-6.089047	-0.927294	0.863660
48	1	0	-6.889590	-1.311733	2.138583
49	1	0	6.172708	-2.299066	1.662212
50	6	0	4.303641	0.743177	-1.224492
51	6	0	6.595686	-0.205851	0.055945
52	6	0	5.513822	1.348875	-1.424549
53	6	0	6.676011	0.869297	-0.782585
54	1	0	3.428478	1.145151	-1.717224
55	1	0	5.581111	2.208950	-2.079118
56	1	0	7.625748	1.360601	-0.952914
57	1	0	7.476915	-0.580682	0.563761

Structure 110.5H_2O (M06-2X/def2-TZVP, H_2O)

Energy (Hartrees): = - 1472.3736162
No imaginary frequencies

Standard orientation:

Center Number	Atomic Number	Atomic Type	Coordinates (Angstroms) X	Y	Z
1	6	0	4.058771	-0.386411	-0.367303
2	6	0	2.914215	-1.231606	-0.239986
3	6	0	3.018826	-2.436263	0.411395
4	6	0	4.246371	-2.865529	0.952899
5	6	0	5.359669	-2.082110	0.832845
6	6	0	5.293834	-0.828366	0.177522
7	6	0	1.626905	-0.827040	-0.836719
8	7	0	0.514348	-1.072295	-0.278328
9	6	0	-0.684803	-0.617651	-0.955937
10	6	0	-1.129400	0.716768	-0.362583
11	6	0	-2.501077	1.109167	-0.898836
12	6	0	-3.493964	-0.030167	-0.704550
13	8	0	-3.000504	-1.237768	-1.280973
14	6	0	-1.768902	-1.678713	-0.782439
15	8	0	-0.136138	1.667781	-0.715139
16	8	0	-1.922976	-2.030635	0.575184
17	6	0	-4.805241	0.257027	-1.400421
18	8	0	-5.778678	-0.743517	-1.129232
19	8	0	-3.036145	2.221023	-0.202842
20	1	0	1.667073	-0.315264	-1.801706
21	1	0	-0.523494	-0.475193	-2.030126
22	1	0	-0.376386	2.548444	-0.360346
23	1	0	-1.199258	0.630252	0.729484

24	1	0	-2.434591	2.979606	-0.266116
25	1	0	-2.406525	1.328835	-1.970101
26	1	0	-5.383241	-1.607907	-1.305854
27	1	0	-5.213513	1.199751	-1.040979
28	1	0	-4.629511	0.333445	-2.477859
29	1	0	-3.668278	-0.176111	0.368120
30	1	0	-1.064466	-1.927214	1.013206
31	1	0	-1.519265	-2.564383	-1.369269
32	1	0	4.302709	-3.823934	1.452540
33	1	0	2.148446	-3.075440	0.485511
34	1	0	-5.045726	-1.884992	1.611142
35	8	0	-4.537811	-2.704889	1.489845
36	1	0	-3.655286	-2.423887	1.201111
37	1	0	-0.834815	3.771602	1.264989
38	8	0	-0.994945	4.091382	0.351423
39	1	0	-0.406780	4.841036	0.207017
40	8	0	-0.574001	2.718690	2.695765
41	1	0	0.225533	2.260464	2.368737
42	1	0	-1.282950	2.066928	2.630747
43	1	0	1.232859	1.527476	0.606110
44	8	0	1.701760	1.693606	1.444695
45	1	0	2.151115	0.867022	1.659397
46	8	0	-6.215801	-0.378798	1.594090
47	1	0	-6.205287	-0.496309	0.624506
48	1	0	-7.091412	-0.665521	1.878001
49	1	0	6.311339	-2.406017	1.237694
50	6	0	4.010739	0.889747	-0.985946
51	6	0	6.438820	-0.001089	0.067807
52	6	0	5.133774	1.665910	-1.075412
53	6	0	6.363805	1.217201	-0.546276
54	1	0	3.075162	1.265377	-1.380839
55	1	0	5.078695	2.637535	-1.549802
56	1	0	7.241984	1.845523	-0.623007
57	1	0	7.373277	-0.355301	0.487116

Structure 110b (M06-2X/def2-TZVP, Gas Phase)

Energy (Hartrees): = - 1090.0961461
No imaginary frequencies

Standard orientation:

Center Number	Atomic Number	Atomic Type	Coordinates (Angstroms) X	Y	Z
1	6	0	1.890152	0.148563	-1.589475
2	6	0	1.227679	-0.628380	-0.437607
3	6	0	1.916809	-0.271215	0.877423
4	6	0	3.407713	-0.494538	0.743677
5	6	0	3.946058	0.335239	-0.415483
6	1	0	1.742838	0.786007	1.105513
7	1	0	3.586110	-1.556769	0.524737
8	1	0	3.781776	1.397841	-0.209569
9	1	0	1.369867	-1.702392	-0.617701
10	8	0	3.266572	-0.053792	-1.602218
11	6	0	5.428572	0.102905	-0.662859
12	1	0	5.614586	-0.976608	-0.739448
13	1	0	5.689579	0.556572	-1.619131
14	8	0	6.234261	0.703063	0.321873
15	1	0	5.894473	0.438413	1.185054
16	8	0	4.079078	-0.120909	1.930736
17	1	0	3.617871	-0.538521	2.667666
18	8	0	1.466156	-1.088553	1.942889
19	1	0	0.570224	-0.827830	2.179102
20	7	0	-0.169308	-0.268255	-0.354921
21	6	0	-2.484474	-1.065768	-0.204929
22	6	0	-3.185627	-2.239788	-0.363952
23	6	0	-3.199367	0.156164	0.025695
24	6	0	-4.590428	-2.282551	-0.333890
25	1	0	-2.636103	-3.159632	-0.526753
26	6	0	-4.620370	0.103304	0.058066
27	6	0	-5.290414	-1.130130	-0.131084
28	1	0	-5.102788	-3.224985	-0.472517
29	1	0	-6.373616	-1.138595	-0.105633
30	6	0	-1.022561	-1.203832	-0.300243
31	1	0	-0.686437	-2.248329	-0.343718
32	1	0	1.533132	-0.237372	-2.548993
33	8	0	1.622310	1.520347	-1.478988
34	1	0	0.701417	1.609430	-1.201471
35	6	0	-2.576651	1.410037	0.242704
36	6	0	-5.360720	1.286914	0.286313
37	6	0	-4.729442	2.481128	0.484162
38	1	0	-5.302775	3.381994	0.659666
39	6	0	-3.322757	2.536090	0.465745
40	1	0	-2.824152	3.482652	0.631045
41	1	0	-1.500726	1.466075	0.232800

42	1	0	-6.442159	1.223734	0.303882

Structure 110b (M06-2X/def2-TZVP, DMSO)

Energy (Hartrees): = - 1090.1247689
No imaginary frequencies

Standard orientation:

Center Number	Atomic Number	Atomic Type	Coordinates (Angstroms) X	Y	Z
1	6	0	1.863400	0.502597	-1.495084
2	6	0	1.232285	-0.521045	-0.539202
3	6	0	1.940678	-0.474578	0.813870
4	6	0	3.430415	-0.637432	0.606602
5	6	0	3.941144	0.445157	-0.336061
6	1	0	1.756806	0.492344	1.295354
7	1	0	3.620189	-1.618685	0.151650
8	1	0	3.774604	1.428061	0.117957
9	1	0	1.370980	-1.520989	-0.967995
10	8	0	3.248110	0.346190	-1.573455
11	6	0	5.418274	0.288780	-0.658709
12	1	0	5.600238	-0.731402	-1.019397
13	1	0	5.673508	0.981484	-1.461931
14	8	0	6.245214	0.600195	0.444079
15	1	0	5.891699	0.126325	1.208178
16	8	0	4.125750	-0.528857	1.838465
17	1	0	3.701590	-1.128441	2.466306
18	8	0	1.521443	-1.530153	1.660318
19	1	0	0.631471	-1.337508	1.980433
20	7	0	-0.164883	-0.190250	-0.360740
21	6	0	-2.471670	-1.026249	-0.326552
22	6	0	-3.154542	-2.195455	-0.580741
23	6	0	-3.206806	0.154412	0.030545
24	6	0	-4.557650	-2.274127	-0.512752
25	1	0	-2.591669	-3.082810	-0.846347
26	6	0	-4.626108	0.065489	0.092880
27	6	0	-5.277255	-1.162548	-0.185412
28	1	0	-5.053553	-3.213099	-0.721926
29	1	0	-6.359289	-1.197112	-0.129854
30	6	0	-1.010019	-1.130137	-0.471010
31	1	0	-0.669764	-2.143685	-0.713006
32	1	0	1.498122	0.326428	-2.510844
33	8	0	1.568051	1.815574	-1.092377
34	1	0	0.683920	1.796301	-0.697497
35	6	0	-2.608698	1.403581	0.333544
36	6	0	-5.388651	1.208176	0.435528
37	6	0	-4.779741	2.399586	0.713838
38	1	0	-5.369018	3.269711	0.974804
39	6	0	-3.374881	2.490649	0.664161
40	1	0	-2.892507	3.433608	0.890978
41	1	0	-1.534886	1.492849	0.306717
42	1	0	-6.467840	1.113662	0.472680

Structure 111 (M06-2X/def2-TZVP, Gas Phase)

Energy (Hartrees): = - 1243.7337955
No imaginary frequencies

Standard orientation:

Center Number	Atomic Number	Atomic Type	Coordinates (Angstroms) X	Y	Z
1	6	0	-2.834355	-1.339132	-1.022922
2	6	0	-2.090662	-0.058182	-0.606207
3	6	0	-2.786565	0.559306	0.605322
4	6	0	-4.254211	0.760149	0.295825
5	6	0	-4.880333	-0.575553	-0.087017
6	1	0	-2.701942	-0.117747	1.461914
7	1	0	-4.343748	1.446077	-0.558388
8	1	0	-4.802744	-1.268627	0.756886
9	1	0	-2.140992	0.661808	-1.433141
10	8	0	-4.190542	-1.089744	-1.218507
11	6	0	-6.342505	-0.444422	-0.484420
12	1	0	-6.440137	0.345207	-1.241085
13	1	0	-6.657776	-1.384041	-0.938348
14	8	0	-7.177441	-0.211703	0.623664
15	1	0	-6.804009	0.521317	1.127465
16	8	0	-4.935079	1.285660	1.417881
17	1	0	-4.430397	2.044767	1.732574
18	8	0	-2.245106	1.829549	0.925301
19	1	0	-1.400588	1.705870	1.370101
20	7	0	-0.731514	-0.389802	-0.246298

21	6	0	1.618677	0.109212	-0.349897
22	6	0	2.572401	1.196010	-0.304657
23	6	0	2.029871	-1.166134	-0.148970
24	6	0	3.939493	0.910333	-0.076798
25	6	0	3.396607	-1.491159	0.097449
26	1	0	1.302681	-1.968029	-0.196164
27	6	0	4.364694	-0.465194	0.126849
28	6	0	0.198867	0.365207	-0.655667
29	1	0	-0.031701	1.231354	-1.283629
30	1	0	-2.465610	-1.685862	-1.992709
31	8	0	-2.672809	-2.335360	-0.050597
32	1	0	-1.784688	-2.232983	0.317237
33	6	0	3.782243	-2.830822	0.296962
34	6	0	5.704009	-0.829961	0.359879
35	6	0	6.060764	-2.142519	0.554569
36	6	0	5.093896	-3.156915	0.524398
37	1	0	5.383441	-4.187887	0.678994
38	1	0	3.019726	-3.599997	0.266635
39	1	0	7.098340	-2.393359	0.733406
40	1	0	6.476858	-0.075939	0.390872
41	6	0	2.170769	2.539307	-0.451689
42	6	0	4.849734	1.983090	-0.040118
43	6	0	4.437175	3.281873	-0.207147
44	1	0	5.160019	4.086438	-0.173514
45	6	0	3.082118	3.564561	-0.407832
46	1	0	2.750993	4.588173	-0.523744
47	1	0	1.125024	2.778349	-0.588075
48	1	0	5.899527	1.792174	0.127387

Structure 111 (M06-2X/def2-TZVP, DMSO)

Energy (Hartrees): = - 1243.7661935
No imaginary frequencies

Standard orientation:

Center Number	Atomic Number	Atomic Type	Coordinates (Angstroms) X	Y	Z
1	6	0	-2.803727	-1.383544	-0.949740
2	6	0	-2.090845	-0.060101	-0.630063
3	6	0	-2.793405	0.648861	0.528552
4	6	0	-4.271025	0.771520	0.225792
5	6	0	-4.856455	-0.610974	-0.033336
6	1	0	-2.671789	0.064487	1.447655
7	1	0	-4.400892	1.382210	-0.677664
8	1	0	-4.736053	-1.228762	0.863325
9	1	0	-2.142181	0.585838	-1.514001
10	8	0	-4.175210	-1.198272	-1.134061
11	6	0	-6.329397	-0.561347	-0.406174
12	1	0	-6.472573	0.158650	-1.221577
13	1	0	-6.630869	-1.545531	-0.767343
14	8	0	-7.149714	-0.251066	0.702186
15	1	0	-6.772144	0.532160	1.123227
16	8	0	-4.963122	1.358224	1.315655
17	1	0	-4.508991	2.180189	1.542294
18	8	0	-2.295263	1.961822	0.714061
19	1	0	-1.427395	1.911929	1.134106
20	7	0	-0.728628	-0.355058	-0.241456
21	6	0	1.619294	0.102058	-0.390188
22	6	0	2.566775	1.192233	-0.306183
23	6	0	2.032225	-1.175890	-0.208207
24	6	0	3.930389	0.911347	-0.049757
25	6	0	3.397460	-1.497235	0.061690
26	1	0	1.317209	-1.986925	-0.288186
27	6	0	4.358780	-0.466396	0.140708
28	6	0	0.202327	0.358917	-0.718285
29	1	0	-0.016669	1.173008	-1.414901
30	1	0	-2.439227	-1.774865	-1.903489
31	8	0	-2.594189	-2.323045	0.072452
32	1	0	-1.723414	-2.134252	0.452747
33	6	0	3.785243	-2.839941	0.240595
34	6	0	5.694753	-0.826447	0.405603
35	6	0	6.053546	-2.142789	0.579712
36	6	0	5.093770	-3.162592	0.497348
37	1	0	5.386647	-4.195684	0.635628
38	1	0	3.027351	-3.611787	0.171028
39	1	0	7.087490	-2.392087	0.783847
40	1	0	6.461574	-0.068167	0.479741
41	6	0	2.156350	2.534313	-0.445083
42	6	0	4.834489	1.988987	0.024900
43	6	0	4.413849	3.287714	-0.133521
44	1	0	5.130306	4.097210	-0.071183
45	6	0	3.060893	3.564733	-0.364205
46	1	0	2.726429	4.588605	-0.474055
47	1	0	1.111237	2.763556	-0.607201

48	1	0	5.882603	1.803768	0.212903

Structure 111.5H_2O (M06-2X/def2-TZVP, Gas Phase)

Energy (Hartrees): = - 1625.9566386
No imaginary frequencies

Standard orientation:

Center Number	Atomic Number	Atomic Type	Coordinates (Angstroms) X	Y	Z
1	6	0	3.346000	0.825308	-0.618022
2	6	0	2.371956	-0.240632	-0.687810
3	6	0	2.708087	-1.500253	-0.318917
4	6	0	4.025444	-1.830698	0.120110
5	6	0	5.017227	-0.827813	0.170847
6	6	0	4.665132	0.532991	-0.201112
7	6	0	1.012714	0.026693	-1.194934
8	7	0	-0.002174	-0.574838	-0.737850
9	6	0	-1.307169	-0.215115	-1.257110
10	6	0	-1.889974	0.870780	-0.353189
11	6	0	-3.370563	1.107668	-0.621078
12	6	0	-4.101451	-0.228031	-0.562543
13	8	0	-3.524063	-1.135922	-1.496760
14	6	0	-2.195944	-1.463050	-1.261073
15	8	0	-1.090058	2.022402	-0.557998
16	8	0	-2.092246	-2.146093	-0.034793
17	6	0	-5.565462	-0.117529	-0.928428
18	8	0	-6.290081	-1.277750	-0.578705
19	8	0	-3.933383	1.928312	0.382933
20	1	0	0.908633	0.770067	-1.989976
21	1	0	-1.257418	0.184276	-2.276873
22	1	0	-1.513608	2.820197	-0.188571
23	1	0	-1.803973	0.532360	0.688335
24	1	0	-3.669939	2.851048	0.254142
25	1	0	-3.498134	1.553822	-1.616363
26	1	0	-5.729046	-2.067603	-0.701897
27	1	0	-6.004227	0.721519	-0.388858
28	1	0	-5.640358	0.075356	-2.005071
29	1	0	-4.016405	-0.630362	0.454387
30	1	0	-1.184102	-2.034009	0.280748
31	1	0	-1.909164	-2.124105	-2.083471
32	1	0	1.965995	-2.287323	-0.385601
33	1	0	-5.005593	-3.155811	1.100878
34	8	0	-4.707438	-3.450723	0.227019
35	1	0	-3.772428	-3.210290	0.180427
36	1	0	-2.170537	3.799352	1.578873
37	8	0	-2.331599	4.185390	0.683977
38	1	0	-2.151201	5.127120	0.708169
39	8	0	-2.017656	2.535546	2.724733
40	1	0	-1.171112	2.067320	2.604177
41	1	0	-2.714234	1.935732	2.428032
42	1	0	0.172218	1.715445	0.859105
43	8	0	0.432084	1.530419	1.777340
44	1	0	0.873301	0.675550	1.753055
45	8	0	-5.941386	-1.743451	2.121012
46	1	0	-6.304135	-1.470541	1.257763
47	1	0	-6.674099	-1.772243	2.739542
48	6	0	2.996718	2.157327	-0.918827
49	6	0	5.594709	1.588075	-0.143436
50	6	0	3.926021	3.164742	-0.851174
51	6	0	5.239778	2.874149	-0.467722
52	1	0	1.975797	2.398989	-1.186263
53	1	0	3.640736	4.182118	-1.084585
54	1	0	5.975443	3.665893	-0.412042
55	1	0	6.610860	1.395200	0.168055
56	6	0	6.307990	-1.199115	0.591461
57	1	0	7.096477	-0.462626	0.643361
58	6	0	4.341120	-3.153906	0.483945
59	6	0	6.596233	-2.494981	0.945701
60	6	0	5.606324	-3.485878	0.893984
61	1	0	7.597547	-2.751833	1.266226
62	1	0	3.562849	-3.905949	0.431819
63	1	0	5.842344	-4.504246	1.173345

Structure 111.5H_2O (M06-2X/def2-TZVP, DMSO)

Energy (Hartrees): = - 1625.9985016
No imaginary frequencies

Standard orientation:

Center Number	Atomic Number	Atomic Type	Coordinates (Angstroms) X	Y	Z

1	6	0	3.431762	0.815150	-0.695825
2	6	0	2.390061	-0.189798	-0.688443
3	6	0	2.655113	-1.445034	-0.250862
4	6	0	3.957320	-1.829735	0.192463
5	6	0	5.009566	-0.888900	0.179076
6	6	0	4.735456	0.468398	-0.267756
7	6	0	1.036338	0.131493	-1.183689
8	7	0	0.004758	-0.398641	-0.675914
9	6	0	-1.293996	-0.022870	-1.199595
10	6	0	-1.917420	0.975536	-0.226792
11	6	0	-3.396727	1.205123	-0.500474
12	6	0	-4.096256	-0.145960	-0.551615
13	8	0	-3.491478	-0.970659	-1.544427
14	6	0	-2.153337	-1.284845	-1.321168
15	8	0	-1.145824	2.160810	-0.323136
16	8	0	-2.057345	-2.077302	-0.157923
17	6	0	-5.556735	-0.047838	-0.923005
18	8	0	-6.216461	-1.295363	-0.765148
19	8	0	-3.985283	1.940581	0.560542
20	1	0	0.952758	0.834035	-2.016421
21	1	0	-1.228717	0.447283	-2.186779
22	1	0	-1.618334	2.920863	0.064029
23	1	0	-1.842333	0.553999	0.785713
24	1	0	-3.771480	2.881220	0.467617
25	1	0	-3.526406	1.724827	-1.457397
26	1	0	-5.589691	-2.013677	-0.964051
27	1	0	-6.050939	0.671997	-0.271384
28	1	0	-5.640457	0.300850	-1.957918
29	1	0	-4.006046	-0.621527	0.433254
30	1	0	-1.170635	-1.947082	0.213060
31	1	0	-1.839903	-1.864256	-2.192458
32	1	0	1.870103	-2.193177	-0.257826
33	1	0	-4.980671	-3.309026	0.744214
34	8	0	-4.559655	-3.547121	-0.097279
35	1	0	-3.671481	-3.161701	-0.062909
36	1	0	-2.285045	3.905987	1.826764
37	8	0	-2.504929	4.261164	0.941800
38	1	0	-2.332455	5.209139	0.933857
39	8	0	-2.059344	2.438368	2.892159
40	1	0	-1.202151	2.016387	2.695609
41	1	0	-2.726895	1.901712	2.441629
42	1	0	0.143164	1.783167	1.016136
43	8	0	0.440240	1.511827	1.903234
44	1	0	0.674576	0.580453	1.802052
45	8	0	-6.148451	-2.229547	1.854985
46	1	0	-6.393990	-1.785908	1.022401
47	1	0	-6.969530	-2.534420	2.255560
48	6	0	3.167171	2.145036	-1.083286
49	6	0	5.730894	1.465046	-0.273843
50	6	0	4.157804	3.096408	-1.075741
51	6	0	5.453112	2.750358	-0.672698
52	1	0	2.167146	2.432178	-1.380472
53	1	0	3.934621	4.112820	-1.374879
54	1	0	6.236617	3.497855	-0.665688
55	1	0	6.735845	1.227714	0.045599
56	6	0	6.281643	-1.311894	0.611571
57	1	0	7.114990	-0.623793	0.615528
58	6	0	4.194872	-3.148749	0.627566
59	6	0	6.493106	-2.602952	1.035877
60	6	0	5.443676	-3.533484	1.046315
61	1	0	7.480554	-2.903022	1.363729
62	1	0	3.370016	-3.851868	0.624263
63	1	0	5.621153	-4.547545	1.381651

Structure 111.5H$_2$O (M06-2X/def2-TZVP, H$_2$O)

Energy (Hartrees): = - 1626.0135286
No imaginary frequencies

Standard orientation:

Center Number	Atomic Number	Atomic Type	Coordinates (Angstroms) X	Y	Z
1	6	0	3.324851	0.776786	-0.668588
2	6	0	2.366309	-0.307486	-0.665055
3	6	0	2.742382	-1.549203	-0.274741
4	6	0	4.085076	-1.840479	0.118887
5	6	0	5.058839	-0.819060	0.105269
6	6	0	4.664194	0.524560	-0.288284
7	6	0	0.985837	-0.068285	-1.130491
8	7	0	-0.020242	-0.611979	-0.581434
9	6	0	-1.325750	-0.278481	-1.118885
10	6	0	-1.945060	0.837164	-0.280335
11	6	0	-3.391772	1.071508	-0.694598
12	6	0	-4.169169	-0.238598	-0.659101

13	8	0	-3.523233	-1.231956	-1.453220
14	6	0	-2.204098	-1.527487	-1.087729
15	8	0	-1.148034	1.993372	-0.483247
16	8	0	-2.204118	-2.119471	0.192790
17	6	0	-5.554548	-0.075601	-1.241921
18	8	0	-6.331727	-1.258322	-1.099995
19	8	0	-4.053588	1.951616	0.196197
20	1	0	0.861773	0.594088	-1.991242
21	1	0	-1.268321	0.066617	-2.156913
22	1	0	-1.507005	2.739617	0.040594
23	1	0	-1.931614	0.551647	0.779446
24	1	0	-3.576023	2.794229	0.249951
25	1	0	-3.402296	1.472069	-1.716396
26	1	0	-5.823767	-2.002191	-1.450995
27	1	0	-6.085890	0.714250	-0.714475
28	1	0	-5.469135	0.198154	-2.298029
29	1	0	-4.241446	-0.584505	0.378663
30	1	0	-1.362424	-1.913155	0.626029
31	1	0	-1.855305	-2.248411	-1.829125
32	1	0	2.024968	-2.361154	-0.284543
33	1	0	-5.230286	-2.678378	1.389424
34	8	0	-4.602444	-3.366604	1.111578
35	1	0	-3.803279	-2.891609	0.834327
36	1	0	-2.058390	3.545348	1.890552
37	8	0	-2.296483	4.004116	1.056844
38	1	0	-1.816024	4.839598	1.047988
39	8	0	-1.615296	2.274799	3.085393
40	1	0	-0.779039	1.998219	2.660823
41	1	0	-2.247056	1.573355	2.884957
42	1	0	0.284920	1.818316	0.779505
43	8	0	0.741542	1.878441	1.638598
44	1	0	1.312838	1.101718	1.682956
45	8	0	-6.588432	-1.372959	1.663389
46	1	0	-6.647403	-1.358029	0.688504
47	1	0	-7.404026	-1.788377	1.965845
48	6	0	2.941455	2.090972	-1.008181
49	6	0	5.575896	1.598180	-0.289762
50	6	0	3.853542	3.117958	-1.001418
51	6	0	5.184065	2.866857	-0.643755
52	1	0	1.910981	2.301893	-1.264764
53	1	0	3.542687	4.120546	-1.265619
54	1	0	5.904020	3.675176	-0.636195
55	1	0	6.604830	1.433151	-0.002872
56	6	0	6.375161	-1.152890	0.479334
57	1	0	7.150569	-0.399905	0.475072
58	6	0	4.440266	-3.148634	0.502526
59	6	0	6.702523	-2.435463	0.852771
60	6	0	5.729185	-3.445554	0.867586
61	1	0	7.721900	-2.666556	1.134948
62	1	0	3.673437	-3.914529	0.501993
63	1	0	5.996906	-4.452169	1.161834

Structure 111b (M06-2X/def2-TZVP, Gas Phase)

Energy (Hartrees): = - 1243.7343277
No imaginary frequencies

Standard orientation:

Center Number	Atomic Number	Atomic Type	Coordinates (Angstroms) X	Y	Z
1	6	0	2.797984	0.107551	-1.584589
2	6	0	2.048496	-0.554626	-0.414548
3	6	0	2.801218	-0.285034	0.886753
4	6	0	4.243310	-0.719140	0.735955
5	6	0	4.877278	0.012099	-0.441067
6	1	0	2.782605	0.788387	1.104499
7	1	0	4.265827	-1.798406	0.529343
8	1	0	4.870082	1.089445	-0.247011
9	1	0	2.032475	-1.639801	-0.582423
10	8	0	4.131099	-0.288563	-1.613335
11	6	0	6.307210	-0.433055	-0.705452
12	1	0	6.335082	-1.528666	-0.774274
13	1	0	6.618206	-0.029485	-1.669173
14	8	0	7.204553	0.052096	0.263451
15	1	0	6.842071	-0.153819	1.133367
16	8	0	4.977212	-0.431423	1.909928
17	1	0	4.472934	-0.774165	2.657049
18	8	0	2.256145	-1.019274	1.968543
19	1	0	1.411998	-0.629409	2.216679
20	7	0	0.718886	0.003670	-0.319306
21	6	0	-1.685775	-0.460223	-0.106184
22	6	0	-2.239688	0.871658	0.063493
23	6	0	-2.524391	-1.527584	-0.183934
24	6	0	-3.647369	1.029276	0.114023

25	6	0	-3.943287	-1.406896	-0.135439
26	1	0	-2.108815	-2.523047	-0.300062
27	6	0	-4.521014	-0.129631	0.005016
28	6	0	-0.257311	-0.798342	-0.220195
29	1	0	-0.072217	-1.880639	-0.234870
30	1	0	2.375983	-0.234098	-2.534605
31	8	0	2.728915	1.504540	-1.487598
32	1	0	1.834589	1.724897	-1.196851
33	6	0	-4.759893	-2.549352	-0.233295
34	6	0	-5.925145	-0.045454	0.038151
35	6	0	-6.708247	-1.170797	-0.060627
36	6	0	-6.125690	-2.438174	-0.196421
37	1	0	-6.750464	-3.318336	-0.270842
38	1	0	-4.288454	-3.519077	-0.340672
39	1	0	-7.785994	-1.075107	-0.032341
40	1	0	-6.412510	0.912558	0.142559
41	6	0	-1.424786	2.012786	0.198294
42	6	0	-4.173042	2.323477	0.279769
43	6	0	-3.357535	3.421351	0.400176
44	1	0	-3.791400	4.404773	0.526758
45	6	0	-1.970375	3.262787	0.364053
46	1	0	-1.322055	4.123492	0.465487
47	1	0	-0.354986	1.890478	0.172572
48	1	0	-5.242253	2.469270	0.318454

Structure 111b (M06-2X/def2-TZVP, DMSO)

Energy (Hartrees): = - 1243.7661832
No imaginary frequencies

Standard orientation:

Center Number	Atomic Number	Atomic Type	Coordinates (Angstroms) X	Y	Z
1	6	0	2.785199	0.120516	-1.583711
2	6	0	2.049332	-0.577037	-0.429065
3	6	0	2.796718	-0.337988	0.882830
4	6	0	4.245978	-0.739906	0.718696
5	6	0	4.866159	0.038063	-0.434706
6	1	0	2.755793	0.726159	1.141395
7	1	0	4.294752	-1.811875	0.485954
8	1	0	4.836548	1.108762	-0.205489
9	1	0	2.035564	-1.655521	-0.628861
10	8	0	4.134140	-0.235250	-1.622352
11	6	0	6.303951	-0.371153	-0.709079
12	1	0	6.353459	-1.461458	-0.821596
13	1	0	6.620972	0.080688	-1.650102
14	8	0	7.189205	0.083742	0.294323
15	1	0	6.804525	-0.162129	1.145693
16	8	0	4.982761	-0.463199	1.899007
17	1	0	4.505118	-0.858106	2.640284
18	8	0	2.264430	-1.118642	1.937395
19	1	0	1.410973	-0.749717	2.196909
20	7	0	0.719498	-0.018108	-0.323880
21	6	0	-1.683264	-0.469305	-0.117156
22	6	0	-2.226831	0.866302	0.068534
23	6	0	-2.529641	-1.529695	-0.209115
24	6	0	-3.634440	1.035073	0.123975
25	6	0	-3.948206	-1.396520	-0.151986
26	1	0	-2.122518	-2.526872	-0.339005
27	6	0	-4.517852	-0.117376	0.010022
28	6	0	-0.257770	-0.821384	-0.235282
29	1	0	-0.082578	-1.902948	-0.265213
30	1	0	2.373131	-0.219011	-2.538048
31	8	0	2.676322	1.516735	-1.475433
32	1	0	1.810795	1.701406	-1.081672
33	6	0	-4.771783	-2.534121	-0.258890
34	6	0	-5.921928	-0.022756	0.060658
35	6	0	-6.711878	-1.144175	-0.047502
36	6	0	-6.137666	-2.413581	-0.208433
37	1	0	-6.769641	-3.288780	-0.289909
38	1	0	-4.304317	-3.504159	-0.382768
39	1	0	-7.789299	-1.043124	-0.006061
40	1	0	-6.404418	0.935849	0.187759
41	6	0	-1.404596	2.002674	0.212755
42	6	0	-4.153224	2.332310	0.297622
43	6	0	-3.329262	3.424568	0.426205
44	1	0	-3.756734	4.410593	0.558826
45	6	0	-1.942348	3.256015	0.389653
46	1	0	-1.287210	4.111291	0.499500
47	1	0	-0.334122	1.879458	0.186506
48	1	0	-5.222028	2.486039	0.333388

Structure 112 (M06-2X/def2-TZVP, Gas Phase)

Energy (Hartrees): = - 1243.7193861
No imaginary frequencies

Standard orientation:

Center Number	Atomic Number	Atomic Type	Coordinates (Angstroms) X	Y	Z
1	6	0	-2.451220	-0.989599	1.394332
2	6	0	-1.677786	-0.695921	0.097947
3	6	0	-2.422991	0.369551	-0.706391
4	6	0	-3.856588	-0.071155	-0.914339
5	6	0	-4.518998	-0.324092	0.434874
6	1	0	-2.426601	1.314165	-0.150957
7	1	0	-3.852824	-1.011985	-1.482634
8	1	0	-4.539912	0.606516	1.010930
9	1	0	-1.637832	-1.611710	-0.506407
10	8	0	-3.775360	-1.320405	1.123766
11	6	0	-5.938531	-0.853080	0.297698
12	1	0	-5.936228	-1.712821	-0.385459
13	1	0	-6.267568	-1.204254	1.275719
14	8	0	-6.841528	0.142184	-0.117966
15	1	0	-6.469084	0.574215	-0.895759
16	8	0	-4.588970	0.917501	-1.610593
17	1	0	-4.077180	1.160908	-2.391049
18	8	0	-1.855120	0.550716	-1.991510
19	1	0	-1.026432	1.033927	-1.910997
20	7	0	-0.362091	-0.201716	0.433382
21	6	0	2.019307	-0.176629	-0.065879
22	6	0	2.347680	1.186331	0.077905
23	6	0	3.032372	-1.152949	-0.139716
24	6	0	3.729754	1.565451	0.122232
25	6	0	4.405015	-0.756149	-0.089127
26	6	0	4.717270	0.591701	0.036027
27	1	0	5.758991	0.892691	0.068666
28	6	0	0.624631	-0.632490	-0.232938
29	1	0	0.468723	-1.394587	-1.005753
30	1	0	-2.029267	-1.871933	1.885565
31	8	0	-2.410220	0.119631	2.249349
32	1	0	-1.517832	0.487559	2.194295
33	6	0	2.753421	-2.552861	-0.228708
34	6	0	5.428277	-1.747659	-0.163474
35	6	0	5.117866	-3.064316	-0.268572
36	1	0	5.899255	-3.810713	-0.324157
37	6	0	3.755783	-3.468694	-0.294010
38	1	0	3.516556	-4.522443	-0.356638
39	1	0	1.729676	-2.899669	-0.216558
40	1	0	6.460743	-1.421029	-0.128579
41	6	0	4.079588	2.943885	0.244011
42	6	0	1.377271	2.235219	0.148312
43	6	0	1.752745	3.537151	0.258861
44	1	0	0.995569	4.308868	0.315257
45	6	0	3.124649	3.904249	0.310981
46	1	0	3.398180	4.946978	0.403868
47	1	0	0.328938	1.980574	0.143590
48	1	0	5.131276	3.202076	0.278855

Structure 112 (M06-2X/def2-TZVP, DMSO)

Energy (Hartrees): = - 1243.7193861
No imaginary frequencies

Standard orientation:

Center Number	Atomic Number	Atomic Type	Coordinates (Angstroms) X	Y	Z
1	6	0	-2.405742	-0.998379	1.382044
2	6	0	-1.673176	-0.709674	0.063468
3	6	0	-2.435706	0.351919	-0.731597
4	6	0	-3.876810	-0.083966	-0.889134
5	6	0	-4.500230	-0.315716	0.481804
6	1	0	-2.412833	1.307143	-0.194533
7	1	0	-3.901241	-1.027768	-1.450032
8	1	0	-4.496481	0.624433	1.043917
9	1	0	-1.642346	-1.629494	-0.532184
10	8	0	-3.749309	-1.308495	1.168253
11	6	0	-5.925165	-0.838303	0.394862
12	1	0	-5.944493	-1.724420	-0.251974
13	1	0	-6.246403	-1.142231	1.392163
14	8	0	-6.831443	0.147882	-0.055237
15	1	0	-6.446744	0.549290	-0.845342
16	8	0	-4.630956	0.906822	-1.567745
17	1	0	-4.160891	1.122936	-2.383770

18	8	0	-1.908454	0.509282	-2.036400
19	1	0	-1.083527	1.008651	-1.986581
20	7	0	-0.349614	-0.214599	0.376305
21	6	0	2.015124	-0.168098	-0.138492
22	6	0	2.339029	1.193645	0.025044
23	6	0	3.029985	-1.144730	-0.175296
24	6	0	3.716998	1.568570	0.164801
25	6	0	4.399230	-0.749515	-0.045072
26	6	0	4.708659	0.595009	0.125051
27	1	0	5.746932	0.892182	0.228494
28	6	0	0.621033	-0.623518	-0.327168
29	1	0	0.457092	-1.362057	-1.118367
30	1	0	-1.979136	-1.889546	1.851336
31	8	0	-2.314865	0.101579	2.250389
32	1	0	-1.451661	0.511921	2.090453
33	6	0	2.753017	-2.543055	-0.303443
34	6	0	5.426164	-1.741048	-0.082377
35	6	0	5.119054	-3.055899	-0.226958
36	1	0	5.901412	-3.803965	-0.254777
37	6	0	3.758908	-3.458525	-0.332494
38	1	0	3.524323	-4.511252	-0.430545
39	1	0	1.729287	-2.887127	-0.361539
40	1	0	6.455124	-1.413954	0.012571
41	6	0	4.062291	2.944739	0.331924
42	6	0	1.369216	2.246877	0.020497
43	6	0	1.740233	3.546815	0.171990
44	1	0	0.985169	4.323486	0.160915
45	6	0	3.105617	3.907422	0.341922
46	1	0	3.372563	4.948921	0.469326
47	1	0	0.324432	2.005217	-0.101810
48	1	0	5.110560	3.195832	0.445807

Structure 112.5H_2O (M06-2X/def2-TZVP, Gas Phase)

Energy (Hartrees): = - 1625.9434559
No imaginary frequencies

Standard orientation:

Center Number	Atomic Number	Atomic Type	Coordinates (Angstroms) X	Y	Z
1	6	0	-3.524933	-0.766002	-0.481026
2	6	0	-2.683781	0.359403	-0.567750
3	6	0	-3.122529	1.623493	-0.130931
4	6	0	-4.444037	1.748427	0.409671
5	6	0	-5.268374	0.631502	0.481957
6	6	0	-4.845200	-0.619356	0.048890
7	6	0	-1.340139	0.151834	-1.145124
8	7	0	-0.280689	0.599819	-0.619130
9	6	0	0.989296	0.226738	-1.208155
10	6	0	1.538421	-0.956702	-0.412682
11	6	0	2.996716	-1.243772	-0.746174
12	6	0	3.795769	0.046446	-0.605884
13	8	0	3.240544	1.056208	-1.443301
14	6	0	1.938279	1.427416	-1.139694
15	8	0	0.670363	-2.043920	-0.681764
16	8	0	1.901354	2.017717	0.137569
17	6	0	5.240560	-0.107039	-1.028642
18	8	0	6.035476	0.982816	-0.611804
19	8	0	3.547520	-2.165952	0.172728
20	1	0	-1.289471	-0.460397	-2.051304
21	1	0	0.891927	-0.084024	-2.255425
22	1	0	1.048499	-2.886350	-0.365859
23	1	0	1.500943	-0.699430	0.654133
24	1	0	3.217273	-3.059113	-0.002330
25	1	0	3.074138	-1.615547	-1.776652
26	1	0	5.512560	1.806430	-0.651396
27	1	0	5.650211	-1.008295	-0.573121
28	1	0	5.273174	-0.215253	-2.119075
29	1	0	3.761879	0.367398	0.442489
30	1	0	1.010170	1.892271	0.494490
31	1	0	1.663133	2.164851	-1.899261
32	1	0	4.899614	2.760191	1.267321
33	8	0	4.594339	3.154127	0.435896
34	1	0	3.645220	2.975681	0.401848
35	1	0	1.715155	-3.980992	1.338956
36	8	0	1.810306	-4.332060	0.419839
37	1	0	1.574489	-5.261796	0.410498
38	8	0	1.690379	-2.783201	2.556716
39	1	0	0.869465	-2.261108	2.493020
40	1	0	2.410612	-2.210220	2.263367
41	1	0	-0.539274	-1.756369	0.820483
42	8	0	-0.728308	-1.598575	1.760311
43	1	0	-1.139190	-0.728733	1.793404
44	8	0	5.781971	1.215586	2.127631
45	1	0	6.109931	1.007918	1.232890

46	1	0	6.531828	1.160810	2.723532
47	1	0	-6.267787	0.739664	0.889522
48	6	0	-3.101300	-2.078135	-0.863190
49	6	0	-5.696550	-1.761012	0.133985
50	6	0	-3.943141	-3.140494	-0.765507
51	6	0	-5.265276	-2.983140	-0.267379
52	1	0	-2.088549	-2.237727	-1.211117
53	1	0	-3.601184	-4.124300	-1.059528
54	1	0	-5.916876	-3.844373	-0.199881
55	1	0	-6.694844	-1.627331	0.533111
56	6	0	-2.331755	2.811471	-0.227322
57	6	0	-4.900888	3.023863	0.859219
58	6	0	-2.808079	4.008496	0.204711
59	6	0	-4.109059	4.120791	0.767199
60	1	0	-2.189988	4.893042	0.119219
61	1	0	-1.340711	2.750226	-0.649946
62	1	0	-4.462902	5.083829	1.110938
63	1	0	-5.900168	3.092975	1.271987

Structure 112.5H_2O (M06-2X/def2-TZVP, DMSO)

Energy (Hartrees): = - 1625.9846781
No imaginary frequencies

Standard orientation:

Center Number	Atomic Number	Atomic Type	Coordinates (Angstroms) X	Y	Z
1	6	0	-3.618974	-0.727901	-0.506647
2	6	0	-2.715439	0.348859	-0.571689
3	6	0	-3.090194	1.629191	-0.123530
4	6	0	-4.403769	1.816823	0.421325
5	6	0	-5.286843	0.744671	0.486679
6	6	0	-4.928253	-0.519658	0.032292
7	6	0	-1.372253	0.086985	-1.134606
8	7	0	-0.310033	0.432857	-0.540469
9	6	0	0.961334	0.081640	-1.143620
10	6	0	1.570951	-1.057511	-0.331827
11	6	0	3.030396	-1.303923	-0.687838
12	6	0	3.788354	0.014223	-0.605240
13	8	0	3.178174	0.990311	-1.445732
14	6	0	1.865453	1.317357	-1.119499
15	8	0	0.749305	-2.191466	-0.551736
16	8	0	1.850147	1.935682	0.148986
17	6	0	5.220554	-0.097317	-1.072221
18	8	0	5.957080	1.081382	-0.780719
19	8	0	3.628434	-2.178930	0.254902
20	1	0	-1.330858	-0.448284	-2.088256
21	1	0	0.854694	-0.245567	-2.183863
22	1	0	1.186577	-3.005761	-0.240315
23	1	0	1.546027	-0.771173	0.728588
24	1	0	3.351634	-3.091131	0.080936
25	1	0	3.104352	-1.710465	-1.703741
26	1	0	5.359758	1.848920	-0.828778
27	1	0	5.709061	-0.926747	-0.561878
28	1	0	5.227452	-0.295512	-2.149360
29	1	0	3.772120	0.358228	0.436666
30	1	0	0.973457	1.793823	0.538579
31	1	0	1.540638	2.027968	-1.883449
32	1	0	4.870578	2.845577	1.102960
33	8	0	4.433109	3.245179	0.334123
34	1	0	3.526702	2.903298	0.342233
35	1	0	1.865991	-4.171654	1.408075
36	8	0	2.036865	-4.452183	0.485861
37	1	0	1.814252	-5.385410	0.396702
38	8	0	1.732991	-2.825944	2.626976
39	1	0	0.887561	-2.358530	2.487494
40	1	0	2.413630	-2.263130	2.232130
41	1	0	-0.508937	-1.901793	0.873982
42	8	0	-0.740816	-1.715360	1.801082
43	1	0	-0.931119	-0.768437	1.810935
44	8	0	6.025525	1.543222	1.961507
45	1	0	6.231784	1.251413	1.054572
46	1	0	6.867902	1.737807	2.385887
47	1	0	-6.277288	0.898219	0.901989
48	6	0	-3.272217	-2.052162	-0.924110
49	6	0	-5.839644	-1.616261	0.107125
50	6	0	-4.169331	-3.070981	-0.838174
51	6	0	-5.476081	-2.853595	-0.318514
52	1	0	-2.279367	-2.254168	-1.305526
53	1	0	-3.886222	-4.065033	-1.161296
54	1	0	-6.171316	-3.681437	-0.258767
55	1	0	-6.826431	-1.434290	0.517089
56	6	0	-2.240224	2.777582	-0.214381
57	6	0	-4.794973	3.110341	0.884572
58	6	0	-2.654388	3.994180	0.230129

59	6	0	-3.947239	4.166697	0.799997
60	1	0	-1.994489	4.848873	0.146086
61	1	0	-1.257886	2.675162	-0.651977
62	1	0	-4.250531	5.144704	1.151660
63	1	0	-5.789350	3.223951	1.300579

Structure 112.5H$_2$O (M06-2X/def2-TZVP, H$_2$O)

Energy (Hartrees): = - 1625.9991907
No imaginary frequencies

Standard orientation:

Center Number	Atomic Number	Atomic Type	Coordinates (Angstroms) X	Y	Z
1	6	0	-3.580656	-0.715592	-0.563175
2	6	0	-2.677715	0.363777	-0.575143
3	6	0	-3.071902	1.634964	-0.116759
4	6	0	-4.404848	1.809809	0.382671
5	6	0	-5.287032	0.735065	0.396032
6	6	0	-4.909088	-0.519843	-0.068363
7	6	0	-1.320478	0.115068	-1.105640
8	7	0	-0.266992	0.473739	-0.500568
9	6	0	1.004932	0.132820	-1.105629
10	6	0	1.633731	-1.016716	-0.325100
11	6	0	3.067505	-1.250480	-0.780256
12	6	0	3.854260	0.052803	-0.708339
13	8	0	3.207137	1.074593	-1.465509
14	6	0	1.897006	1.374105	-1.074423
15	8	0	0.815365	-2.154245	-0.549710
16	8	0	1.919512	1.949767	0.213445
17	6	0	5.235069	-0.099135	-1.304924
18	8	0	6.024199	1.069617	-1.120426
19	8	0	3.739923	-2.169909	0.061886
20	1	0	-1.259363	-0.418849	-2.058868
21	1	0	0.896707	-0.178867	-2.150741
22	1	0	1.163021	-2.915916	-0.040763
23	1	0	1.649567	-0.769279	0.744033
24	1	0	3.243150	-3.001488	0.116450
25	1	0	3.053816	-1.610238	-1.817044
26	1	0	5.527262	1.830269	-1.450265
27	1	0	5.761665	-0.913221	-0.810605
28	1	0	5.142100	-0.331027	-2.370341
29	1	0	3.935779	0.364752	0.339618
30	1	0	1.072168	1.756877	0.644035
31	1	0	1.538891	2.105617	-1.801266
32	1	0	4.949795	2.376449	1.448768
33	8	0	4.342137	3.094556	1.204518
34	1	0	3.535640	2.656099	0.891056
35	1	0	1.735666	-3.743438	1.789718
36	8	0	1.959956	-4.194978	0.948021
37	1	0	1.475348	-5.028085	0.938143
38	8	0	1.305430	-2.497858	3.011751
39	1	0	0.472658	-2.194979	2.598638
40	1	0	1.944849	-1.795236	2.841929
41	1	0	-0.616768	-1.970244	0.722482
42	8	0	-1.054984	-2.038076	1.590481
43	1	0	-1.649441	-1.278950	1.638067
44	8	0	6.288786	1.029042	1.652271
45	1	0	6.347816	1.069473	0.678599
46	1	0	7.112942	1.409248	1.977390
47	1	0	-6.292257	0.879501	0.777083
48	6	0	-3.216943	-2.030237	-0.997264
49	6	0	-5.821125	-1.618278	-0.049371
50	6	0	-4.116149	-3.050349	-0.967333
51	6	0	-5.440837	-2.845414	-0.489300
52	1	0	-2.209592	-2.223397	-1.343354
53	1	0	-3.820999	-4.036504	-1.302638
54	1	0	-6.135701	-3.675111	-0.473137
55	1	0	-6.821674	-1.445231	0.329108
56	6	0	-2.222524	2.786777	-0.155807
57	6	0	-4.817138	3.093491	0.854565
58	6	0	-2.657226	3.994168	0.294282
59	6	0	-3.970308	4.153384	0.819466
60	1	0	-1.997749	4.851847	0.251002
61	1	0	-1.222132	2.692721	-0.552147
62	1	0	-4.289070	5.123768	1.177682
63	1	0	-5.826037	3.196073	1.236379

Structure 115 (M06-2X, Gas Phase)

Energy (Hartrees): = - 936.3271529
No imaginary frequencies

Standard orientation:

Center Number	Atomic Number	Atomic Type	Coordinates (Angstroms) X	Y	Z
1	6	0	0.592989	1.511248	0.925433
2	6	0	0.368393	0.333847	-0.038776
3	6	0	1.338486	-0.823832	0.228298
4	6	0	2.710520	-0.676092	-0.422591
5	6	0	3.533703	0.504664	0.071281
6	1	0	1.410034	1.393767	1.662527
7	1	0	1.477852	-0.936593	1.315278
8	1	0	2.557832	-0.545054	-1.503917
9	1	0	3.546346	0.514174	1.170819
10	1	0	0.516365	0.732484	-1.049977
11	8	0	2.879303	1.653025	-0.452916
12	6	0	4.984967	0.428922	-0.407139
13	1	0	4.996004	0.189776	-1.479586
14	1	0	5.441658	1.415612	-0.282119
15	8	0	5.750313	-0.471505	0.351200
16	1	0	5.310762	-1.326278	0.276476
17	8	0	3.456623	-1.854966	-0.174957
18	1	0	2.861675	-2.595697	-0.339574
19	8	0	0.829550	-2.028578	-0.311890
20	1	0	-0.107272	-2.057833	-0.080374
21	8	0	-0.099416	2.485881	0.914724
22	7	0	-0.986429	-0.161935	0.150103
23	6	0	-3.346646	0.140248	-0.197756
24	6	0	-3.726623	-0.914740	0.636410
25	6	0	-4.322698	0.859087	-0.887098
26	6	0	-5.065308	-1.245708	0.769790
27	1	0	-2.957518	-1.454752	1.175029
28	6	0	-5.665418	0.525103	-0.754278
29	1	0	-4.026630	1.683566	-1.527266
30	6	0	-6.036790	-0.527292	0.073567
31	1	0	-5.358421	-2.062232	1.418630
32	1	0	-6.419723	1.085904	-1.292570
33	1	0	-7.082945	-0.787724	0.181562
34	6	0	-1.930855	0.511675	-0.363988
35	1	0	-1.736075	1.410658	-0.960102
36	1	0	3.335756	2.440121	-0.143756

Structure 115 (M06-2X, DMSO)

Energy (Hartrees): = - 936.3539074
No imaginary frequencies

Standard orientation:

Center Number	Atomic Number	Atomic Type	Coordinates (Angstroms) X	Y	Z
1	6	0	0.651914	1.572291	0.877443
2	6	0	0.364315	0.387902	-0.055193
3	6	0	1.300088	-0.798312	0.194933
4	6	0	2.683735	-0.677286	-0.434643
5	6	0	3.529234	0.469931	0.100937
6	1	0	1.356145	1.388976	1.709648
7	1	0	1.416406	-0.939991	1.279087
8	1	0	2.558300	-0.525510	-1.516146
9	1	0	3.501782	0.469649	1.199041
10	1	0	0.452470	0.751094	-1.086390
11	8	0	2.935068	1.648187	-0.429695
12	6	0	4.988427	0.361846	-0.341462
13	1	0	5.022634	0.160583	-1.421272
14	1	0	5.472522	1.326721	-0.166480
15	8	0	5.709020	-0.601784	0.397381
16	1	0	5.218978	-1.425921	0.292567
17	8	0	3.393717	-1.885472	-0.194760
18	1	0	2.780415	-2.606943	-0.380289
19	8	0	0.760675	-1.976900	-0.388771
20	1	0	-0.132857	-2.083372	-0.040188
21	8	0	0.085898	2.623556	0.758012
22	7	0	-0.991363	-0.048972	0.253085
23	6	0	-3.345544	0.094808	-0.224072
24	6	0	-3.737923	-0.740614	0.827338
25	6	0	-4.312746	0.635056	-1.073335
26	6	0	-5.080638	-1.029644	1.019911
27	1	0	-2.982560	-1.153429	1.485962

28	6	0	-5.659485	0.345156	-0.877960
29	1	0	-4.005280	1.283153	-1.887828
30	6	0	-6.043757	-0.487233	0.167845
31	1	0	-5.382230	-1.675969	1.836051
32	1	0	-6.405573	0.767355	-1.540993
33	1	0	-7.092333	-0.714053	0.323386
34	6	0	-1.927922	0.423668	-0.461271
35	1	0	-1.728806	1.102018	-1.298933
36	1	0	3.356330	2.412325	-0.018986

Structure 116 (M06-2X, Gas Phase)

Energy (Hartrees): = - 936.3289894
No imaginary frequencies

Standard orientation:

Center Number	Atomic Number	Atomic Type	Coordinates (Angstroms) X	Y	Z
1	6	0	-0.635340	-1.662157	0.754817
2	6	0	-0.325814	-0.433222	-0.103294
3	6	0	-1.238302	0.747268	0.230031
4	6	0	-2.595351	0.698610	-0.461147
5	6	0	-3.451578	-0.506030	-0.092171
6	1	0	-1.390408	0.781328	1.314940
7	1	0	-2.426233	0.664389	-1.547925
8	1	0	-3.426770	-0.659988	0.993517
9	1	0	-0.423532	-0.722019	-1.158432
10	8	0	-2.857475	-1.606443	-0.778862
11	6	0	-4.907859	-0.326228	-0.524391
12	1	0	-4.933045	0.034966	-1.562013
13	1	0	-5.394812	-1.305700	-0.501793
14	8	0	-5.627446	0.506631	0.349037
15	1	0	-5.156976	1.347566	0.363442
16	8	0	-3.314608	1.871955	-0.121088
17	1	0	-2.694078	2.605151	-0.200853
18	8	0	-0.660793	1.962043	-0.227422
19	1	0	0.207755	2.037402	0.181021
20	7	0	1.043647	-0.069827	0.236060
21	6	0	3.390525	-0.163404	-0.281237
22	6	0	3.791480	0.449952	0.909155
23	6	0	4.346654	-0.515466	-1.232814
24	6	0	5.133417	0.709804	1.135391
25	1	0	3.035762	0.706457	1.641686
26	6	0	5.692770	-0.253304	-1.004594
27	1	0	4.033969	-0.994434	-2.155065
28	6	0	6.085636	0.359755	0.178985
29	1	0	5.443815	1.183271	2.059012
30	1	0	6.431878	-0.527234	-1.747443
31	1	0	7.134216	0.563701	0.360462
32	6	0	1.968880	-0.443657	-0.546317
33	1	0	1.751155	-0.990170	-1.475155
34	1	0	-0.190881	-2.602129	0.370382
35	8	0	-1.236502	-1.633264	1.789175
36	1	0	-3.263460	-2.412679	-0.449415

Structure 116 (M06-2X, DMSO)

Energy (Hartrees): = - 936.3556975
No imaginary frequencies
Standard orientation:

Center Number	Atomic Number	Atomic Type	Coordinates (Angstroms) X	Y	Z
1	6	0	-0.644024	-1.648849	0.701034
2	6	0	-0.330685	-0.425058	-0.158561
3	6	0	-1.240818	0.759875	0.162359
4	6	0	-2.617921	0.683156	-0.484200
5	6	0	-3.450044	-0.517600	-0.050226
6	1	0	-1.361839	0.831321	1.249540
7	1	0	-2.488019	0.623949	-1.574417
8	1	0	-3.389242	-0.635101	1.039030
9	1	0	-0.404955	-0.708278	-1.214958
10	8	0	-2.871471	-1.635214	-0.716415
11	6	0	-4.920479	-0.371970	-0.441641

12	1	0	-4.985710	-0.068025	-1.495550
13	1	0	-5.398196	-1.351086	-0.346260
14	8	0	-5.621815	0.513443	0.406405
15	1	0	-5.132672	1.344227	0.371271
16	8	0	-3.338201	1.862035	-0.147719
17	1	0	-2.727555	2.600218	-0.263043
18	8	0	-0.682190	1.964592	-0.347674
19	1	0	0.179383	2.081496	0.069964
20	7	0	1.035162	-0.062285	0.211263
21	6	0	3.388407	-0.153229	-0.275271
22	6	0	3.775387	0.531763	0.881806
23	6	0	4.360045	-0.581723	-1.181039
24	6	0	5.117759	0.782673	1.123220
25	1	0	3.015938	0.860779	1.581527
26	6	0	5.706532	-0.329560	-0.936712
27	1	0	4.057241	-1.114644	-2.076752
28	6	0	6.085723	0.352043	0.214874
29	1	0	5.414957	1.314583	2.019626
30	1	0	6.456524	-0.666698	-1.642659
31	1	0	7.134053	0.549327	0.407913
32	6	0	1.970272	-0.436108	-0.562377
33	1	0	1.768358	-0.984800	-1.490488
34	1	0	-0.323088	-2.610809	0.262158
35	8	0	-1.111296	-1.588258	1.806882
36	1	0	-3.262455	-2.435723	-0.347167

Structure 117e (M06-2X, Gas Phase)

Energy (Hartrees): = - 936.3352045
No imaginary frequencies

Standard orientation:

Center Number	Atomic Number	Atomic Type	Coordinates (Angstroms) X	Y	Z
1	6	0	0.553110	-0.301139	-1.174370
2	6	0	0.237685	0.809868	-0.192618
3	6	0	1.526700	1.555926	0.056841
4	6	0	2.501273	0.510311	0.600558
5	6	0	2.631387	-0.719498	-0.316042
6	1	0	1.071851	0.099226	-2.061434
7	1	0	1.918287	1.964496	-0.883904
8	1	0	2.106820	0.173357	1.570538
9	1	0	3.107952	-0.401441	-1.255002
10	1	0	-0.084012	0.339805	0.749370
11	8	0	1.348473	-1.282915	-0.586539
12	6	0	3.492391	-1.810067	0.307440
13	1	0	3.127660	-2.016954	1.323470
14	1	0	3.370405	-2.717113	-0.286132
15	8	0	4.857832	-1.471887	0.292616
16	1	0	4.937514	-0.588554	0.669516
17	8	0	3.791018	1.067511	0.767335
18	1	0	3.675782	1.880914	1.270679
19	8	0	1.454980	2.570102	1.045674
20	1	0	1.055587	3.351501	0.656159
21	8	0	-0.704178	-0.787566	-1.542556
22	7	0	-0.855244	1.446181	-0.907180
23	6	0	-2.753320	-0.119582	-0.451347
24	6	0	-3.941653	0.610215	-0.470575
25	6	0	-2.594866	-1.151339	0.470711
26	6	0	-4.956025	0.327199	0.435208
27	1	0	-4.075529	1.398630	-1.205833
28	6	0	-3.612530	-1.435018	1.376003
29	1	0	-1.682610	-1.736242	0.456963
30	6	0	-4.790611	-0.696517	1.363171
31	1	0	-5.877290	0.896994	0.412107
32	1	0	-3.486684	-2.240292	2.089960
33	1	0	-5.582013	-0.922930	2.067537
34	6	0	-1.641341	0.273665	-1.399852
35	1	0	-1.440848	1.992848	-0.284080
36	1	0	-2.054363	0.516381	-2.382657

Structure 117e (M06-2X, DMSO)

Energy (Hartrees): = - 936.3618653

No imaginary frequencies

Standard orientation:

Center Number	Atomic Number	Atomic Type	Coordinates (Angstroms) X	Y	Z
1	6	0	0.550190	-0.245293	-1.209306
2	6	0	0.234581	0.827128	-0.188515
3	6	0	1.525149	1.559172	0.096979
4	6	0	2.492656	0.487667	0.602837
5	6	0	2.617989	-0.714718	-0.351123
6	1	0	1.082538	0.182631	-2.073618
7	1	0	1.920143	2.005570	-0.824348
8	1	0	2.104751	0.123331	1.564501
9	1	0	3.101373	-0.372065	-1.277097
10	1	0	-0.098545	0.331317	0.735669
11	8	0	1.331827	-1.261421	-0.654439
12	6	0	3.450455	-1.835662	0.254499
13	1	0	3.042598	-2.089074	1.242579
14	1	0	3.363347	-2.715461	-0.385751
15	8	0	4.820552	-1.496789	0.326419
16	1	0	4.865968	-0.615650	0.717226
17	8	0	3.792438	1.030442	0.779572
18	1	0	3.690105	1.822343	1.321902
19	8	0	1.446599	2.528643	1.128749
20	1	0	1.061877	3.332110	0.762195
21	8	0	-0.708248	-0.710235	-1.613735
22	7	0	-0.849101	1.492367	-0.892998
23	6	0	-2.738330	-0.095313	-0.442942
24	6	0	-3.946443	0.603782	-0.439601
25	6	0	-2.547876	-1.140298	0.459833
26	6	0	-4.949067	0.272498	0.464489
27	1	0	-4.100505	1.408301	-1.152742
28	6	0	-3.553142	-1.472615	1.364604
29	1	0	-1.618575	-1.697833	0.441235
30	6	0	-4.752823	-0.767419	1.370357
31	1	0	-5.884821	0.819617	0.458563
32	1	0	-3.399660	-2.286914	2.063638
33	1	0	-5.535371	-1.030822	2.072617
34	6	0	-1.650001	0.343879	-1.401664
35	1	0	-1.421554	2.055515	-0.269874
36	1	0	-2.095340	0.618553	-2.360918

Structure 117a (M06-2X, Gas Phase)

Energy (Hartrees): = - 936.3367878
No imaginary frequencies

Standard orientation:

Center Number	Atomic Number	Atomic Type	Coordinates (Angstroms) X	Y	Z
1	6	0	-0.539761	-0.274312	1.297901
2	6	0	-0.210288	0.881306	0.367406
3	6	0	-1.489214	1.584483	0.003829
4	6	0	-2.366258	0.499828	-0.628206
5	6	0	-2.511533	-0.759286	0.247664
6	1	0	-1.165872	0.060659	2.144375
7	1	0	-1.993298	1.976803	0.900593
8	1	0	-1.874017	0.206848	-1.566824
9	1	0	-3.080194	-0.491091	1.150256
10	1	0	0.217595	0.447894	-0.543954
11	8	0	-1.231895	-1.275856	0.612041
12	6	0	-3.267703	-1.868099	-0.473989
13	1	0	-2.819278	-2.021031	-1.465553
14	1	0	-3.142000	-2.787943	0.098538
15	8	0	-4.646479	-1.599668	-0.550629
16	1	0	-4.744153	-0.713327	-0.915684
17	8	0	-3.664941	0.996536	-0.892443
18	1	0	-3.558822	1.805761	-1.404029
19	8	0	-1.354928	2.599185	-0.970922
20	1	0	-0.644586	3.188418	-0.702542
21	8	0	0.706294	-0.680591	1.781306
22	7	0	0.875556	1.556630	1.070696
23	6	0	2.632844	-0.049802	0.435352
24	6	0	3.451003	0.908262	-0.162282
25	6	0	2.753893	-1.386458	0.068290
26	6	0	4.384135	0.530922	-1.118892

27	1	0	3.335501	1.949425	0.115639
28	6	0	3.689518	-1.761360	-0.891223
29	1	0	2.103921	-2.120379	0.527320
30	6	0	4.506183	-0.806558	-1.484513
31	1	0	5.014344	1.280494	-1.582731
32	1	0	3.776110	-2.802782	-1.177472
33	1	0	5.232428	-1.101331	-2.232662
34	6	0	1.646499	0.382271	1.503686
35	1	0	0.501799	2.014588	1.900408
36	1	0	2.171316	0.606589	2.434485

Structure 117a (M06-2X, DMSO)

Energy (Hartrees): = - 936.3647092
No imaginary frequencies

Standard orientation:

Center Number	Atomic Number	Atomic Type	Coordinates (Angstroms) X	Y	Z
1	6	0	-0.539348	-0.143962	1.303323
2	6	0	-0.208978	0.899446	0.249515
3	6	0	-1.492944	1.583003	-0.146343
4	6	0	-2.398435	0.454682	-0.647595
5	6	0	-2.536359	-0.715456	0.344881
6	1	0	-1.142483	0.288685	2.118505
7	1	0	-1.958537	2.066602	0.722929
8	1	0	-1.946991	0.067294	-1.571938
9	1	0	-3.083540	-0.357199	1.228560
10	1	0	0.181251	0.366309	-0.625639
11	8	0	-1.255140	-1.209989	0.740700
12	6	0	-3.301627	-1.882525	-0.263580
13	1	0	-2.846906	-2.145667	-1.228264
14	1	0	-3.206324	-2.741050	0.403730
15	8	0	-4.679806	-1.598710	-0.396458
16	1	0	-4.741599	-0.737024	-0.826371
17	8	0	-3.705139	0.941488	-0.913439
18	1	0	-3.607112	1.696809	-1.506121
19	8	0	-1.378542	2.493551	-1.224344
20	1	0	-0.918283	3.278543	-0.907987
21	8	0	0.709026	-0.531744	1.801001
22	7	0	0.899516	1.620981	0.865591
23	6	0	2.664909	-0.041671	0.439082
24	6	0	3.738432	0.771584	0.070546
25	6	0	2.542770	-1.309952	-0.121113
26	6	0	4.678013	0.321567	-0.848298
27	1	0	3.831141	1.762390	0.504525
28	6	0	3.483502	-1.760043	-1.046179
29	1	0	1.708554	-1.938823	0.165398
30	6	0	4.551576	-0.948304	-1.410196
31	1	0	5.509681	0.958715	-1.127080
32	1	0	3.378546	-2.747420	-1.481751
33	1	0	5.283828	-1.301071	-2.127560
34	6	0	1.657128	0.499222	1.436518
35	1	0	0.539005	2.186155	1.633975
36	1	0	2.168135	0.827565	2.342916

Structure 118e (M06-2X, Gas Phase)

Energy (Hartrees): = - 936.3326884
No imaginary frequencies

Standard orientation:

Center Number	Atomic Number	Atomic Type	Coordinates (Angstroms) X	Y	Z
1	6	0	-0.398705	-0.607286	-0.064160
2	6	0	-0.560124	0.780065	-0.657267
3	6	0	-1.684698	1.455124	0.090002
4	6	0	-2.903791	0.554647	-0.112870
5	6	0	-2.639827	-0.906965	0.293927
6	1	0	-0.300198	-0.549923	1.033201
7	1	0	-1.451008	1.511029	1.161164
8	1	0	-3.145274	0.571826	-1.185938
9	1	0	-2.488744	-0.940226	1.382988
10	1	0	-0.861643	0.651970	-1.708814

11	8	0	-1.490597	-1.416201	-0.377761
12	6	0	-3.812818	-1.815430	-0.051833
13	1	0	-4.083834	-1.661815	-1.105896
14	1	0	-3.483852	-2.848767	0.064761
15	8	0	-4.905790	-1.616151	0.811280
16	1	0	-5.087541	-0.670142	0.829635
17	8	0	-4.003523	1.027430	0.641033
18	1	0	-4.103619	1.961065	0.425101
19	8	0	-2.053768	2.733020	-0.405139
20	1	0	-1.455248	3.389437	-0.041136
21	8	0	0.792269	-1.072041	-0.621748
22	7	0	0.807726	1.242369	-0.516183
23	6	0	2.943329	-0.016563	-0.295913
24	6	0	3.995995	-0.632191	-0.966877
25	6	0	3.138234	0.461416	0.997768
26	6	0	5.234657	-0.775005	-0.351578
27	1	0	3.842435	-1.007443	-1.973487
28	6	0	4.376252	0.321672	1.612803
29	1	0	2.316854	0.954461	1.503087
30	6	0	5.425508	-0.297568	0.940042
31	1	0	6.049001	-1.257313	-0.879037
32	1	0	4.524462	0.697687	2.618211
33	1	0	6.390599	-0.405853	1.420840
34	6	0	1.592052	0.071868	-0.960767
35	1	0	1.020284	2.043758	-1.099690
36	1	0	1.718139	0.052511	-2.052675

Structure 118e (M06-2X, DMSO)

Energy (Hartrees): = - 936.362116
No imaginary frequencies

Standard orientation:

Center Number	Atomic Number	Atomic Type	Coordinates (Angstroms) X	Y	Z
1	6	0	-0.432563	-0.624175	-0.066496
2	6	0	-0.540737	0.825040	-0.493140
3	6	0	-1.674791	1.439726	0.292183
4	6	0	-2.905440	0.599739	-0.054593
5	6	0	-2.690036	-0.908687	0.168969
6	1	0	-0.386555	-0.708405	1.030813
7	1	0	-1.474908	1.368448	1.368768
8	1	0	-3.123932	0.760886	-1.119691
9	1	0	-2.571495	-1.083974	1.247789
10	1	0	-0.803302	0.838264	-1.562584
11	8	0	-1.527309	-1.366499	-0.523926
12	6	0	-3.866134	-1.731740	-0.336334
13	1	0	-4.068340	-1.463723	-1.382272
14	1	0	-3.586919	-2.786647	-0.303899
15	8	0	-5.013061	-1.571180	0.473733
16	1	0	-5.128701	-0.621606	0.601765
17	8	0	-4.018233	0.994901	0.733465
18	1	0	-4.108006	1.950232	0.627257
19	8	0	-1.996856	2.772719	-0.069796
20	1	0	-1.379056	3.364958	0.372024
21	8	0	0.776718	-1.054344	-0.623296
22	7	0	0.836214	1.226158	-0.257878
23	6	0	2.955080	-0.055211	-0.289626
24	6	0	4.083508	-0.008663	-1.101011
25	6	0	3.099236	-0.262339	1.083529
26	6	0	5.354233	-0.162267	-0.547273
27	1	0	3.968400	0.149060	-2.168801
28	6	0	4.362758	-0.420748	1.635792
29	1	0	2.216072	-0.291523	1.713213
30	6	0	5.493765	-0.369653	0.819605
31	1	0	6.230303	-0.124055	-1.184554
32	1	0	4.471750	-0.583607	2.702069
33	1	0	6.480000	-0.492202	1.252959
34	6	0	1.579831	0.113593	-0.880905
35	1	0	1.074180	2.096372	-0.725801
36	1	0	1.654669	0.231939	-1.969554

Structure 118a (M06-2X, Gas Phase)

Energy (Hartrees): = - 936.3372261
No imaginary frequencies

Standard orientation:

Center Number	Atomic Number	Atomic Type	Coordinates (Angstroms) X	Y	Z
1	6	0	-0.448845	-0.665648	-0.097951
2	6	0	-0.520334	0.812969	-0.440703
3	6	0	-1.631108	1.443923	0.355086
4	6	0	-2.885377	0.647758	-0.011132
5	6	0	-2.711862	-0.871861	0.165507
6	1	0	-0.377265	-0.813534	0.995183
7	1	0	-1.446000	1.340984	1.435419
8	1	0	-3.088270	0.847542	-1.073219
9	1	0	-2.586197	-1.084367	1.237528
10	1	0	-0.775292	0.878528	-1.505445
11	8	0	-1.578534	-1.340975	-0.563126
12	6	0	-3.927302	-1.645337	-0.330711
13	1	0	-4.175046	-1.309485	-1.347436
14	1	0	-3.654960	-2.700283	-0.381021
15	8	0	-5.020155	-1.529158	0.547214
16	1	0	-5.157225	-0.589353	0.709353
17	8	0	-3.985171	1.054754	0.781090
18	1	0	-4.050991	2.012651	0.702178
19	8	0	-1.908293	2.790431	0.025878
20	1	0	-1.092203	3.294028	0.091170
21	8	0	0.734085	-1.083194	-0.709058
22	7	0	0.864855	1.237384	-0.291231
23	6	0	2.935029	-0.099168	-0.324686
24	6	0	3.818937	0.980964	-0.326797
25	6	0	3.336694	-1.320760	0.205420
26	6	0	5.093321	0.840415	0.206296
27	1	0	3.498096	1.930429	-0.742034
28	6	0	4.616143	-1.457962	0.736729
29	1	0	2.645728	-2.153612	0.194355
30	6	0	5.494309	-0.381472	0.739518
31	1	0	5.774497	1.682911	0.204129
32	1	0	4.926735	-2.411373	1.147402
33	1	0	6.489366	-0.493007	1.153515
34	6	0	1.556630	0.091959	-0.902388
35	1	0	1.111695	1.253985	0.698668
36	1	0	1.610486	0.262719	-1.982241

Structure 118a (M06-2X, DMSO)

Energy (Hartrees): = - 936.3643644
No imaginary frequencies

Standard orientation:

Center Number	Atomic Number	Atomic Type	Coordinates (Angstroms) X	Y	Z
1	6	0	-0.447708	-0.659800	-0.132112
2	6	0	-0.513175	0.832110	-0.410545
3	6	0	-1.618798	1.421621	0.428328
4	6	0	-2.876925	0.651275	0.022985
5	6	0	-2.713067	-0.877081	0.114450
6	1	0	-0.378337	-0.856580	0.951132
7	1	0	-1.420574	1.257804	1.496435
8	1	0	-3.092848	0.910669	-1.023168
9	1	0	-2.589178	-1.148514	1.172808
10	1	0	-0.771500	0.951259	-1.469995
11	8	0	-1.578303	-1.316716	-0.633578
12	6	0	-3.924597	-1.610665	-0.444099
13	1	0	-4.130935	-1.237385	-1.456447
14	1	0	-3.679619	-2.672014	-0.515979
15	8	0	-5.054470	-1.492784	0.396230
16	1	0	-5.142154	-0.555731	0.608878
17	8	0	-3.972908	1.011109	0.849939
18	1	0	-4.040406	1.973623	0.824342
19	8	0	-1.899135	2.786288	0.174752
20	1	0	-1.172595	3.312627	0.525856
21	8	0	0.732307	-1.062769	-0.764823
22	7	0	0.878043	1.234133	-0.239531
23	6	0	2.938668	-0.105650	-0.340235
24	6	0	3.959959	0.781641	-0.680384
25	6	0	3.193828	-1.144194	0.551225
26	6	0	5.227360	0.633252	-0.128749
27	1	0	3.758013	1.588169	-1.378279

28	6	0	4.465473	-1.294615	1.099088
29	1	0	2.399387	-1.836174	0.803344
30	6	0	5.482470	-0.407653	0.761774
31	1	0	6.017798	1.324714	-0.397080
32	1	0	4.660898	-2.107772	1.788846
33	1	0	6.472153	-0.527566	1.187659
34	6	0	1.561871	0.119535	-0.908072
35	1	0	1.111802	1.203661	0.754032
36	1	0	1.619708	0.340602	-1.977752

Structure 119e (M06-2X, Gas Phase)

Energy (Hartrees): = - 936.3522072
No imaginary frequencies

Standard orientation:

Center Number	Atomic Number	Atomic Type	Coordinates (Angstroms) X	Y	Z
1	6	0	0.420689	-0.627035	1.066491
2	6	0	0.493760	0.909786	1.023178
3	6	0	1.543442	1.398467	0.030689
4	6	0	2.823045	0.608229	0.171114
5	6	0	2.505222	-0.865606	-0.060513
6	1	0	1.179495	1.241162	-0.994391
7	1	0	3.217698	0.725031	1.191292
8	1	0	2.030088	-0.991825	-1.041023
9	1	0	0.706117	1.342388	2.002468
10	8	0	1.628875	-1.298384	0.975475
11	6	0	3.740512	-1.754116	-0.002054
12	1	0	4.304599	-1.523893	0.912891
13	1	0	3.409517	-2.791772	0.057439
14	8	0	4.530822	-1.624636	-1.158747
15	1	0	4.717309	-0.686042	-1.269873
16	8	0	3.777931	1.033055	-0.782414
17	1	0	3.804684	1.995378	-0.745703
18	8	0	1.847750	2.765877	0.225431
19	1	0	1.034073	3.261571	0.089775
20	7	0	-0.833645	1.297861	0.549061
21	6	0	-2.684661	-0.056016	-0.326623
22	6	0	-3.567618	0.977702	-0.641724
23	6	0	-3.181384	-1.301648	0.040496
24	6	0	-4.938579	0.767822	-0.579446
25	1	0	-3.173697	1.946320	-0.931676
26	6	0	-4.556749	-1.509104	0.100475
27	1	0	-2.486155	-2.100016	0.266855
28	6	0	-5.435823	-0.478212	-0.206947
29	1	0	-5.619846	1.573805	-0.824594
30	1	0	-4.940050	-2.481576	0.385767
31	1	0	-6.505441	-0.644032	-0.161372
32	6	0	-1.200766	0.214271	-0.376667
33	1	0	-0.032260	-0.979912	1.997804
34	8	0	-0.431998	-0.934752	-0.026895
35	1	0	-1.493645	1.294855	1.321912
36	1	0	-0.910565	0.496996	-1.394616

Structure 119e (M06-2X, DMSO)

Energy (Hartrees): = - 936.3770404
No imaginary frequencies

Standard orientation:

Center Number	Atomic Number	Atomic Type	Coordinates (Angstroms) X	Y	Z
1	6	0	0.436655	-0.715873	1.051708
2	6	0	0.502240	0.820616	1.104832
3	6	0	1.541733	1.393581	0.144842
4	6	0	2.820677	0.591457	0.200494
5	6	0	2.497038	-0.859798	-0.137999
6	1	0	1.166469	1.333845	-0.885885
7	1	0	3.239783	0.629870	1.215954
8	1	0	1.997576	-0.908220	-1.113960
9	1	0	0.711322	1.177754	2.113855
10	8	0	1.647002	-1.378682	0.879683
11	6	0	3.733821	-1.746178	-0.173208
12	1	0	4.309269	-1.596055	0.750610

13	1	0	3.412089	-2.788808	-0.206272
14	8	0	4.519700	-1.507947	-1.323289
15	1	0	4.678358	-0.556864	-1.349414
16	8	0	3.761867	1.082192	-0.742147
17	1	0	3.819492	2.036962	-0.615388
18	8	0	1.860176	2.737981	0.459477
19	1	0	1.059156	3.259973	0.333879
20	7	0	-0.837933	1.221390	0.673929
21	6	0	-2.678556	-0.043325	-0.334873
22	6	0	-3.517140	0.817743	-1.040923
23	6	0	-3.225914	-1.086209	0.408631
24	6	0	-4.895929	0.638344	-1.003364
25	1	0	-3.085803	1.627011	-1.621904
26	6	0	-4.605480	-1.268380	0.441346
27	1	0	-2.568783	-1.758160	0.948707
28	6	0	-5.441993	-0.406811	-0.262482
29	1	0	-5.543172	1.308817	-1.557135
30	1	0	-5.027978	-2.085178	1.015521
31	1	0	-6.515867	-0.552214	-0.238175
32	6	0	-1.192448	0.212916	-0.330935
33	1	0	0.017833	-1.122996	1.975618
34	8	0	-0.451483	-0.975078	-0.027889
35	1	0	-1.477346	1.130661	1.460843
36	1	0	-0.873185	0.557650	-1.319437

Structure 119a (M06-2X, Gas Phase)

Energy (Hartrees): = - 936.342927
No imaginary frequencies

Standard orientation:

Center Number	Atomic Number	Atomic Type	Coordinates (Angstroms) X	Y	Z
1	6	0	-0.414196	-0.333775	-1.223797
2	6	0	-0.552066	1.157374	-0.874430
3	6	0	-1.609298	1.396035	0.209821
4	6	0	-2.840515	0.551620	-0.038076
5	6	0	-2.427867	-0.913102	-0.109293
6	1	0	-1.214274	1.103963	1.192837
7	1	0	-3.281853	0.832516	-1.005781
8	1	0	-1.891184	-1.197035	0.804365
9	1	0	-0.815901	1.746060	-1.756224
10	8	0	-1.588013	-1.067266	-1.246902
11	6	0	-3.610230	-1.854868	-0.290076
12	1	0	-4.234594	-1.492426	-1.119108
13	1	0	-3.222082	-2.836556	-0.564648
14	8	0	-4.345234	-2.004900	0.900194
15	1	0	-4.588109	-1.120512	1.194420
16	8	0	-3.779323	0.725784	1.006048
17	1	0	-3.902265	1.675007	1.114263
18	8	0	-2.047119	2.749492	0.247057
19	1	0	-1.387889	3.288347	0.689386
20	7	0	0.822348	1.411726	-0.466522
21	6	0	2.689942	-0.009140	0.299302
22	6	0	3.382317	-0.335159	1.459334
23	6	0	3.367186	0.038856	-0.918062
24	6	0	4.746430	-0.610312	1.410108
25	1	0	2.852522	-0.378615	2.405570
26	6	0	4.725394	-0.238117	-0.970112
27	1	0	2.818443	0.311268	-1.811853
28	6	0	5.417272	-0.563273	0.195228
29	1	0	5.281294	-0.862800	2.317781
30	1	0	5.249747	-0.200418	-1.917568
31	1	0	6.478537	-0.777893	0.153517
32	6	0	1.204702	0.252483	0.344918
33	1	0	0.028534	-0.464512	-2.213223
34	8	0	0.477051	-0.824029	-0.231544
35	1	0	0.969356	2.290455	0.016251
36	1	0	0.889953	0.336691	1.397662

Structure 119a (M06-2X, DMSO)

Energy (Hartrees): = - 936.3724153
No imaginary frequencies

Standard orientation:

Center Number	Atomic Number	Atomic Type	Coordinates (Angstroms) X	Y	Z
1	6	0	-0.451035	-0.462306	-1.251092
2	6	0	-0.556871	1.051810	-1.006881
3	6	0	-1.589737	1.410783	0.069804
4	6	0	-2.832253	0.560082	-0.067061
5	6	0	-2.431951	-0.907779	-0.006022
6	1	0	-1.174156	1.222769	1.068620
7	1	0	-3.303154	0.748718	-1.042238
8	1	0	-1.867672	-1.103706	0.914860
9	1	0	-0.823473	1.571698	-1.929607
10	8	0	-1.631898	-1.187669	-1.149325
11	6	0	-3.625229	-1.850077	-0.063728
12	1	0	-4.252822	-1.581283	-0.924439
13	1	0	-3.255925	-2.865887	-0.217321
14	8	0	-4.361146	-1.843257	1.142735
15	1	0	-4.552272	-0.917988	1.338250
16	8	0	-3.744166	0.839491	0.983700
17	1	0	-3.840841	1.799049	1.022269
18	8	0	-2.009917	2.766122	-0.026654
19	1	0	-1.329233	3.328838	0.358087
20	7	0	0.829113	1.321943	-0.634099
21	6	0	2.677171	-0.025036	0.283345
22	6	0	3.379254	0.004424	1.483022
23	6	0	3.355908	-0.286955	-0.908153
24	6	0	4.755407	-0.221247	1.497970
25	1	0	2.849288	0.204801	2.408902
26	6	0	4.724725	-0.517590	-0.895006
27	1	0	2.802711	-0.302605	-1.841332
28	6	0	5.427435	-0.484079	0.310391
29	1	0	5.298046	-0.194560	2.435963
30	1	0	5.248733	-0.722831	-1.821663
31	1	0	6.496532	-0.663293	0.318997
32	6	0	1.188728	0.217964	0.259551
33	1	0	-0.076004	-0.669562	-2.255228
34	8	0	0.505363	-0.904041	-0.293682
35	1	0	0.950261	2.220889	-0.174661
36	1	0	0.830991	0.366227	1.288209

Structure 120e (M06-2X, Gas Phase)

Energy (Hartrees): = - 936.3522103
No imaginary frequencies

Standard orientation:

Center Number	Atomic Number	Atomic Type	Coordinates (Angstroms) X	Y	Z
1	6	0	0.948389	-1.630049	-1.136522
2	6	0	0.486314	-1.913939	0.305642
3	6	0	0.621081	-0.687536	1.206135
4	6	0	1.946671	-0.002893	0.971706
5	6	0	2.014121	0.399932	-0.497476
6	1	0	-0.161466	0.041513	0.966828
7	1	0	2.771627	-0.700399	1.181048
8	1	0	1.135764	1.006198	-0.752962
9	1	0	1.043492	-2.735063	0.760152
10	8	0	2.041064	-0.791154	-1.278118
11	6	0	3.267810	1.192972	-0.840032
12	1	0	4.146716	0.668184	-0.439434
13	1	0	3.362055	1.229166	-1.926284
14	8	0	3.185384	2.518396	-0.374852
15	1	0	2.979641	2.472724	0.565364
16	8	0	2.058785	1.153033	1.778844
17	1	0	1.767120	0.906403	2.663358
18	8	0	0.543211	-1.040003	2.575784
19	1	0	-0.355471	-1.340259	2.742267
20	7	0	-0.925455	-2.267767	0.141059
21	6	0	-2.112862	-0.192243	-0.546423
22	6	0	-1.915475	1.022938	-1.196984
23	6	0	-3.025659	-0.271656	0.504056
24	6	0	-2.616991	2.153180	-0.788479
25	1	0	-1.202673	1.078099	-2.010440
26	6	0	-3.727369	0.857699	0.907898
27	1	0	-3.165177	-1.219510	1.011188

28	6	0	-3.522334	2.073913	0.263248
29	1	0	-2.451176	3.098576	-1.291157
30	1	0	-4.431331	0.790408	1.729036
31	1	0	-4.065006	2.956156	0.581098
32	6	0	-1.368547	-1.439292	-0.990245
33	1	0	1.229406	-2.551585	-1.655465
34	8	0	-0.212478	-1.080530	-1.744056
35	1	0	-0.995946	-3.244895	-0.120989
36	1	0	-2.028030	-2.037435	-1.626971

Structure 120e (M06-2X, DMSO)

Energy (Hartrees): = - 936.3768678
No imaginary frequencies

Standard orientation:

Center Number	Atomic Number	Atomic Type	Coordinates (Angstroms) X	Y	Z
1	6	0	0.936608	-1.622843	-1.147971
2	6	0	0.492546	-1.913188	0.296362
3	6	0	0.645966	-0.699347	1.210974
4	6	0	1.963196	-0.005376	0.958723
5	6	0	2.009220	0.406818	-0.508548
6	1	0	-0.144785	0.030514	1.000928
7	1	0	2.797060	-0.693454	1.158410
8	1	0	1.126523	1.015382	-0.745515
9	1	0	1.051029	-2.746485	0.724391
10	8	0	2.026561	-0.774325	-1.303357
11	6	0	3.260644	1.195571	-0.864834
12	1	0	4.145299	0.648608	-0.510825
13	1	0	3.322598	1.273502	-1.951982
14	8	0	3.217341	2.510026	-0.346932
15	1	0	3.011588	2.423472	0.591560
16	8	0	2.076625	1.156281	1.766682
17	1	0	1.831573	0.900875	2.664437
18	8	0	0.602420	-1.067479	2.579440
19	1	0	-0.288637	-1.387835	2.762354
20	7	0	-0.921003	-2.260635	0.142288
21	6	0	-2.124556	-0.188807	-0.537598
22	6	0	-1.911000	1.038857	-1.161397
23	6	0	-3.063626	-0.280913	0.490224
24	6	0	-2.621279	2.165323	-0.752144
25	1	0	-1.180405	1.112173	-1.957711
26	6	0	-3.773279	0.843702	0.897055
27	1	0	-3.223546	-1.235521	0.979213
28	6	0	-3.552266	2.071748	0.277283
29	1	0	-2.444229	3.118151	-1.238470
30	1	0	-4.496940	0.762508	1.700374
31	1	0	-4.102583	2.949829	0.595628
32	6	0	-1.378378	-1.436452	-0.981678
33	1	0	1.214361	-2.544157	-1.666546
34	8	0	-0.228296	-1.071587	-1.749187
35	1	0	-0.990419	-3.237625	-0.125945
36	1	0	-2.041445	-2.039387	-1.608189

Structure 120a (M06-2X, Gas Phase)

Energy (Hartrees): = - 936.3448573
No imaginary frequencies

Standard orientation:

Center Number	Atomic Number	Atomic Type	Coordinates (Angstroms) X	Y	Z
1	6	0	0.881424	1.631464	0.993089
2	6	0	0.470972	1.779219	-0.476580
3	6	0	0.732057	0.476779	-1.236792
4	6	0	2.124030	-0.031199	-0.928428
5	6	0	2.234850	-0.278325	0.571318
6	1	0	0.020038	-0.293772	-0.916360
7	1	0	2.862013	0.735352	-1.210383
8	1	0	1.463092	-0.992891	0.882212
9	1	0	1.014518	2.591829	-0.965176
10	8	0	2.064142	0.966227	1.243770

11	6	0	3.594654	-0.826540	0.981853
12	1	0	4.382475	-0.203127	0.535943
13	1	0	3.678669	-0.752261	2.066929
14	8	0	3.732754	-2.183279	0.635620
15	1	0	3.537478	-2.255864	-0.305059
16	8	0	2.379084	-1.239883	-1.616753
17	1	0	2.115203	-1.093630	-2.531831
18	8	0	0.652009	0.654661	-2.647501
19	1	0	-0.254054	0.518216	-2.931718
20	7	0	-0.935573	2.120883	-0.304143
21	6	0	-2.298042	0.189757	0.549497
22	6	0	-1.943457	-1.121593	0.852223
23	6	0	-3.511431	0.442677	-0.091500
24	6	0	-2.786129	-2.170774	0.496327
25	1	0	-1.011025	-1.308754	1.370418
26	6	0	-4.350137	-0.605552	-0.449494
27	1	0	-3.805252	1.467162	-0.303122
28	6	0	-3.985726	-1.916873	-0.158244
29	1	0	-2.503936	-3.189452	0.735019
30	1	0	-5.290205	-0.400283	-0.947594
31	1	0	-4.638809	-2.736226	-0.433323
32	6	0	-1.396565	1.359924	0.887399
33	1	0	0.977376	2.607419	1.476386
34	8	0	-0.224545	0.921117	1.539596
35	1	0	-1.488272	1.885079	-1.120930
36	1	0	-1.936854	2.050507	1.545733

Structure 120a (M06-2X, DMSO)

Energy (Hartrees): = - 936.3706688
No imaginary frequencies

Standard orientation:

Center Number	Atomic Number	Atomic Type	Coordinates (Angstroms) X	Y	Z
1	6	0	0.813021	1.503627	1.085957
2	6	0	0.510905	1.752447	-0.397759
3	6	0	0.909186	0.542296	-1.247626
4	6	0	2.281581	0.045791	-0.846380
5	6	0	2.267897	-0.319716	0.631935
6	1	0	0.195099	-0.274934	-1.085289
7	1	0	3.020445	0.844818	-1.002910
8	1	0	1.493415	-1.074609	0.815363
9	1	0	1.049374	2.631690	-0.760732
10	8	0	2.000589	0.862551	1.383933
11	6	0	3.605980	-0.854560	1.120770
12	1	0	4.402638	-0.159698	0.821542
13	1	0	3.585441	-0.898026	2.211404
14	8	0	3.851080	-2.163981	0.649201
15	1	0	3.713517	-2.138915	-0.305302
16	8	0	2.633916	-1.105226	-1.596976
17	1	0	2.461687	-0.895118	-2.523268
18	8	0	0.980582	0.857602	-2.632754
19	1	0	0.093034	0.826826	-3.005632
20	7	0	-0.930880	1.995572	-0.335959
21	6	0	-2.411028	0.151151	0.488927
22	6	0	-2.028445	-1.188145	0.452710
23	6	0	-3.716376	0.505263	0.146480
24	6	0	-2.944360	-2.164042	0.069060
25	1	0	-1.017817	-1.461389	0.733391
26	6	0	-4.628032	-0.469567	-0.244271
27	1	0	-4.016892	1.548085	0.187865
28	6	0	-4.242555	-1.807250	-0.283934
29	1	0	-2.642953	-3.205341	0.047914
30	1	0	-5.640163	-0.187414	-0.511137
31	1	0	-4.953105	-2.569070	-0.583561
32	6	0	-1.434646	1.245521	0.849196
33	1	0	0.829272	2.444593	1.644190
34	8	0	-0.295324	0.712614	1.493282
35	1	0	-1.398144	1.674983	-1.178683
36	1	0	-1.924194	1.959150	1.522672

Structure 108β (B3LYP, Gas Phase)

Energy (Hartrees): = - 1622.3665917
No imaginary frequencies

Standard orientation:

Center Number	Atomic Number	Atomic Type	Coordinates (Angstroms) X	Y	Z
1	6	0	0.159070	1.324978	-0.327523
2	6	0	-0.592288	0.004664	-0.578659
3	6	0	0.289553	-1.139902	-0.037901
4	6	0	1.710221	-1.070029	-0.620640
5	6	0	2.309576	0.338901	-0.417200
6	1	0	0.278945	1.527748	0.743996
7	1	0	0.348448	-1.044160	1.046143
8	1	0	1.660865	-1.277217	-1.691035
9	1	0	2.448510	0.524124	0.654330
10	1	0	-0.715833	-0.097313	-1.663134
11	8	0	1.413420	1.300814	-0.975363
12	6	0	3.642796	0.478466	-1.146636
13	1	0	4.185018	-0.467431	-1.123883
14	1	0	3.456220	0.787257	-2.176931
15	8	0	4.462301	1.512100	-0.571351
16	8	0	2.584244	-2.103502	-0.116397
17	8	0	-0.309361	-2.445184	-0.206527
18	8	0	-0.587267	2.362438	-0.930617
19	7	0	-1.863379	0.012680	0.119164
20	6	0	-4.266082	0.011433	0.031060
21	6	0	-4.422570	0.078025	1.443512
22	6	0	-5.414221	-0.024387	-0.783703
23	6	0	-5.715142	0.108295	1.990896
24	6	0	-6.686982	0.005390	-0.235458
25	1	0	-5.283691	-0.075293	-1.862229
26	6	0	-6.826690	0.072459	1.159558
27	1	0	-5.813460	0.159803	3.069923
28	1	0	-7.562940	-0.022321	-0.874825
29	1	0	-7.819391	0.096754	1.600240
30	6	0	-2.947087	-0.016977	-0.575610
31	1	0	-2.912261	-0.065831	-1.672879
32	8	0	-3.371354	0.113252	2.273258
33	1	0	-2.548960	0.083027	1.712297
34	6	0	-0.569771	-2.933467	-1.442292
35	6	0	-1.178474	-4.310852	-1.340464
36	1	0	-1.396886	-4.684888	-2.339948
37	1	0	-0.485900	-4.986673	-0.830602
38	1	0	-2.094029	-4.272208	-0.743480
39	8	0	-0.346012	-2.343675	-2.479031
40	6	0	2.886847	-2.160936	1.212531
41	6	0	3.959805	-3.186438	1.463685
42	1	0	3.923546	-3.499205	2.507198
43	1	0	3.859165	-4.043750	0.795786
44	1	0	4.924753	-2.705818	1.271266
45	8	0	2.379785	-1.457222	2.057810
46	6	0	5.291224	1.126627	0.430638
47	6	0	6.065935	2.304096	0.965159
48	1	0	6.806413	1.955842	1.684151
49	1	0	6.551358	2.843272	0.147584
50	1	0	5.378051	3.001386	1.453324
51	8	0	5.378223	-0.014683	0.833198
52	6	0	-0.459270	3.615708	-0.384807
53	6	0	-1.272893	4.606916	-1.175104
54	1	0	-0.913169	4.641058	-2.207637
55	1	0	-2.320371	4.293507	-1.203357
56	1	0	-1.188228	5.591712	-0.717636
57	8	0	0.208038	3.856916	0.590137
